# Combined Theoretical and Experimental Studies Unravel Multiple Pathways to Convergent Asymmetric Hydrogenation of Enamides

**DOI:** 10.1021/jacs.1c09573

**Published:** 2021-12-14

**Authors:** Jianping Yang, Luca Massaro, Suppachai Krajangsri, Thishana Singh, Hao Su, Emanuele Silvi, Sudipta Ponra, Lars Eriksson, Mårten S. G. Ahlquist, Pher G. Andersson

**Affiliations:** † Department of Organic Chemistry, Stockholm University, Arrhenius Laboratory, 106 91 Stockholm, Sweden; ‡ School of Chemistry and Physics, University of Kwazulu-Natal, Private Bag X54001, Durban 4000, South Africa; § School of Biotechnology, KTH Royal Institute of Technology, 106 91 Stockholm, Sweden; ∥ Department of Materials and Environmental Chemistry, Stockholm University, Svante Arrhenius väg 16C, 106 91 Stockholm, Sweden

## Abstract

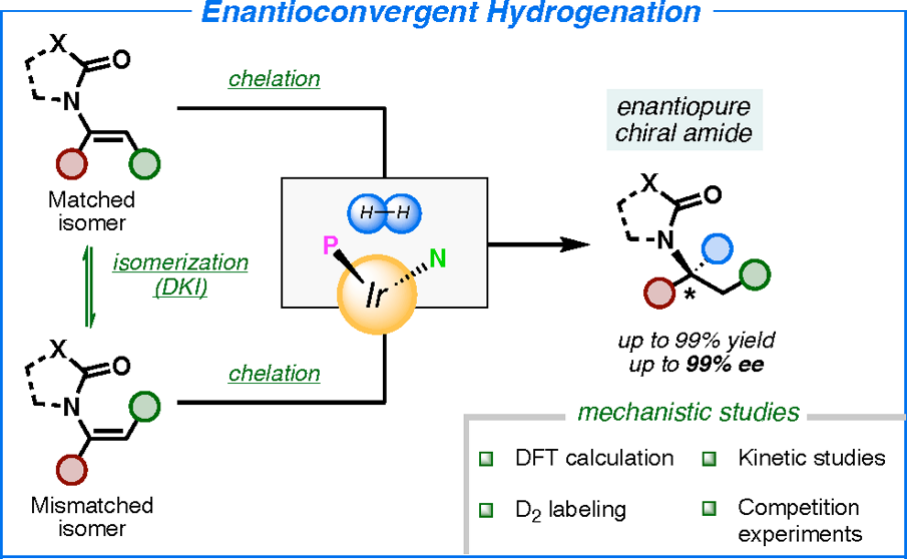

We present a highly efficient convergent asymmetric hydrogenation of *E*/*Z* mixtures of enamides catalyzed by N,P–iridium complexes supported by mechanistic studies. It was found that reduction of the olefinic isomers (*E* and *Z* geometries) produces chiral amides with the same absolute configuration (enantioconvergent hydrogenation). This allowed the hydrogenation of a wide range of *E*/*Z* mixtures of trisubstituted enamides with excellent enantioselectivity (up to 99% *ee*). A detailed mechanistic study using deuterium labeling and kinetic experiments revealed two different pathways for the observed enantioconvergence. For α-aryl enamides, fast isomerization of the double bond takes place, and the overall process results in kinetic resolution of the two isomers. For α-alkyl enamides, no double bond isomerization is detected, and competition experiments suggested that substrate chelation is responsible for the enantioconvergent stereochemical outcome. DFT calculations were performed to predict the correct absolute configuration of the products and strengthen the proposed mechanism of the iridium-catalyzed isomerization pathway.

## Introduction

The asymmetric hydrogenation of prochiral olefins is one of the most practical and efficient transformations for the preparation of enantiopure compounds.^1^ A wide number of Rh(I), Ru(II), and Ir(I) catalytic systems have been extensively studied and applied to diversely functionalized olefins.^2^

**Scheme 1:**
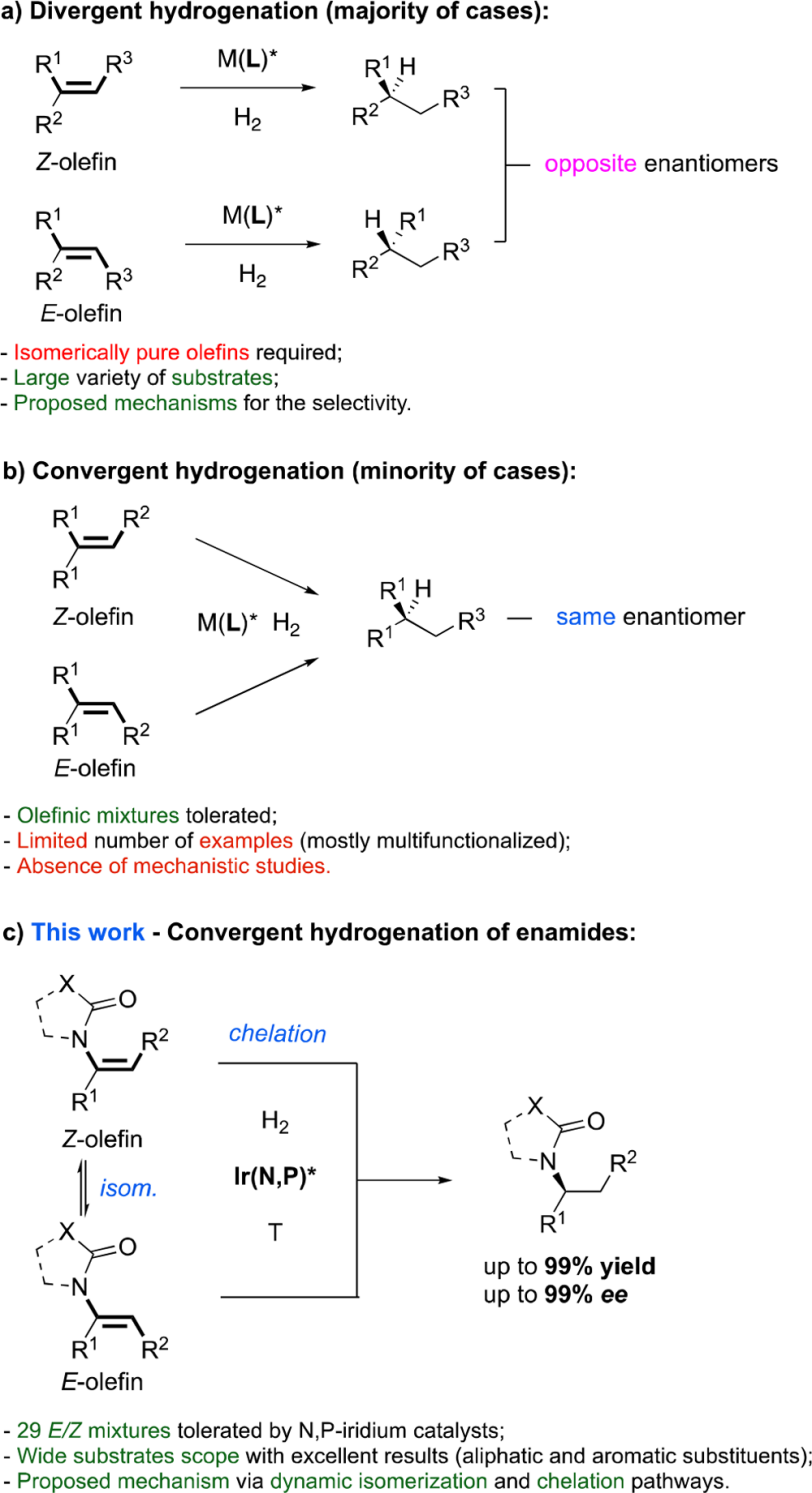
Enantiodivergent and Enantioconvergent Hydrogenation

Despite the successful results and considerable mechanistic understanding in this field, several challenges still remain. In the asymmetric hydrogenation of trisubstituted olefins, the *E* and *Z* geometries of the substrate generally produce opposite enantiomers of the products (Scheme 1a, divergent hydrogenation).^3^ This limits the possibility of achieving high stereoselectivity in the reduction of isomeric mixtures, which leads to a difficult and time-consuming purification of the olefinic substrates. Therefore, catalytic systems that can directly hydrogenate *E*/*Z* mixtures to yield enantiomerically pure products are highly desired. Unfortunately, only a few catalysts have been reported to efficiently transform both *E* and *Z* isomers into the same enantiomer of the product with equally high enantioselectivity (Scheme 1b, convergent hydrogenation). From a mechanistic point, the literature reports several proposals that are used to rationalize the stereochemical outcome of the hydrogenation, especially for catalytic systems having rhodium, ruthenium, and iridium complexes.^4^ Detailed mechanistic insight is required to identify what clearly distinguishes these two opposite enantioselective behaviors (divergence and convergence) and to understand whether the differences lie in the catalyst properties or the nature of the substrates. The divergent outcome has been rationalized and demonstrated in several cases.^4c,5^ However, to the best of our knowledge, studies to elucidate the enantioconvergent outcome have rarely been reported.^2a^

Historically, enamides have received much attention as strategic starting materials to synthesize valuable chiral amines.^6^ The limitation of many of the reported methodologies is related to the geometry of the starting materials (Scheme 1a).^3b,3h,7^ An exception is the hydrogenation of multifunctionalized enamides, such as α-dehydroamino acids, using Rh–DuPHOS catalysts, which reduced the *E* and *Z* isomers to the same enantiopure products (Scheme 1b).^8^ Recent catalytic systems based on BINAPO–Ru and Ni–Binapine also showed excellent convergence for the hydrogenation of amino acid precursors.^9^ Mechanistic studies on these systems excluded the presence of double-bond isomerization, but in-depth mechanistic studies were not presented.

**Figure 1:**
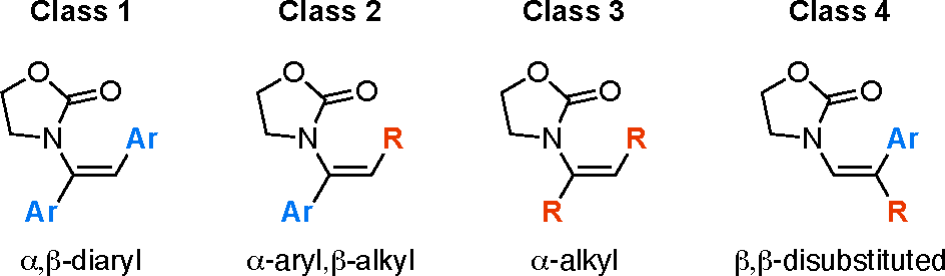
Classes of enamides.

We decided to investigate the asymmetric hydrogenation of simple trisubstituted enamides with our N,P–iridium complexes, since this class of catalysts have often shown a strong dependence on the double-bond geometry. During the optimization of the reaction conditions, we surprisingly found that both the *E* and *Z* isomers were converted to the same enantiomer with high optical purity (Scheme 1c). Intrigued by these enantioconvergent results, we examined four different classes of enamides, defined by different geometric and electronic properties (Figure 1). Each class was evaluated against a large number of substrates, and in-depth mechanistic studies were carried out to elucidate the origin of this unusual selectivity. The optimized results and catalyst structures for each class are presented in Table 2 and Scheme 7.

## Results and Discussion

### Mechanistic Investigation

#### Class 1

**Scheme 2:**
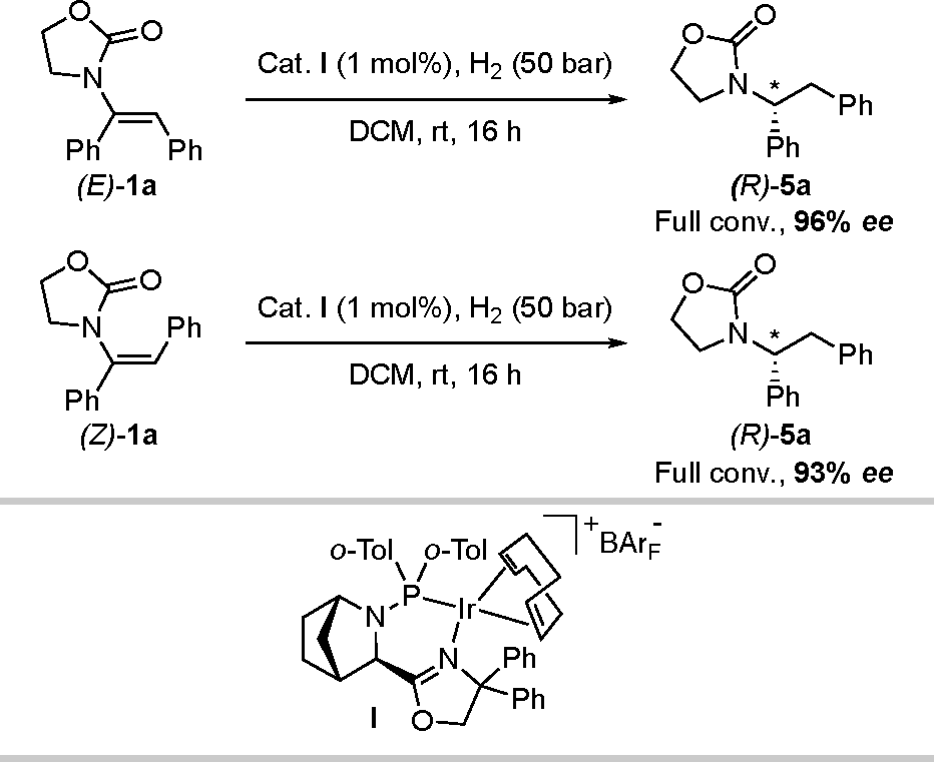
Hydrogenation of Class 1 Enamides

**Scheme 3:**
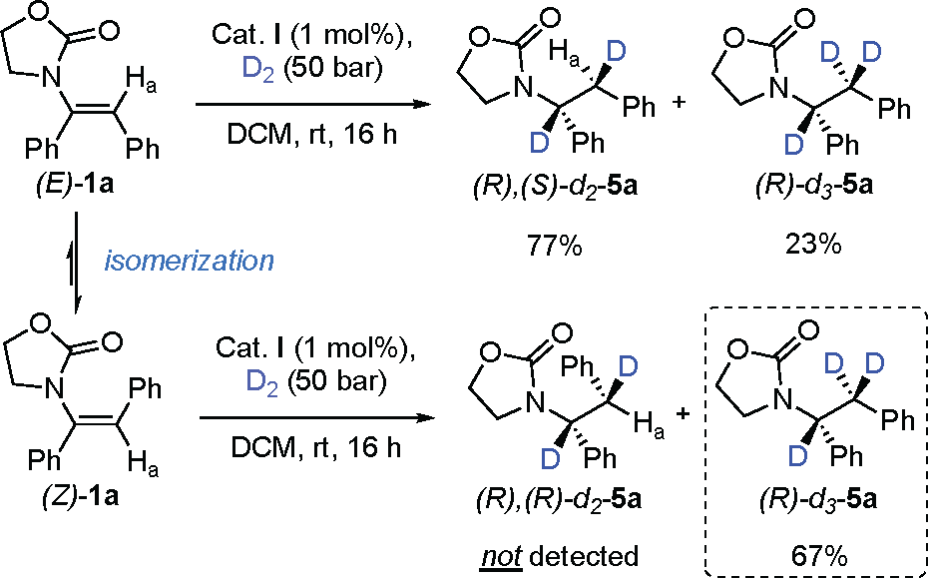
Deuterium Labeling Experiments for Class 1

**Figure 2:**
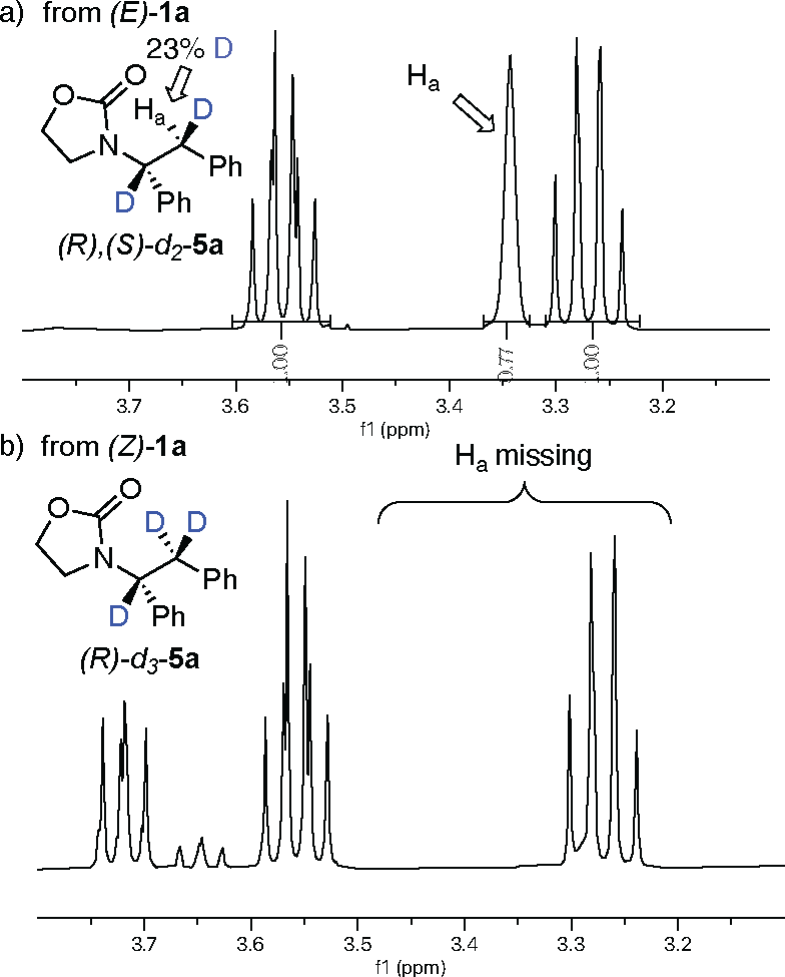
^1^H NMR spectra for the deuterium experiments. (a) For (*E*)-**1a**, H_a_ of the product at δ 3.34 integrates to 0.77. (b) (*Z*)-**1a** shows the presence of product *d*_3_-**5a** without the benzylic proton. The **5a**/*Z*/*E* ratio is 12:5:1.

**Figure 3:**
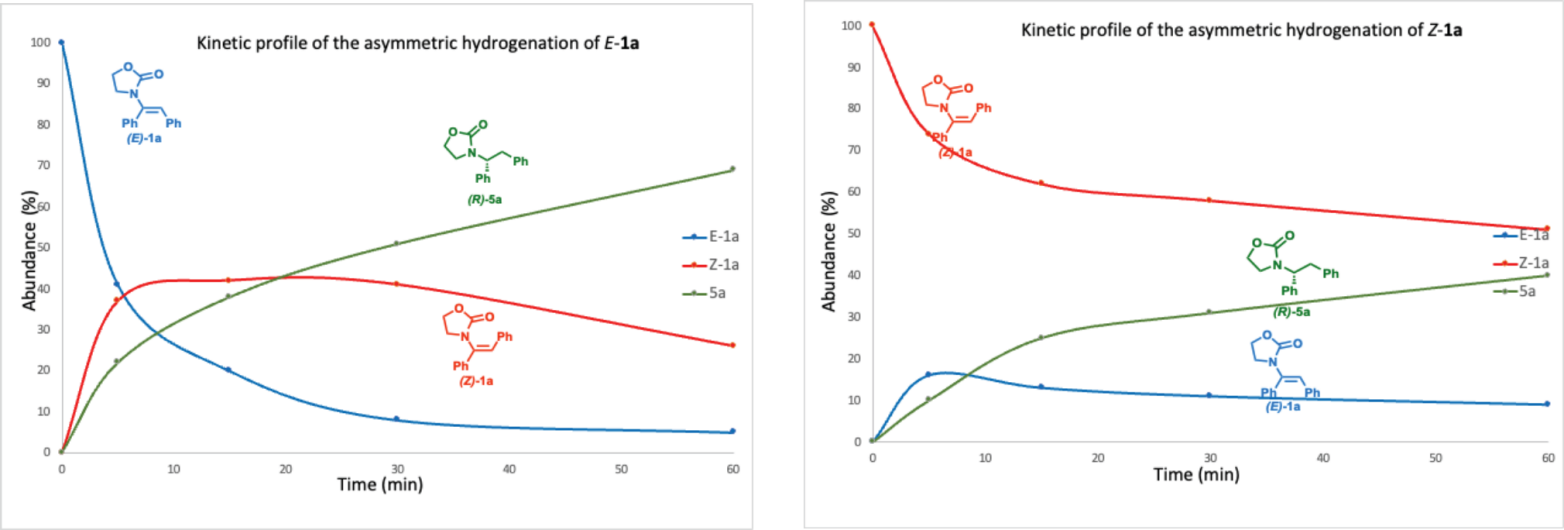
Kinetic profiles for hydrogenation of (a) *E*-**1a** and (b) *Z*-**1a**.

The *E* and *Z* isomers of α,β-diphenyl-substituted enamide **1a** were used as model substrates for class 1 (Scheme 2). Under the optimized reaction conditions (for optimization details, see Tables S1–S5), both the *E* and *Z* isomers of **1a** gave the *R* enantiomer with high selectivity (96% and 93% *ee*, respectively). To shed light on the enantioselective outcome of this transformation, hydrogenations were performed using D_2_ gas (Scheme 3). We hypothesized that a stereospecific cis addition of D_2_ to the *E* and *Z* enamide carbon–carbon double bond would generate a pair of diastereomeric products unless isomerization occurs, which would instead result in the formation of a single diastereomer.^10^ The *E* isomer was examined first and gave full conversion to the expected deuterated product *d*_2_-**5a**, showing the signal of proton H_a_ as a singlet at 3.34 ppm (Figure 2a; the complete spectrum is given in Figure S2). However, the *Z* isomer surprisingly resulted in trideuterated *d*_3_-**5a** as the major product. The signal of the benzylic proton H_a_ disappeared because H_a_ was completely exchanged with a deuterium atom, and in addition, some remaining starting material (*Z* isomer) and the formation of the *E* isomer were detected from the residual oxazolidinone peaks (Figure 2b). These results could be explained by an isomerization of the double bond in which the catalyst exchanges the vinylic hydrogen with a deuterium atom.^11^ Next, we investigated the kinetic profile for the hydrogenation of the two isomers (*E*)-**1a** and (*Z*)-**1a** (Figure 3). In both cases, the formation of the other isomer could be detected by NMR spectroscopy *before* complete conversion to the reduced products. Moreover, thermodynamic equilibrium between the two isomers of the starting material was achieved in less than 60 min.^12^ The reaction starting from the *E* isomer gave a 69% yield of the product **5a**, while the one starting from the *Z* isomer produced only a 40% yield of the hydrogenated product over 1 h. In both cases the isomeric ratio of **1a** in the remaining reaction mixture was in favor of the thermodynamically more stable but less reactive *Z* isomer. This is also in agreement with the deuterium experiment, in which (*Z*)-**1a** is not hydrogenated directly but instead undergoes an isomerization involving the complete exchange of the benzylic proton (Figure 3 and Table S6).

**Figure 4:**
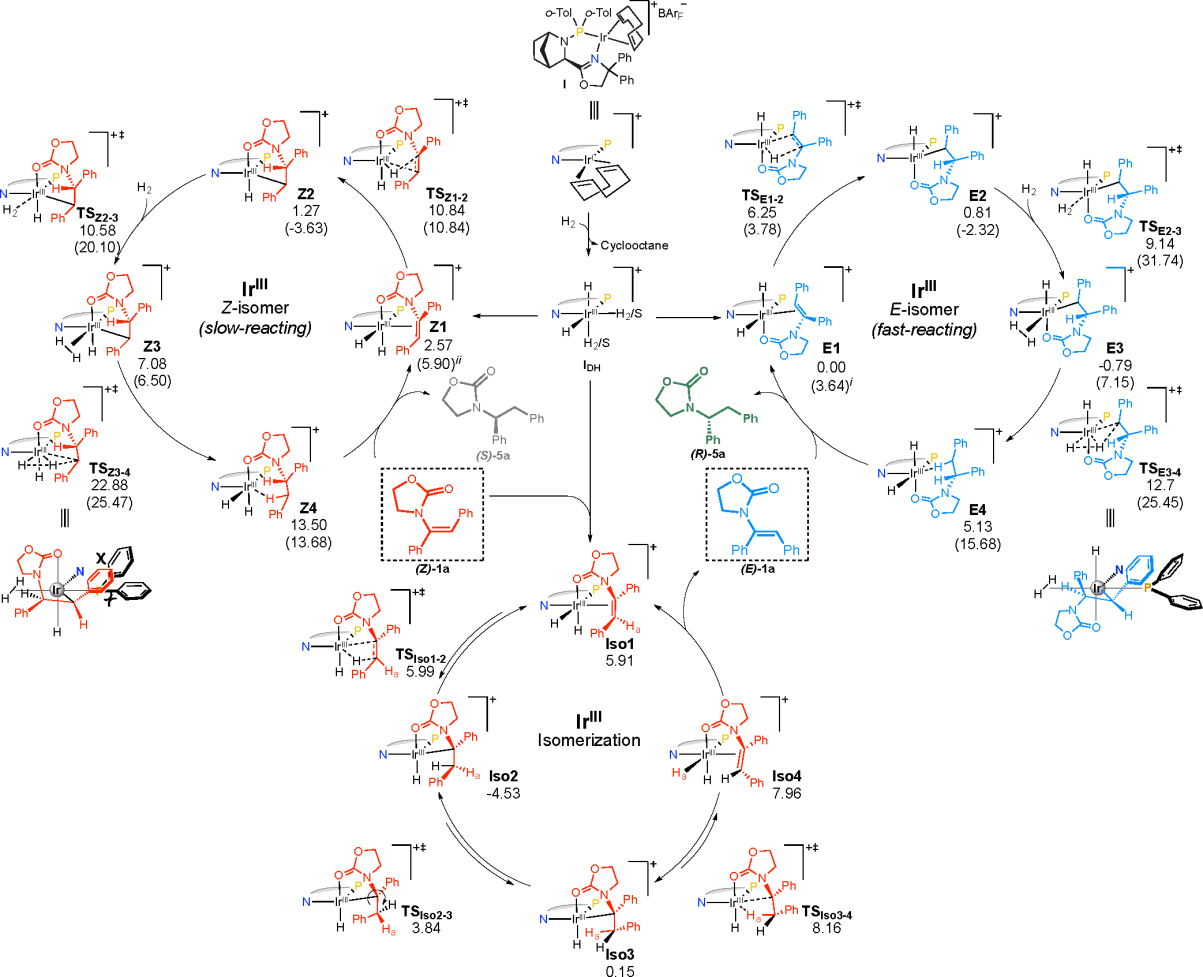
DFT-calculated free energy profile for the hydrogenation of class 1 enamides: (right) *E* isomer; (left) *Z* isomer; (bottom) reversible isomerization pathway. ^*i*^The free energy of the pathway toward the *S* configuration is shown in parentheses. ^*ii*^The free energy of the pathway toward the *R* configuration is shown in parentheses.

These data suggest that the reduced product is generated via the less abundant diastereoisomer present in the reaction (the *E* isomer).^13^ The thermodynamically stable but slow-reacting *Z* isomer instead isomerizes to the fast-reacting *E* isomer via reversible migratory insertion/β-hydride elimination and is consequently hydrogenated. DFT calculations for the asymmetric hydrogenation of the two isomers and for the isomerization process were carried out to support this assumption (Figure 4). The dihydride species **I**_**DH**_ (Figure 4, center) is the starting point for all of the reactions.^14^ We considered various starting geometries in which the iridium dihydride coordinates exclusively to the enamide double bond (Figure S6), but these complexes resulted in much higher energies than the chelated species, where both the carbonyl group and the double bond are coordinated. Moreover, chelation trans to nitrogen resulted in the most reasonable energies, which is in agreement with previously reported studies of the hydrogenation of functionalized olefins.^15^ The calculated pathways with the lowest energy are presented for both (*Z*)-**1a** (Figures 4, left, and S7) and (*E*)-**1a** (Figures 4, right, and S8). The migratory insertion barrier revealed a Δ*E* of ∼2 kcal mol^–1^ in favor of the *E* isomer (**TS**_**Z1–2**_ vs **TS**_**E1–2**_; Figure S9). The catalytic cycles then continue with coordination of a new dihydrogen molecule to form the respective intermediates **Z3** and **E3**. To conclude, σ-complex-assisted metathesis releases the respective products in the rate-determining step. Here as well, the Δ*E* barrier favors the *E* isomer route (∼2.3 kcal mol^–1^; Figure S9), which generates product **5a** with the correct *R* configuration in agreement with the experimental results. Interestingly, the two favored mechanistic pathways related to the *E* and *Z* geometries would lead, as often reported, to opposite enantiomers.^3^ The convergent outcome is enabled by an isomerization and involves another migratory insertion step in which the iridium atom coordinates to the more hindered prochiral carbon of the *Z* isomer (**Iso2**; Figure 4, bottom). Notably, this process is almost barrierless (**TS**_**Iso1–2**_, 0.09 kcal mol^–1^). Rotation of the C–C single bond followed by β-hydride elimination (**TS**_**Iso3–4**_) forms the *E* isomer. The energy barrier for the described steps is lower than 13 kcal mol^–1^, suggesting that this process is faster than the hydrogenation rate-determining step (Δ*G*_**TS_Z3-4_**–**Z3**_ = 15.8 kcal mol^–1^ and Δ*G*_**TS_E3-4_**–**E3**_ = 13.49 kcal mol^–1^; Figure S9). These calculations correspond with the experimental results, supporting the iridium-catalyzed dynamic isomerization as the mechanistic reason for the enantioconvergent hydrogenation and confirming the presence of fast- and slow-reacting isomers.

#### Class 2

We then turned our attention to the second class of enamides, which have an aliphatic chain as the β-substituent (Table 1). The hydrogenation of the *E* and *Z* isomers of compound **2a** using the standard reaction conditions and thiazole-based catalyst **II** yielded considerably different results. While the *Z* isomer gave 99% *ee* favoring the *R* enantiomer (Table 1, entry 1), the *E* isomer had a modest *ee* of 38% with the opposite configuration (Table 1, entry 2). Intrigued by these results, we re-evaluated the reaction conditions, which revealed that the stereochemical outcome of the reaction is strongly dependent on both the temperature and the hydrogen pressure (Table S4). Indeed, with 1 bar H_2_ and an increased temperature of 60 °C, the enantioselective outcome for *E* isomer of **2a** shifted to favor the *R* enantiomer with 80% *ee* (Table 1, entry 4). For the *Z* isomer of **2a**, these reaction conditions had a negligible effect, since the change was from 99% to 97% *ee* in favor of the *R* enantiomer (Table 1, entry 3). The enantioconvergent results obtained using the new reaction conditions are in accordance with the proposed mechanism for class 1 enamides, as the elevated temperature increases the rate of the isomerization process and the lower hydrogen pressure retards the hydrogenation.^4b^ We began deuterium experiments on the *E* and *Z* isomers of **2a** using a pressure of 50 bar at room temperature (Figure S3). However, when the modified reaction conditions were used (i.e., higher temperature and lower pressure), deuterium exchange occurred, resembling that of the class 1 enamides. Here, the *E* isomer showed deuterium exchange of H_a_ (Table 1, entry 4), while the *Z* isomer was completely converted to product *d*_2_-**6a** and no proton exchange was detected (Table 1, entry 3, and Figure S4).

**Table 1:**
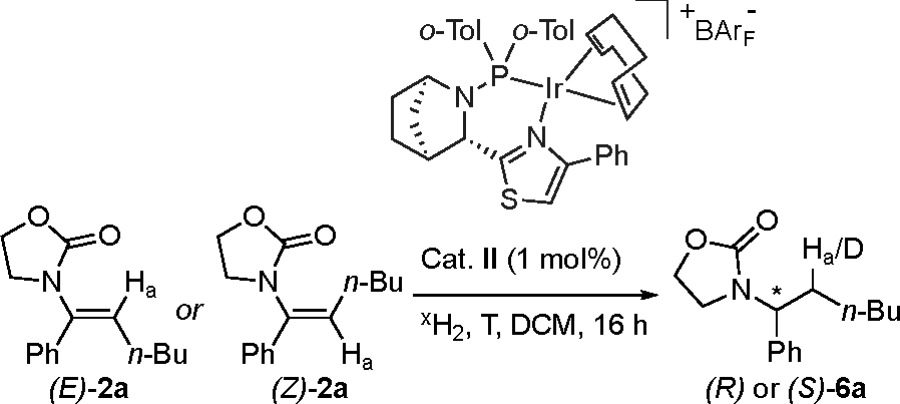
Class 2 Optimization of Enantioconvergence^a^

entry	isomer	H_2_ or D_2_ pressure (bar)	temp.	conv.	*ee* (%)	H_a_/D exchange (%)
1	*Z*	50	rt	full	99 (*R*)	<5
2	*E*	50	rt	full	38 (*S*)	<5
3^b^	*Z*	1	60 °C	full	97 (*R*)	<5
4^b^	*E*	1	60 °C	95%	80 (*R*)	42

a Reaction conditions: 0.05 mmol of substrates in 0.5 mL of DCM. Hydrogenation and deuterium labeling studies were carried out following the same protocol.

b Dichloroethane was used as the solvent.

**Scheme 4:**
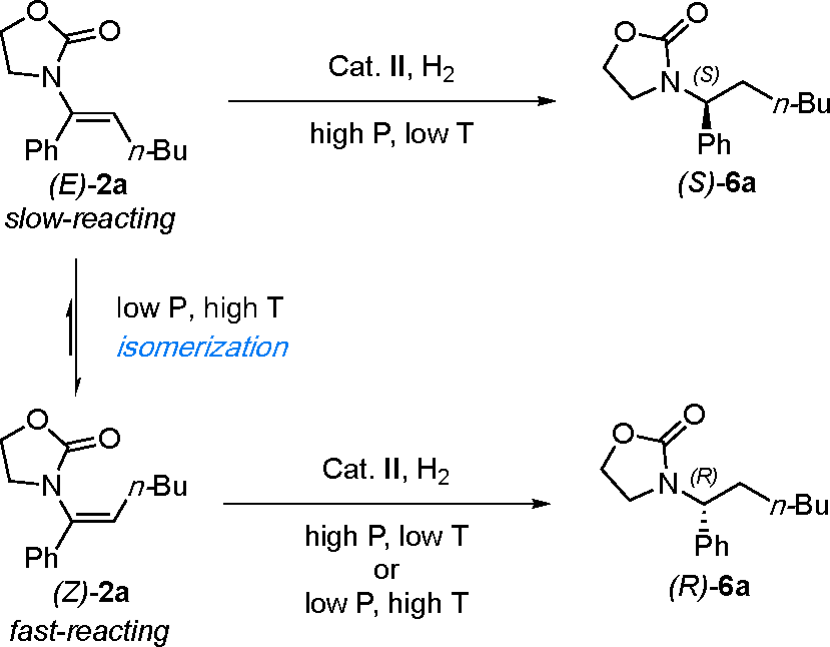
Hydrogenation Pathways for Class 2 Enamides The enantioselective outcome for (*E*)-**2a** is dependent on the H_2_ pressure and the temperature. Low pressure and high temperature favor the isomerization toward (Z)-**2a**, enabling an enantioconvergent hydrogenation.

These data suggest an enantiodivergent outcome at room temperature, which can be changed by the use of low pressure and high temperature, favoring the isomerization of the *E* isomer to the *Z* isomer and resulting in an enantioconvergent reaction (Scheme 4, low *P* and high *T*).

#### Class 3

**Scheme 5:**
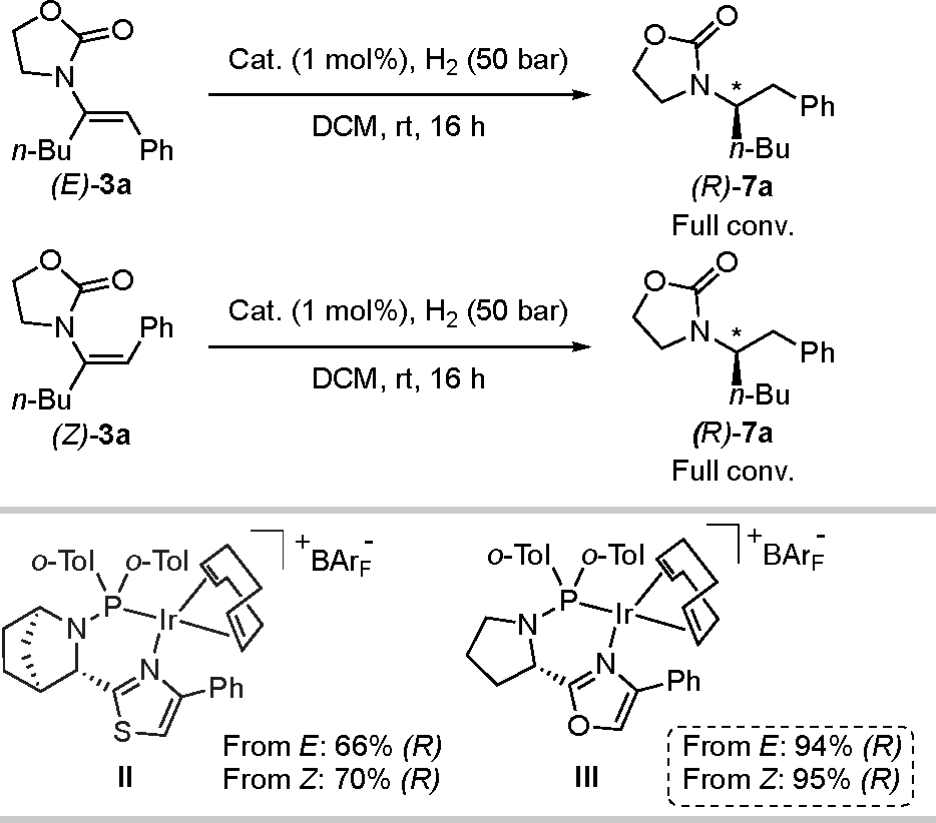
Hydrogenation of Class 3 Enamides

**Scheme 6:**
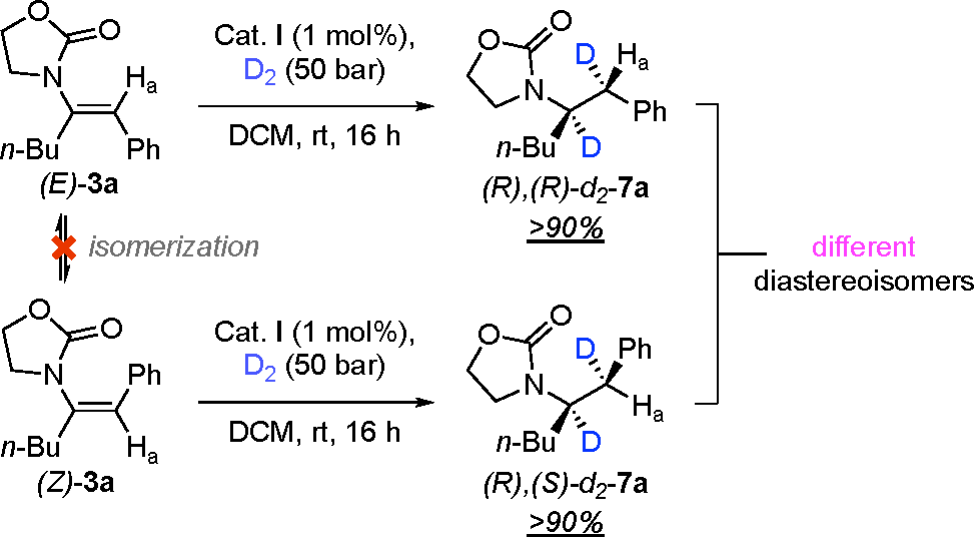
Deuterium Labeling Experiments for Class 3

**Figure 5:**
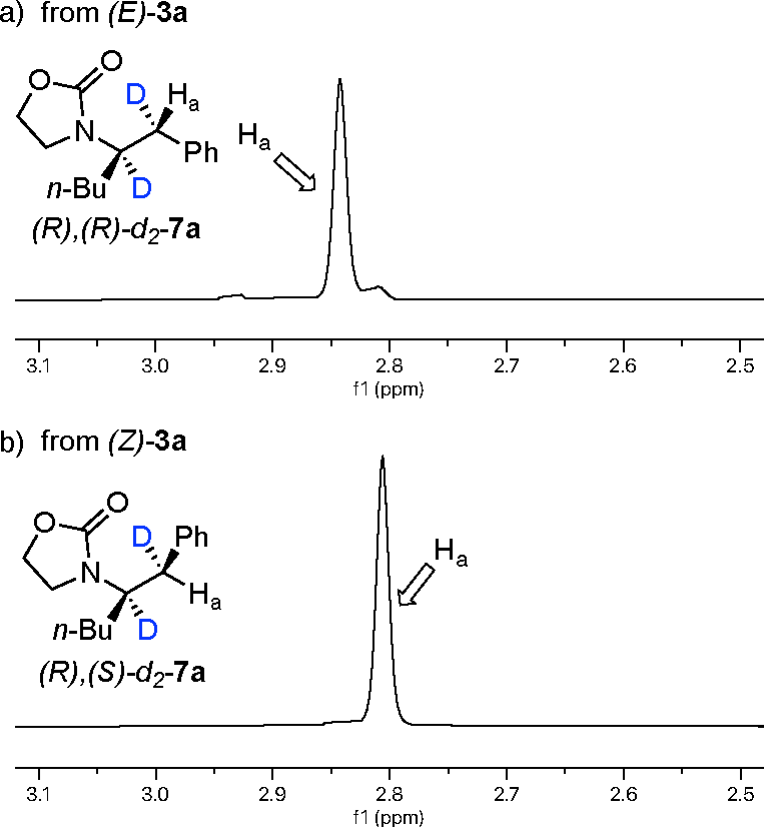
^1^H NMR spectra for the deuterium experiments (a) from (*E*)-**3a** and (b) from (*Z*)-**3a**. The two diastereoisomers can be clearly distinguished thanks to the H_a_ signal.

Enamides **3a** of class 3, which bear an aliphatic moiety at the α-position and a phenyl substituent at the β-position, were also investigated (Scheme 5). For this class, the *E* and *Z* stereoisomers again afforded convergent results under the standard reaction conditions as described for class 1. Proline-based catalyst **III**(16) gave the highest enantioselectivity for the hydrogenation of both (*E*)-**3a** and (*Z*)-**3a** (94% and 95% *ee*, respectively) and products having the same absolute configuration. Deuterium experiments indicated an absence of isomerization for both the *E* and *Z* isomers (Scheme 6 and Figure 5). A 1:1 *E*/*Z* mixture was also hydrogenated using D_2_ gas and produced an equal diastereomeric mixture of 1:1 (Figure S5).

This class of enamides, even without isomerization, achieved a high level of convergent stereoselectivity. A possible explanation for this is that the chelation-controlled hydrogenation for both the *E* and *Z* isomers leads to the same enantiomer of the product. As mentioned earlier, this is a rather uncommon observation in asymmetric hydrogenations, and to get further support for it, DFT calculations were carried out (Figures S11 and S12). Interestingly, when class 3 substrate **3b** was subjected to DFT calculations, it was found that both the *E* and *Z* isomers resulted in low-energy pathways that produce the same and correct *R* enantiomer.

#### Class 4

**Scheme 7:**
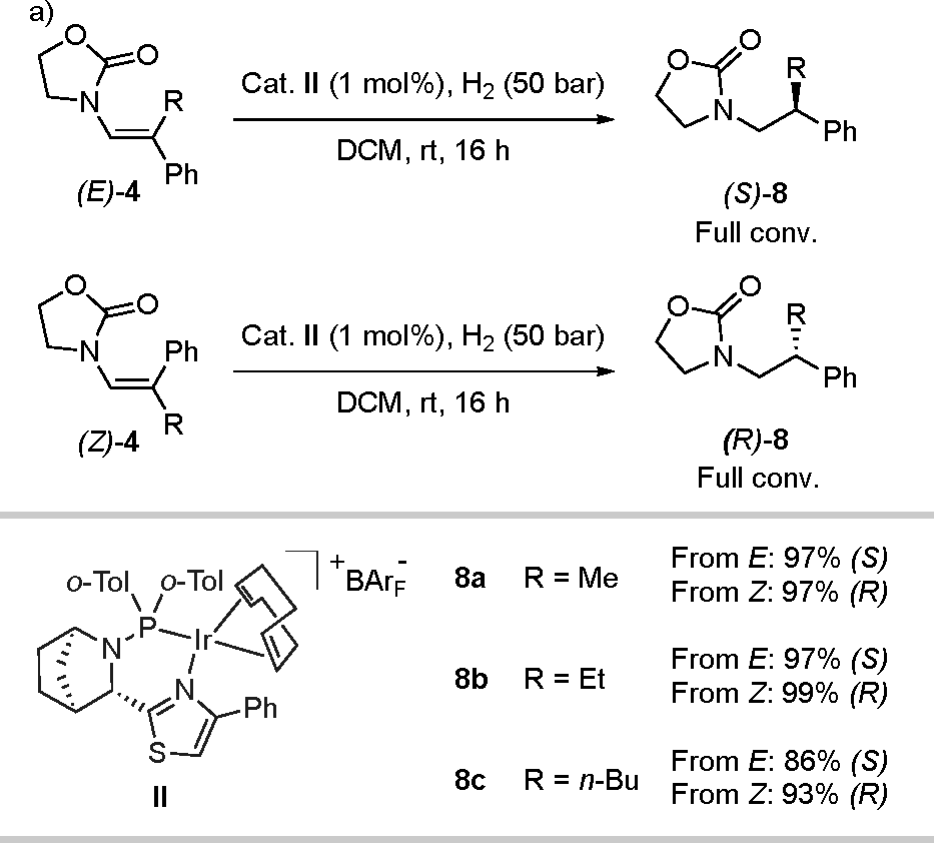
Divergent Hydrogenation of *E* and *Z* Isomers of Class 4 Enamides Reaction conditions: 0.1 mmol of substrates in 1 mL of DCM. Conversion was determined by ^1^H NMR spectroscopy. Enantiomeric excess was determined by supercritical fluid chromatography (SFC) analysis using chiral stationary phases.

Finally, the β,β-disubstituted enamides (class 4) were evaluated (Scheme 7). When the two isomers of compound **4a** were hydrogenated, the resulting products indicated that an enantiodivergent mechanism was followed. The selectivity for the *E* isomer was 97% *ee* in favor of the *S* product, whereas the *Z* isomer selectivity was 97% *ee* favoring the *R* product. The same trend was observed for substrates with longer alkyl chains, producing the opposite enantiomers for products **8b** and **8c** with good selectivity. For this class of enamides, the chelating group binds to the non-prochiral carbon, which precludes them from undergoing the same isomerization that is operative for classes 1 and 2.

### Chelation Effect

**Scheme 8:**
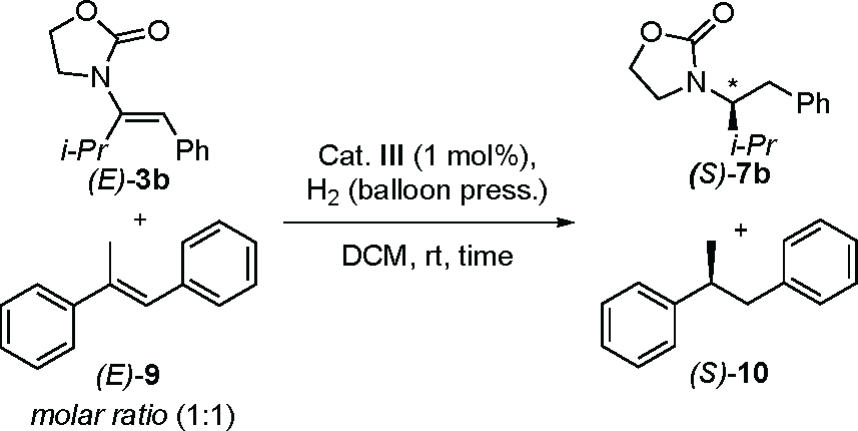
Competition Experiments

**Figure 6:**
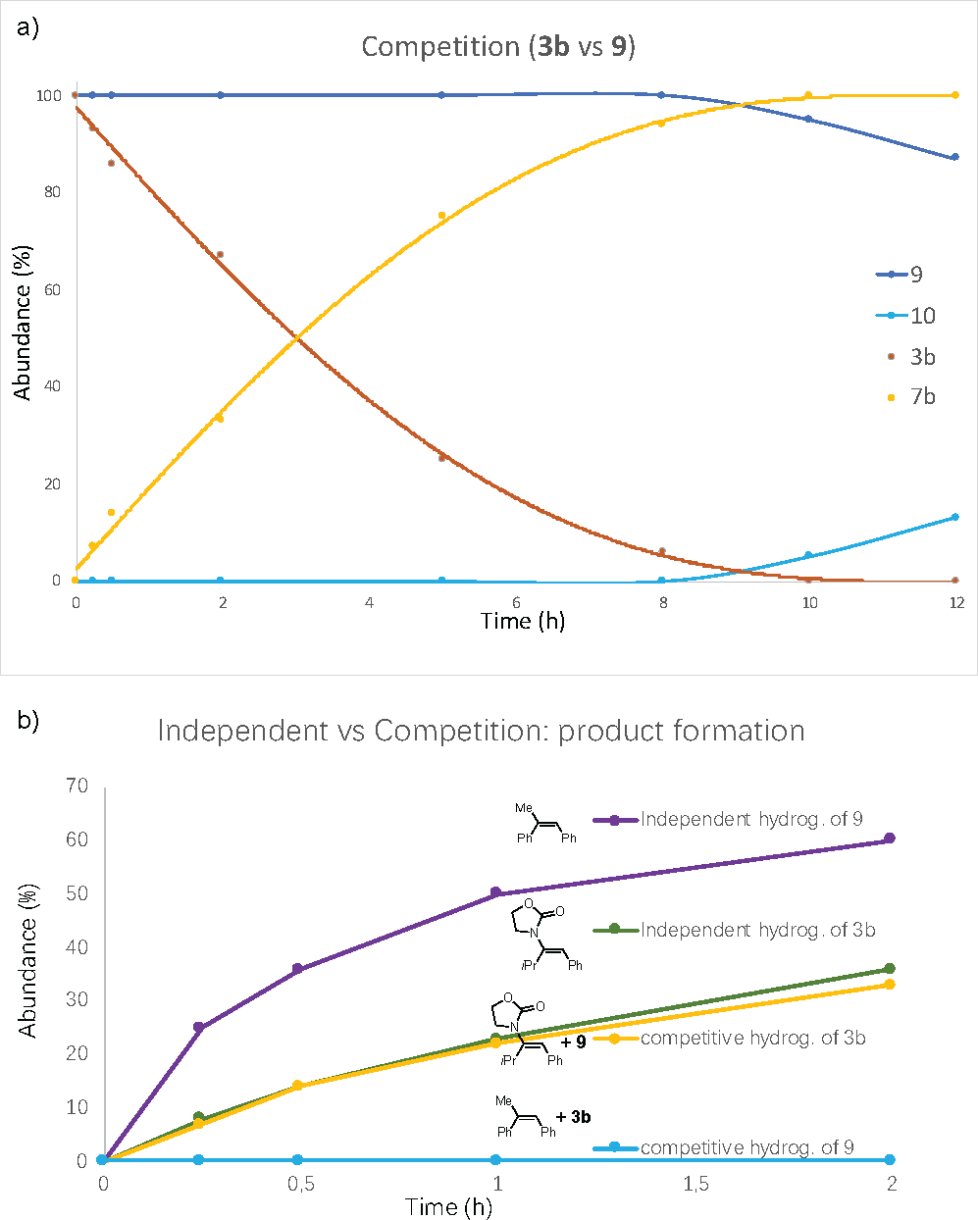
(a) Kinetic profiles for the competition experiment between enamide (*E*)***-*3b** and methylstilbene (*E*)***-*9**. (b) Kinetic profiles for independent and competitive hydrogenation of (*E*)-**3b** and (*E*)***-*9**.

As mentioned above, the different classes can undergo convergent hydrogenation either because of isomerization toward the fast-reacting isomer or simply because the *E* and *Z* isomers are reduced to the same enantiomer with favorable energies. Regardless of these mechanistic differences, DFT calculations performed for classes 1 and 3 unanimously showed that the carbonyl coordinates to iridium in the hydrogenation process (see Figure 4 for class 1 and Figures S11 and S12 for class 3). This observation is in stark contrast with the mechanism normally associated with asymmetric hydrogenation of olefins using N,P–iridium complexes. We hypothesized that chelation of the amide group would result in a chemoselective hydrogenation of the enamide in the presence of a simple olefin. A competition experiment was carried out in which an equimolar mixture of *trans*-methylstilbene (*E*)***-*9** and enamide (*E*)-**3b** was subjected to hydrogenation (Scheme 8). The reaction was monitored over time, and it was found that the enamide was consumed over 9 h and that no conversion of **9** was observed *before***3b** had been consumed (Figure 6a). Interestingly, the independent hydrogenation of *trans*-methylstilbene with N,P–iridium complex **III** showed a reversed order of reactivity, with more than 60% conversion in 2 h (Figure 6b) versus 35% conversion of the amide. The same competition experiment was also performed for hydrogenation of class 1 enamides and resulted in the same outcome (Figure S1).

### Substrate Scope

To show the usefulness of the enantioconvergent hydrogenation, a wide scope of *E*/*Z* mixtures of enamides belonging to each class was evaluated (Table 2). Class 1 substrates were hydrogenated as 2:1 *E*/*Z* mixtures using the standard conditions (50 bar, room temperature) (Table 2). Different substituents on the α-phenyl ring were well-tolerated. Products **5b** and **5c** bearing a *p*-chloro and *p*-methyl group, respectively, were obtained with excellent enantioselectivity and isolated yield. Interestingly, enamides **1d** with a *p*-methoxy group and **1e** with a *p*-trifluoromethyl group showed lower reactivity. However, it was possible to attain full conversion in excellent yield and enantioselectivity using the modified reaction conditions suitable for class 2, favoring the isomerization of the extremely unreactive *Z* isomer of these two compounds. Also, products bearing heteroaromatic groups were tested (Table 2, **5f** and **5g**) and showed behavior similar to those with aromatic rings. Gratifyingly, the 2-furyl group showed very high selectivity. Next, we evaluated different substituents on the β-phenyl ring, which gave products in high yields with high selectivity (over 92% *ee*) in all cases (Table 2, **5h**–**k**). We continued with class 2 using the optimized conditions for isomeric mixtures (Table 1, entries 3 and 4) and a 1:4 *Z*/*E* isomer ratio, which can be easily obtained from a recent protocol developed in our laboratory.^17^ Different aliphatic linear chains (butyl, propyl, ethyl, and pentyl) were tested, and all gave good selectivity and excellent yields (Table 2, **6a**–**d**).

**Table 2:**
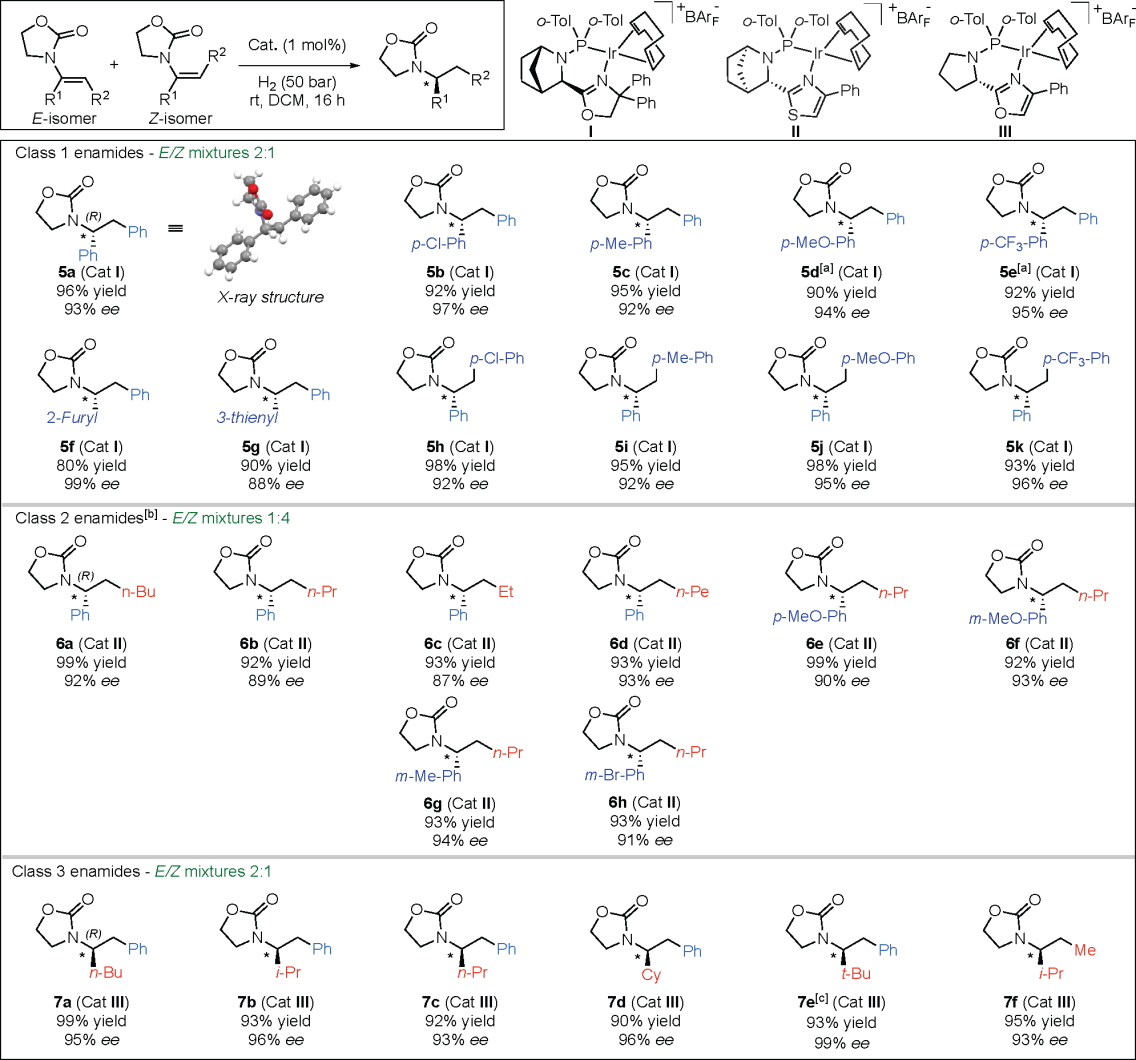
Substrate Scope

a Reaction conditions: 0.15 mmol of substrate, 1 mol % catalyst, and 1.5 mL of DCM. Conversion was determined by ^1^H NMR spectroscopy. Enantiomeric excess was determined by SFC or GC analysis using chiral stationary phases.

b Class 2 optimized conditions: H_2_ (1 bar) at 60 °C in 1 mL of DCE.

c A 9:1 *E*/*Z* mixture was employed.

We then tested different substituents on the aromatic ring and obtained the best results for the methoxy and methyl electron-donating groups. The enamide having bromine at the meta position also gave **6h** with an *ee* and yield higher than 90%. The enamides of class 3 were evaluated using the novel optimized catalyst **III** (Table 2). No isomerization was observed for these substrates, but stereochemical convergence was still achieved, probably as a result of chelation-controlled hydrogenation. The hydrogenation of a 2:1 *E*/*Z* mixture of enamide **3a** bearing the *n*-butyl chain resulted in an excellent 95% *ee* of product **7a**, and similar results were obtained for isopropyl, *n*-propyl, and cyclohexyl (**7b**, **7c**, and **7d**, respectively). When the aliphatic moiety was changed to the more hindered *tert*-butyl group, the conversion was lower, but the thermodynamic 9:1 *E*/*Z* mixture gave an excellent 99% *ee* and 93% yield (Table 2, **7e**)

To further improve the usefulness in synthesis, different amides that are easier to deprotect were also evaluated (Table 3).^18^ Products with the acetamide group (**12a** and **12b**) were obtained with an almost perfect selectivity of 98% *ee.* The benzamide group and the methyl carbamate (**12c** and **12d**) were also tolerated, giving 90% and 92% *ee*, respectively. Finally, when the hydrogenation of the *E*/*Z* mixture of enamide **11a** was scaled up, the high selectivity of 98% *ee* was retained, and the subsequent deprotection of the acetyl group furnished chiral amine **13a** in good yield.

**Table 3:**
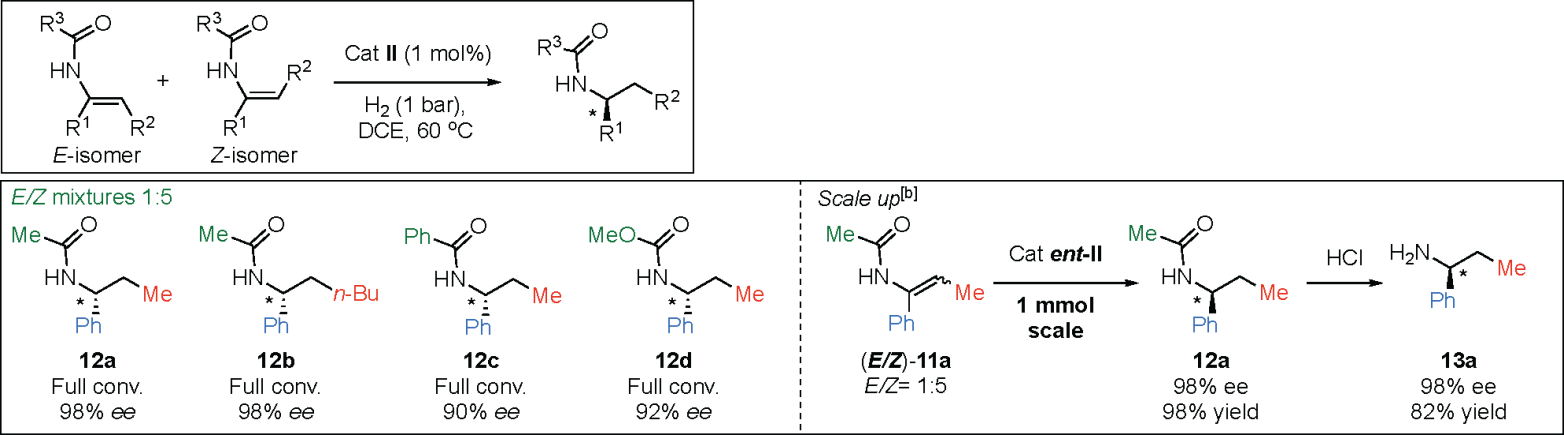
Scope of Amide Groups^a^

a Reaction conditions: 0.05 mmol of substrate, 1 mol % catalyst, 0.5 mL of DCE.

b 1 mmol of substrate, 1 mol % catalyst, 2 mL of DCE.

## Conclusions

We have developed a new and efficient asymmetric hydrogenation of trisubstituted linear enamides using N,P–iridium catalysts. These catalytic systems successfully hydrogenated a wide range of differently substituted mixtures of *E* and *Z* isomers with excellent enantioselectivities. This is an uncommon feature for N,P–iridium catalysts, since the majority of reported proposed mechanisms involve a stereoselectivity-determining step based on steric discrimination of the non-prochiral carbon of the double bond. Furthermore, we have revealed the presence of at least two different mechanistic pathways for the enantioconvergent hydrogenation of enamides: via isomerization and via chelation control. These mechanisms are strongly influenced by the stereoelectronic properties of the substrates, and division of the enamides into classes helped to rationalize the different results.

Finally, DFT studies were carried out to understand the enantioconvergent routes for the hydrogenation of chelating olefins, and they predicted the correct absolute configuration and the fast isomerization of the *E* and *Z* isomers.

## Supporting Information Available

Experimental procedures, additional experimental details, full spectroscopic data for all new compounds, and computational details with Cartesian coordinates of optimized structures (PDF)

## Accession Codes

CCDC 1955080 contains the supplementary crystallographic data for this paper. These data can be obtained free of charge via www.ccdc.cam.ac.uk/data_request/cif, or by emailing data_request@ccdc.cam.ac.uk, or by contacting The Cambridge Crystallographic Data Centre, 12 Union Road, Cambridge CB2 1EZ, U.K.; fax: +44 1223 336033.
