# Supplementary material for: Combined Theoretical and Experimental Studies Unravel Multiple Pathways to Convergent Asymmetric Hydrogenation of Enamides
Source: J Am Chem Soc. 2021 Dec 14;143(51):21594–603. doi: 10.1021/jacs.1c09573 (PMC8719336; doi:10.1021/jacs.1c09573)
Supplement: Supplementary file 1 — ja1c09573_si_001.pdf [file ja1c09573_si_001.pdf]

Combined Theoretical and Experimental Studies Unravel Multiple Pathways to Convergent Asymmetric Hydrogenation of Enamides.

Jianping Yang ‡ [a], Luca Massaro ‡ [a], Suppachai Krajangsri [a], Thishana Singh [b], Hao Su [c], Emanuele Silvi [a], Sudipta Ponra [a], Lars Eriksson [d], Mårten S. G. Ahlquist [c] and Pher G. Andersson* [a],[b].

[a] Department of Organic Chemistry, Stockholm University, Arrhenius Laboratory, 106 91, Stockholm, Sweden. Corresponding author: pher.andersson@su.se
[b] School of Chemistry and Physics, University of Kwazulu-Natal, Private Bag X54001, Durban, 4000, South Africa.
[c] School of Biotechnology, KTH Royal Institute of Technology, 106 91, Stockholm, Sweden.
[d] Department of Materials and Environmental Chemistry, Stockholm University, Svante Arrhenius väg 16C, 106 91, Stockholm, Sweden.
‡ Authors contributed equally to this work

Table of contents

Table of contents S1
1. General methods S2
2. General procedure for substrates synthesis S2
   2.1. General procedure for preparation of 1,2-diarylethan-1-one S2
   2.2 General procedure for preparation α-Aryl-Alkyl ketones S2
   2.3 General procedure for preparation of aldehydes S2
   2.4. General procedure for the preparation of the α,β-enamides (*Z*-isomer) S3
   2.5. General procedure for the preparation of the α,β- enamides (*E*-isomer) S4
   2.6. General procedure for the preparation of the (*Z*)-a,β-enamides from *E* isomer S6
3. General procedure for asymmetric hydrogenation S8
4. General procedure for the preparation of iridium complexes S13
5. Separation method data and specific rotation of chiral compounds S15
6. Absolute configuration determination S19
   6.1 The absolute configuration of the different classes of hydrogenated products S19
   6.2 Di-aryl enamides by single crystal X-ray diffraction S19
7. Conditions screening S20
8. Mechanistic study S23
9. DTF computational details S26
10. NMR spectra of new compounds S30
11. Chromatogram of chiral compounds S81
12. XYZ Coordinates S97
13. References S247

1. General methods

All reactions were conducted under dry and inert atmosphere using magnetic stirring. CH_2Cl_2, used in the hydrogenation, was freshly distilled from CaH_2 under nitrogen. THF was freshly distilled from sodium-benzophenone under nitrogen. All reagents were used as supplied commercially without further purification. Chromatographic separations were performed on Kiesel gel 60 H silica gel (particle size: 0.063-0.100 mm) or Brockmann I, activated. Thin layer chromatography (TLC) was performed on aluminum plates coated with Kieselgel 60 (0.20 mm, UV254) and visualized under ultraviolet light (ν = 254 nm). ^{1}H NMR spectra were recorded on a Bruker 400 or 500 at 400/500 MHz in $CDCl_3$ and referenced internally to the residual $CHCl_3$ peak (7.26 ppm). ^{13}C NMR spectra were recorded at 100 MHz in $CDCl_3$ and referenced to the central peak of $CDCl_3$ (77.16 ppm). Chemical shifts are reported in ppm (δ scale). Enantiomeric excesses were determined either using chiral HPLC, SFC or GC with a diode array detector at 220 and 254 nm. Racemic compounds were used for comparison. HRMS data were obtained using a Bruker MicroTOF-Q II instrument operation at ambient temperature. Optical rotations were recorded on an Autopol IV polarimeter from Rudolp Research Analytical, equipped with a sodium lamp (589 nm) and a 10 cm cell.

2. General procedure for substrates synthesis

2.1. General procedure for preparation of 1,2-diarylethan-1-one

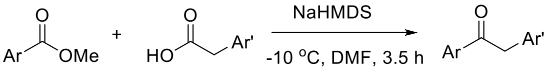

To a solution of aromatic acetic acid (10 mmol, 1.0 equiv.) and aromatic methyl ester (10 mmol, 1.0 equiv.) in DMF (30 mL) was added NaHMDS (2.0 M in THF) (20 mL, 40 mmol, 4.0 equiv.) at -10°C over 1 min. The resulting mixture was stirred at -10°C for 3.5 hours. To the resulting solution was then added saturated aqueous NH_4Cl solution. The resulting mixture was extracted with EtOAc (2x100 ml). The combined organic phase was washed with brine, dried over Na_2SO_4 and concentrated in vacuo. The residue was purified by silica gel column chromatography.

2.2 General procedure for preparation α-Aryl-Alkyl ketones

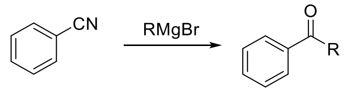

Grignard reagent (15 mmol, 1.5 equiv.) was slowly added to a solution of cyano (-CN) (10 mmol, 1.0 equiv.) aromatic compounds in dry THF (30 mL) at 0°C. The reaction was allowed to stir at room temperature for 16 h. The reaction was quenched by addition of a saturated aqueous NH_4Cl solution, and 1M HCl was added and the mixture was stirred for 1 h. The mixture was extracted with Et_2O (3x100 ml). The combined organic phases were combined and washed brine and dried over Na_2SO_4. The solvent was evaporated under reduced pressure to afford the crude products which then were purified by column chromatography on silica gel.

2.3 General procedure for preparation of aldehydes

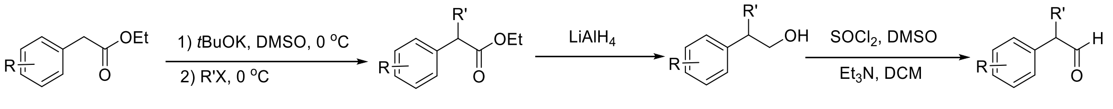

Potassium tert-butoxide (1 equiv.) was suspended in dry DMF (7 mL/10 mmol) at 0°C under nitrogen atmosphere and methyl phenylacetate (1 equiv.) was added in one portion, followed

by alkyl halide (1 equiv.) after 2 min. The reaction was allowed to warm up to room temperature and magnetic stirring was continued for 1 h. Water (10 mL) was added and the solution was extracted with CH_2Cl_2 (2 times). The organic layers were washed with 10 mL of a saturated, aqueous solution of NH_4Cl and 10 mL of water, and dried over Na_2SO_4. The solvent was evaporated and the crude product was purified by column chromatography.
$LiAlH_4$ (1.2 equiv.) was suspended in dry Et_2O (3 mL/10 mmol). Then, a solution of alkylated ester (1 equiv.) in 20 mL of Et_2O was added slowly while stirring. The reaction was monitored by TLC and after complete consumption of the ester the reaction was quenched by adding water. The layers were separated and the aqueous layer was extracted with CH_2Cl_2 (2 times). The combined organic layers were dried over Na_2SO_4. The solvent was evaporated and the product was obtained as colorless oil after purification by column chromatography.
To a solution of oxalyl chloride (1.1 equiv.) in CH_2Cl_2 (13 mL/10 mmol) was added a solution of DMSO (2.4 equiv.) in CH_2Cl_2 (3 mL/10 mmol) at -78 °C. The solution was stirred for 10 min and alcohol (1 equiv.) in CH_2Cl_2 (20 mL/10 mmol) was added. After 15 min, Et_3N (7.5 mL/ 10 mmol of alcohol) was added, and the solution was allowed to warm up to room temperature and stirred for 1 h. Water was added to quench the reaction, the phases were separated, and the aqueous layer was extracted with CH_2Cl_2 (2 times). The organic phase was washed with water, dried over Na_2SO_4, and the solvent was evaporated. The corresponding aldehyde was obtained as colorless solid after silica-gel flash chromatography.

2.4. General procedure for the preparation of the α,β-enamides (*Z*-isomer)

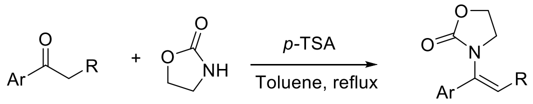

A solution of the ketone (10 mmol, 1.0 equiv.), 2-oxazolidinone (30 mmol, 2.610g, 3 equiv.), and 10 mol% of *p*-toluenesulfonic acid (1 mmol, 190 mg) in toluene (30 mL: 10 mmol ketone) was heated to reflux and the formed water was removed by azeotropic distillation over 36 h. After cooling the solution to room temperature, it was washed with a saturated, aqueous solution of NH_4Cl (10 mL) and water (10 mL), and the combined organic layers were dried over Na_2SO_4. After evaporation of the organic solvent and purification by silica-gel flash chromatography (pentane/ethyl acetate 80:20 to 70:30), the pure Z-isomers of the α,β-enamides were obtained.
Characterization for the following compounds have been previously reported: **1a-1k**,[1] **2a-2c**,[1] **2f-2h**,[1] **4a**[2], **4b**[3], **4c**[1], **11c**[4,5], **11d**[6].

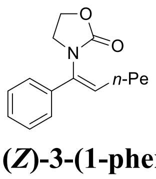

(*Z*)-3-(1-phenylhept-1-en-1-yl)oxazolidin-2-one(*Z*-2d) White solid. ^{1}H NMR (400 MHz, Chloroform-*d*) δ 7.39 – 7.27 (m, 5H), 6.01 (t, *J* = 7.2 Hz, 1H), 4.54 – 4.41 (m, 2H), 3.70 – 3.55 (m, 2H), 2.22 (q, *J* = 7.3 Hz, 2H), 1.58 – 1.44 (m, 2H), 1.44 – 1.31 (m, 4H), 0.91 (t, *J* = 7.4 Hz, 3H). ^{13}C NMR (101 MHz, Chloroform-*d*) δ 156.6, 136.0, 133.8, 131.0, 128.8, 128.3, 126.0, 62.4, 45.8, 31.8, 28.7, 28.4, 22.6, 14.1. HRMS-ESI calcd for $C_{16}H_{21}NO_2$ $[M+Na]^+$: 282.1465, found: 282.1463.

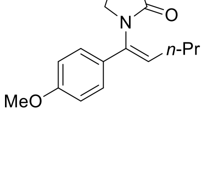

(*Z*)-3-(1-(4-methoxyphenyl)pent-1-en-1-yl)oxazolidin-2-one(*Z*-2e) Colorless oil**.** ^{1}H NMR (400 MHz, Chloroform-*d*) δ 7.30 – 7.23 (m, 2H), 6.92 – 6.83 (m, 2H), 5.89 (t, *J* = 7.2 Hz, 1H), 4.53 – 4.41 (m, 2H), 3.81 (s, 3H), 3.68 – 3.60 (m, 2H), 2.18 (q, *J* = 7.3 Hz, 2H), 1.62 – 1.48 (m, 2H), 0.98 (t, *J* = 7.4 Hz, 3H). ^{13}C NMR (101 MHz, Chloroform-d) δ 159.8, 156.6, 133.6, 128.9, 128.6, 127.3, 114.2, 62.4, 55.5, 45.9, 30.4, 22.4, 14.2. HRMS-ESI calcd for $C_{15}H_{19}NO_3$ $[M+Na]^+$: 284.1257, found: 284.1244.

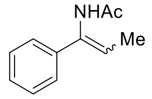

(*Z/E*)-*N*-(1-phenylprop-1-en-1-yl)acetamide(11a)[7] White solid. (*Z*)-*N*-Acetyl-1-phenylpropenamine (rotamers ratio 1.9:1): Major rotamer: ^{1}H NMR (400 MHz, $CDCl_3$): δ 7.23-7.45 (m, 5H), 6.57 (br, s, 1H), 5.97 (q, *J* = 6.9 Hz, 1H,), 2.19 (s, 3H) 1.77 (d, *J* = 7.0 Hz, 3H,). Minor rotamer: δ 7.23-7.45 (m, 5H), 6.58 (br, s, 1H), 6.05 (q, *J* = 7.0 Hz, 1H,), 1.86 (d, *J* = 7.0 Hz, 3H), 1.82 (s, 3H).

(*Z*)-*N*-Acetyl-1-phenylpropenamine (rotamers are not splitted in DMSO): ^{1}H NMR (400 MHz, DMSO-d_6) δ 9.10 (s, 1H), 7.21 - 7.40 (m, 5H), 5.90 (q, *J* = 6.8 Hz, 1H), 2.01 (s, 3H), 1.63 - 1.70(m, 3H).

Isolated (*E*)- *N*-Acetyl-1-phenylpropenamine: 1H NMR (400 MHz, $CDCl_3$): δ 7.30-7.40 (m, 5H), 6.62 (br, s, 1H), 6.33 (q, *J* = 6.42, 1H), 2.03-2.04 (m, 3H), 1.83-1.86 (m, 1H), 1.70 (d, *J* = 7.2 Hz, 3H). HRMS-ESI calcd for $C_{11}H_{13}NO$ $[M+Na]^+$: 198.0889, found: 198.0895.

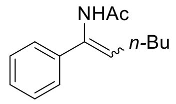

(*Z/E*)-*N*-(1-phenylhex-1-en-1-yl)acetamide(11b) White solid. (*Z*)-*N*-(1-phenylhex-1-en-1-yl)acetamide (rotamers ratio 1.6:1) Major rotamer: ^{1}H NMR (400 MHz, Chloroform-*d*) δ 7.47 – 7.12 (m, 5H), 6.61 (br, s, 1H), 5.86 (t, *J* = 7.11 Hz, 1H), 2.21 – 2.11 (m, 5H), 1.52 – 1.33 (m, 4H), 0.93 (t, *J* = 7.20 Hz, 3H). Minor rotamer: ^{1}H NMR (400 MHz, Chloroform-*d*) δ 7.46 – 7.20 (m, 5H), 6.61 (br, s, 1H), 5.94 (t, *J* = 7.42 Hz, 1H), 2.28 – 2.21 (m, 2H), 2.17 (s, 3H), 1.52 – 1.33 (m, 4H), 0.93 (t, *J* = 7.30 Hz, 3H).

(*E*)-*N*-(1-phenylhex-1-en-1-yl)acetamide. ^{1}H NMR (400 MHz, Chloroform-*d*) δ 7.46 – 7.22 (m, 5H), 6.52 (br, s, 1H), 6.30 (t, *J* = 7.79 Hz, 1H), 2.07 – 2.00 (m, 5H), 1.35 – 1.17 (m, 4H), 0.83 (t, *J* = 7.19 Hz, 3H). HRMS-ESI calcd for $C_{14}H_{19}NO$ $[M+Na]^+$: 240.1359, found: 240.1346.

2.5. General procedure for the preparation of the α,β- enamides (*E*-isomer)

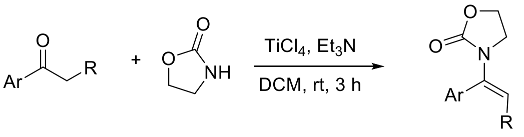

A nitrogen-flooded sealed vial containing corresponding ketones (1.1 equiv.) in dry CH_2Cl_2 was cooled down to 0°C before $TiCl_4$ (1M solution in CH_2Cl_2 or neat, 1.2 equiv.) was added dropwise. The resulting light yellow/orange solution was stirred at this temperature for 5 min and 0°C 2-oxazolidone (1 equiv.) pre-dissolved in CH_2Cl_2 (5 mL) was added slowly followed by dropwise addition of NEt_3 (5 equiv.). The color of the reaction mixture turned black upon addition of the base, a color change that did not occur when using most other bases. The reaction mixture was allowed to warm up to r.t. and was stirred over 3 h with regular monitoring using thin layer chromatography plates (eluent: ethyl acetate 1:2 pentane). The crude mixture was cooled to 0°C, quenched by water (4 equiv.) and diluted with CH_2Cl_2

followed by portwise addition of slica. The mixture was filtered by celite. The filtrate was washed with water. The layers were separated and the organic phase was extracted with CH_2Cl_2 (3 × 50 mL). The combined organic layers were dried over Na_2SO_4 and concentrated under reduced pressure. The residue was then purified by flash chromatography on silica gel (ethyl acetate/pentane, gradient from 1:4 to 1:2) to provide clean *E*-enamides.

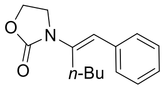

(*E*)-3-(1-phenylhex-1-en-2-yl)oxazolidin-2-one(*E*-3a) Colorless oil. ^{1}H NMR (400 MHz, Chloroform-*d*) δ 7.36 – 7.30 (m, 2H), 7.25 – 7.19 (m, 3H), 6.18 (s, 1H), 4.44 – 4.35 (m, 2H), 3.94 – 3.83 (m, 2H), 2.77 – 2.66 (m, 2H), 1.56 – 1.47 (m, 2H), 1.33 (h, *J* = 7.3 Hz, 2H), 0.87 (t, *J* = 7.3 Hz, 3H). ^{13}C NMR (101 MHz, Chloroform-*d*) 155.2, 140.1, 136.4, 129.0, 128.4, 126.7, 117.3, 61.5, 46.5, 30.8, 27.4, 22.6, 14.0, HRMS-ESI calcd for $C_{15}H_{19}NO_2$ $[M+Na]^+$: 268.1313, found: 268.1308.

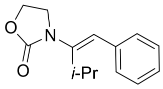

(*E*)-3-(3-methyl-1-phenylbut-1-en-2-yl)oxazolidin-2-one(*E*-3b) White solid. ^{1}H NMR (400 MHz, Chloroform-*d*) δ 7.35 – 7.27 (m, 4H), 7.26 – 7.21 (m, 1H), 6.34 (s, 1H), 4.31 – 4.21 (m, 2H), 3.49 – 3.40 (m, 2H), 3.00 (heptd, *J* = 6.8, 1.4 Hz, 1H), 1.19 (d, *J* = 6.9 Hz, 6H). ^{13}C NMR (101 MHz, Chloroform-*d*) δ 156.8, 142.5, 135.6, 128.7, 128.0, 127.6, 123.0, 62.6, 46.0, 31.8, 20.8. HRMS-ESI calcd for $C_{14}H_{17}NO_2$ $[M+Na]^+$: 254.1157, found: 254.1169.

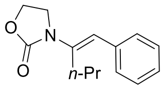

(*E*)-3-(1-phenylpent-1-en-2-yl)oxazolidin-2-one(*E*-3c) Colorless oil. ^{1}H NMR (400 MHz, Chloroform-*d*) δ 7.36 – 7.30 (m, 2H), 7.25 – 7.18 (m, 3H), 6.19 (s, 1H), 4.44 – 4.36 (m, 2H), 3.94 – 3.87 (m, 2H), 2.74 – 2.62 (m, 2H), 1.62 – 1.48 (m, 2H), 0.92 (t, *J* = 7.4 Hz, 3H). ^{13}C NMR (101 MHz, Chloroform-*d*) δ 155.2, 139.9, 136.4, 129.0, 128.4, 126.7, 61.5, 46.5, 29.5, 21.9, 13.9, HRMS-ESI calcd for $C_{14}H_{17}NO_2$ $[M+Na]^+$: 254.1157, found: 254.11538.

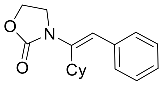

(*E*)-3-(1-cyclohexyl-2-phenylvinyl)oxazolidin-2-one(*E*-3d) Colorless oil. ^{1}H NMR (400 MHz, Chloroform-*d*) δ 7.35 – 7.26 (m, 4H), 7.25 – 7.20 (m, 1H), 6.30 (s, 1H), 4.31 – 4.20 (m, 2H), 3.49 – 3.34 (m, 2H), 2.61 (tdt, *J* = 10.1, 3.0, 1.4 Hz, 1H), 2.06 – 1.94 (m, 2H), 1.82 (dt, *J* = 12.4, 3.0 Hz, 2H), 1.77 – 1.69 (m, 1H), 1.45 – 1.09 (m, 5H). ^{13}C NMR (101 MHz, Chloroform-*d*) δ 156.9, 141.9, 135.7, 128.7, 128.1, 127.5, 123.5, 62.5, 46.2, 41.7, 31.5, 26.4, 26.4. HRMS-ESI calcd for $C_{17}H_{21}NO_2$ $[M+Na]^+$: 294.1470, found: 294.1479.

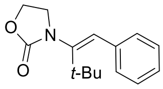

(*E*)-3-(3,3-dimethyl-1-phenylbut-1-en-2-yl)oxazolidin-2-one(*E*-3e) White solid. ^{1}H NMR (400 MHz, Chloroform-*d*) δ 7.38 – 7.27 (m, 2H), 7.30 – 7.20 (m, 3H), 6.64 (s, 1H), 4.43 – 3.88 (m, 2H), 3.71 – 3.17 (m, 2H), 1.27 (d, *J* = 1.0 Hz, 9H). ^{13}C NMR (101 MHz, Chloroform-*d*) δ 158.1, 145.4, 135.5, 128.8, 128.0, 127.7, 126.6, 62.3, 47.3, 38.8, 29.7, HRMS-ESI calcd for $C_{15}H_{19}NO_2$ $[M+Na]^+$: 268.1313, found: 268.1302.

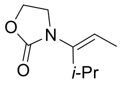

(*E*)-3-(4-methylpent-2-en-3-yl)oxazolidin-2-one(*E*-3f) Colorless oil. ^{1}H NMR (400 MHz, Chloroform-*d*) δ 5.50 (q, *J* = 7.1 Hz, 1H), 4.38 – 4.29 (m, 2H), 3.77 – 3.67 (m, 2H), 2.96 (p, *J* = 7.0 Hz, 1H), 1.72 (d, *J* = 7.1 Hz, 3H), 1.10 (d, *J* = 7.0 Hz, 6H). ^{13}C NMR (101 MHz, Chloroform-*d*) δ 158.1, 141.7, 123.6, 61.8, 49.7, 29.2, 20.8, 12.9, HRMS-ESI calcd for $C_9H_{15}NO_2$ $[M+Na]^+$: 192.1000, found: 192.0993.

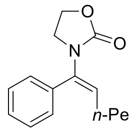

(*E*)-3-(1-phenylhept-1-en-1-yl)oxazolidin-2-one(*E*-2d) White solid**.** ^{1}H NMR (500 MHz, Chloroform-*d*) δ 7.40 – 7.35 (m, 2H), 7.35 – 7.31 (m, 1H), 7.28 – 7.25 (m, 2H), 5.86 (t, *J* = 7.8 Hz, 1H), 4.36 – 4.23 (m, 2H), 3.59 – 3.44 (m, 2H), 2.10 (q, *J* = 7.7 Hz, 2H), 1.42 (p, *J* = 7.3 Hz, 2H), 1.30 – 1.20 (m, 4H), 0.86 (t, *J* = 7.0 Hz, 3H). ^{13}C NMR (126 MHz, Chloroform-*d*) δ 156.6, 134.8, 134.4, 129.0, 128.5, 128.3, 126.0, 61.6, 46.2, 31.5, 29.7, 28.1, 22.6, 14.1. HRMS-ESI calcd for $C_{16}H_{21}NO_2$ $[M+Na]^+$: 282.1465, found: 282.1463.

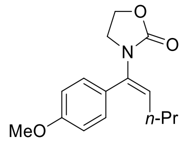

(*E*)-3-(1-(4-methoxyphenyl)pent-1-en-1-yl)oxazolidin-2-one(*E*-2e) White solid. ^{1}H NMR (400 MHz, Chloroform-*d*) δ 7.30 – 7.21 (m, 2H), 6.90 – 6.82 (m, 2H), 5.88 (t, *J* = 7.2 Hz, 1H), 4.52 – 4.40 (m, 2H), 3.81 (s, 3H), 3.68 – 3.58 (m, 2H), 2.18 (q, *J* = 7.3 Hz, 2H), 1.60 – 1.46 (m, 2H), 0.97 (t, *J* = 7.4 Hz, 3H). ^{13}C NMR (101 MHz, Chloroform-*d*) δ 159.8, 156.6, 133.6, 128.9, 128.6, 127.3, 114.2, 62.4, 55.5, 45.9, 30.4, 22.3, 14.2. HRMS-ESI calcd for $C_{15}H_{19}NO_3$ $[M+Na]^+$: 284.1257, found: 284.1232.

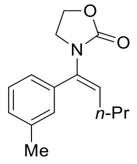

(*E*)-3-(1-(m-tolyl)pent-1-en-1-yl)oxazolidin-2-one(*E*-2g) Colorless oil. ^{1}H NMR (400 MHz, Chloroform-*d*) δ 7.30 – 7.23 (m, 1H), 7.14 (d, *J* = 7.6 Hz, 1H), 7.06 (d, *J* = 7.4 Hz, 2H), 5.84 (t, *J* = 7.7 Hz, 1H), 4.34 – 4.25 (m, 2H), 3.59 – 3.47 (m, 2H), 2.36 (s, 3H), 2.08 (q, *J* = 7.6 Hz, 2H), 1.44 (h, *J* = 7.3 Hz, 2H), 0.90 (t, *J* = 7.4 Hz, 3H). ^{13}C NMR (101 MHz, Chloroform-*d*) δ 156.7, 138.2, 134.8, 134.7, 129.5, 129.1, 128.4, 126.2, 125.8, 61.6, 46.2, 30.2, 23.2, 21.6, 13.9. HRMS-ESI calcd for $C_{15}H_{19}NO_2$ $[M+Na]^+$: 268.1308, found: 268.1314.

2.6. General procedure for the preparation of the (*Z*)-a,β-enamides from *E* isomer

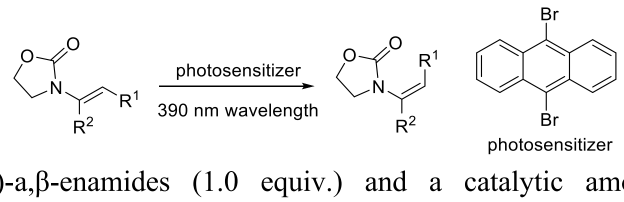

A solution of (*E*)-a,β-enamides (1.0 equiv.) and a catalytic amount (5 mol%) of photosensitizer in CH_2Cl_2 (2 mL/2 mmol enamides) was irradiated using 390 nm sodium lamp overnight. The solvent was removed under vacuum. After purification by silica-gel flash chromatography, the pure *Z*-enamides were obtained.

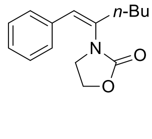

(*Z*)-3-(1-phenylhex-1-en-2-yl)oxazolidin-2-one(*Z*-3a) Colorless oil. ^{1}H NMR (400 MHz, Chloroform-*d*) δ 7.36 – 7.27 (m, 4H), 7.26 – 7.21 (m, 1H), 6.28 (s, 1H), 4.34 – 4.24 (m, 2H), 3.52 – 3.44 (m, 2H), 2.59 – 2.47 (m, 2H), 1.55 – 1.32 (m, 4H), 0.95 (t, *J* = 7.2 Hz, 3H). ^{13}C NMR (101 MHz, Chloroform-*d*) δ 156.6, 137.1, 135.6, 128.7, 128.2, 127.6, 124.5, 62.7, 45.6, 33.9, 29.5, 22.5, 14.1, HRMS-ESI calcd for $C_{15}H_{19}NO_2$ [M+Na]$^{+}$: 268.1313, found: 268.1309.

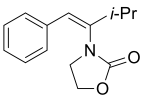

(*Z*)-3-(3-methyl-1-phenylbut-1-en-2-yl)oxazolidin-2-one(*Z*-3b) White solid. ^{1}H NMR (400 MHz, Chloroform-*d*) δ 7.35 – 7.28 (m, 2H), 7.27 – 7.20 (m, 3H), 6.47 (s, 1H), 4.41 – 4.31 (m, 2H), 3.85 (ddd, *J* = 9.2, 6.7, 1.6 Hz, 2H), 3.15 (pd, *J* = 7.0, 1.5 Hz, 1H), 1.13 (dt, *J* = 7.0, 1.3 Hz, 6H). ^{13}C NMR (101 MHz, Chloroform-*d*) δ 157.8, 143.7, 135.7, 128.9, 128.4, 128.4, 127.5, 61.9, 49.9, 30.2, 21.3. HRMS-ESI calcd for $C_{14}H_{17}NO_2$ [M+Na]$^{+}$: 254.1157, found: 254.1154.

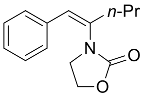

(*Z*)-3-(1-phenylpent-1-en-2-yl)oxazolidin-2-one(*Z*-3c) Colorless oil. ^{1}H NMR (400 MHz, Chloroform-*d*) δ 7.35 – 7.28 (m, 4H), 7.25 – 7.20 (m, 1H), 6.28 (s, 1H), 4.33 – 4.22 (m, 2H), 3.51 – 3.42 (m, 2H), 2.57 – 2.47 (m, 2H), 1.63 – 1.49 (m, 2H), 0.99 (t, *J* = 7.3 Hz, 3H). ^{13}C NMR (101 MHz, Chloroform-*d*) δ 156.6, 136.9, 135.6, 128.7, 128.2, 127.6, 124.7, 62.7, 45.6, 36.3, 20.5, 13.8, HRMS-ESI calcd for $C_{14}H_{17}NO_2$ [M+Na]$^{+}$: 254.1157, found: 254.1157.

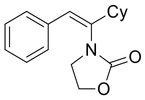

(*Z*)-3-(1-cyclohexyl-2-phenylvinyl)oxazolidin-2-one(*Z*-3d)

^{1}H NMR (400 MHz, Chloroform-*d*) δ 7.40 – 7.32 (m, 2H), 7.31 – 7.21 (m, 3H), 6.49 (s, 1H), 4.44 – 4.35 (m, 2H), 3.92 – 3.83 (m, 2H), 2.81 (tt, *J* = 12.1, 3.1 Hz, 1H), 1.90 – 1.70 (m, 4H), 1.70 – 1.60 (m, 1H), 1.53 – 1.37 (m, 2H), 1.31 – 1.16 (m, 3H). ^{13}C NMR (101 MHz, Chloroform-*d*) δ 157.9, 143.2, 135.6, 129.2, 128.9, 128.5, 127.5, 61.9, 50.1, 41.1, 31.4, 26.2, 26.0. HRMS-ESI calcd for $C_{17}H_{21}NO_2$ [M+Na]$^{+}$: 294.1465, found: 294.1453.

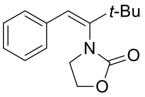

(*Z*)-3-(3,3-dimethyl-1-phenylbut-1-en-2-yl)oxazolidin-2-one(*Z*-3e) White solid. ^{1}H NMR (400 MHz, Chloroform-*d*) δ 7.34 – 7.28 (m, 2H), 7.28 – 7.21 (m, 3H), 6.68 (s, 1H), 4.44 – 4.36 (m, 2H), 3.93 – 3.85 (m, 2H), 1.08 (s, 9H). ^{13}C NMR (101 MHz, Chloroform-*d*) δ 158.1, 146.5, 136.9, 131.6, 128.7, 128.0, 127.1, 61.9, 50.8, 38.6, 30.7. HRMS-ESI calcd for $C_{15}H_{19}NO_2$ [M+Na]$^{+}$: 268.1313, found: 268.1324.

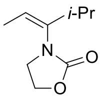

(*Z*)-3-(4-methylpent-2-en-3-yl)oxazolidin-2-one(*Z*-3f) Colorless oil. ^{1}H NMR (400 MHz, Chloroform-*d*) δ 5.48 (qd, *J* = 6.8, 1.3 Hz, 1H), 4.46 – 4.36 (m, 2H), 3.75 – 3.66 (m, 2H), 2.49 (ttt, *J* = 6.8, 5.4, 1.5 Hz, 1H), 1.61 (dd, *J* = 6.9, 1.4 Hz, 3H), 1.08 (s, 3H), 1.07 (s, 3H). ^{13}C NMR (101 MHz, Chloroform-*d*) δ 156.5, 140.8, 120.0, 62.3, 46.2, 31.6, 20.9, 13.0. HRMS-ESI calcd for $C_9H_{15}NO_2$ [M+Na]$^{+}$: 192.1000, found: 192.0992.

3. General procedure for asymmetric hydrogenation

A vial was charged with substrate (0.015 mmol) and Ir-complex (1 mol%). Dry CH_2Cl_2 (1.5 mL) was added (so that the concentration of the substrate was 0.1 M) and the vial was placed in a high-pressure hydrogenation apparatus. The reactor was purged three times with Ar gas, then filled with H_2. The reaction was stirred at room temperature for 16 hours before the H_2 pressure was released and the solvent was removed *in vacuo*. The crude product was filtered through on a short plug of silica. Conversions were determined by ^{1}H NMR spectroscopy and *ee* values were determined by HPLC , SFC or GCMS using a chiral stationary phase.

Characterization for the following compounds have been previously reported: **12a**[8], **12b**[9], **12c**[10], **12d**[11], **13a**[12].

The characterization of the hydrogenated compounds:

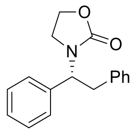

(*R*)-3-(1,2-diphenylethyl)oxazolidin-2-one(5a) White solid, 98% yield. ^{1}H NMR (400 MHz, Chloroform-*d*) δ 7.44 – 7.34 (m, 4H), 7.34 – 7.29 (m, 2H), 7.27 (tt, *J* = 3.5, 2.0 Hz, 3H), 7.24 – 7.18 (m, 1H), 5.35 (dd, *J* = 9.7, 6.7 Hz, 1H), 4.29 – 3.87 (m, 2H), 3.53 (td, *J* = 8.2, 6.9 Hz, 1H), 3.45 – 3.03 (m, 3H). ^{13}C NMR (101 MHz, Chloroform-*d*) δ 158.0, 138.4, 137.4, 128.8, 128.8, 128.7, 128.2, 127.7, 126.8, 61.9, 57.5, 40.9, 36.8, HRMS-ESI calcd for $C_{17}H_{17}NO_2$ $[M+Na]^+$: 290.1151, found: 290.1157.

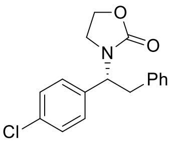

(*R*)-3-(1-(4-chlorophenyl)-2-phenylethyl)oxazolidin-2-one(5b) white solid, 92% yield. ^{1}H NMR (400 MHz, Chloroform-*d*) δ 7.34 (s, 4H), 7.32 – 7.27 (m, 2H), 7.25 – 7.19 (m, 3H), 5.31 (dd, *J* = 9.4, 7.1 Hz, 1H), 4.25 – 4.12 (m, 2H), 3.52 (td, *J* = 8.3, 6.7 Hz, 1H), 3.39 – 3.11 (m, 3H). ^{13}C NMR (101 MHz, Chloroform-*d*) δ 158.0, 137.1, 136.9, 134.1, 129.1, 129.1, 128.8, 128.8, 127.0, 62.0, 57.0, 41.0, 36.9, HRMS-ESI calcd for $C_{17}H_{16}ClNO_2$ $[M+Na]^+$: 324.0762, found: 324.0757.

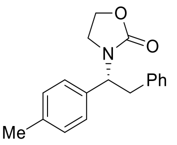

(*R*)-3-(2-phenyl-1-(p-tolyl)ethyl)oxazolidin-2-one(5c) White solid, 95% yield. ^{1}H NMR (400 MHz, Chloroform-*d*) δ 7.32 – 7.24 (m, 6H), 7.23 – 7.15 (m, 3H), 5.32 (dd, *J* = 9.6, 6.7 Hz, 1H), 4.18 – 4.10 (m, 2H), 3.52 (td, *J* = 8.2, 6.9 Hz, 1H), 3.36 – 3.20 (m, 3H), 2.35 (s, 3H). ^{13}C NMR (101 MHz, Chloroform-*d*) δ 158.0, 138.0, 137.6, 135.4, 129.5, 128.9, 128.7, 127.6, 126.8, 61.9, 57.2, 40.8, 36.9, 21.2, HRMS-ESI calcd for $C_{18}H_{19}NO_2$ $[M+Na]^+$: 304.1308, found: 304.1306.

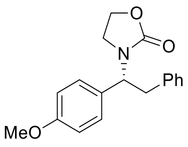

(*R*)-3-(1-(4-methoxyphenyl)-2-phenylethyl)oxazolidin-2-one(5d) white solid, 90% yield. ^{1}H NMR (400 MHz, Chloroform-*d*) δ 7.35 – 7.26 (m, 4H), 7.26 – 7.18 (m, 3H), 6.93 – 6.83 (m, 2H), 5.30 (dd, *J* = 9.5, 6.9 Hz, 1H), 4.18 – 4.12 (m, 2H), 3.80 (s, 3H), 3.51 (td, *J* = 8.2, 6.8 Hz, 1H), 3.35 – 3.18 (m, 3H). ^{13}C NMR (101 MHz, Chloroform-*d*) δ 159.4, 158.0, 137.6, 130.5, 129.0, 128.9, 128.7, 126.8, 114.2, 61.9, 57.0, 55.4, 40.8, 37.1, HRMS-ESI calcd for $C_{18}H_{19}NO_3$ $[M+Na]^+$: 320.1257, found: 320.1259.

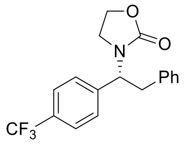

(*R*)-3-(2-phenyl-1-(4-(trifluoromethyl)phenyl)ethyl)oxazolidin-2-one(5e) White solid, 92% yield. ^{1}H NMR (400 MHz, Chloroform-*d*) δ 7.63 (d, *J* = 8.1 Hz, 2H), 7.53 (d, *J* = 8.1 Hz, 2H), 7.38 – 7.18 (m, 5H), 5.38 (dd, *J* = 9.3, 7.1 Hz, 1H), 4.30 – 4.10 (m, 2H), 3.56 (td, *J* = 8.3, 6.7 Hz, 1H), 3.39 – 3.14 (m, 3H). ^{13}C NMR (101 MHz, Chloroform-d) δ 158.0, 142.5, 136.8, 130.5 (q, J = 32.7 Hz), 128.9, 128.8, 128.1, 128.1, 127.1, 125.9 (q, J = 3.8 Hz), 122.7 (q, J = 272.2 Hz), 62.0, 57.2, 41.1, 36.8. ^{19}F NMR (377 MHz, Chloroform-*d*) δ -62.7. HRMS-ESI calcd for $C_{18}H_{16}F_3NO_2$ $[M+Na]^+$: 358.1025, found: 358.1024.

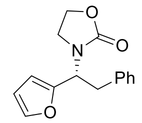

(*R*)-3-(1-(furan-2-yl)-2-phenylethyl)oxazolidin-2-one(5f) Colorless oil, 80% yield. ^{1}H NMR (400 MHz, Chloroform-*d*) δ 7.26 (dd, *J* = 1.8, 0.8 Hz, 1H), 7.17 – 7.12 (m, 2H), 7.10 – 7.03 (m, 4H), 6.20 – 6.04 (m, 2H), 5.23 – 5.13 (m, 1H), 4.15 – 4.00 (m, 2H), 3.46 (td, *J* = 8.7, 6.1 Hz, 1H), 3.28 – 3.16 (m, 2H), 3.01 (dd, *J* = 14.1, 8.8 Hz, 1H). ^{13}C NMR (101 MHz, Chloroform-*d*) δ 158.0, 152.0, 142.5, 136.8, 128.9, 128.7, 127.0, 110.5, 108.5, 62.1, 52.0, 41.4, 36.6. HRMS-ESI calcd for $C_{15}H_{15}NO_3$ $[M+Na]^+$: 280.0950, found: 280.0967.

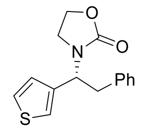

(*R*)-3-(2-phenyl-1-(thiophen-3-yl)ethyl)oxazolidin-2-one(5g) Colorless oil, 90% yield. ^{1}H NMR (400 MHz, Chloroform-*d*) δ 7.32 – 7.28 (m, 1H), 7.28 – 7.25 (m, 2H), 7.25 – 7.17 (m, 4H), 7.09 (dd, *J* = 5.0, 1.4 Hz, 1H), 5.39 (dd, *J* = 9.5, 6.4 Hz, 1H), 4.21 – 4.04 (m, 2H), 3.46 (dd, *J* = 8.7, 5.9 Hz, 1H), 3.34 (dd, *J* = 14.3, 6.3 Hz, 1H), 3.25 – 3.13 (m, 2H). ^{13}C NMR (101 MHz, Chloroform-*d*) δ 158.0, 139.8, 137.2, 128.7, 128.7, 127.1, 126.9, 126.5, 122.6, 61.9, 53.3, 40.7, 37.7, HRMS-ESI calcd for $C_{15}H_{15}NO_2S$ $[M+Na]^+$: 296.0716, found: 296.0721.

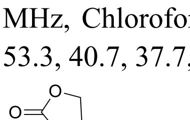

(*R*)-3-(2-(4-chlorophenyl)-1-phenylethyl)oxazolidin-2-one(5h) White solid, 98% yield. ^{1}H NMR (400 MHz, Chloroform-*d*) δ 7.34 (s, 4H), 7.31 – 7.27 (m, 2H), 7.25 – 7.19 (m, 3H), 5.31 (dd, *J* = 9.4, 7.1 Hz, 1H), 4.22 – 4.11 (m, 2H), 3.52 (td, *J* = 8.3, 6.7 Hz, 1H), 3.34 – 3.15 (m, 3H). ^{13}C NMR (101 MHz, Chloroform-*d*) δ 158.0, 137.1, 136.9, 134.1, 129.1, 129.1, 128.8, 128.8, 127.0, 62.0, 57.0, 41.0, 36.9, HRMS-ESI calcd for $C_{17}H_{16}ClNO_2$ $[M+Na]^+$: 324.0762, found: 324.0754.

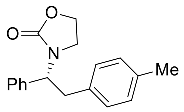

(*R*)-3-(1-phenyl-2-(p-tolyl)ethyl)oxazolidin-2-one(5i) White solid, 95% yield. ^{1}H NMR (400 MHz, Chloroform-*d*) δ 7.43 – 7.34 (m, 4H), 7.34 – 7.28 (m, 1H), 7.14 (d, *J* = 8.1 Hz, 2H), 7.09 (d, *J* = 7.9 Hz, 2H), 5.33 (dd, *J* = 9.8, 6.5 Hz, 1H), 4.20 – 4.09 (m, 2H), 3.53 (td, *J* = 8.3, 6.9 Hz, 1H), 3.37 – 3.18 (m, 3H), 2.30 (s, 3H). ^{13}C NMR (101 MHz, Chloroform-*d*) δ 158.1, 138.6, 136.4, 134.3, 129.4, 128.9, 128.7, 128.2, 127.7, 61.9, 57.5, 40.8, 36.5, 21.2, HRMS-ESI calcd for $C_{18}H_{19}NO_2$ $[M+Na]^+$: 304.1308, found: 304.1304.

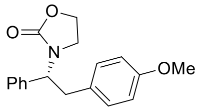

(*R*)-3-(2-(4-methoxyphenyl)-1-phenylethyl)oxazolidin-2-one(5j) Colorless oil, 98% yield. ^{1}H NMR (400 MHz, Chloroform-*d*) δ 7.42 – 7.28 (m, 5H), 7.19 – 7.14 (m, 2H), 6.85 – 6.80 (m, 2H), 5.29 (dd, *J* = 9.7, 6.6 Hz, 1H), 4.19 – 4.13 (m, 2H), 3.77 (s, 3H), 3.52 (td, *J* = 8.2, 7.0 Hz, 1H), 3.32 – 3.18 (m, 3H). ^{13}C NMR (101 MHz, Chloroform-*d*) δ 158.4, 158.1, 138.5, 129.8, 129.4, 128.9, 128.2, 127.7, 114.1, 61.9, 57.6, 55.3, 40.9, 36.0, HRMS-ESI calcd for $C_{18}H_{19}NO_3$ $[M+Na]^+$: 320.1257, found: 320.1242.

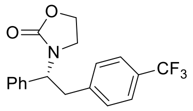

(*R*)-3-(1-phenyl-2-(4-(trifluoromethyl)phenyl)ethyl)oxazolidin-2-one(5k) White solid, 93% yield. ^{1}H NMR (400 MHz, Chloroform-*d*) δ 7.54 (d, *J* = 8.0 Hz, 2H), 7.42 – 7.32 (m, 7H), 5.34 (dd, *J* = 8.6, 7.7 Hz, 1H), 4.18 (dd, *J* = 8.6, 7.4 Hz, 2H), 3.57 – 3.45 (m, 1H), 3.39 (d, *J* = 8.2 Hz, 2H), 3.24 (q, *J* = 8.3 Hz, 1H). ^{13}C NMR (101 MHz, Chloroform-*d*) δ 158.0, 142.5, 136.8, 130.5 (q, *J* = 32.7 Hz), 128.9, 128.8, 128.1, 128.1, 127.1, 125.9 (q, *J* = 3.8 Hz), 122.7 (q, *J* = 272.2 Hz), 62.0, 57.2, 41.1, 36.8. ^{19}F NMR (376 MHz, Chloroform-*d*) δ -62.5. HRMS-ESI calcd for $C_{18}H_{16}F_3NO_2$ $[M+Na]^+$: 358.1025, found: 358.1028.

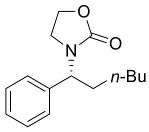

(*R*)-3-(1-phenylhexyl)oxazolidin-2-one(6a) Colorless oil, 99% yield. ^{1}H NMR (400 MHz, Chloroform-*d*) δ 7.44 – 7.27 (m, 5H), 4.99 (dd, *J* = 8.6, 7.2 Hz, 1H), 4.35 – 4.15 (m, 2H), 3.54 – 3.39 (m, 1H), 3.25 – 3.11 (m, 1H), 2.02 – 1.85 (m, 2H), 1.40 – 1.26 (m, 6H), 0.89 (t, *J* = 7.1 Hz, 3H). ^{13}C NMR (101 MHz, Chloroform-*d*) δ 158.3, 138.9, 128.8, 128.1, 127.8, 62.0, 56.6, 40.3, 31.7, 30.4, 26.2, 22.7, 14.1. HRMS-ESI calcd for $C_{15}H_{21}NO_2$ $[M+Na]^+$: 270.1465, found: 270.1479.

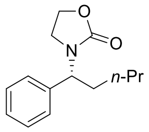

(*R*)-3-(1-phenylpentyl)oxazolidin-2-one(6b) Colorless oil, 92% yield. ^{1}H NMR (400 MHz, Chloroform-*d*) δ 7.39 – 7.27 (m, 5H), 4.98 (dd, *J* = 8.5, 7.3 Hz, 1H), 4.33 – 4.25 (m, 1H), 4.24 – 4.17 (m, 1H), 3.51 – 3.43 (m, 1H), 3.22 – 3.14 (m, 1H), 2.03 – 1.87 (m, 2H), 1.48 – 1.28 (m, 4H), 0.92 (t, *J* = 7.1 Hz, 3H). ^{13}C NMR (101 MHz, Chloroform-*d*) δ 158.13, 138.74, 128.64, 127.87, 127.55, 61.82, 56.36, 40.09, 29.89, 28.48, 22.36, 13.92. HRMS-ESI calcd for $C_{14}H_{19}NO_2$ $[M+Na]^+$: 256.1308, found: 256.1313.

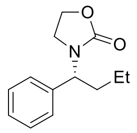

(*R*)-3-(1-phenylbutyl)oxazolidin-2-one(6c) Colorless oil, 93% yield. ^{1}H NMR (400 MHz, Chloroform-*d*) δ 7.38 – 7.27 (m, 5H), 5.00 (s, 1H), 4.33 – 4.14 (m, 2H), 3.52 – 3.40 (m, 1H), 3.24 – 3.14 (m, 1H), 1.99 – 1.88 (m, 2H), 1.46 – 1.32 (m, 2H), 0.99 (t, *J* = 7.4 Hz, 3H). ^{13}C NMR (101 MHz, Chloroform-*d*) δ 158.3, 138.9, 128.8, 128.1, 127.7, 62.0, 56.2, 40.2, 32.5, 19.7, 13.9. HRMS-ESI calcd for $C_{13}H_{17}NO_2$ [M+Na]$^+$: 242.1151, found: 242.1165.

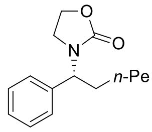

(*R*)-3-(1-phenylheptyl)oxazolidin-2-one(6d) Colorless oil, 93% yield. Colorless oil, 98% yield. ^{1}H NMR (400 MHz, Chloroform-*d*) δ 7.40 – 7.27 (m, 5H), δ 5.03 – 4.92 (m, 1H), 4.37 – 4.09 (m, 2H), 3.53 – 3.42 (m, 1H), 3.29 – 3.13 (m, 1H), 2.04 – 1.84 (m, 2H), 1.45 – 1.20 (m, 8H), 0.88 (t, *J* = 6.9 Hz, 3H). ^{13}C NMR (101 MHz, Chloroform-*d*) δ 158.3, 138.9, 128.8, 128.1, 127.7, 62.0, 56.6, 40.3, 31.8, 30.4, 29.1, 26.5, 22.7, 14.2. HRMS-ESI calcd for $C_{16}H_{23}NO_2$ [M+Na]$^+$: 284.1621, found: 284.1641.

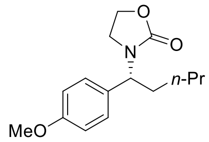

(*R*)-3-(1-(4-methoxyphenyl)pentyl)oxazolidin-2-one(6e) Colorless oil, 95% yield. ^{1}H NMR (400 MHz, Chloroform-*d*) δ 7.31 – 7.21 (m, 2H), 6.92 – 6.82 (m, 2H), 4.93 (t, *J* = 7.9 Hz, 1H), 4.33 – 4.14 (m, 2H), 3.44 (td, *J* = 8.8, 6.1 Hz, 1H), 3.23 – 3.09 (m, 1H), 1.98 – 1.84 (m, 3H), 1.47 – 1.23 (m, 5H), 0.91 (t, *J* = 7.1 Hz, 2H). ^{13}C NMR (101 MHz, Chloroform-*d*) δ 159.3, 158.3, 131.0, 128.9, 114.1, 62.0, 56.0, 55.4, 40.2, 30.3, 28.7, 22.5, 14.1. HRMS-ESI calcd for $C_{15}H_{21}NO_3$ [M+Na]$^+$: 286.1414, found: 286.1389.

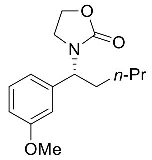

(*R*)-3-(1-(3-methoxyphenyl)pentyl)oxazolidin-2-one(6f) Colorless oil, 90% yield. ^{1}H NMR (400 MHz, Chloroform-*d*) δ 7.27 (t, *J* = 7.9 Hz, 1H), 6.93 – 6.81 (m, 3H), 4.95 (dd, *J* = 8.7, 7.1 Hz, 1H), 4.37 – 4.09 (m, 2H), 3.50 – 3.42 (m, 1H), 3.24 – 3.15 (m, 1H), 2.00 – 1.80 (m, 2H), 1.49 – 1.18 (m, 4H), 0.91 (t, *J* = 7.0 Hz, 3H). ^{13}C NMR (101 MHz, Chloroform-*d*) δ 160.0, 158.3, 140.5, 129.8, 119.9, 113.8, 113.2, 62.0, 56.5, 55.4, 40.3, 30.1, 28.6, 22.6, 14.1. HRMS-ESI calcd for $C_{15}H_{21}NO_3$ [M+Na]$^+$: 286.1414, found: 286.1420.

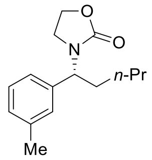

(*R*)-3-(1-(*m*-tolyl)pentyl)oxazolidin-2-one(6g) Colorless oil, 99% yield. ^{1}H NMR (400 MHz, Chloroform-*d*) δ 7.23 (t, *J* = 7.5 Hz, 1H), 7.16 – 7.07 (m, 3H), 4.94 (t, *J* = 7.9 Hz, 1H), 4.33 – 4.23 (m, 1H), 4.23 – 4.14 (m, 1H), 3.52 – 3.41 (m, 1H), 3.25 – 3.12 (m, 1H), 2.34 (s, 3H), 1.99 – 1.86 (m, 2H), 1.46 – 1.26 (m, 4H), 0.91 (t, *J* = 7.1 Hz, 3H). ^{13}C NMR (101 MHz, Chloroform-*d*) δ 158.12, 138.63, 138.35, 128.59, 128.49, 128.42, 124.31, 61.79, 56.30, 40.05,

29.86, 28.48, 22.35, 21.40, 13.91. HRMS-ESI calcd for $C_{15}H_{21}NO_2$ $[M+Na]^+$: 270.1465, found: 270.1467.

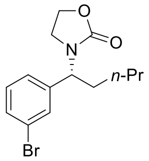

(*R*)-3-(1-(3-bromophenyl)pentyl)oxazolidin-2-one(6h) Colorless oil, 97% yield. ^{1}H NMR (400 MHz, Chloroform-*d*) δ 7.49 – 7.41 (m, 2H), 7.30 – 7.19 (m, 2H), 4.94 (t, *J* = 7.9 Hz, 1H), 4.35 – 4.18 (m, 2H), 3.47 (td, *J* = 8.8, 6.2 Hz, 1H), 3.25 – 3.15 (m, 1H), 1.91 (q, *J* = 8.4, 8.0 Hz, 1H), 1.46 – 1.24 (m, 4H), 0.92 (t, *J* = 7.1 Hz, 3H). ^{13}C NMR (101 MHz, Chloroform-*d*) δ 158.05, 141.19, 131.05, 130.29, 126.32, 122.79, 61.87, 55.88, 40.04, 29.80, 28.38, 22.29, 13.89. HRMS-ESI calcd for $C_{14}H_{18}BrNO_2$ $[M+Na]^+$: 334.0413, found: 334.0419.

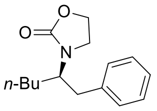

(*R*)-3-(1-phenylhexan-2-yl)oxazolidin-2-one(7a) Colorless oil, 99% yield. ^{1}H NMR (400 MHz, Chloroform-*d*) δ 7.32 – 7.26 (m, 2H), 7.24 – 7.17(m, 3H), 4.19 (dtd, *J* = 31.0, 8.7, 7.0 Hz, 2H), 4.09– 3.95(m, 1H), 3.45– 3.28 (m, 2H), 2.90 – 2.74 (m, 2H), 1.62 – 1.50 (m, 2H), 1.41– 1.20 (m, 4H), 0.88 (t, *J* = 7.0 Hz, 3H). ^{13}C NMR (101 MHz, Chloroform-*d*) δ 158.2, 138.0, 129.0, 128.6, 126.7, 62.0, 54.7, 40.7, 39.2, 31.6, 28.6, 22.5, 14.1, HRMS-ESI calcd for $C_{15}H_{21}NO_2$ $[M+Na]^+$: 270.1470, found: 270.1459.

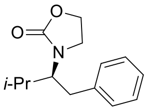

(*S*)-3-(3-methyl-1-phenylbutan-2-yl)oxazolidin-2-one(7b) Colorless oil, 93% yield. ^{1}H NMR (400 MHz, Chloroform-*d*) δ 7.31 – 7.24 (m, 2H), 7.20 (dt, *J* = 5.9, 1.4 Hz, 3H), 4.19 – 4.09 (m, 1H), 4.05 – 3.91 (m, 1H), 3.80 – 3.67 (m, 1H), 3.41 – 3.21 (m, 2H), 3.12 (dd, *J* = 14.6, 4.4 Hz, 1H), 2.67 (dd, *J* = 14.6, 11.4 Hz, 1H), 1.97 – 1.79 (m, 1H), 1.08 (d, *J* = 6.7 Hz, 3H), 0.97 (d, *J* = 6.7 Hz, 3H). ^{13}C NMR (101 MHz, Chloroform-*d*) δ 158.4, 138.2, 128.6, 126.6, 61.9, 60.5, 41.5, 36.1, 31.1, 20.2, 19.9. HRMS-ESI calcd for $C_{14}H_{19}NO_2$ $[M+Na]^+$: 256.1313, found: 256.1308.

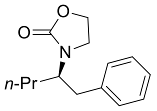

(*R*)-3-(1-phenylpentan-2-yl)oxazolidin-2-one(7c) Colorless oil, 92% yield. ^{1}H NMR (400 MHz, Chloroform-*d*) δ 7.31 – 7.26 (m, 2H), 7.23 – 7.18 (m, 3H), 4.26 – 4.02 (m, 3H), 3.43 – 3.29 (m, 2H), 2.87 – 2.76 (m, 2H), 1.63 – 1.46 (m, 2H), 1.42 – 1.24 (m, 2H), 0.92 (t, *J* = 7.3 Hz, 3H). ^{13}C NMR (101 MHz, Chloroform-*d*) δ 158.2, 138.0, 129.0, 128.6, 126.7, 62.0, 54.4, 40.7, 39.2, 34.0, 19.7, 13.9, HRMS-ESI calcd for $C_{14}H_{19}NO_2$ $[M+Na]^+$: 256.1313, found: 256.1317.

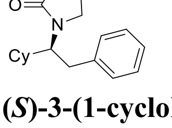

(*S*)-3-(1-cyclohexyl-2-phenylethyl)oxazolidin-2-one(7d) Colorless oil, 90% yield. ^{1}H NMR (400 MHz, Chloroform-*d*) δ 7.31 – 7.26 (m, 2H), 7.20 (s, 3H), 4.13 (ddd, *J* = 9.3, 8.4, 6.2 Hz, 1H), 4.03 – 3.95 (m, 1H), 3.78 (ddd, *J* = 11.4, 9.4, 4.5 Hz, 1H), 3.34 (ddd, *J* = 9.3, 8.3, 6.2 Hz, 1H), 3.29 – 3.20 (m, 1H), 3.13 (dd, *J* = 14.6, 4.5 Hz, 1H), 2.68 (dd, *J* = 14.6, 11.4 Hz,

1H), 1.96 – 1.88 (m, 1H), 1.85 – 1.55 (m, 5H), 1.36 – 1.00 (m, 5H). ^{13}C NMR (101 MHz, Chloroform-*d*) δ 158.4, 138.4, 128.7, 128.7, 126.6, 61.9, 59.6, 42.0, 40.4, 35.8, 30.6, 30.1, 26.4, 26.1, 26.1, HRMS-ESI calcd for $C_{17}H_{23}NO_2$ [M+Na]$^+$: 296.1626, found: 296.1615.

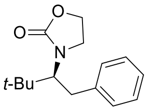

(*S*)-3-(3,3-dimethyl-1-phenylbutan-2-yl)oxazolidin-2-one(7e) Colorless oil, 93% yield. ^{1}H NMR (400 MHz, Chloroform-*d*) δ 7.32 – 7.26 (m, 2H), 7.23 – 7.16 (m, 3H), 4.26 – 4.00 (m, 2H), 3.97 – 3.74 (m, 1H), 3.70 – 3.52 (m, 1H), 3.51 – 3.40 (m, 1H), 3.17 – 3.00 (m, 1H), 2.87 – 2.60 (m, 1H), 1.08 (s, 9H). ^{13}C NMR (101 MHz, Chloroform-d) δ 159.2, 138.7, 128.8, 128.5, 126.7, 77.4, 62.0, 42.1, 35.9, 32.5, 27.7. HRMS-ESI calcd for $C_{15}H_{21}NO_2$ [M+Na]$^+$: 270.1465, found: 270.1478.

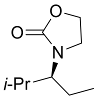

(*S*)-3-(2-methylpentan-3-yl)oxazolidin-2-one(7f) Colorless oil, 95% yield. ^{1}H NMR (400 MHz, Chloroform-*d*) δ 4.31 (dd, *J* = 8.4, 7.7 Hz, 2H), 3.41 (td, *J* = 7.8, 2.0 Hz, 2H), 3.33 (ddd, *J* = 11.4, 9.4, 3.7 Hz, 1H), 1.81 – 1.60 (m, 2H), 1.40 – 1.24 (m, 1H), 0.95 (d, *J* = 6.6 Hz, 3H), 0.91 – 0.83 (m, 6H). ^{13}C NMR (101 MHz, Chloroform-*d*) δ 159.1, 62.0, 61.2, 40.2, 31.0, 22.5, 20.0, 19.9, 11.0, HRMS-ESI calcd for $C_9H_{17}NO_2$ [M+Na]$^+$: 194.1151, found: 194.1156.

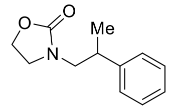

3-(2-phenylpropyl)oxazolidin-2-one (8a) Colorless oil, ^{1}H NMR (400 MHz, Chloroform-*d*) δ 7.35 – 7.27 (m, 2H), 7.27 – 7.19 (m, 3H), 4.14 (dd, *J* = 8.5, 7.6 Hz, 2H), 3.54 (dd, *J* = 13.9, 6.9 Hz, 1H), 3.35 – 3.21 (m, 2H), 3.18 – 3.00 (m, 2H), 1.28 (d, *J* = 7.0 Hz, 3H). ^{13}C NMR (101 MHz, $CDCl_3$) δ 158.5, 143.8, 128.6, 127.0, 126.7, 61.6, 51.2, 45.1, 38.6, 19.1. HRMS-ESI calcd for $C_{12}H_{15}NO_2$ [M+Na]$^+$: 228.0995, found: 228.1000.

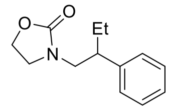

3-(2-phenylbutyl)oxazolidin-2-one (8b) Colorless oil, ^{1}H NMR (400 MHz, Chloroform-*d*) δ 7.35 – 7.27 (m, 2H), 7.27 – 7.19 (m, 3H), 4.14 (dd, *J* = 8.5, 7.6 Hz, 2H), 3.54 (dd, *J* = 13.9, 6.9 Hz, 1H), 3.35 – 3.21 (m, 2H), 3.18 – 3.00 (m, 2H), 1.60 – 1.50 (m, 2H), 0.95 (t, *J* = 7.3 Hz, 3H).. ^{13}C NMR (101 MHz, $CDCl_3$) δ 158.5, 143.7, 128.5, 127.0, 126.7, 62.0, 54.4, 40.7, 39.2, 24.0, 21.2 HRMS-ESI calcd for $C_{13}H_{17}NO_2$ [M+Na]$^+$: 242.1157, found: 242.1162.

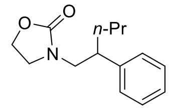

3-(2-phenylpentyl)oxazolidin-2-one (8c) Colorless oil, ^{1}H NMR (400 MHz, $CDCl_3$) δ 7.36 – 7.28 (m, 2H), 7.26 – 7.18 (m, 3H), 4.17 – 4.02 (m, 2H), 3.62 (dd, *J* = 13.9, 6.2 Hz, 1H), 3.31 – 3.17 (m, 2H), 3.07 – 2.97 (m, 1H), 2.90 (tt, *J* = 9.5, 5.8 Hz, 1H), 1.70 – 1.50 (m, 2H), 1.20 (h, *J* = 7.5 Hz, 2H), 0.85 (t, *J* = 7.3 Hz, 3H).^{13}C NMR (101 MHz, $CDCl_3$) δ 158.5, 143.7, 128.5 127.0, 126.7, 54.4, 46.0, 40.7, 39.2, 34.0, 19.9, 13.9. HRMS-ESI calcd for $C_{14}H_{19}NO_2$ [M+Na]$^+$: 256.1313, found: 256.1307.

4. General procedure for the preparation of iridium complexes

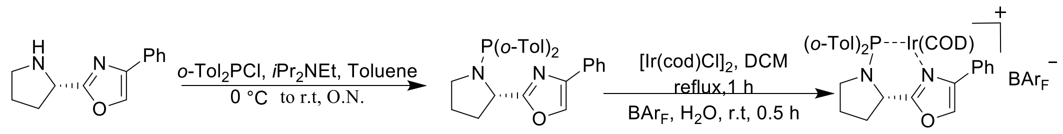

Amine (1 mmol) was co-evaporated with dry toluene (3 x 20 mL) and dissolved in dry toluene or THF (6 mL) under N_2. Freshly distilled di-*iso*-propylethylamine (3 mmol) was added and the solution was cooled to 0 °C in an ice-bath. Freshly distilled (prepared) suitable phosphine chloride (1.2 mmol) was dissolved in toluene (2 mL) and added dropwise to the reaction mixture and stirred at room temperature for 24 hours. After completion of the reaction, monitored by TLC, it was quenched with saturated $NaHCO_3$ solution and the reaction mixture was extracted in CH_2Cl_2 (3 x10 mL). The combined organic layers were dried over Na_2SO_4, filtered and concentrated under reduced pressure. The oily residual was quickly passed through a small pad of silica and used to make the Ir-complex without further characterization.

The synthesized oxazoline ligand was dissolved in CH_2Cl_2 (20 mL) and $[Ir(COD)Cl]_2$ (0.25 mmol) was added. The atmosphere in the flask was evacuated and replenished three times with N_2. The mixture was heated to reflux for 1 hour. After the solution was cooled to room temperature, distilled H_2O (20 mL) was added. Under vigorous stirring, $NaBAr_F.xH_2O$ (0.6 mmol) was added to the biphasic solution in one portion. The mixture was stirred vigorously for 30 minutes and extracted with CH_2Cl_2 (3 x10 mL). Combined organic phase was dried over Na_2SO_4. After concentration in vacuum, the residue was purified on silica gel with CH_2Cl_2: pentane (1 : 1) as the eluent to afford the iridium complex as an orange solid.

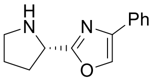

White solid. ^1H NMR (400 MHz, Chloroform-d) δ 7.83 (s, 1H), 7.77 – 7.68 (m, 2H), 7.42 – 7.33 (m, 2H), 7.33 – 7.26 (m, 1H), 4.39 (ddd, J = 7.6, 6.0, 1.2 Hz, 1H), 3.23 – 3.13 (m, 1H), 3.09 – 2.89 (m, 1H), 2.44 (s, 1H), 2.32 – 2.05 (m, 2H), 2.05 – 1.69 (m, 2H). ^{13}C NMR (101 MHz, Chloroform-*d*) δ 166.8, 140.6, 133.5, 131.2, 128.8, 128.03, 125.6, 55.74, 47.0, 31.0, 25.5. HRMS-EI calcd for $C_{13}H_{14}NO[M+Na]^+$: 727.2425, found: 727.2445.

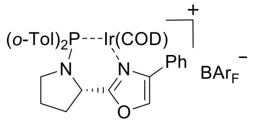

Orange solid. $[\alpha]_D^{25.0}$ = +51 (C= 0.10 in $CHCl_3$). ^1H NMR (400 MHz, Chloroform-*d*): δ 7.73 (p, *J* = 2.2 Hz, 8H), 7.62 (s, 1H), 7.52 (s, 5H), 7.43 (q, *J* = 8.4, 7.7 Hz, 4H), 7.32 (d, *J* = 7.7 Hz, 3H), 7.25 – 7.20 (m, 1H), 7.11 (dd, *J* = 12.7, 7.9 Hz, 2H), 6.98 (d, *J* = 7.6 Hz, 2H), 6.66 (t, *J* = 9.2 Hz, 1H), 5.22 (t, *J* = 7.1 Hz, 1H), 4.73 – 4.56 (m, 1H), 4.20 (s, 1H), 3.62 – 3.48 (m, 1H), 3.33 (t, *J* = 6.8 Hz, 1H), 3.07 (dd, *J* = 11.2, 5.4 Hz, 1H), 2.86 (s, 3H), 2.74 (s, 3H), 2.65 (td, *J* = 11.9, 10.0, 5.4 Hz, 2H), 2.49 (dd, *J* = 13.5, 6.9 Hz, 1H), 2.28 – 1.82 (m, 6H), 1.68 (d, *J* = 10.9 Hz, 1H), 1.41 (td, *J* = 9.8, 8.7, 4.7 Hz, 2H). ^{13}C NMR (100 MHz, Chloroform-*d*) δ 165.6, 161.8 (ddd, *J* = 99.7, 49.8, 3.2 Hz), 141.9, 140.9 (dd, *J* = 105.7, 14.0 Hz), 136.1, 134.9, 133.3 – 132.2 (m), 131.8, 131.6 (d, *J* = 47.2 Hz), 130.7, 129.4, 129.1 (dt, *J* = 5.8, 3.1 Hz), 129.0 – 128.6 (m), 128.5 (d, *J* = 3.6 Hz), 128.0, 127.5 (d, *J* = 7.4 Hz), 126.9 (d, *J* = 10.1 Hz), 126.4, 126.0 (d, *J* = 2.2 Hz), 123.3 (d, *J* = 2.2 Hz), 121.8 (d, *J* = 62.7 Hz), 120.6, 117.6, 94.5 (d, *J* = 10.9 Hz), 86.9 (d, *J* = 14.0 Hz), 69.7, 68.0, 59.0 (d, *J* = 11.5 Hz), 47.8, 34.3, 31.0 (d, *J* = 170.9 Hz), 27.9 – 26.6 (m), 26.4, 25.1 (d, *J* = 6.3 Hz), 21.3 (d, *J* = 6.7 Hz). ^{31}P NMR (162

MHz, Chloroform-*d*) δ 51.1 (s). HRMS-EI calcd for $C_{35}H_{40}IrN_2OP$ $[M]^+$: 727.2425, found: 727.2445.

5. Separation method data and specific rotation of chiral compounds

Entry	Product	Separation method	Optical rotation	*ee* (%)
1	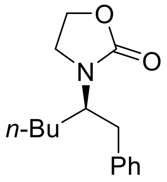	**SFC**, OZH column, 10% MeOH, 2 ml/min, t_R = 7.4 min (major)/8.2 (minor)	$[\alpha]_D^{25.0}$ = -18 (C= 0.10 in $CHCl_3$)	95
2	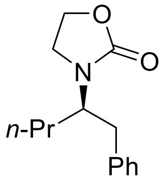	**SFC**, IF column, 15% MeOH, 2 ml/min, t_R = 5.8 min (minor)/6.1 (major)	$[\alpha]_D^{25.0}$ = -23 (C= 0.10 in $CHCl_3$)	93
3	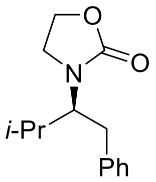	**SFC**, ASH column, 15% MeOH, 2 ml/min, t_R = 2.8 min (minor)/2.9 (major)	$[\alpha]_D^{25.0}$ = -45 (C= 0.10 in $CHCl_3$)	96
4	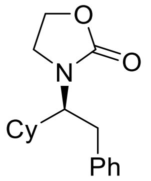	**SFC**, OZH column, 15% MeOH, 2 ml/min, t_R = 7.5 min (major)/8.4 (minor)	$[\alpha]_D^{25.0}$ = -22 (C= 0.10 in $CHCl_3$)	96
5	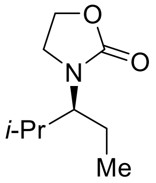	**GC-MS**, Hydrodex β-3P, 50 °C to 175 °C, 1 degree/min, t_R = 87.6 min (minor)/88.7 (major)	$[\alpha]_D^{25.0}$ = -4 (C= 0.10 in $CHCl_3$)	93
6	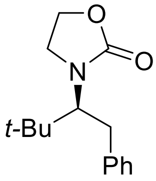	**SFC**, IF column, 15% MeOH, 2 ml/min, t_R = 5.3 min (minor)/5.8 (major)	$[\alpha]_D^{25.0}$ = -12 (C= 0.10 in $CHCl_3$)	99
7	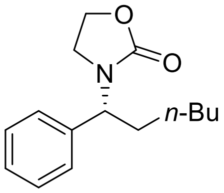	**SFC**, IF column, 15% MeOH, 2 ml/min, t_R = 6.0 min (major)/7.7 (minor)	$[\alpha]_D^{25.0}$ = +165 (C= 0.73 in $CHCl_3$)	92
8	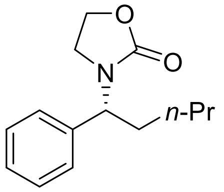	**SFC**, IF column, 15% MeOH, 2 ml/min, t_R = 5.5 min (major)/6.3 (minor)	$[\alpha]_D^{25.0}$ = +106.8 (C = 0.54 in $CHCl_3$)	89

9	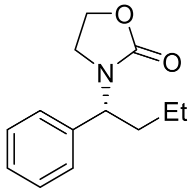	**HPLC**, ASH column, 25% *i*PrOH in hexane, 0.5 ml/min, t_R = 30.5 min (major)/40.8 min (minor)	$[\alpha]_D^{25.0}$ = +97 (C= 0.10 in $CHCl_3$)	88
10	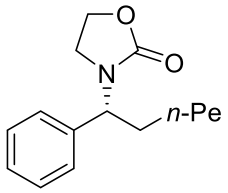	**SFC**, IF column, 15% MeOH, 2 ml/min, t_R = 6.1 min (major)/7.1 (minor)	$[\alpha]_D^{25.0}$ = +101 (C = 0.10 in $CHCl_3$)	93
11	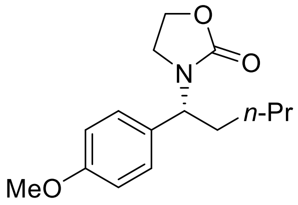	**SFC**, IF column, ASH column, 25% *i*PrOH in hexane, t_R = 45.3 min (major)/53.6 (minor)	$[\alpha]_D^{25.0}$ = +180 (C= 0.10in $CDCl_3$)	93
12	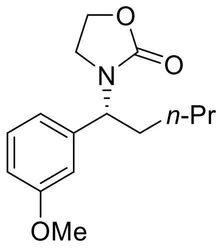	**SFC**, IF column, 10% MeOH, 2 ml/min, t_R = 11.0 min (major)/13.7 (minor)	$[\alpha]_D^{25.0}$ = +63 (C= 0.10 in $CHCl_3$)	93
13	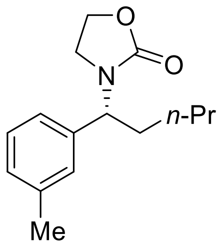	**SFC**, ASH column, 15% MeOH, 2 ml/min, t_R = 3.2 min (minor)/3.6 (major)	$[\alpha]_D^{25.0}$ = +100.7 (C = 0.77 in $CHCl_3$)	95
14	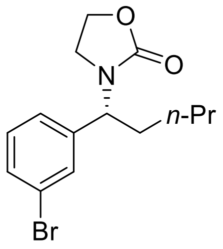	**SFC**, ASH column, 15% MeOH, 2 ml/min, t_R = 4.4 min (minor)/5.0 (major)	$[\alpha]_D^{25.0}$ = +85.6 (C= 0.73 in $CHCl_3$)	91
15	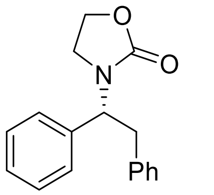	**SFC**, IF column, 15% MeOH, 2 ml/min, t_R = 12.2 min (major) / 13.2 (minor)	$[\alpha]_D^{25.0}$ = +82 (C= 0.10 in $CHCl_3$)	93
16	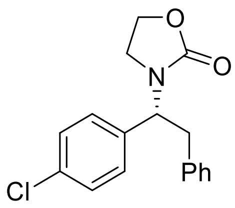	**SFC**, IF column, 15% MeOH, 2 ml/min, t_R = 14.6 min (minor)/ 14.9 (major)	$[\alpha]_D^{25.0}$ = +67 (C= 0.45in $CHCl_3$)	97

17	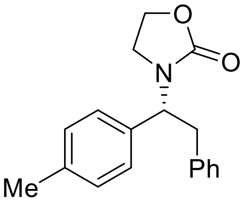	**SFC**, OJH column, 15% MeOH, 2 ml/min, t_R = 5.3 min (major)/ 9.3(minor)	$[\alpha]_D^{25.0}$ = +82 (C= 0.10 in $CHCl_3$)	92
18	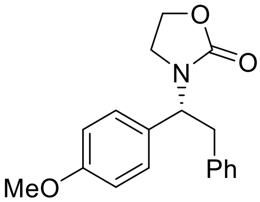	**SFC**, OZH column, 10% MeOH, 2 ml/min, t_R = 7.0 min (minor)/7.8 (major)	$[\alpha]_D^{25.0}$ = +90(C= 0.10 in $CHCl_3$)	94
19	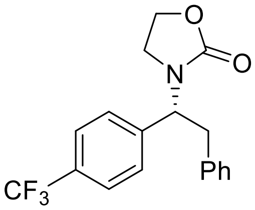	**SFC**, OZH column, 15% MeOH, 2 ml/min, t_R = 4.5 min (minor)/5.0 (major)	$[\alpha]_D^{25.0}$ = +68(C= 0.10 in $CHCl_3$)	95
20	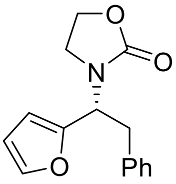	**SFC**, ODH column, 15% MeOH, 2 ml/min, t_R = 4.4 min (major)/4.6 (minor)	$[\alpha]_D^{25.0}$ = +83(C= 0.10 in $CHCl_3$)	99
21	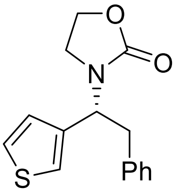	**SFC**, IF column, 15% MeOH, 2 ml/min, t_R = 16.5 min (major)/ 18.0 (minor)	$[\alpha]_D^{25.0}$ = +78 (C= 0.10 in $CHCl_3$)	88
22	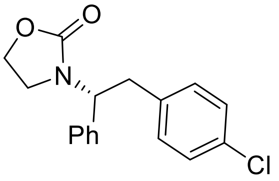	**SFC**, ASH column, 15% MeOH, 2 ml/min, t_R = 6.9 min (minor)/9.3 (major)	$[\alpha]_D^{25.0}$ = +79 (C= 0.10 in $CHCl_3$)	92
23	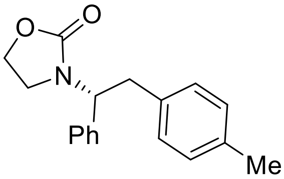	**SFC**, ASH column, 15% MeOH, 2 ml/min, t_R = 6.1 min (minor)/7.2 (major)	$[\alpha]_D^{25.0}$ = +37 (C= 0.10 in $CHCl_3$)	92
24	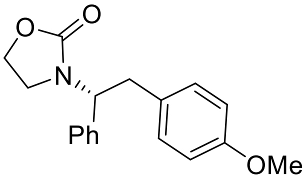	**SFC**, ASH column, 15% MeOH, 2 ml/min, t_R = 6.4 min (minor)/7.5 (major)	$[\alpha]_D^{25.0}$ = +67 (C= 0.10 in $CHCl_3$)	95
25	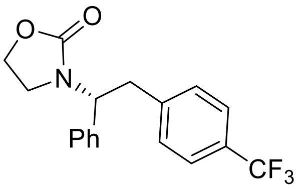	**SFC**, IF column, 15% MeOH, 2 ml/min, t_R = 6.0 min (minor)/6.6 (major)	$[\alpha]_D^{25.0}$ = +73 (C= 0.10 in $CHCl_3$)	96

26	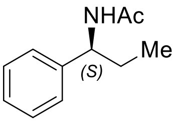	**SFC**, ADH column, 15% MeOH, 2 ml/min, t_R = 5.7 min (minor)/6.6 (major)	$[\alpha]_D^{25.0}$ = -110.5 (C= 0.20 in $CHCl_3$)	98
27	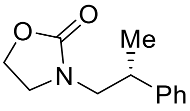	**SFC**, ASH column, 15% MeOH, 2 ml/min, 15min, t_R = 4.7 min (minor)/5.2 min (major)	$[\alpha]_D^{25.0}$ = -31 (C= 0.1 in $CHCl_3$)	97
28	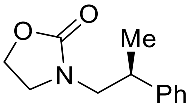	**SFC**, ASH column, 15% MeOH, 2 ml/min, 15min, t_R = 4.7 min (majorr)/5.2 min (minor)	$[\alpha]_D^{25.0}$ = 31 (C= 0.1 in $CHCl_3$)	97
29	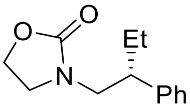	**SFC**, IF column, 15% MeOH, 2 ml/min, t_R = 5.1 min (minor)/5.5 (major)	$[\alpha]_D^{25.0}$ = -22 (C= 0.10 in $CHCl_3$)	96
30	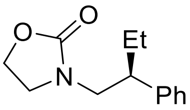	**SFC**, IF column, 15% MeOH, 2 ml/min, t_R = 5.1 min (minor)/5.5 (major)	$[\alpha]_D^{25.0}$ = 21 (C= 0.10 in $CHCl_3$)	93
31	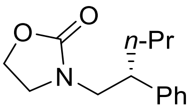	**SFC**, IF column, 15% MeOH, 2 ml/min, t_R = 5.4 min (minor)/5.7 (major)	$[\alpha]_D^{25.0}$ = -25 (C= 0.10 in $CHCl_3$)	96
32	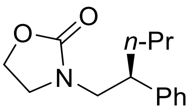	**SFC**, IF column, 15% MeOH, 2 ml/min, t_R = 5.4 min (minor)/5.7 (major)	$[\alpha]_D^{25.0}$ = 22 (C= 0.10 in $CHCl_3$)	90

6. Absolute configuration determination

6.1 The absolute configuration of the different classes of hydrogenated products

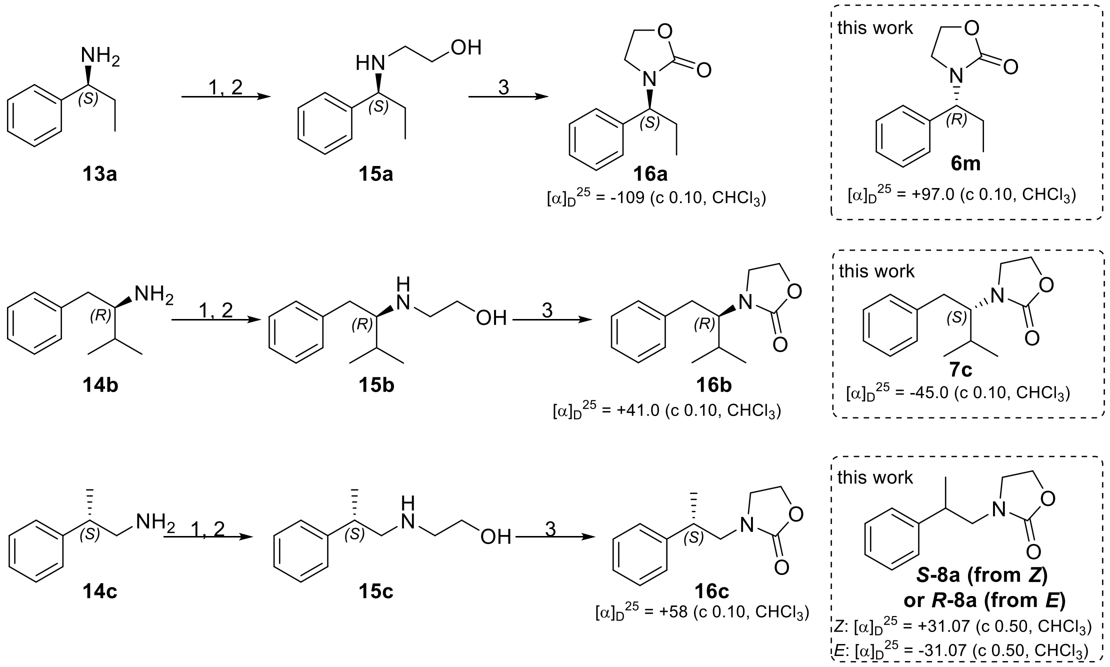

Reaction condition (1) ethyl 2-chloro-2-oxoacetate, NEt_3, CH_2Cl_2, r.t., 2 h. (2) LAH, THF, reflux. o.n. (3) triphosgene, NEt_3, rt. o.n.

The absolute configuration of the different classes of hydrogenated product was determined by transformation of known compounds **13a, 14b**, **14c** into compound **15a-15c** and comparison with hydrogenated products **6m**, **7c**, ***S*-8a** and ***Z*-8a**. First, the animes **12a-12c** were treated with ethyl 2-chloro-2-oxoacetate to furnish compounds **13a, 14b**, **14c**. Then a cyclization using triphosgene and NEt_3 afforded compound **15a-15c**, of which optical rotation were compared to hydrogenated products **6m**, **7c**, ***S*-8a** and ***Z*-8a**. The absolute configuration of the other products has been tentatively assigned by comparison of elution order from HPLC and SFC, using a chiral stationary phase and the optical rotation of products **6m**, **7c**, ***S*-8a** and ***Z*-8a**.

6.2 Di-aryl enamides by single crystal X-ray diffraction

Single crystal of **5a** was grown by dissolving in pentane diethyl ether and slow evaporation at room temperature. Detailed information for this crystal is available in CCDC 1955080.

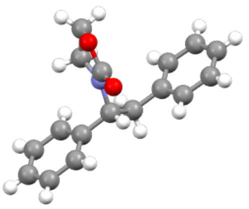

X-ray crystal structure of **5.**

7. Conditions screening

Table S1 The catalyst screening [a]

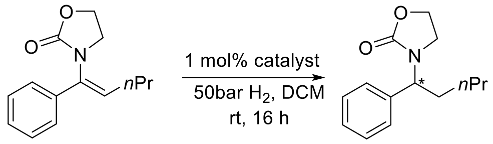

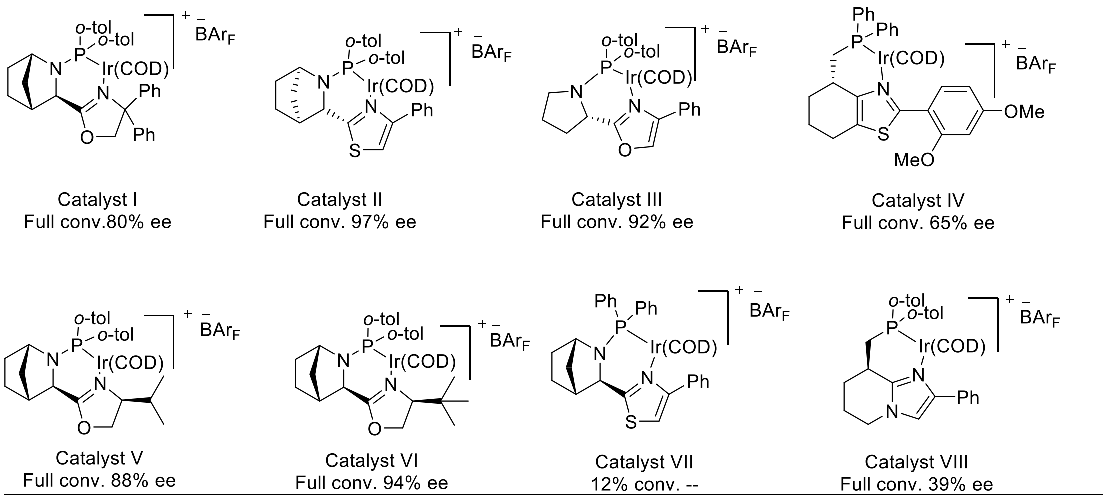

[a] 0.05 mmol of substrates in 0.5 mL of DCM. Conversion was determined by ^{1}H NMR spectroscopy. Enantiomeric excess was determined by SFC analysis, using chiral stationary phases.

Table S2 The solvent screening [a]

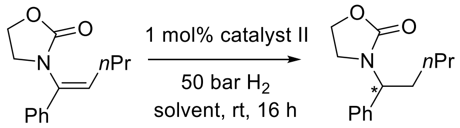

Entry	Solvent	Conv.(%)	ee (%)
1	DCM	Full	97
2	Toluene	Full	97
3	PhCF3	Full	97
4	Benzene	Full	97
5	DCE	Full	97

[a] 0.05 mmol of substrates in 0.5 mL of solvent. Conversion was determined by ^{1}H NMR spectroscopy. Enantiomeric excess was determined by SFC analysis, using chiral stationary phases.

Table S3 The condition screening of class 1

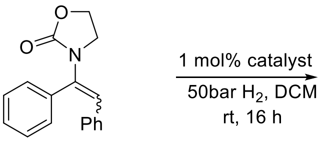

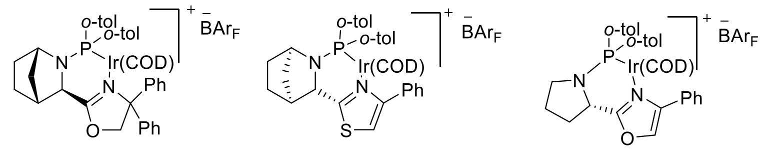

Catalyst I	Catalyst II	Catalyst III
E: Full conv.96% ee *(R)*	*E:* Full conv. 55% ee *(S)*	*E*: Full conv.78% ee *(S)*
Z: Full conv.93% ee *(R)*	*Z:* Full conv. 26% ee *(S)*	*Z*: Full conv. 4% ee *(S)*

[a] 0.05 mmol of substrates in 0.5 mL of DCM. Conversion was determined by ^{1}H NMR spectroscopy. Enantiomeric excess was determined by SFC analysis, using chiral stationary phase

Table S4 The additional condition screening of class 2 [a]

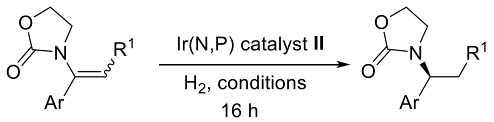

Entry	Subs.	Solvent	Press.(bar)	Temp.(°C)	Conv.(%)	ee(%)
1	**(*E*)-2c**	DCM	50	r.t	99	43(S)
	**(*Z*)-2c**	DCM	50	r.t	99	98(R)
2	**(*E*)-2c**	Toluene	50	60	99	10(R)
	**(*Z*)-2c**	Toluene	50	60	99	97(R)
3	**(*E*)-2c**	Toluene	3	60	99	47(R)
	**(*Z*)-2c**	Toluene	3	60	99	97(R)
4	**(*E*)-2c**	Toluene	1	60	76	60(R)
	**(*Z*)-2c**	Toluene	1	60	99	97(R)
5	**(*E*)-2c**	DCE	1	60	99	63(R)
	**(*Z*)-2c**	DCE	1	60	99	97(R)
6	**(*E*)-2a**	DCE	1	60	99	80(R)
	**(*Z*)-2a**	DCE	1	60	99	97(R)

[a] 0.05 mmol of substrates in 0.5 mL of solvent. Conversion was determined by ^{1}H NMR spectroscopy. Enantiomeric excess was determined by SFC analysis, using chiral stationary phase

Table S5 The condition screening of class 3 [a]

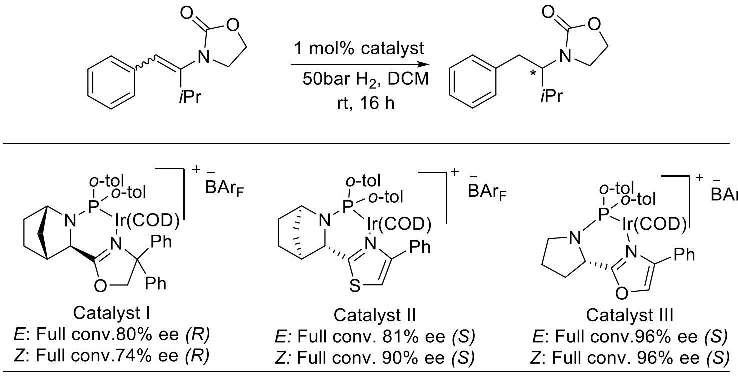

[a] 0.05 mmol of substrates in 0.5 mL of DCM. Conversion was determined by ^{1}H NMR spectroscopy. Enantiomeric excess was determined by SFC analysis, using chiral stationary phase

8. Mechanistic study

Table S6 Kinetic study on hydrogenation of separated geometric isomer

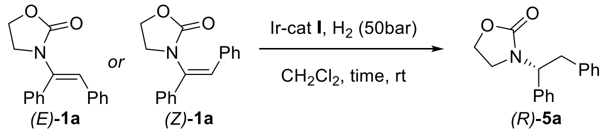

Entry	Time (min)	*E*		*Z*	
		Z/E (ratio)	conv.to **5a**	*Z/E* (ratio)	conv.to **5a**
1	5	1:1.1	22%	5.8:1	10%
2	15	1:1.1	31%	5.9:1	27%
3	30	5:1	51%	6:1	31%
4	60	6:1	69%	6:1	40%

Table S7 Acid catalyzed isomerization

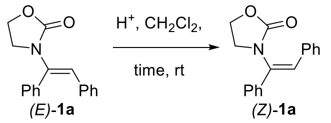

Entry	H^+ source	*Z/E* (ratio)	Time (days)
1	AcOH (100 mol%)	2:1	14
2	TCA (100 mol%)	6:1	7
3	TCA (5 mol%)	5:1	14
4	Ir-H (5 mol%)	6:1	8

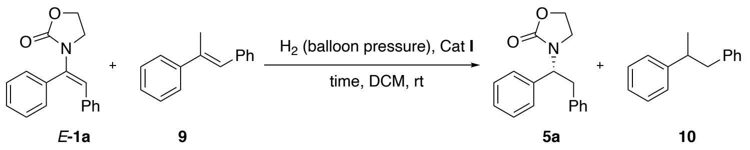

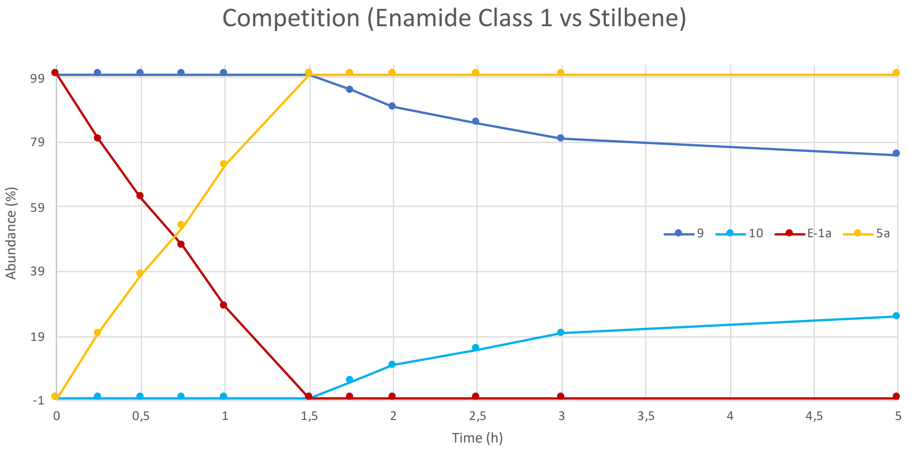

Figure S1 Competition experiments for class 1 enamide and trans methyl stilbene.

Labeling study

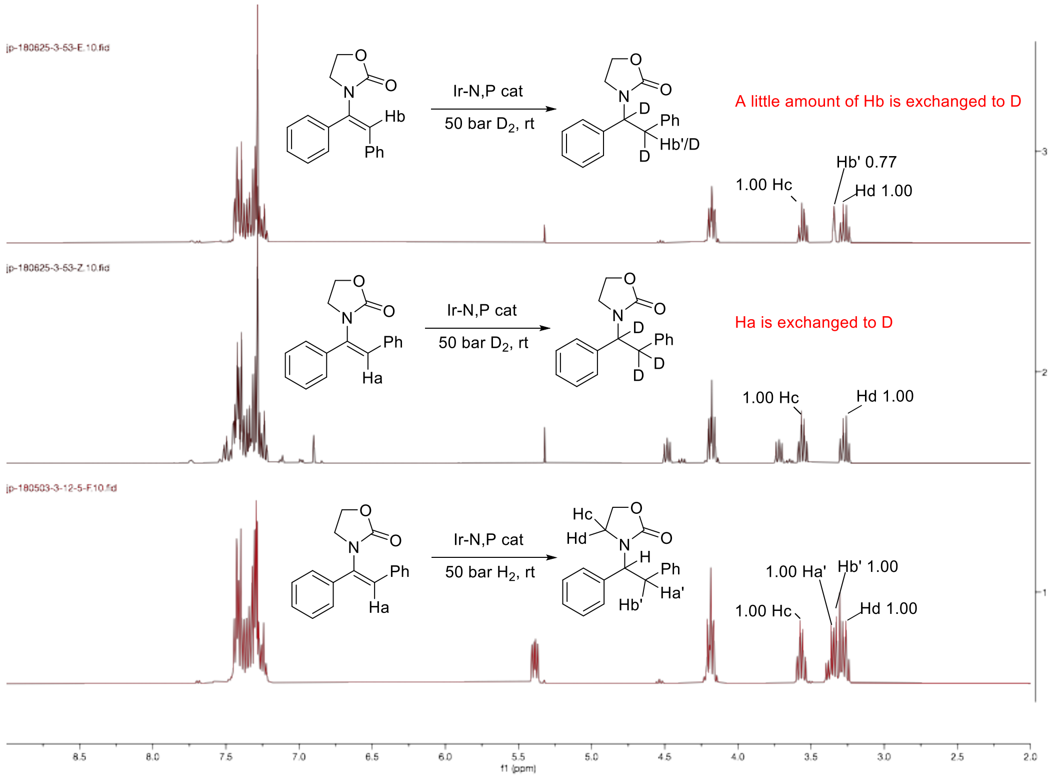

Figure S2 Deuterium study on **1a**

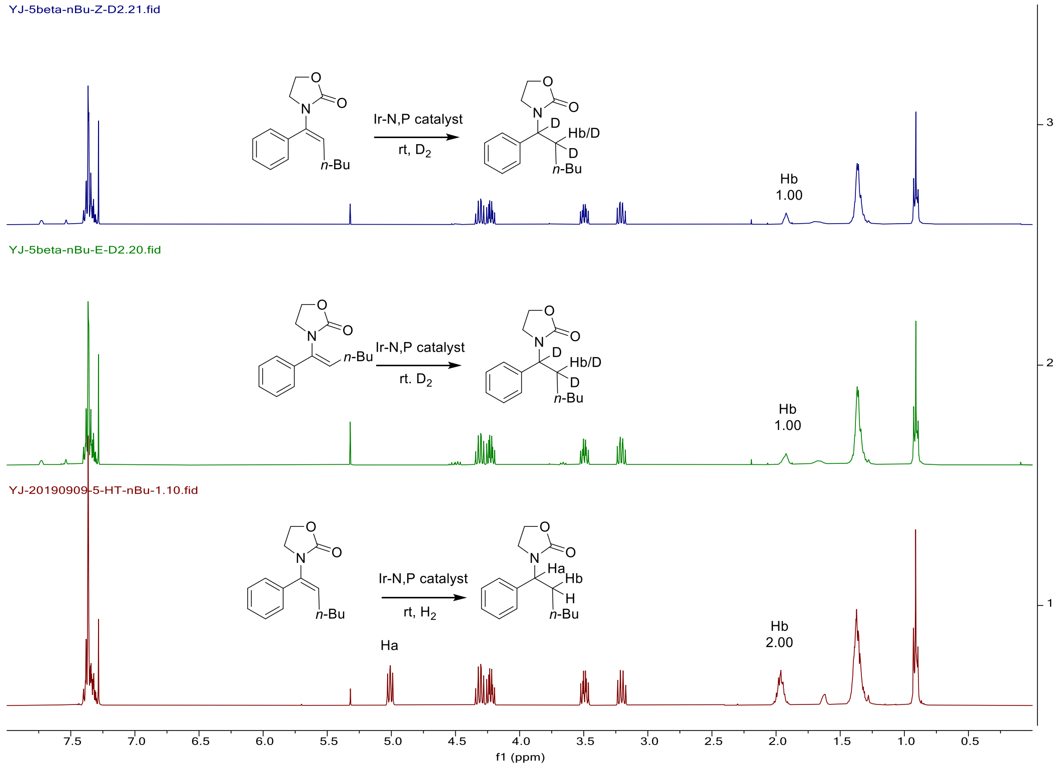

Figure S3 Deuterium study on **2a** (rt, 50bar)

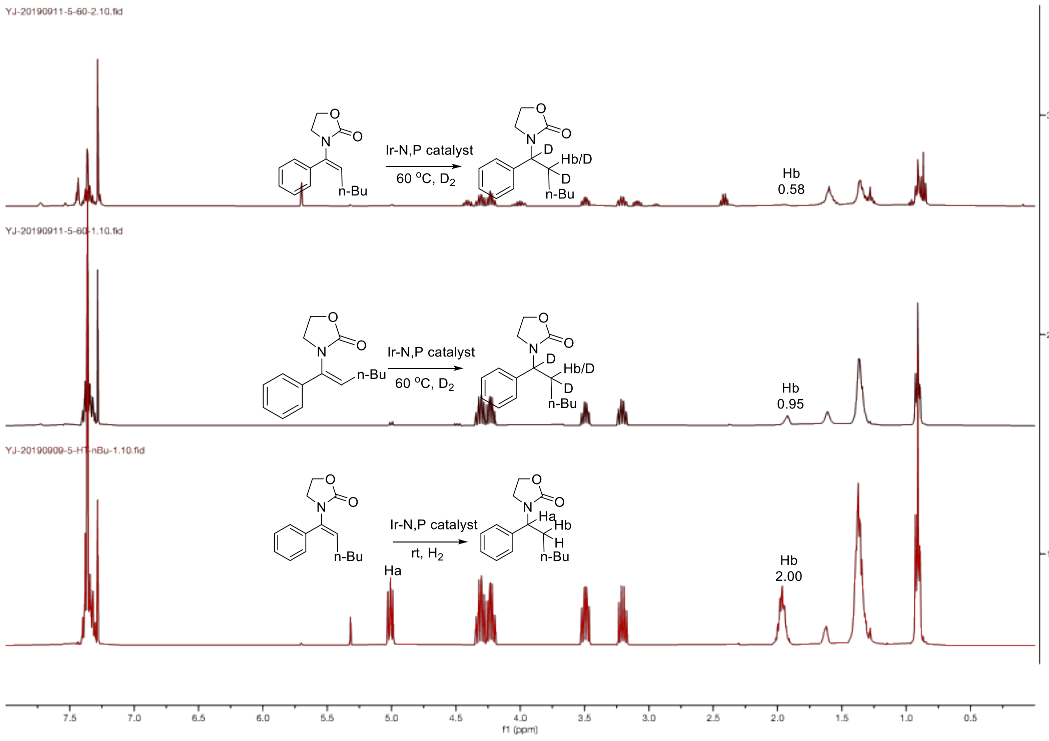

Figure S4 Deuterium study on **2a** (60 °C, 1 bar)

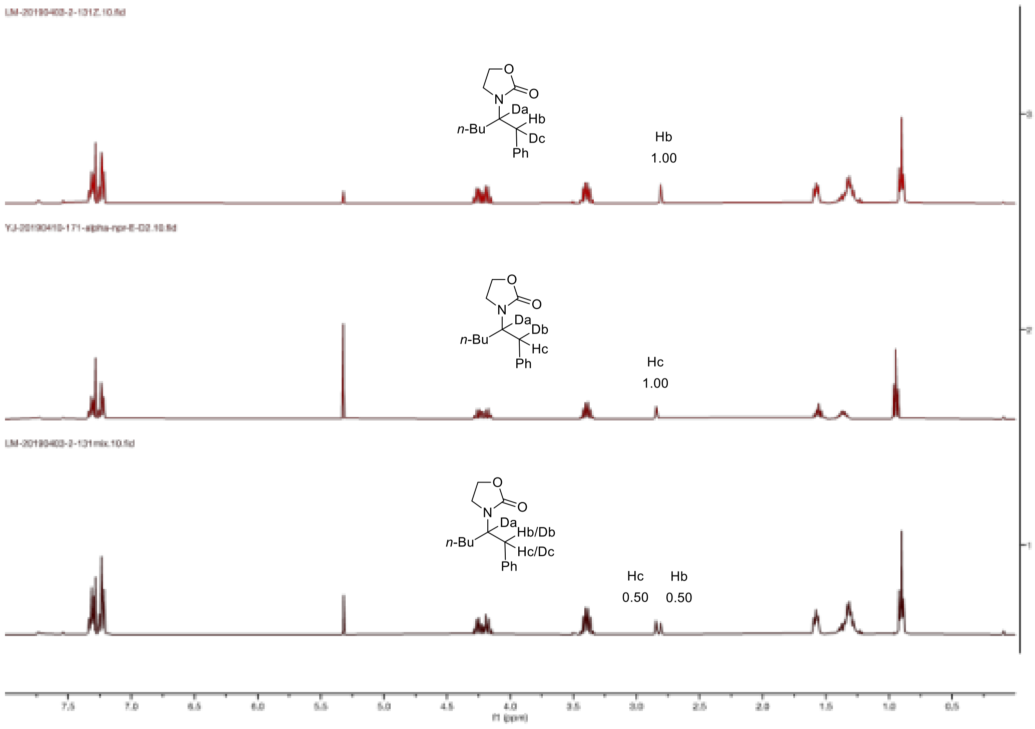

Figure S5 Deuterium study on **3a** (rt)

9. DTF computational details

Single point free energy with solvation (dichloromethane) were carried out with Jaguar[13] (version 10.1) using the B3LYP-D3[14,15] functional in combination with the psLACVP** basis set.[16] The structures were first optimized in the gas phase. The transition states were characterized by one negative vibrational frequency and QRC[17] (quick reaction coordinate) calculations. The solvent energies for the optimized structures were then calculated using the Poisson-Boltzmann solver with dichloromethane as the solvent. The energy that is depicted with the XYZ coordinates (listed below) was obtained from the optimized gas phase calculation. All computations were carried out using the computational cluster resources at the National Supercomputer Centre based at Linkoping University, Sweden and at the Center for High Performance Computing, South Africa.

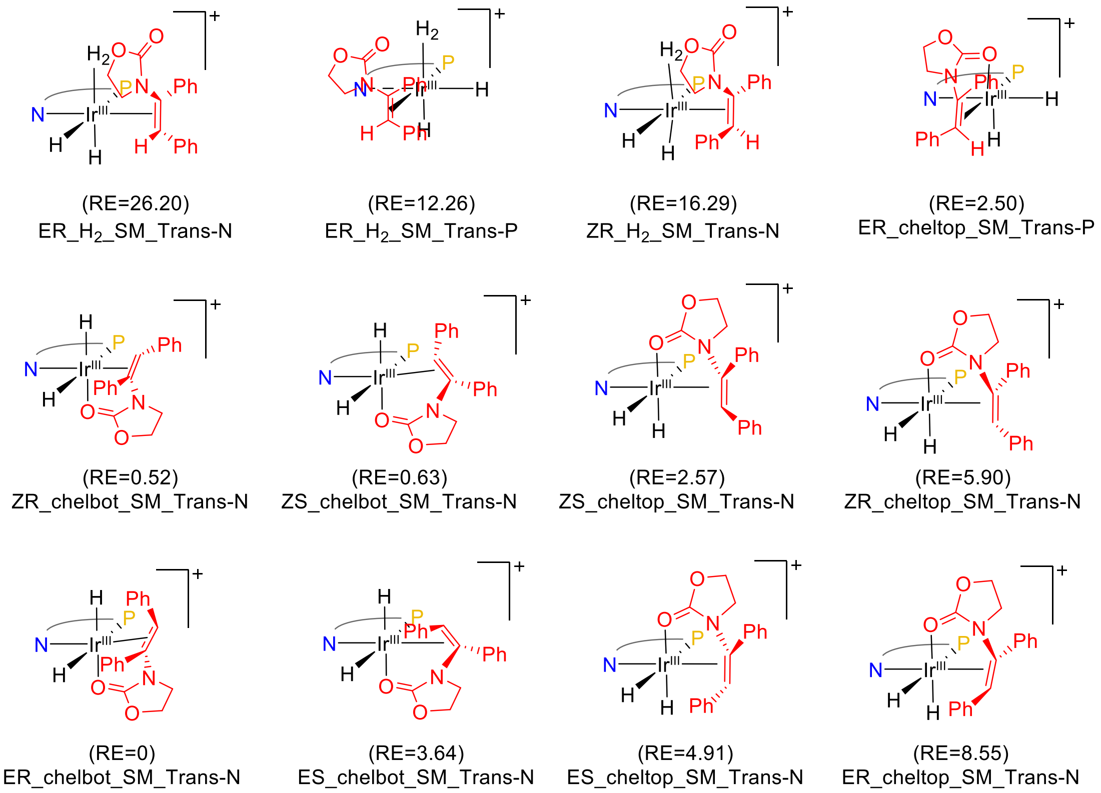

Figure S6 The substrate-catalyst complexes explored in this calculation. The P, N ligand is abbreviated as P and N; the relative energies are shown in parentheses.

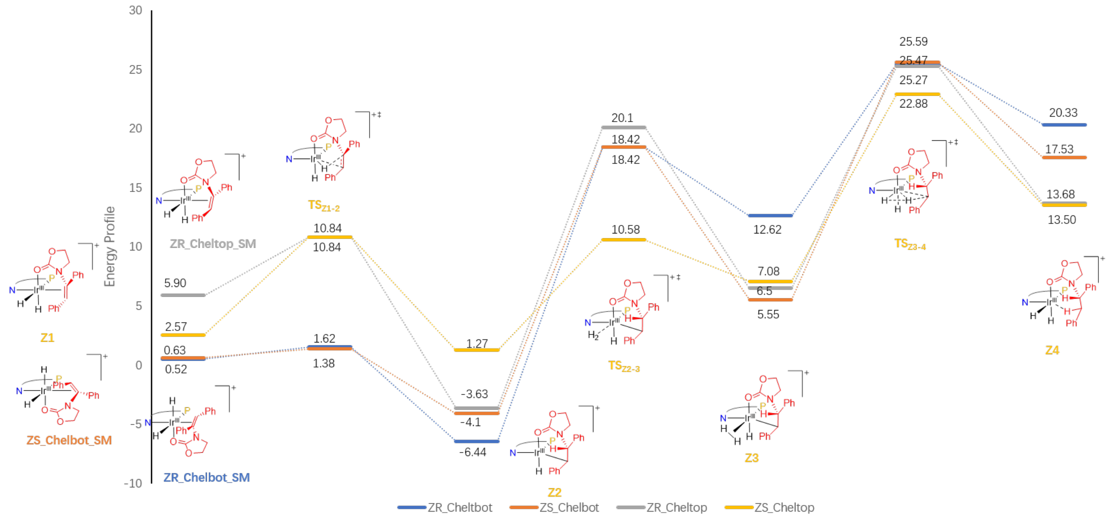

Figure S7 Free energy profile of hydrogenation for the *Z* isomer. ZS_cheltop is the most favored pathway.

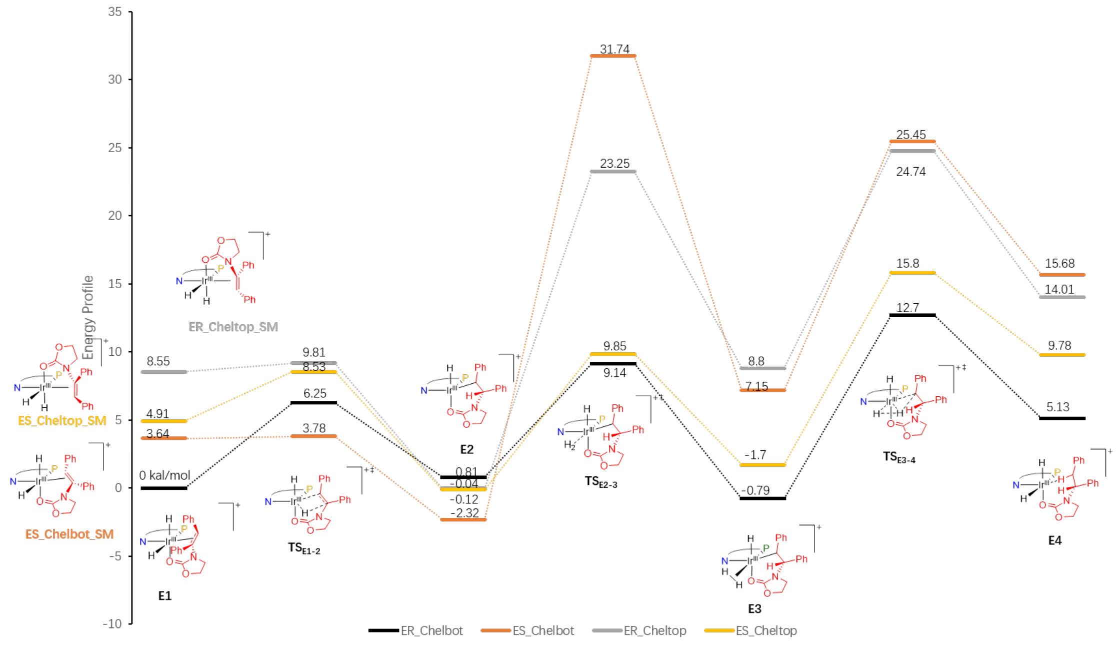

Figure S8 Free energy profile of hydrogenation for the *E* isomer. ER_Chelbot is the most favored pathway.

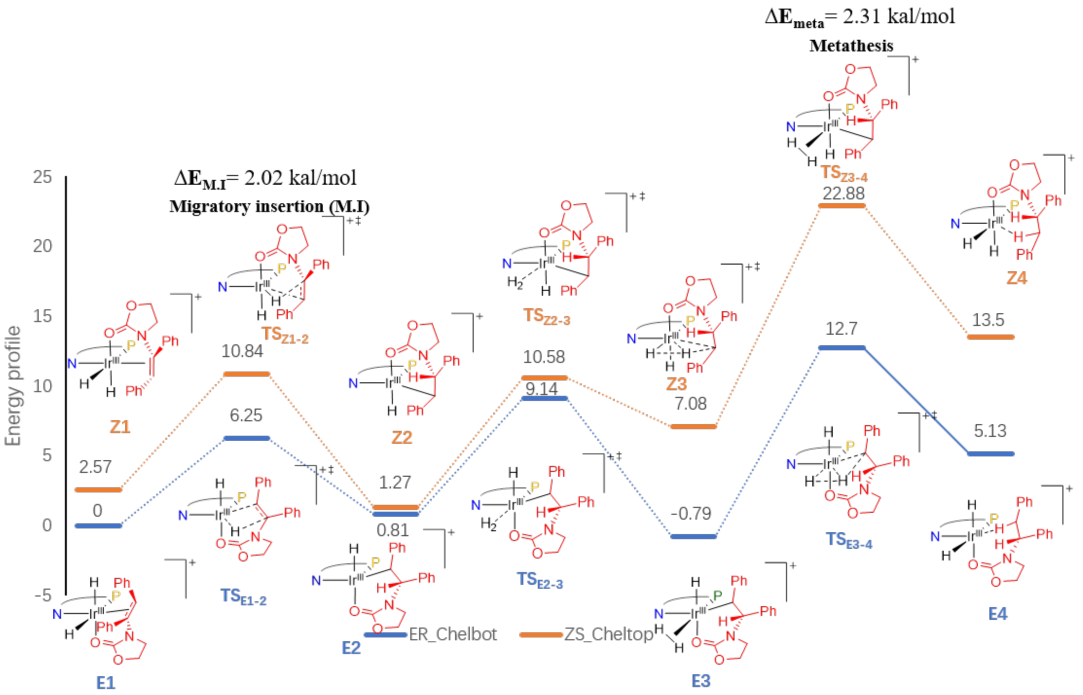

Figure S9 Energy comparison of the favored pathway for the hydrogenation of *Z*-isomer and *E*-isomer.

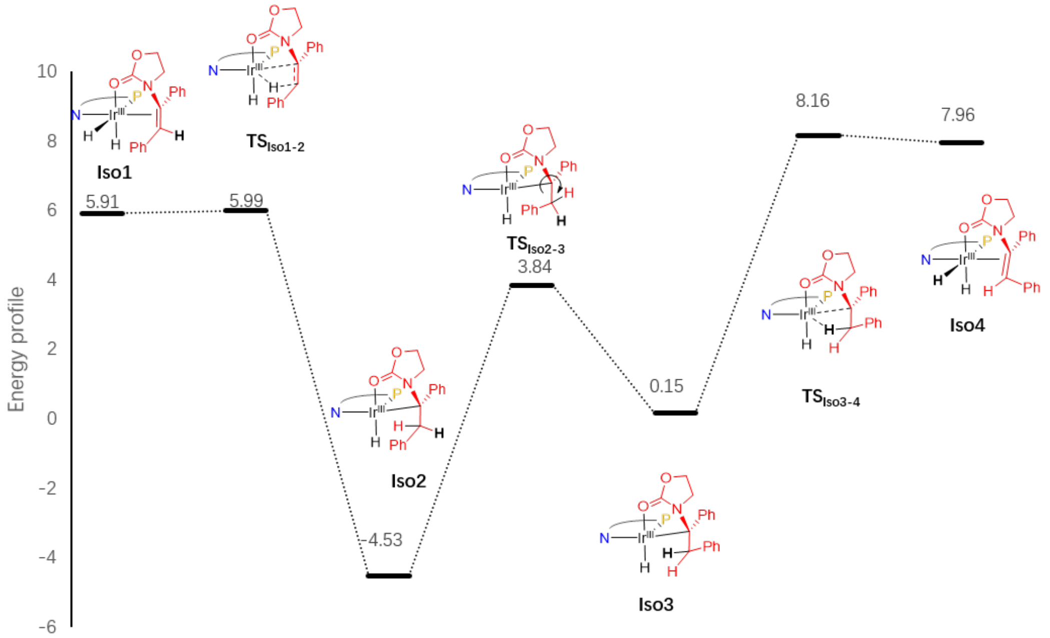

Figure S10 Free energy profile of *Z*-*E* isomerization.

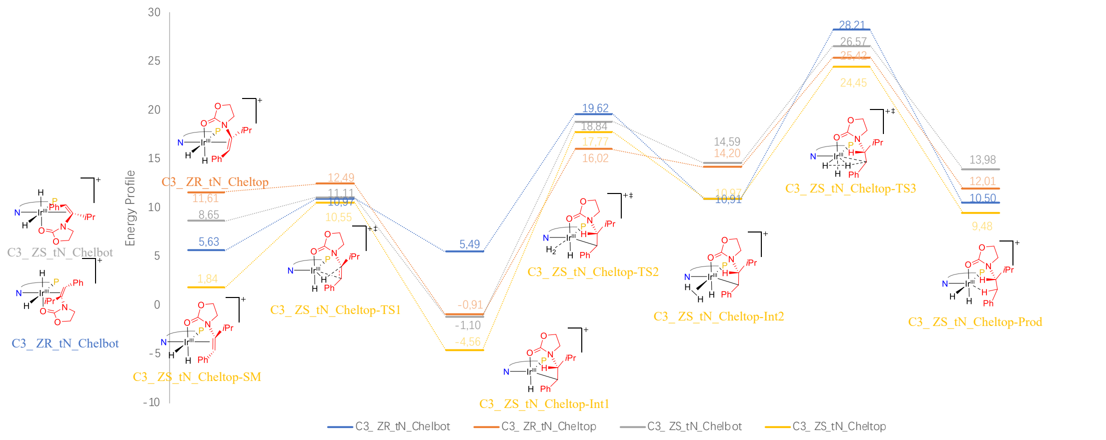

Figure S11 Free energy profile of hydrogenation for the *Z* isomer (class 3). ZS_Cheltop is the most favored pathway.

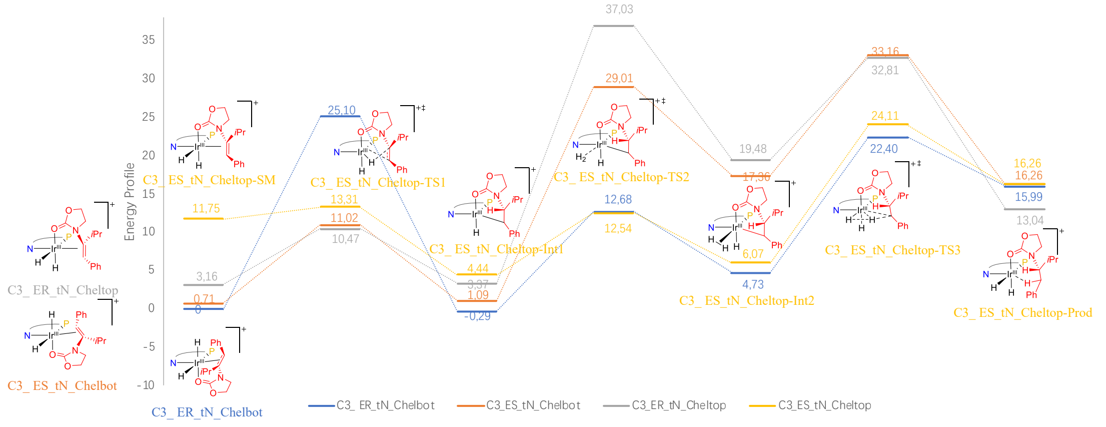

Figure S12 Free energy profile of hydrogenation for the *E* isomer (class 3). ES_Cheltop is the most favored pathway.

10. NMR spectra of new compounds

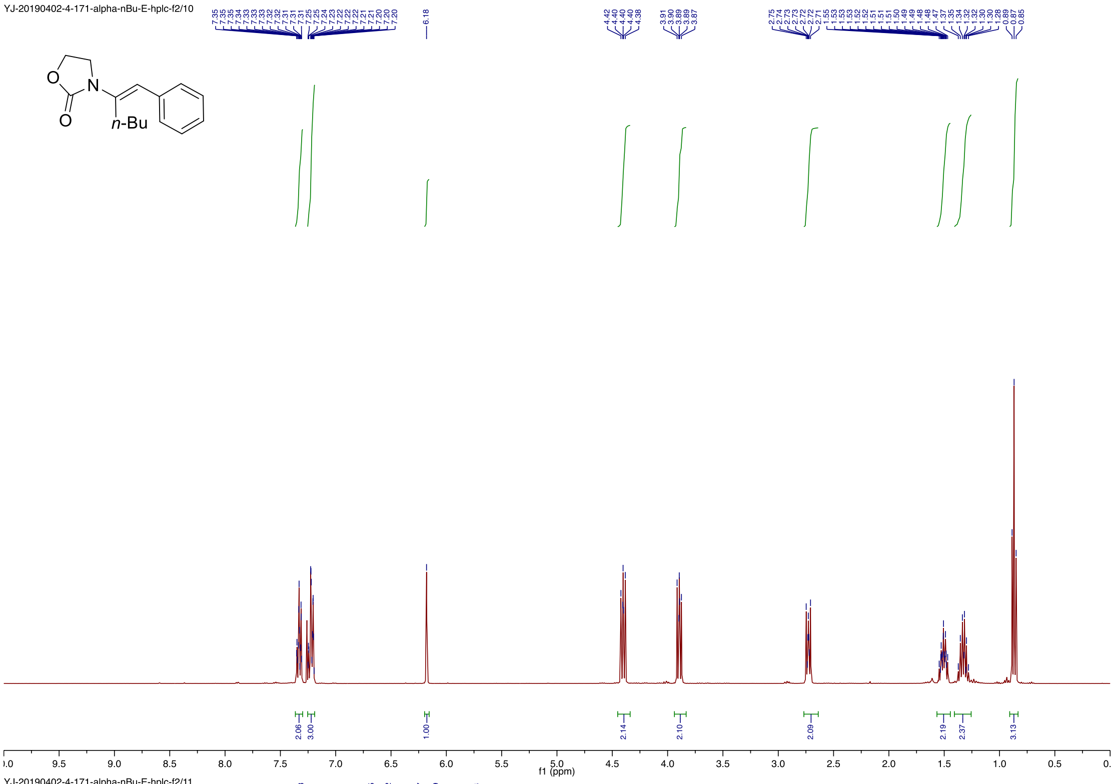

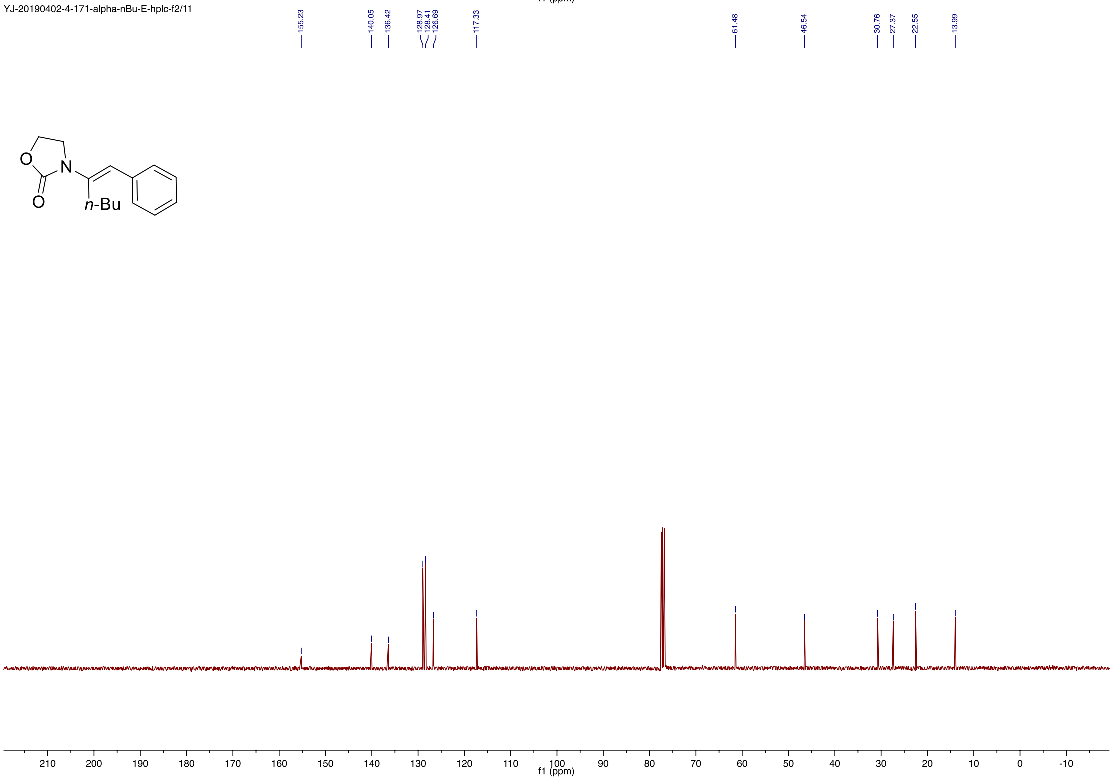

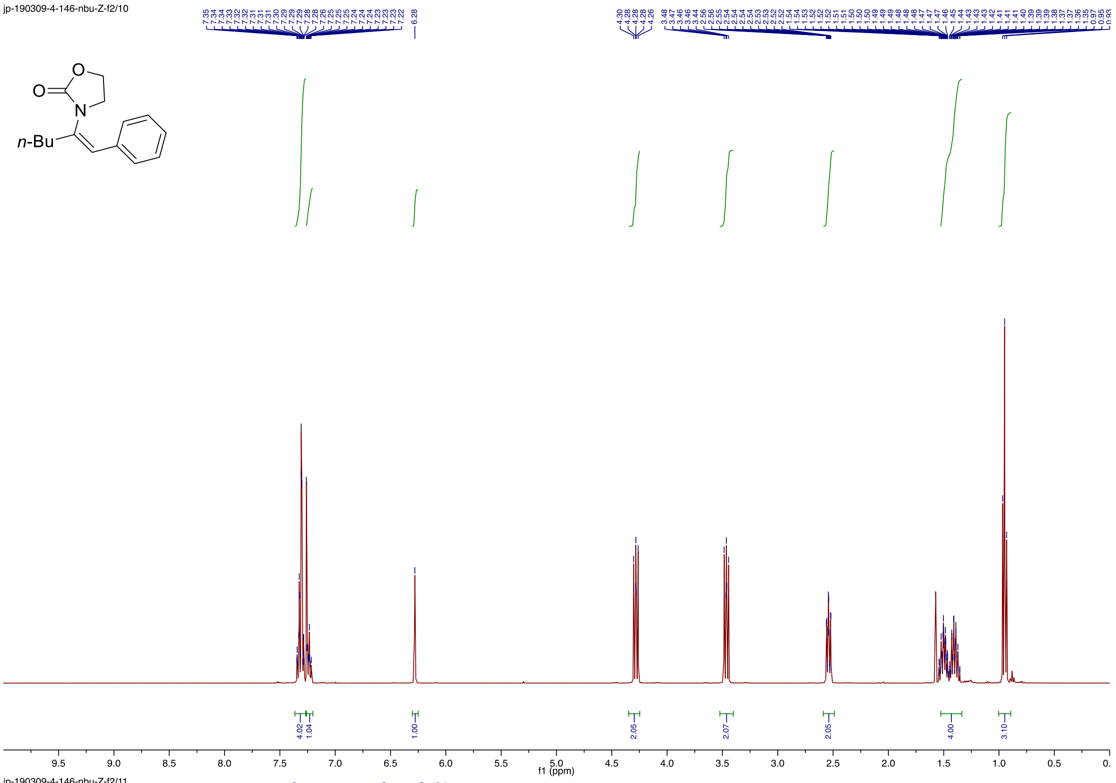
jp-190309-4-146-nbu-Z-f2/10
O
O
N
n-Bu
4.02
1.04
1.00
2.05
2.07
2.05
4.00
3.10
f1 (ppm)

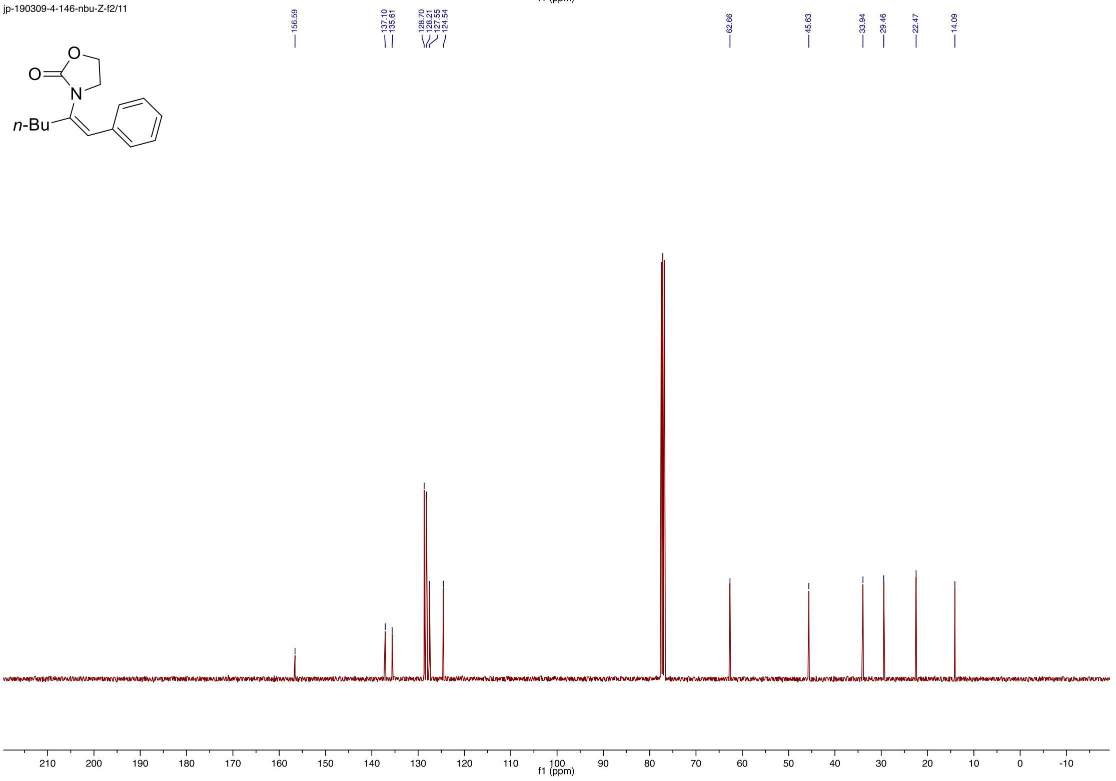
jp-190309-4-146-nbu-Z-f2/11
156.59
137.10
135.61
128.70
128.21
127.55
124.54
62.66
45.63
33.94
29.46
22.47
14.09
O
O
N
n-Bu
f1 (ppm)

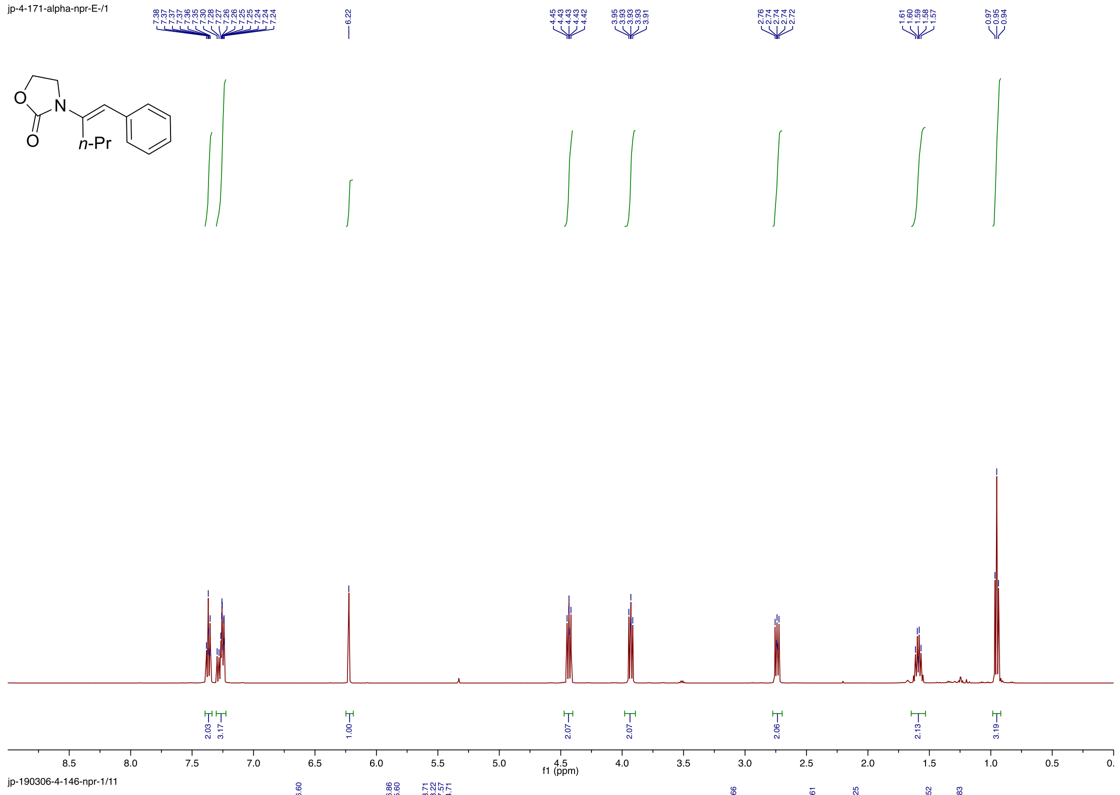
jp-4-171-alpha-npr-E-/1
n-Pr
f1 (ppm)

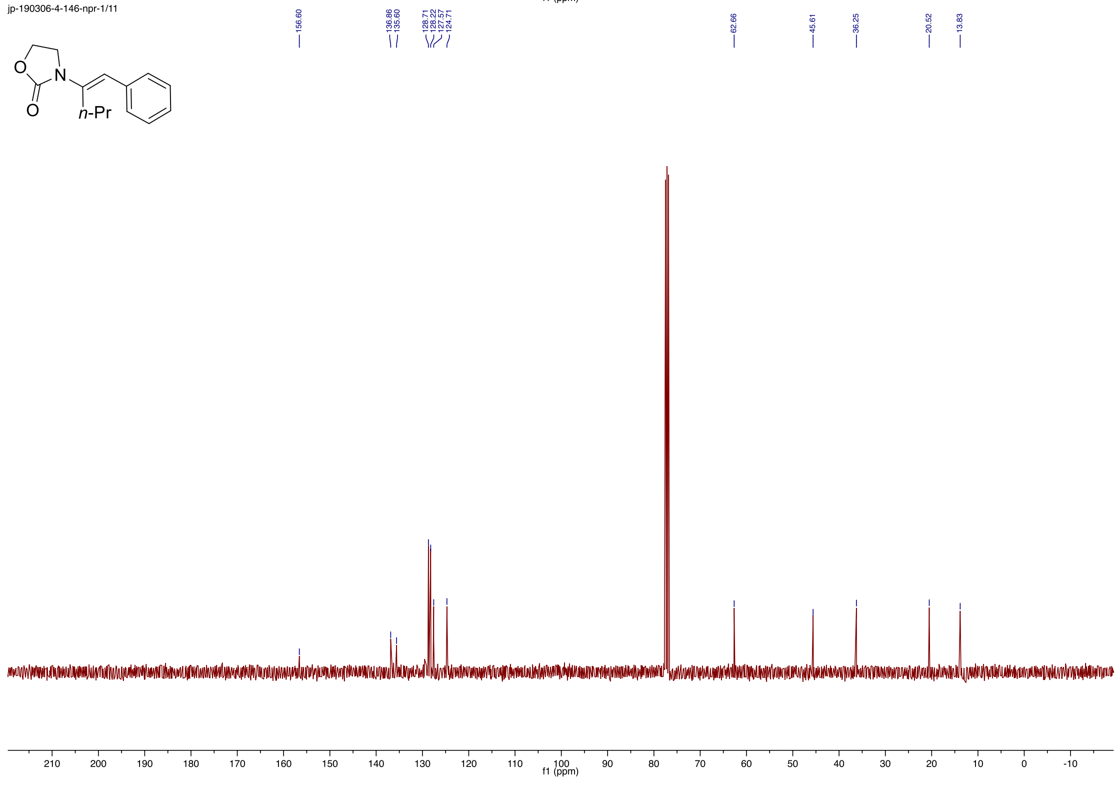
jp-190306-4-146-npr-1/11
n-Pr
f1 (ppm)

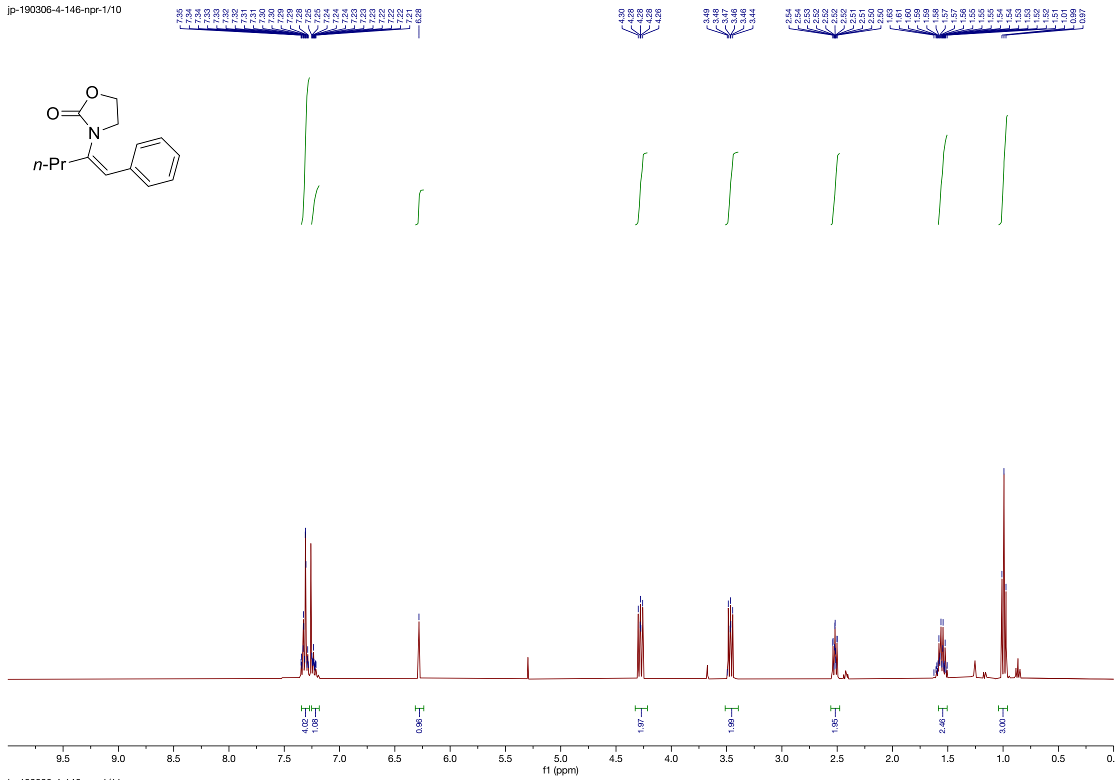
jp-190306-4-146-npr-1/10
n-Pr
f1 (ppm)

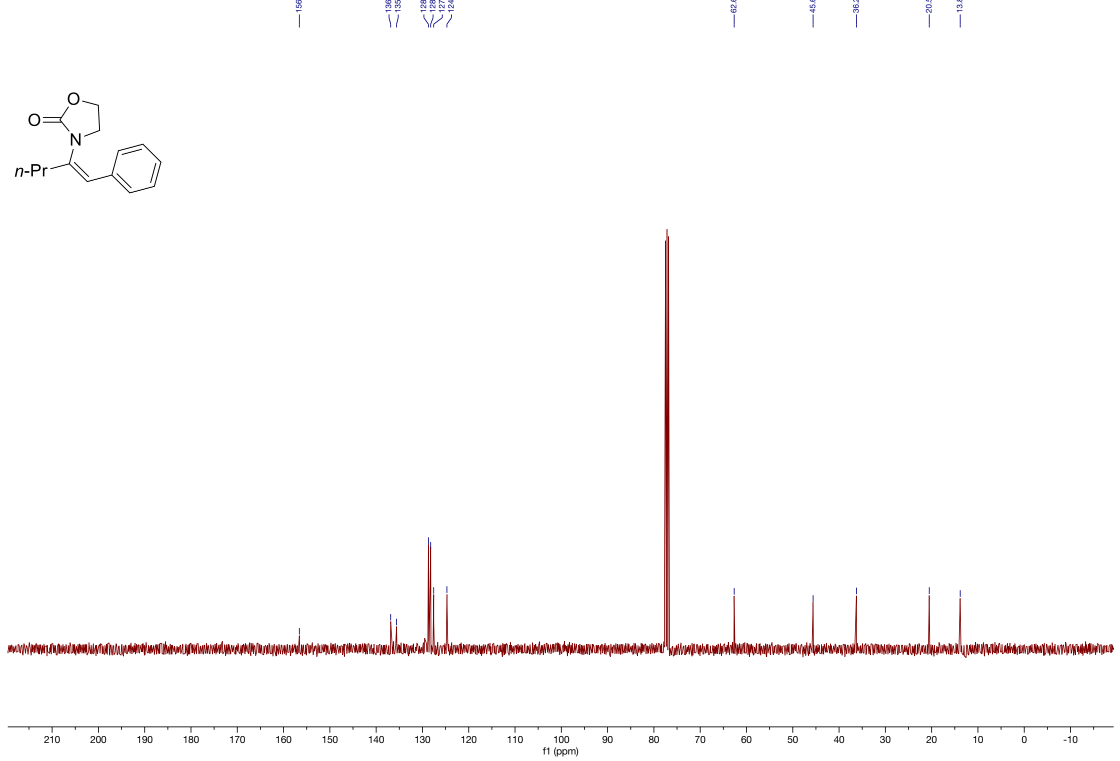
jp-190306-4-146-npr-1/11
156.60
136.86
135.60
128.71
128.22
127.57
124.71
62.66
45.61
36.25
20.52
13.83
n-Pr
f1 (ppm)

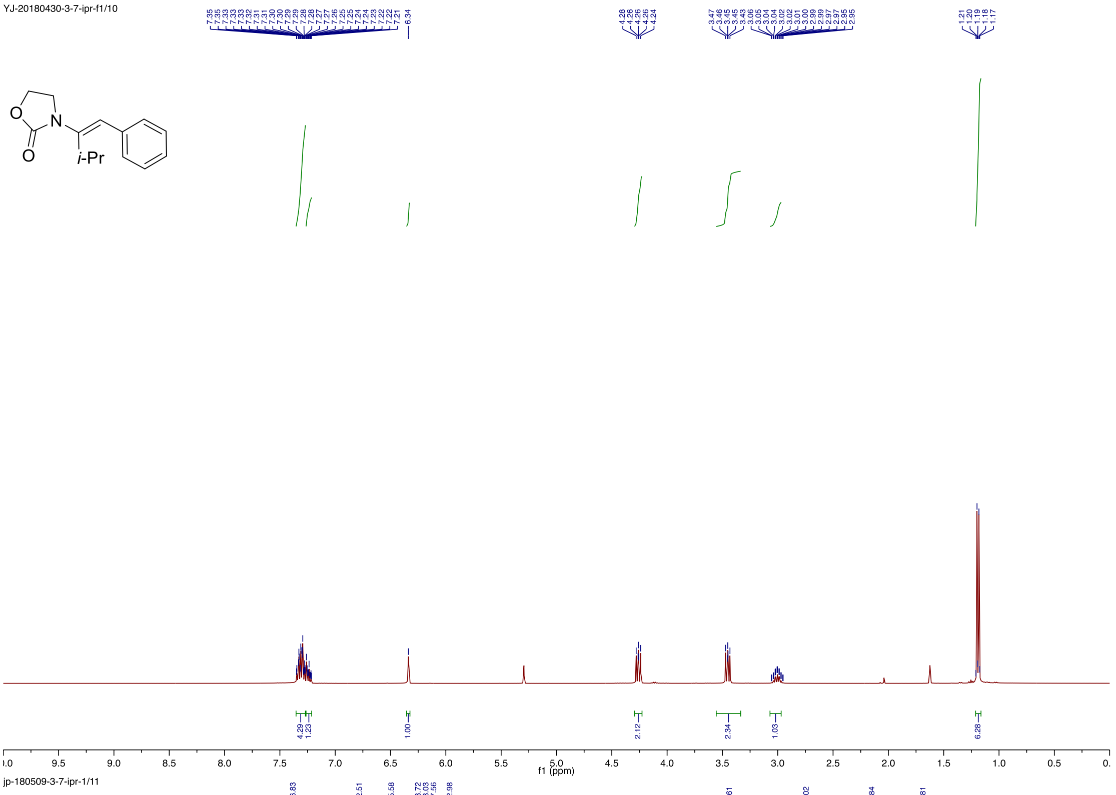
YJ-20180430-3-7-ipr-f1/10
7.35 7.35 7.33 7.33 7.32 7.31 7.31 7.30 7.30 7.29 7.29 7.28 7.28 7.27 7.27 7.26 7.25 7.24 7.24 7.23 7.22 7.22 7.21 6.34
4.28 4.26 4.26 4.26 4.24
3.47 3.46 3.45 3.45 3.43 3.06 3.05 3.04 3.04 3.02 3.02 3.01 3.00 2.99 2.99 2.97 2.97 2.95 2.95
1.21 1.20 1.19 1.18 1.17
O
N
O
i-Pr
4.29
1.23
1.00
2.12
2.34
1.03
6.28
f1 (ppm)

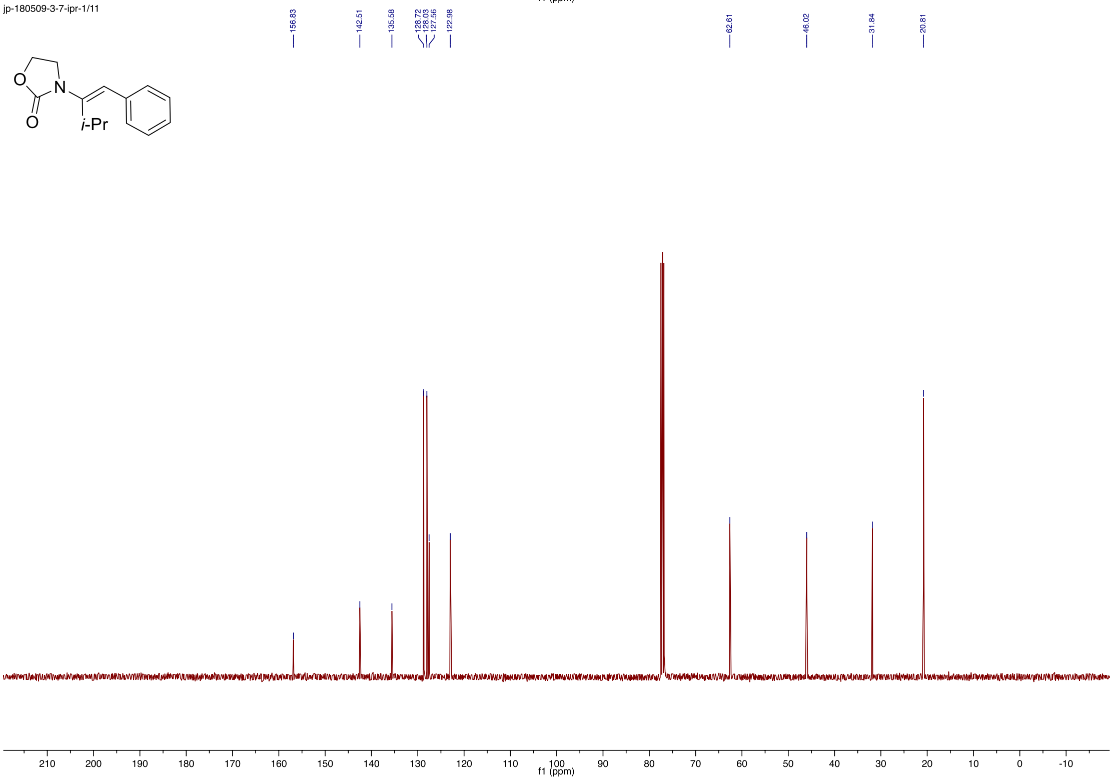
jp-180509-3-7-ipr-1/11
156.83
142.51
135.58
128.72 128.03 127.56
122.98
62.61
46.02
31.84
20.81
O
N
O
i-Pr
f1 (ppm)

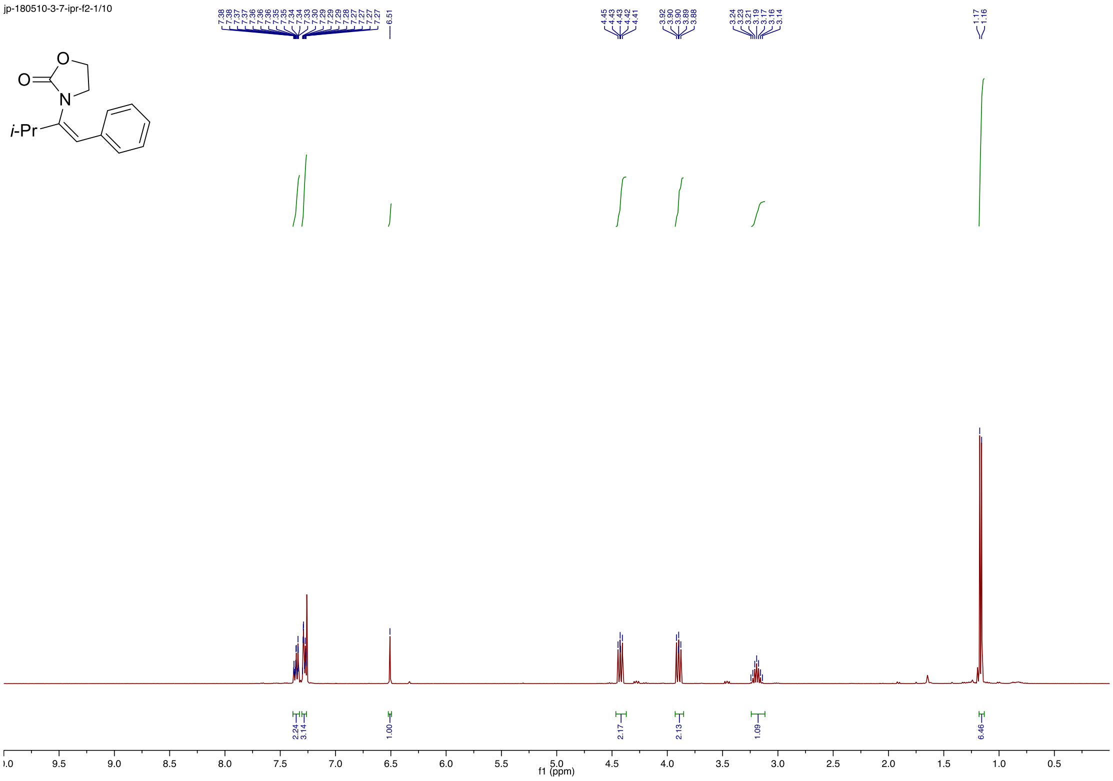
jp-180510-3-7-ipr-f2-1/10
O
N
O
i-Pr
f1 (ppm)

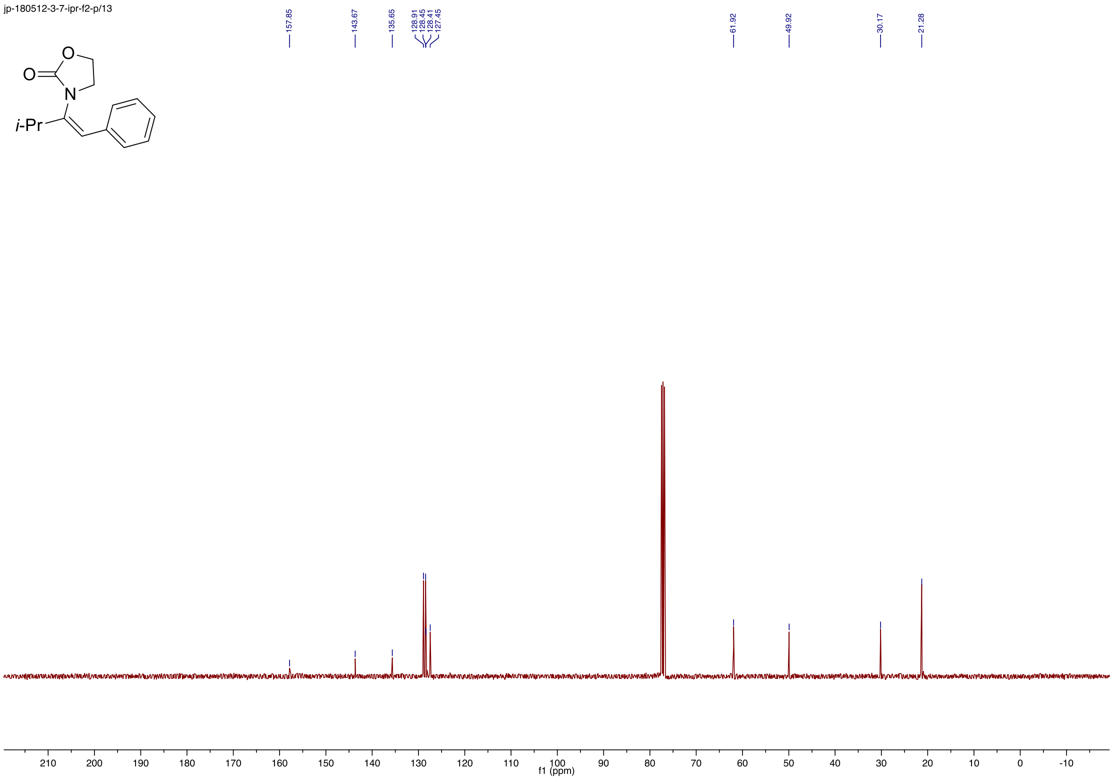
jp-180512-3-7-ipr-f2-p/13
O
N
O
i-Pr
f1 (ppm)

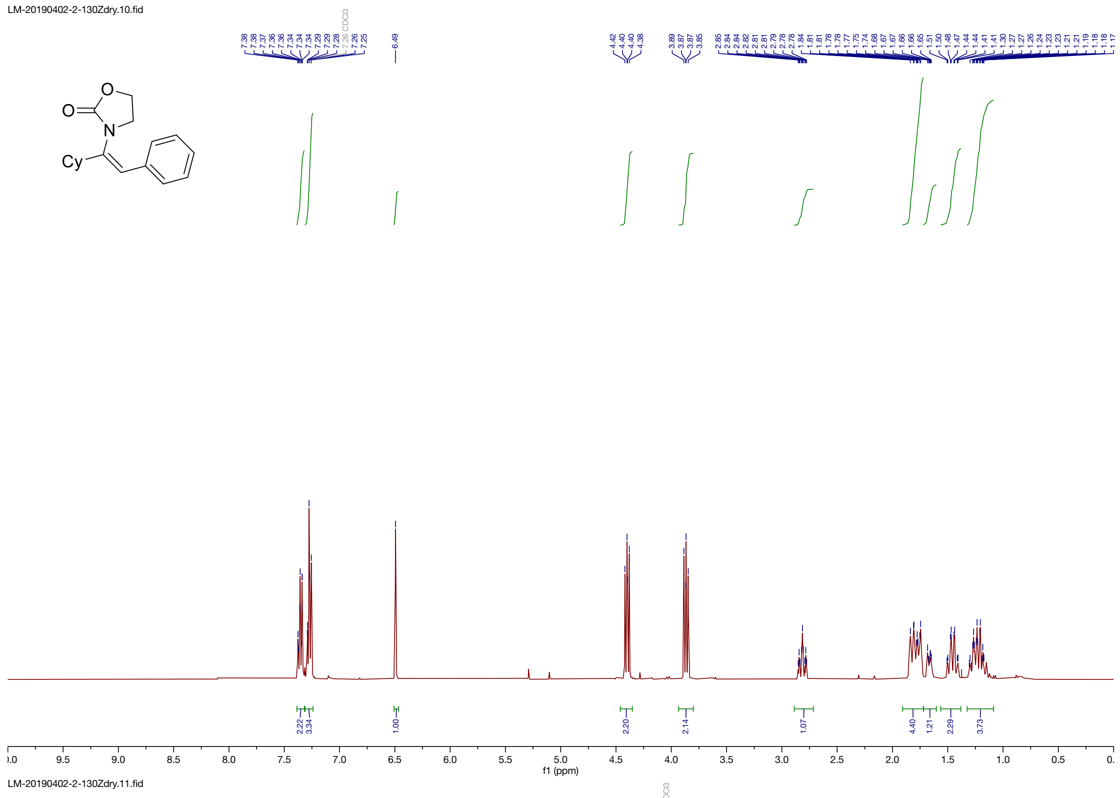
LM-20190402-2-130Zdry.10.fid
Cy
f1 (ppm)

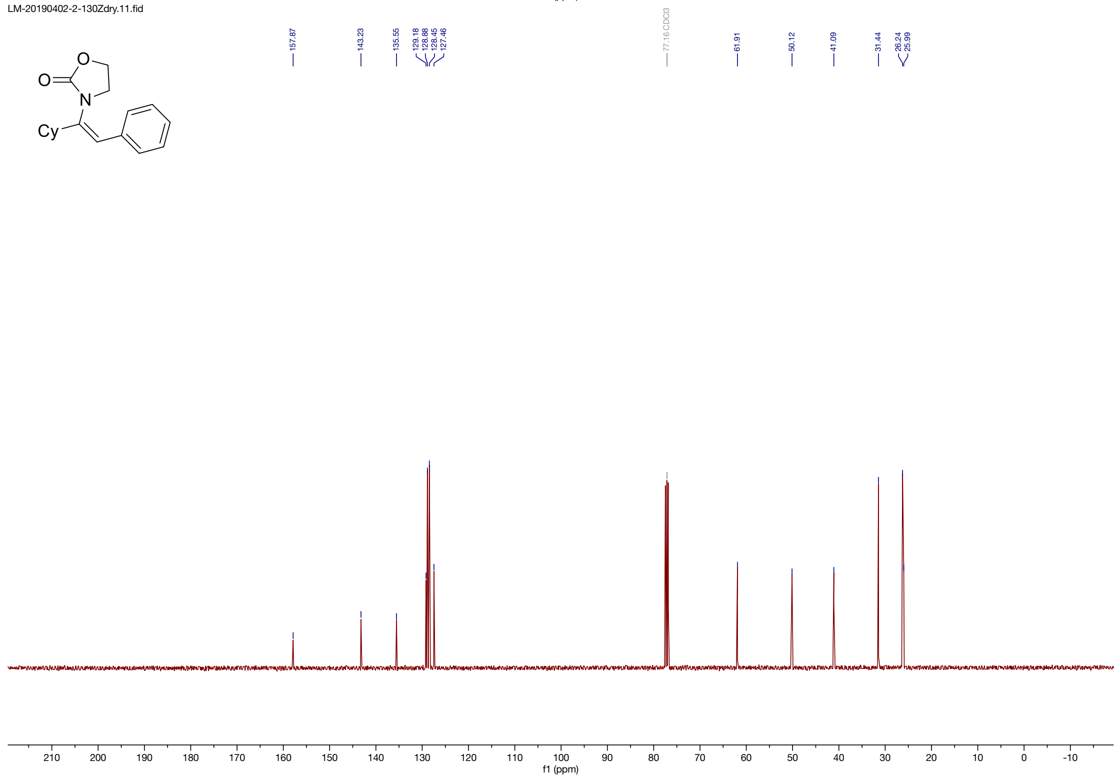
LM-20190402-2-130Zdry.11.fid
Cy
157.87
143.23
135.55
129.18
128.88
128.45
127.46
77.16 CDCl3
61.91
50.12
41.09
31.44
26.24
25.99
f1 (ppm)

LM-20190219-2-65cy.10.fid

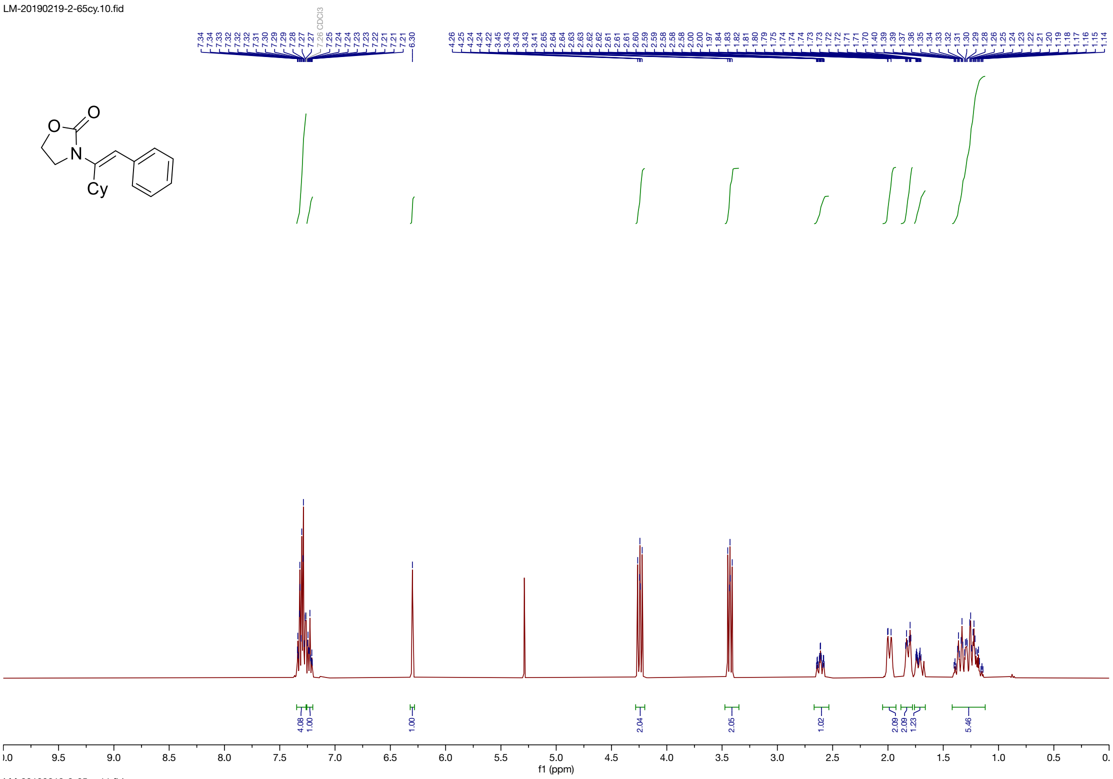

LM-20190219-2-65cy.11.fid

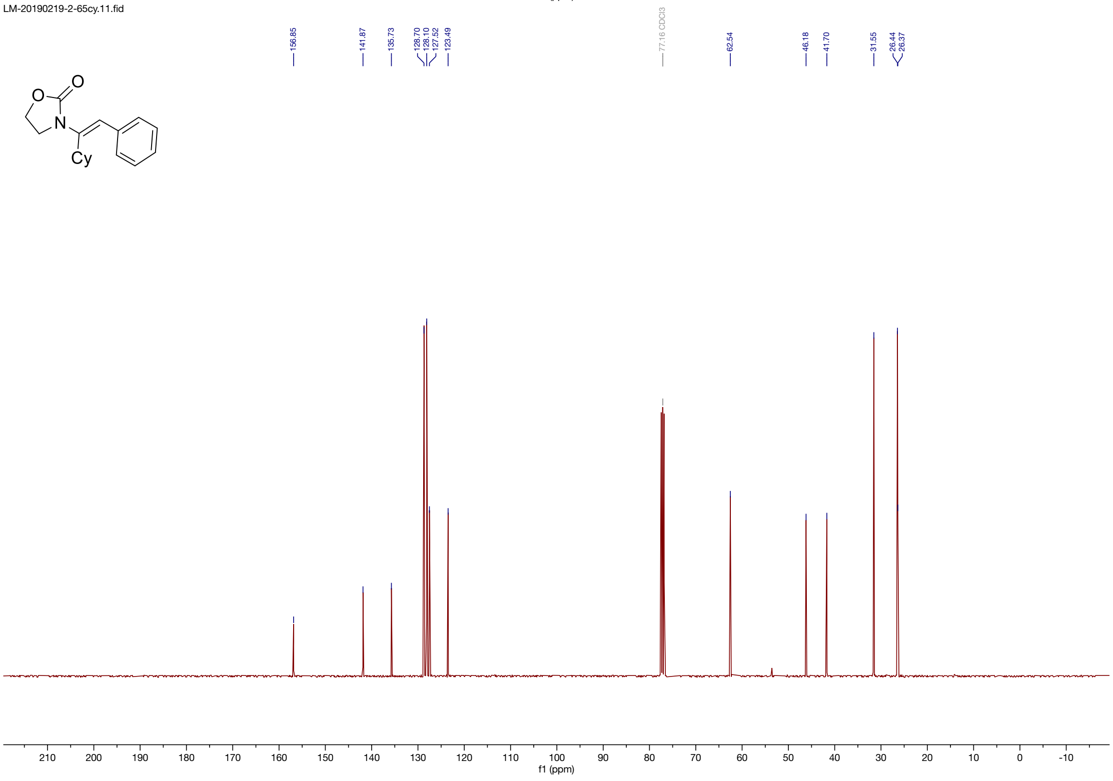

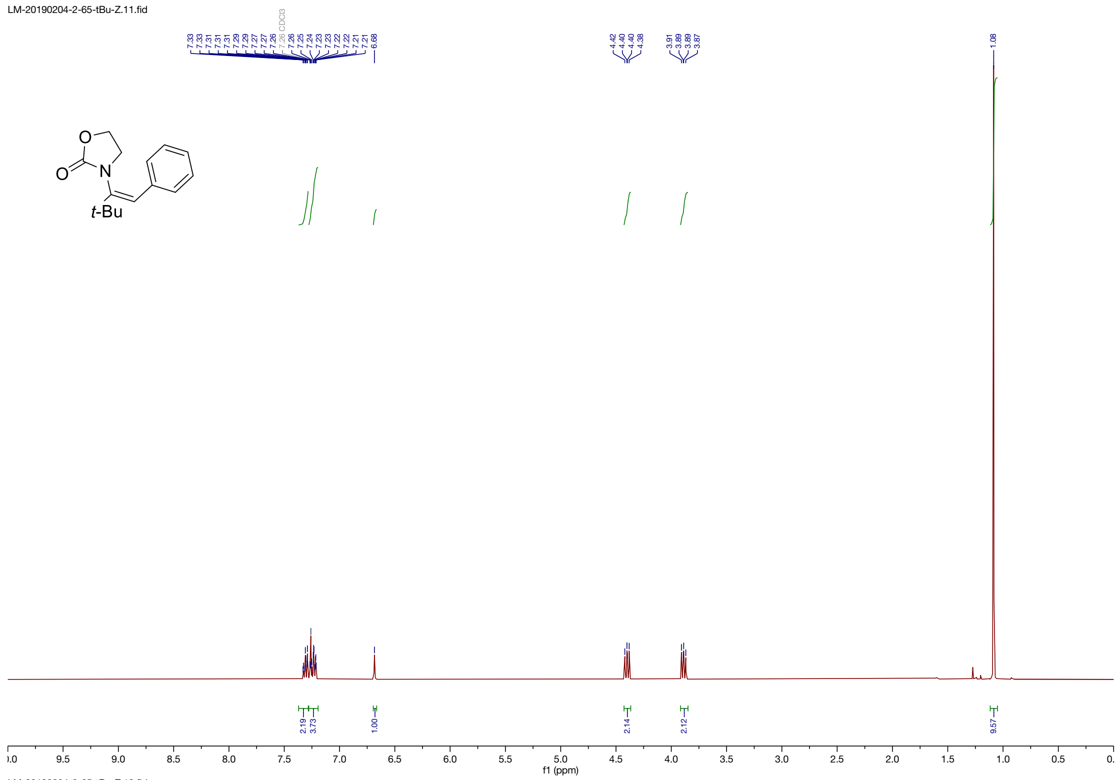

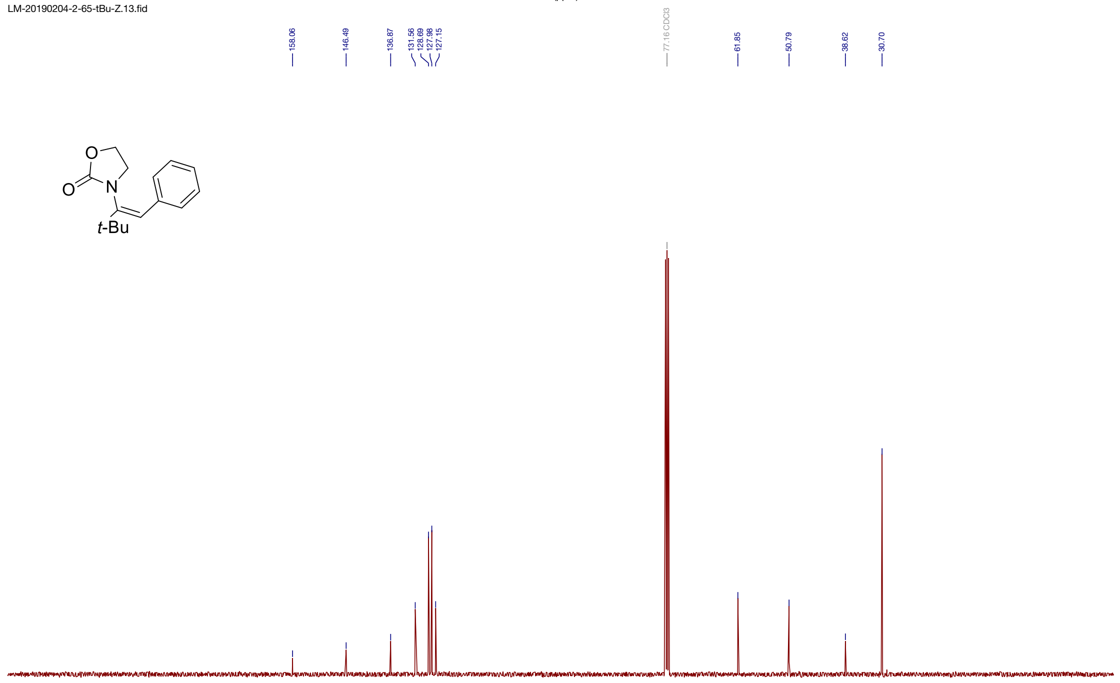

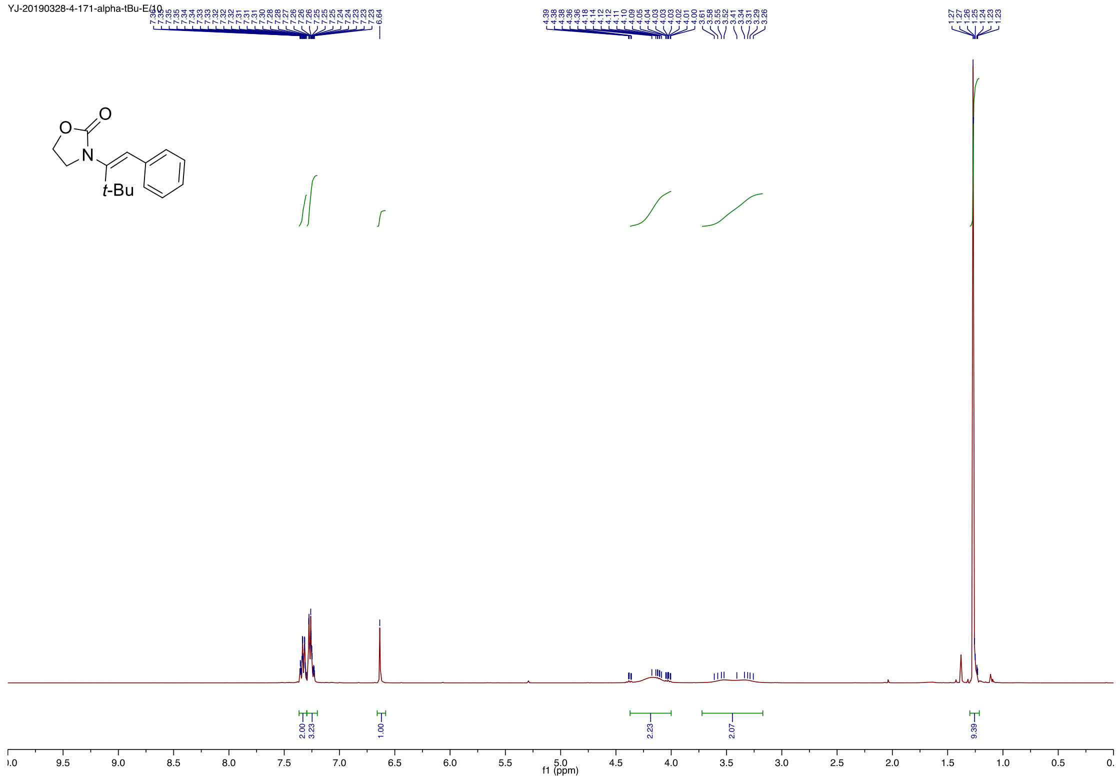
YJ-20190328-4-171-alpha-tBu-E/10
t-Bu
2.00
3.23
1.00
2.23
2.07
9.39
f1 (ppm)

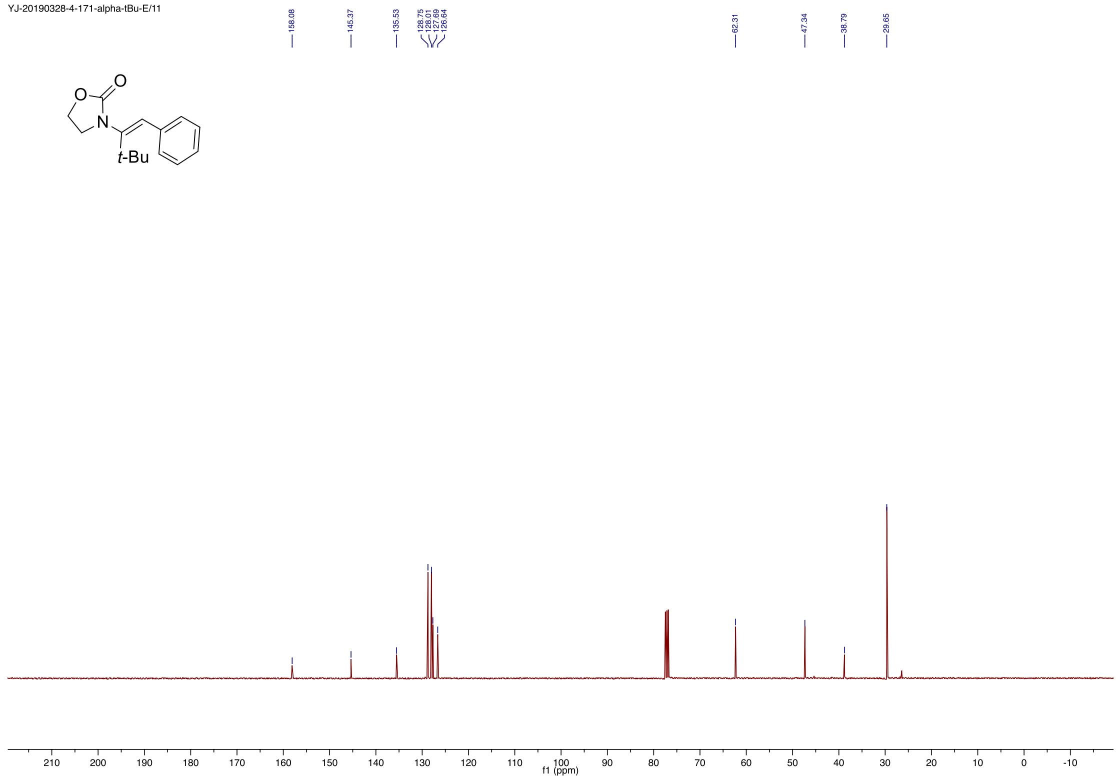
YJ-20190328-4-171-alpha-tBu-E/11
158.08
145.37
135.53
128.75
128.01
127.69
126.64
62.31
47.34
38.79
29.65
t-Bu
f1 (ppm)

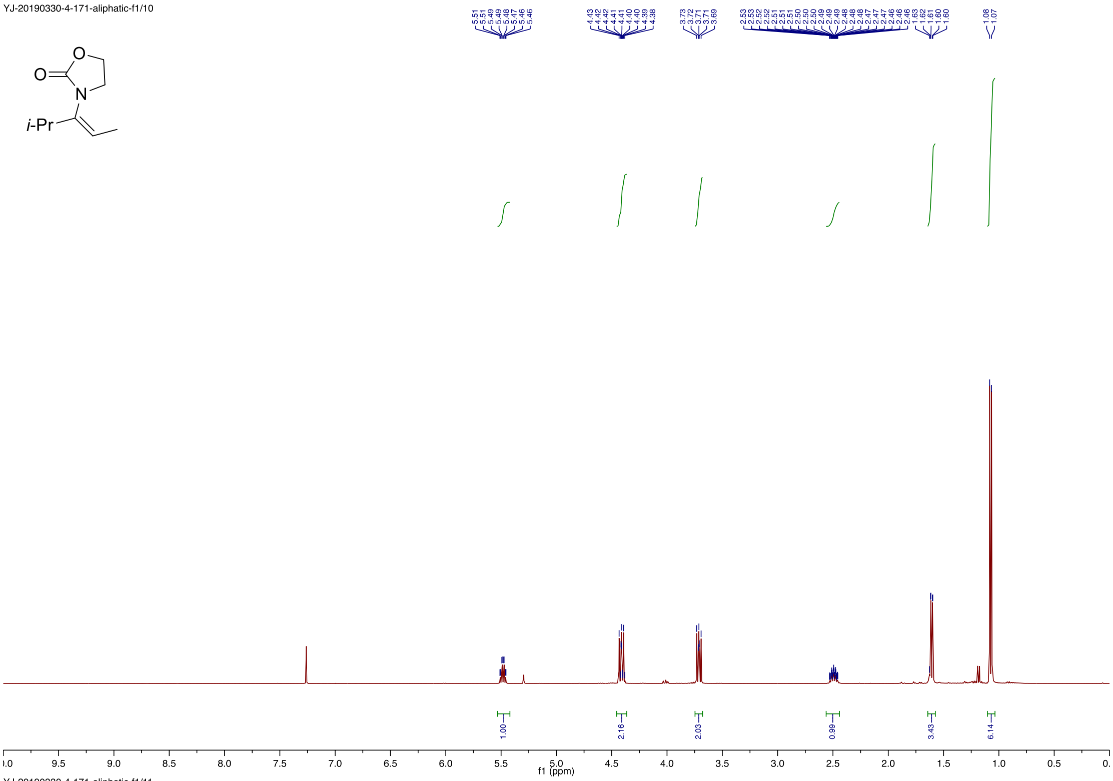
YJ-20190330-4-171-aliphatic-f1/10
O
N
O
i-Pr
5.51
5.51
5.49
5.49
5.48
5.47
5.46
5.46
4.43
4.42
4.42
4.41
4.40
4.40
4.39
4.38
3.73
3.72
3.71
3.71
3.69
1.63
1.62
1.61
1.60
1.60
1.08
1.07
1.00
2.16
2.03
0.99
3.43
6.14
f1 (ppm)

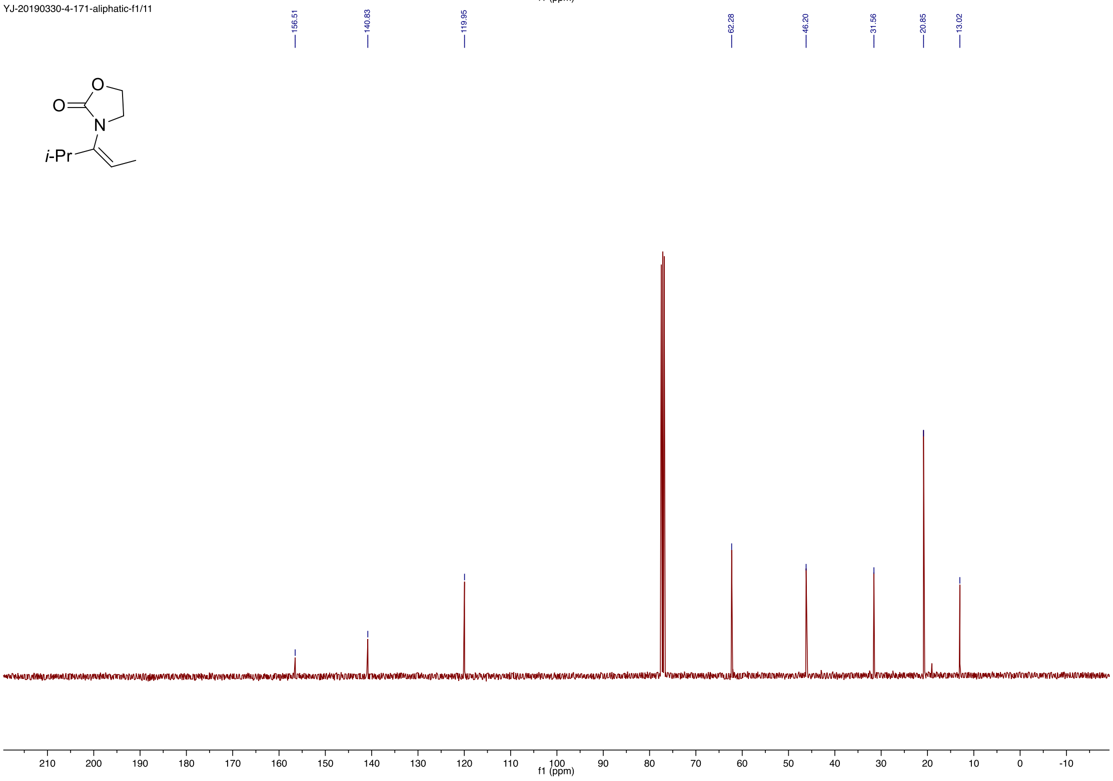
YJ-20190330-4-171-aliphatic-f1/11
O
N
O
i-Pr
156.51
140.83
119.95
62.28
46.20
31.56
20.85
13.02
f1 (ppm)

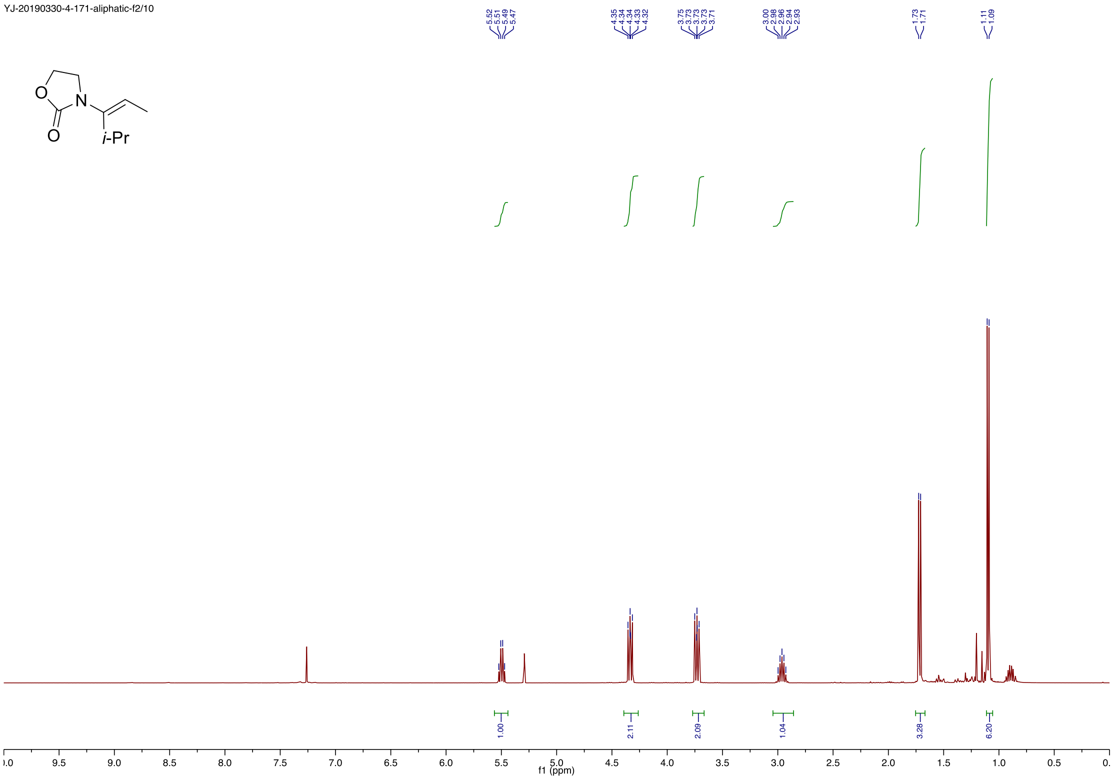
YJ-20190330-4-171-aliphatic-f2/10
O
N
O
i-Pr
5.52
5.51
5.49
5.47
4.35
4.34
4.34
4.33
4.32
3.75
3.73
3.73
3.73
3.71
3.00
2.98
2.96
2.94
2.93
1.73
1.71
1.11
1.09
1.00
2.11
2.09
1.04
3.28
6.20
f1 (ppm)

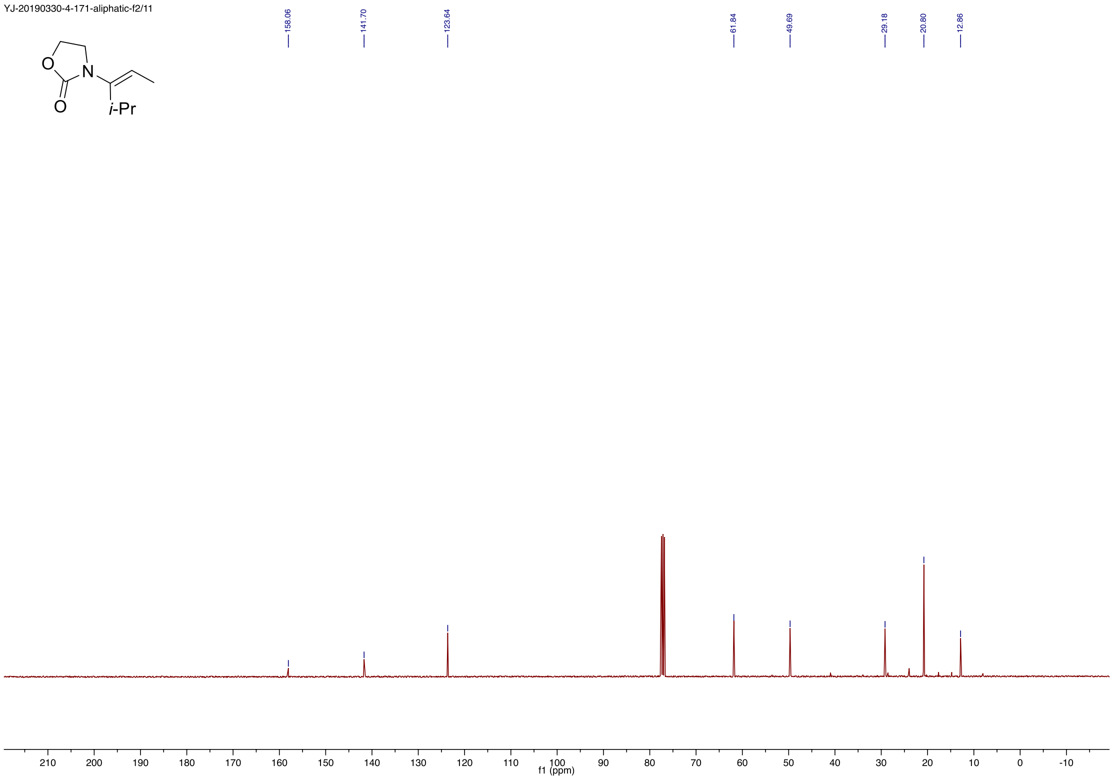
YJ-20190330-4-171-aliphatic-f2/11
O
N
O
i-Pr
158.06
141.70
123.64
61.84
49.69
29.18
20.80
12.86
f1 (ppm)

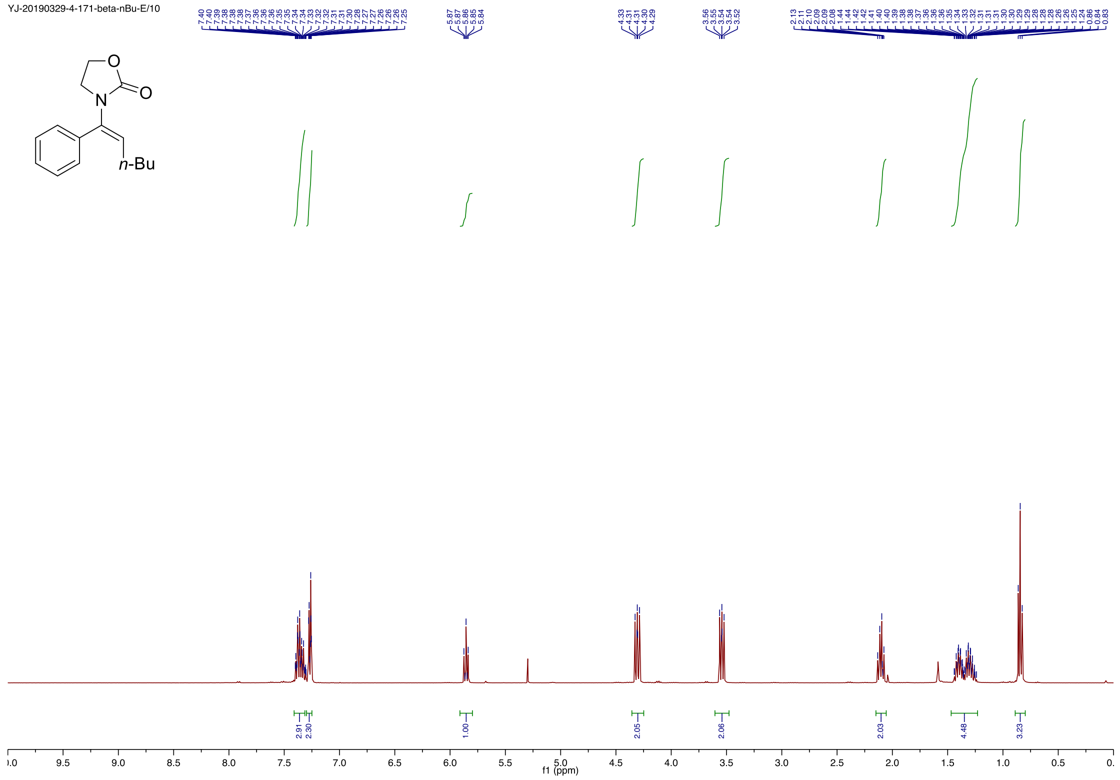
YJ-20190329-4-171-beta-nBu-E/10
n-Bu
f1 (ppm)

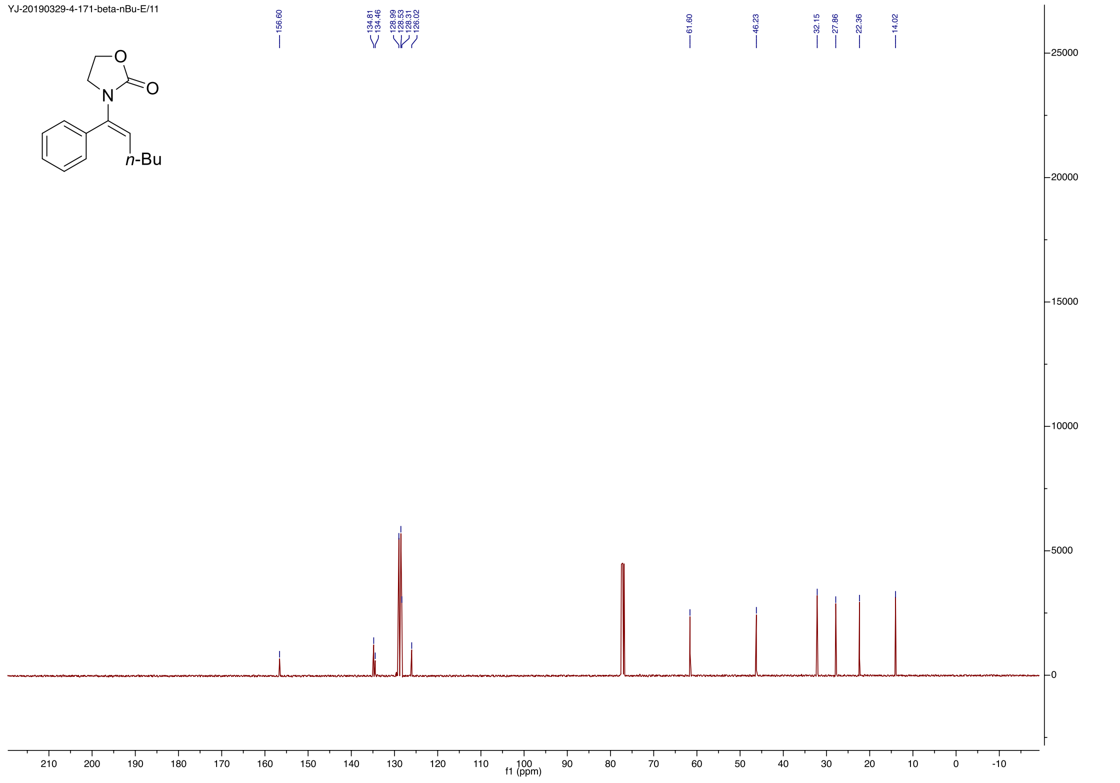
YJ-20190329-4-171-beta-nBu-E/11
n-Bu
156.60
134.81
134.46
128.99
128.53
128.31
126.02
61.60
46.23
32.15
27.86
22.36
14.02
f1 (ppm)

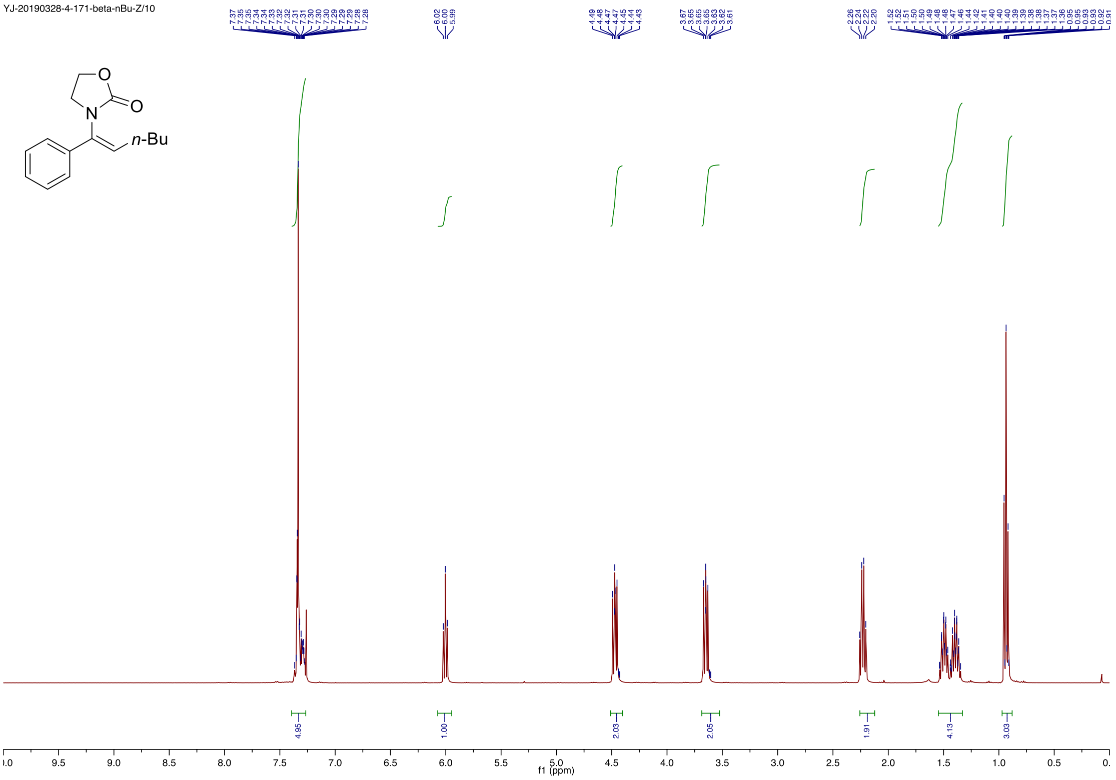
YJ-20190328-4-171-beta-nBu-Z/10
O
N
O
n-Bu
4.95
1.00
2.03
2.05
1.91
4.13
3.03
f1 (ppm)

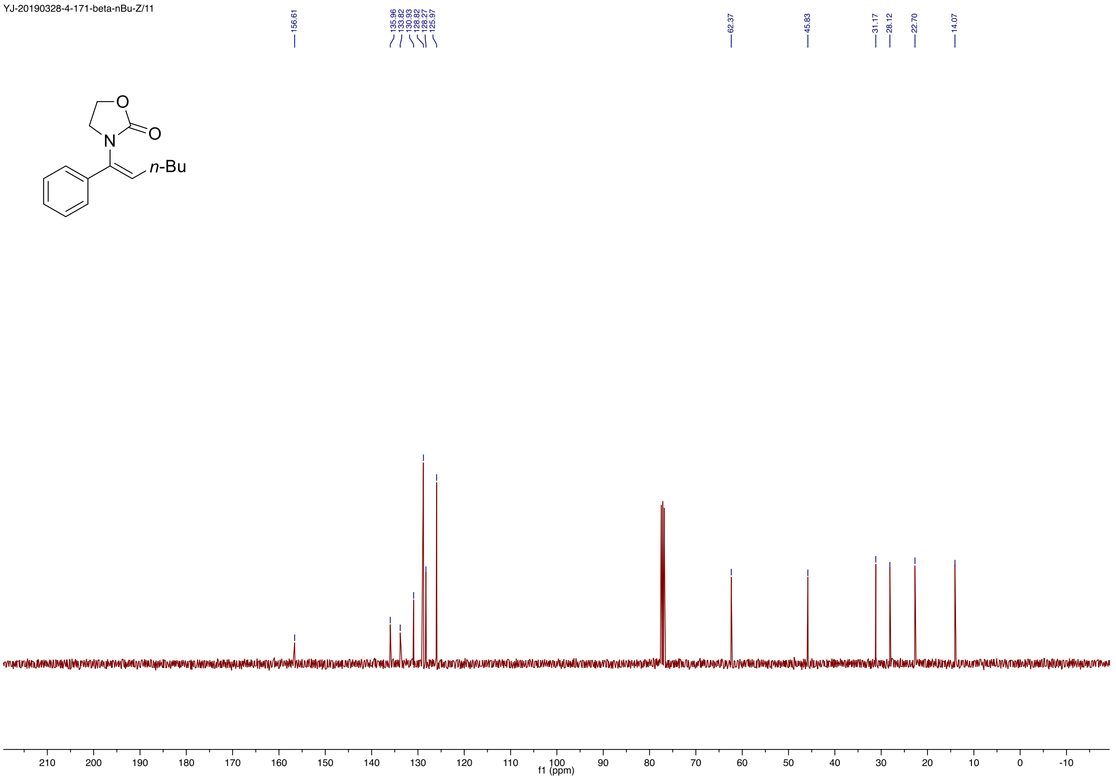
YJ-20190328-4-171-beta-nBu-Z/11
156.61
135.96
133.82
130.93
128.82
128.27
125.97
62.37
45.83
31.17
28.12
22.70
14.07
O
N
O
n-Bu
f1 (ppm)

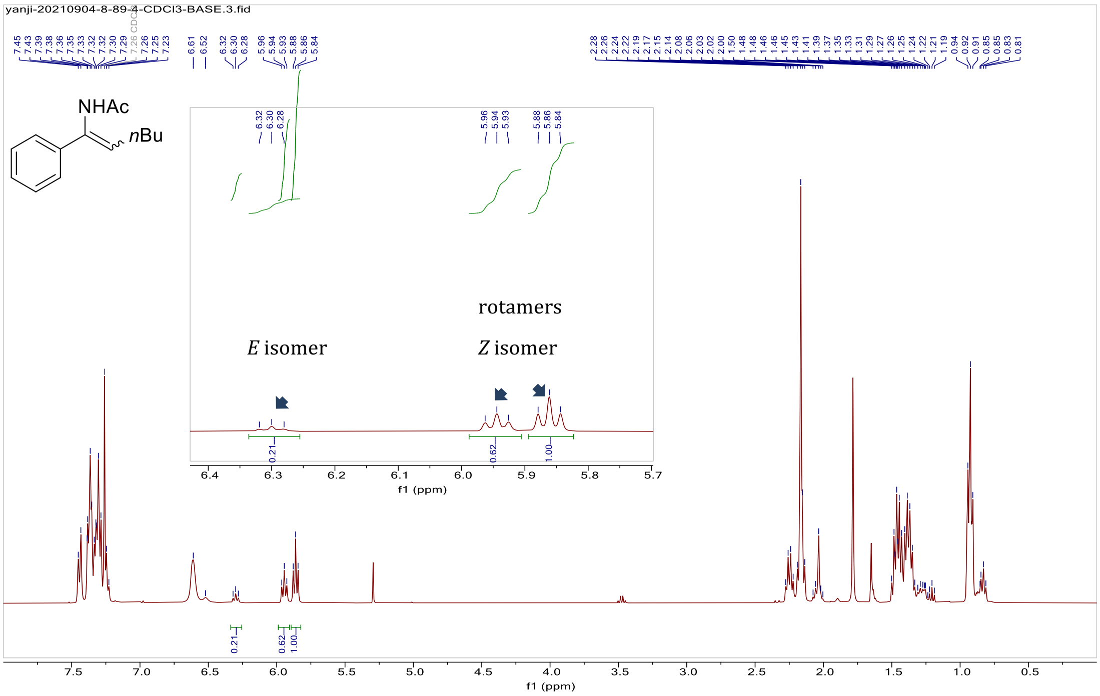
yanji-20210904-8-89-4-CDCl3-BASE.3.fid
NHAc
nBu
E isomer
rotamers
Z isomer
f1 (ppm)

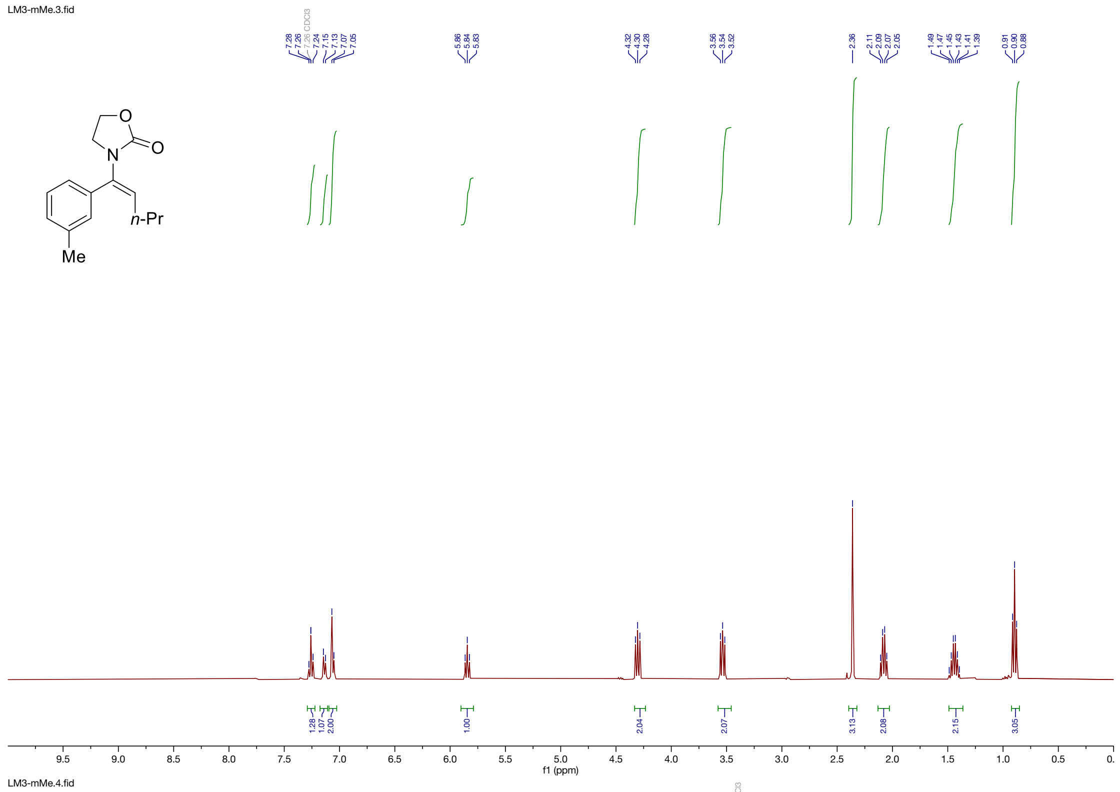
LM3-mMe.3.fid
Me
n-Pr
f1 (ppm)

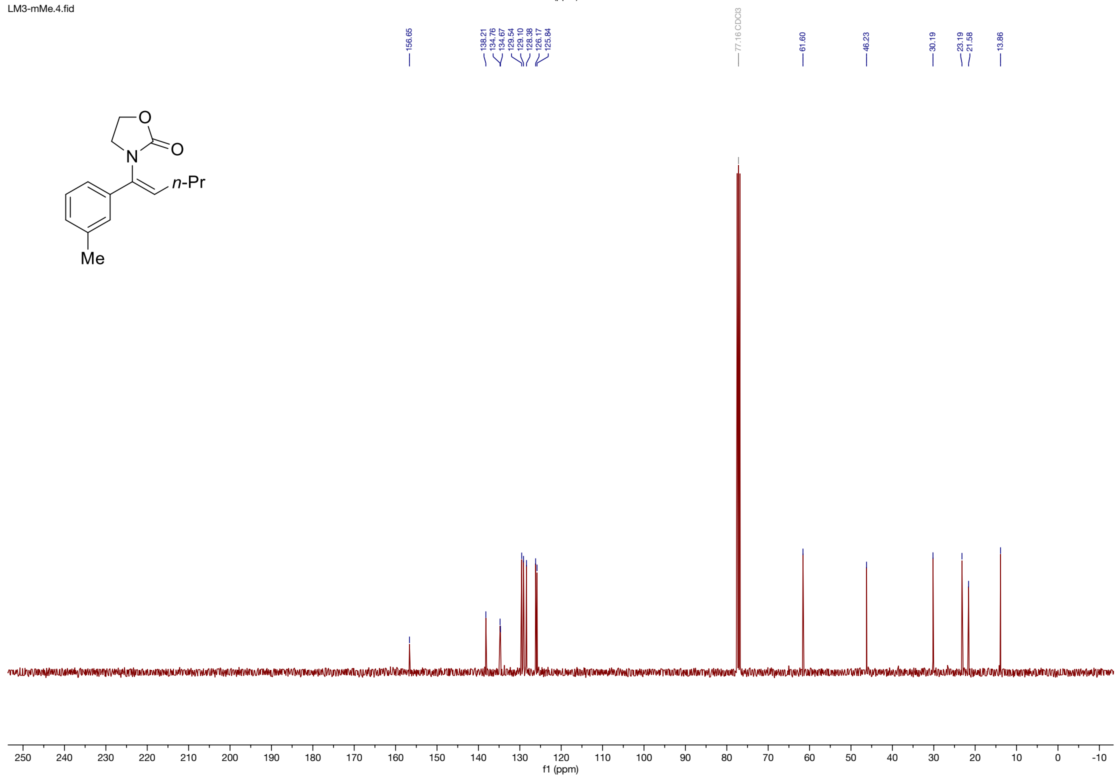
LM3-mMe.4.fid
Me
n-Pr
f1 (ppm)

LM-20190907-3-47-nPe.10.fid

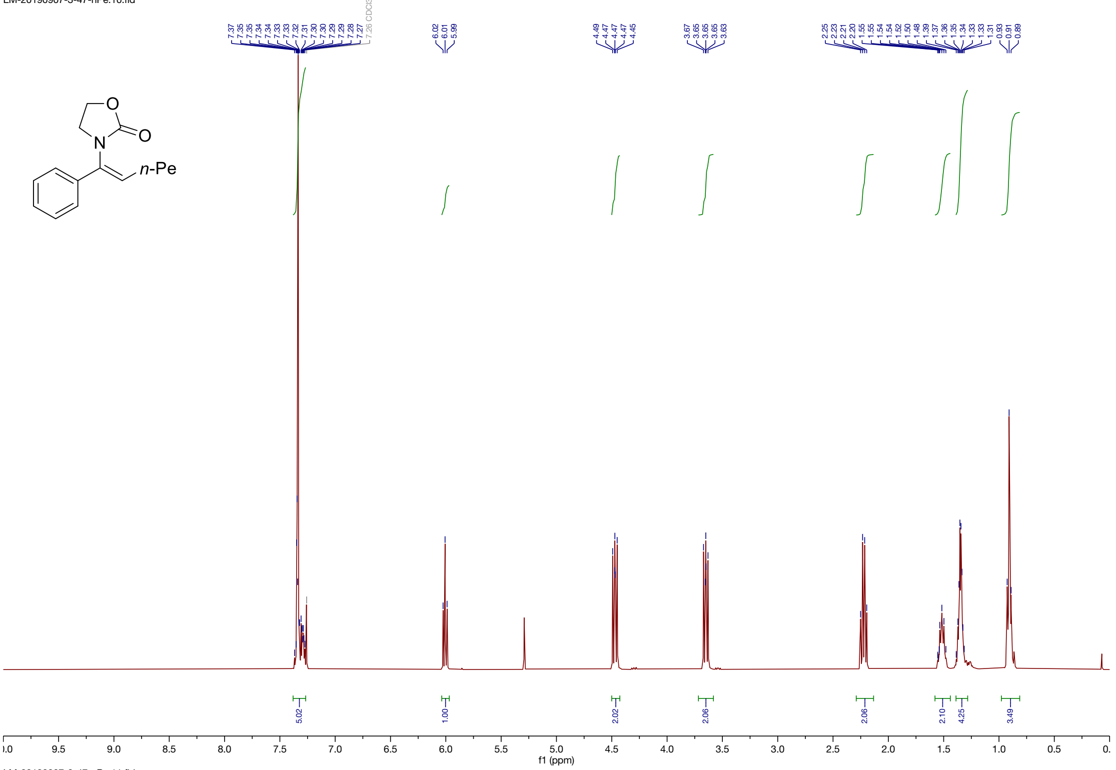

LM-20190907-3-47-nPe.11.fid

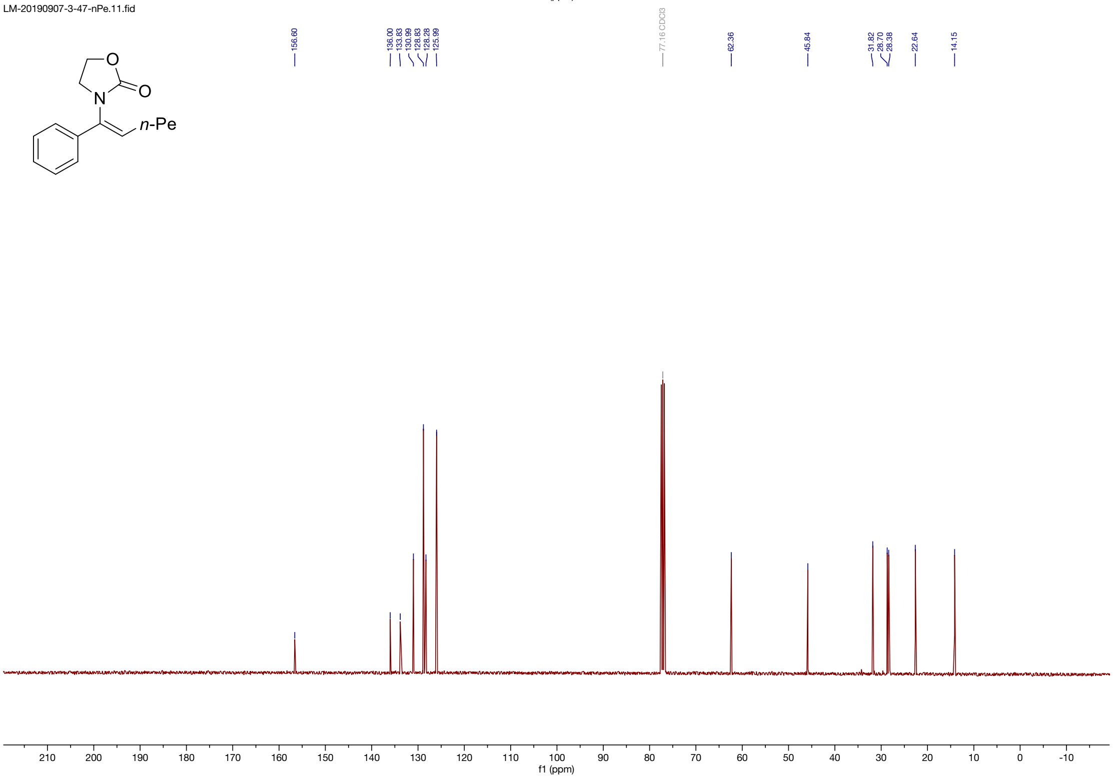

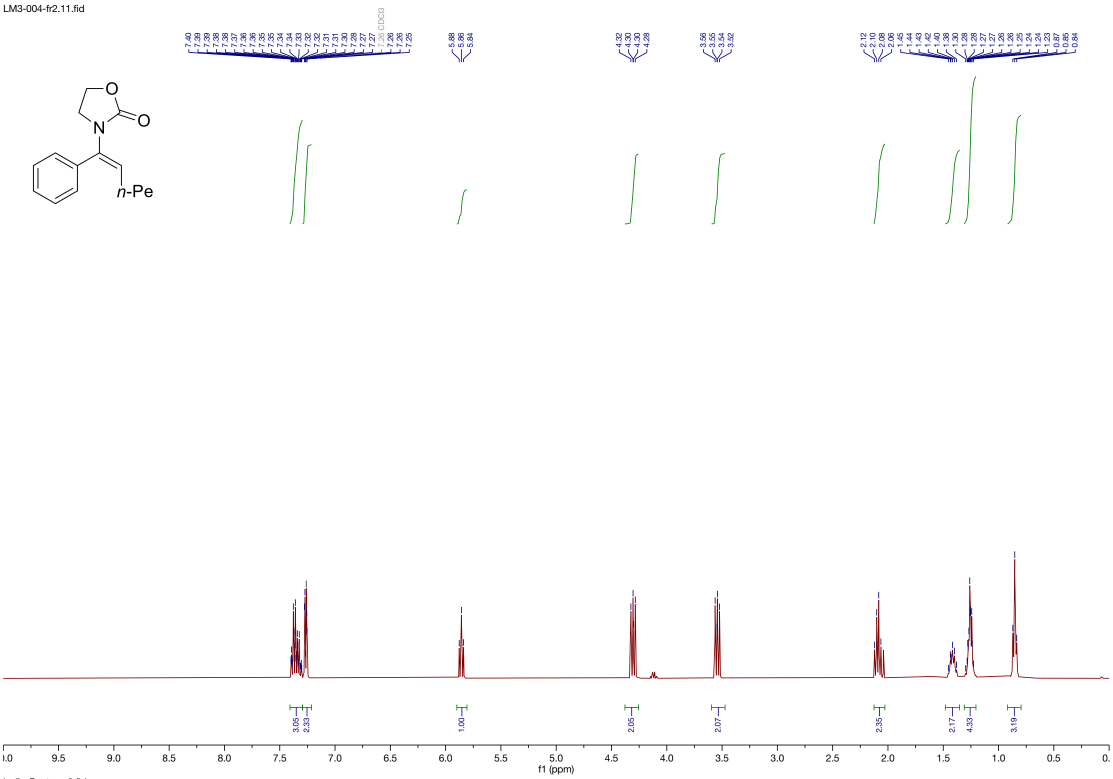

LM3-004-fr2.11.fid
7.40 7.39 7.39 7.38 7.38 7.37 7.36 7.36 7.35 7.35 7.34 7.34 7.33 7.33 7.32 7.31 7.31 7.30 7.28 7.27 7.27 7.26 CDCl3 7.26 7.26 7.25
5.88 5.86 5.84
4.32 4.30 4.30 4.28
3.56 3.55 3.54 3.52
2.12 2.10 2.08 2.06 1.45 1.44 1.43 1.42 1.40 1.38 1.30 1.28 1.28 1.27 1.27 1.26 1.25 1.24 1.24 1.23 0.87 0.85 0.84
O
N
O
n-Pe
3.05 2.33 1.00 2.05 2.07 2.35 2.17 4.33 3.19
9.5 9.0 8.5 8.0 7.5 7.0 6.5 6.0 5.5 5.0 4.5 4.0 3.5 3.0 2.5 2.0 1.5 1.0 0.5
f1 (ppm)

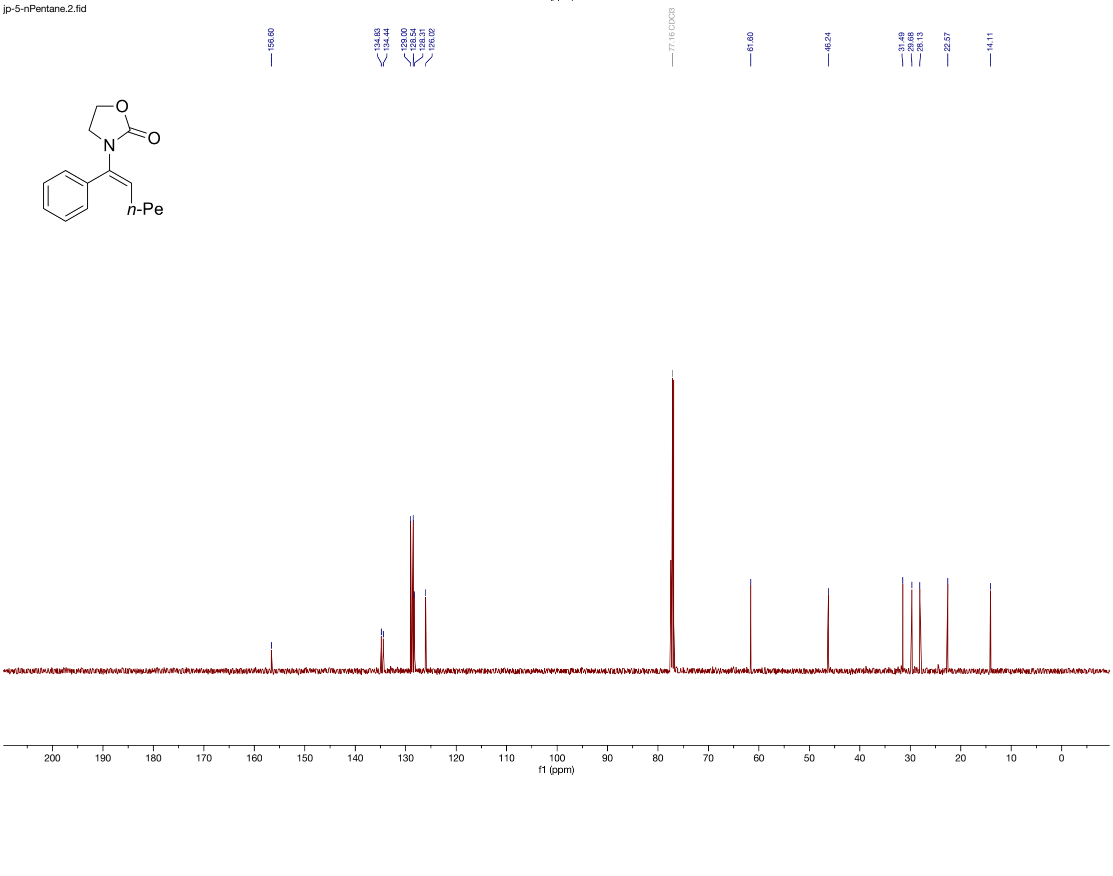

jp-5-nPentane.2.fid
156.60
134.83 134.44 129.00 128.54 128.31 126.02
77.16 CDCl3
61.60
46.24
31.49 29.68 28.13 22.57 14.11
O
N
O
n-Pe
200 190 180 170 160 150 140 130 120 110 100 90 80 70 60 50 40 30 20 10 0
f1 (ppm)

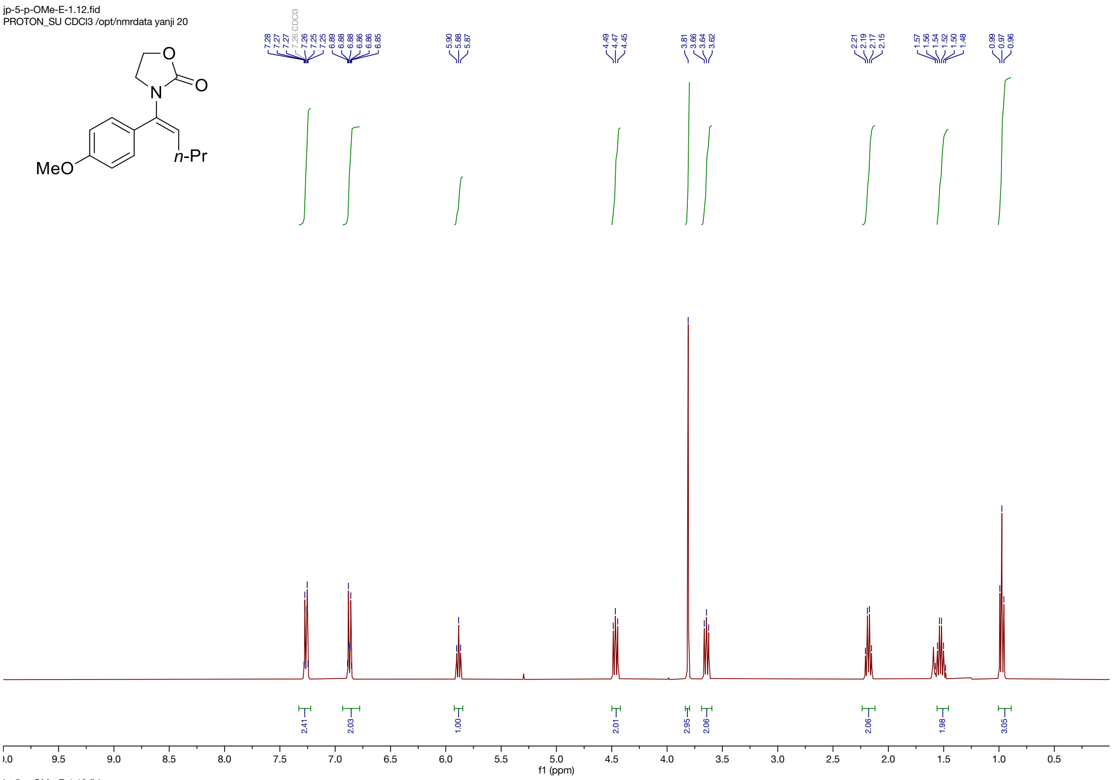
jp-5-p-OMe-E-1.12.fid
PROTON_SU CDCl3 /opt/nmrdata yanji 20
O
N
O
MeO
n-Pr
7.28 7.27 7.27 7.26 CDCl3 7.26 7.25 7.25 6.89 6.88 6.88 6.86 6.86 6.85
5.90 5.88 5.87
4.49 4.47 4.45
3.81 3.66 3.64 3.62
2.21 2.19 2.17 2.15
1.57 1.56 1.54 1.52 1.50 1.48
0.99 0.97 0.96
2.41 2.03 1.00 2.01 2.95 2.06 2.06 1.98 3.05
10.0 9.5 9.0 8.5 8.0 7.5 7.0 6.5 6.0 5.5 5.0 4.5 4.0 3.5 3.0 2.5 2.0 1.5 1.0 0.5
f1 (ppm)

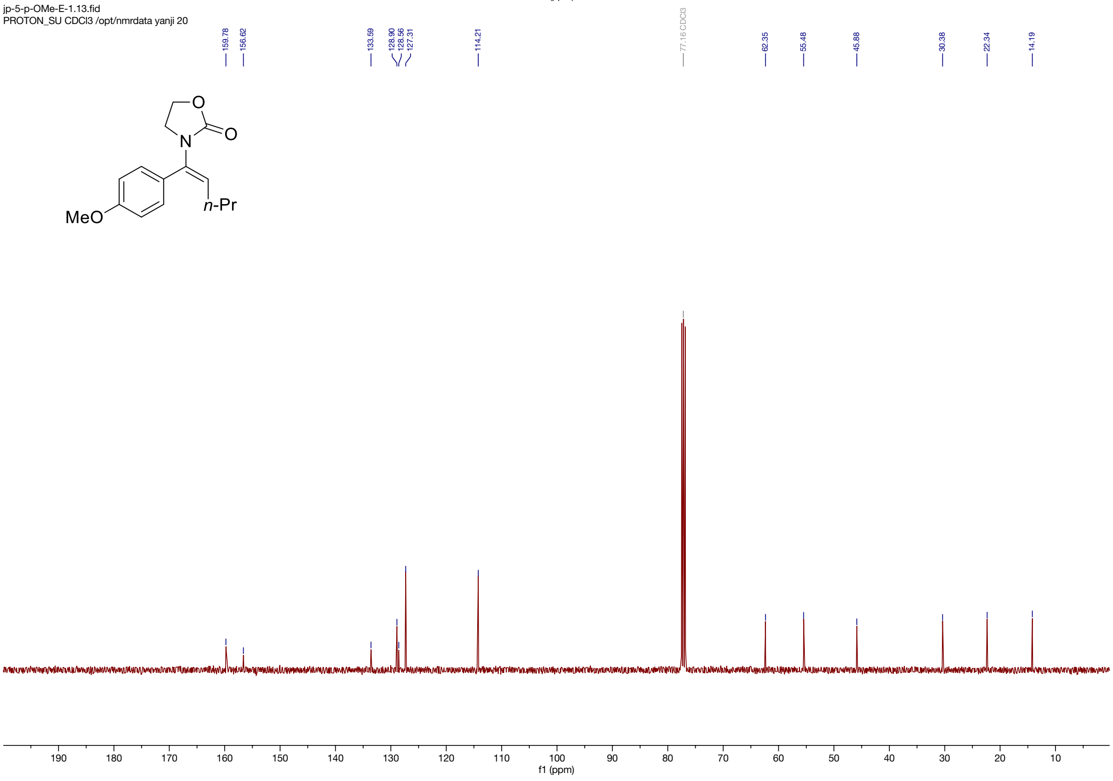
jp-5-p-OMe-E-1.13.fid
PROTON_SU CDCl3 /opt/nmrdata yanji 20
159.78
156.62
133.59
128.90
128.56
127.31
114.21
77.16 CDCl3
62.35
55.48
45.88
30.38
22.34
14.19
O
N
O
MeO
n-Pr
190 180 170 160 150 140 130 120 110 100 90 80 70 60 50 40 30 20 10
f1 (ppm)

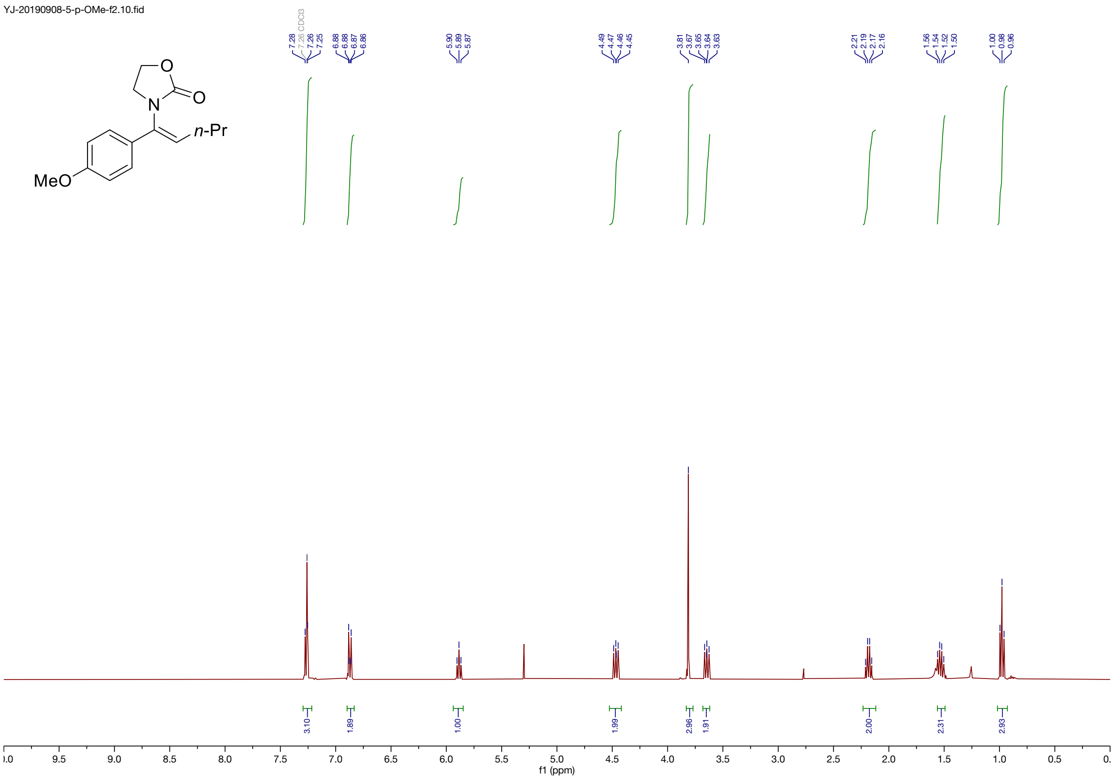
YJ-20190908-5-p-OMe-f2.10.fid
7.28
7.26 CDCl3
7.26
7.25
6.88
6.88
6.87
6.86
5.90
5.89
5.87
4.49
4.47
4.46
4.45
3.81
3.67
3.65
3.64
3.63
2.21
2.19
2.17
2.16
1.56
1.54
1.52
1.50
1.00
0.98
0.96
N
O
O
n-Pr
MeO
3.10
1.89
1.00
1.99
2.96
1.91
2.00
2.31
2.93
f1 (ppm)

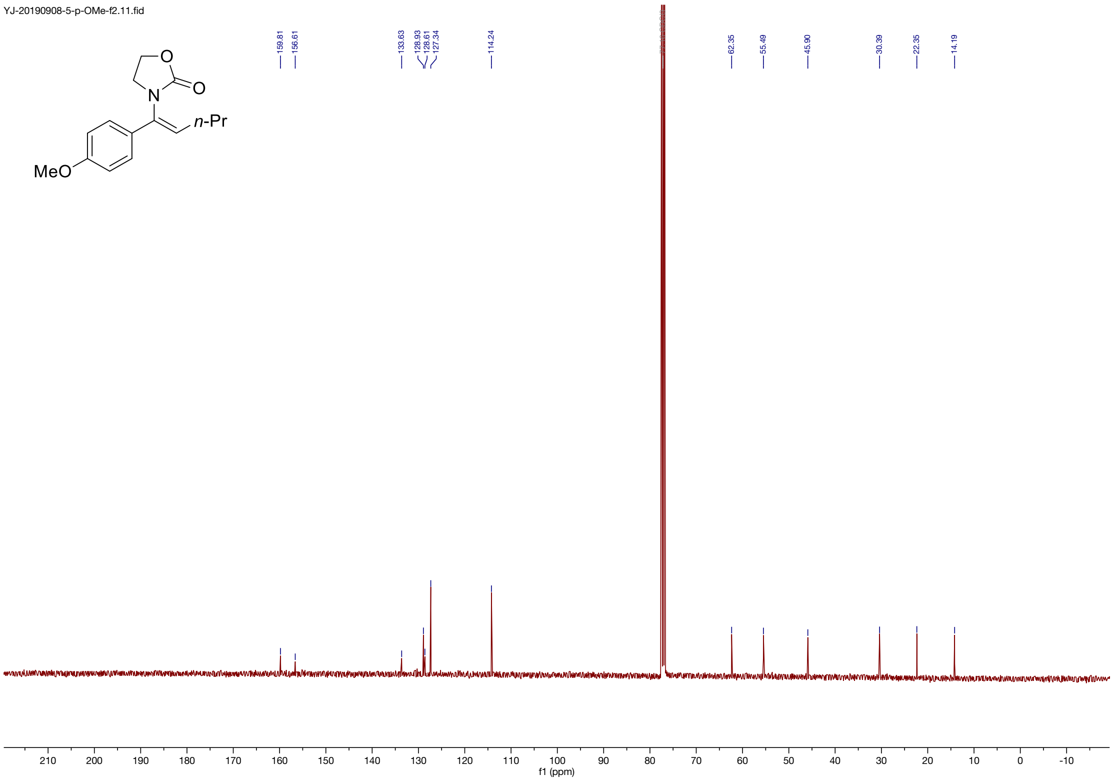
YJ-20190908-5-p-OMe-f2.11.fid
159.81
156.61
133.63
128.93
128.61
127.34
114.24
62.35
55.49
45.90
30.39
22.35
14.19
N
O
O
n-Pr
MeO
f1 (ppm)

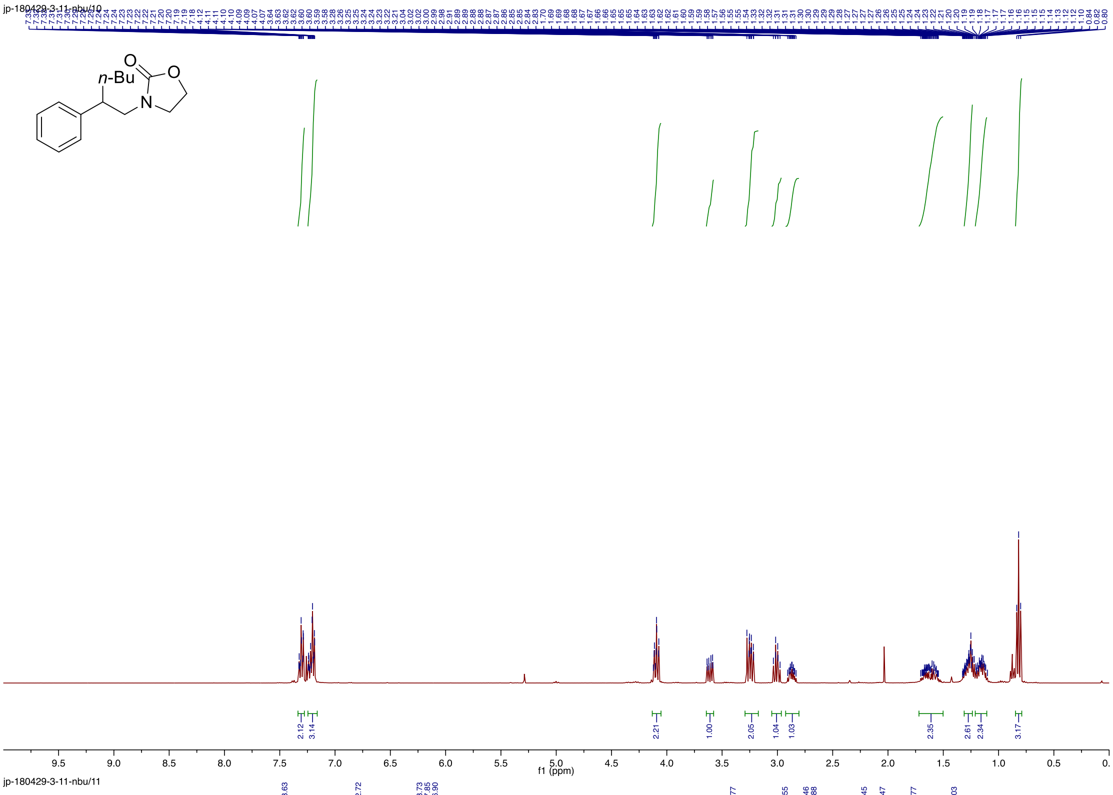
jp-180429-3-11-nbu/10
n-Bu
O
N
O
2.12
3.14
2.21
1.00
2.05
1.04
1.03
2.35
2.61
2.34
3.17
f1 (ppm)

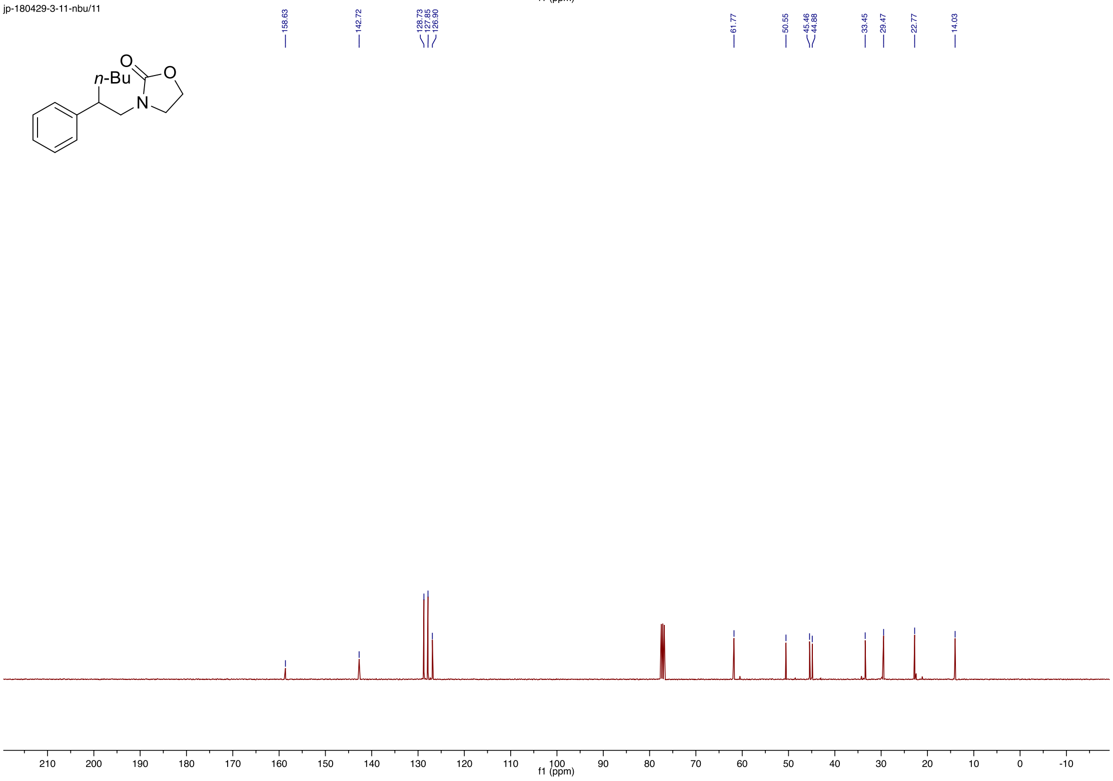
jp-180429-3-11-nbu/11
158.63
142.72
128.73
127.85
126.90
61.77
50.55
45.46
44.88
33.45
29.47
22.77
14.03
n-Bu
O
N
O
f1 (ppm)

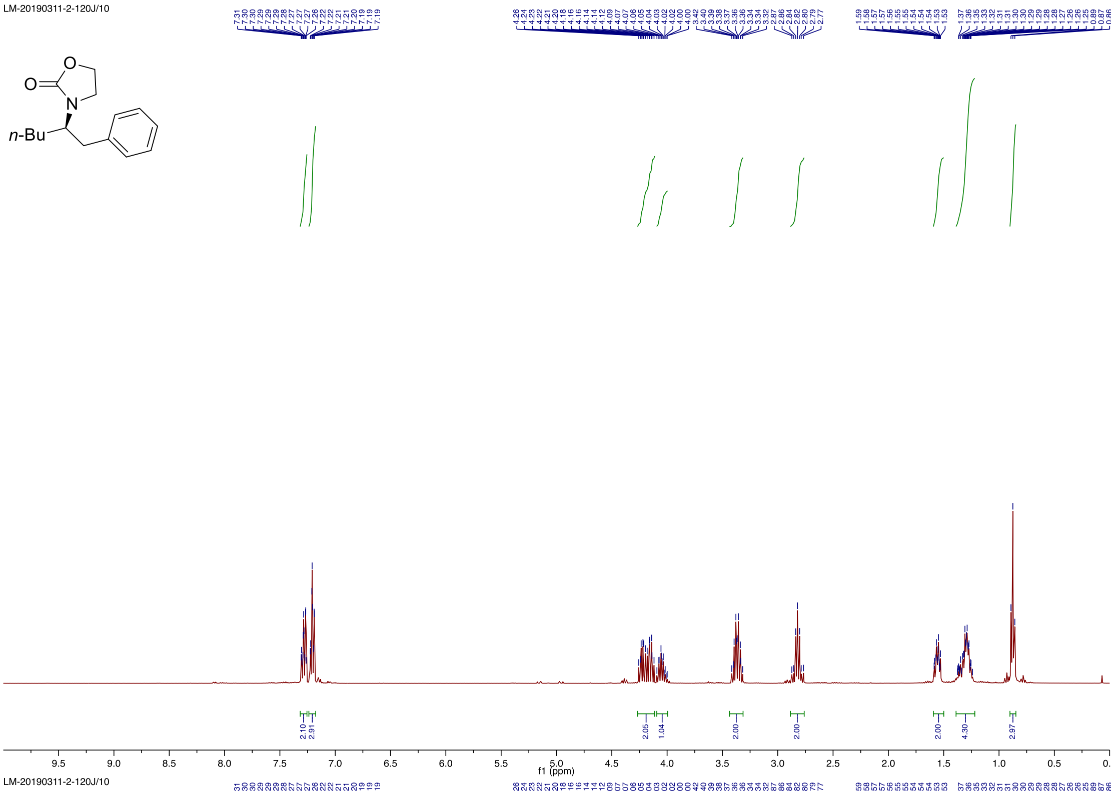
LM-20190311-2-120J/10
n-Bu
f1 (ppm)

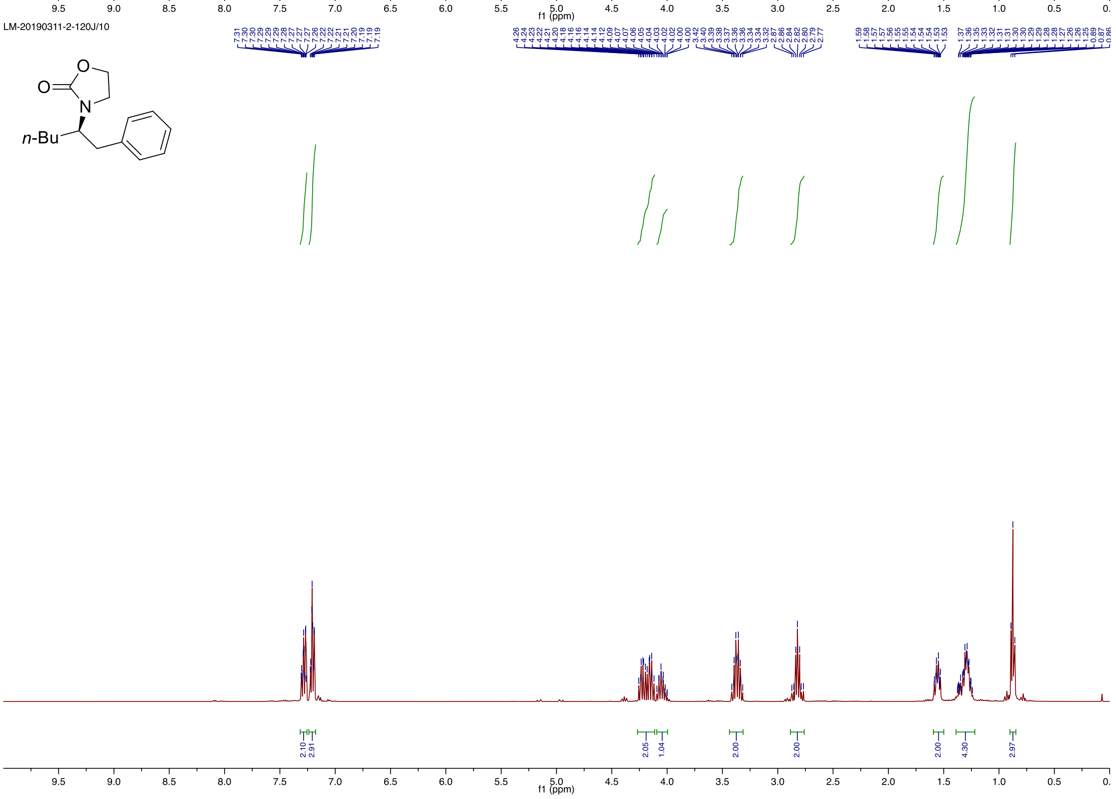
LM-20190311-2-120J/10
n-Bu
f1 (ppm)

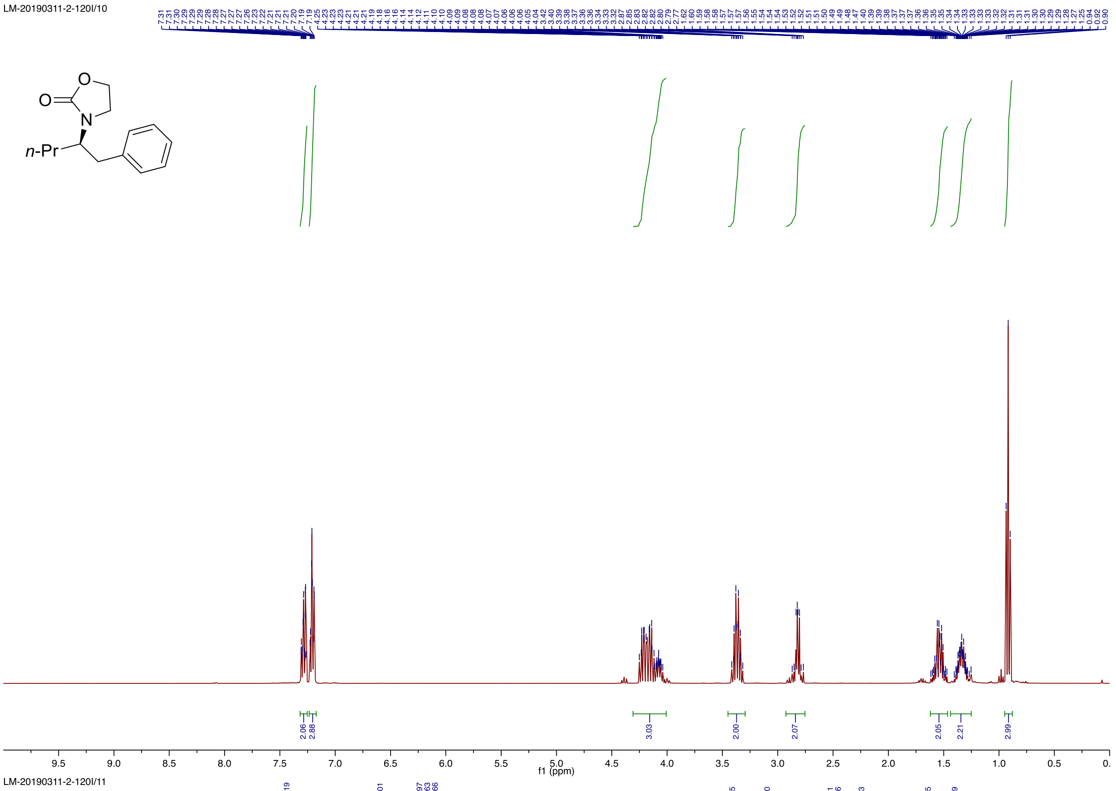
LM-20190311-2-120I/10
O
N
n-Pr
2.06
2.88
3.03
2.00
2.07
2.05
2.21
2.99
f1 (ppm)

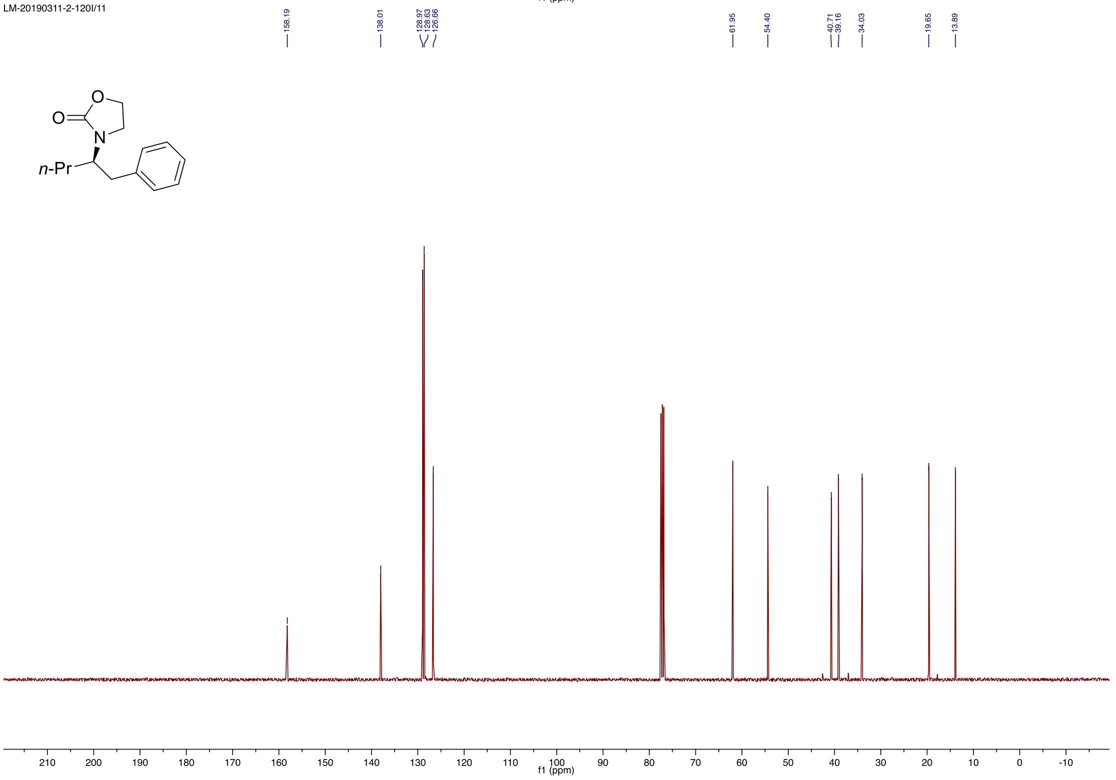
LM-20190311-2-120I/11
158.19
138.01
128.97
128.63
126.66
61.95
54.40
40.71
39.16
34.03
19.65
13.89
O
N
n-Pr
f1 (ppm)

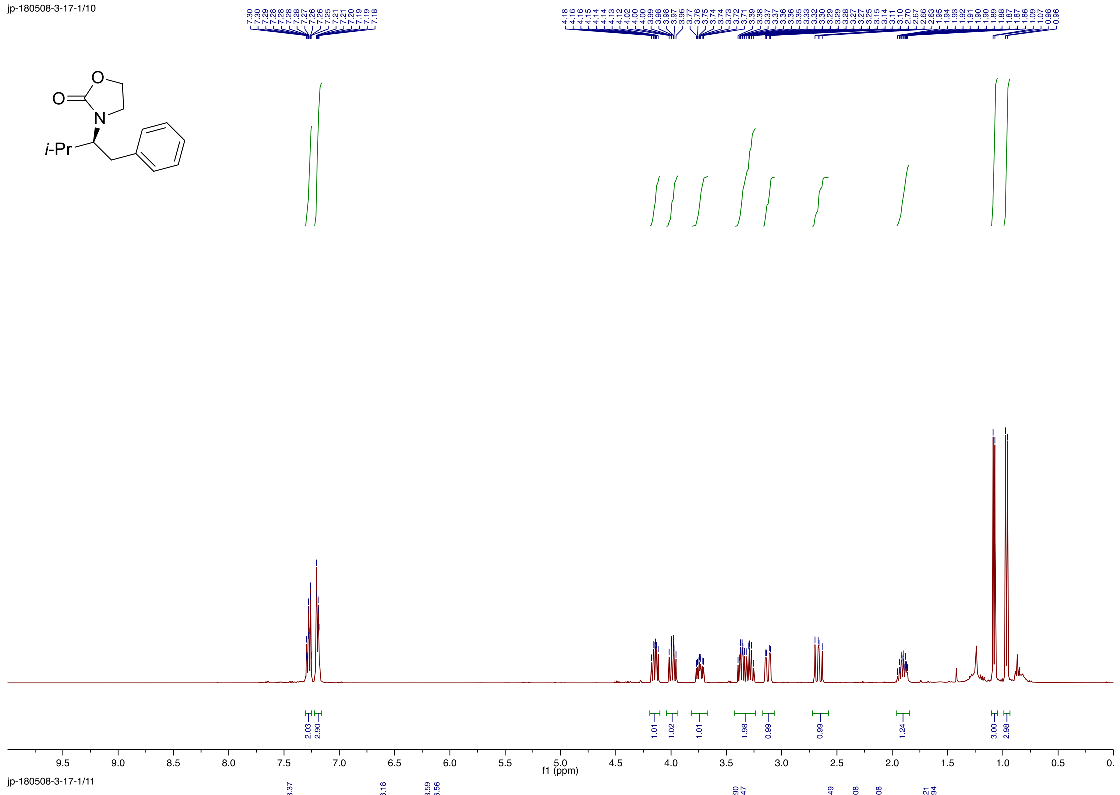
jp-180508-3-17-1/10
O
N
i-Pr
7.30 7.30 7.29 7.28 7.28 7.28 7.27 7.26 7.26 7.25 7.21 7.21 7.20 7.19 7.19 7.18
4.18 4.16 4.16 4.15 4.14 4.14 4.13 4.12 4.02 4.00 3.99 3.98 3.98 3.97 3.96 3.77 3.76 3.75 3.74 3.74 3.73 3.72 3.71 3.39 3.38 3.37 3.37 3.36 3.36 3.35 3.33 3.32 3.30 3.29 3.29 3.28 3.27 3.27 3.25 3.15 3.14 3.11 3.10 2.70 2.67 2.66 2.63 1.95 1.94 1.93 1.92 1.91 1.90 1.90 1.89 1.88 1.87 1.87 1.86 1.09 1.07 0.98 0.96
2.03 2.90 1.01 1.02 1.01 1.98 0.99 0.99 1.24 3.00 2.98
f1 (ppm)

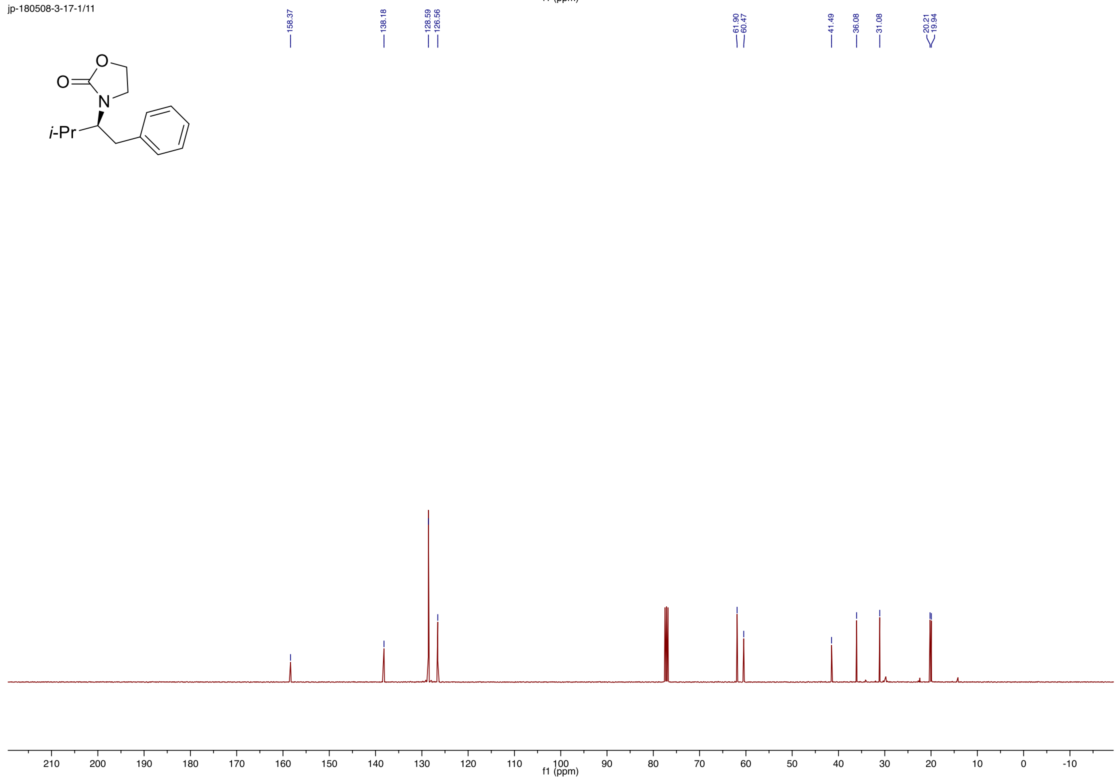
jp-180508-3-17-1/11
O
N
i-Pr
158.37
138.18
128.59
126.56
61.90
60.47
41.49
36.08
31.08
20.21
19.94
f1 (ppm)

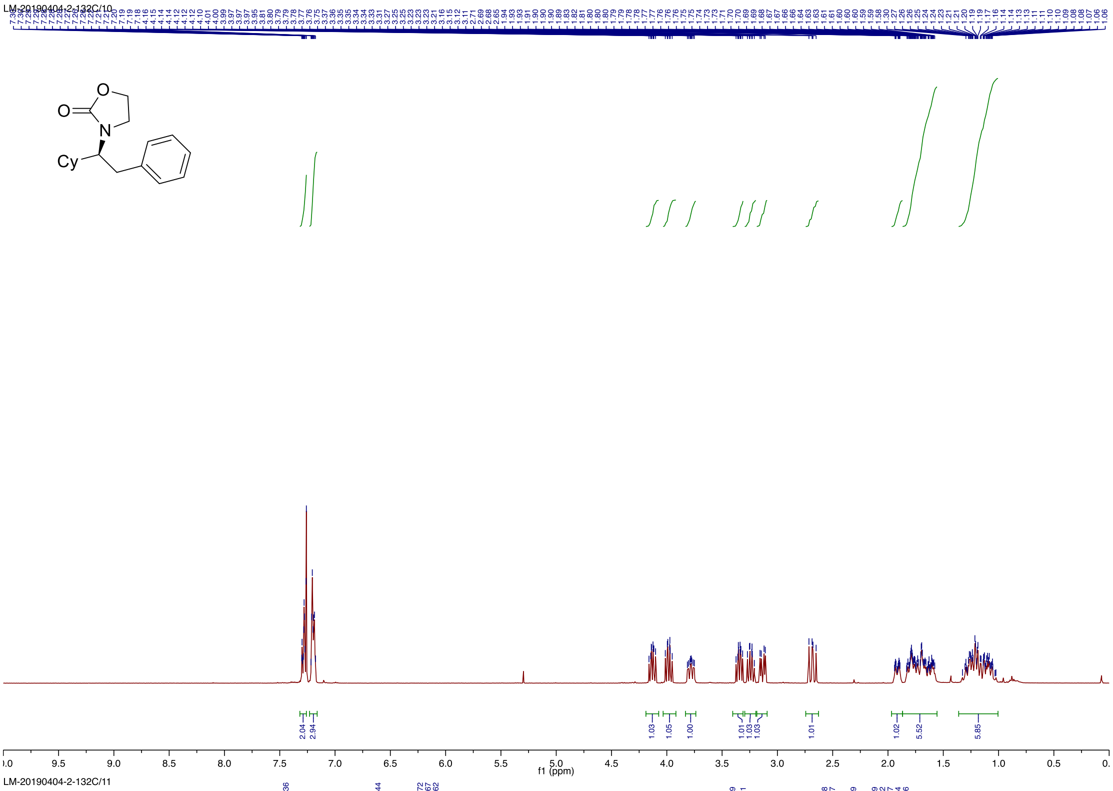

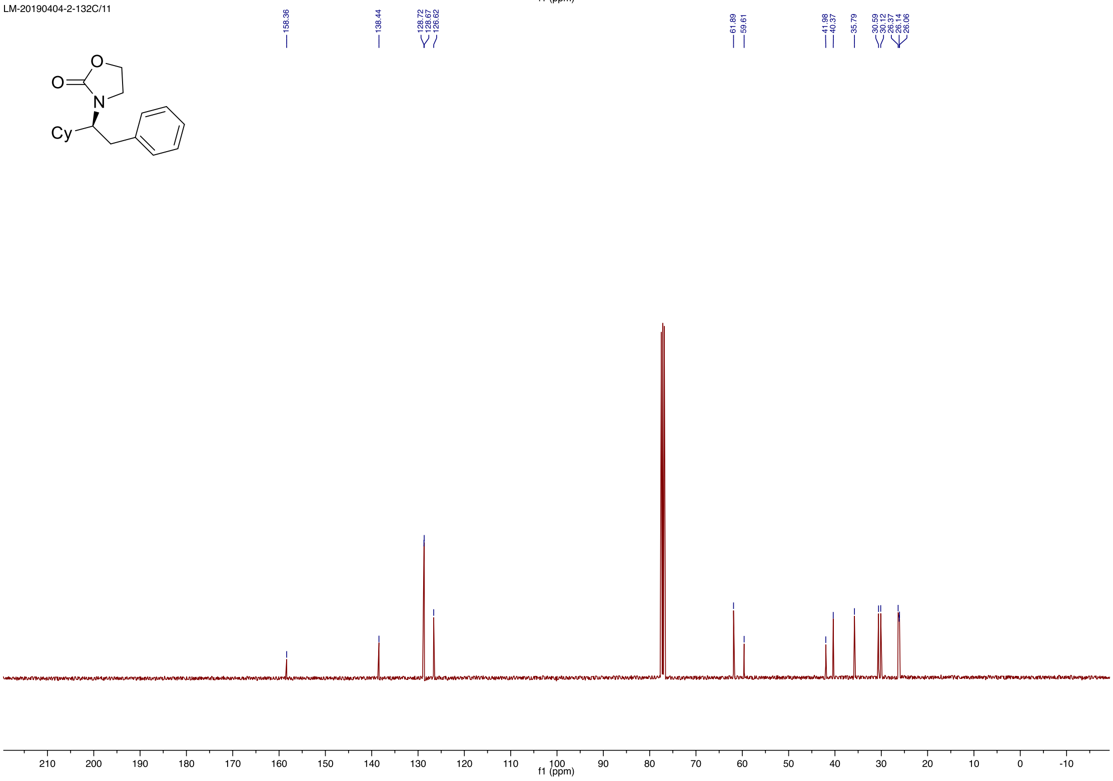

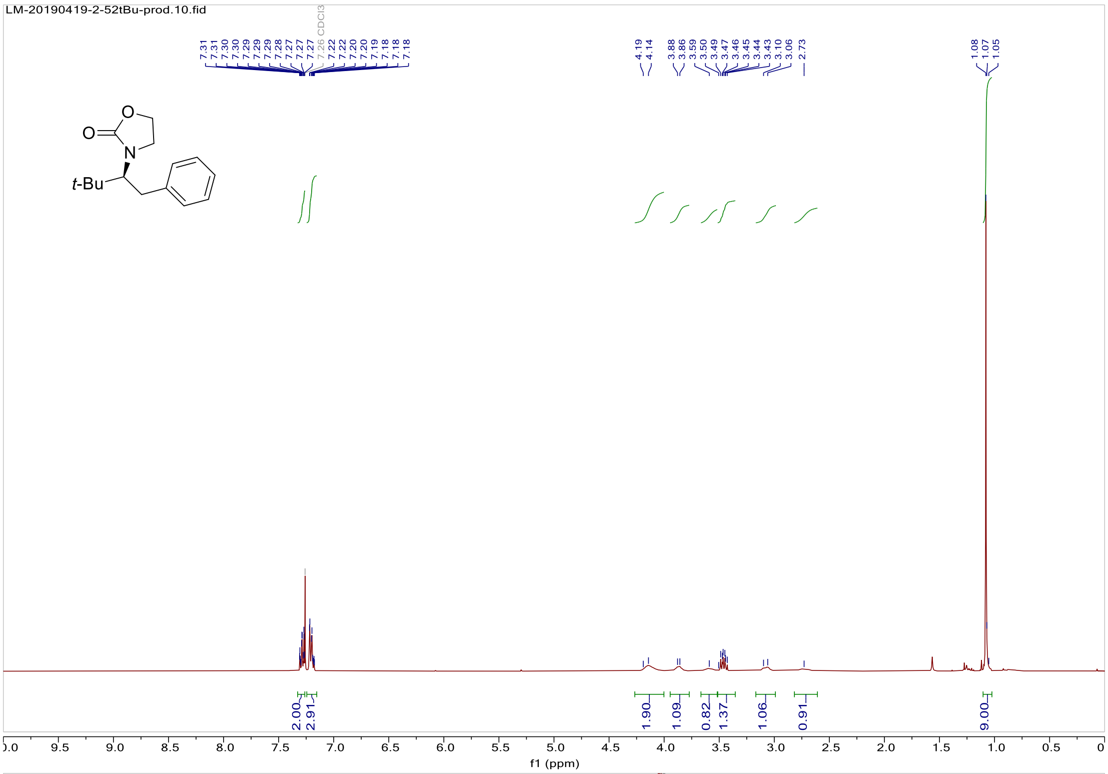
LM-20190419-2-52tBu-prod.10.fid
t-Bu
f1 (ppm)

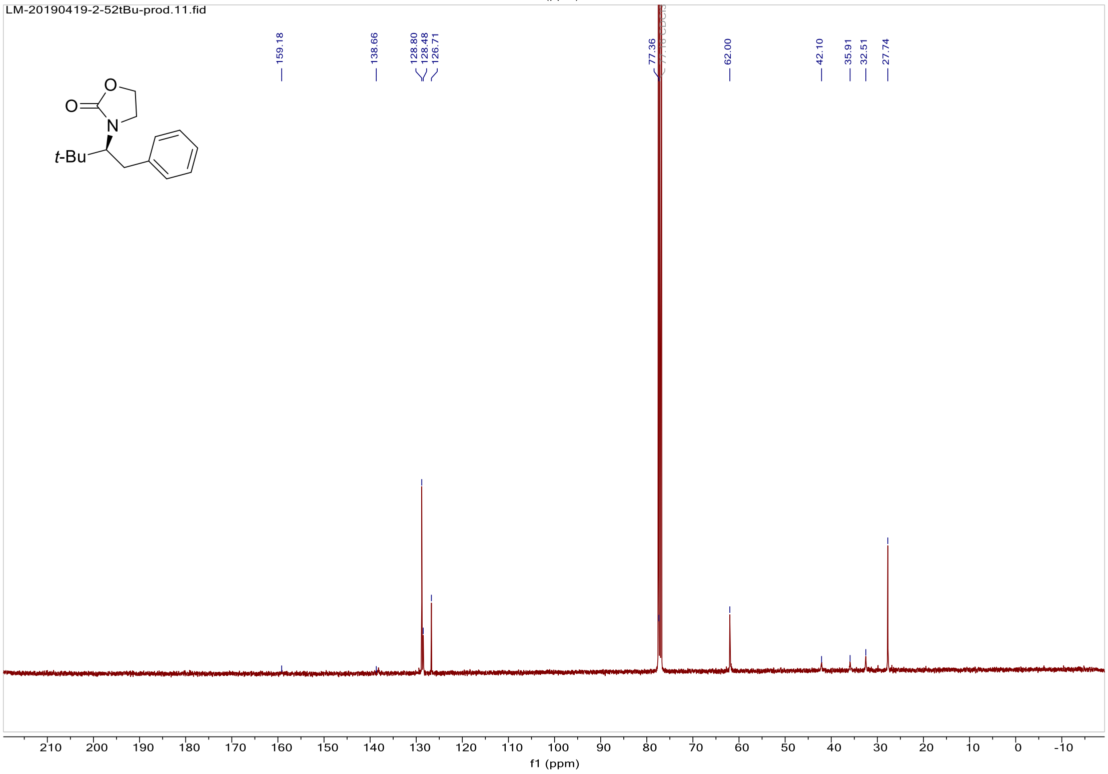
LM-20190419-2-52tBu-prod.11.fid
t-Bu
f1 (ppm)

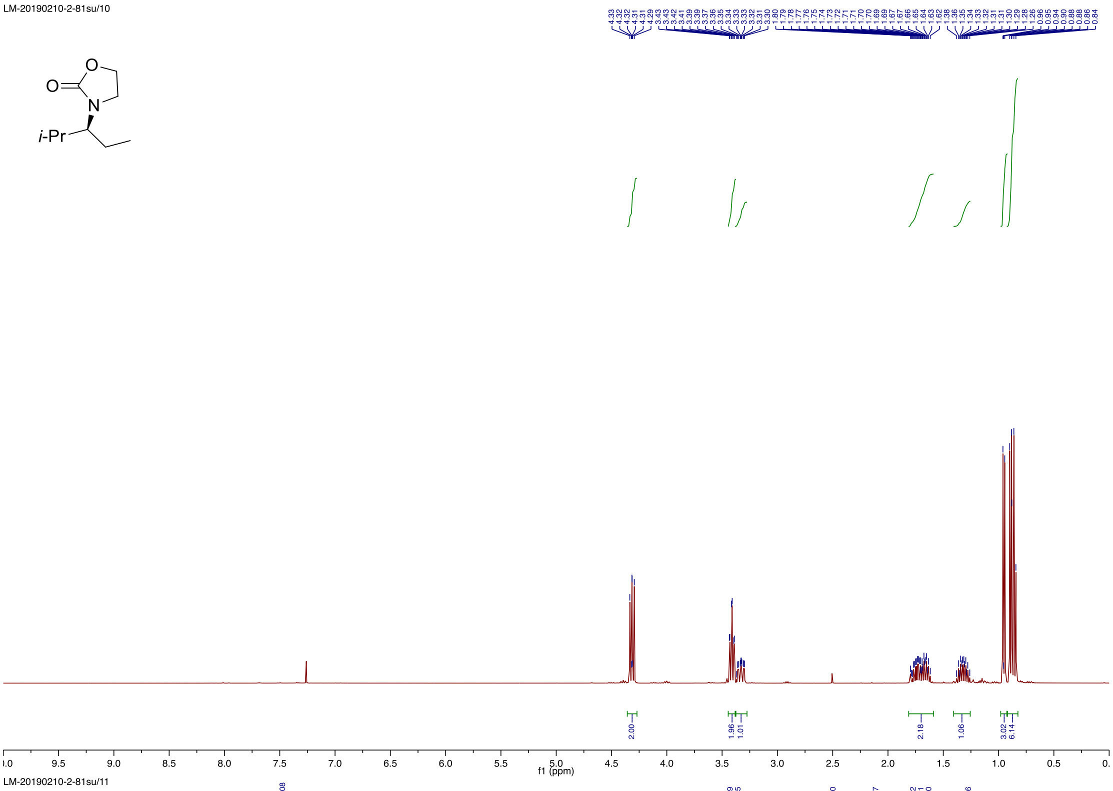

LM-20190210-2-81su/10
O
N
i-Pr
2.00
1.96
1.01
2.18
1.06
3.02
6.14
f1 (ppm)

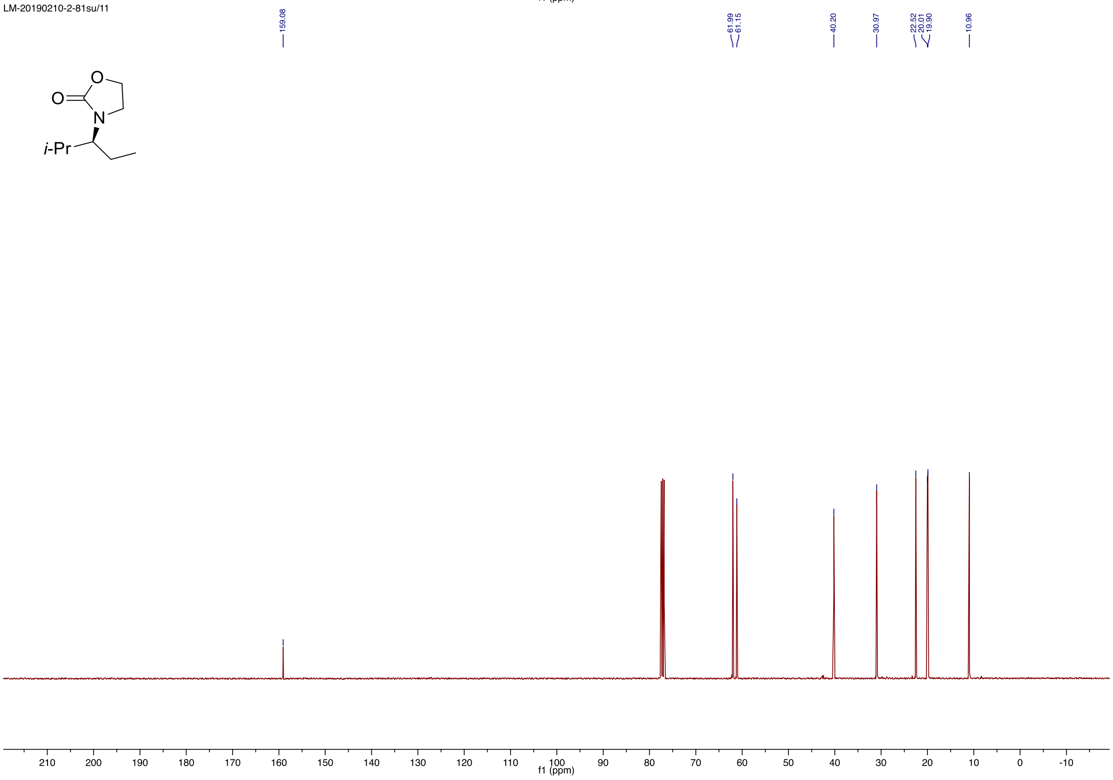

LM-20190210-2-81su/11
159.08
61.99
61.15
40.20
30.97
22.52
20.01
19.90
10.96
O
N
i-Pr
f1 (ppm)

YJ-20190909-5-HT-nBu-1.10.fid

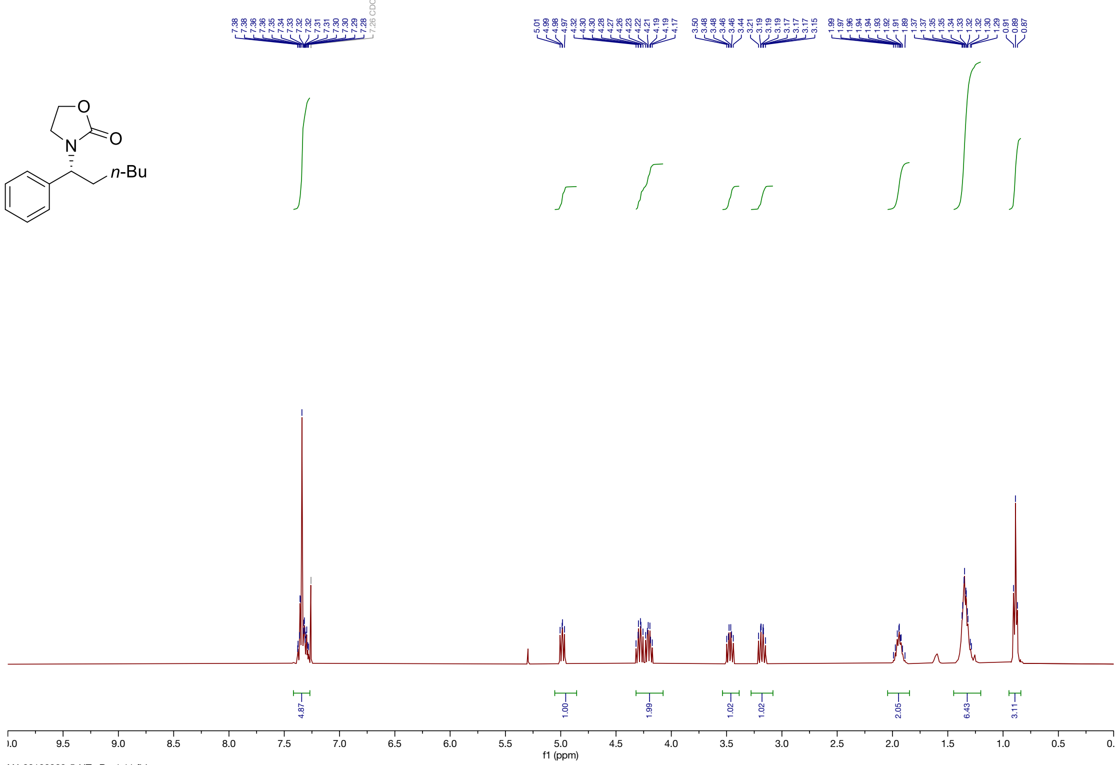

YJ-20190909-5-HT-nBu-1.11.fid

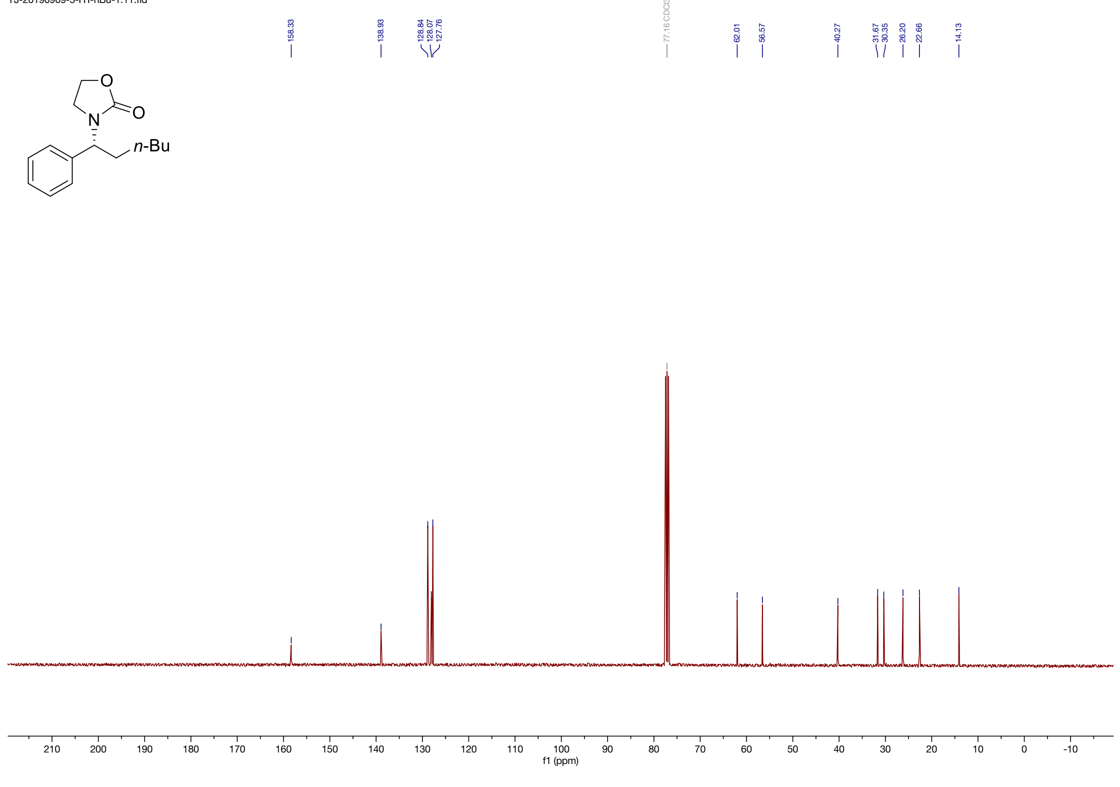

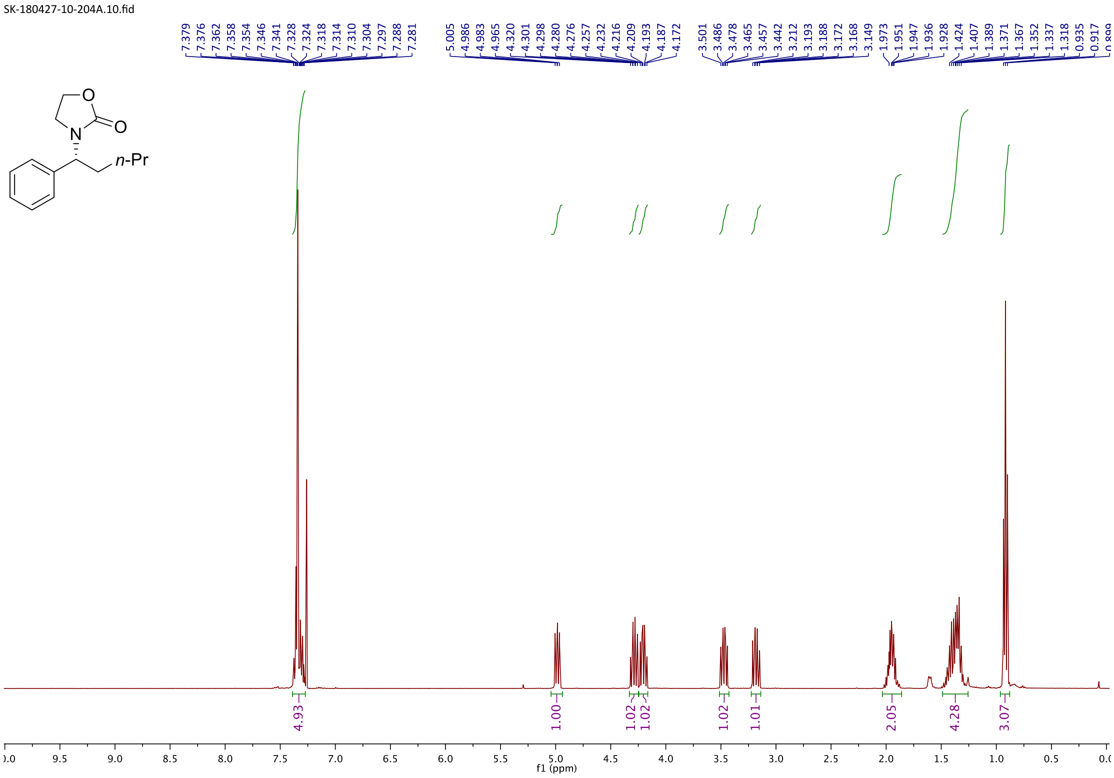
SK-180427-10-204A.10.fid
n-Pr
4.93
1.00
1.02
1.02
1.02
1.01
2.05
4.28
3.07
f1 (ppm)

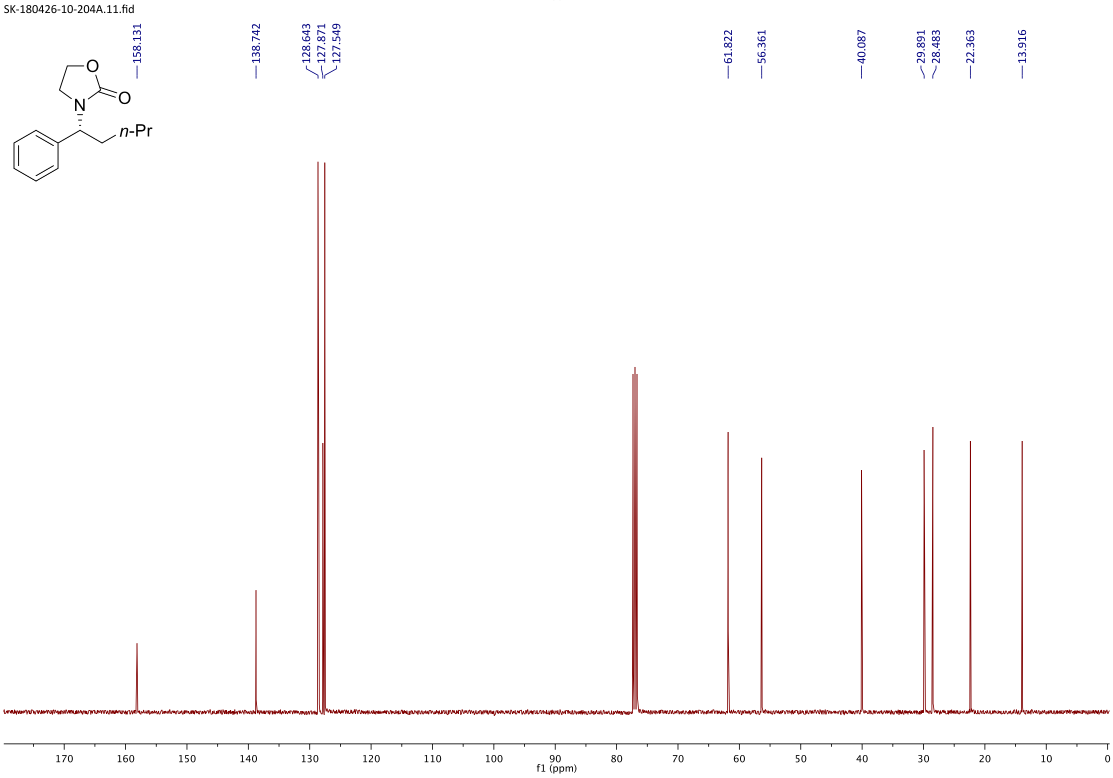
SK-180426-10-204A.11.fid
158.131
138.742
128.643
127.871
127.549
61.822
56.361
40.087
29.891
28.483
22.363
13.916
n-Pr
f1 (ppm)

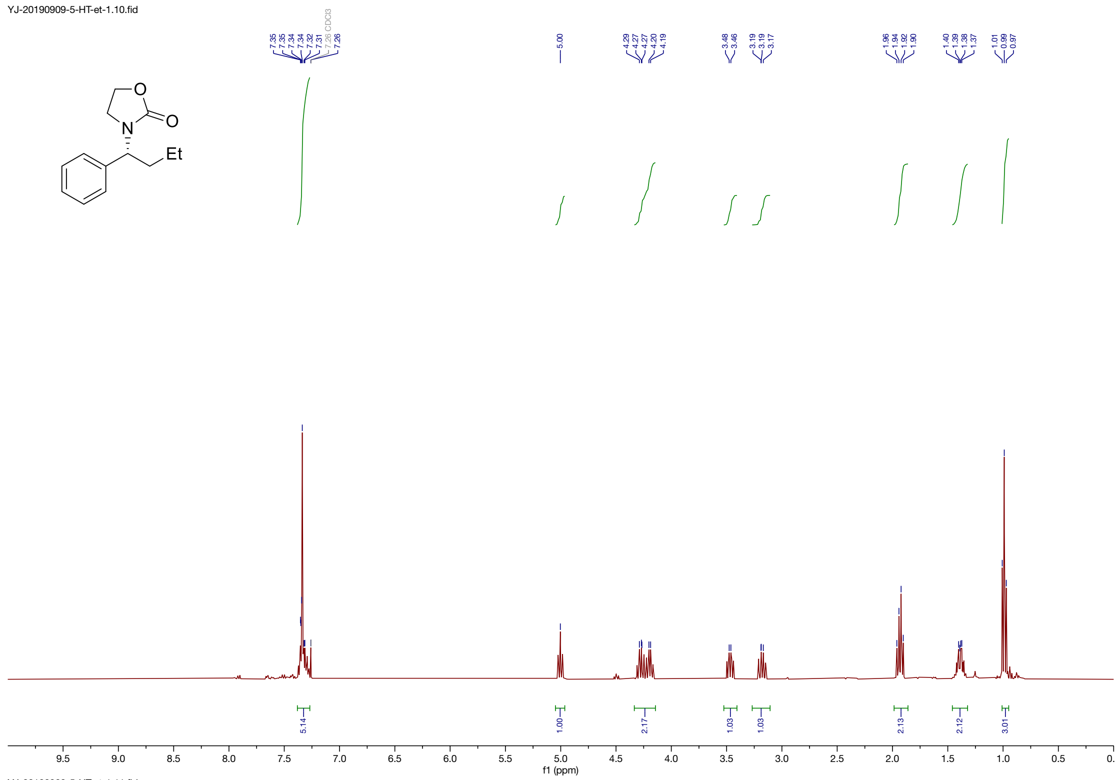
YJ-20190909-5-HT-et-1.10.fid
O
N
O
Et
5.00
4.29
4.27
4.27
4.20
4.19
3.48
3.46
3.19
3.19
3.17
1.96
1.94
1.92
1.90
1.40
1.39
1.38
1.37
1.01
0.99
0.97
5.14
1.00
2.17
1.03
1.03
2.13
2.12
3.01
f1 (ppm)

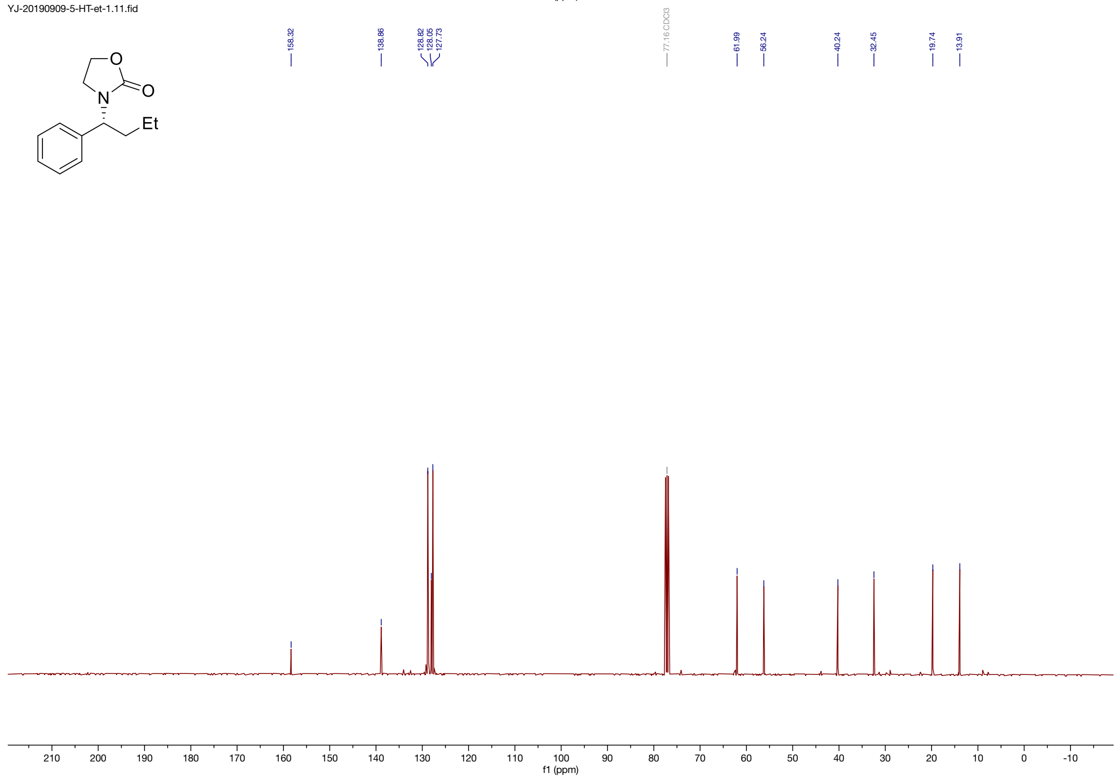
YJ-20190909-5-HT-et-1.11.fid
O
N
O
Et
158.32
138.86
128.82
128.05
127.73
77.16 CDCl3
61.99
56.24
40.24
32.45
19.74
13.91
f1 (ppm)

YJ-20190908-5-HT-nPen.10.fid

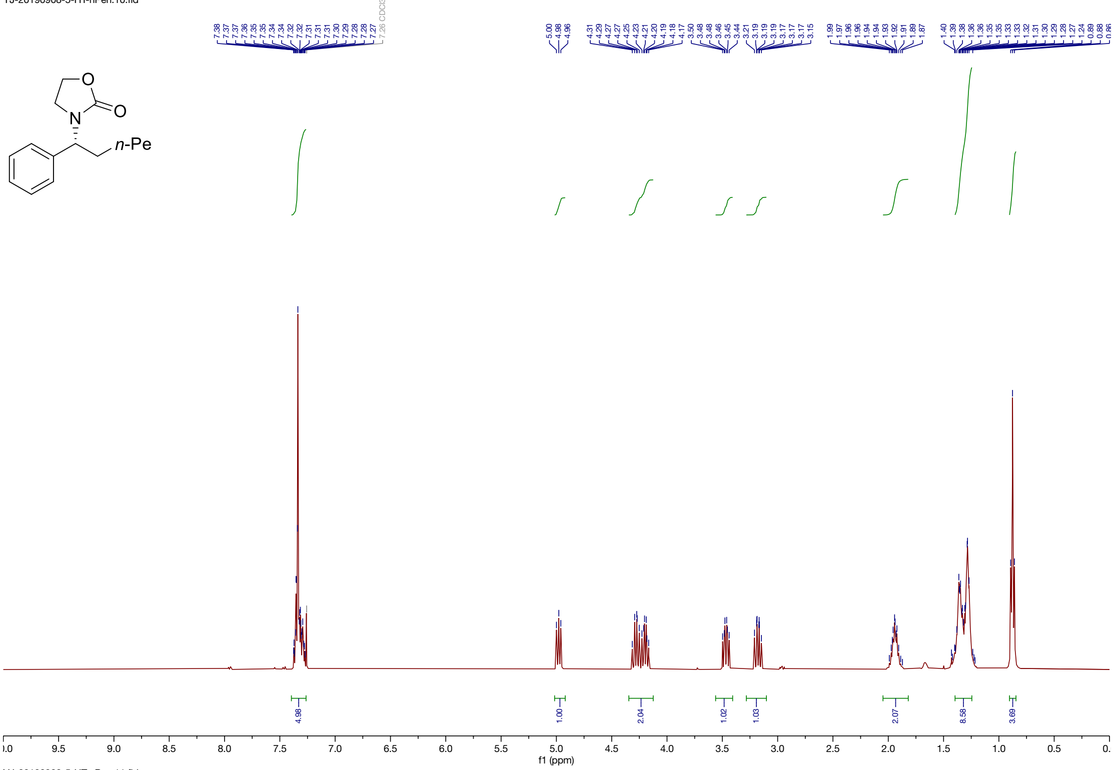

YJ-20190908-5-HT-nPen.11.fid

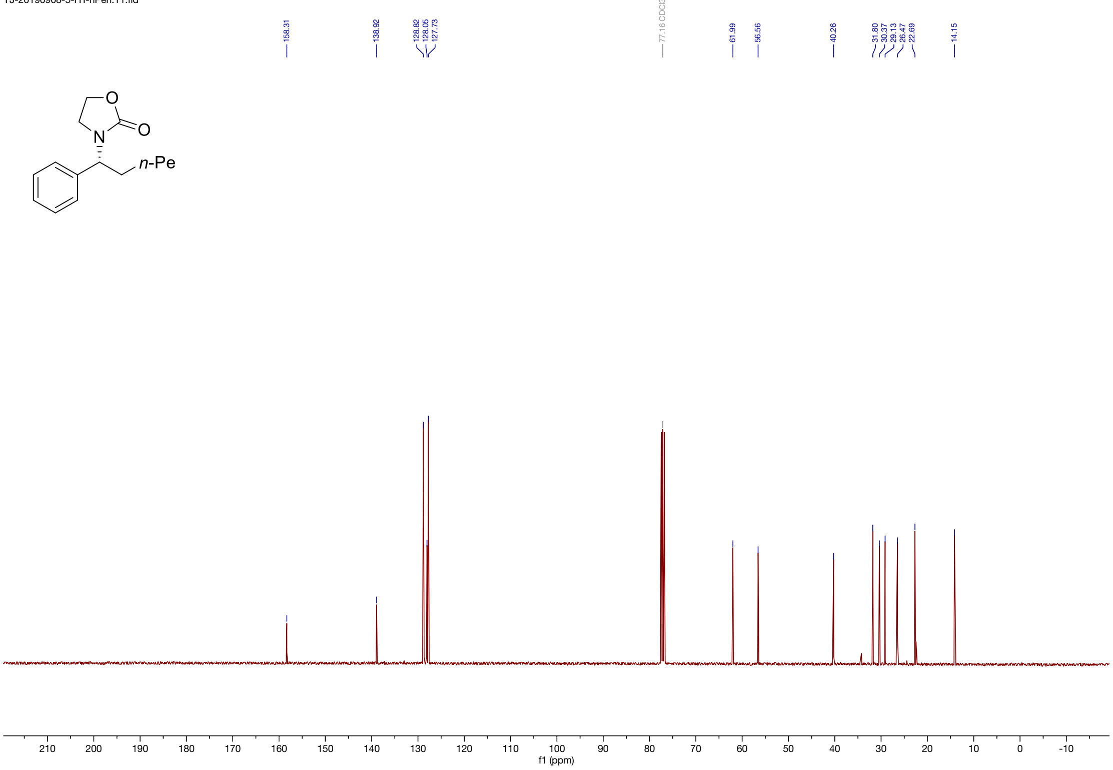

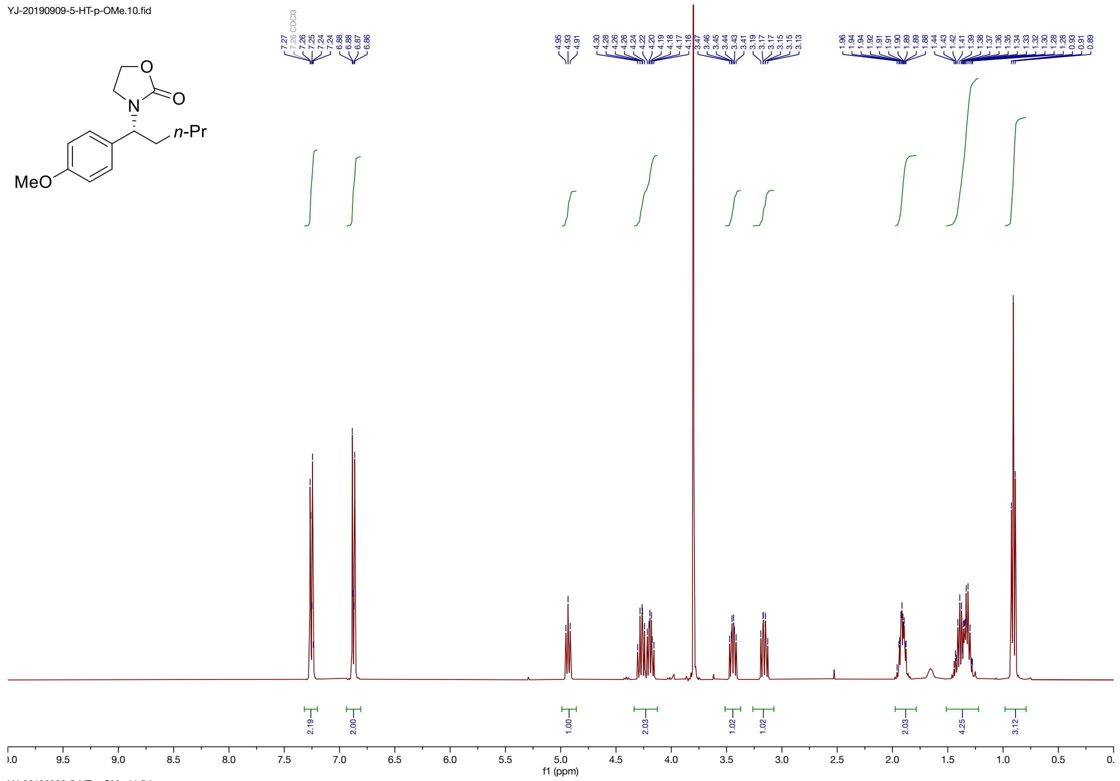
YJ-20190909-5-HT-p-OMe.10.fid
MeO
N
O
O
n-Pr
7.27
7.26 CDCl3
7.26
7.25
7.24
7.24
6.88
6.88
6.87
6.86
4.95
4.93
4.91
4.30
4.28
4.26
4.26
4.24
4.22
4.20
4.19
4.18
4.17
4.16
3.47
3.46
3.45
3.44
3.43
3.41
3.19
3.17
3.17
3.15
3.15
3.13
1.96
1.94
1.94
1.92
1.91
1.91
1.90
1.89
1.89
1.88
1.44
1.43
1.42
1.41
1.39
1.38
1.37
1.36
1.35
1.34
1.33
1.32
1.30
1.29
1.28
0.93
0.91
0.89
2.19
2.00
1.00
2.03
1.02
1.02
2.03
4.25
3.12
f1 (ppm)

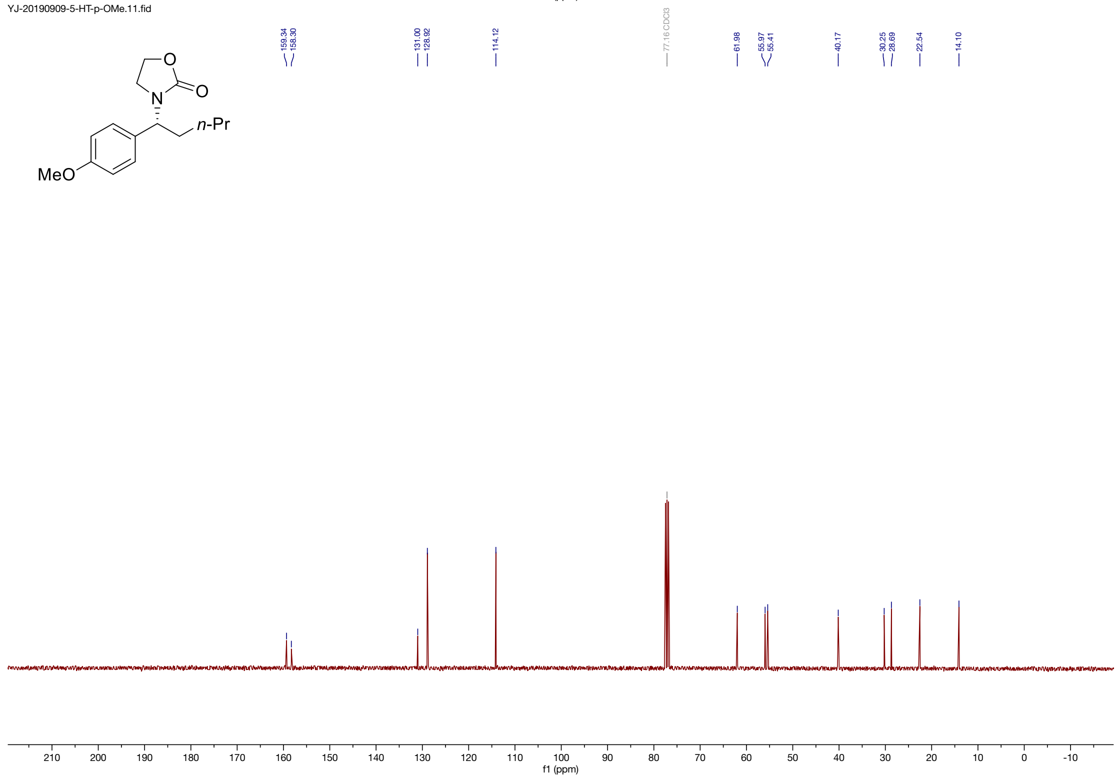
YJ-20190909-5-HT-p-OMe.11.fid
MeO
N
O
O
n-Pr
159.34
158.30
131.00
128.92
114.12
77.16 CDCl3
61.98
55.97
55.41
40.17
30.25
28.69
22.54
14.10
f1 (ppm)

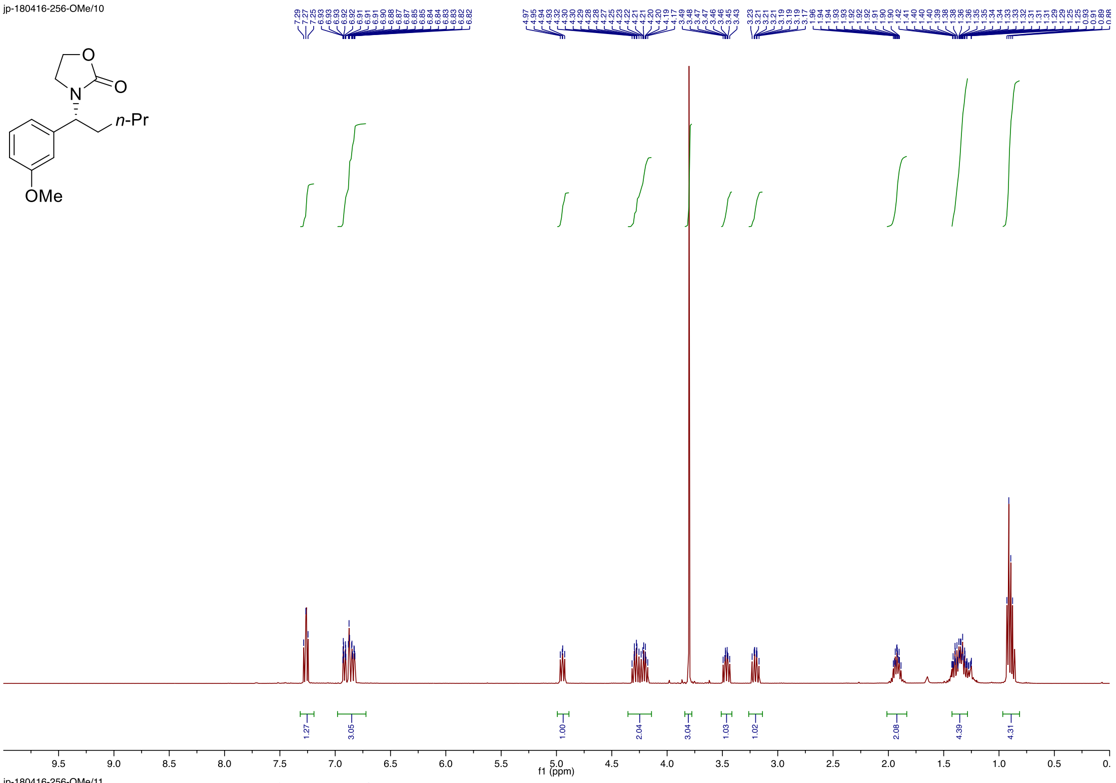
jp-180416-256-OMe/10
O
N
O
n-Pr
OMe
1.27
3.05
1.00
2.04
3.04
1.03
1.02
2.08
4.39
4.31
f1 (ppm)

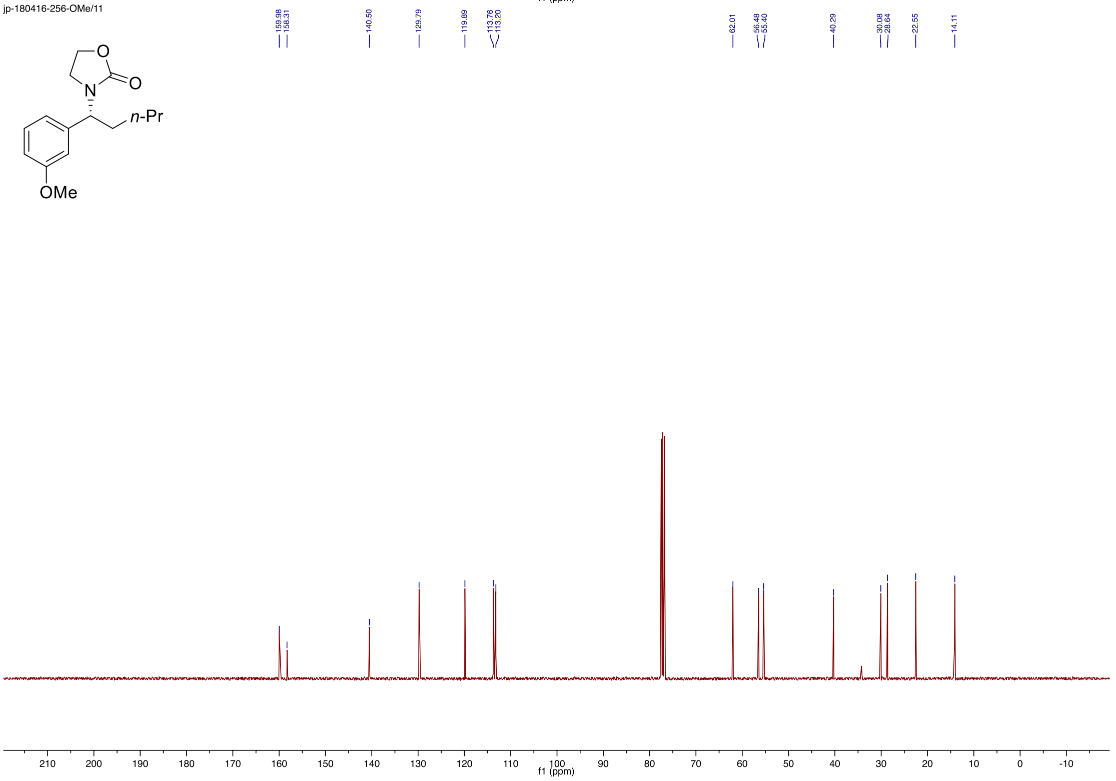
jp-180416-256-OMe/11
159.98
158.31
140.50
129.79
119.89
113.76
113.20
62.01
56.48
55.40
40.29
30.08
28.64
22.55
14.11
O
N
O
n-Pr
OMe
f1 (ppm)

SK-180428-10-232.10.fid

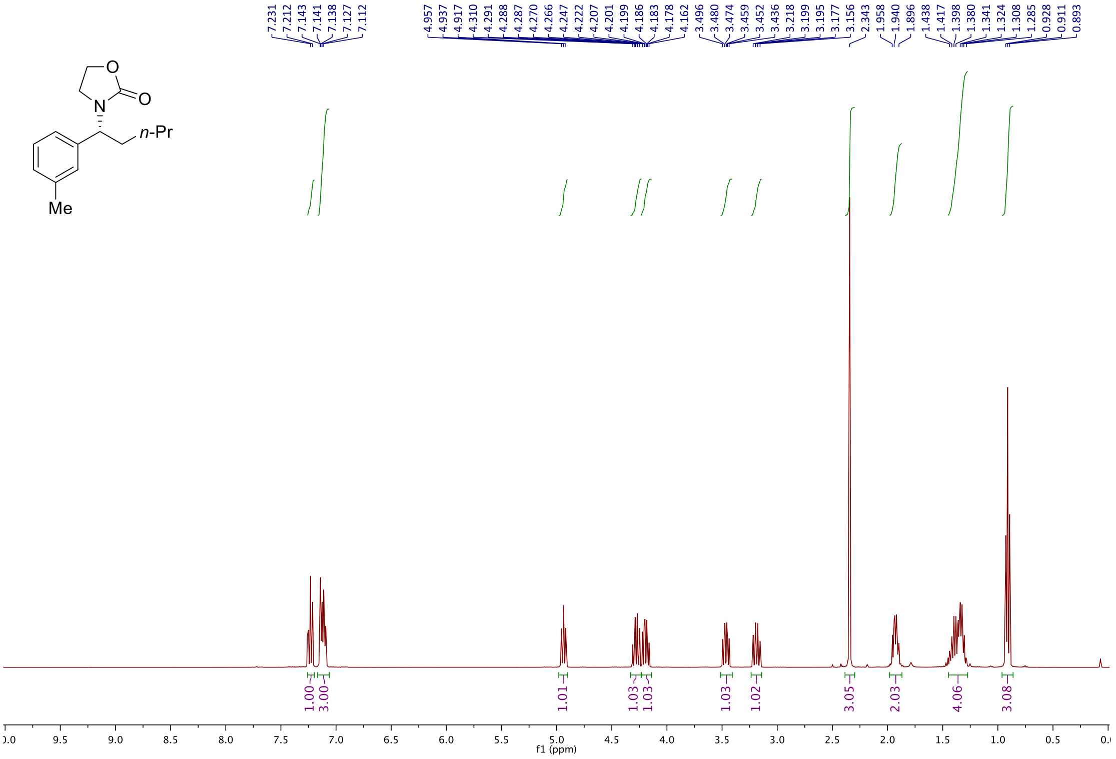

SK-180428-10-232.11.fid

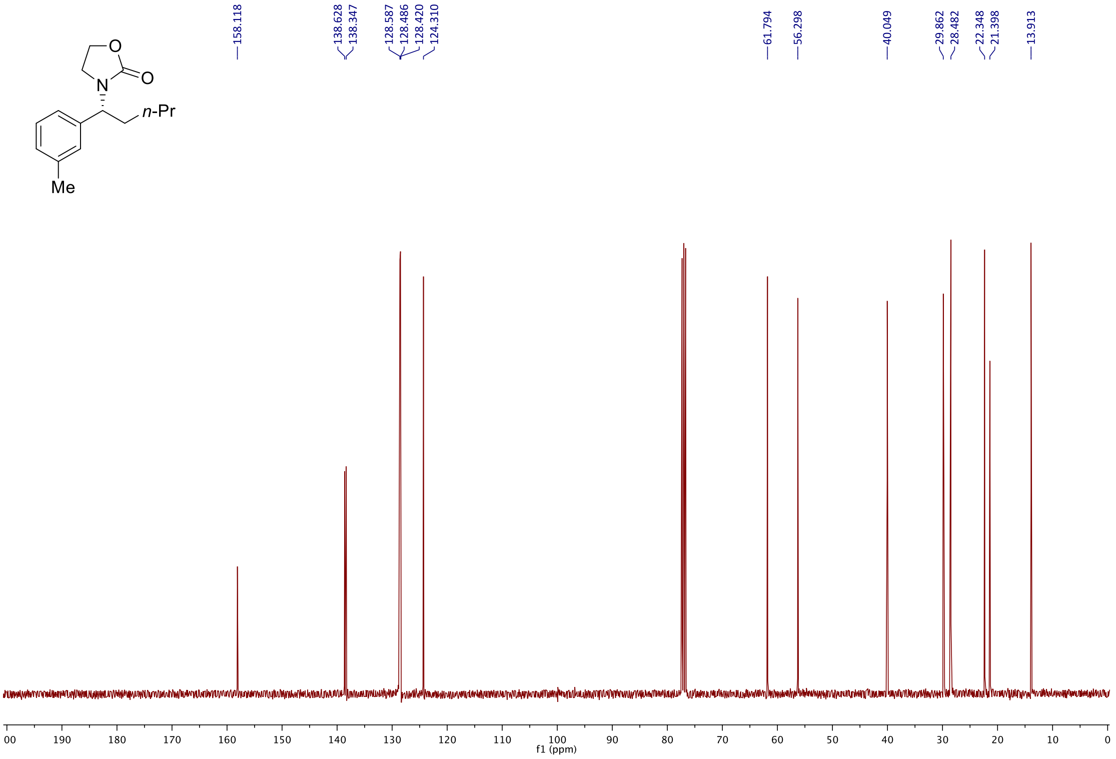

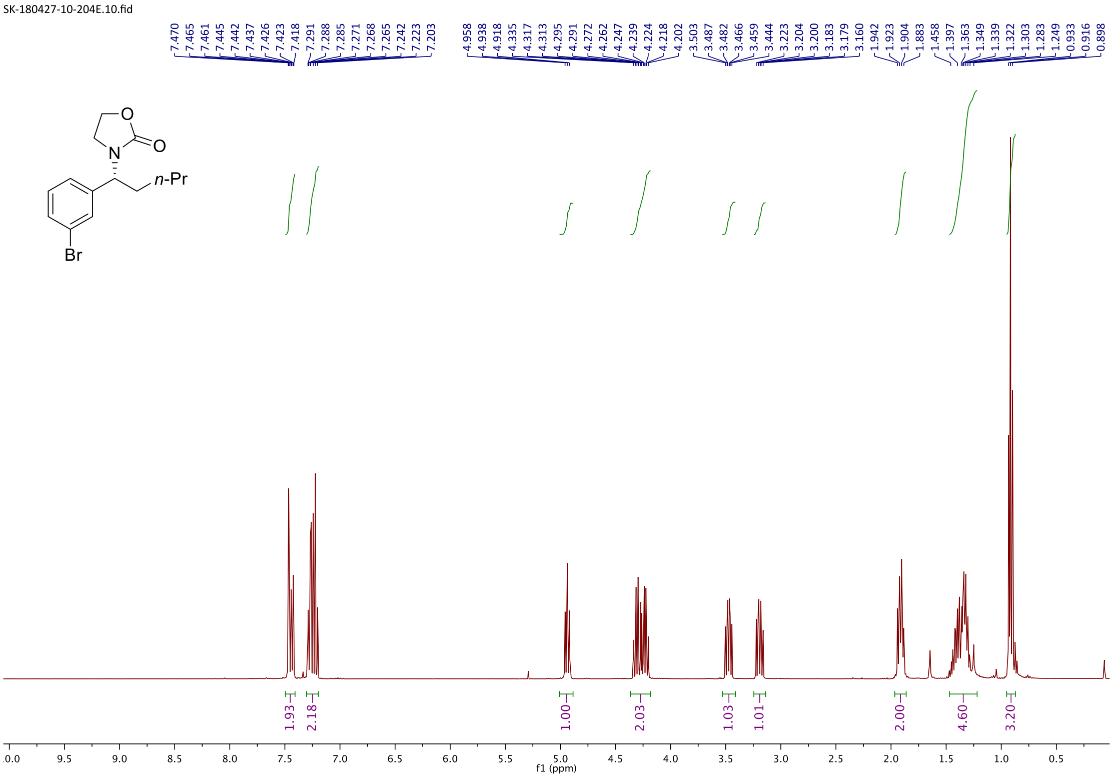
SK-180427-10-204E.10.fid
7.470
7.465
7.461
7.445
7.442
7.437
7.426
7.423
7.418
7.291
7.288
7.285
7.271
7.268
7.265
7.242
7.223
7.203
4.958
4.938
4.918
4.335
4.317
4.313
4.295
4.291
4.272
4.262
4.247
4.239
4.224
4.218
4.202
3.503
3.487
3.482
3.466
3.459
3.444
3.223
3.204
3.200
3.183
3.179
3.160
1.942
1.923
1.904
1.883
1.458
1.397
1.363
1.349
1.339
1.322
1.303
1.283
1.249
0.933
0.916
0.898
O
N
O
n-Pr
Br
1.93
2.18
1.00
2.03
1.03
1.01
2.00
4.60
3.20
f1 (ppm)

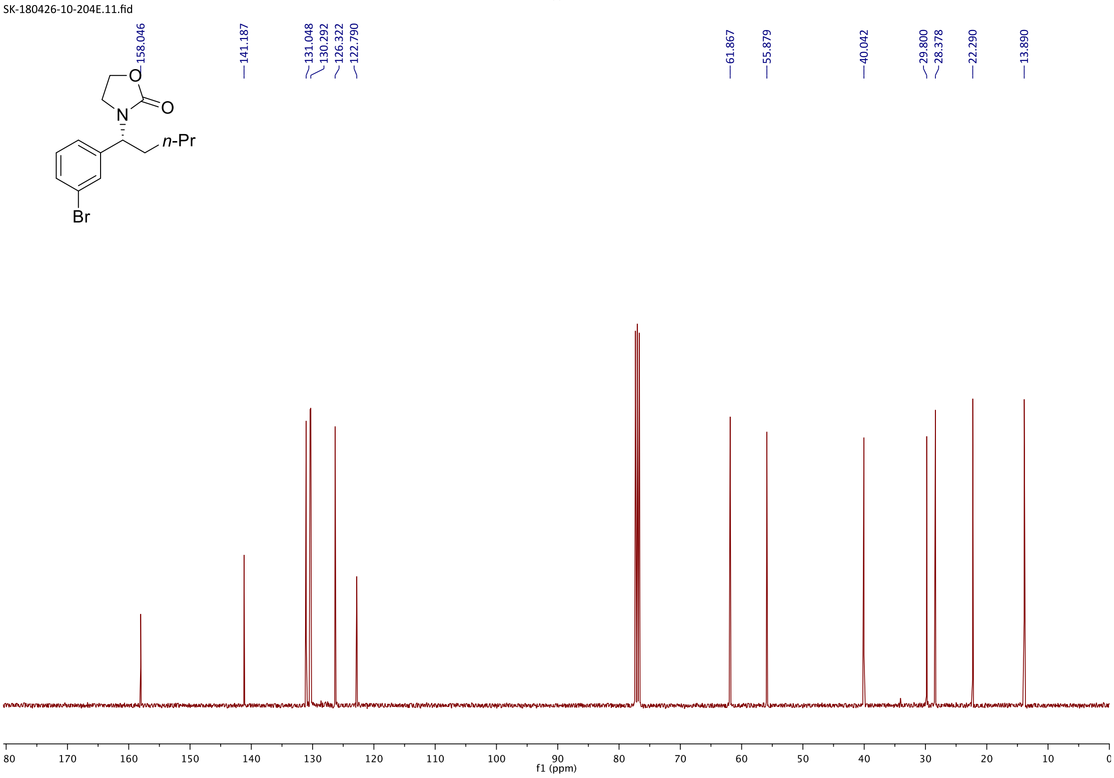
SK-180426-10-204E.11.fid
158.046
141.187
131.048
130.292
126.322
122.790
61.867
55.879
40.042
29.800
28.378
22.290
13.890
O
N
O
n-Pr
Br
f1 (ppm)

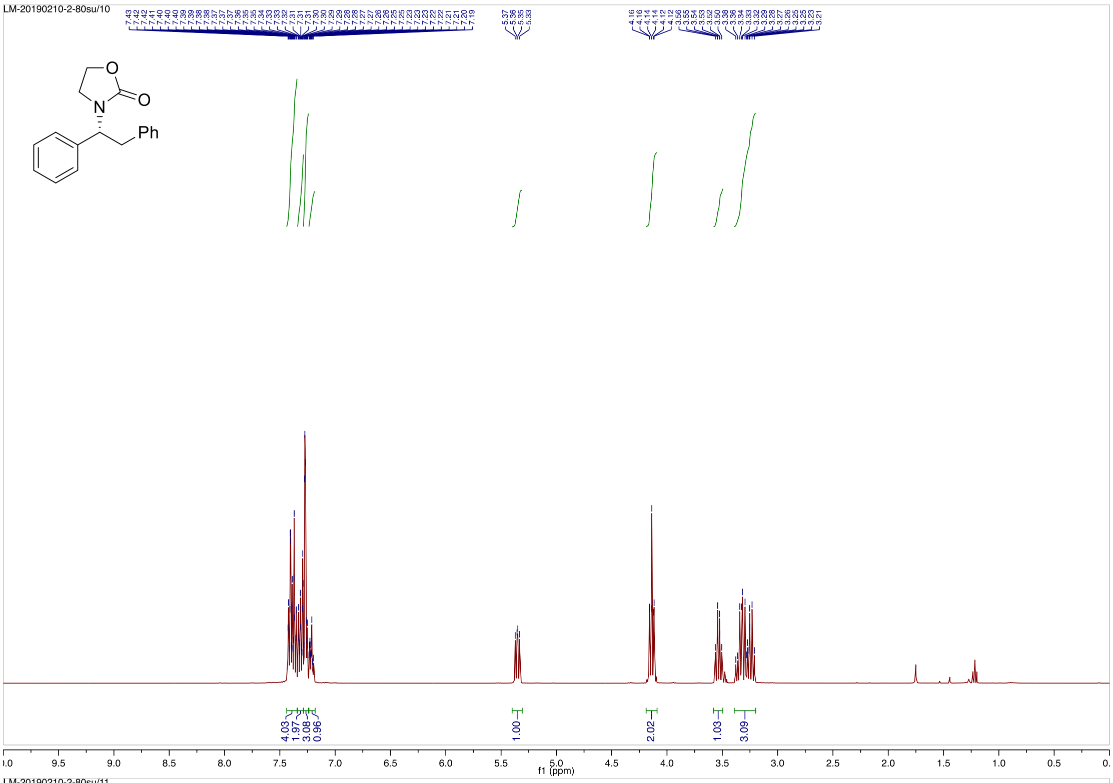
LM-20190210-2-80su/10
Ph
N
O
O
f1 (ppm)
4.03
1.97
3.08
0.96
1.00
2.02
1.03
3.09

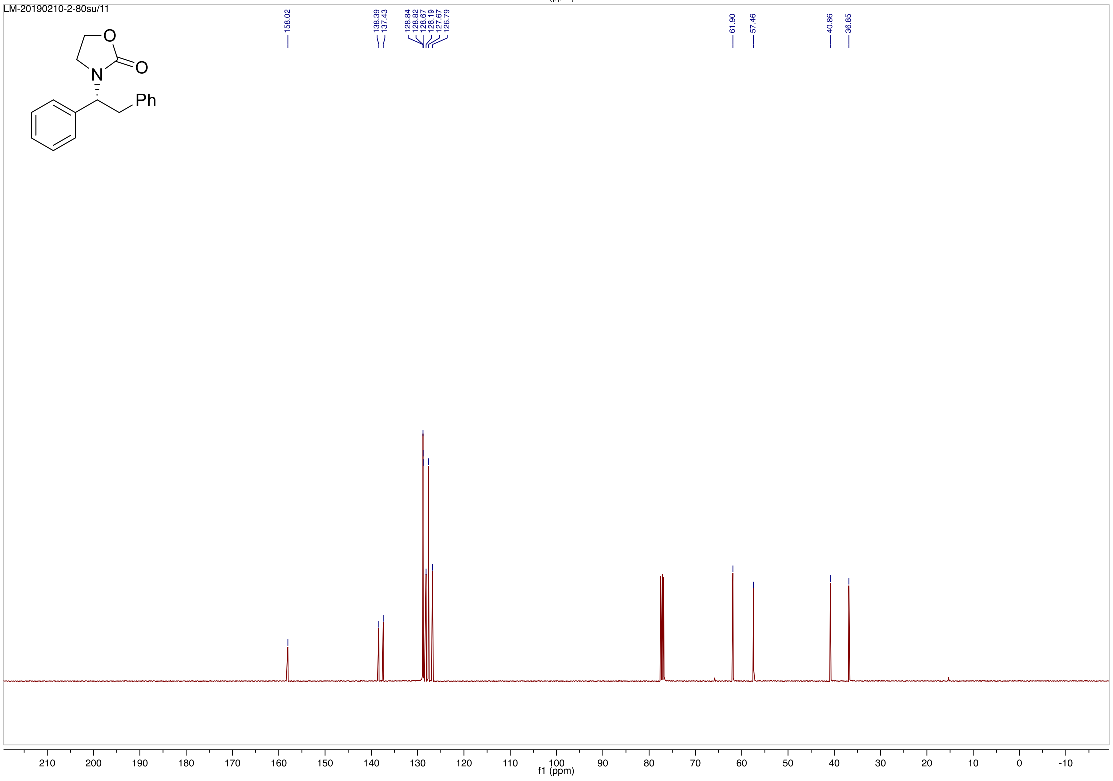
LM-20190210-2-80su/11
158.02
138.39
137.43
128.84
128.82
128.67
128.19
127.67
126.79
61.90
57.46
40.86
36.85
Ph
N
O
O
f1 (ppm)

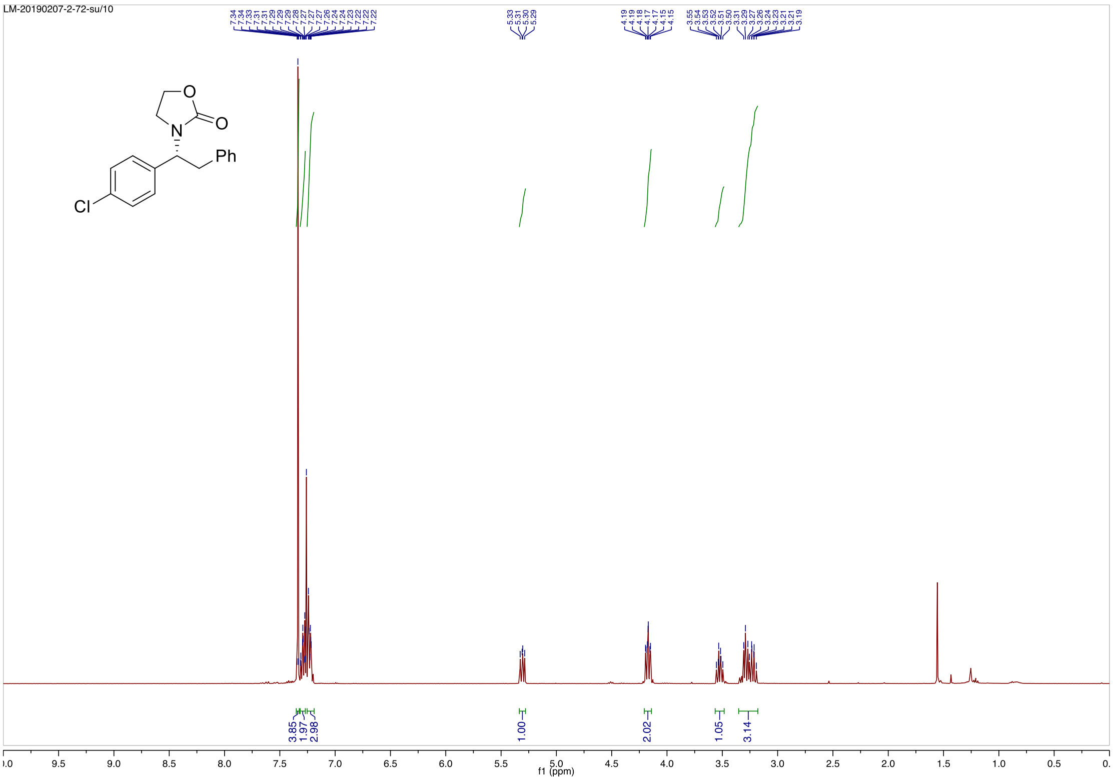

LM-20190207-2-72-su/10
O
N
O
Ph
Cl
7.34
7.34
7.33
7.31
7.29
7.29
7.28
7.27
7.27
7.26
7.24
7.24
7.23
7.22
7.22
5.33
5.31
5.30
5.29
4.19
4.19
4.18
4.17
4.17
4.15
4.15
3.55
3.54
3.53
3.52
3.51
3.50
3.31
3.29
3.27
3.26
3.24
3.23
3.21
3.19
3.85
1.97
2.98
1.00
2.02
1.05
3.14
f1 (ppm)

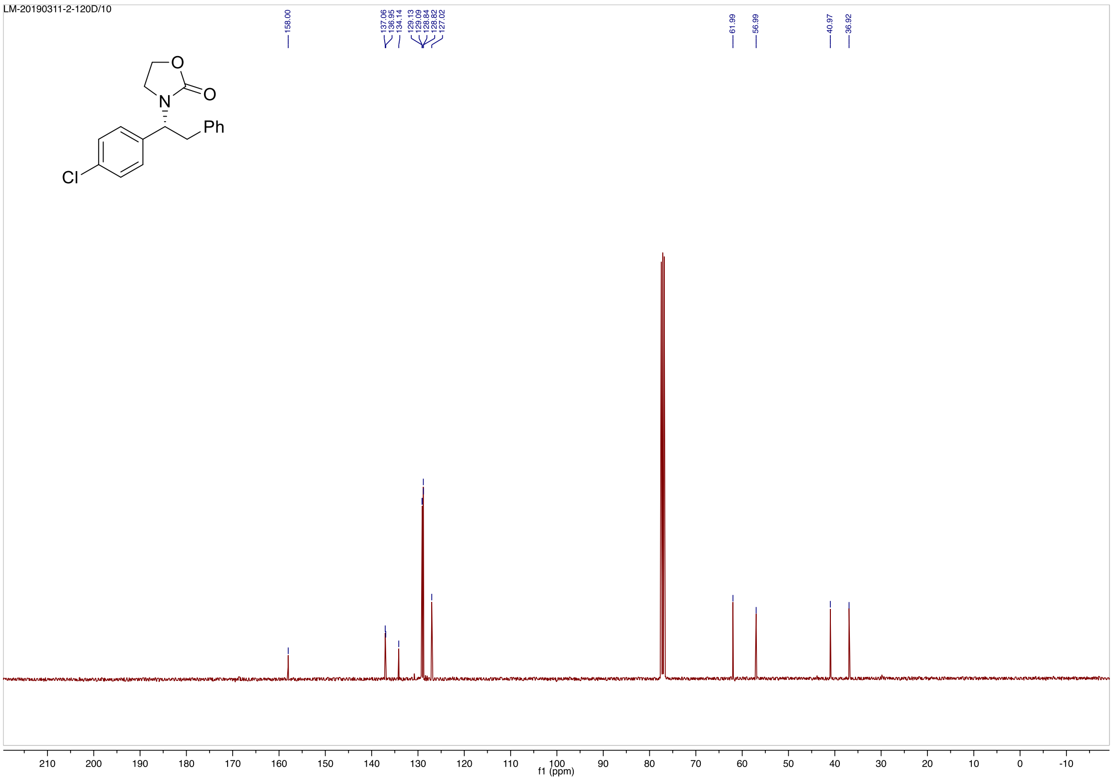

LM-20190311-2-120D/10
O
N
O
Ph
Cl
158.00
137.06
136.95
134.14
129.13
129.09
128.84
128.82
127.02
61.99
56.99
40.97
36.92
f1 (ppm)

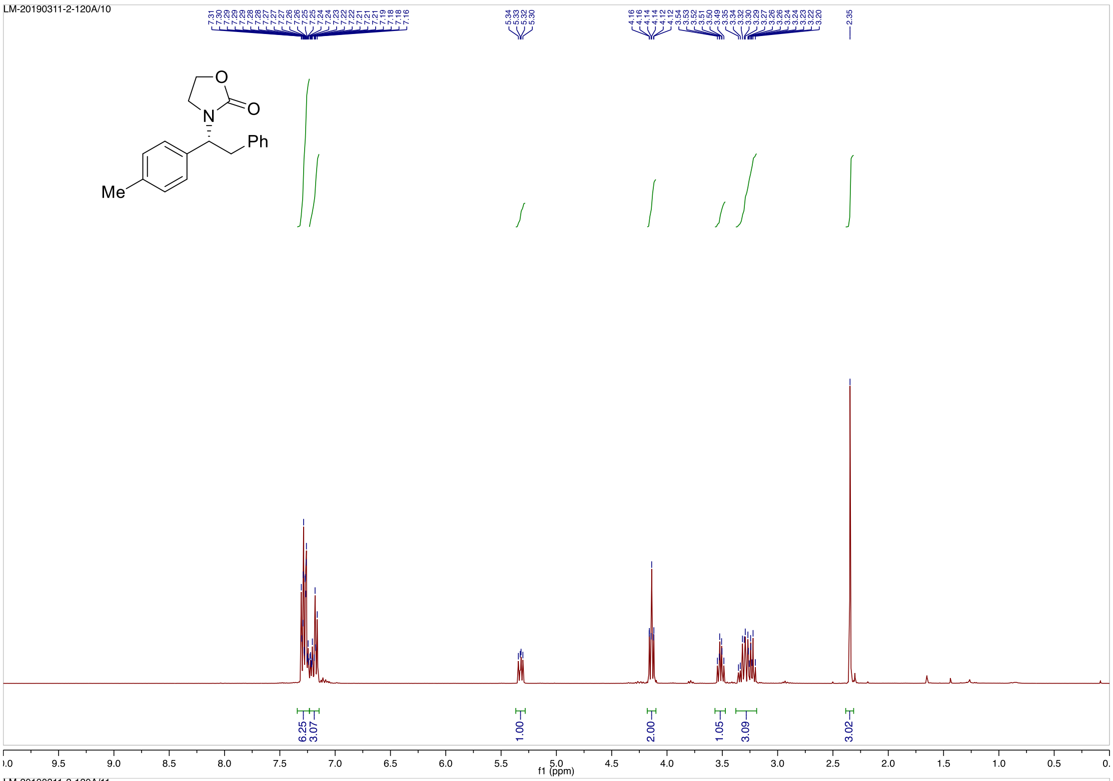
LM-20190311-2-120A/10
Me
N
O
O
Ph
f1 (ppm)

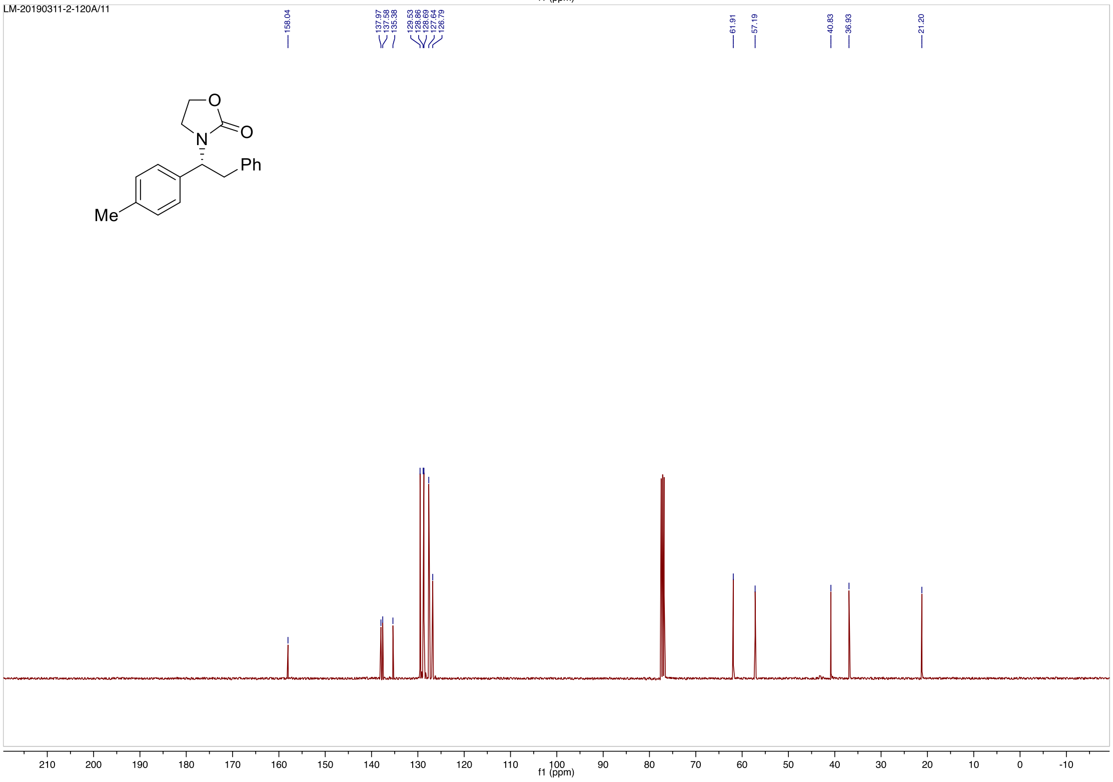
LM-20190311-2-120A/11
Me
N
O
O
Ph
158.04
137.97
137.58
135.38
129.53
128.86
128.69
127.64
126.79
61.91
57.19
40.83
36.93
21.20
f1 (ppm)

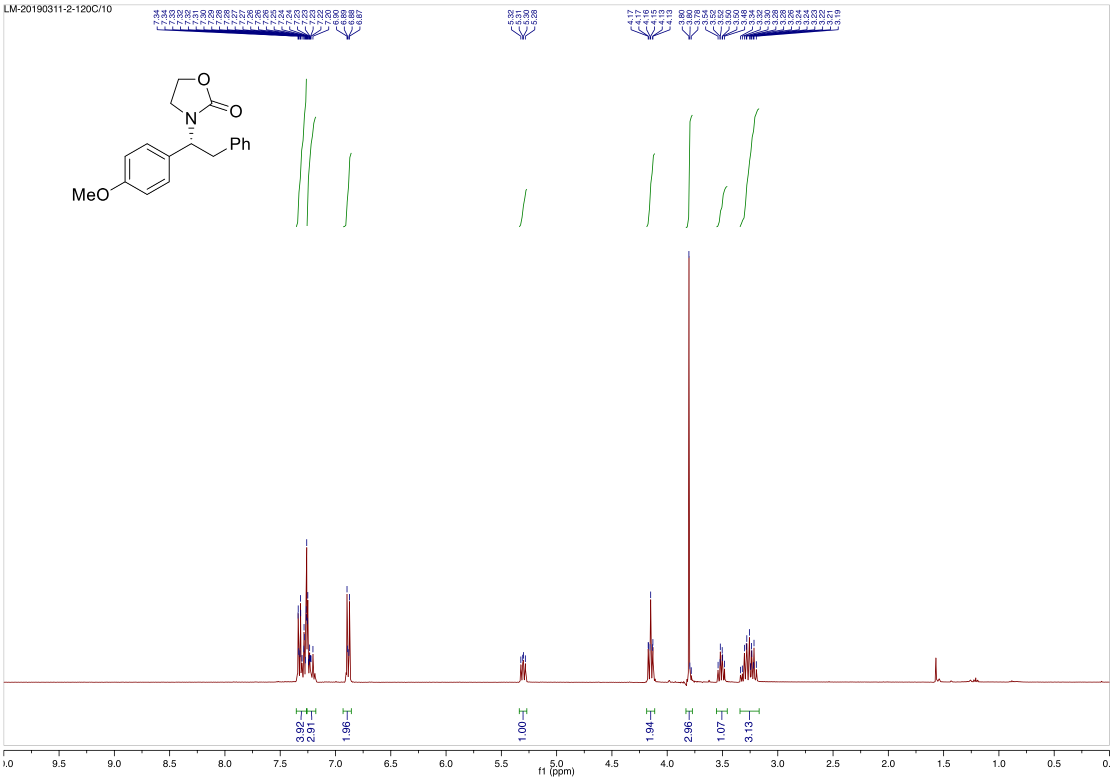
LM-20190311-2-120C/10
MeO
Ph
f1 (ppm)

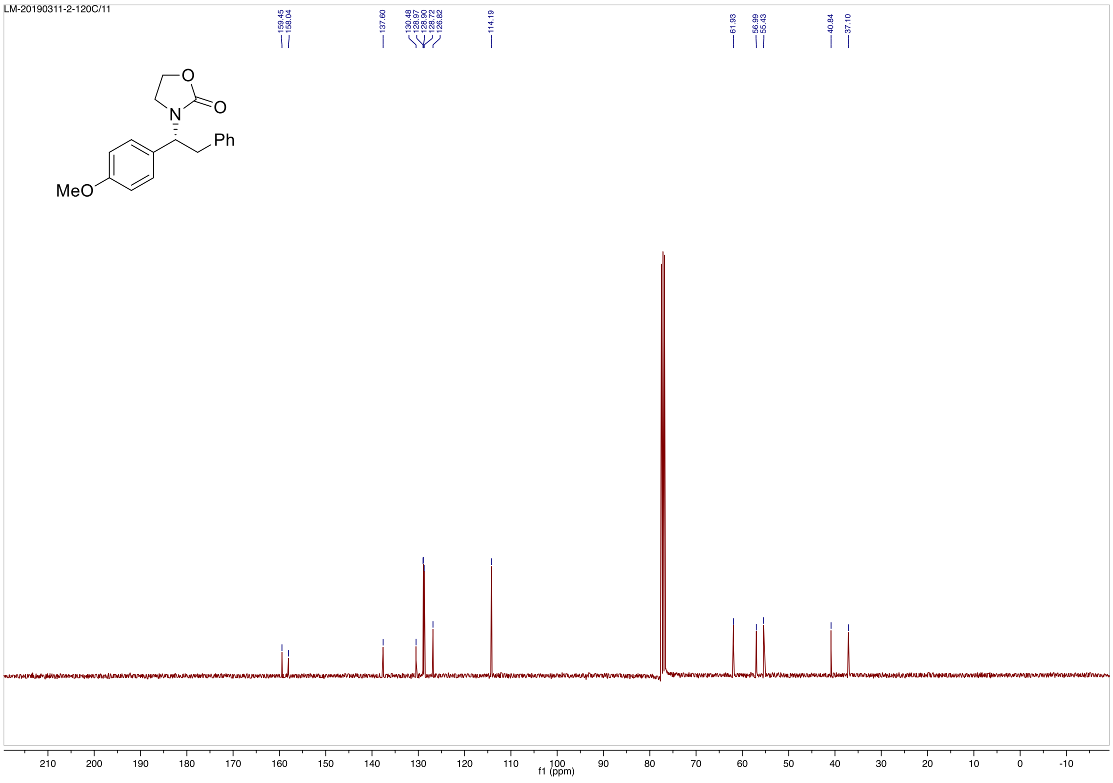
LM-20190311-2-120C/11
159.45
158.04
137.60
130.48
128.97
128.90
128.72
126.82
114.19
61.93
56.99
55.43
40.84
37.10
MeO
Ph
f1 (ppm)

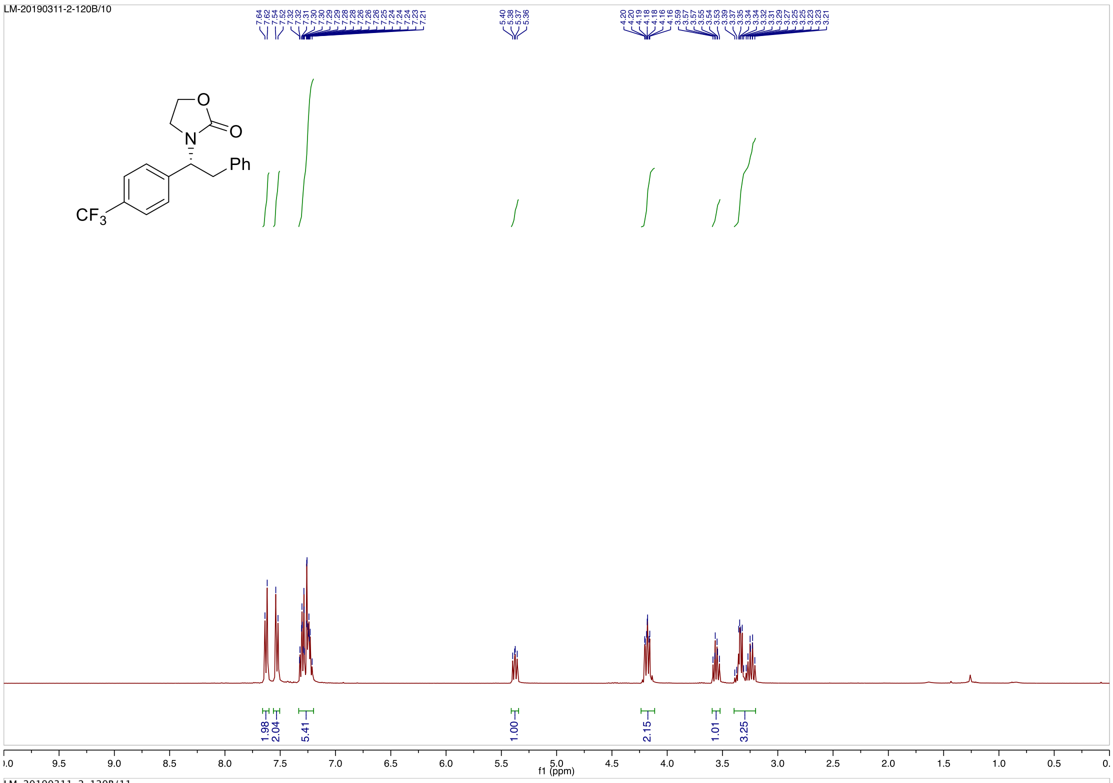

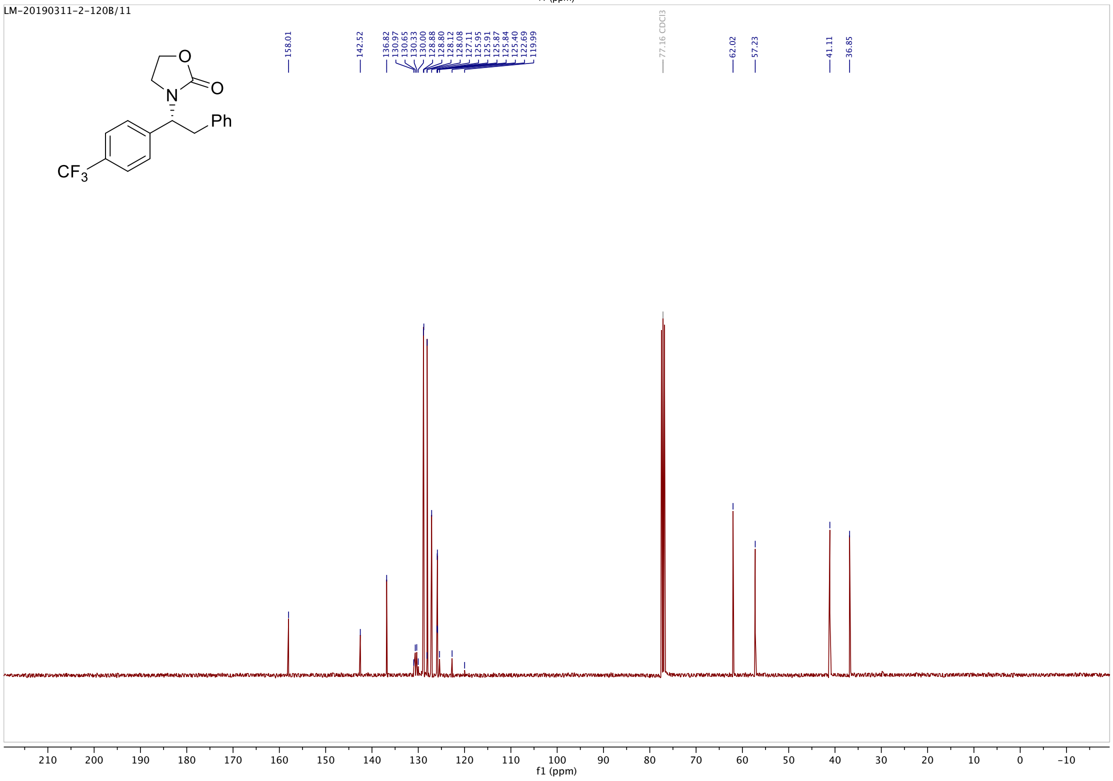

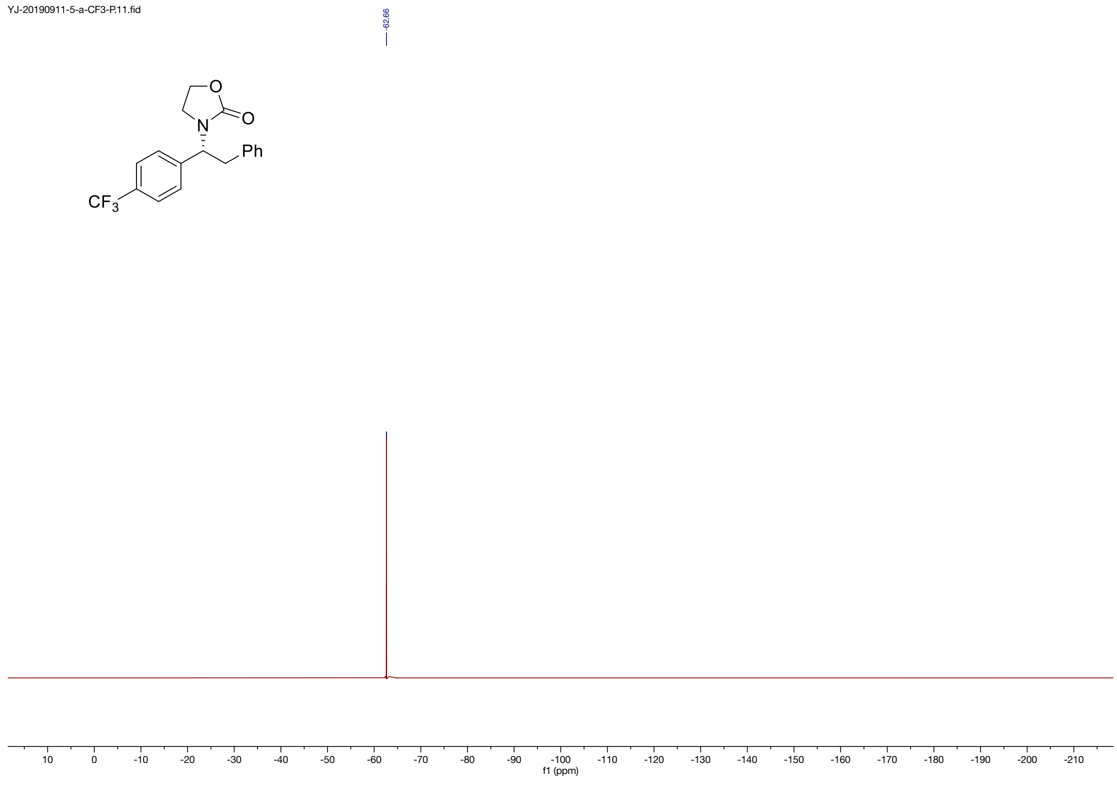
YJ-20190911-5-a-CF3-P.11.fid
-62.66
O
N
O
Ph
CF3
10
0
-10
-20
-30
-40
-50
-60
-70
-80
-90
-100
-110
-120
-130
-140
-150
-160
-170
-180
-190
-200
-210
f1 (ppm)

YJ-20190511-5-3-1-p-pure-1.10.fid

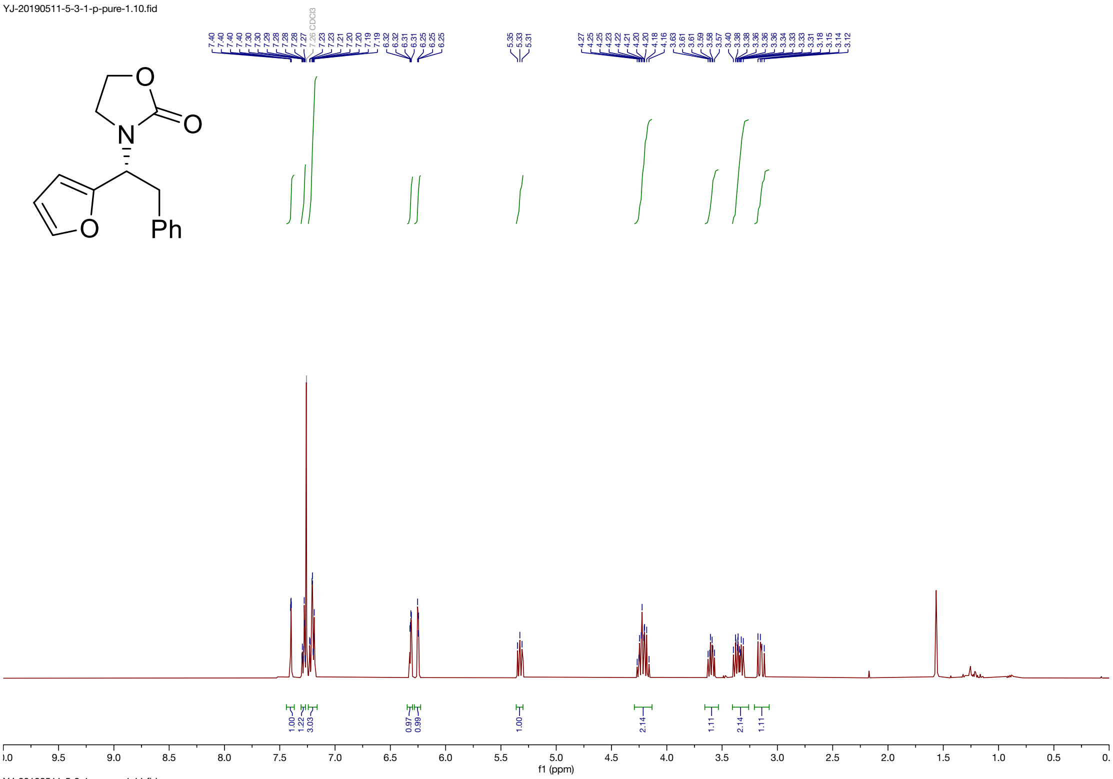

YJ-20190511-5-3-1-p-pure-1.11.fid

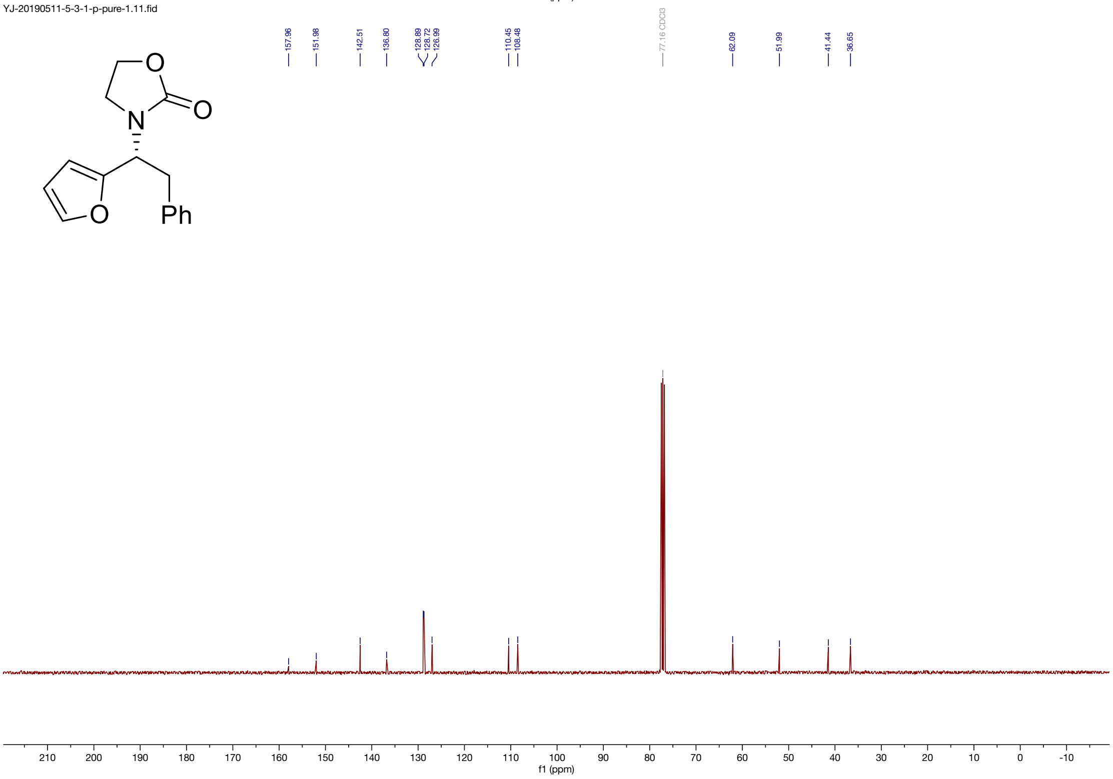

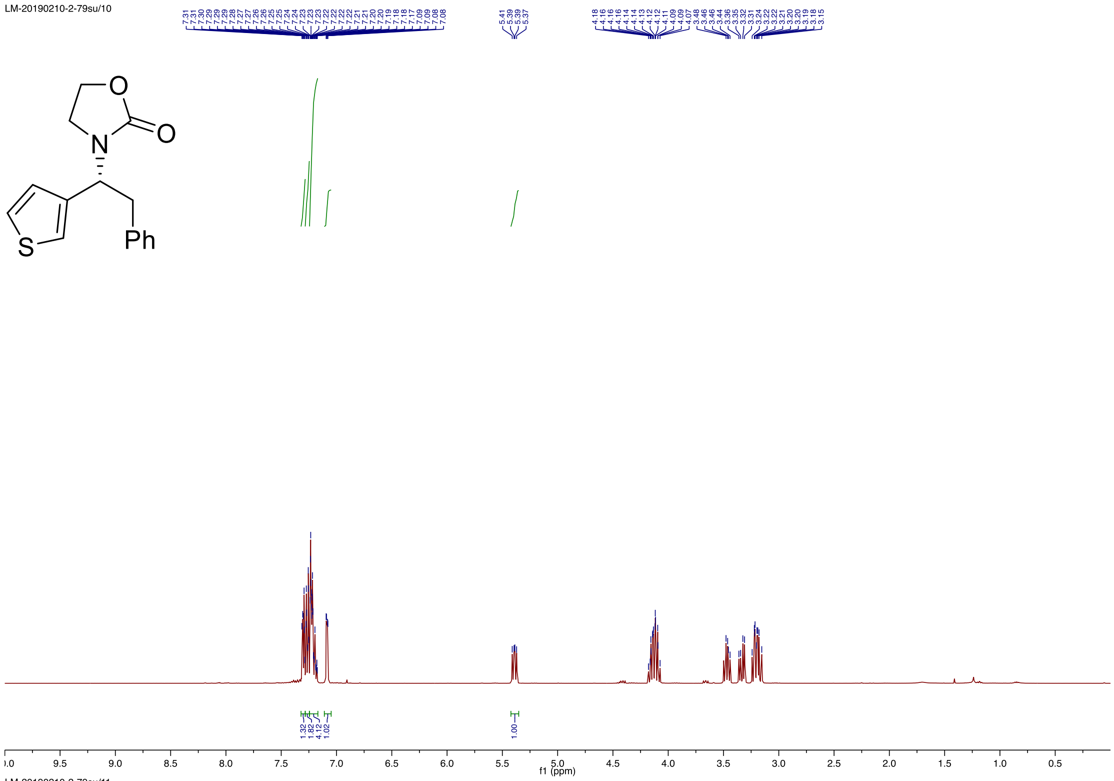
LM-20190210-2-79su/10
O
O
N
S
Ph
1.32
1.82
4.12
1.02
1.00
f1 (ppm)

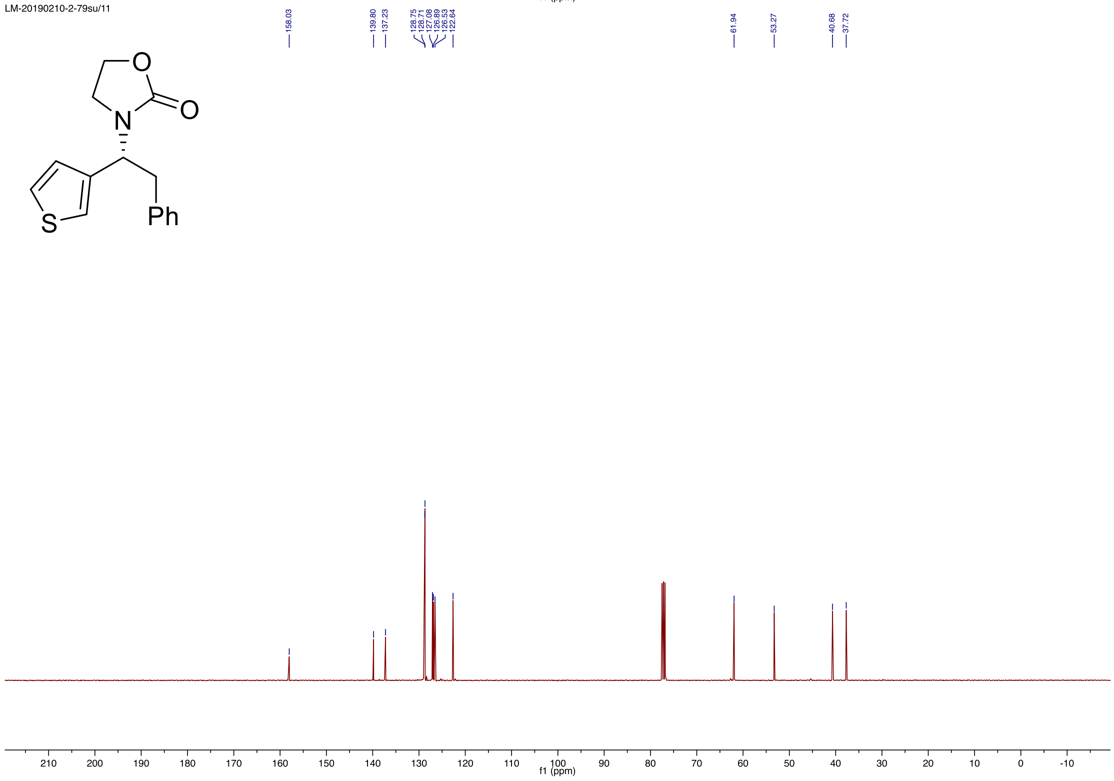
LM-20190210-2-79su/11
158.03
139.80
137.23
128.75
128.71
127.08
126.89
126.53
122.64
61.94
53.27
40.68
37.72
O
O
N
S
Ph
f1 (ppm)

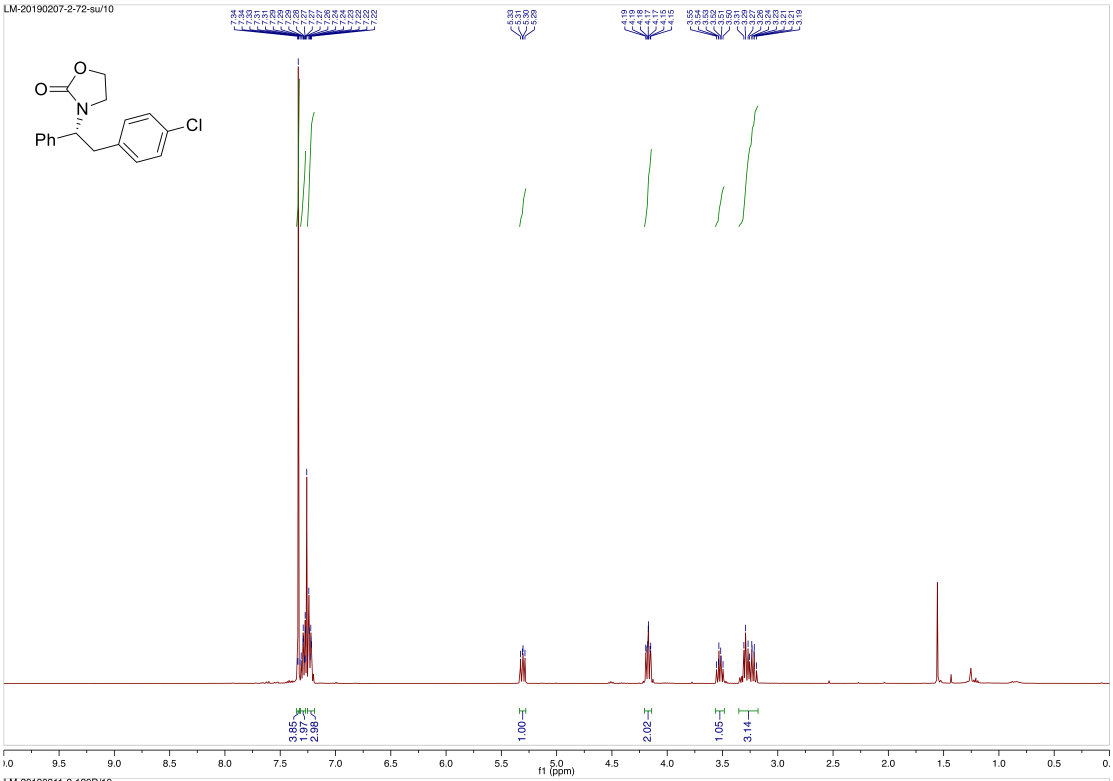

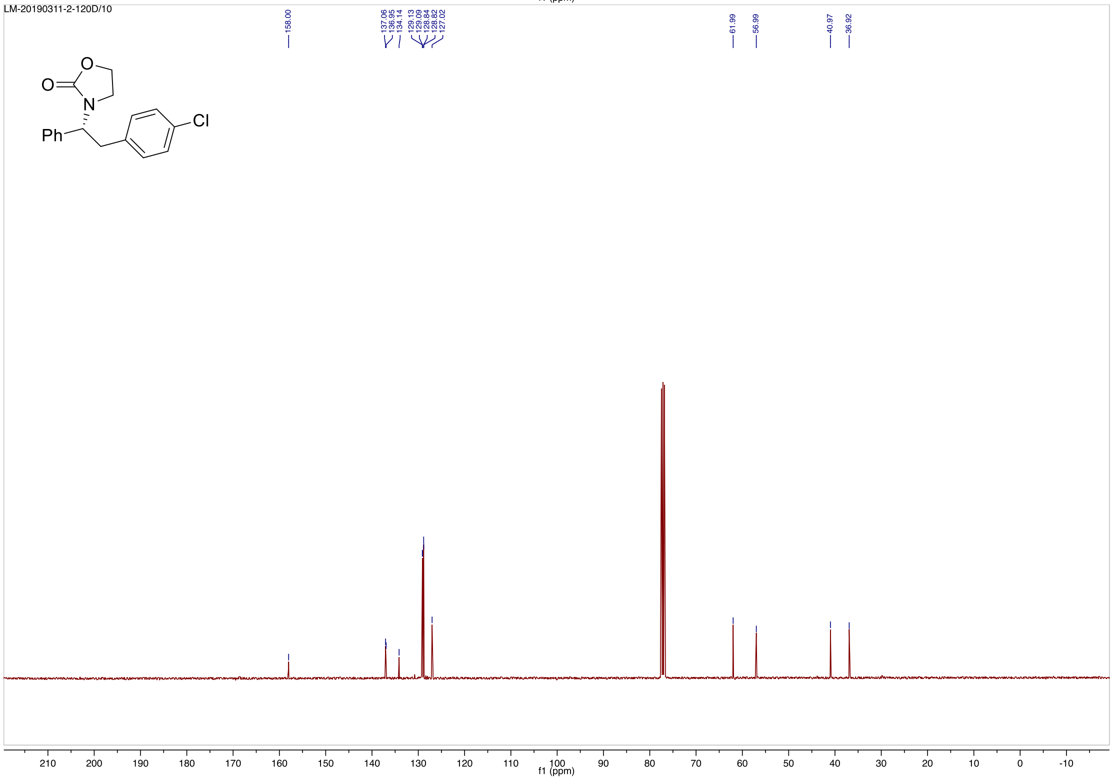

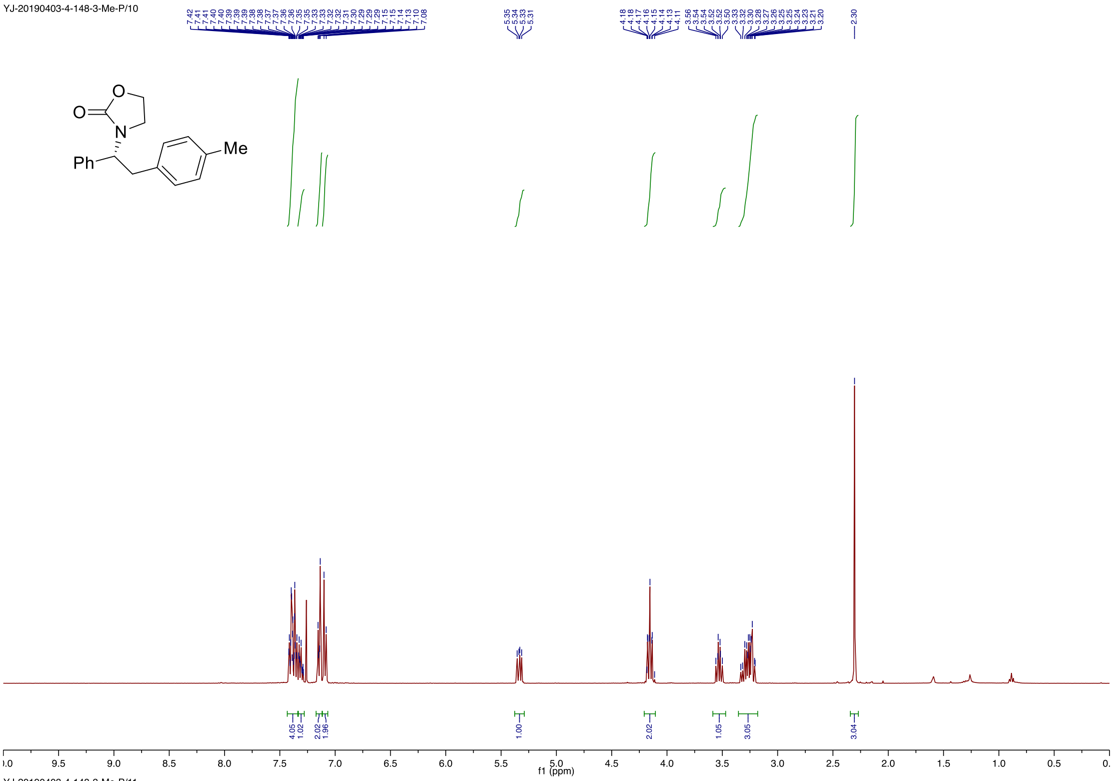
YJ-20190403-4-148-3-Me-P/10
Ph
Me
f1 (ppm)

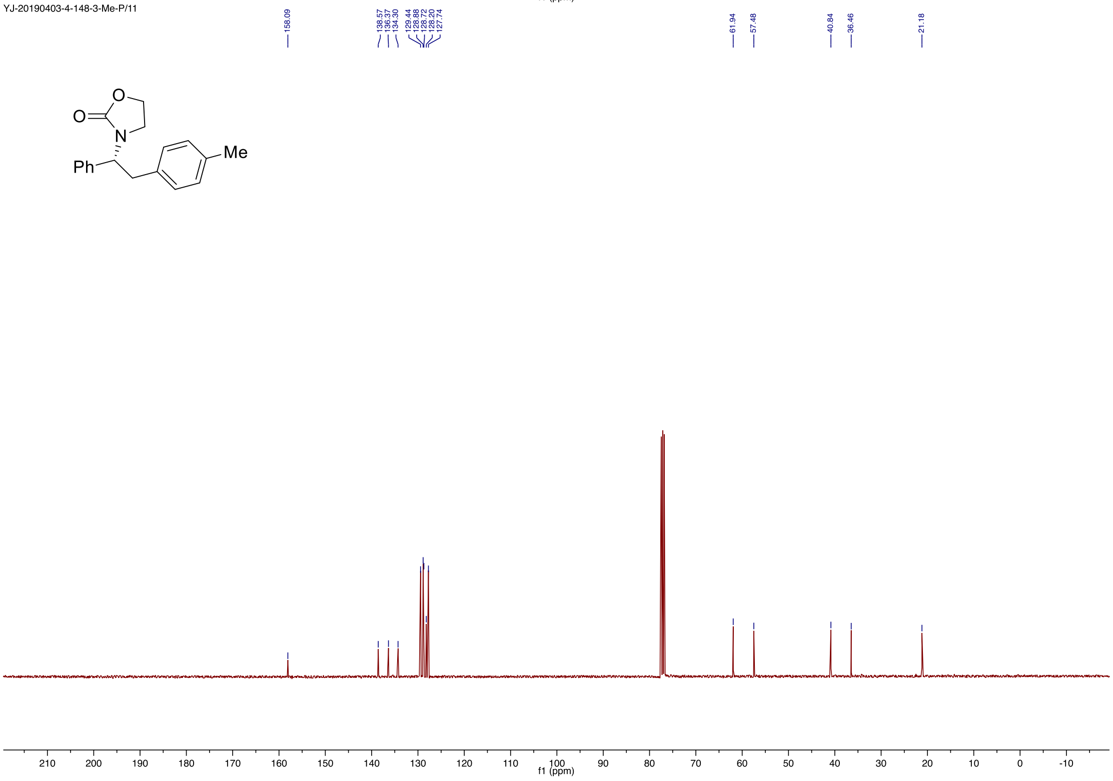
YJ-20190403-4-148-3-Me-P/11
Ph
Me
f1 (ppm)

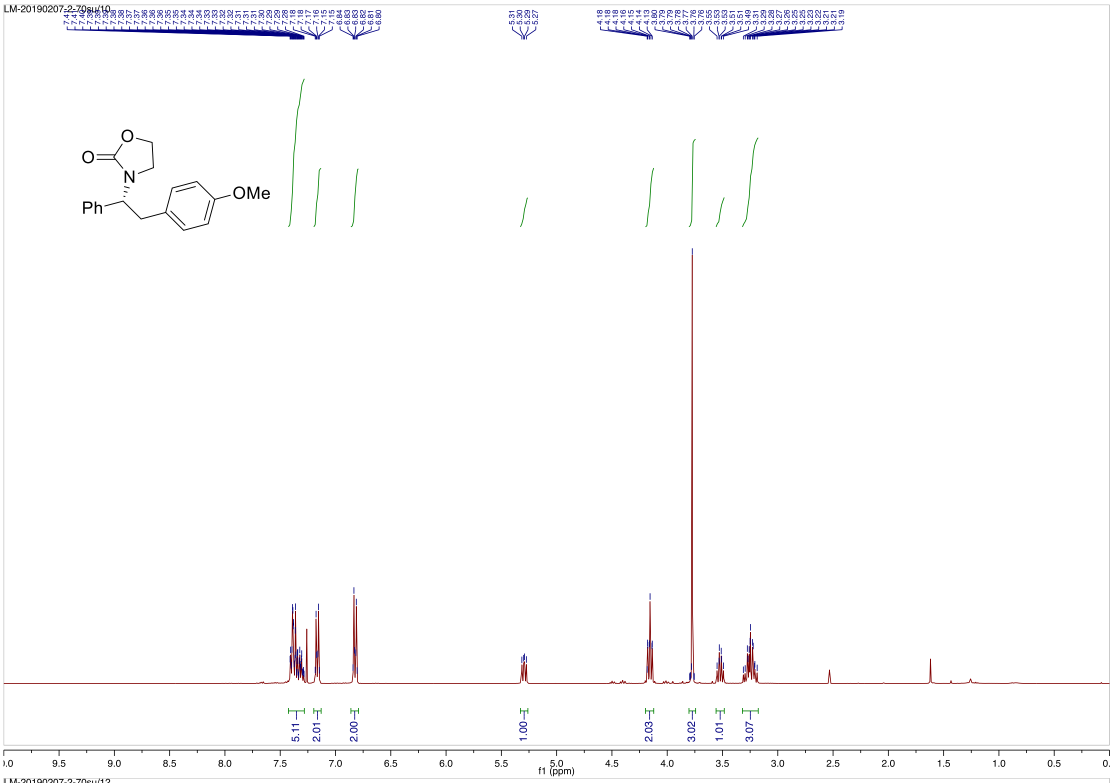

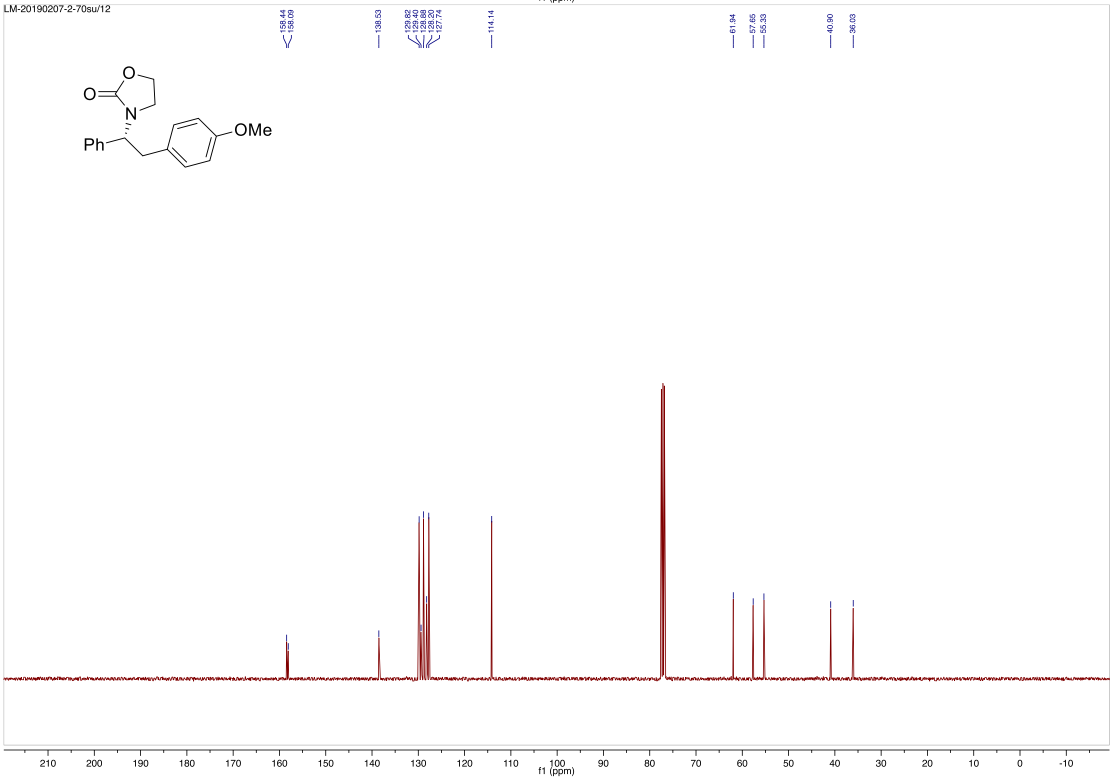

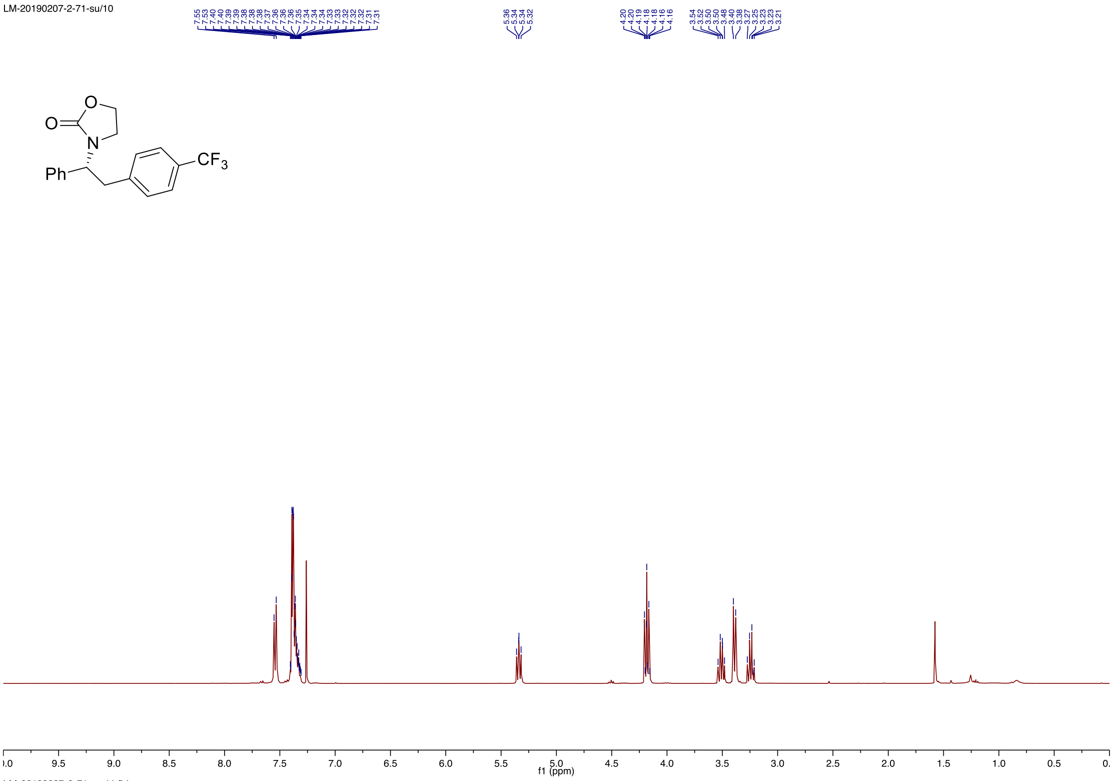
LM-20190207-2-71-su/10
O
N
Ph
CF3
f1 (ppm)

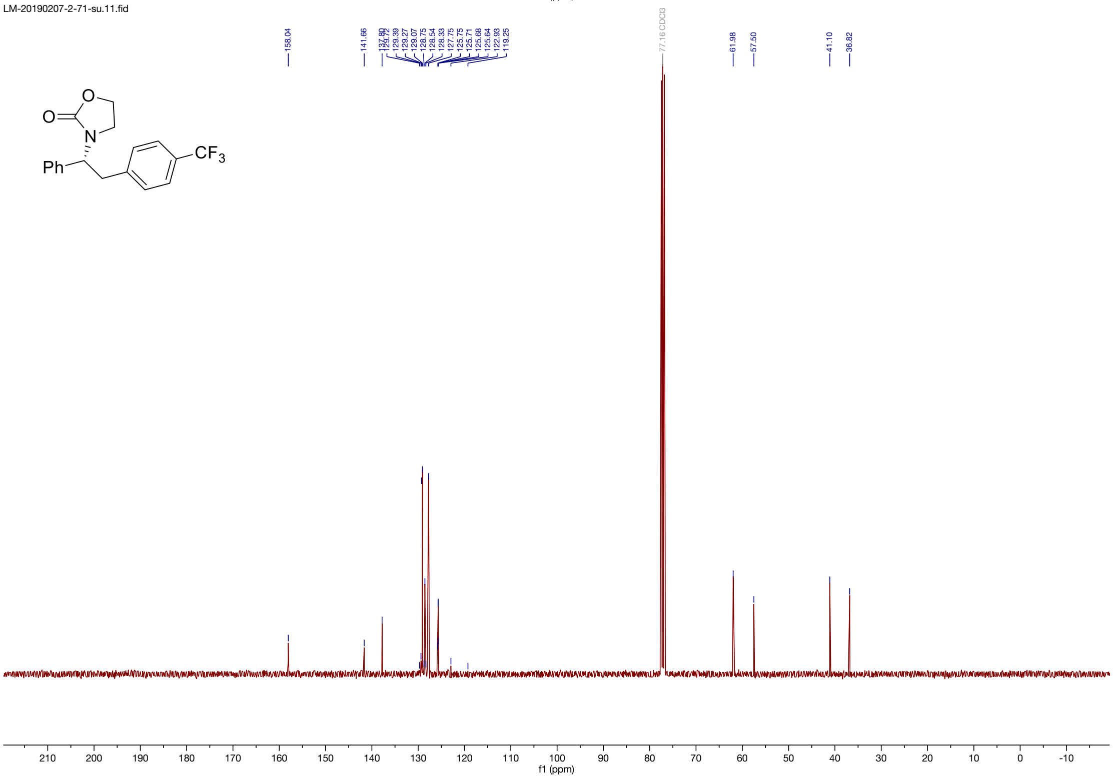
LM-20190207-2-71-su.11.fid
158.04
141.66
77.16 CDCl3
61.98
57.50
41.10
36.82
O
N
Ph
CF3
f1 (ppm)

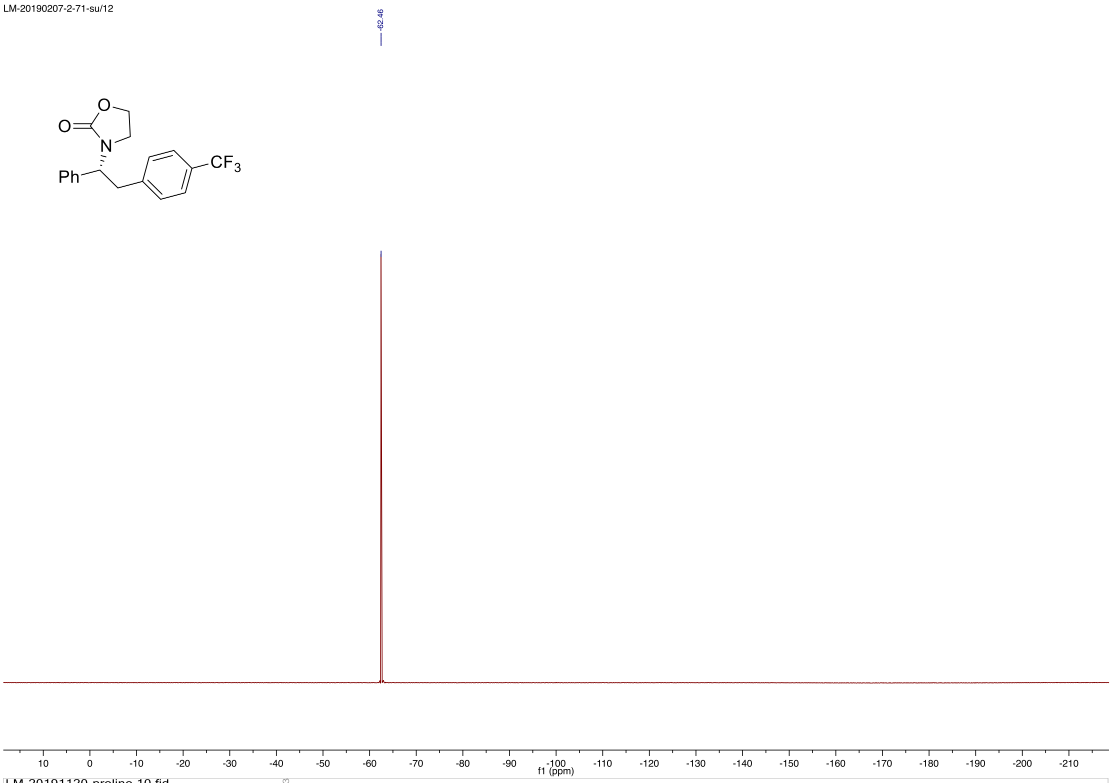
LM-20190207-2-71-su/12
-62.46
O
N
O
Ph
CF_3
f1 (ppm)

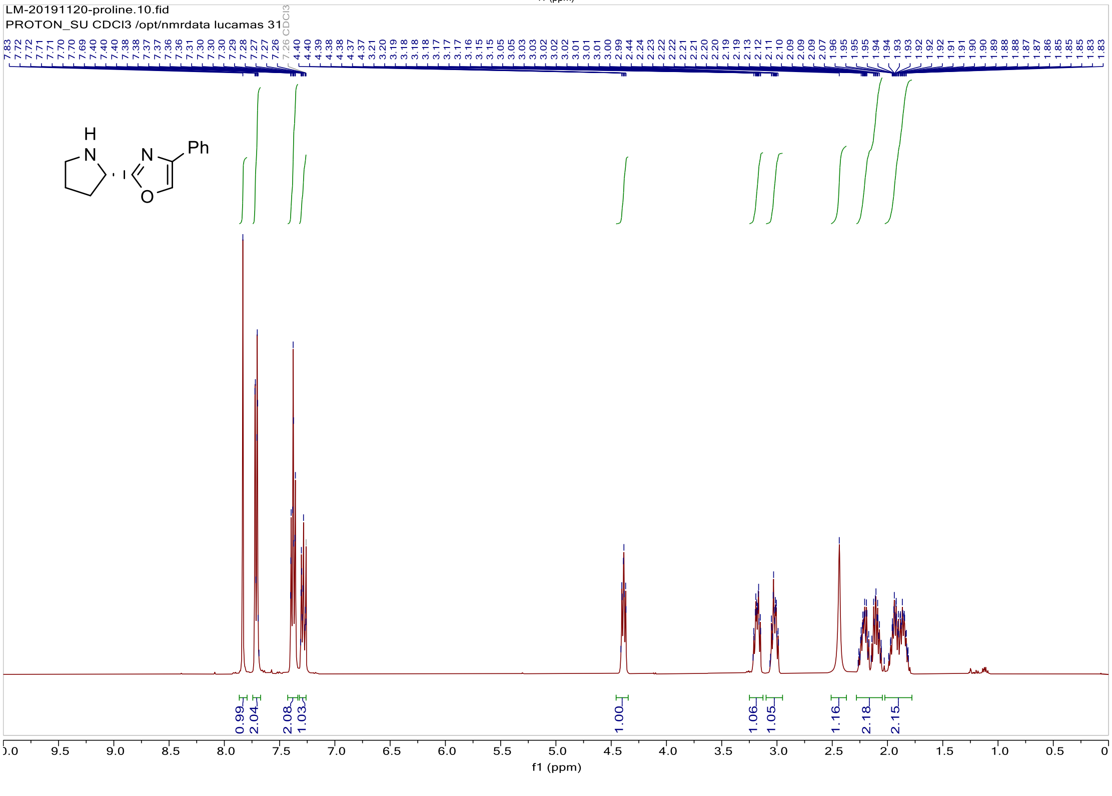
LM-20191120-proline.10.fid
PROTON_SU CDCl3 /opt/nmrdata lucamas 31
CDCl3
H
N
N
O
Ph
0.99
2.04
2.08
1.03
1.00
1.06
1.05
1.16
2.18
2.15
f1 (ppm)

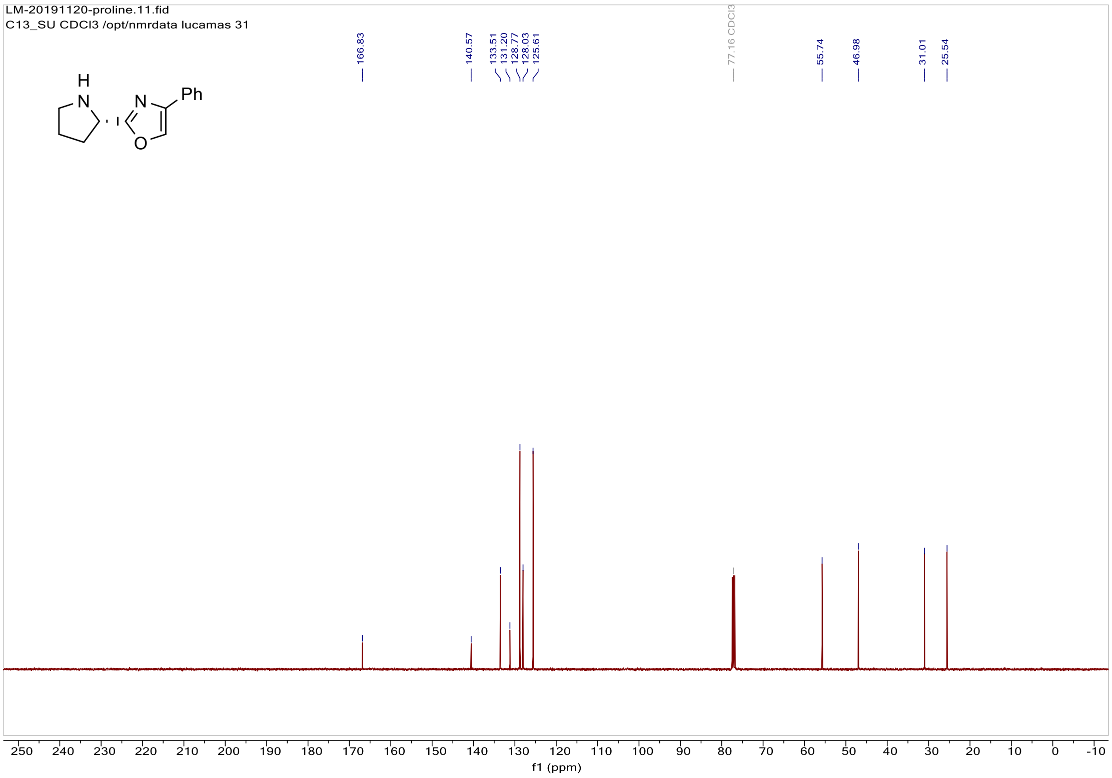

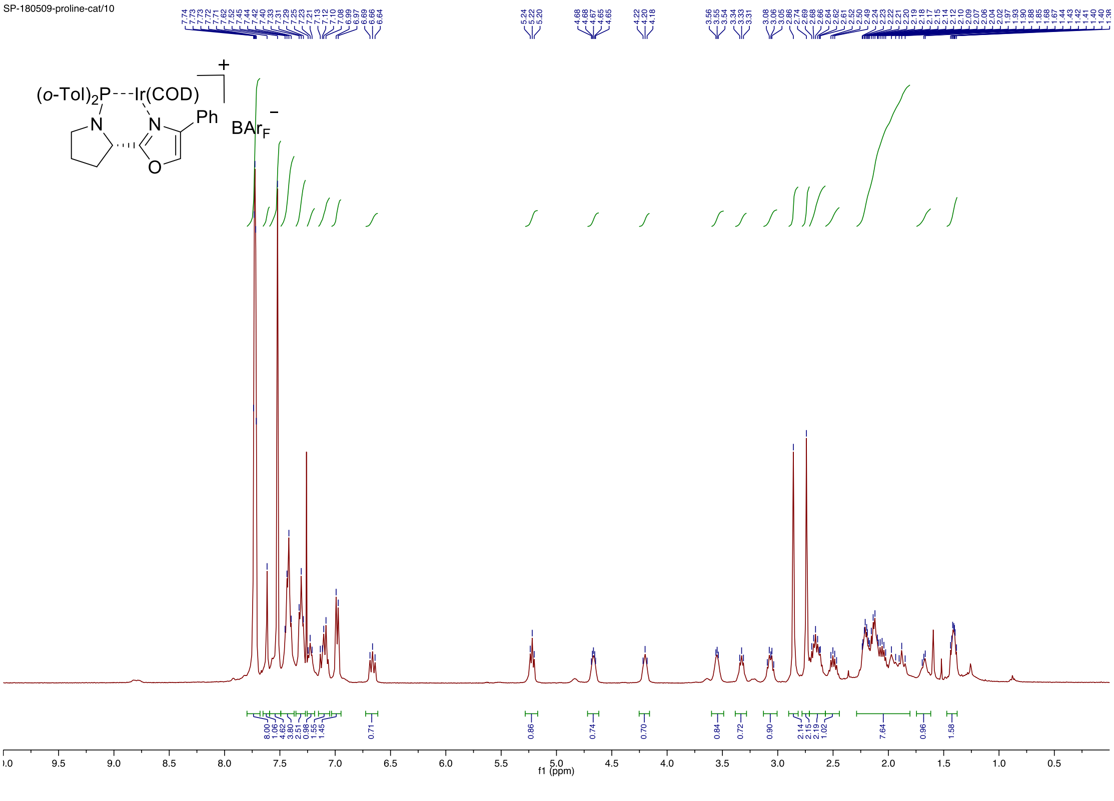

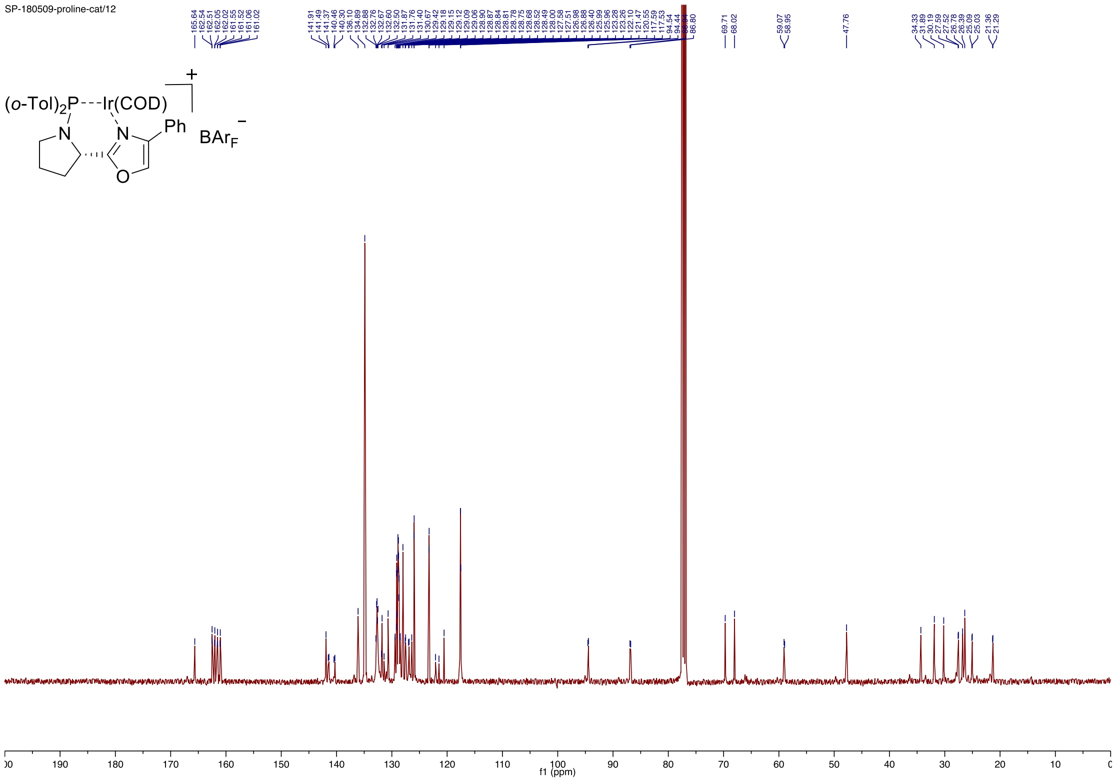
SP-180509-proline-cat/12
(o-Tol)2P---Ir(COD)
N
N
Ph
O
BArF
f1 (ppm)

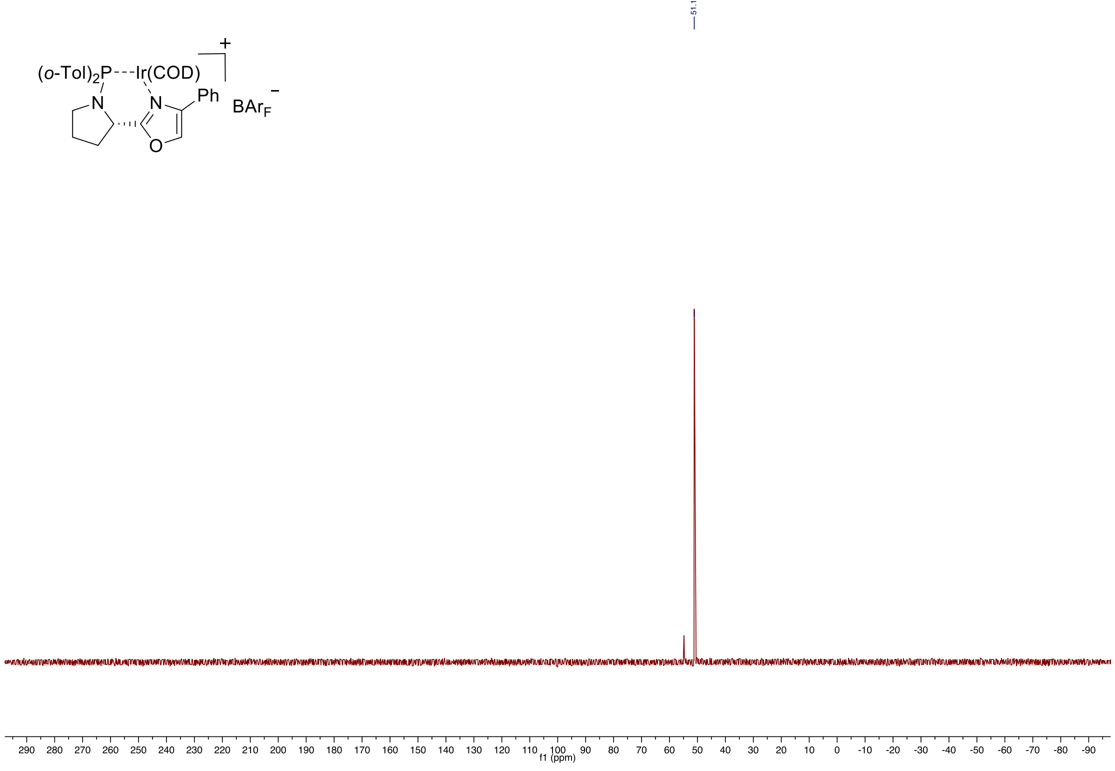
SP-180509-proline-cat/11
51.14
(o-Tol)2P---Ir(COD)
N
N
Ph
O
BArF
f1 (ppm)

11. Chromatogram of chiral compounds

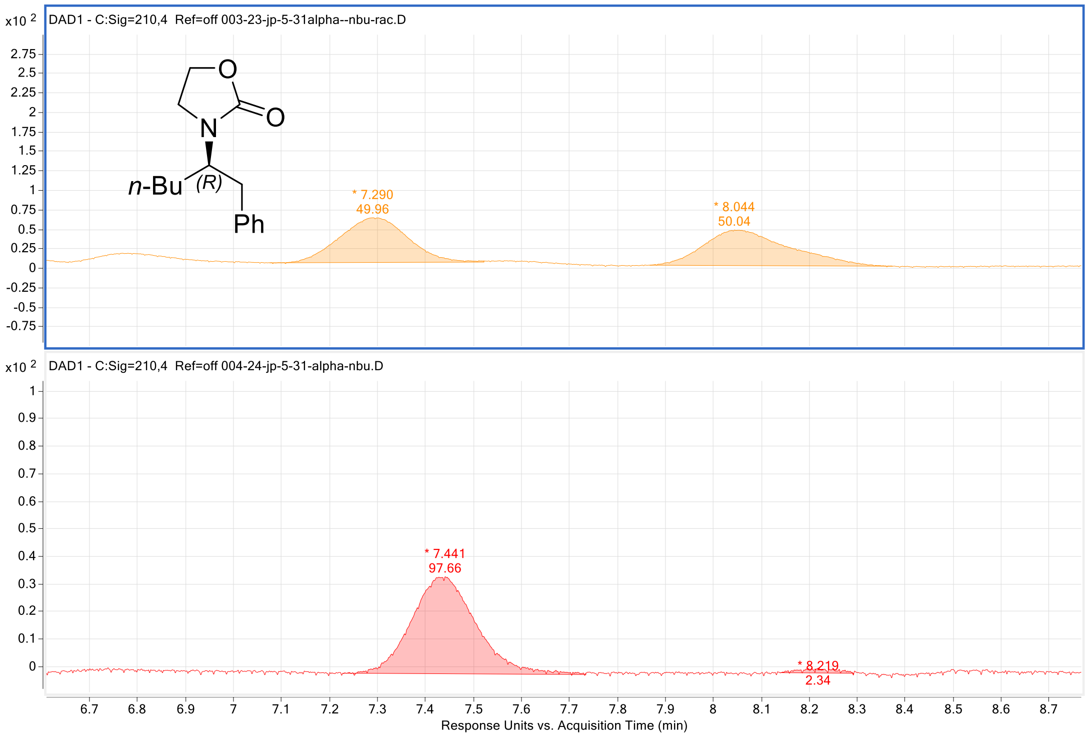

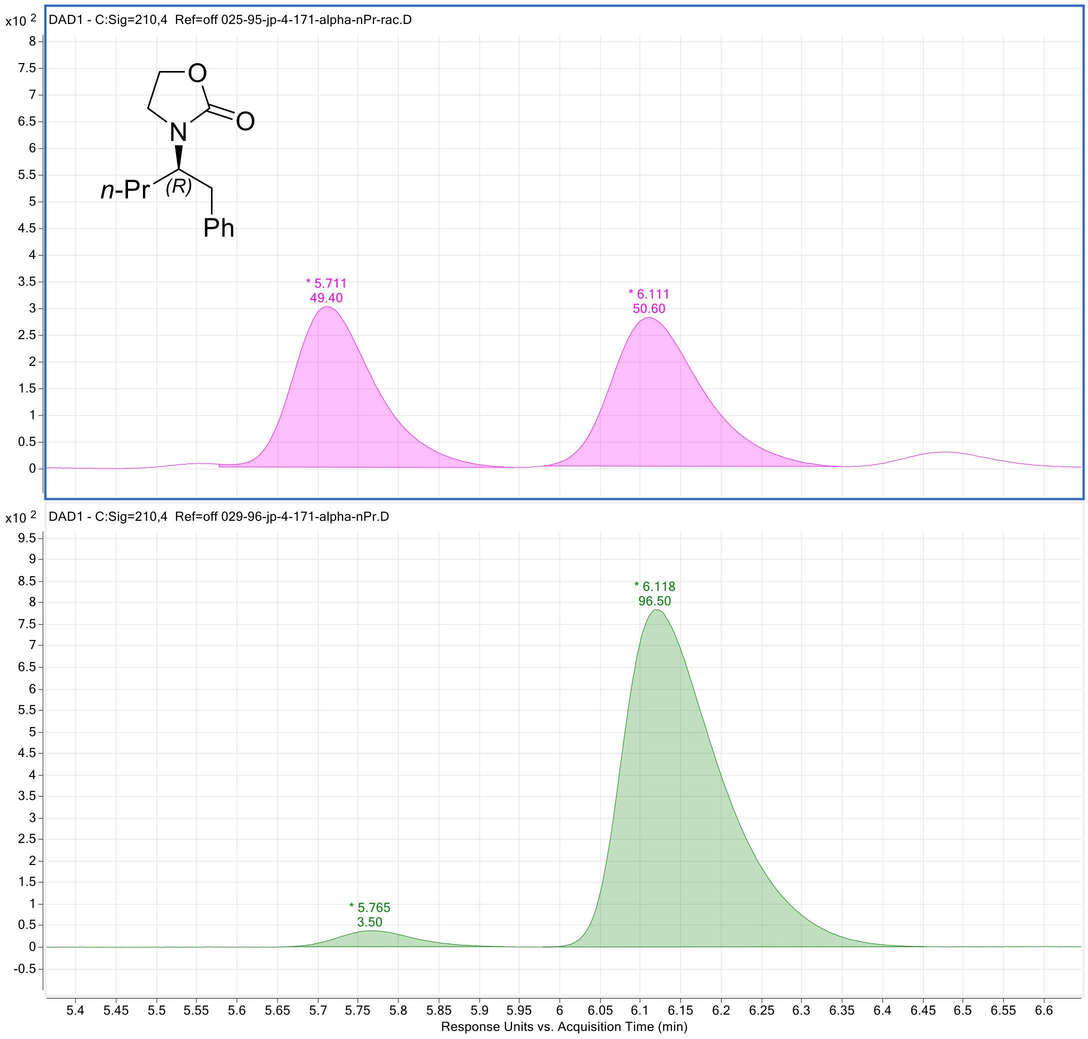

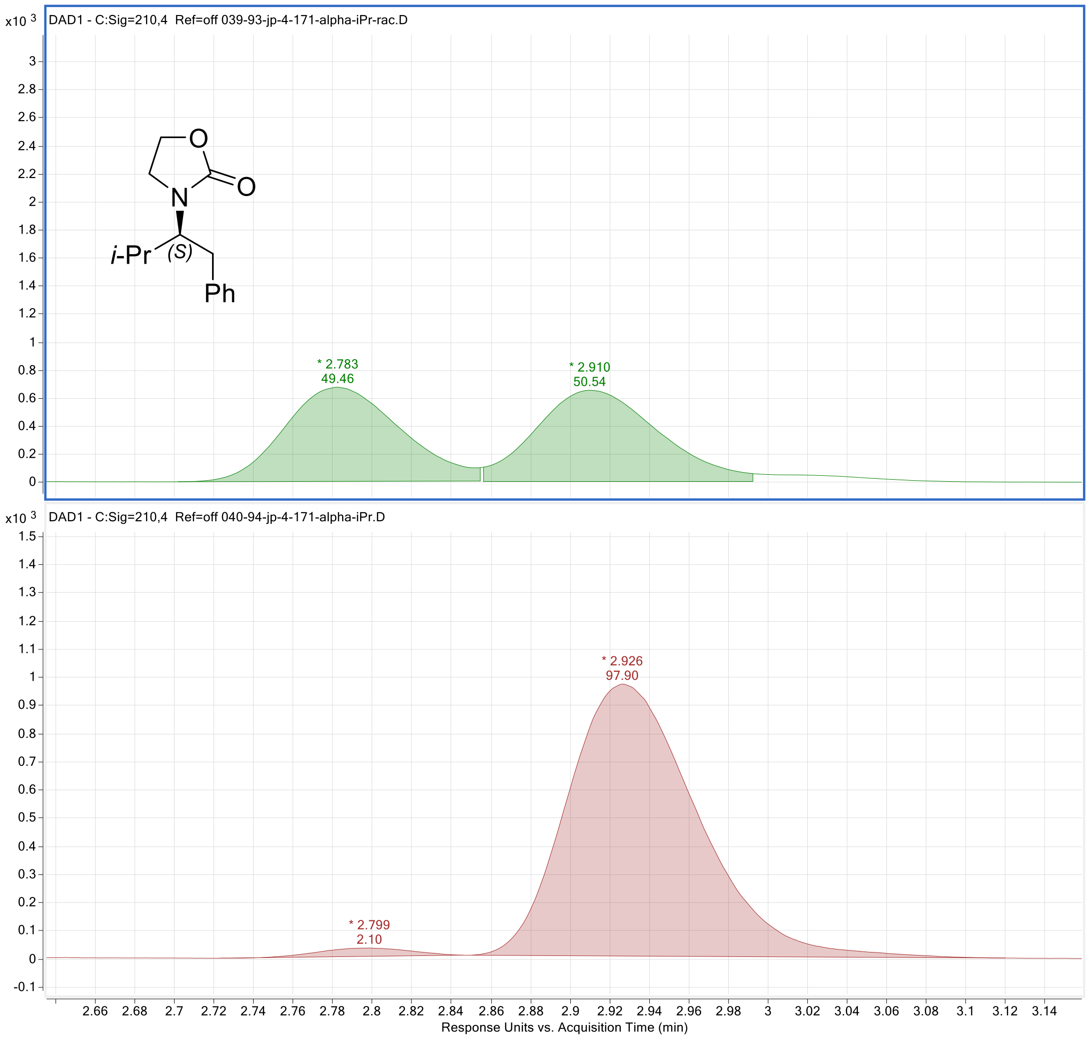
DAD1 - C:Sig=210,4 Ref=off 039-93-jp-4-171-alpha-iPr-rac.D
i-Pr (S) Ph
* 2.783
49.46
* 2.910
50.54
DAD1 - C:Sig=210,4 Ref=off 040-94-jp-4-171-alpha-iPr.D
* 2.799
2.10
* 2.926
97.90
Response Units vs. Acquisition Time (min)

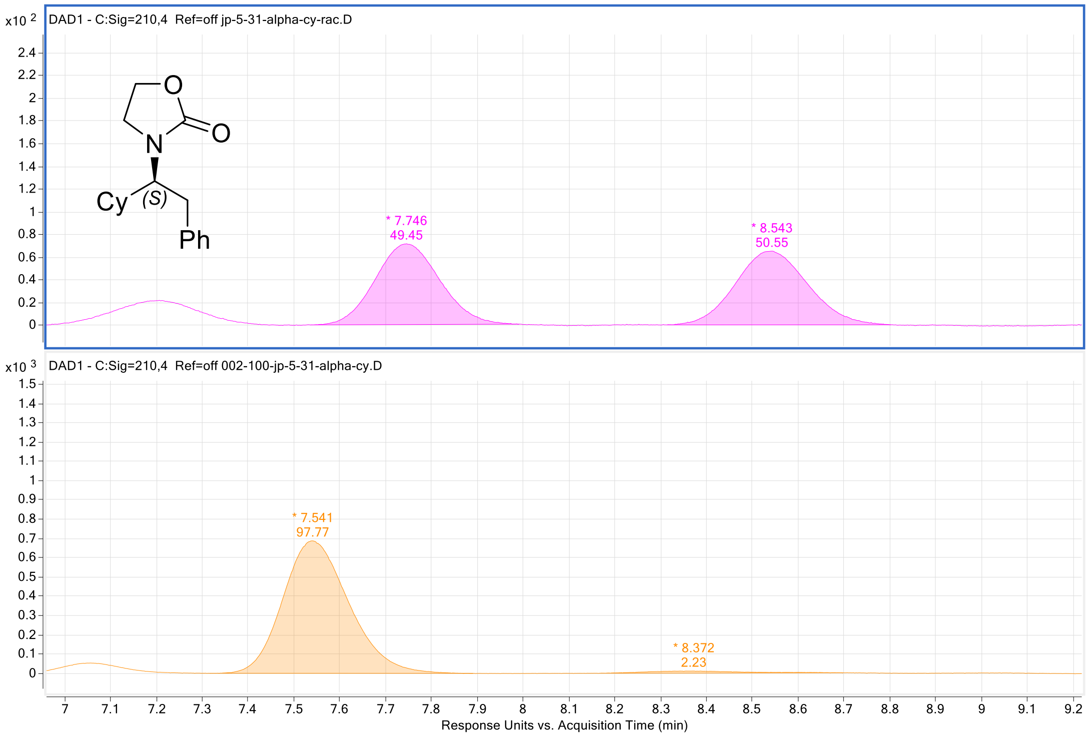
DAD1 - C:Sig=210,4 Ref=off jp-5-31-alpha-cy-rac.D
Cy (S) Ph
* 7.746
49.45
* 8.543
50.55
DAD1 - C:Sig=210,4 Ref=off 002-100-jp-5-31-alpha-cy.D
* 7.541
97.77
* 8.372
2.23
Response Units vs. Acquisition Time (min)

Chromatogram Plots

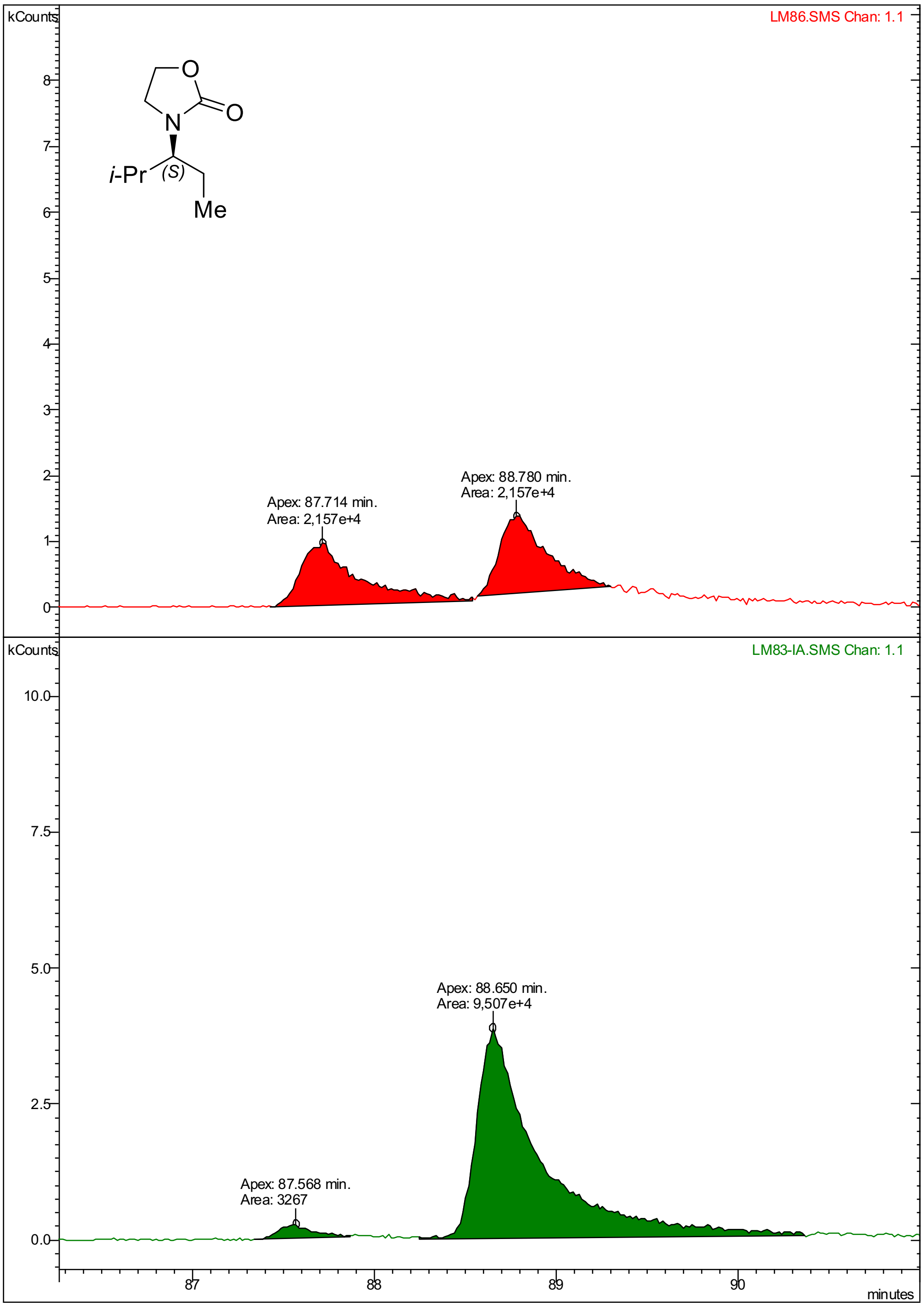

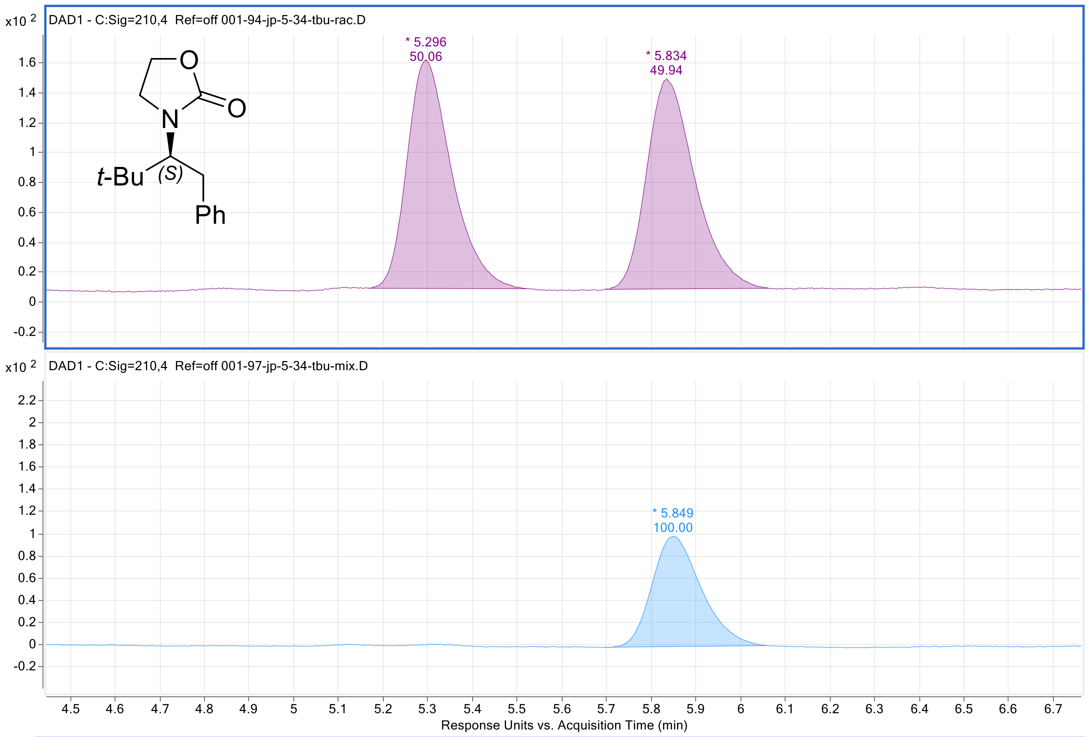

x10 2
DAD1 - C:Sig=210,4 Ref=off 001-94-jp-5-34-tbu-rac.D
* 5.296
50.06
* 5.834
49.94
t-Bu
(S)
Ph
x10 2
DAD1 - C:Sig=210,4 Ref=off 001-97-jp-5-34-tbu-mix.D
* 5.849
100.00
Response Units vs. Acquisition Time (min)

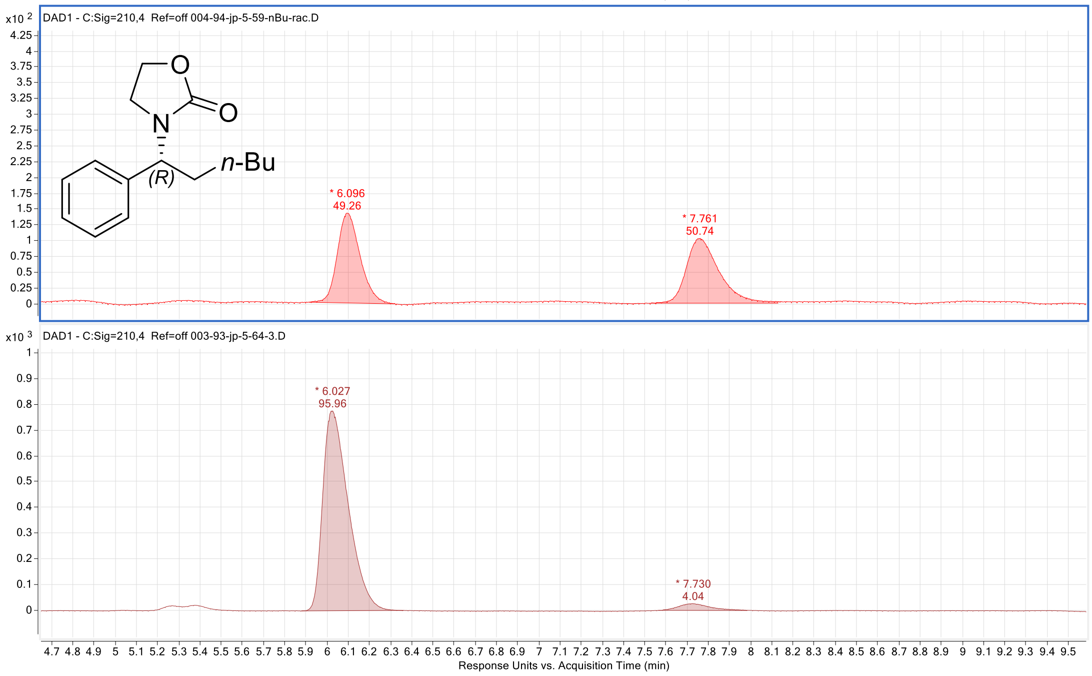

x10 2
DAD1 - C:Sig=210,4 Ref=off 004-94-jp-5-59-nBu-rac.D
(R)
n-Bu
* 6.096
49.26
* 7.761
50.74
x10 3
DAD1 - C:Sig=210,4 Ref=off 003-93-jp-5-64-3.D
* 6.027
95.96
* 7.730
4.04
Response Units vs. Acquisition Time (min)

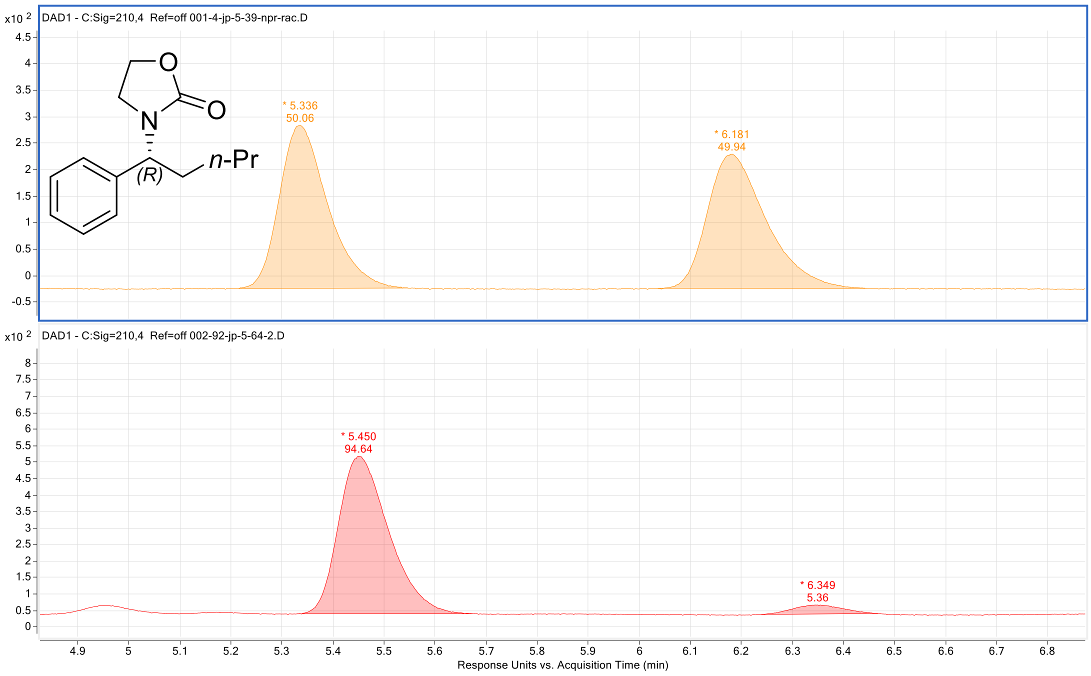

DAD1 - C:Sig=210,4 Ref=off 001-4-jp-5-39-npr-rac.D
* 5.336
50.06
* 6.181
49.94
n-Pr
(R)
DAD1 - C:Sig=210,4 Ref=off 002-92-jp-5-64-2.D
* 5.450
94.64
* 6.349
5.36
Response Units vs. Acquisition Time (min)

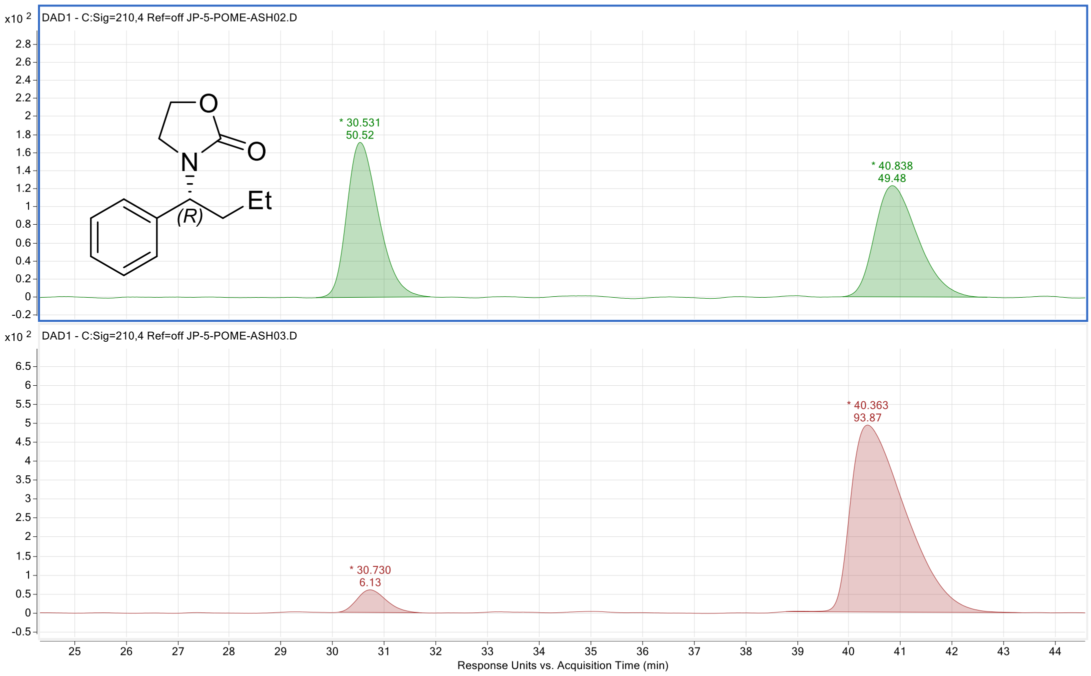

DAD1 - C:Sig=210,4 Ref=off JP-5-POME-ASH02.D
* 30.531
50.52
* 40.838
49.48
Et
(R)
DAD1 - C:Sig=210,4 Ref=off JP-5-POME-ASH03.D
* 30.730
6.13
* 40.363
93.87
Response Units vs. Acquisition Time (min)

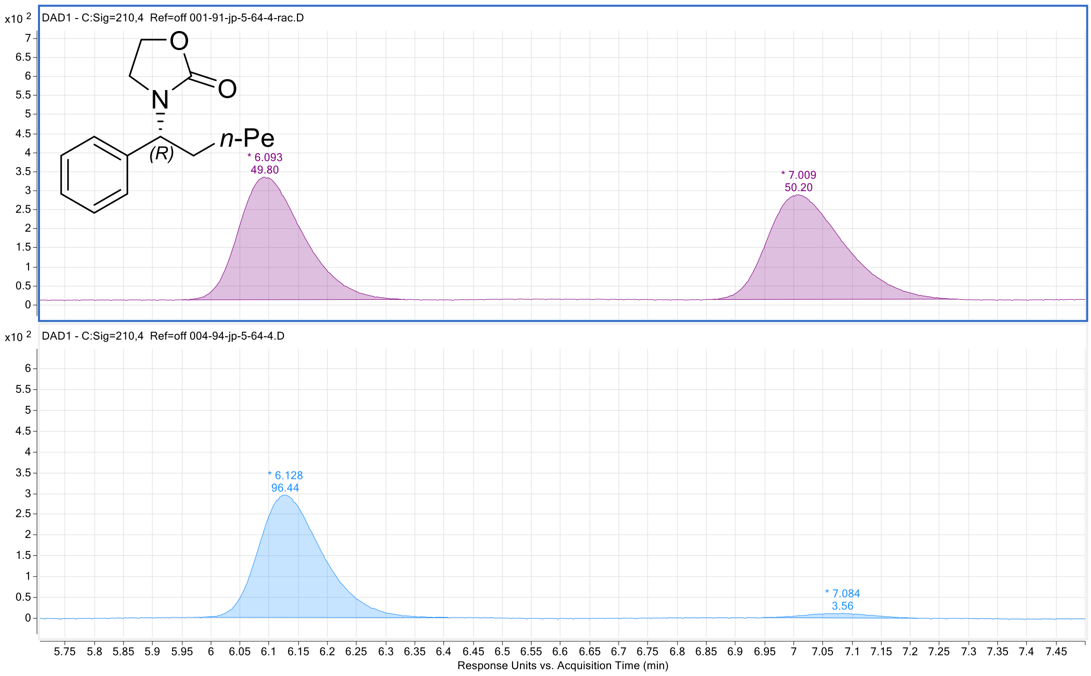

x10 2
DAD1 - C:Sig=210,4 Ref=off 001-91-jp-5-64-4-rac.D
* 6.093
49.80
* 7.009
50.20
x10 2
DAD1 - C:Sig=210,4 Ref=off 004-94-jp-5-64-4.D
* 6.128
96.44
* 7.084
3.56
Response Units vs. Acquisition Time (min)
n-Pe
(R)

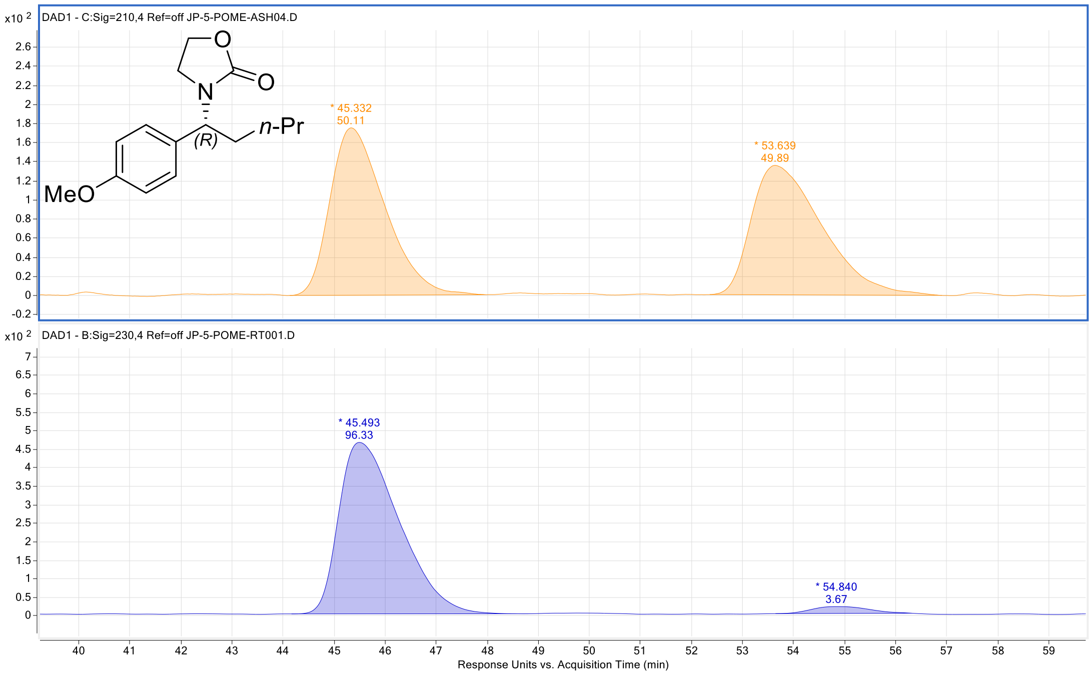

x10 2
DAD1 - C:Sig=210,4 Ref=off JP-5-POME-ASH04.D
* 45.332
50.11
* 53.639
49.89
x10 2
DAD1 - B:Sig=230,4 Ref=off JP-5-POME-RT001.D
* 45.493
96.33
* 54.840
3.67
Response Units vs. Acquisition Time (min)
n-Pr
(R)
MeO

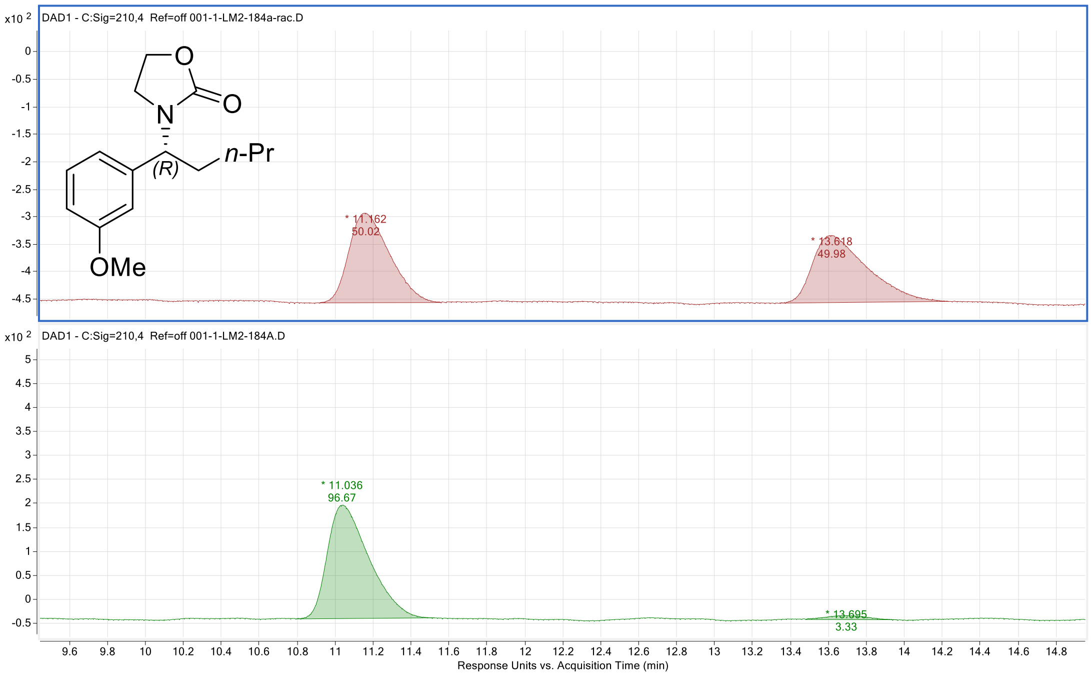
DAD1 - C:Sig=210,4 Ref=off 001-1-LM2-184a-rac.D
(R)
n-Pr
OMe
* 11.162
50.02
* 13.618
49.98
DAD1 - C:Sig=210,4 Ref=off 001-1-LM2-184A.D
* 11.036
96.67
* 13.695
3.33
Response Units vs. Acquisition Time (min)

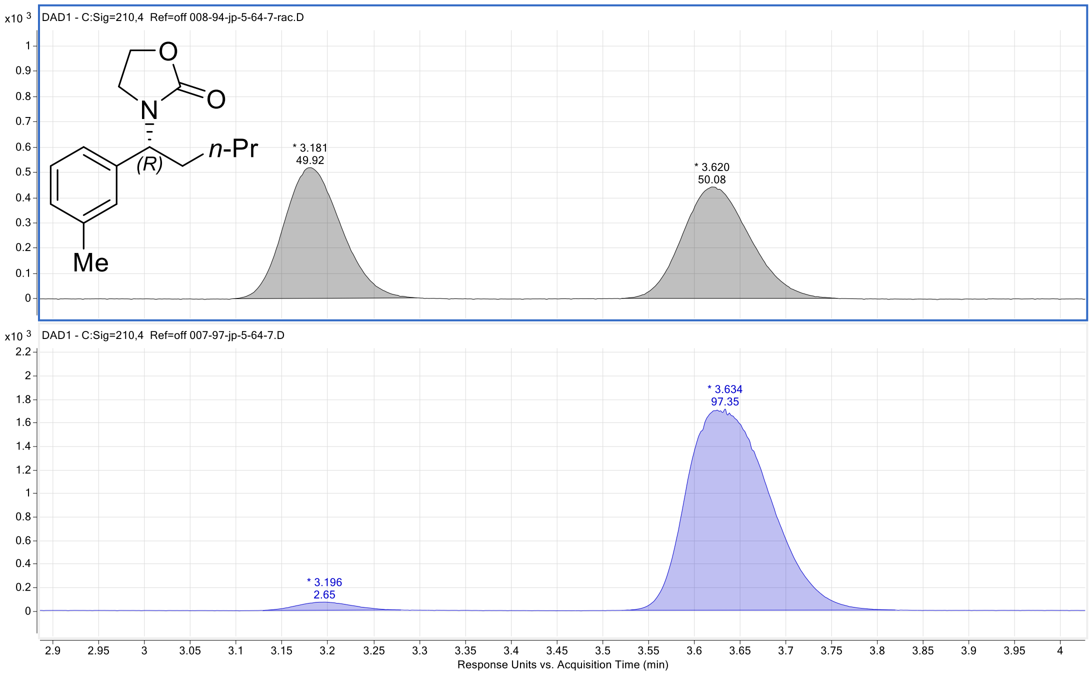
DAD1 - C:Sig=210,4 Ref=off 008-94-jp-5-64-7-rac.D
(R)
n-Pr
Me
* 3.181
49.92
* 3.620
50.08
DAD1 - C:Sig=210,4 Ref=off 007-97-jp-5-64-7.D
* 3.634
97.35
* 3.196
2.65
Response Units vs. Acquisition Time (min)

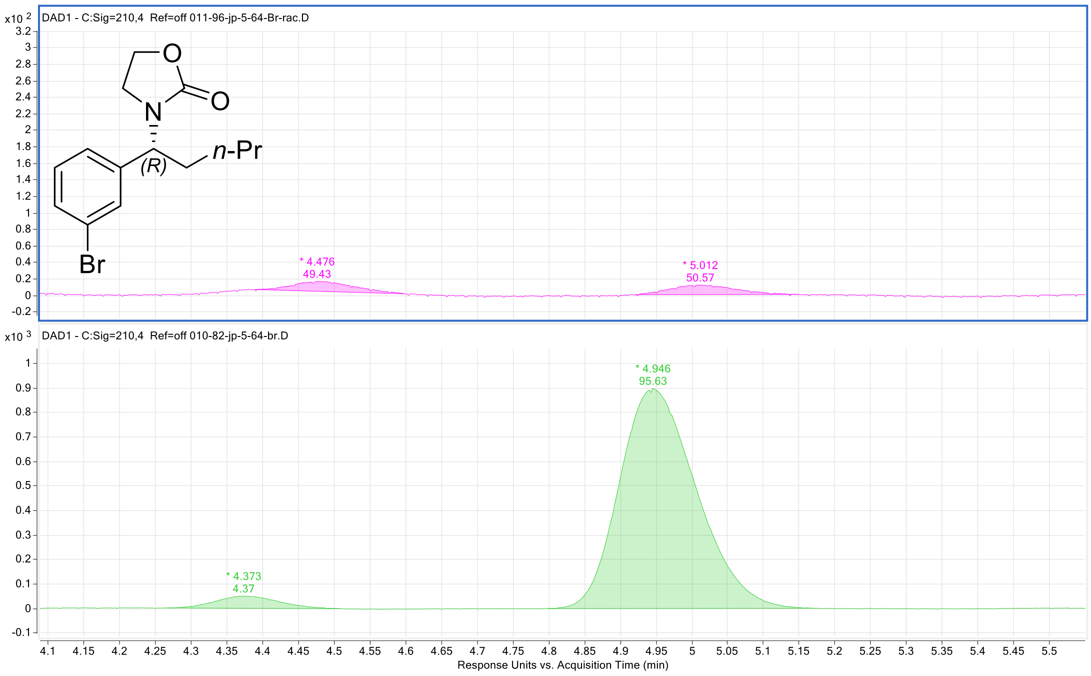
x10 2
DAD1 - C:Sig=210,4 Ref=off 011-96-jp-5-64-Br-rac.D
O
O
N
(R)
n-Pr
Br
* 4.476
49.43
* 5.012
50.57
x10 3
DAD1 - C:Sig=210,4 Ref=off 010-82-jp-5-64-br.D
* 4.946
95.63
* 4.373
4.37
Response Units vs. Acquisition Time (min)

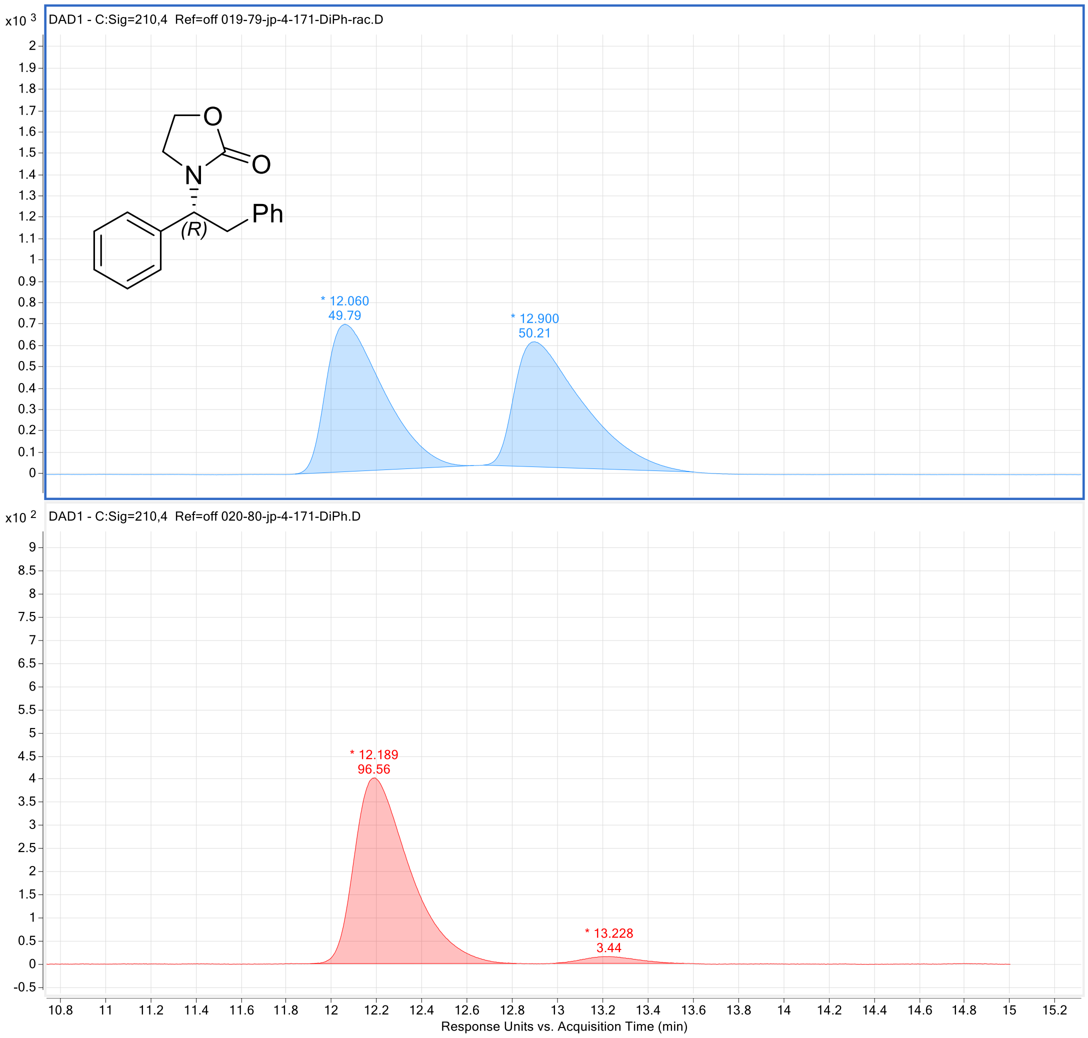
x10 3
DAD1 - C:Sig=210,4 Ref=off 019-79-jp-4-171-DiPh-rac.D
O
O
N
(R)
Ph
* 12.060
49.79
* 12.900
50.21
x10 2
DAD1 - C:Sig=210,4 Ref=off 020-80-jp-4-171-DiPh.D
* 12.189
96.56
* 13.228
3.44
Response Units vs. Acquisition Time (min)

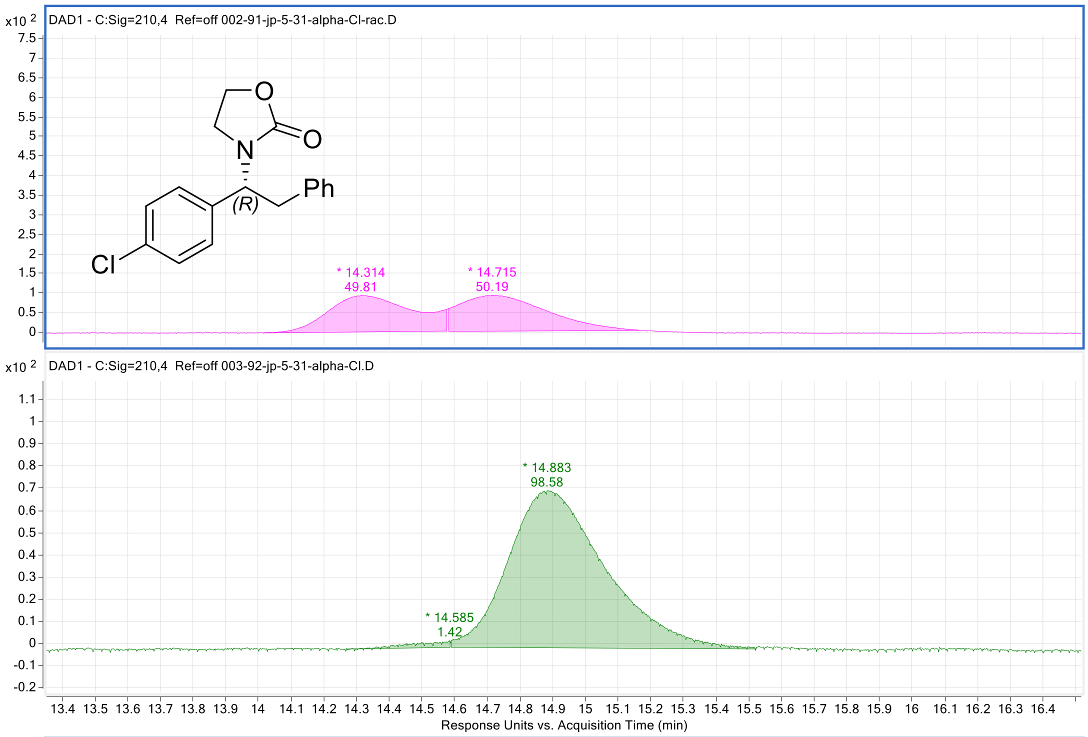
DAD1 - C:Sig=210,4 Ref=off 002-91-jp-5-31-alpha-Cl-rac.D
* 14.314
49.81
* 14.715
50.19
DAD1 - C:Sig=210,4 Ref=off 003-92-jp-5-31-alpha-Cl.D
* 14.883
98.58
* 14.585
1.42
Response Units vs. Acquisition Time (min)
Cl
N
O
O
Ph
(R)

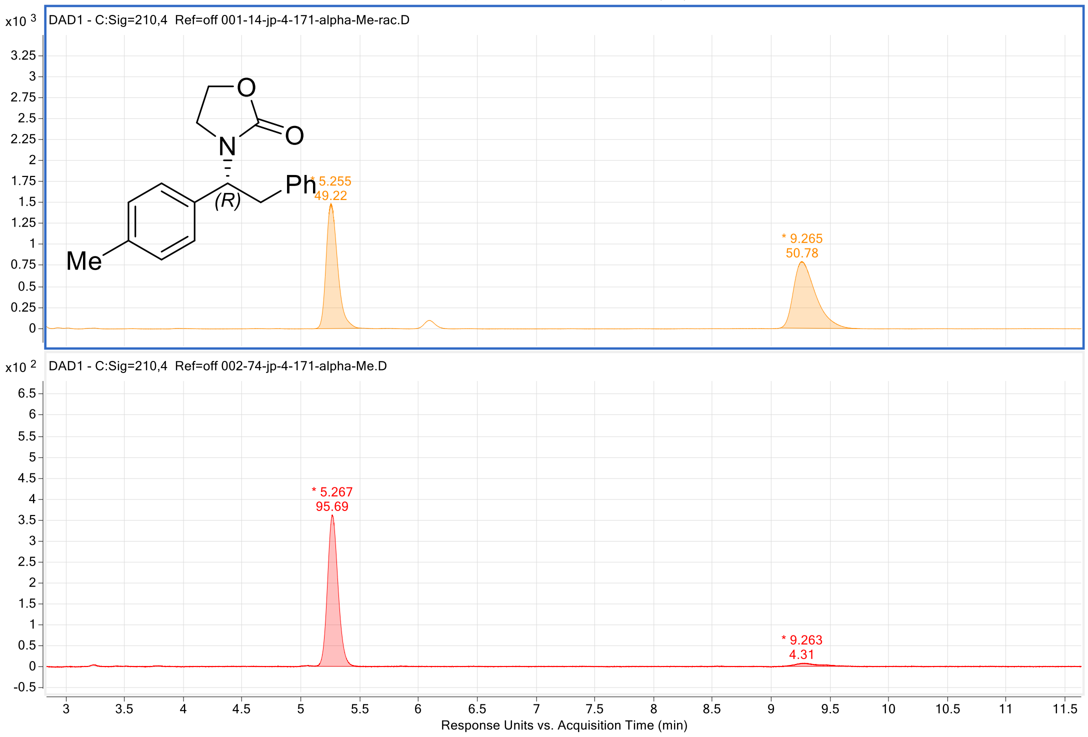
DAD1 - C:Sig=210,4 Ref=off 001-14-jp-4-171-alpha-Me-rac.D
5.255
49.22
* 9.265
50.78
DAD1 - C:Sig=210,4 Ref=off 002-74-jp-4-171-alpha-Me.D
* 5.267
95.69
* 9.263
4.31
Response Units vs. Acquisition Time (min)
Me
N
O
O
Ph
(R)

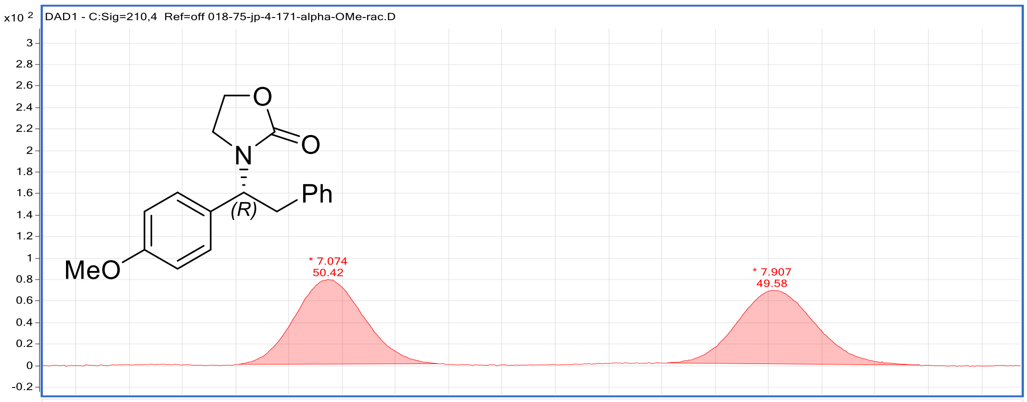
x10 2
DAD1 - C:Sig=210,4 Ref=off 018-75-jp-4-171-alpha-OMe-rac.D
MeO
N
O
O
(R)
Ph
* 7.074
50.42
* 7.907
49.58

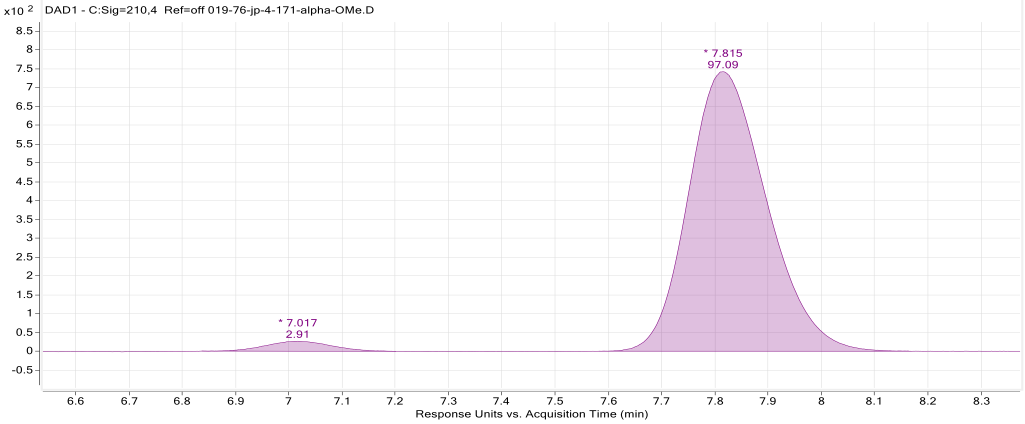
x10 2
DAD1 - C:Sig=210,4 Ref=off 019-76-jp-4-171-alpha-OMe.D
* 7.017
2.91
* 7.815
97.09
Response Units vs. Acquisition Time (min)

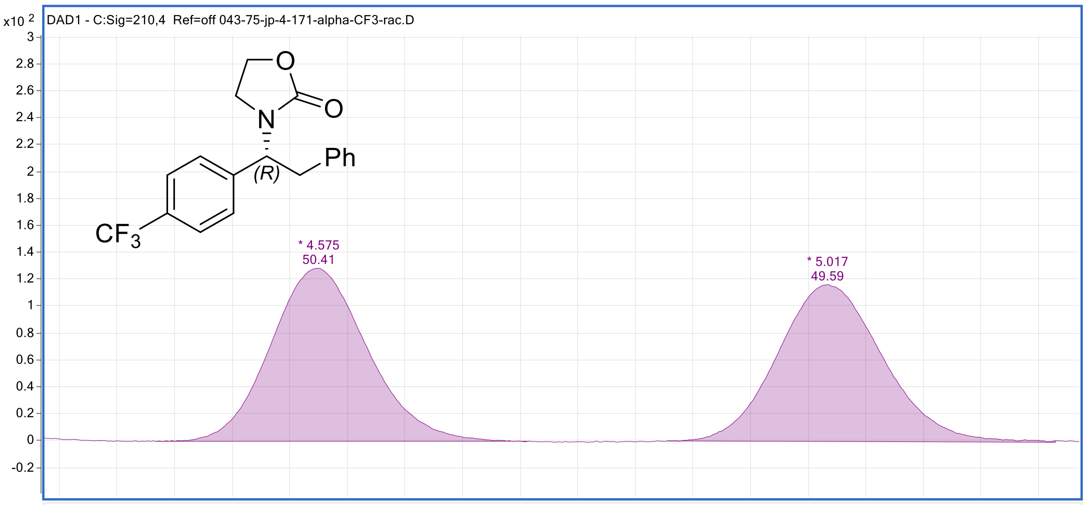
x10 2
DAD1 - C:Sig=210,4 Ref=off 043-75-jp-4-171-alpha-CF3-rac.D
CF3
N
O
O
(R)
Ph
* 4.575
50.41
* 5.017
49.59

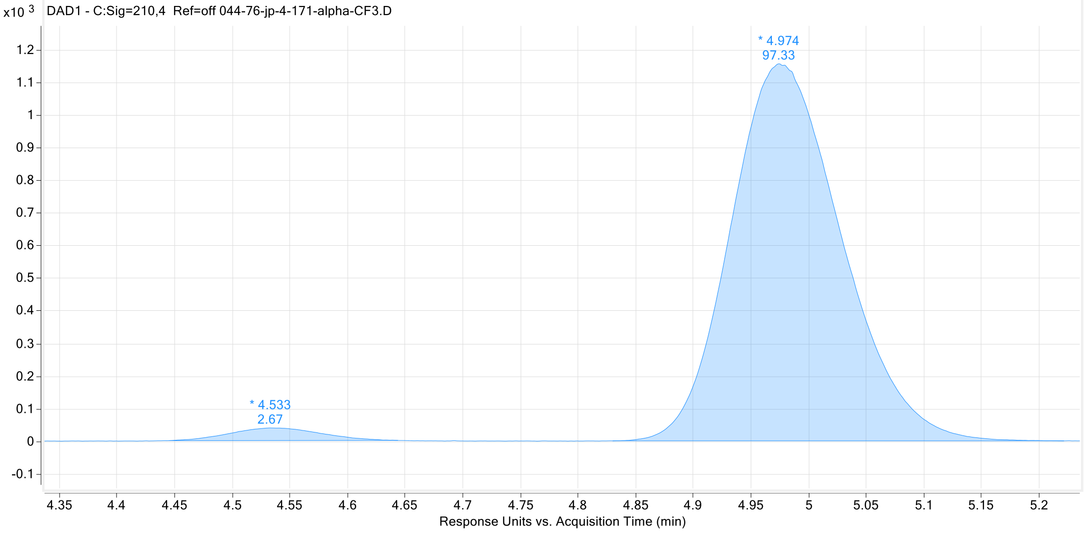
x10 3
DAD1 - C:Sig=210,4 Ref=off 044-76-jp-4-171-alpha-CF3.D
* 4.533
2.67
* 4.974
97.33
Response Units vs. Acquisition Time (min)

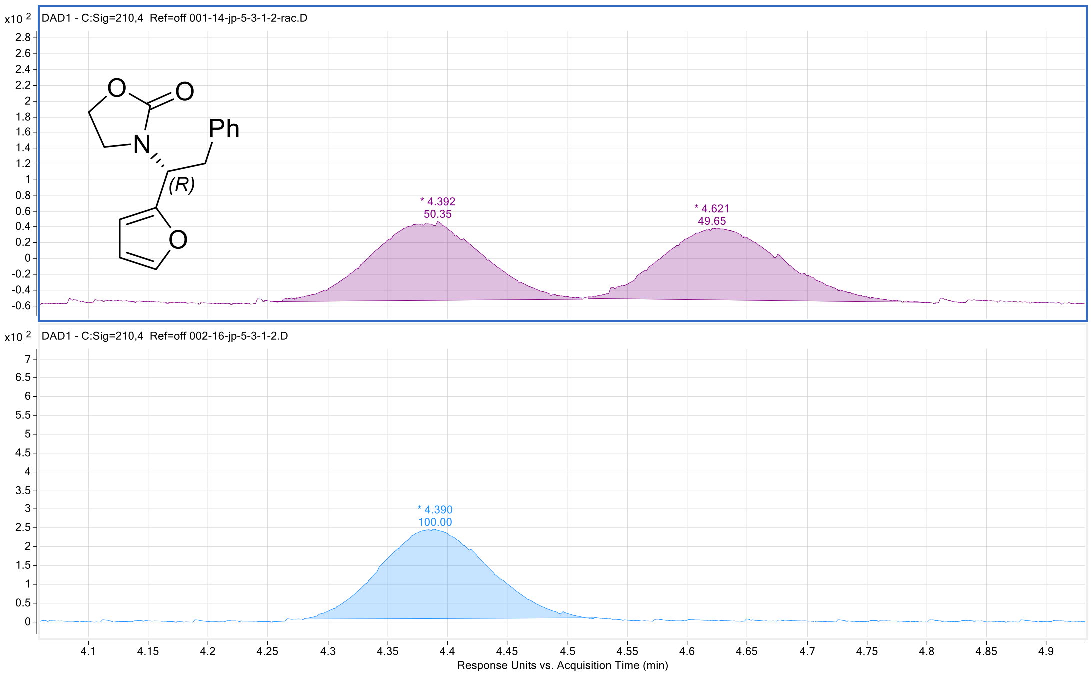
DAD1 - C:Sig=210,4 Ref=off 001-14-jp-5-3-1-2-rac.D
* 4.392
50.35
* 4.621
49.65
DAD1 - C:Sig=210,4 Ref=off 002-16-jp-5-3-1-2.D
* 4.390
100.00
Response Units vs. Acquisition Time (min)
Ph
(R)

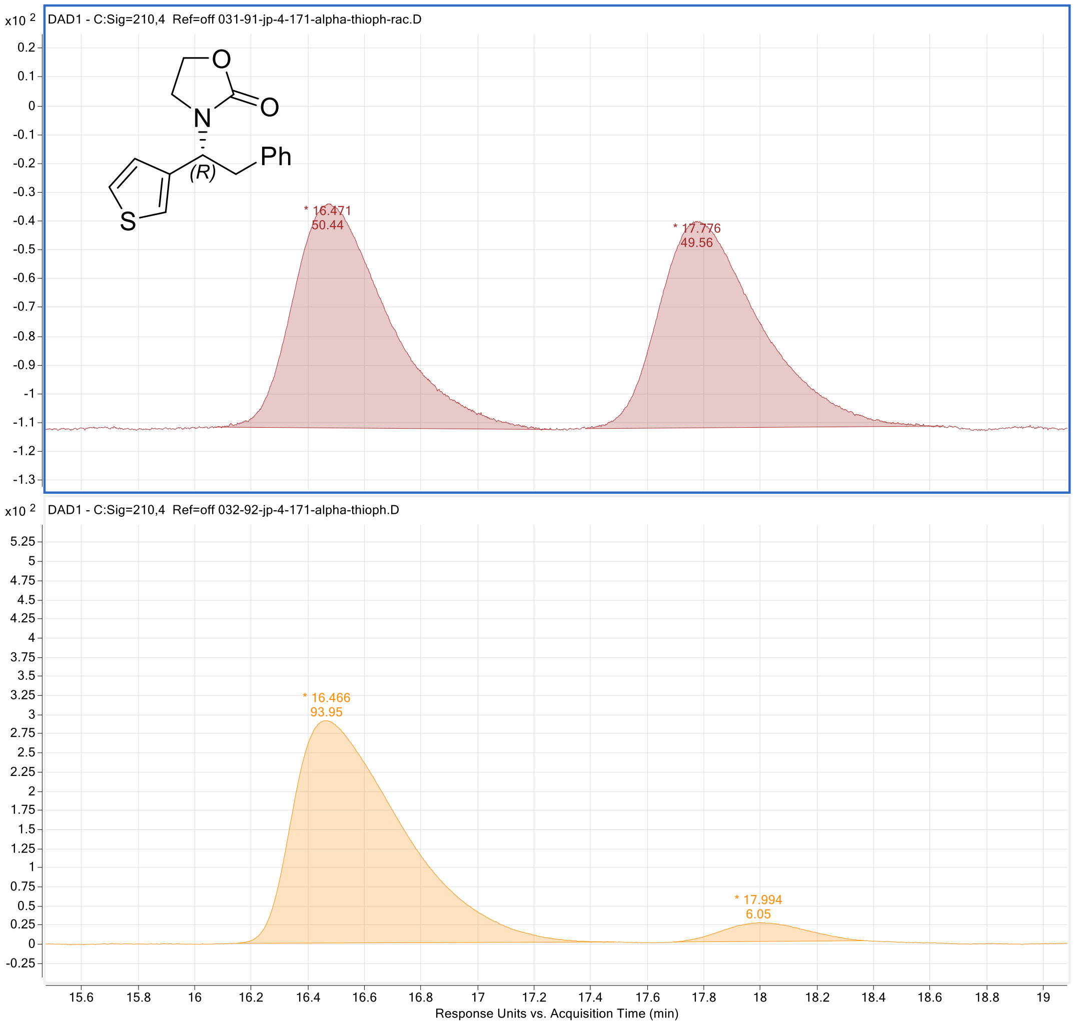
DAD1 - C:Sig=210,4 Ref=off 031-91-jp-4-171-alpha-thioph-rac.D
* 16.471
50.44
* 17.776
49.56
DAD1 - C:Sig=210,4 Ref=off 032-92-jp-4-171-alpha-thioph.D
* 16.466
93.95
* 17.994
6.05
Response Units vs. Acquisition Time (min)
Ph
(R)

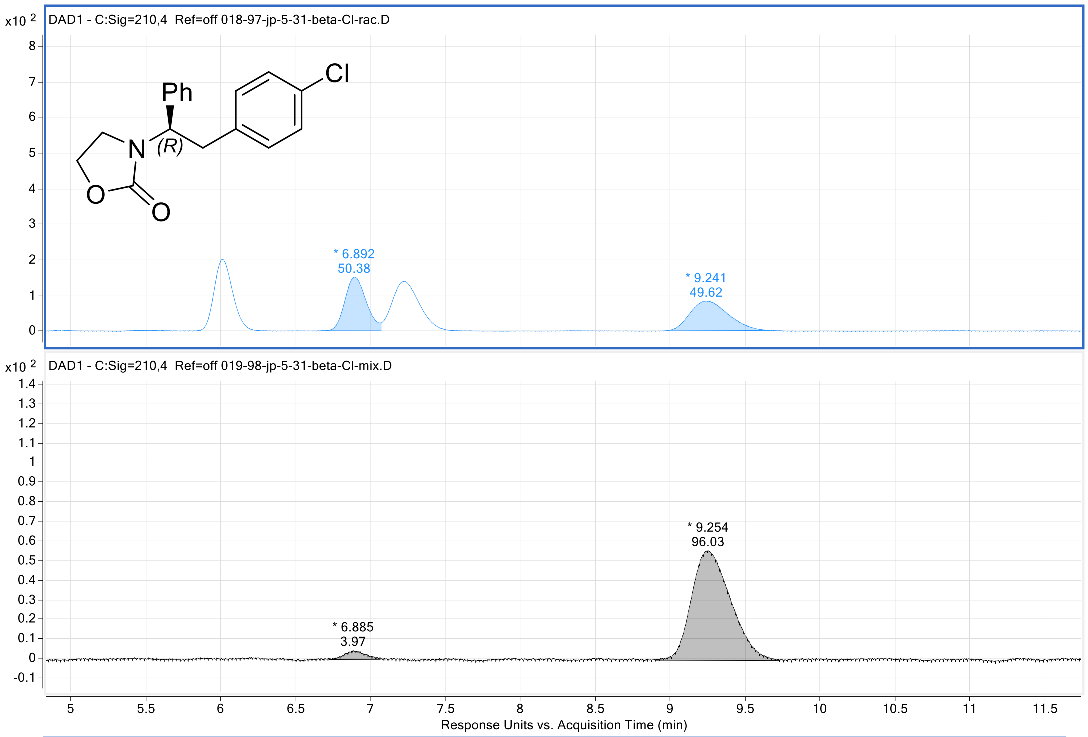
DAD1 - C:Sig=210,4 Ref=off 018-97-jp-5-31-beta-Cl-rac.D
x10 2
Ph
Cl
N
(R)
O
O
* 6.892
50.38
* 9.241
49.62
DAD1 - C:Sig=210,4 Ref=off 019-98-jp-5-31-beta-Cl-mix.D
* 6.885
3.97
* 9.254
96.03
Response Units vs. Acquisition Time (min)

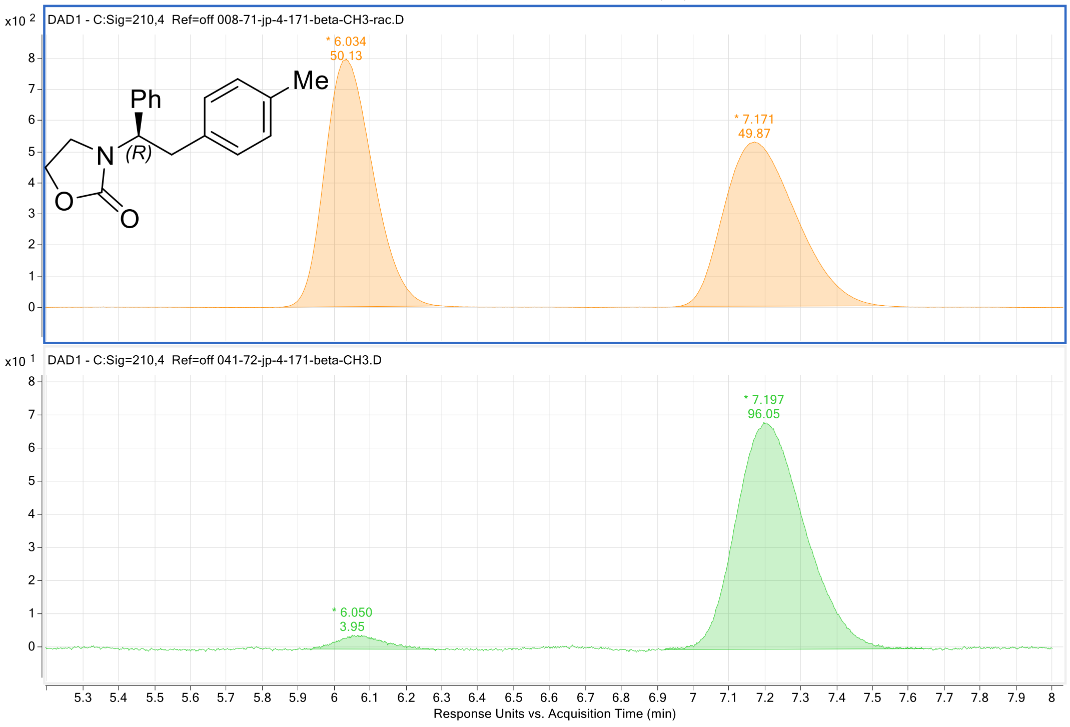
DAD1 - C:Sig=210,4 Ref=off 008-71-jp-4-171-beta-CH3-rac.D
x10 2
Ph
Me
N
(R)
O
O
* 6.034
50.13
* 7.171
49.87
DAD1 - C:Sig=210,4 Ref=off 041-72-jp-4-171-beta-CH3.D
x10 1
* 6.050
3.95
* 7.197
96.05
Response Units vs. Acquisition Time (min)

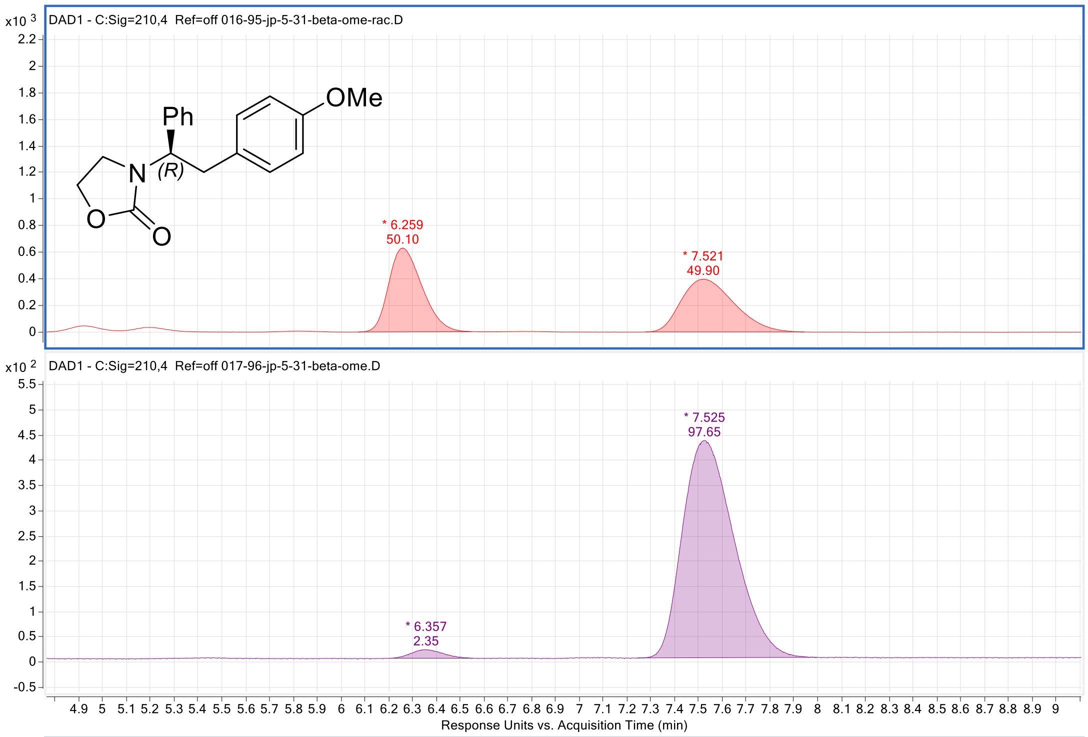
DAD1 - C:Sig=210,4 Ref=off 016-95-jp-5-31-beta-ome-rac.D
OMe
Ph
N
(R)
O
O
* 6.259
50.10
* 7.521
49.90
DAD1 - C:Sig=210,4 Ref=off 017-96-jp-5-31-beta-ome.D
* 7.525
97.65
* 6.357
2.35
Response Units vs. Acquisition Time (min)

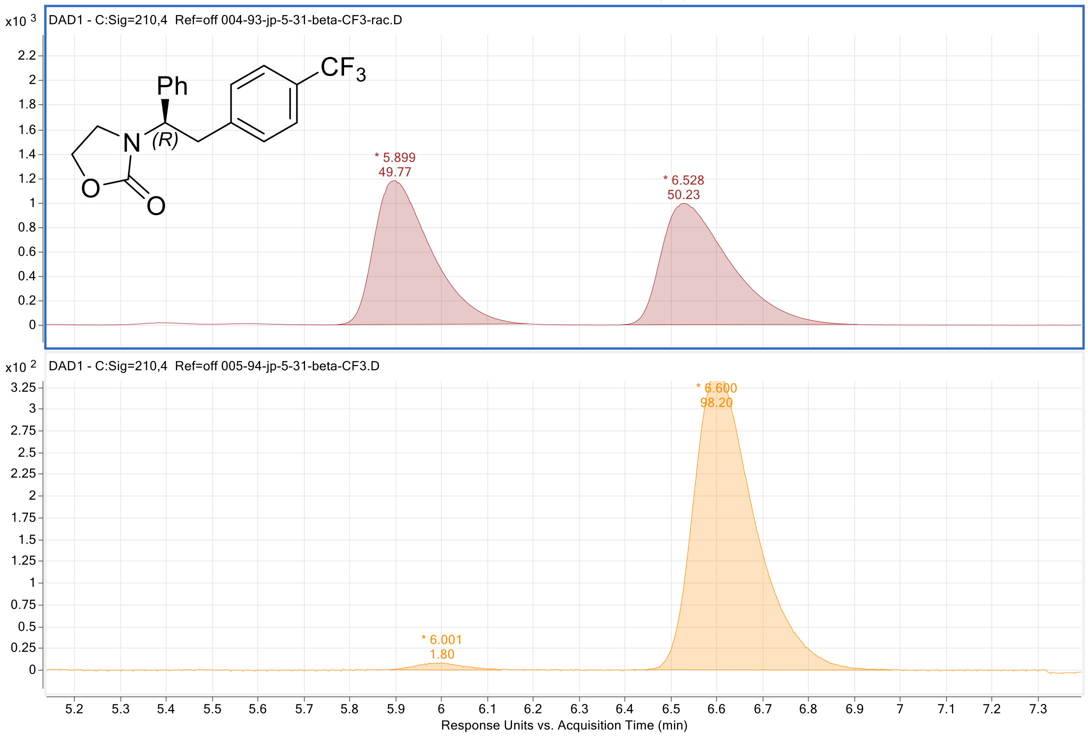
DAD1 - C:Sig=210,4 Ref=off 004-93-jp-5-31-beta-CF3-rac.D
CF3
Ph
N
(R)
O
O
* 5.899
49.77
* 6.528
50.23
DAD1 - C:Sig=210,4 Ref=off 005-94-jp-5-31-beta-CF3.D
* 6.600
98.20
* 6.001
1.80
Response Units vs. Acquisition Time (min)

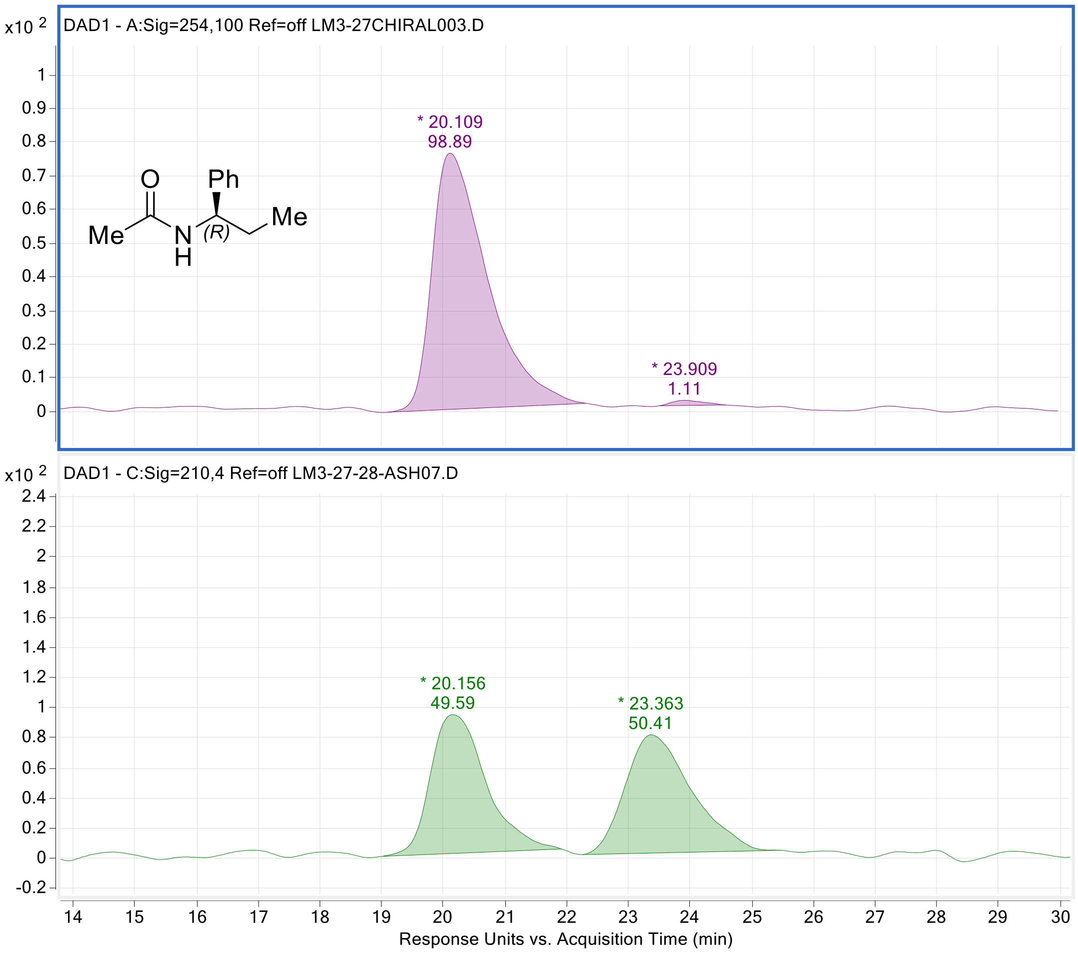

DAD1 - A:Sig=254,100 Ref=off LM3-27CHIRAL003.D
x10 2
* 20.109
98.89
* 23.909
1.11
O
Ph
Me
N
(R)
Me
H
DAD1 - C:Sig=210,4 Ref=off LM3-27-28-ASH07.D
* 20.156
49.59
* 23.363
50.41
Response Units vs. Acquisition Time (min)

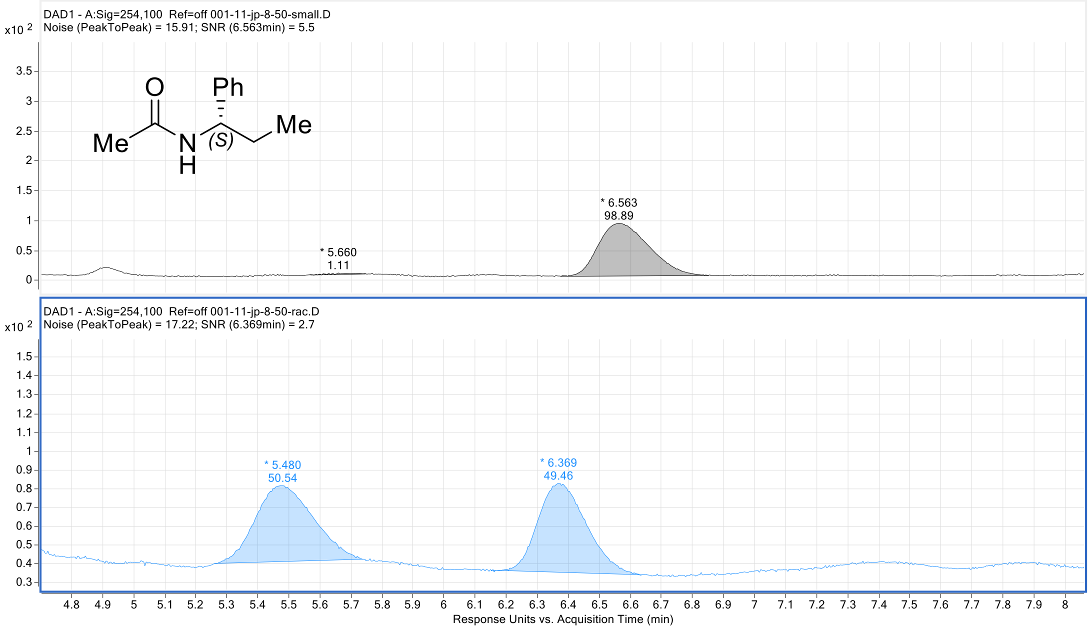

DAD1 - A:Sig=254,100 Ref=off 001-11-jp-8-50-small.D
Noise (PeakToPeak) = 15.91; SNR (6.563min) = 5.5
O
Ph
Me
N
(S)
Me
H
* 5.660
1.11
* 6.563
98.89
DAD1 - A:Sig=254,100 Ref=off 001-11-jp-8-50-rac.D
Noise (PeakToPeak) = 17.22; SNR (6.369min) = 2.7
* 5.480
50.54
* 6.369
49.46
Response Units vs. Acquisition Time (min)

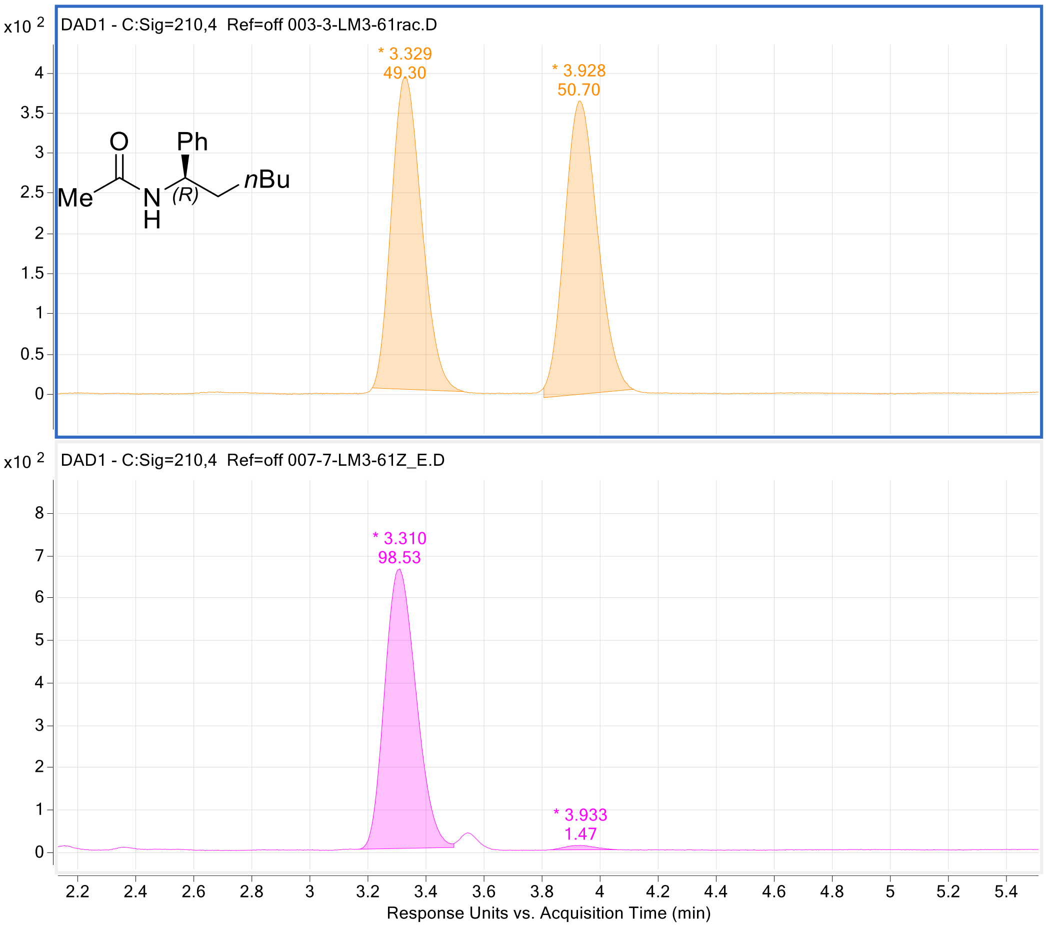
DAD1 - C:Sig=210,4 Ref=off 003-3-LM3-61rac.D
* 3.329
49.30
* 3.928
50.70
O
Ph
Me
N
H
(R)
nBu
DAD1 - C:Sig=210,4 Ref=off 007-7-LM3-61Z_E.D
* 3.310
98.53
* 3.933
1.47
x10 2
Response Units vs. Acquisition Time (min)

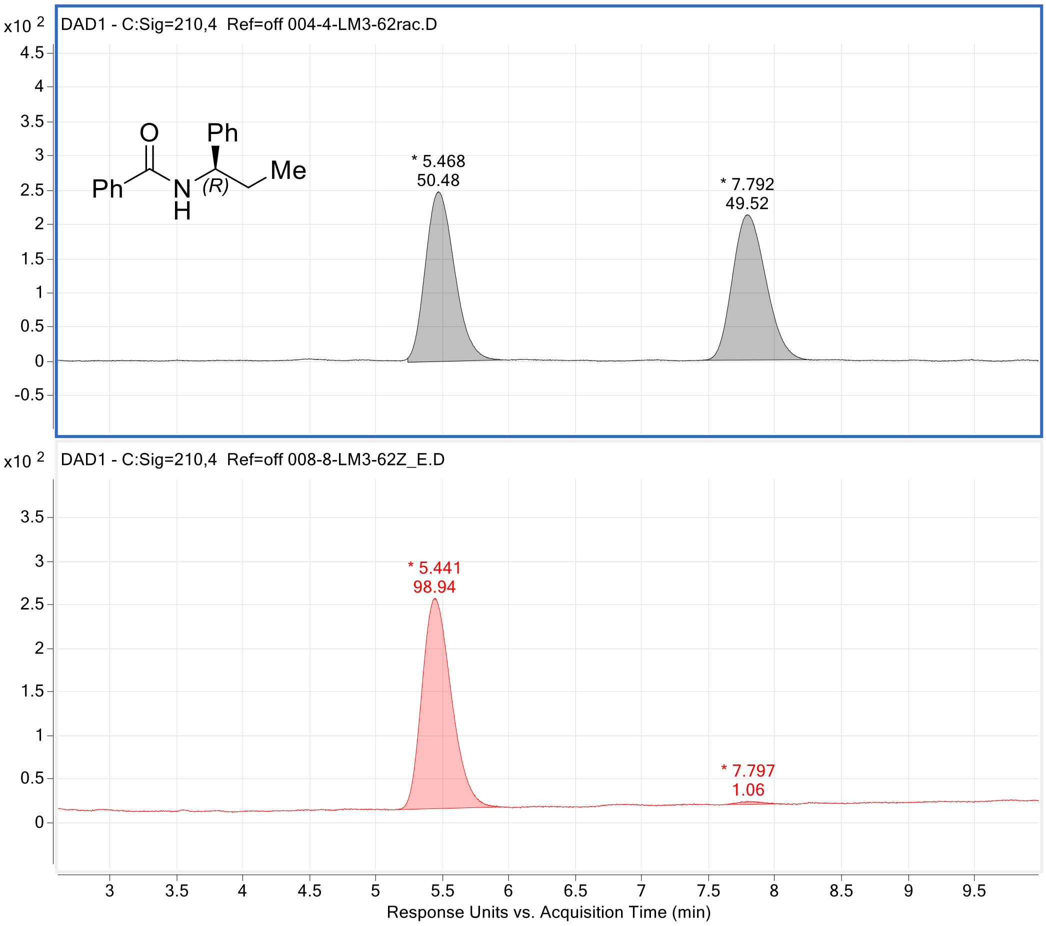
DAD1 - C:Sig=210,4 Ref=off 004-4-LM3-62rac.D
O
Ph
Ph
N
H
(R)
Me
* 5.468
50.48
* 7.792
49.52
DAD1 - C:Sig=210,4 Ref=off 008-8-LM3-62Z_E.D
* 5.441
98.94
* 7.797
1.06
x10 2
Response Units vs. Acquisition Time (min)

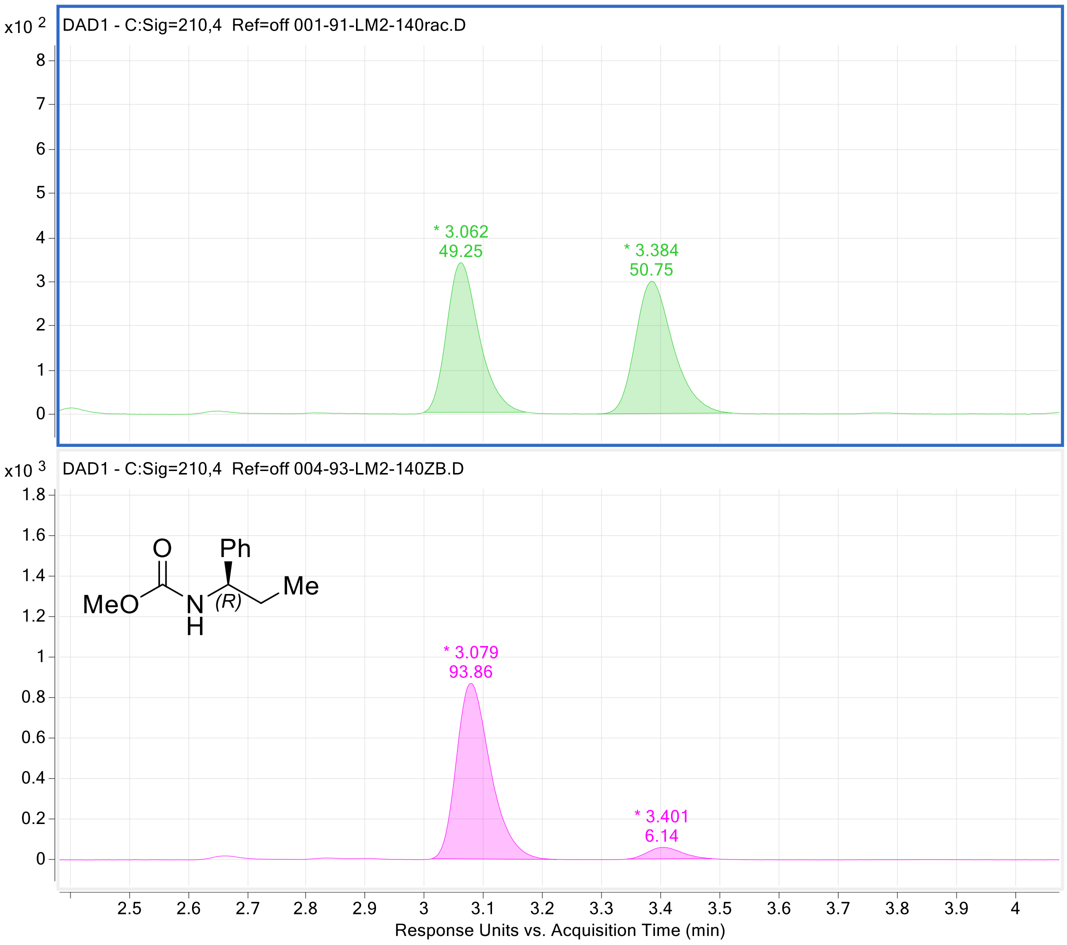

x10 2
DAD1 - C:Sig=210,4 Ref=off 001-91-LM2-140rac.D
* 3.062
49.25
* 3.384
50.75
x10 3
DAD1 - C:Sig=210,4 Ref=off 004-93-LM2-140ZB.D
MeO
O
N
H
Ph
(R)
Me
* 3.079
93.86
* 3.401
6.14
Response Units vs. Acquisition Time (min)

12. XYZ Coordinates

C1_E_R_tN_Chelbot

E1				TS_{E1-2}			
C1	-4,217983	-2,961081	-0,742644	C1	-4,326353	-3,069149	-0,140508
C2	-2,146720	-4,230141	-1,006060	C2	-2,298479	-4,317481	-0,691570
C3	-3,448169	-3,861797	-1,767178	C3	-3,698641	-3,965591	-1,261091
C4	-3,244984	-2,932842	0,457047	C4	-3,200364	-3,040437	0,917641
H5	-5,174511	-3,406053	-0,449666	H5	-5,234702	-3,516393	0,276092
H6	-3,242012	-3,348974	-2,711854	H6	-3,635005	-3,454494	-2,226967
H7	-3,666576	-2,567680	1,394082	H7	-3,492970	-2,686622	1,906936
H8	-4,416042	-1,955037	-1,117025	H8	-4,574771	-2,062567	-0,483851
H9	-1,599874	-5,081628	-1,411687	H9	-1,801689	-5,158727	-1,174628
H10	-4,011077	-4,769262	-2,002925	H10	-4,278898	-4,880162	-1,412470
C11	-2,661192	-4,356709	0,444933	C11	-2,604801	-4,455684	0,816870
H12	-1,867298	-4,511814	1,181620	H12	-1,715398	-4,594538	1,439202
H13	-3,420952	-5,135101	0,558334	H13	-3,329531	-5,246423	1,030452
C14	0,136670	-3,117726	-0,470256	C14	0,029577	-3,170008	-0,512794
N15	0,945008	-2,141631	-0,217192	N15	0,845228	-2,194731	-0,297325
C16	2,285599	-2,703712	0,189977	C16	2,230390	-2,744495	-0,142560
C17	1,961868	-4,240930	0,205644	C17	1,989389	-4,264031	-0,471878
P18	-1,642551	-0,562963	0,482416	P18	-1,701858	-0,619263	0,613492
Ir19	0,507575	-0,096073	-0,642132	Ir19	0,268037	-0,103879	-0,509097
C20	-1,584430	-0,516713	2,312500	C20	-1,565712	-0,388031	2,416768
C21	-1,306935	-0,427523	5,105348	C21	-1,061501	0,028596	5,139894
C22	-1,562094	-1,700793	3,064011	C22	-1,332524	-1,477949	3,266335
C23	-1,444521	0,714030	2,976223	C23	-1,533391	0,915829	2,938951
C24	-1,308760	0,754856	4,362232	C24	-1,281444	1,118262	4,293713
C25	-1,429720	-1,654058	4,452087	C25	-1,088141	-1,267963	4,624533
H26	-1,439490	1,642076	2,420113	H26	-1,691820	1,772571	2,293468
H27	-1,199532	1,715866	4,856587	H27	-1,250387	2,131733	4,682901
H28	-1,412366	-2,579102	5,021146	H28	-0,909408	-2,118618	5,276163
H29	-1,202378	-0,393318	6,186140	H29	-0,866253	0,190082	6,196336
O30	0,601784	-4,352360	-0,305102	O30	0,544130	-4,397058	-0,582575
C31	2,708978	-2,294362	1,598708	C31	2,730609	-2,593664	1,294562
C32	1,873687	-1,591198	2,467328	C32	2,010779	-1,902579	2,271580
C33	3,947759	-2,751317	2,078196	C33	3,928071	-3,226263	1,666424
C34	2,279970	-1,309031	3,773741	C34	2,489850	-1,814182	3,580310
H35	0,902173	-1,258948	2,138525	H35	1,070139	-1,435256	2,022996
C36	4,352384	-2,474159	3,381663	C36	4,409299	-3,136010	2,970557
H37	4,605795	-3,316608	1,423298	H37	4,496541	-3,788116	0,929360
C38	3,521596	-1,742162	4,233446	C38	3,693234	-2,421217	3,933232
H39	1,609716	-0,755633	4,424736	H39	1,905697	-1,272607	4,318942
H40	5,318218	-2,828055	3,730890	H40	5,342112	-3,626144	3,234479

H41	3,839997	-1,521054	5,248203	H41	4,068228	-2,349612	4,950148
C42	-3,105029	0,438837	0,002537	C42	-3,163881	0,345875	0,082363
C43	-3,991432	1,029815	0,910083	C43	-4,137639	0,818475	0,971414
C44	-3,335795	0,582522	-1,377719	C44	-3,329497	0,547047	-1,298585
C45	-5,083130	1,767663	0,444787	C45	-5,252463	1,503642	0,486761
H46	-3,836449	0,921464	1,978259	H46	-4,027051	0,662314	2,039464
C47	-4,427992	1,316089	-1,836038	C47	-4,449318	1,223660	-1,777266
C48	-5,302309	1,917454	-0,924484	C48	-5,408674	1,709532	-0,884080
H49	-5,763259	2,223892	1,158548	H49	-5,999182	1,875358	1,182631
H50	-4,598848	1,419094	-2,903984	H50	-4,573866	1,376074	-2,845695
H51	-6,151228	2,492966	-1,281399	H51	-6,277328	2,244172	-1,257524
H52	1,217781	0,301025	0,671998	H52	0,809250	0,302155	0,885602
H53	1,966990	-0,193766	-1,251191	H53	1,761691	0,324223	-1,126202
H54	-1,621030	-2,661793	2,566426	H54	-1,314295	-2,485659	2,864748
H55	-2,659288	0,112099	-2,087244	H55	-2,580808	0,172955	-1,992892
H56	2,613839	-4,832065	-0,438096	H56	2,421554	-4,567450	-1,427849
C57	5,054144	-1,695549	-3,004939	C57	4,668804	-0,975381	-3,284282
C58	3,980620	-2,560454	-3,230625	C58	3,413773	-1,524739	-3,560180
C59	3,115557	-2,883708	-2,188385	C59	2,657527	-2,085738	-2,535536
C60	3,300606	-2,348532	-0,905519	C60	3,130491	-2,104447	-1,212755
C61	4,362372	-1,462754	-0,696142	C61	4,382883	-1,545776	-0,945475
C62	5,240365	-1,149591	-1,736252	C62	5,148338	-0,991676	-1,976867
H63	5,737463	-1,449884	-3,812761	H63	5,267369	-0,543320	-4,081299
H64	3,820805	-2,987374	-4,216507	H64	3,024195	-1,526816	-4,575128
H65	2,278247	-3,548897	-2,386337	H65	1,673687	-2,485027	-2,762653
H66	4,505742	-1,006868	0,275629	H66	4,767213	-1,526120	0,067613
H67	6,066795	-0,469186	-1,549668	H67	6,120499	-0,563825	-1,748669
C68	3,267813	4,712826	-1,323067	C68	3,347349	4,696527	-1,269022
C69	2,107553	3,939122	-1,346076	C69	2,257195	3,863278	-1,506913
C70	2,182263	2,550650	-1,537215	C70	2,361124	2,483024	-1,282835
C71	3,434405	1,959059	-1,735215	C71	3,556407	1,952717	-0,797408
C72	4,592860	2,732861	-1,711982	C72	4,645917	2,789070	-0,552133
C73	4,514114	4,110933	-1,499640	C73	4,544937	4,159851	-0,790562
H74	3,196343	5,785120	-1,164808	H74	3,257307	5,765118	-1,440894
H75	1,142227	4,413318	-1,204033	H75	1,313108	4,283658	-1,842150
H76	3,500934	0,889610	-1,896792	H76	3,628938	0,889274	-0,599538
H77	5,558408	2,256440	-1,857889	H77	5,570902	2,368436	-0,168303
H78	5,418000	4,713050	-1,475040	H78	5,391708	4,811301	-0,594551
C79	0,902514	1,769095	-1,664965	C79	1,157428	1,631428	-1,598407
C80	-0,237991	1,965231	-0,820551	C80	-0,154618	1,918012	-0,987593
C81	0,558192	2,556093	-4,091901	C81	1,504788	2,137058	-4,129043
C82	-0,096858	0,424383	-3,470273	C82	0,319956	0,254852	-3,453272
C83	0,155122	1,716382	-5,317908	C83	1,218739	1,194877	-5,315228
H84	1,530633	3,038250	-4,202904	H84	2,559418	2,405080	-4,046797
H85	-0,187469	3,318441	-3,830072	H85	0,907966	3,057059	-4,159715
H86	-0,575690	2,199179	-5,967003	H86	0,743135	1,685709	-6,164536
H87	1,022577	1,399900	-5,904479	H87	2,118496	0,667777	-5,644443
N88	0,599315	1,508777	-3,069530	N88	1,085669	1,282304	-3,019554

O89	-0,461746	0,520574	-4,757112	O89	0,291728	0,200603	-4,792627
O90	-0,389742	-0,552526	-2,762735	O90	-0,283618	-0,560806	-2,738448
H91	1,964421	-4,648753	1,216712	H91	2,324442	-4,930267	0,322429
N92	-2,035846	-2,150666	0,080105	N92	-2,054302	-2,251580	0,395421
C93	-1,290393	-2,942639	-0,929703	C93	-1,458690	-3,015969	-0,725121
H94	-1,249303	-2,439083	-1,900732	H94	-1,574455	-2,503202	-1,689593
H96	-1,194468	1,932784	-1,335777	H96	-0,962564	1,948410	-1,718876
C97	-0,498673	4,443000	2,681663	C97	-0,985647	4,668138	2,219882
C98	-1,561021	4,344187	1,783534	C98	-1,913474	4,439515	1,203315
C99	-1,450823	3,527516	0,656440	C99	-1,611458	3,559742	0,162861
C100	-0,278246	2,796547	0,409463	C100	-0,382765	2,880911	0,123258
C101	0,785585	2,902920	1,324251	C101	0,536328	3,109490	1,162412
C102	0,674778	3,719417	2,444452	C102	0,241342	3,999650	2,190582
H103	-0,581478	5,077476	3,559638	H103	-1,215570	5,357422	3,027490
H104	-2,478782	4,898225	1,958897	H104	-2,875106	4,945002	1,217179
H105	-2,288299	3,445093	-0,031522	H105	-2,347117	3,377852	-0,616807
H106	1,697500	2,343717	1,156198	H106	1,481502	2,578997	1,172862
H107	1,507146	3,789394	3,139051	H107	0,969596	4,164491	2,980032

E2				TS_{E2-3}			
C1	-4,310252	-2,924883	-0,331866	C1	-4,379163	-2,466986	-0,570456
C2	-2,335369	-4,299957	-0,749330	C2	-2,518987	-3,920038	-1,199674
C3	-3,684718	-3,888881	-1,395958	C3	-3,806656	-3,278377	-1,780971
C4	-3,240892	-2,935730	0,783455	C4	-3,337855	-2,755309	0,533395
H5	-5,264549	-3,303359	0,048446	H5	-5,365366	-2,833067	-0,266676
H6	-3,547236	-3,412233	-2,371652	H6	-3,596981	-2,648985	-2,651604
H7	-3,567149	-2,543731	1,747216	H7	-3,649388	-2,501741	1,547016
H8	-4,477788	-1,914490	-0,711340	H8	-4,466252	-1,397555	-0,770684
H9	-1,860365	-5,177825	-1,187676	H9	-2,117193	-4,753144	-1,776493
H10	-4,309415	-4,773470	-1,548834	H10	-4,500832	-4,060062	-2,102078
C11	-2,722906	-4,384139	0,743944	C11	-2,950066	-4,217981	0,253925
H12	-1,875215	-4,557498	1,414056	H12	-2,138595	-4,581238	0,892112
H13	-3,501852	-5,127177	0,937118	H13	-3,797556	-4,906863	0,311831
C14	0,048710	-3,271948	-0,515508	C14	-0,068588	-3,203272	-0,720364
N15	0,867103	-2,323492	-0,227775	N15	0,827096	-2,417788	-0,237825
C16	2,230002	-2,895045	-0,033744	C16	2,125528	-3,148807	-0,159069
C17	2,004946	-4,364624	-0,540303	C17	1,691392	-4,589260	-0,623258
P18	-1,598852	-0,615465	0,580967	P18	-1,485328	-0,605602	0,761344
Ir19	0,316723	-0,213543	-0,443297	Ir19	0,501156	-0,238348	-0,128020
C20	-1,518308	-0,421521	2,390162	C20	-1,427804	-0,744901	2,576922
C21	-1,145881	-0,071354	5,141192	C21	-1,077195	-0,936324	5,347086
C22	-1,456268	-1,534034	3,239332	C22	-1,467774	-1,995812	3,207787
C23	-1,371556	0,870271	2,924533	C23	-1,189389	0,411065	3,340824
C24	-1,187587	1,039322	4,293923	C24	-1,018596	0,310591	4,718797
C25	-1,279222	-1,355842	4,612617	C25	-1,299789	-2,087288	4,590690
H26	-1,385641	1,740031	2,276880	H26	-1,118755	1,382259	2,864281
H27	-1,067555	2,041546	4,694532	H27	-0,828851	1,209564	5,297719
H28	-1,234850	-2,222899	5,265627	H28	-1,333497	-3,060084	5,072872

H29	-1,003119	0,064363	6,209590	H29	-0,942385	-1,010820	6,422489
O30	0,554934	-4,501211	-0,641230	O30	0,297433	-4,451438	-1,025002
C31	2,620530	-2,888413	1,445135	C31	2,659326	-3,223281	1,265878
C32	1,873682	-2,208993	2,409726	C32	1,942736	-2,720863	2,353953
C33	3,758114	-3,602557	1,851339	C33	3,859123	-3,911626	1,502969
C34	2,260830	-2,229865	3,751081	C34	2,430909	-2,872573	3,653485
H35	0,989240	-1,660306	2,121231	H35	1,005222	-2,207361	2,194083
C36	4,146315	-3,622584	3,188999	C36	4,345603	-4,063904	2,799427
H37	4,356600	-4,135040	1,115902	H37	4,421272	-4,321764	0,667303
C38	3,398394	-2,931582	4,145199	C38	3,634417	-3,537851	3,880614
H39	1,660076	-1,695653	4,481712	H39	1,857784	-2,468659	4,483561
H40	5,032614	-4,177043	3,483833	H40	5,279886	-4,592790	2,964873
H41	3,700777	-2,946691	5,188376	H41	4,015727	-3,654000	4,891040
C42	-2,988850	0,424621	0,009737	C42	-2,791648	0,605202	0,352697
C43	-3,935209	0,975723	0,883932	C43	-3,664908	1,143449	1,305132
C44	-3,135409	0,608714	-1,375971	C44	-2,959896	0,926050	-1,006044
C45	-5,003560	1,718615	0,379234	C45	-4,685970	2,007268	0,904484
H46	-3,841287	0,835581	1,955323	H46	-3,555323	0,895374	2,355122
C47	-4,206658	1,346558	-1,873335	C47	-3,979844	1,787782	-1,398754
C48	-5,138986	1,908644	-0,995517	C48	-4,841875	2,333960	-0,441942
H49	-5,728862	2,149065	1,063737	H49	-5,357824	2,424673	1,648854
H50	-4,315444	1,487015	-2,945073	H50	-4,103403	2,036957	-2,448738
H51	-5,970279	2,489610	-1,384446	H51	-5,635153	3,009527	-0,748552
H52	0,809053	0,157331	0,981979	H52	0,968672	-0,141924	1,352492
H53	2,047387	0,863061	-1,374622	H53	2,266332	2,540376	-0,831852
H54	-1,516365	-2,536595	2,830006	H54	-1,601152	-2,898364	2,621524
H55	-2,408008	0,175781	-2,058066	H55	-2,285478	0,505054	-1,748260
H56	2,421253	-4,542872	-1,535173	H56	2,255235	-4,962075	-1,479695
C57	4,834744	-0,651981	-2,701715	C57	4,696218	-1,261266	-3,114600
C58	3,663794	-1,251717	-3,170682	C58	3,509661	-1,896637	-3,490989
C59	2,853897	-1,975094	-2,298984	C59	2,708411	-2,498927	-2,525404
C60	3,191436	-2,109314	-0,940943	C60	3,076157	-2,486679	-1,170520
C61	4,356489	-1,491161	-0,477299	C61	4,253264	-1,827376	-0,799977
C62	5,175415	-0,774653	-1,355161	C62	5,060280	-1,224668	-1,769188
H63	5,473597	-0,092268	-3,378792	H63	5,336226	-0,806461	-3,866172
H64	3,382810	-1,163289	-4,216768	H64	3,206374	-1,926867	-4,533200
H65	1,932889	-2,413621	-2,671358	H65	1,780565	-2,973331	-2,832966
H66	4,630280	-1,561623	0,568838	H66	4,546510	-1,782760	0,241930
H67	6,082135	-0,309267	-0,978912	H67	5,976253	-0,725560	-1,465943
C68	2,660470	5,313225	-1,876282	C68	-0,752253	5,308402	-2,843922
C69	1,819634	4,204236	-1,916397	C69	-0,260874	4,033248	-2,573675
C70	2,342395	2,916028	-1,740419	C70	0,831014	3,860769	-1,713484
C71	3,711113	2,753727	-1,514358	C71	1,422272	4,981887	-1,127836
C72	4,552174	3,867034	-1,465784	C72	0,926473	6,259920	-1,391208
C73	4,028538	5,146673	-1,648049	C73	-0,160134	6,425780	-2,249302
H74	2,246482	6,308930	-2,005749	H74	-1,599398	5,432459	-3,512889
H75	0,750667	4,338862	-2,052288	H75	-0,731962	3,162429	-3,025384
H76	4,117708	1,756544	-1,369740	H76	2,256624	4,852811	-0,442675

H77	5,614189	3,734129	-1,280425	H77	1,387934	7,124467	-0,922774
H78	4,681263	6,013712	-1,603976	H78	-0,546675	7,420000	-2,454012
C79	1,422904	1,711106	-1,783340	C79	1,299681	2,461003	-1,338421
C80	0,097319	1,789934	-0,975761	C80	0,252126	1,828277	-0,369658
C81	1,327522	2,153929	-4,362892	C81	2,379657	2,255228	-3,635790
C82	0,394676	0,227534	-3,477145	C82	0,942216	0,548706	-2,997785
C83	0,951166	1,156350	-5,473193	C83	2,261227	1,192754	-4,746260
H84	2,356316	2,509075	-4,441496	H84	3,408553	2,387213	-3,286018
H85	0,661688	3,024430	-4,327564	H85	1,986055	3,230554	-3,935077
H86	0,329850	1,584084	-6,260358	H86	1,932439	1,598781	-5,704309
H87	1,830817	0,678451	-5,914814	H87	3,183567	0,623382	-4,881949
N88	1,145242	1,309095	-3,182027	N88	1,549182	1,676539	-2,574485
O89	0,177093	0,125476	-4,798603	O89	1,245437	0,270825	-4,276384
O90	-0,060548	-0,614869	-2,682659	O90	0,194444	-0,223518	-2,367461
H91	2,359818	-5,120694	0,158207	H91	1,727602	-5,312094	0,193050
N92	-2,022513	-2,224649	0,307754	N92	-2,049981	-2,086587	0,189963
C93	-1,423224	-3,048065	-0,771947	C93	-1,490750	-2,779336	-0,995755
H94	-1,477038	-2,548494	-1,749471	H94	-1,447903	-2,124893	-1,876957
H96	-0,729762	1,934787	-1,677822	H96	-0,704380	1,951594	-0,883318
C97	-0,414768	4,575743	2,278842	C97	0,021719	4,188704	3,252687
C98	-1,416475	4,395741	1,324955	C98	-1,028026	4,195892	2,333744
C99	-1,221674	3,512891	0,260294	C99	-0,939815	3,444468	1,160010
C100	-0,030623	2,781645	0,135076	C100	0,188995	2,658177	0,889353
C101	0,968399	2,972693	1,105483	C101	1,239919	2,668108	1,819706
C102	0,781599	3,861168	2,159571	C102	1,161865	3,424918	2,986472
H103	-0,560455	5,266837	3,104342	H103	-0,043594	4,775173	4,164909
H104	-2,351666	4,943510	1,405705	H104	-1,916820	4,790886	2,526293
H105	-2,012523	3,372766	-0,473041	H105	-1,756673	3,465031	0,443840
H106	1,897454	2,415045	1,033031	H106	2,114056	2,050170	1,636152
H107	1,570145	3,995205	2,895200	H107	1,986454	3,413178	3,694376
				H108	3,293888	0,440742	-0,341385
				H109	3,385246	0,864873	0,262884

E3				TS_{E3-4}			
C1	-4,355556	-2,461423	-0,796930	C1	-4,413515	-2,302062	-0,638785
C2	-2,464517	-3,925156	-1,300068	C2	-2,573687	-3,727476	-1,382061
C3	-3,708816	-3,278811	-1,965573	C3	-3,831807	-2,993428	-1,917442
C4	-3,387604	-2,750304	0,372202	C4	-3,398989	-2,726113	0,447706
H5	-5,361223	-2,822084	-0,557254	H5	-5,411763	-2,679139	-0,393277
H6	-3,440042	-2,652892	-2,822424	H6	-3,586142	-2,279212	-2,710042
H7	-3,760128	-2,490877	1,363835	H7	-3,723856	-2,569196	1,476909
H8	-4,424135	-1,392214	-1,005325	H8	-4,479944	-1,216799	-0,729393
H9	-2,031946	-4,762519	-1,847532	H9	-2,182898	-4,507789	-2,035118
H10	-4,384329	-4,058292	-2,329190	H10	-4,536185	-3,717304	-2,336824
C11	-2,991084	-4,215905	0,123019	C11	-3,042881	-4,161916	0,025095
H12	-2,224535	-4,581682	0,813192	H12	-2,253650	-4,608941	0,636961
H13	-3,844385	-4,899983	0,127859	H13	-3,907675	-4,830721	-0,005156
C14	-0,041523	-3,216597	-0,678653	C14	-0,120623	-3,128987	-0,781702

N15	0,855807	-2,435612	-0,188945	N15	0,842850	-2,407734	-0,317074
C16	2,150333	-3,183753	-0,098660	C16	2,093723	-3,245358	-0,251711
C17	1,693364	-4,631656	-0,500993	C17	1,513592	-4,657923	-0,600204
P18	-1,544302	-0,606122	0,700196	P18	-1,514687	-0,591532	0,806256
Ir19	0,592166	-0,231970	-0,063785	Ir19	0,759673	-0,294219	-0,009525
C20	-1,546094	-0,756007	2,518475	C20	-1,527905	-0,749885	2,623980
C21	-1,268381	-0,974106	5,295448	C21	-1,192945	-0,974831	5,402275
C22	-1,613054	-2,010979	3,136475	C22	-1,582486	-2,005246	3,244667
C23	-1,320531	0,390585	3,298644	C23	-1,289125	0,393669	3,406372
C24	-1,186329	0,277843	4,679770	C24	-1,127005	0,277702	4,785551
C25	-1,480049	-2,116059	4,522682	C25	-1,420279	-2,113807	4,628231
H26	-1,228715	1,365464	2,832543	H26	-1,211470	1,371615	2,941757
H27	-1,006549	1,170753	5,271393	H27	-0,939308	1,169871	5,376001
H28	-1,534139	-3,092813	4,994983	H28	-1,468306	-3,092136	5,098143
H29	-1,161769	-1,058831	6,373224	H29	-1,062974	-1,061784	6,476900
O30	0,315250	-4,477679	-0,946080	O30	0,153901	-4,405804	-1,055895
C31	2,692002	-3,204161	1,325974	C31	2,656906	-3,306900	1,165422
C32	1,933285	-2,734603	2,402212	C32	1,851683	-2,996121	2,267468
C33	3,925829	-3,819134	1,584584	C33	3,933919	-3,837038	1,392778
C34	2,414434	-2,841554	3,707916	C34	2,332424	-3,153284	3,566629
H35	0,964536	-2,285964	2,228755	H35	0,845660	-2,624832	2,118836
C36	4,404621	-3,928758	2,888826	C36	4,412205	-4,001835	2,692640
H37	4,519070	-4,210503	0,762383	H37	4,562518	-4,118616	0,553695
C38	3,652706	-3,432261	3,955601	C38	3,617779	-3,649556	3,784303
H39	1,808509	-2,464015	4,527096	H39	1,692948	-2,885840	4,403490
H40	5,364893	-4,402755	3,070793	H40	5,408485	-4,405297	2,849429
H41	4,028208	-3,514824	4,971468	H41	3,995484	-3,770533	4,795471
C42	-2,833127	0,613549	0,264873	C42	-2,782174	0,663567	0,374632
C43	-3,739434	1,151767	1,185436	C43	-3,678867	1,242747	1,280670
C44	-2,932165	0,965633	-1,093502	C44	-2,844866	1,033951	-0,982416
C45	-4,723273	2,044848	0,755652	C45	-4,605088	2,192258	0,842814
H46	-3,681164	0,882844	2,234835	H46	-3,651992	0,963920	2,329021
C47	-3,915922	1,855544	-1,516099	C47	-3,771994	1,980211	-1,415246
C48	-4,810581	2,400715	-0,589921	C48	-4,650489	2,568971	-0,500624
H49	-5,421157	2,461786	1,476048	H49	-5,292730	2,638111	1,555837
H50	-3,985367	2,127252	-2,565685	H50	-3,805810	2,262920	-2,463592
H51	-5,575048	3,098789	-0,918751	H51	-5,368613	3,311869	-0,834699
H52	0,938347	-0,159584	1,456331	H52	1,201376	-0,519862	1,467952
H53	2,237083	2,572303	-0,914469	H53	2,235772	2,842658	-0,820397
H54	-1,742447	-2,906701	2,538588	H54	-1,725901	-2,899988	2,647340
H55	-2,232119	0,545014	-1,811352	H55	-2,159470	0,583233	-1,697304
H56	2,268557	-5,060674	-1,322576	H56	2,053272	-5,173120	-1,394721
C57	4,568871	-1,291076	-3,176957	C57	4,634502	-1,691483	-3,416030
C58	3,404767	-1,992444	-3,500726	C58	3,491573	-2,436607	-3,713740
C59	2,670007	-2,620247	-2,498426	C59	2,705712	-2,947164	-2,683056
C60	3,079091	-2,565381	-1,157038	C60	3,042282	-2,725863	-1,340225
C61	4,241555	-1,853755	-0,842254	C61	4,169474	-1,947620	-1,053712
C62	4,982539	-1,223934	-1,846404	C62	4,964774	-1,442494	-2,083470

H63	5,154549	-0,811033	-3,956473	H63	5,265520	-1,314213	-4,216714
H64	3,065233	-2,051321	-4,530629	H64	3,211678	-2,623443	-4,746369
H65	1,754383	-3,139489	-2,767786	H65	1,809273	-3,506118	-2,938483
H66	4,568313	-1,776593	0,188657	H66	4,426158	-1,727026	-0,024981
H67	5,887109	-0,682849	-1,583338	H67	5,845499	-0,854931	-1,839697
C68	-0,941122	5,302451	-2,740422	C68	-1,239374	4,946582	-2,928325
C69	-0,431422	4,033568	-2,472621	C69	-0,551899	3,791548	-2,561081
C70	0,727766	3,879871	-1,700349	C70	0,578611	3,869342	-1,737125
C71	1,364559	5,016680	-1,197934	C71	1,010644	5,118463	-1,287847
C72	0,851294	6,289036	-1,459581	C72	0,321163	6,276717	-1,653362
C73	-0,300712	6,436348	-2,232445	C73	-0,804963	6,194623	-2,473050
H74	-1,839673	5,408351	-3,342105	H74	-2,114219	4,874586	-3,568649
H75	-0,939400	3,152935	-2,860069	H75	-0,897795	2,821789	-2,911454
H76	2,254152	4,906666	-0,582773	H76	1,878616	5,186070	-0,636870
H77	1,352876	7,164651	-1,057594	H77	0,664912	7,243025	-1,295989
H78	-0,698665	7,425733	-2,437694	H78	-1,340163	7,095563	-2,757880
C79	1,231840	2,485709	-1,345623	C79	1,274027	2,589293	-1,281250
C80	0,279531	1,841264	-0,299986	C80	0,372635	1,899074	-0,228570
C81	2,151619	2,261051	-3,712667	C81	2,400094	2,320958	-3,538157
C82	0,782285	0,546597	-2,957598	C82	1,014190	0,596770	-2,844703
C83	2,014814	1,157578	-4,780137	C83	2,468118	1,132737	-4,513051
H84	3,191369	2,438017	-3,419097	H84	3,385466	2,620698	-3,168950
H85	1,711908	3,213123	-4,023323	H85	1,902296	3,199144	-3,963897
H86	1,646053	1,522587	-5,740083	H86	2,285224	1,405084	-5,553185
H87	2,942325	0,598696	-4,927832	H87	3,403706	0,573880	-4,427801
N88	1,393658	1,692068	-2,593554	N88	1,591135	1,753662	-2,455804
O89	1,034454	0,235540	-4,241015	O89	1,400449	0,249112	-4,082562
O90	0,078574	-0,220276	-2,272604	O90	0,219975	-0,126608	-2,215423
H91	1,690070	-5,312458	0,351887	H91	1,447322	-5,296437	0,281753
N92	-2,080084	-2,087632	0,108145	N92	-2,087663	-2,064249	0,206832
C93	-1,446616	-2,787698	-1,033627	C93	-1,525662	-2,638929	-1,042371
H94	-1,346212	-2,138015	-1,912527	H94	-1,450423	-1,900535	-1,850148
H96	-0,716715	1,933407	-0,735762	H96	-0,597644	1,756589	-0,693654
C97	0,235005	4,156271	3,364446	C97	-0,236548	4,407980	3,247056
C98	-0,859212	4,173709	2,498619	C98	-1,216081	4,311537	2,257278
C99	-0,829433	3,439081	1,310489	C99	-1,010305	3,503095	1,138514
C100	0,285664	2,660352	0,968642	C100	0,175897	2,770876	0,994377
C101	1,384234	2,665596	1,842038	C101	1,155033	2,879072	1,991695
C102	1,363454	3,404076	3,023469	C102	0,954964	3,691472	3,106647
H103	0,214639	4,726860	4,288540	H103	-0,398089	5,034912	4,119123
H104	-1,739287	4,762289	2,744004	H104	-2,146081	4,864790	2,353170
H105	-1,684065	3,464982	0,640713	H105	-1,775623	3,439963	0,371914
H106	2,255799	2,060338	1,605442	H106	2,075091	2,302378	1,912977
H107	2,225188	3,385879	3,685470	H107	1,724494	3,756468	3,870542
H108	2,247137	-0,082602	-0,743504	H108	1,441819	1,010523	0,528848
H109	2,300523	0,156327	0,049631	H109	2,296573	-0,250062	-0,468067

C1_E_S_tN_Chelbot

E4				ES_Chelbot_SM			
C1	-4,387460	-2,211852	-0,807837	C1	-3,836737	-3,471497	-1,259336
C2	-2,560348	-3,673862	-1,510030	C2	-1,575812	-4,394314	-1,183623
C3	-3,781732	-2,902100	-2,076284	C3	-2,779710	-4,212660	-2,145241
C4	-3,428396	-2,686235	0,307894	C4	-3,076036	-3,299379	0,073748
H5	-5,407566	-2,555375	-0,607275	H5	-4,735065	-4,078844	-1,107657
H6	-3,493339	-2,185626	-2,852141	H6	-2,506930	-3,647510	-3,042214
H7	-3,790207	-2,539275	1,326188	H7	-3,689116	-3,013060	0,929069
H8	-4,408994	-1,122843	-0,879941	H8	-4,148384	-2,510838	-1,671522
H9	-2,163541	-4,451804	-2,162495	H9	-0,850984	-5,148978	-1,489145
H10	-4,489920	-3,603704	-2,526086	H10	-3,146271	-5,189082	-2,473613
C11	-3,091201	-4,122596	-0,128994	C11	-2,285763	-4,615981	0,171169
H12	-2,335354	-4,599039	0,502410	H12	-1,602327	-4,654871	1,024821
H13	-3,969194	-4,770443	-0,204618	H13	-2,925090	-5,503106	0,175121
C14	-0,115842	-3,133136	-0,819463	C14	0,397301	-2,993957	-0,274591
N15	0,855719	-2,422475	-0,344808	N15	0,994784	-1,926316	0,128865
C16	2,088143	-3,287961	-0,208621	C16	2,361894	-2,307373	0,663661
C17	1,496230	-4,686332	-0,596486	C17	2,212405	-3,860688	0,717650
P18	-1,463684	-0,638096	0,799795	P18	-1,887688	-0,707420	0,306425
Ir19	0,764257	-0,393387	-0,081898	Ir19	0,360292	0,089759	-0,263447
C20	-1,434252	-0,882337	2,610002	C20	-2,135881	-0,625658	2,115336
C21	-1,050883	-1,224185	5,370405	C21	-2,235048	-0,404548	4,909137
C22	-1,659851	-2,128835	3,206427	C22	-2,068036	-1,771869	2,918547
C23	-1,001241	0,192121	3,407457	C23	-2,237870	0,635968	2,725764
C24	-0,816415	0,020691	4,777355	C24	-2,290180	0,742376	4,113113
C25	-1,471666	-2,295649	4,581704	C25	-2,120844	-1,658999	4,309610
H26	-0,791768	1,158814	2,956523	H26	-2,264456	1,536426	2,124824
H27	-0,479380	0,858597	5,381073	H27	-2,365927	1,725373	4,568829
H28	-1,649047	-3,267840	5,033054	H28	-2,065328	-2,553441	4,923345
H29	-0,902197	-1,357017	6,438074	H29	-2,274006	-0,319762	5,991346
O30	0,140693	-4,417845	-1,058913	O30	1,019899	-4,156116	-0,068915
C31	2,575370	-3,374404	1,235853	C31	2,597816	-1,790388	2,078822
C32	1,738916	-3,034309	2,303963	C32	1,526073	-1,419158	2,897940
C33	3,818086	-3,961689	1,513001	C33	3,889310	-1,804741	2,623743
C34	2,157313	-3,219027	3,621350	C34	1,743895	-1,008563	4,212969
H35	0,757704	-2,618897	2,117960	H35	0,515500	-1,440064	2,513029
C36	4,233500	-4,153228	2,830433	C36	4,107174	-1,400935	3,940909
H37	4,468741	-4,266833	0,698631	H37	4,731903	-2,119531	2,015629
C38	3,409275	-3,771222	3,889778	C38	3,036921	-0,987775	4,735983
H39	1,494281	-2,928672	4,431452	H39	0,894925	-0,707890	4,820268
H40	5,203891	-4,600364	3,026734	H40	5,116374	-1,406836	4,342766
H41	3,738409	-3,913053	4,915210	H41	3,210579	-0,663028	5,757954
C42	-2,695400	0,694027	0,507896	C42	-3,389500	0,037719	-0,447270
C43	-3,510995	1,248321	1,502547	C43	-4,450416	0,582424	0,285180
C44	-2,802919	1,173814	-0,810901	C44	-3,453890	0,047937	-1,852055
C45	-4,405507	2,274103	1,188812	C45	-5,538757	1,159076	-0,374869
H46	-3,443706	0,891041	2,524994	H46	-4,431403	0,570788	1,369333
C47	-3,698230	2,196855	-1,119891	C47	-4,537761	0,627590	-2,507199

C48	-4,498141	2,755788	-0,118213
H49	-5,029501	2,698374	1,970489
H50	-3,761413	2,569191	-2,137926
H51	-5,189586	3,558702	-0,356404
H52	1,334877	-0,565343	1,349212
H53	2,149311	3,251278	-1,021340
H54	-1,951088	-2,978885	2,598262
H55	-2,173458	0,752234	-1,591435
H56	2,038645	-5,185579	-1,399547
C57	4,834472	-1,733562	-3,191210
C58	3,691967	-2,441800	-3,568945
C59	2,840907	-2,960146	-2,595211
C60	3,111117	-2,781081	-1,232241
C61	4,239823	-2,039708	-0,864913
C62	5,100784	-1,529550	-1,836827
H63	5,514478	-1,348777	-3,946903
H64	3,463428	-2,594027	-4,619719
H65	1,944476	-3,487728	-2,911461
H66	4,442397	-1,845599	0,181491
H67	5,980021	-0,969166	-1,531299
C68	-1,619221	4,680817	-3,163111
C69	-0,772133	3,659344	-2,735448
C70	0,327739	3,948895	-1,918474
C71	0,564092	5,270574	-1,531943
C72	-0,288416	6,292483	-1,953748
C73	-1,381457	6,000909	-2,770938
H74	-2,465959	4,447639	-3,802926
H75	-0,972236	2,632587	-3,030194
H76	1,407548	5,500227	-0,885605
H77	-0,096951	7,315657	-1,643653
H78	-2,042679	6,796158	-3,102243
C79	1,213216	2,823567	-1,397356
C80	0,505030	2,121767	-0,211166
C81	2,505019	2,394979	-3,524387
C82	1,012506	0,747479	-2,861258
C83	2,672634	1,127555	-4,376003
H84	3,449881	2,743866	-3,097502
H85	2,035246	3,223588	-4,068778
H86	2,639344	1,308128	-5,450906
H87	3,572152	0,564111	-4,113036
N88	1,608002	1,904113	-2,475788
O89	1,530593	0,306335	-4,018970
O90	0,105644	0,116493	-2,294220
H91	1,418863	-5,346297	0,268038
N92	-2,097414	-2,050395	0,126010
C93	-1,503158	-2,612989	-1,115709
H94	-1,388952	-1,858094	-1,903955
H96	-0,467384	1,790031	-0,549387

C48	-5,580224	1,198237	-1,768330
H49	-6,353853	1,580232	0,206638
H50	-4,575811	0,622694	-3,593265
H51	-6,425218	1,652493	-2,277076
H52	0,608897	0,404722	1,229184
H53	1,928447	0,164483	-0,493086
H54	-1,945313	-2,749349	2,464864
H55	-2,663078	-0,416282	-2,432207
H56	3,053669	-4,399567	0,282697
C57	5,053766	-0,914149	-2,449808
C58	4,295305	-2,073232	-2,626408
C59	3,468129	-2,530832	-1,603228
C60	3,386346	-1,846427	-0,382129
C61	4,145640	-0,679903	-0,219943
C62	4,974457	-0,219299	-1,244106
H63	5,699427	-0,556992	-3,247079
H64	4,344835	-2,619914	-3,563588
H65	2,867147	-3,419729	-1,776360
H66	4,088834	-0,114113	0,700613
H67	5,551694	0,688682	-1,093785
C68	-1,304366	4,199888	2,244393
C69	-0,506681	3,450245	1,382024
C70	-1,034630	2,920125	0,190256
C71	-2,392371	3,156656	-0,086778
C72	-3,189872	3,900135	0,783879
C73	-2,651271	4,432892	1,954603
H74	-0,865607	4,601276	3,153717
H75	0,522738	3,272204	1,651035
H76	-2,844845	2,726444	-0,972318
H77	-4,238464	4,049667	0,543237
H78	-3,268447	5,017175	2,630616
C79	-0,212417	2,109027	-0,765035
C80	1,236053	1,998019	-0,778653
C81	-0,935934	3,245910	-2,968553
C82	-0,604715	0,968105	-2,897709
C83	-1,484582	2,573157	-4,233728
H84	0,035666	3,732796	-3,131542
H85	-1,626170	3,968899	-2,532463
H86	-2,576900	2,505584	-4,216071
H87	-1,148363	3,022190	-5,168140
N88	-0,777338	2,062452	-2,113428
O89	-0,953693	1,217123	-4,168339
O90	-0,188645	-0,141709	-2,532029
H91	2,025618	-4,212674	1,733630
N92	-1,955636	-2,335852	-0,120541
C93	-0,939826	-2,999620	-0,973852
H94	-0,797152	-2,470932	-1,924990
H96	1,632593	1,919787	-1,792671

C97	0,042285	4,614948	3,271978	C97	4,106730	4,162268	1,598419
C98	-1,019338	4,449636	2,380960	C98	3,600068	4,678341	0,404511
C99	-0,869644	3,643445	1,252427	C99	2,670158	3,947225	-0,332235
C100	0,346525	2,995341	1,008341	C100	2,223482	2,693929	0,112234
C101	1,407988	3,162052	1,906104	C101	2,742664	2,181849	1,308224
C102	1,258170	3,969559	3,032783	C102	3,675540	2,912540	2,044777
H103	-0,077135	5,240585	4,152132	H103	4,833841	4,729271	2,172496
H104	-1,968753	4,944422	2,564869	H104	3,929354	5,649145	0,044810
H105	-1,697485	3,515661	0,562448	H105	2,279596	4,355741	-1,261870
H106	2,347995	2,641552	1,733005	H106	2,412168	1,214930	1,667817
H107	2,085266	4,089246	3,726858	H107	4,065613	2,496102	2,969302
H108	1,259546	1,308447	0,135352				
H109	2,294829	-0,311753	-0,568377				

C1_ES_Chelbot_TS1				C1_ES_Chelbot_INT1			
C1	-3,890112	-3,534552	-1,110363	C1	-3,922167	-3,535557	-0,845760
C2	-1,631229	-4,463929	-1,079330	C2	-1,682248	-4,505975	-0,875384
C3	-2,860492	-4,296359	-2,010860	C3	-2,953609	-4,365892	-1,753156
C4	-3,098485	-3,354119	0,203868	C4	-3,070026	-3,317475	0,424945
H5	-4,791446	-4,130297	-0,932523	H5	-4,830153	-4,096773	-0,602200
H6	-2,611115	-3,749735	-2,925971	H6	-2,742784	-3,875484	-2,708887
H7	-3,692147	-3,060030	1,070139	H7	-3,621589	-2,980046	1,302946
H8	-4,200129	-2,574781	-1,526957	H8	-4,225582	-2,587775	-1,293789
H9	-0,912709	-5,220574	-1,394775	H9	-0,986636	-5,283712	-1,190540
H10	-3,239313	-5,277707	-2,309765	H10	-3,364253	-5,354445	-1,977010
C11	-2,305924	-4,670014	0,295949	C11	-2,293896	-4,641574	0,537149
H12	-1,601790	-4,695968	1,133361	H12	-1,553521	-4,641675	1,343164
H13	-2,943691	-5,557847	0,326556	H13	-2,942847	-5,516412	0,634798
C14	0,375664	-3,038611	-0,272659	C14	0,392823	-3,057206	-0,284548
N15	0,964758	-1,964872	0,122388	N15	0,973673	-1,977302	0,101590
C16	2,361157	-2,312735	0,580526	C16	2,391639	-2,285109	0,467499
C17	2,287653	-3,875147	0,555195	C17	2,451027	-3,830399	0,178239
P18	-1,910208	-0,754765	0,349199	P18	-1,853245	-0,733679	0,405834
Ir19	0,258869	0,046517	-0,229981	Ir19	0,162355	0,023971	-0,226352
C20	-2,166115	-0,608508	2,153143	C20	-2,106193	-0,495120	2,195510
C21	-2,285923	-0,278419	4,932011	C21	-2,224971	-0,020267	4,948810
C22	-2,091489	-1,722022	2,998906	C22	-1,997456	-1,560878	3,096608
C23	-2,282990	0,676374	2,709582	C23	-2,252957	0,815868	2,679828
C24	-2,345880	0,836609	4,091358	C24	-2,315188	1,047145	4,050899
C25	-2,155165	-1,554879	4,384161	C25	-2,063542	-1,321142	4,470965
H26	-2,311218	1,552412	2,071727	H26	-2,299480	1,654485	1,993543
H27	-2,433659	1,835787	4,508617	H27	-2,425078	2,064209	4,415679
H28	-2,096141	-2,423513	5,033743	H28	-1,981091	-2,151921	5,165988
H29	-2,334347	-0,151092	6,009859	H29	-2,273650	0,163365	6,018355
O30	1,043765	-4,187098	-0,141976	O30	1,112447	-4,180872	-0,282738
C31	2,620273	-1,843382	2,009016	C31	2,653678	-2,025228	1,948413
C32	1,561546	-1,505858	2,859452	C32	1,642403	-1,613607	2,820671
C33	3,923944	-1,845388	2,524295	C33	3,933065	-2,277612	2,466512

C34	1,802708	-1,114500	4,176428	C34	1,912668	-1,417012	4,176514
H35	0,543045	-1,534022	2,496000	H35	0,641530	-1,442598	2,449502
C36	4,165740	-1,459895	3,842702	C36	4,202199	-2,084241	3,819623
H37	4,758040	-2,132333	1,890729	H37	4,726595	-2,614502	1,803764
C38	3,107118	-1,078671	4,669629	C38	3,192788	-1,643410	4,678766
H39	0,963357	-0,837997	4,808594	H39	1,110925	-1,089531	4,832750
H40	5,184133	-1,453199	4,220924	H40	5,200432	-2,276392	4,202194
H41	3,299200	-0,767310	5,692646	H41	3,404345	-1,488175	5,732729
C42	-3,390656	-0,008389	-0,442763	C42	-3,313379	-0,014336	-0,432881
C43	-4,477904	0,520503	0,262061	C43	-4,424227	0,501728	0,245164
C44	-3,415408	0,004708	-1,848767	C44	-3,327834	-0,065129	-1,838067
C45	-5,557299	1,080916	-0,426530	C45	-5,521627	0,983296	-0,472164
H46	-4,487374	0,507958	1,346507	H46	-4,441957	0,533224	1,328818
C47	-4,490757	0,567569	-2,532213	C47	-4,422684	0,418417	-2,548940
C48	-5,562904	1,118700	-1,821105	C48	-5,520833	0,951413	-1,865920
H49	-6,394213	1,489442	0,132900	H49	-6,378083	1,382686	0,063519
H50	-4,498692	0,566789	-3,618943	H50	-4,425781	0,369922	-3,634506
H51	-6,401826	1,559208	-2,351904	H51	-6,375877	1,328324	-2,419691
H52	0,457604	0,364248	1,272720	H52	0,358663	0,395830	1,270501
H53	1,838351	0,335246	-0,465687	H53	1,871495	0,851245	-0,692610
H54	-1,956438	-2,714707	2,583102	H54	-1,834992	-2,569632	2,732095
H55	-2,598603	-0,440567	-2,407009	H55	-2,488276	-0,498212	-2,371281
H56	3,109171	-4,345687	0,014995	H56	3,155528	-4,097031	-0,611527
C57	4,816541	-0,608844	-2,574212	C57	4,704679	0,044809	-2,381224
C58	4,066745	-1,766983	-2,793793	C58	3,824536	-0,953112	-2,809206
C59	3,323707	-2,326082	-1,757026	C59	3,120652	-1,706632	-1,874740
C60	3,317342	-1,747101	-0,479742	C60	3,283051	-1,485582	-0,496839
C61	4,069059	-0,583788	-0,272697	C61	4,162720	-0,481686	-0,080571
C62	4,814453	-0,020288	-1,310833	C62	4,868908	0,277489	-1,017569
H63	5,395670	-0,171067	-3,382532	H63	5,255310	0,635584	-3,107678
H64	4,056111	-2,231644	-3,775444	H64	3,681512	-1,139944	-3,869559
H65	2,723676	-3,209141	-1,961149	H65	2,414839	-2,454559	-2,226424
H66	4,069425	-0,096633	0,693143	H66	4,291531	-0,269082	0,972870
H67	5,384835	0,885651	-1,124589	H67	5,537115	1,060542	-0,671961
C68	-1,312077	4,269219	2,125774	C68	-1,293136	4,392223	1,919171
C69	-0,481794	3,501711	1,310432	C69	-0,432849	3,588225	1,170940
C70	-0,972984	2,923672	0,126520	C70	-0,869760	2,960597	-0,006787
C71	-2,326072	3,134474	-0,194809	C71	-2,211631	3,160841	-0,386631
C72	-3,154931	3,896090	0,627310	C72	-3,072504	3,957567	0,367018
C73	-2,652283	4,473680	1,792664	C73	-2,618370	4,586561	1,527293
H74	-0,903382	4,708587	3,031712	H74	-0,917234	4,866891	2,821494
H75	0,542235	3,349637	1,612343	H75	0,574745	3,443768	1,527002
H76	-2,747985	2,667276	-1,076987	H76	-2,602182	2,651659	-1,260435
H77	-4,198644	4,025099	0,355404	H77	-4,104223	4,073485	0,046179
H78	-3,294276	5,071701	2,433071	H78	-3,283940	5,212161	2,114844
C79	-0,141191	2,059050	-0,773661	C79	-0,035033	2,001046	-0,815950
C80	1,325181	1,901088	-0,730143	C80	1,528273	1,952514	-0,804268
C81	-0,738153	3,220427	-3,014941	C81	-0,493325	3,152482	-3,120438

C82	-0,506030	0,928816	-2,912554	C82	-0,366617	0,858869	-2,949818
C83	-1,264999	2,554987	-4,294921	C83	-0,967209	2,474263	-4,416301
H84	0,249749	3,682628	-3,149341	H84	0,511373	3,588726	-3,211370
H85	-1,424039	3,962468	-2,603526	H85	-1,180186	3,920758	-2,762166
H86	-2,359110	2,534707	-4,321333	H86	-2,056776	2,502854	-4,517208
H87	-0,874589	2,979665	-5,219596	H87	-0,498017	2,854180	-5,323827
N88	-0,639548	2,040288	-2,149315	N88	-0,472891	1,992406	-2,224088
O89	-0,796713	1,177204	-4,197871	O89	-0,573248	1,078384	-4,255755
O90	-0,166263	-0,196571	-2,512724	O90	-0,111426	-0,273208	-2,497675
H91	2,211398	-4,293652	1,559984	H91	2,653979	-4,414920	1,075975
N92	-1,979476	-2,397273	-0,019156	N92	-1,941224	-2,393571	0,121692
C93	-0,994454	-3,064104	-0,905874	C93	-1,024587	-3,105439	-0,800368
H94	-0,894851	-2,547034	-1,869506	H94	-0,992803	-2,635734	-1,793755
H96	1,754188	1,850258	-1,733389	H96	1,890278	2,146172	-1,819924
C97	4,039952	4,049582	1,832525	C97	3,962379	4,173220	1,975169
C98	3,634125	4,563725	0,599762	C98	3,602111	4,725439	0,744703
C99	2,760803	3,835634	-0,206261	C99	2,815826	3,994281	-0,145069
C100	2,271669	2,589590	0,210919	C100	2,375330	2,708806	0,187351
C101	2,690171	2,076926	1,444958	C101	2,746334	2,156933	1,419282
C102	3,568065	2,804094	2,248849	C102	3,533377	2,886670	2,309082
H103	4,721350	4,615800	2,460738	H103	4,577018	4,741068	2,667633
H104	3,996747	5,531341	0,264487	H104	3,934321	5,724264	0,476356
H105	2,444894	4,242523	-1,164574	H105	2,536109	4,428420	-1,102477
H106	2,321234	1,115366	1,782109	H106	2,409950	1,159499	1,684405
H107	3,881413	2,389230	3,202543	H107	3,813598	2,446717	3,262111

C1_ES_Chelbot_TS2				C1_ES_Chelbot_INT2			
C1	-3,650305	-3,586452	-1,241988	C1	-3,667825	-3,630981	-1,228670
C2	-1,338706	-4,369441	-1,173943	C2	-1,348608	-4,386477	-1,123571
C3	-2,561092	-4,280839	-2,125448	C3	-2,561129	-4,325891	-2,089436
C4	-2,881591	-3,320390	0,069718	C4	-2,915992	-3,337030	0,087052
H5	-4,498515	-4,251430	-1,048284	H5	-4,510983	-4,302555	-1,035418
H6	-2,331831	-3,717474	-3,035668	H6	-2,327993	-3,774555	-3,005954
H7	-3,499735	-3,042241	0,923327	H7	-3,547028	-3,054098	0,929832
H8	-4,042814	-2,667627	-1,677501	H8	-4,064352	-2,722299	-1,681437
H9	-0,576787	-5,090752	-1,467999	H9	-0,576528	-5,104267	-1,399279
H10	-2,872577	-5,283084	-2,431975	H10	-2,857415	-5,336666	-2,383267
C11	-2,018488	-4,588765	0,194891	C11	-2,041194	-4,594271	0,240845
H12	-1,321679	-4,571294	1,038206	H12	-1,354692	-4,556401	1,092076
H13	-2,612162	-5,506219	0,234050	H13	-2,623771	-5,518616	0,285708
C14	0,563521	-2,848393	-0,317652	C14	0,541263	-2,842588	-0,285806
N15	1,066515	-1,758079	0,145894	N15	1,050429	-1,751769	0,170334
C16	2,426403	-2,073775	0,726181	C16	2,411576	-2,050986	0,735784
C17	2,525042	-3,620846	0,452218	C17	2,521310	-3,597967	0,457111
P18	-1,822959	-0,694314	0,327291	P18	-1,893270	-0,690939	0,318551
Ir19	0,140518	0,230819	-0,282948	Ir19	0,193972	0,247753	-0,286031
C20	-2,047449	-0,716779	2,138345	C20	-2,101447	-0,686860	2,136290
C21	-2,265128	-0,681916	4,931522	C21	-2,305027	-0,609038	4,931207

C22	-1,900591	-1,901155	2,873567	C22	-1,967284	-1,861006	2,888711
C23	-2,283428	0,488321	2,816920	C23	-2,313545	0,532084	2,798446
C24	-2,393678	0,503574	4,204604	C24	-2,416844	0,567976	4,186990
C25	-2,016462	-1,881495	4,263482	C25	-2,074431	-1,820493	4,279495
H26	-2,376489	1,415156	2,268935	H26	-2,391479	1,454238	2,238376
H27	-2,574237	1,445204	4,714993	H27	-2,580896	1,519488	4,684602
H28	-1,895201	-2,803562	4,824371	H28	-1,964736	-2,736621	4,852600
H29	-2,351672	-0,670895	6,014354	H29	-2,388260	-0,580602	6,013990
O30	1,274375	-3,969402	-0,201321	O30	1,264423	-3,957179	-0,179555
C31	2,484549	-1,818452	2,231097	C31	2,484058	-1,804271	2,241722
C32	1,434164	-1,267911	2,962645	C32	1,429094	-1,258855	2,972583
C33	3,642894	-2,220551	2,917874	C33	3,641077	-2,209107	2,927661
C34	1,541047	-1,102907	4,346600	C34	1,532206	-1,096807	4,356498
H35	0,529187	-0,965066	2,462850	H35	0,519619	-0,965553	2,473032
C36	3,753482	-2,052980	4,294891	C36	3,745402	-2,048587	4,306629
H37	4,473781	-2,654762	2,367637	H37	4,472162	-2,644142	2,377984
C38	2,699365	-1,488051	5,016939	C38	2,689383	-1,485091	5,027158
H39	0,705529	-0,673753	4,890985	H39	0,693320	-0,673756	4,900751
H40	4,660942	-2,365142	4,803848	H40	4,650409	-2,364096	4,817885
H41	2,782321	-1,357183	6,092093	H41	2,770053	-1,358941	6,103013
C42	-3,404295	-0,073061	-0,366221	C42	-3,494091	-0,088258	-0,358760
C43	-4,459636	0,406910	0,419526	C43	-4,543050	0,398174	0,430846
C44	-3,577133	-0,170765	-1,757214	C44	-3,682519	-0,188532	-1,748070
C45	-5,652237	0,815585	-0,179074	C45	-5,740956	0,808633	-0,158935
H46	-4,367942	0,458161	1,497162	H46	-4,438416	0,462273	1,507323
C47	-4,767950	0,238507	-2,350571	C47	-4,877671	0,223313	-2,333728
C48	-5,805962	0,745849	-1,562382	C48	-5,909991	0,735923	-1,540774
H49	-6,461904	1,185751	0,443037	H49	-6,543170	1,183473	0,470236
H50	-4,895209	0,141859	-3,425338	H50	-5,012023	0,123501	-3,407515
H51	-6,735729	1,064533	-2,024556	H51	-6,842909	1,054844	-1,995864
H52	0,426716	0,674167	1,182408	H52	0,365066	0,684781	1,203127
H53	1,213594	2,719704	-2,168545	H53	1,258609	2,637417	-2,208456
H54	-1,667540	-2,831993	2,371920	H54	-1,750809	-2,802608	2,399629
H55	-2,798473	-0,608262	-2,372083	H55	-2,908530	-0,622366	-2,371948
H56	3,338924	-3,887404	-0,223646	H56	3,328707	-3,857095	-0,229944
C57	5,405154	-0,025842	-1,682206	C57	5,315983	0,083749	-1,708907
C58	4,498827	-0,919771	-2,259289	C58	4,382249	-0,784697	-2,283656
C59	3,553345	-1,560772	-1,463797	C59	3,473548	-1,464851	-1,476530
C60	3,490666	-1,322050	-0,081959	C60	3,461690	-1,281469	-0,083770
C61	4,385196	-0,408945	0,481979	C61	4,380934	-0,392404	0,479157
C62	5,345118	0,224225	-0,312992	C62	5,314018	0,267262	-0,327779
H63	6,153177	0,467092	-2,296485	H63	6,036680	0,604917	-2,331797
H64	4,531409	-1,118643	-3,326671	H64	4,367594	-0,938371	-3,358858
H65	2,852872	-2,249858	-1,929367	H65	2,753244	-2,133499	-1,941444
H66	4,341616	-0,185095	1,541302	H66	4,380101	-0,209390	1,547170
H67	6,039158	0,922821	0,145341	H67	6,039592	0,931012	0,132966
C68	-1,708231	3,874909	2,305937	C68	-1,604444	3,941757	2,271165
C69	-0,916093	3,206987	1,376789	C69	-0,817438	3,281424	1,331314

C70	-1,407634	2,855384	0,105601
C71	-2,758770	3,122226	-0,156161
C72	-3,566779	3,765842	0,786400
C73	-3,043264	4,168415	2,012511
H74	-1,288139	4,140072	3,272666
H75	0,092987	2,923977	1,646266
H76	-3,213191	2,784044	-1,079480
H77	-4,612733	3,944449	0,553383
H78	-3,667794	4,681722	2,737979
C79	-0,465556	2,198501	-0,888494
C80	0,718068	3,195837	-1,316237
C81	-1,611294	3,016398	-3,162003
C82	-0,904734	0,853407	-2,900058
C83	-2,174247	2,123654	-4,271265
H84	-0,773607	3,624654	-3,518881
H85	-2,356904	3,674051	-2,717172
H86	-3,228900	1,882907	-4,108992
H87	-2,024690	2,511207	-5,278865
N88	-1,148301	1,983514	-2,210043
O89	-1,409715	0,889720	-4,142304
O90	-0,303024	-0,157367	-2,481060
H91	2,591048	-4,205527	1,370044
N92	-1,816098	-2,298649	-0,178865
C93	-0,772948	-2,937490	-1,009045
H94	-0,662468	-2,435359	-1,977645
H95	0,190929	4,070891	-1,718362
C96	3,883141	4,849470	1,165850
C97	2,557910	5,228119	1,385219
C98	1,532960	4,702389	0,599380
C99	1,799578	3,768518	-0,414107
C100	3,143746	3,445489	-0,656257
C101	4,173660	3,968888	0,125231
H102	4,680124	5,258241	1,780604
H103	2,318096	5,944828	2,166170
H104	0,519347	5,049690	0,759873
H105	3,386092	2,823360	-1,513493
H106	5,203097	3,696802	-0,092055
H107	2,819279	1,196713	-0,936483
H108	2,611383	0,899730	-1,585830

C1_ES_Chelbot_TS3

C1	-3,687428	-3,668740	-1,267620
C2	-1,367048	-4,416472	-1,152490
C3	-2,569987	-4,344974	-2,129406
C4	-2,953396	-3,403422	0,065073
H5	-4,533364	-4,344078	-1,101193
H6	-2,330437	-3,778407	-3,034973
H7	-3,599036	-3,143257	0,903864

C70	-1,340844	2,870040	0,089846
C71	-2,709334	3,084892	-0,130233
C72	-3,508037	3,726256	0,822209
C73	-2,960138	4,177228	2,021382
H74	-1,158960	4,253618	3,212055
H75	0,214053	3,059932	1,571001
H76	-3,181840	2,707015	-1,028248
H77	-4,566872	3,860000	0,619564
H78	-3,577690	4,685303	2,756068
C79	-0,423112	2,200669	-0,920572
C80	0,763888	3,153339	-1,375943
C81	-1,611731	2,969333	-3,176808
C82	-0,947598	0,798387	-2,876817
C83	-2,228857	2,060141	-4,243783
H84	-0,771596	3,548628	-3,574463
H85	-2,329147	3,653815	-2,725338
H86	-3,282548	1,847043	-4,041971
H87	-2,101622	2,415230	-5,266264
N88	-1,143672	1,951993	-2,212696
O89	-1,488899	0,811922	-4,103280
O90	-0,362764	-0,217232	-2,446833
H91	2,605140	-4,182761	1,373263
N92	-1,862538	-2,305979	-0,162793
C93	-0,800090	-2,945069	-0,972224
H94	-0,687492	-2,456342	-1,947006
H95	0,289901	4,045841	-1,802597
C96	3,813558	4,653363	1,312632
C97	2,584626	5,306426	1,201113
C98	1,608592	4,816989	0,336351
C99	1,825743	3,655514	-0,421688
C100	3,083396	3,042333	-0,337913
C101	4,063169	3,527855	0,529803
H102	4,577037	5,033683	1,985255
H103	2,389201	6,203623	1,781790
H104	0,658570	5,336595	0,251996
H105	3,327115	2,199766	-0,978698
H106	5,028947	3,034393	0,576921
H107	1,766102	0,905772	-0,108723
H108	1,749935	0,617851	-0,922717

C1_ES_Chelbot_PROD

C1	-3,668498	-3,640196	-1,327243
C2	-1,348884	-4,392078	-1,239709
C3	-2,547434	-4,272620	-2,216826
C4	-2,943162	-3,449399	0,023486
H5	-4,518301	-4,319846	-1,204179
H6	-2,304050	-3,661673	-3,092115
H7	-3,598002	-3,236830	0,868555

H8	-4,077587	-2,749719	-1,705134	H8	-4,051825	-2,697125	-1,717171
H9	-0,586433	-5,122722	-1,434761	H9	-0,565011	-5,080350	-1,555409
H10	-2,859116	-5,352324	-2,441811	H10	-2,834646	-5,263213	-2,580285
C11	-2,073377	-4,659283	0,199984	C11	-2,060452	-4,708192	0,096121
H12	-1,396594	-4,634450	1,059333	H12	-1,389326	-4,721618	0,960119
H13	-2,649001	-5,589064	0,216555	H13	-2,630677	-5,640763	0,059782
C14	0,498407	-2,865991	-0,269807	C14	0,512630	-2,884525	-0,283919
N15	1,021788	-1,763271	0,148763	N15	1,065293	-1,796807	0,148525
C16	2,375599	-2,056324	0,751744	C16	2,412885	-2,121786	0,764372
C17	2,430662	-3,620133	0,598191	C17	2,411346	-3,684804	0,620562
P18	-1,909134	-0,750646	0,359581	P18	-1,891068	-0,791254	0,407180
Ir19	0,289021	0,185794	-0,314224	Ir19	0,317679	0,056497	-0,228298
C20	-2,145908	-0,768079	2,174071	C20	-2,134319	-0,806354	2,220584
C21	-2,409055	-0,684076	4,965811	C21	-2,389691	-0,676820	5,010409
C22	-2,106517	-1,950425	2,925569	C22	-2,230674	-1,981153	2,976998
C23	-2,285759	0,459932	2,838697	C23	-2,135527	0,434892	2,878007
C24	-2,421918	0,499979	4,224633	C24	-2,269939	0,498120	4,262803
C25	-2,241255	-1,906163	4,313784	C25	-2,359925	-1,913828	4,365875
H26	-2,284737	1,386587	2,279902	H26	-2,023537	1,352449	2,310469
H27	-2,533279	1,459046	4,722523	H27	-2,273184	1,465273	4,757639
H28	-2,208107	-2,829078	4,885816	H28	-2,432570	-2,831010	4,943433
H29	-2,517665	-0,653759	6,046309	H29	-2,493653	-0,628324	6,090747
O30	1,197551	-3,982760	-0,084106	O30	1,190506	-4,013289	-0,103354
C31	2,445341	-1,718915	2,239428	C31	2,482625	-1,799706	2,256286
C32	1,340549	-1,280092	2,969060	C32	1,345087	-1,510966	3,011922
C33	3,644548	-1,977221	2,923686	C33	3,710524	-1,959292	2,917889
C34	1,435188	-1,069619	4,346941	C34	1,434808	-1,338281	4,394449
H35	0,398415	-1,102562	2,475472	H35	0,382516	-1,416484	2,533050
C36	3,739142	-1,772542	4,297889	C36	3,799360	-1,793770	4,298335
H37	4,513646	-2,333218	2,376924	H37	4,603454	-2,207111	2,350790
C38	2,633118	-1,310480	5,015445	C38	2,661413	-1,473352	5,041447
H39	0,558631	-0,726193	4,888061	H39	0,535108	-1,103628	4,955334
H40	4,676864	-1,974497	4,807791	H40	4,759153	-1,914711	4,792593
H41	2,706881	-1,149009	6,087142	H41	2,732489	-1,339730	6,117192
C42	-3,499164	-0,108409	-0,315476	C42	-3,477749	-0,120284	-0,248902
C43	-4,535405	0,417740	0,465720	C43	-4,510618	0,377337	0,554155
C44	-3,681914	-0,199759	-1,706441	C44	-3,652289	-0,145407	-1,643330
C45	-5,715443	0,868144	-0,132166	C45	-5,690774	0,849856	-0,025180
H46	-4,433417	0,480714	1,542902	H46	-4,402720	0,399512	1,633005
C47	-4,860414	0,248103	-2,300759	C47	-4,831216	0,323598	-2,219111
C48	-5,880446	0,795578	-1,515028	C48	-5,853854	0,829238	-1,409704
H49	-6,508393	1,271619	0,491200	H49	-6,486131	1,226850	0,611728
H50	-4,991284	0,149411	-3,375210	H50	-4,962946	0,272057	-3,296826
H51	-6,800611	1,142186	-1,975961	H51	-6,775759	1,190249	-1,856377
H52	0,667863	0,606360	1,130818	H52	0,721904	0,403421	1,226187
H53	1,265819	3,002237	-2,324231	H53	1,221148	3,309406	-2,484816
H54	-1,943914	-2,904291	2,438959	H54	-2,177709	-2,950241	2,494881
H55	-2,914979	-0,652929	-2,325382	H55	-2,879010	-0,564988	-2,278457

H56	3,265684	-3,970069	-0,010106	H56	3,257058	-4,072403	0,052128
C57	5,280433	-0,101817	-1,828584	C57	5,348083	-0,187807	-1,780960
C58	4,469260	-1,134276	-2,307325	C58	4,627738	-1,293994	-2,234462
C59	3,563824	-1,761152	-1,454977	C59	3,710074	-1,919866	-1,393617
C60	3,441363	-1,368515	-0,113453	C60	3,494140	-1,454267	-0,090589
C61	4,241259	-0,320120	0,351824	C61	4,199635	-0,326858	0,345049
C62	5,163601	0,296891	-0,497722	C62	5,128501	0,293470	-0,490632
H63	5,995917	0,383188	-2,485903	H63	6,071579	0,297345	-2,429795
H64	4,544111	-1,453363	-3,342841	H64	4,781460	-1,670725	-3,241397
H65	2,930343	-2,551600	-1,850974	H65	3,142581	-2,765997	-1,773905
H66	4,142959	0,026255	1,373942	H66	4,015822	0,078323	1,332532
H67	5,790719	1,096832	-0,114917	H67	5,679539	1,158640	-0,133056
C68	-1,426527	3,845266	2,321838	C68	-1,519890	3,977947	2,221694
C69	-0,654603	3,239486	1,334035	C69	-0,710806	3,483084	1,201607
C70	-1,218045	2,870185	0,097983	C70	-1,265821	3,122624	-0,037695
C71	-2,593665	3,068325	-0,080613	C71	-2,652063	3,206724	-0,204105
C72	-3,371042	3,660026	0,919706	C72	-3,461885	3,693104	0,824737
C73	-2,792040	4,069961	2,119339	C73	-2,899285	4,097625	2,033349
H74	-0,959115	4,126389	3,261507	H74	-1,070387	4,258004	3,170237
H75	0,388940	3,031901	1,530275	H75	0,350658	3,360152	1,375770
H76	-3,082050	2,722559	-0,983313	H76	-3,120138	2,848431	-1,113994
H77	-4,436968	3,786769	0,754053	H77	-4,536702	3,737216	0,678077
H78	-3,394630	4,539036	2,891450	H78	-3,529602	4,482315	2,830001
C79	-0,326110	2,322887	-1,000298	C79	-0,340696	2,662307	-1,155593
C80	0,758272	3,412119	-1,441227	C80	0,720735	3,729703	-1,604146
C81	-1,552076	3,017529	-3,204018	C81	-1,590754	3,139172	-3,347643
C82	-0,953385	0,815901	-2,884879	C82	-0,937183	0,961525	-2,908423
C83	-2,280964	2,105454	-4,192807	C83	-2,305273	2,156577	-4,278207
H84	-0,723859	3,552046	-3,680956	H84	-0,784951	3,677468	-3,858641
H85	-2,208418	3,739197	-2,717253	H85	-2,265631	3,863031	-2,887773
H86	-3,322510	1,936663	-3,904859	H86	-3,343396	1,989618	-3,976548
H87	-2,219895	2,430702	-5,231181	H87	-2,253954	2,423932	-5,333489
N88	-1,052446	2,007897	-2,246997	N88	-1,048514	2,199818	-2,351467
O89	-1,575309	0,838530	-4,073795	O89	-1,579943	0,913007	-4,089447
O90	-0,395683	-0,213048	-2,475731	O90	-0,369871	-0,037953	-2,459251
H91	2,431416	-4,128781	1,562492	H91	2,354877	-4,180806	1,589692
N92	-1,903346	-2,362716	-0,142698	N92	-1,892824	-2,401175	-0,112082
C93	-0,835088	-2,972932	-0,968819	C93	-0,825192	-2,957234	-0,980759
H94	-0,725099	-2,461302	-1,932216	H94	-0,724393	-2,393324	-1,916743
H95	0,184935	4,284679	-1,772032	H95	0,165610	4,617095	-1,927353
C96	3,668142	4,769177	1,453715	C96	3,635672	4,854472	1,373627
C97	2,522566	5,524009	1,197068	C97	2,617615	5,741429	1,021110
C98	1,594238	5,083460	0,255529	C98	1,682494	5,379280	0,051612
C99	1,791114	3,884269	-0,443335	C99	1,755268	4,131695	-0,580224
C100	2,962528	3,153858	-0,204520	C100	2,794314	3,255678	-0,237877
C101	3,887137	3,587311	0,745375	C101	3,721757	3,612871	0,739910
H102	4,391328	5,107164	2,190549	H102	4,361467	5,131902	2,132960
H103	2,350569	6,454303	1,730972	H103	2,548041	6,712422	1,503306

H104	0,694699	5,665834	0,073543	H104	0,879439	6,065666	-0,205829
H105	3,168962	2,246564	-0,763404	H105	2,873728	2,287398	-0,722489
H106	4,786797	3,005785	0,923994	H106	4,515941	2,920998	1,004532
H107	1,817888	0,469221	-0,698205	H107	1,811776	0,456909	-0,605517
H108	0,978495	1,530354	-0,784155	H108	0,357523	1,838784	-0,797711

C1_E_R_tN_Cheltop

C1_ER_Cheltop_SM				C1_ER_Cheltop_TS1			
C1	-4,302899	-3,269123	0,477358	C1	-4,286837	-3,352529	0,426943
C2	-2,234129	-4,424221	-0,110304	C2	-2,199749	-4,483993	-0,140674
C3	-3,701964	-4,254214	-0,579317	C3	-3,662853	-4,325548	-0,628286
C4	-3,088001	-2,998150	1,395876	C4	-3,090734	-3,087898	1,372293
H5	-5,110120	-3,735254	1,051931	H5	-5,106897	-3,824474	0,977817
H6	-3,766758	-3,866753	-1,601135	H6	-3,718388	-3,933490	-1,648908
H7	-3,316796	-2,531709	2,354787	H7	-3,339635	-2,637759	2,334084
H8	-4,696438	-2,354347	0,033750	H8	-4,669314	-2,431553	-0,014609
H9	-1,714728	-5,288447	-0,523966	H9	-1,663649	-5,335884	-0,558576
H10	-4,212548	-5,221149	-0,563506	H10	-4,164215	-5,297420	-0,623942
C11	-2,389101	-4,368486	1,426287	C11	-2,378708	-4,451782	1,393717
H12	-1,439335	-4,356936	1,969424	H12	-1,437516	-4,437401	1,951435
H13	-3,024821	-5,168384	1,815710	H13	-3,011708	-5,263925	1,761569
C14	-0,002198	-3,133241	-0,266159	C14	0,018307	-3,154722	-0,270531
N15	0,766005	-2,099433	-0,226667	N15	0,762321	-2,105515	-0,204842
C16	2,205075	-2,573141	-0,131995	C16	2,207523	-2,547578	-0,132412
C17	1,991230	-4,110631	0,068169	C17	2,031791	-4,093063	0,037113
P18	-1,919840	-0,525555	0,560710	P18	-1,923668	-0,595165	0,600194
Ir19	0,164061	-0,058183	-0,624820	Ir19	0,085291	-0,060798	-0,554580
C20	-1,837053	0,029039	2,300580	C20	-1,860499	-0,053368	2,343610
C21	-1,361624	1,003374	4,893823	C21	-1,384675	0,922331	4,931644
C22	-1,278160	-0,801845	3,281893	C22	-1,308609	-0,887565	3,325153
C23	-2,164294	1,352567	2,636372	C23	-2,176064	1,274694	2,671821
C24	-1,928352	1,833539	3,924106	C24	-1,939032	1,756883	3,958363
C25	-1,044974	-0,317082	4,569458	C25	-1,077303	-0,401633	4,612871
H26	-2,608373	2,005351	1,892753	H26	-2,614352	1,927322	1,923877
H27	-2,194874	2,857350	4,172941	H27	-2,193318	2,784536	4,203230
H28	-0,612812	-0,973485	5,319492	H28	-0,653009	-1,058844	5,366510
H29	-1,174678	1,380491	5,894729	H29	-1,198058	1,300967	5,932005
O30	0,574234	-4,332342	-0,178612	O30	0,622321	-4,344541	-0,225983
C31	2,905725	-2,040141	1,112898	C31	2,911195	-2,017880	1,110460
C32	2,168997	-1,569838	2,201171	C32	2,182989	-1,546120	2,203911
C33	4,297294	-2,163935	1,242284	C33	4,304674	-2,136296	1,223507
C34	2,808335	-1,170048	3,373585	C34	2,834980	-1,140024	3,367954
H35	1,094785	-1,478376	2,124841	H35	1,107083	-1,457548	2,138739
C36	4,936465	-1,774415	2,417961	C36	4,955145	-1,740646	2,390215
H37	4,887805	-2,555183	0,419791	H37	4,885545	-2,524558	0,391915
C38	4,194339	-1,265143	3,485196	C38	4,222534	-1,229909	3,463697
H39	2,215171	-0,771340	4,191732	H39	2,249768	-0,740422	4,191342
H40	6,016173	-1,864436	2,496214	H40	6,035925	-1,825643	2,457256

H41	4,695087	-0,948253	4,395547	H41	4,732035	-0,907610	4,367141
C42	-3,532178	0,052655	-0,081620	C42	-3,510682	0,013018	-0,067890
C43	-4,625225	0,333816	0,753062	C43	-4,625385	0,251716	0,750925
C44	-3,723611	0,000009	-1,470139	C44	-3,652987	0,070750	-1,462002
C45	-5,885661	0,564937	0,203069	C45	-5,860621	0,551748	0,179077
H46	-4,500499	0,359873	1,830830	H46	-4,533710	0,198026	1,831134
C47	-4,986797	0,224342	-2,014354	C47	-4,890852	0,366154	-2,028320
C48	-6,070883	0,506633	-1,180224	C48	-5,995215	0,607268	-1,209543
H49	-6,725485	0,781365	0,857342	H49	-6,719412	0,736629	0,817824
H50	-5,121517	0,192381	-3,091235	H50	-4,988032	0,425016	-3,107821
H51	-7,054671	0,682545	-1,605206	H51	-6,959235	0,840868	-1,652079
H52	-0,435034	-0,548872	-1,980053	H52	-0,539363	-0,570115	-1,890886
H53	1,588385	-0,151212	-1,322466	H53	1,512869	0,041373	-1,323708
H54	-1,016799	-1,825873	3,034088	H54	-1,050987	-1,912725	3,076942
H55	-2,879198	-0,201801	-2,121573	H55	-2,790241	-0,095418	-2,098070
H56	2,563314	-4,730981	-0,621481	H56	2,625534	-4,686835	-0,657657
C57	3,984224	-1,592429	-3,974116	C57	3,945391	-1,409283	-3,949645
C58	3,103811	-2,668184	-3,849196	C58	3,121858	-2,531247	-3,850158
C59	2,558973	-2,985306	-2,605396	C59	2,585814	-2,899702	-2,616403
C60	2,889343	-2,243420	-1,463048	C60	2,871367	-2,163345	-1,458710
C61	3,753906	-1,148967	-1,607112	C61	3,679300	-1,023084	-1,576255
C62	4,300792	-0,830754	-2,850506	C62	4,216194	-0,653660	-2,810222
H63	4,408542	-1,341302	-4,941800	H63	4,360137	-1,117816	-4,909909
H64	2,838430	-3,260459	-4,720246	H64	2,893728	-3,120702	-4,733631
H65	1,857482	-3,813115	-2,537239	H65	1,928924	-3,764611	-2,567585
H66	3,999304	-0,537673	-0,746867	H66	3,897414	-0,424724	-0,698580
H67	4,966835	0,022438	-2,940336	H67	4,839862	0,232782	-2,880176
C68	-3,025228	2,780749	-3,433654	C68	-2,969114	2,940590	-3,459192
C69	-1,850460	2,287163	-2,872265	C69	-1,797577	2,408809	-2,923319
C70	-1,549668	2,521044	-1,518909	C70	-1,506963	2,540585	-1,555675
C71	-2,495310	3,218273	-0,748858	C71	-2,459706	3,189512	-0,749076
C72	-3,677904	3,696992	-1,308849	C72	-3,635313	3,710292	-1,283317
C73	-3,943075	3,493775	-2,661013	C73	-3,892418	3,599989	-2,648672
H74	-3,227502	2,596650	-4,485181	H74	-3,159113	2,832804	-4,523598
H75	-1,177508	1,710174	-3,488962	H75	-1,119592	1,884817	-3,579757
H76	-2,312078	3,378234	0,307451	H76	-2,280515	3,279639	0,316375
H77	-4,391239	4,225732	-0,683172	H77	-4,351075	4,198500	-0,628110
H78	-4,859258	3,872621	-3,104347	H78	-4,803663	4,011542	-3,072649
C79	-0,272201	2,065134	-0,890670	C79	-0,248410	2,030883	-0,917981
C80	0,942489	1,720350	-1,598378	C80	0,987982	1,642943	-1,615878
C92	0,303878	4,150068	0,510510	C92	0,356285	4,175216	0,399014
C93	0,734094	2,050287	1,326804	C93	0,723904	2,099485	1,317144
C94	0,684668	4,208735	1,995722	C94	0,711910	4,287502	1,888587
H95	-0,554609	4,774028	0,260227	H95	-0,497346	4,790361	0,111642
H96	1,141261	4,415674	-0,150291	H96	1,204520	4,415047	-0,258004
H97	1,468415	4,928285	2,232671	H97	1,508312	4,998152	2,109684
H98	-0,186864	4,375521	2,636899	H98	-0,167089	4,505497	2,503655
N99	-0,003521	2,716083	0,395521	N99	0,044321	2,741296	0,331961

O100	1,196364	2,874938	2,274277	O100	1,184783	2,955432	2,239114
O101	0,989271	0,841848	1,310468	O101	0,935149	0,882223	1,366944
H102	2,191756	-4,414172	1,096744	H102	2,231816	-4,409332	1,062147
N103	-2,052431	-2,210710	0,669314	N103	-2,052378	-2,280894	0,674570
C104	-1,503502	-3,087580	-0,393018	C104	-1,483954	-3,134066	-0,397517
H105	-1,730096	-2,716589	-1,402352	H105	-1,713814	-2,752235	-1,402398
C107	1,629127	2,213194	-5,816506	C107	1,557874	1,984005	-5,866112
C108	1,455273	3,311866	-4,971562	C108	1,620856	3,100521	-5,028592
C109	1,225948	3,113663	-3,612845	C109	1,443288	2,950676	-3,655794
C110	1,159031	1,817117	-3,077706	C110	1,191868	1,686429	-3,100905
C111	1,349096	0,723662	-3,925993	C111	1,154129	0,571361	-3,940716
C112	1,581390	0,923648	-5,288464	C112	1,332593	0,722120	-5,317075
H113	1,801211	2,364751	-6,878269	H113	1,688588	2,099489	-6,938266
H114	1,490807	4,321282	-5,371578	H114	1,801026	4,087224	-5,445576
H115	1,071771	3,970131	-2,960588	H115	1,472931	3,824330	-3,008669
H116	1,316617	-0,280810	-3,521626	H116	0,997732	-0,413452	-3,516012
H117	1,726390	0,063636	-5,936058	H117	1,299815	-0,153490	-5,959196
H107	1,843120	2,004118	-1,049859	H107	1,879356	1,990612	-1,088375

C1_ER_Cheltop_INT1				C1_ER_Cheltop_TS2			
C1	-4,226375	-3,382837	0,456156	C1	-4,086516	-3,371088	1,275450
C2	-2,132592	-4,536453	-0,041782	C2	-2,062815	-4,535970	0,579430
C3	-3,594362	-4,407823	-0,544119	C3	-3,588434	-4,496900	0,312334
C4	-3,041075	-3,079813	1,402035	C4	-2,772404	-2,906951	1,946211
H5	-5,057226	-3,822782	1,017287	H5	-4,765337	-3,764769	2,039330
H6	-3,646503	-4,070964	-1,584328	H6	-3,820306	-4,284581	-0,736405
H7	-3,302762	-2,590662	2,341002	H7	-2,900024	-2,299575	2,841851
H8	-4,595199	-2,478438	-0,030994	H8	-4,606441	-2,565752	0,760769
H9	-1,588617	-5,402771	-0,418041	H9	-1,557742	-5,437680	0,235240
H10	-4,093514	-5,379433	-0,489665	H10	-4,035975	-5,464304	0,556944
C11	-2,323620	-4,438350	1,488466	C11	-2,001667	-4,230710	2,093024
H12	-1,387096	-4,395063	2,052795	H12	-0,987339	-4,099671	2,482127
H13	-2,956543	-5,236185	1,886949	H13	-2,534327	-4,972525	2,694591
C14	0,083680	-3,195037	-0,253152	C14	0,050340	-3,236770	-0,126110
N15	0,799178	-2,128629	-0,201023	N15	0,785475	-2,182928	-0,244849
C16	2,245320	-2,516010	-0,094856	C16	2,221748	-2,650976	-0,113575
C17	2,142921	-4,084469	-0,115820	C17	2,051479	-4,173112	-0,394002
P18	-1,853243	-0,620424	0,592018	P18	-1,840601	-0,570371	0,590710
Ir19	0,007333	-0,096391	-0,527968	Ir19	-0,112727	-0,196721	-0,777157
C20	-1,735947	-0,062844	2,326915	C20	-1,610779	0,254558	2,196185
C21	-1,236858	0,919029	4,906301	C21	-1,097209	1,601797	4,597896
C22	-1,272686	-0,920661	3,332725	C22	-1,003467	-0,416720	3,266910
C23	-1,949935	1,292630	2,624820	C23	-1,958418	1,606926	2,339199
C24	-1,701084	1,778379	3,907158	C24	-1,702117	2,274956	3,534605
C25	-1,030395	-0,430814	4,617350	C25	-0,755810	0,255565	4,462941
H26	-2,315999	1,963335	1,854179	H26	-2,442721	2,127775	1,522918
H27	-1,874559	2,827747	4,129868	H27	-1,986313	3,318300	3,641259
H28	-0,677847	-1,105909	5,391962	H28	-0,297668	-0,276200	5,291839

H29	-1,042987	1,299594	5,904806	H29	-0,900285	2,122405	5,530456
O30	0,718341	-4,369738	-0,229652	O30	0,656356	-4,424132	-0,085279
C31	2,847055	-2,100297	1,247133	C31	2,706219	-2,461753	1,330983
C32	2,046770	-1,658930	2,299660	C32	2,002541	-1,677541	2,243954
C33	4,221957	-2,277174	1,464998	C33	3,894877	-3,081733	1,744446
C34	2,608846	-1,361891	3,541426	C34	2,471465	-1,515676	3,549404
H35	0,988308	-1,510971	2,143526	H35	1,116219	-1,151546	1,923350
C36	4,782643	-1,989958	2,707449	C36	4,363162	-2,921808	3,045829
H37	4,857736	-2,626854	0,655719	H37	4,476273	-3,669095	1,037738
C38	3,976670	-1,524529	3,749793	C38	3,651818	-2,134341	3,954752
H39	1,969655	-0,990476	4,337625	H39	1,916856	-0,884914	4,238223
H40	5,849751	-2,123661	2,860341	H40	5,287411	-3,405448	3,348342
H41	4,416074	-1,290547	4,715334	H41	4,018843	-2,006387	4,969012
C42	-3,440861	0,021971	-0,039673	C42	-3,556290	-0,253481	0,054074
C43	-4,508889	0,306159	0,826719	C43	-4,542293	0,178211	0,955420
C44	-3,645773	0,062573	-1,426201	C44	-3,944806	-0,770596	-1,191245
C45	-5,761066	0,631086	0,307553	C45	-5,890441	0,101069	0,609063
H46	-4,370279	0,268264	1,902391	H46	-4,268916	0,547954	1,937097
C47	-4,899485	0,385540	-1,937899	C47	-5,293693	-0,860868	-1,524527
C48	-5,958312	0,669473	-1,073649	C48	-6,269090	-0,421803	-0,627677
H49	-6,582942	0,851412	0,982844	H49	-6,645064	0,439306	1,313209
H50	-5,044471	0,432229	-3,012603	H50	-5,583049	-1,261543	-2,491659
H51	-6,934698	0,924609	-1,475574	H51	-7,320696	-0,489244	-0,891041
H52	-0,694161	-0,623209	-1,828489	H52	-0,943801	-0,903491	-1,912028
H53	1,543876	0,579390	-1,588868	H53	-0,195438	1,338848	-3,550153
H54	-1,092031	-1,967195	3,109914	H54	-0,719257	-1,457827	3,165609
H55	-2,819050	-0,134374	-2,099433	H55	-3,189954	-1,106243	-1,894402
H56	2,653276	-4,555840	-0,956692	H56	2,211773	-4,446948	-1,439700
C57	4,124138	-0,762351	-3,604468	C57	4,741670	-0,694513	-3,061633
C58	3,372615	-1,933947	-3,713843	C58	3,716524	-1,554768	-3,460454
C59	2,787942	-2,500832	-2,581756	C59	2,938817	-2,210018	-2,504191
C60	2,959692	-1,922758	-1,315785	C60	3,159794	-2,008958	-1,134274
C61	3,701158	-0,737633	-1,218836	C61	4,192534	-1,146333	-0,745495
C62	4,278107	-0,163361	-2,354411	C62	4,981618	-0,502922	-1,700266
H63	4,571460	-0,314523	-4,486531	H63	5,347446	-0,183895	-3,804429
H64	3,231155	-2,402424	-4,683525	H64	3,517878	-1,715194	-4,516311
H65	2,181614	-3,395386	-2,697828	H65	2,123046	-2,844081	-2,840505
H66	3,827704	-0,261101	-0,252467	H66	4,383437	-0,968411	0,305593
H67	4,850853	0,754814	-2,257765	H67	5,778139	0,160045	-1,374722
C68	-2,934329	2,874876	-3,497142	C68	-4,041492	1,826175	-3,080540
C69	-1,750384	2,335358	-2,993803	C69	-2,717920	1,518616	-2,789386
C70	-1,389073	2,509781	-1,647612	C70	-2,058827	2,085463	-1,684199
C71	-2,293993	3,211893	-0,828239	C71	-2,806502	2,937224	-0,862484
C72	-3,484488	3,735605	-1,326834	C72	-4,145195	3,229903	-1,135430
C73	-3,810851	3,581138	-2,673509	C73	-4,767875	2,688506	-2,255317
H74	-3,172807	2,729878	-4,547507	H74	-4,514529	1,380263	-3,951085
H75	-1,119530	1,766732	-3,660144	H75	-2,184662	0,827356	-3,433121
H76	-2,070621	3,335492	0,225776	H76	-2,342625	3,396362	0,001890

H77	-4,158382	4,261209	-0,656109	H77	-4,693736	3,893339	-0,472280
H78	-4,732989	3,994595	-3,070656	H78	-5,804364	2,922800	-2,478209
C79	-0,147961	1,926460	-1,025081	C79	-0,585175	1,798897	-1,467293
C80	1,166380	1,642484	-1,823318	C80	0,190079	2,051477	-2,814758
C92	0,575641	4,119874	0,206825	C81	0,141036	4,157024	-0,403737
C93	0,915896	2,057502	1,155916	C82	0,782446	2,172208	0,552999
C94	1,020176	4,253510	1,671657	C83	0,620462	4,368491	1,035163
H95	-0,274617	4,754495	-0,045557	H84	-0,791063	4,667928	-0,640373
H96	1,391832	4,326682	-0,500415	H85	0,892005	4,461648	-1,134928
H97	1,855753	4,935486	1,830121	H86	1,346085	5,173608	1,151424
H98	0,187835	4,522773	2,329816	H87	-0,206006	4,503046	1,742125
N99	0,219934	2,695003	0,184487	N88	-0,019093	2,683735	-0,401437
O100	1,458342	2,911360	2,032255	O89	1,281759	3,113091	1,364387
O101	1,072987	0,829018	1,231394	O90	1,074585	0,967979	0,707416
H102	2,490764	-4,525281	0,818505	H91	2,656972	-4,804926	0,251933
N103	-1,998956	-2,301271	0,677675	N92	-1,873660	-2,220516	0,964777
C104	-1,422776	-3,194926	-0,357408	C93	-1,449944	-3,241134	-0,017432
H105	-1,663445	-2,858762	-1,376121	H94	-1,848542	-3,050612	-1,024395
C107	1,317174	2,035884	-6,107680	C95	0,329240	6,067238	-4,488217
C108	1,459600	3,157008	-5,287134	C96	-0,902931	5,430563	-4,344037
C109	1,419615	3,016250	-3,901196	C97	-0,981904	4,140632	-3,815542
C110	1,231318	1,756128	-3,321834	C98	0,175851	3,459546	-3,409518
C111	1,104049	0,635701	-4,146704	C99	1,410084	4,108554	-3,578877
C112	1,143949	0,775108	-5,533922	C100	1,491742	5,396034	-4,108651
H113	1,344817	2,145131	-7,187957	H101	0,383605	7,071469	-4,898940
H114	1,598540	4,140442	-5,726960	H102	-1,814790	5,937712	-4,648186
H115	1,518184	3,892782	-3,264536	H103	-1,952981	3,671967	-3,728041
H116	0,974268	-0,343535	-3,696995	H104	2,323731	3,596935	-3,281781
H117	1,043428	-0,102177	-6,166878	H105	2,461211	5,872799	-4,224903
H107	1,977254	2,227550	-1,373961	H106	1,237121	1,793709	-2,688421
				H107	2,418223	0,613369	-1,428334
				H108	2,158133	0,188329	-1,984950

C1_ER_Cheltop_INT2

C1	-4,137901	-3,412395	1,327088
C2	-2,112310	-4,596984	0,674184
C3	-3,636475	-4,564864	0,397333
C4	-2,827482	-2,936069	1,998049
H5	-4,826919	-3,783078	2,093276
H6	-3,861024	-4,381874	-0,658446
H7	-2,964274	-2,310099	2,879279
H8	-4,646105	-2,615604	0,786356
H9	-1,602904	-5,505572	0,353605
H10	-4,087041	-5,524644	0,665742
C11	-2,059748	-4,256894	2,180524
H12	-1,047597	-4,117583	2,572355
H13	-2,596278	-4,985283	2,795005
C14	-0,002780	-3,313277	-0,085491

C1_ER_Cheltop_TS3

C1	-4,146374	-3,505526	1,373241
C2	-2,052622	-4,634007	0,841155
C3	-3,566742	-4,677968	0,514518
C4	-2,882256	-2,965302	2,084887
H5	-4,870290	-3,864226	2,112200
H6	-3,763242	-4,556906	-0,555628
H7	-3,071033	-2,316211	2,940190
H8	-4,636904	-2,737795	0,773902
H9	-1,494544	-5,528703	0,566517
H10	-3,985302	-5,642295	0,816320
C11	-2,068956	-4,246649	2,336546
H12	-1,078198	-4,051071	2,758071
H13	-2,594007	-4,980111	2,954799
C14	0,016324	-3,276251	0,079785

N15	0,703751	-2,255374	-0,270126	N15	0,692566	-2,210951	-0,194348
C16	2,157032	-2,655334	-0,153609	C16	2,167227	-2,554129	-0,087194
C17	2,033550	-4,192889	-0,352305	C17	2,088087	-4,107803	-0,111774
P18	-1,888504	-0,613459	0,629171	P18	-1,975140	-0,646416	0,682235
Ir19	-0,118743	-0,285076	-0,853649	Ir19	-0,153699	-0,399017	-0,962993
C20	-1,640841	0,215022	2,241394	C20	-1,746251	0,191378	2,298251
C21	-1,102763	1,580543	4,638277	C21	-1,198572	1,566444	4,688542
C22	-1,058253	-0,455197	3,326019	C22	-1,102916	-0,455931	3,362191
C23	-1,956523	1,576514	2,373807	C23	-2,126191	1,533891	2,455338
C24	-1,687339	2,252799	3,562827	C24	-1,853538	2,215658	3,640238
C25	-0,797118	0,224581	4,516414	C25	-0,832435	0,227938	4,547693
H26	-2,430018	2,101064	1,552918	H26	-2,667508	2,036673	1,663151
H27	-1,948164	3,303782	3,654420	H27	-2,169111	3,249803	3,752102
H28	-0,356542	-0,311793	5,351984	H28	-0,340098	-0,291456	5,365032
H29	-0,896840	2,106318	5,565608	H29	-0,987787	2,096359	5,612805
O30	0,647170	-4,476353	0,000232	O30	0,706799	-4,398478	0,258024
C31	2,673939	-2,376229	1,262950	C31	2,728299	-2,126074	1,274517
C32	1,931708	-1,669830	2,205889	C32	1,962830	-1,472422	2,234983
C33	3,946116	-2,850435	1,616263	C33	4,065149	-2,436976	1,567627
C34	2,443984	-1,442504	3,484676	C34	2,519707	-1,116123	3,464524
H35	0,970290	-1,265072	1,934442	H35	0,942887	-1,204115	2,012183
C36	4,455656	-2,630761	2,892779	C36	4,621192	-2,088614	2,794894
H37	4,550971	-3,379265	0,883488	H37	4,680247	-2,931702	0,820098
C38	3,702520	-1,924576	3,834734	C38	3,847959	-1,421411	3,749188
H39	1,855045	-0,873352	4,197719	H39	1,909240	-0,584816	4,188826
H40	5,442028	-3,005114	3,150796	H40	5,659724	-2,328750	3,003364
H41	4,100749	-1,748706	4,829759	H41	4,283868	-1,139795	4,703417
C42	-3,610511	-0,254534	0,132577	C42	-3,704382	-0,287209	0,197183
C43	-4,581679	0,171414	1,052952	C43	-4,703533	0,045067	1,124767
C44	-4,019422	-0,708952	-1,130099	C44	-4,066180	-0,574675	-1,126911
C45	-5,933108	0,145469	0,711257	C45	-6,039455	0,094302	0,729581
H46	-4,292580	0,502874	2,044230	H46	-4,445253	0,253354	2,158067
C47	-5,371790	-0,746039	-1,461623	C47	-5,403748	-0,536164	-1,515370
C48	-6,331650	-0,320793	-0,542262	C48	-6,392574	-0,201516	-0,588656
H49	-6,675241	0,477613	1,431557	H49	-6,806248	0,351959	1,454738
H50	-5,675557	-1,097728	-2,443199	H50	-5,672602	-0,754662	-2,544605
H51	-7,385797	-0,350852	-0,802215	H51	-7,435032	-0,169670	-0,892337
H52	-1,034947	-0,968793	-1,942098	H52	-1,000337	-1,349643	-1,898896
H53	0,024973	1,335818	-3,585450	H53	0,069502	1,509152	-3,728913
H54	-0,800788	-1,504878	3,241999	H54	-0,810964	-1,495565	3,263632
H55	-3,278482	-1,032441	-1,853531	H55	-3,297430	-0,816919	-1,853324
H56	2,179671	-4,524834	-1,382743	H56	2,258868	-4,552800	-1,093902
C57	4,175405	-0,333916	-3,208758	C57	4,146788	-0,674479	-3,457336
C58	3,498777	-1,505495	-3,556312	C58	3,420459	-1,851951	-3,640604
C59	2,902963	-2,292078	-2,569283	C59	2,817003	-2,478713	-2,551419
C60	2,976698	-1,926378	-1,218510	C60	2,935555	-1,947801	-1,260445
C61	3,664423	-0,752422	-0,876753	C61	3,658965	-0,760229	-1,088318
C62	4,258995	0,035361	-1,865034	C62	4,260861	-0,130294	-2,177327

H63	4,634765	0,280598	-3,977228
H64	3,427479	-1,805645	-4,597709
H65	2,346538	-3,172592	-2,875519
H66	3,707811	-0,436452	0,158803
H67	4,784431	0,942475	-1,580128
C68	-3,914812	1,790012	-3,247800
C69	-2,593128	1,500221	-2,930502
C70	-1,979443	2,035092	-1,783357
C71	-2,766840	2,839255	-0,951609
C72	-4,103235	3,114623	-1,252926
C73	-4,682421	2,603605	-2,409629
H74	-4,353308	1,369748	-4,148838
H75	-2,025231	0,847777	-3,585194
H76	-2,337484	3,269554	-0,055119
H77	-4,685082	3,739578	-0,580963
H78	-5,717546	2,823945	-2,652161
C79	-0,515606	1,746312	-1,523617
C80	0,349331	2,033137	-2,806166
C81	0,175431	4,059403	-0,355579
C82	0,760649	2,041831	0,565052
C83	0,598437	4,222244	1,107423
H84	-0,744295	4,583915	-0,611564
H85	0,956446	4,380423	-1,047307
H86	1,324053	5,017562	1,278003
H87	-0,254822	4,339862	1,784476
N88	0,004380	2,588999	-0,403900
O89	1,236923	2,951358	1,423841
O90	1,024765	0,829422	0,699560
H91	2,670577	-4,767546	0,316595
N92	-1,922931	-2,268669	1,011402
C93	-1,502605	-3,315699	0,047759
H94	-1,909820	-3,145305	-0,959594
C95	0,631385	6,053215	-4,429762
C96	-0,615476	5,432918	-4,351029
C97	-0,736068	4,140973	-3,836629
C98	0,391772	3,443747	-3,378748
C99	1,641494	4,075454	-3,481263
C100	1,765480	5,364782	-3,997758
H101	0,719551	7,058931	-4,830736
H102	-1,504255	5,954319	-4,696466
H103	-1,714804	3,681233	-3,798038
H104	2,531739	3,546938	-3,144689
H105	2,745370	5,829640	-4,063542
H106	1,382225	1,762122	-2,572066
H107	1,403385	-0,105371	-1,725533
H108	0,873670	-0,454531	-2,275755

C1_ER_Cheltop_PROD

H63	4,618317	-0,185339	-4,304996
H64	3,317139	-2,280990	-4,632960
H65	2,221633	-3,368432	-2,732456
H66	3,729623	-0,311905	-0,104264
H67	4,820370	0,788493	-2,022221
C68	-3,914323	2,033685	-3,378723
C69	-2,592686	1,701803	-3,100763
C70	-1,988779	2,073807	-1,887467
C71	-2,776559	2,751736	-0,952723
C72	-4,107966	3,073880	-1,220139
C73	-4,681233	2,727828	-2,439520
H74	-4,349320	1,744723	-4,331183
H75	-2,026136	1,156075	-3,849847
H76	-2,350005	3,033418	0,000389
H77	-4,693273	3,596660	-0,469191
H78	-5,714727	2,982104	-2,653736
C79	-0,523615	1,751958	-1,643533
C80	0,401596	2,100262	-2,867640
C81	0,105676	3,962135	-0,297585
C82	0,784381	1,903066	0,459558
C83	0,557915	4,029752	1,163922
H84	-0,836703	4,472180	-0,491399
H85	0,860708	4,358991	-0,976132
H86	1,256274	4,839564	1,374935
H87	-0,279860	4,057787	1,868590
N88	-0,020058	2,490850	-0,448732
O89	1,253531	2,765727	1,364761
O90	1,079925	0,696271	0,495664
H91	2,735962	-4,575720	0,626716
N92	-1,981831	-2,291878	1,106799
C93	-1,485171	-3,344450	0,190848
H94	-1,874456	-3,231130	-0,831606
C95	0,924589	6,241306	-4,045216
C96	-0,354262	5,683724	-4,044312
C97	-0,546177	4,352229	-3,674313
C98	0,539847	3,553939	-3,285388
C99	1,822929	4,122143	-3,308902
C100	2,017024	5,451983	-3,683343
H101	1,069253	7,278626	-4,333133
H102	-1,209936	6,286157	-4,337131
H103	-1,547552	3,941233	-3,689893
H104	2,679224	3,514079	-3,024270
H105	3,019496	5,870697	-3,692331
H106	1,390385	1,704516	-2,618569
H107	1,025620	-0,408231	-2,044249
H108	-0,572072	0,292056	-2,344071

C1_E_S_tN_Cheltop

C1_ES_Cheltop_SM

C1	-4,108083	-4,025846	0,538970
C2	-1,876337	-4,968475	0,274129
C3	-3,307108	-5,064570	-0,315496
C4	-3,034940	-3,518627	1,532057
H5	-4,936027	-4,496837	1,078518
H6	-3,330923	-4,842357	-1,387199
H7	-3,426050	-2,996903	2,405332
H8	-4,520495	-3,207605	-0,054980
H9	-1,208476	-5,786929	0,004860
H10	-3,697563	-6,077595	-0,182857
C11	-2,177600	-4,772932	1,777078
H12	-1,290968	-4,572712	2,385619
H13	-2,739814	-5,608083	2,204514
C14	0,161224	-3,375687	-0,015341
N15	0,765598	-2,245585	-0,196307
C16	2,223677	-2,413628	0,196293
C17	2,316546	-3,962328	0,124197
P18	-2,038616	-1,000412	0,569323
Ir19	-0,116039	-0,525259	-0,805459
C20	-2,099900	-0,256912	2,242292
C21	-2,022986	1,042067	4,731438
C22	-1,844504	-0,983150	3,412929
C23	-2,296229	1,132088	2,328868
C24	-2,258188	1,774030	3,564149
C25	-1,816078	-0,335412	4,650580
H26	-2,485778	1,715506	1,432818
H27	-2,414126	2,848066	3,612406
H28	-1,625435	-0,912015	5,551468
H29	-1,997584	1,542293	5,694936
O30	0,936993	-4,411064	0,298800
C31	2,449521	-1,978151	1,650609
C32	1,456690	-1,414826	2,448738
C33	3,722623	-2,190418	2,203616
C34	1,728665	-1,054800	3,770777
H35	0,472503	-1,229012	2,045799
C36	3,993782	-1,838665	3,521934
H37	4,513786	-2,612347	1,589097
C38	2,994017	-1,264613	4,311595
H39	0,941271	-0,603588	4,366479
H40	4,986150	-2,006081	3,930510
H41	3,205162	-0,983289	5,339237
C42	-3,667902	-0,565728	-0,170451
C43	-4,778100	-0,172600	0,590746
C44	-3,812565	-0,700021	-1,562632
C45	-5,999969	0,097119	-0,027783
H46	-4,688912	-0,063927	1,667032
C47	-5,036585	-0,436730	-2,178014
C48	-6,133241	-0,029785	-1,412401

C1	-4,058318	-3,565409	0,059551
C2	-1,868330	-4,632708	-0,011502
C3	-3,240745	-4,638964	-0,730987
C4	-3,037534	-3,090362	1,121744
H5	-4,929764	-4,005838	0,555172
H6	-3,150955	-4,400153	-1,795681
H7	-3,462096	-2,527225	1,952839
H8	-4,413431	-2,749720	-0,569850
H9	-1,238193	-5,499413	-0,210410
H10	-3,697880	-5,629658	-0,655333
C11	-2,285822	-4,390691	1,456776
H12	-1,445218	-4,238168	2,140065
H13	-2,938041	-5,178931	1,843028
C14	0,289342	-3,206229	-0,039854
N15	0,991342	-2,124014	-0,061005
C16	2,355600	-2,450001	0,516288
C17	2,328384	-4,005280	0,420952
P18	-1,869994	-0,672048	0,114232
Ir19	0,360007	-0,203232	-0,780506
C20	-2,198309	0,188538	1,693561
C21	-2,436837	1,663845	4,073781
C22	-1,958259	-0,419884	2,933816
C23	-2,564655	1,544263	1,660659
C24	-2,680997	2,275058	2,841930
C25	-2,082498	0,314647	4,114505
H26	-2,781135	2,024243	0,711493
H27	-2,969282	3,321668	2,800132
H28	-1,904638	-0,172456	5,069230
H29	-2,528359	2,233407	4,993652
O30	0,912404	-4,326300	0,323634
C31	2,419714	-2,075277	2,005020
C32	1,323560	-1,571081	2,700566
C33	3,607148	-2,329355	2,710048
C34	1,413503	-1,294567	4,066304
H35	0,405599	-1,362006	2,173449
C36	3,697237	-2,059344	4,073128
H37	4,472926	-2,724655	2,185070
C38	2,598091	-1,533947	4,758681
H39	0,553126	-0,874223	4,578375
H40	4,627776	-2,254300	4,598509
H41	2,669848	-1,314269	5,819743
C42	-3,335599	-0,372192	-0,942283
C43	-4,581505	0,022343	-0,433700
C44	-3,232178	-0,748051	-2,291199
C45	-5,702863	0,043420	-1,263300
H46	-4,683579	0,302499	0,609740
C47	-4,356932	-0,733646	-3,113581
C48	-5,595286	-0,338318	-2,602300

H49	-6,849756	0,405528	0,574736	H49	-6,663079	0,352551	-0,860252
H50	-5,133328	-0,543575	-3,254901	H50	-4,263008	-1,014769	-4,158277
H51	-7,085484	0,179528	-1,890842	H51	-6,470526	-0,323187	-3,245139
H52	-0,743069	-1,359252	-1,966227	H52	-0,029588	-0,825621	-2,156933
H53	-1,673549	1,598960	-3,371206	H53	1,848624	-0,275747	-1,317886
H54	-1,639625	-2,047044	3,360552	H54	-1,665664	-1,463170	2,979884
H55	-2,958898	-0,999712	-2,164479	H55	-2,268398	-1,033893	-2,697402
H56	2,657731	-4,341792	-0,841329	H56	2,816179	-4,409252	-0,468605
C57	4,612981	-0,181652	-2,650617	C57	5,382240	-0,565831	-1,966770
C58	4,060871	-1,410630	-3,014334	C58	4,689876	-1,687098	-2,426679
C59	3,319078	-2,145595	-2,091060	C59	3,731871	-2,297686	-1,618760
C60	3,129309	-1,673263	-0,786880	C60	3,460367	-1,814206	-0,330529
C61	3,679038	-0,434115	-0,434076	C61	4,148599	-0,677413	0,113064
C62	4,413801	0,305722	-1,358810	C62	5,101091	-0,060508	-0,697951
H63	5,193584	0,389499	-3,369849	H63	6,127337	-0,087427	-2,595723
H64	4,201311	-1,796091	-4,019970	H64	4,888096	-2,083469	-3,418514
H65	2,862793	-3,075593	-2,416078	H65	3,176876	-3,140338	-2,019790
H66	3,511150	-0,030488	0,556826	H66	3,932633	-0,267179	1,092956
H67	4,830743	1,264470	-1,062513	H67	5,628753	0,816533	-0,332897
C68	-4,159253	3,354906	-0,659299	C68	1,860865	3,456766	-4,799203
C69	-3,104192	2,679817	-1,266512	C69	1,291795	3,152012	-3,564777
C70	-1,774684	2,988935	-0,943379	C70	1,585962	1,937533	-2,923334
C71	-1,527086	3,983033	0,008623	C71	2,460105	1,037998	-3,537376
C72	-2,584371	4,671582	0,606901	C72	3,027297	1,342565	-4,775486
C73	-3,902422	4,359567	0,276805	C73	2,728899	2,548995	-5,409944
H74	-5,179671	3,084214	-0,911429	H74	1,618229	4,395663	-5,288653
H75	-3,325310	1,892757	-1,978033	H75	0,595267	3,845785	-3,102977
H76	-0,511231	4,221835	0,302356	H76	2,685790	0,095998	-3,052421
H77	-2,373476	5,448067	1,336586	H77	3,699967	0,630757	-5,246011
H78	-4,724043	4,889157	0,749881	H78	3,165593	2,780374	-6,377513
C79	-0,648162	2,187287	-1,592308	C79	0,989849	1,719253	-1,559443
C80	-0,710005	2,061233	-3,135593	C80	-0,423190	1,797097	-1,310796
C81	1,421467	3,745750	-1,564667	C92	2,509508	3,580016	-0,575297
C82	1,267776	2,010721	-0,014321	C93	1,780771	1,825754	0,751351
C83	2,352841	3,982307	-0,362194	C94	3,224552	3,570785	0,789922
H84	0,760743	4,590049	-1,769651	H95	1,769041	4,385811	-0,663939
H85	1,967404	3,506937	-2,480342	H96	3,200759	3,645014	-1,417234
H86	3,385152	4,189743	-0,644966	H97	4,260433	3,228188	0,706647
H87	1,989907	4,771637	0,304174	H98	3,191474	4,526096	1,314229
N88	0,667051	2,574422	-1,096476	N99	1,854224	2,272999	-0,520568
O89	2,332286	2,730892	0,373459	O100	2,496866	2,592059	1,586792
O90	0,946351	0,972057	0,569397	O101	1,146441	0,829601	1,122806
H91	2,910626	-4,391873	0,927894	H102	2,722379	-4,489422	1,312457
N92	-2,034951	-2,677397	0,820548	N103	-1,920954	-2,326405	0,491559
C93	-1,326036	-3,576744	-0,129100	C104	-1,178031	-3,289805	-0,362172
H94	-1,579832	-3,356304	-1,175408	H105	-1,280921	-3,067156	-1,433986
C95	-0,159791	5,680951	-5,448716	C107	-3,381279	2,529983	-4,341602
C96	-1,266154	5,558527	-4,606731	C108	-3,501692	3,083403	-3,068010

C97	-1,457781	4,396612	-3,858972
C98	-0,538049	3,340068	-3,932057
C99	0,561193	3,469812	-4,791863
C100	0,751396	4,628775	-5,544846
H101	-0,014721	6,585607	-6,032154
H102	-1,988539	6,367045	-4,536203
H103	-2,330244	4,314555	-3,220968
H104	1,274077	2,651777	-4,873712
H105	1,608235	4,708023	-6,207924
H106	0,054285	1,332848	-3,417752
H107	1,102180	-0,314064	-1,831597
H108	-0,890966	1,139564	-1,213599

C1_ES_Cheltop_TS1

C1	-4,026752	-3,759265	0,025393
C2	-1,807645	-4,773433	0,022319
C3	-3,163969	-4,825659	-0,727846
C4	-3,051042	-3,262224	1,117920
H5	-4,913263	-4,206915	0,486361
H6	-3,056941	-4,606043	-1,794963
H7	-3,513456	-2,707796	1,934797
H8	-4,362837	-2,945323	-0,619038
H9	-1,147652	-5,622171	-0,155863
H10	-3,598929	-5,825482	-0,641729
C11	-2,270461	-4,538438	1,478183
H12	-1,452506	-4,356539	2,181538
H13	-2,907943	-5,344600	1,851997
C14	0,313774	-3,269555	-0,002880
N15	0,969328	-2,159626	-0,031176
C16	2,348363	-2,428026	0,514782
C17	2,389545	-3,986227	0,432649
P18	-1,903817	-0,816213	0,172941
Ir19	0,176472	-0,244997	-0,705693
C20	-2,184660	0,023858	1,767467
C21	-2,406447	1,465068	4,160683
C22	-2,013464	-0,621301	2,998441
C23	-2,461278	1,400700	1,745528
C24	-2,571493	2,115293	2,935269
C25	-2,131425	0,098130	4,189095
H26	-2,603968	1,909546	0,796176
H27	-2,790565	3,179026	2,907897
H28	-2,008282	-0,413212	5,139826
H29	-2,494978	2,022634	5,088552
O30	0,987883	-4,368593	0,345093
C31	2,427819	-2,033160	1,996418
C32	1,326685	-1,558584	2,705633
C33	3,640243	-2,226325	2,677317
C34	1,435801	-1,252576	4,063289

C109	-2,537109	2,815543	-2,097277
C110	-1,444398	1,980092	-2,371756
C111	-1,345484	1,416693	-3,654692
C112	-2,295819	1,699106	-4,629750
H113	-4,127328	2,740447	-5,102555
H114	-4,342083	3,728740	-2,828799
H115	-2,626239	3,277433	-1,116801
H116	-0,520271	0,754438	-3,885867
H117	-2,194238	1,260044	-5,618562
H107	-0,690930	2,271203	-0,366928

C1_ES_Cheltop_INT1

C1	-4,236530	-3,496566	0,109269
C2	-2,154753	-4,671755	0,595512
C3	-3,432607	-4,783393	-0,276097
C4	-3,319204	-2,843704	1,169685
H5	-5,208465	-3,742612	0,548955
H6	-3,201833	-4,834686	-1,344954
H7	-3,790027	-2,074813	1,782508
H8	-4,412890	-2,830017	-0,736778
H9	-1,574338	-5,589957	0,688158
H10	-3,983226	-5,692102	-0,016767
C11	-2,722539	-4,061643	1,896217
H12	-1,960304	-3,790996	2,633362
H13	-3,478162	-4,699038	2,363917
C14	0,117265	-3,413369	0,460217
N15	0,806376	-2,331031	0,427287
C16	2,171229	-2,615017	0,961806
C17	2,167200	-4,177844	0,913184
P18	-1,835972	-0,740733	-0,032032
Ir19	0,252906	-0,525898	-0,678026
C20	-2,108180	0,330749	1,417023
C21	-2,276153	2,062747	3,615447
C22	-2,001991	-0,170725	2,721889
C23	-2,284159	1,710908	1,221379
C24	-2,370496	2,568761	2,315647
C25	-2,092382	0,693176	3,814449
H26	-2,360309	2,112427	0,214866
H27	-2,513764	3,633442	2,154849
H28	-2,018181	0,293115	4,821685
H29	-2,345449	2,733214	4,466864
O30	0,749560	-4,528580	0,837682
C31	2,289397	-2,107729	2,402599
C32	1,359973	-1,218749	2,948065
C33	3,354237	-2,541153	3,206120
C34	1,494478	-0,766378	4,260917

H35	0,388139	-1,397231	2,197933	H35	0,538453	-0,859953	2,346253
C36	3,749161	-1,928024	4,032454	C36	3,491425	-2,087173	4,517503
H37	4,508942	-2,595981	2,138460	H37	4,096419	-3,227013	2,804494
C38	2,644209	-1,433273	4,730545	C38	2,559945	-1,194529	5,051890
H39	0,570776	-0,856944	4,587198	H39	0,760531	-0,069345	4,656177
H40	4,696835	-2,076370	4,542142	H40	4,326936	-2,431931	5,119840
H41	2,730023	-1,191021	5,785891	H41	2,665880	-0,839622	6,072703
C42	-3,379567	-0,427293	-0,832560	C42	-3,195709	-0,406639	-1,197071
C43	-4,597641	-0,039200	-0,253156	C43	-4,384565	0,215741	-0,787043
C44	-3,317844	-0,652276	-2,215963	C44	-3,111253	-0,947208	-2,489952
C45	-5,730119	0,128573	-1,049135	C45	-5,464748	0,307450	-1,665451
H46	-4,666979	0,131628	0,816375	H46	-4,477263	0,622673	0,214140
C47	-4,452266	-0,485645	-3,006366	C47	-4,191787	-0,853882	-3,360914
C48	-5,659591	-0,093918	-2,425160	C48	-5,370345	-0,225033	-2,950990
H49	-6,667896	0,432379	-0,592380	H49	-6,380632	0,793236	-1,341296
H50	-4,387142	-0,641848	-4,078493	H50	-4,107395	-1,254919	-4,365968
H51	-6,542481	0,042371	-3,043236	H51	-6,211614	-0,148483	-3,633877
H52	-0,230137	-0,919524	-2,052003	H52	-0,250375	-1,412071	-1,866816
H53	1,642090	0,147778	-1,401239	H53	1,925585	1,117744	-2,875022
H54	-1,775983	-1,679299	3,030751	H54	-1,834204	-1,230337	2,882616
H55	-2,376687	-0,931660	-2,675942	H55	-2,190635	-1,411962	-2,823041
H56	2,890705	-4,377735	-0,455010	H56	2,650197	-4,571650	0,013693
C57	5,289439	-0,436039	-1,992737	C57	4,911375	-0,820029	-1,904028
C58	4,671404	-1,609231	-2,428285	C58	3,819963	-1,579157	-2,330983
C59	3,724379	-2,242784	-1,624599	C59	2,973109	-2,170369	-1,392913
C60	3,399517	-1,738107	-0,357331	C60	3,187719	-1,996755	-0,012385
C61	4,010230	-0,547637	0,059296	C61	4,277392	-1,227273	0,402542
C62	4,944382	0,097546	-0,752038	C62	5,137608	-0,654016	-0,537894
H63	6,023804	0,060734	-2,619843	H63	5,582517	-0,370810	-2,630904
H64	4,917159	-2,027869	-3,400112	H64	3,632994	-1,724065	-3,391346
H65	3,230906	-3,130785	-2,007206	H65	2,137415	-2,770589	-1,739678
H66	3,762609	-0,130714	1,029014	H66	4,446498	-1,048812	1,457539
H67	5,413468	1,014846	-0,405614	H67	5,985676	-0,067933	-0,194278
C68	2,065579	3,392295	-4,763384	C68	0,674376	5,515007	-2,762253
C69	1,512088	3,101018	-3,519468	C69	0,939545	4,304360	-2,125669
C70	1,731941	1,848539	-2,925207	C70	1,290273	3,171416	-2,870900
C71	2,490867	0,891460	-3,596382	C71	1,379420	3,271304	-4,260625
C72	3,038800	1,182103	-4,846583	C72	1,106764	4,481095	-4,900785
C73	2,829025	2,431190	-5,430724	C73	0,752124	5,604384	-4,154125
H74	1,884977	4,360764	-5,220610	H74	0,401351	6,387615	-2,175121
H75	0,881080	3,830891	-3,021486	H75	0,868577	4,232107	-1,042638
H76	2,637661	-0,084617	-3,147564	H76	1,631510	2,392401	-4,847458
H77	3,622887	0,427614	-5,365610	H77	1,166985	4,543791	-5,983584
H78	3,248246	2,653965	-6,407687	H78	0,537030	6,545301	-4,652562
C79	1,116157	1,605328	-1,572825	C79	1,466707	1,827277	-2,172568
C80	-0,340749	1,713569	-1,383165	C80	0,059565	1,287973	-1,727192
C92	2,689443	3,420946	-0,562317	C92	3,718276	2,642193	-1,259829
C93	1,687283	1,817259	0,791545	C93	2,340261	1,487518	0,186670

C94	3,251552	3,459270	0,874037
H95	2,049246	4,282163	-0,790233
H96	3,470797	3,354222	-1,321456
H97	4,267560	3,057461	0,928581
H98	3,221548	4,448715	1,331044
N99	1,907108	2,188945	-0,492585
O100	2,386183	2,579115	1,645086
O101	0,960452	0,887150	1,157636
H102	2,808714	-4,445266	1,326166
N103	-1,944695	-2,472467	0,509032
C104	-1,152276	-3,411789	-0,325411
H105	-1,254276	-3,199388	-1,399464
C107	-3,175575	2,396908	-4,548633
C108	-3,331045	2,993715	-3,298652
C109	-2,397218	2,754981	-2,291275
C110	-1,299814	1,907351	-2,502464
C111	-1,167473	1,294427	-3,761210
C112	-2,089010	1,547397	-4,773052
H113	-3,896103	2,586057	-5,339253
H114	-4,176260	3,648591	-3,106121
H115	-2,520468	3,237733	-1,323814
H116	-0,342147	0,616604	-3,945697
H117	-1,962775	1,072715	-5,742507
H107	-0,605482	2,315601	-0,512655

C1_ES_Cheltop_TS2

C1	-4,270611	-3,334181	0,391654
C2	-2,197265	-4,561846	0,760893
C3	-3,535809	-4,654367	-0,014407
C4	-3,259085	-2,680708	1,363016
H5	-5,212097	-3,537685	0,912211
H6	-3,383710	-4,738425	-1,095097
H7	-3,660975	-1,881134	1,985466
H8	-4,495254	-2,689444	-0,458966
H9	-1,643496	-5,496912	0,842171
H10	-4,093285	-5,538606	0,307457
C11	-2,649951	-3,897644	2,080542
H12	-1,828602	-3,632566	2,753238
H13	-3,390500	-4,495283	2,619450
C14	0,096471	-3,389849	0,460621
N15	0,865908	-2,378601	0,260947
C16	2,199445	-2,697368	0,881588
C17	2,077814	-4,244128	1,039371
P18	-1,824999	-0,694376	-0,082670
Ir19	0,236056	-0,559728	-0,859118
C20	-2,084959	0,497509	1,271939
C21	-2,226919	2,416779	3,308323
C22	-1,896915	0,123948	2,609850

C94	4,177869	2,826490	0,193662
H95	3,611564	3,587728	-1,793709
H96	4,372968	1,974377	-1,832310
H97	5,227264	2,581196	0,360332
H98	3,966818	3,830810	0,573717
N99	2,413534	2,005931	-1,053818
O100	3,373213	1,883084	0,948665
O101	1,486310	0,719863	0,665301
H102	2,577948	-4,650626	1,803012
N103	-2,081141	-2,319603	0,523147
C104	-1,323240	-3,490878	0,029357
H105	-1,300870	-3,537355	-1,070525
C107	-2,433211	1,481414	-5,258193
C108	-2,703069	2,296030	-4,159063
C109	-1,906565	2,217683	-3,017088
C110	-0,838693	1,315391	-2,937578
C111	-0,587364	0,493972	-4,047258
C112	-1,369002	0,578415	-5,197462
H113	-3,045260	1,548319	-6,153354
H114	-3,529202	3,001140	-4,192874
H115	-2,102779	2,884706	-2,180884
H116	0,230020	-0,222274	-4,000589
H117	-1,150730	-0,061957	-6,048448
H107	-0,313217	2,030575	-1,008705

C1_ES_Cheltop_INT2

C1	-4,284394	-3,315052	0,401414
C2	-2,221698	-4,565507	0,750002
C3	-3,558142	-4,631278	-0,031672
C4	-3,270202	-2,689578	1,389504
H5	-5,228525	-3,523474	0,915211
H6	-3,403050	-4,692145	-1,113629
H7	-3,669331	-1,901107	2,027871
H8	-4,502768	-2,651283	-0,435964
H9	-1,675084	-5,506373	0,812358
H10	-4,123172	-5,518561	0,268003
C11	-2,673588	-3,926818	2,082395
H12	-1,851756	-3,683603	2,762510
H13	-3,420330	-4,530604	2,605637
C14	0,082356	-3,405084	0,478186
N15	0,862755	-2,404869	0,270551
C16	2,190204	-2,722838	0,905151
C17	2,056360	-4,265123	1,082713
P18	-1,826299	-0,684601	-0,026825
Ir19	0,290779	-0,602884	-0,908234
C20	-2,068451	0,505354	1,336931
C21	-2,198114	2,425606	3,381282
C22	-1,894127	0,128603	2,675438

C23	-2,353553	1,841687	0,964297	C23	-2,314294	1,855004	1,032772
C24	-2,420865	2,794458	1,977579	C24	-2,375411	2,806811	2,048745
C25	-1,973480	1,080882	3,621945	C25	-1,964516	1,084622	3,690158
H26	-2,523263	2,142083	-0,064254	H26	-2,476531	2,161554	0,004224
H27	-2,631503	3,830524	1,728353	H27	-2,570916	3,845823	1,799027
H28	-1,836113	0,780624	4,656952	H28	-1,839532	0,777567	4,724818
H29	-2,282029	3,160120	4,098355	H29	-2,250083	3,167201	4,172801
O30	0,641724	-4,482760	1,005016	O30	0,617049	-4,495101	1,039013
C31	2,308524	-2,075141	2,280275	C31	2,298954	-2,074210	2,291472
C32	1,320527	-1,250367	2,812633	C32	1,300584	-1,257340	2,817166
C33	3,440156	-2,367598	3,057120	C33	3,435304	-2,342590	3,070194
C34	1,459985	-0,713869	4,093988	C34	1,433107	-0,707105	4,093958
H35	0,458170	-0,996558	2,216348	H35	0,432579	-1,023216	2,221257
C36	3,580091	-1,835507	4,335874	C36	3,566831	-1,798518	4,345432
H37	4,228411	-2,995874	2,648334	H37	4,230409	-2,966781	2,667999
C38	2,587367	-1,002407	4,858891	C38	2,563092	-0,974446	4,863718
H39	0,686438	-0,056312	4,480808	H39	0,649709	-0,057141	4,473294
H40	4,465340	-2,065226	4,921885	H40	4,454885	-2,013548	4,932838
H41	2,697921	-0,581277	5,854073	H41	2,667245	-0,545515	5,855964
C42	-3,210043	-0,449196	-1,247607	C42	-3,224192	-0,409461	-1,173183
C43	-4,421750	0,117257	-0,820831	C43	-4,428471	0,164547	-0,735699
C44	-3,120075	-0,991417	-2,538353	C44	-3,147525	-0,938432	-2,470778
C45	-5,518856	0,151437	-1,680650	C45	-5,529848	0,218861	-1,589255
H46	-4,518632	0,525051	0,179847	H46	-4,513945	0,566009	0,268841
C47	-4,218927	-0,954936	-3,391579	C47	-4,251325	-0,885782	-3,317873
C48	-5,419161	-0,382179	-2,965790	C48	-5,443317	-0,305435	-2,879870
H49	-6,451402	0,594920	-1,343448	H49	-6,456037	0,668890	-1,243264
H50	-4,130626	-1,356378	-4,396163	H50	-4,171658	-1,277735	-4,327008
H51	-6,273815	-0,348704	-3,635399	H51	-6,301924	-0,258240	-3,543636
H52	-0,285583	-1,454994	-2,033056	H52	-0,294534	-1,521870	-2,040990
H53	1,706015	1,610778	-2,985633	H53	1,763996	1,590537	-2,943609
H54	-1,682918	-0,909433	2,860323	H54	-1,695967	-0,908064	2,926061
H55	-2,182111	-1,408102	-2,885373	H55	-2,215490	-1,358526	-2,829946
H56	2,523408	-4,812612	0,219737	H56	2,506177	-4,847656	0,274816
C57	5,322285	-1,381730	-1,837350	C57	5,085677	-1,290685	-1,989207
C58	4,550563	-2,505095	-2,141787	C58	4,365455	-2,462661	-2,232934
C59	3,558603	-2,933160	-1,260793	C59	3,478177	-2,950750	-1,274716
C60	3,325844	-2,255549	-0,056020	C60	3,296110	-2,283494	-0,055463
C61	4,089838	-1,115445	0,229904	C61	4,018649	-1,106223	0,178612
C62	5,083010	-0,686396	-0,652575	C62	4,908890	-0,617425	-0,780203
H63	6,101058	-1,053006	-2,519618	H63	5,779530	-0,910950	-2,733818
H64	4,719279	-3,048248	-3,067044	H64	4,491000	-2,994848	-3,171472
H65	2,950531	-3,789222	-1,536703	H65	2,897527	-3,838370	-1,507120
H66	3,907818	-0,555762	1,139656	H66	3,871881	-0,555842	1,100738
H67	5,673313	0,192458	-0,406019	H67	5,470335	0,290381	-0,574921
C68	0,056245	5,720461	-1,875603	C68	-0,007559	5,679008	-1,921578
C69	0,451845	4,430427	-1,527431	C69	0,402257	4,399047	-1,553533
C70	0,859849	3,522664	-2,513347	C70	0,868320	3,497423	-2,519195

C71	0,867548	3,925583	-3,850280	C71	0,919291	3,896545	-3,856210
C72	0,466656	5,215663	-4,201249	C72	0,503957	5,176289	-4,227463
C73	0,060692	6,115170	-3,215827	C73	0,040349	6,069650	-3,262004
H74	-0,257728	6,418251	-1,104072	H74	-0,367381	6,371656	-1,165596
H75	0,438038	4,120882	-0,484364	H75	0,352360	4,091932	-0,510894
H76	1,159995	3,218602	-4,622255	H76	1,257146	3,194005	-4,613583
H77	0,467710	5,515538	-5,245243	H77	0,539133	5,473071	-5,271747
H78	-0,251885	7,119000	-3,488865	H78	-0,283347	7,065526	-3,550919
C79	1,208645	2,088125	-2,132571	C79	1,220196	2,069598	-2,119000
C80	-0,106432	1,303807	-1,804859	C80	-0,087803	1,265586	-1,841775
C81	3,457737	2,838283	-1,164236	C81	3,406461	2,860647	-1,044793
C82	2,084958	1,572292	0,197177	C82	1,983023	1,586924	0,259823
C83	3,843637	3,012860	0,311323	C83	3,717419	3,049666	0,446996
H84	3,315333	3,789740	-1,680429	H84	3,268576	3,807599	-1,570635
H85	4,175844	2,225938	-1,724570	H85	4,163832	2,264574	-1,568998
H86	4,901370	2,850268	0,519921	H86	4,765716	2,902755	0,708502
H87	3,531753	3,983902	0,708714	H87	3,374942	4,019817	0,820717
N88	2,187114	2,121613	-1,028658	N88	2,146509	2,117464	-0,968749
O89	3,087374	1,980393	0,999836	O89	2,941710	2,013081	1,106138
O90	1,249433	0,756181	0,617528	O90	1,128129	0,776668	0,650839
H91	2,455710	-4,608715	1,992641	H91	2,422879	-4,619229	2,044333
N92	-2,049495	-2,214234	0,622235	N92	-2,055513	-2,213198	0,665236
C93	-1,367268	-3,426609	0,105935	C93	-1,382088	-3,422757	0,122587
H94	-1,416899	-3,494665	-0,990914	H94	-1,431564	-3,465963	-0,975108
C95	-2,496480	1,482293	-5,406655	C95	-2,423782	1,440246	-5,479051
C96	-2,839568	2,252882	-4,295885	C96	-2,783083	2,205633	-4,369748
C97	-2,070604	2,186262	-3,135093	C97	-2,030378	2,134208	-3,199113
C98	-0,961685	1,335117	-3,047745	C98	-0,916780	1,289464	-3,101544
C99	-0,623095	0,573673	-4,175502	C99	-0,565161	0,529594	-4,225935
C100	-1,379427	0,645499	-5,344056	C100	-1,307354	0,603594	-5,403896
H101	-3,090263	1,535550	-6,314909	H101	-3,005541	1,495559	-6,395040
H102	-3,701741	2,913017	-4,334669	H102	-3,647464	2,862370	-4,416209
H103	-2,322783	2,821537	-2,289726	H103	-2,300711	2,757683	-2,350660
H104	0,231913	-0,096415	-4,122521	H104	0,289632	-0,140033	-4,167529
H105	-1,100943	0,045596	-6,206661	H105	-1,017951	0,005467	-6,264103
H106	-0,597482	1,920986	-1,045311	H106	-0,615783	1,854357	-1,085278
H107	2,532304	-0,352007	-1,755555	H107	2,027015	-0,529438	-1,430504
H108	2,514286	-0,506386	-2,487906	H108	1,606264	-0,798939	-2,085190

C1_ES_Cheltop_TS3				C1_ES_Cheltop_PROD			
C1	-4,312000	-3,322437	0,506855	C1	-4,286520	-3,433255	0,557377
C2	-2,228695	-4,536352	0,867410	C2	-2,187377	-4,581184	1,034519
C3	-3,570544	-4,638056	0,098770	C3	-3,508585	-4,754013	0,241444
C4	-3,294897	-2,656585	1,464824	C4	-3,314131	-2,702360	1,513435
H5	-5,246358	-3,532051	1,037909	H5	-5,238264	-3,633237	1,060540
H6	-3,422934	-4,722414	-0,982623	H6	-3,334590	-4,898461	-0,829603
H7	-3,695770	-1,854008	2,084342	H7	-3,750533	-1,881319	2,082874
H8	-4,550559	-2,683811	-0,344254	H8	-4,499326	-2,836608	-0,331551

H9	-1,671929	-5,469413	0,950086	H9	-1,611043	-5,495292	1,176370
H10	-4,120002	-5,526019	0,424192	H10	-4,048363	-5,632590	0,606416
C11	-2,676873	-3,866879	2,186180	C11	-2,686442	-3,861136	2,307694
H12	-1,852297	-3,595053	2,851985	H12	-1,885748	-3,536658	2,978804
H13	-3,410744	-4,465824	2,732755	H13	-3,419062	-4,452393	2,864468
C14	0,052693	-3,345789	0,563420	C14	0,072331	-3,345417	0,700789
N15	0,844791	-2,353677	0,325540	N15	0,866858	-2,381923	0,356428
C16	2,161971	-2,643977	1,012910	C16	2,183249	-2,573539	1,092725
C17	2,011777	-4,172752	1,260762	C17	2,035995	-4,068791	1,495299
P18	-1,863207	-0,693989	-0,053029	P18	-1,851121	-0,775982	-0,038358
Ir19	0,402377	-0,702897	-0,978802	Ir19	0,389529	-0,865133	-0,935307
C20	-2,148262	0,551585	1,251311	C20	-2,152066	0,511704	1,226906
C21	-2,299444	2,580513	3,186648	C21	-2,357452	2,602339	3,092542
C22	-1,972204	0,251648	2,609521	C22	-2,056612	0,249776	2,600108
C23	-2,408299	1,880569	0,875739	C23	-2,353195	1,835586	0,800672
C24	-2,477294	2,887660	1,836430	C24	-2,447932	2,873197	1,725296
C25	-2,055969	1,260839	3,569479	C25	-2,169428	1,289336	3,525454
H26	-2,588091	2,124018	-0,165777	H26	-2,468080	2,053697	-0,256712
H27	-2,682228	3,909477	1,529525	H27	-2,605738	3,890634	1,377624
H28	-1,933705	1,012924	4,620263	H28	-2,109447	1,070034	4,588038
H29	-2,360386	3,363316	3,937030	H29	-2,440473	3,408565	3,815740
O30	0,573706	-4,398031	1,190802	O30	0,599225	-4,306768	1,456946
C31	2,218164	-1,937711	2,374916	C31	2,215711	-1,731852	2,374649
C32	1,211768	-1,085321	2,823121	C32	1,189879	-0,864957	2,743461
C33	3,312842	-2,192426	3,215421	C33	3,308338	-1,893796	3,241824
C34	1,299150	-0,483021	4,079589	C34	1,256822	-0,157227	3,944959
H35	0,371162	-0,864556	2,184224	H35	0,344884	-0,715349	2,089839
C36	3,400845	-1,595141	4,469450	C36	3,375585	-1,191504	4,441489
H37	4,113106	-2,845885	2,877271	H37	4,121090	-2,561750	2,966345
C38	2,391752	-0,732827	4,905694	C38	2,346653	-0,315376	4,796618
H39	0,511703	0,193761	4,398184	H39	0,452293	0,525520	4,200986
H40	4,259135	-1,797705	5,103492	H40	4,231366	-1,325717	5,096986
H41	2,461736	-0,260851	5,881514	H41	2,398520	0,236985	5,730655
C42	-3,257445	-0,480523	-1,219502	C42	-3,218802	-0,524706	-1,233028
C43	-4,490595	0,061757	-0,824146	C43	-4,468126	-0,011273	-0,846436
C44	-3,124688	-1,010665	-2,511487	C44	-3,028362	-0,929162	-2,562703
C45	-5,565868	0,080777	-1,711153	C45	-5,501289	0,098967	-1,774729
H46	-4,615026	0,469342	0,174215	H46	-4,632543	0,310626	0,177587
C47	-4,202763	-0,993441	-3,393732	C47	-4,064628	-0,819468	-3,489327
C48	-5,423972	-0,446724	-2,996077	C48	-5,300511	-0,304041	-3,097447
H49	-6,515332	0,505397	-1,397252	H49	-6,462844	0,500076	-1,466574
H50	-4,083037	-1,390586	-4,397037	H50	-3,898226	-1,116038	-4,520379
H51	-6,262784	-0,428759	-3,686038	H51	-6,105841	-0,211355	-3,820714
H52	-0,120143	-1,747571	-2,020912	H52	-0,045028	-1,974537	-1,943550
H53	1,705924	1,797545	-3,103234	H53	1,637235	2,100797	-3,373237
H54	-1,764667	-0,767448	2,918045	H54	-1,881583	-0,762643	2,948867
H55	-2,169271	-1,409881	-2,833806	H55	-2,061142	-1,308618	-2,876142
H56	2,477237	-4,795680	0,493493	H56	2,499788	-4,761899	0,789564

C57	5,328281	-1,524135	-1,731502	C57	5,398528	-1,813733	-1,714134
C58	4,529719	-2,639708	-1,991381	C58	4,541385	-2,901847	-1,893010
C59	3,539230	-3,015127	-1,086898	C59	3,530358	-3,151417	-0,969517
C60	3,331218	-2,289797	0,094669	C60	3,358892	-2,326012	0,150235
C61	4,131181	-1,169197	0,343319	C61	4,212202	-1,231545	0,315276
C62	5,122561	-0,791264	-0,563076	C62	5,227107	-0,980674	-0,609655
H63	6,103613	-1,232088	-2,434233	H63	6,192095	-1,620250	-2,430599
H64	4,673179	-3,214017	-2,901951	H64	4,655580	-3,551625	-2,755808
H65	2,898854	-3,856806	-1,334187	H65	2,844935	-3,973751	-1,153281
H66	3,969086	-0,572690	1,232134	H66	4,076826	-0,558155	1,151787
H67	5,739420	0,077740	-0,348227	H67	5,891424	-0,133400	-0,458319
C68	-0,068364	5,672034	-1,450077	C68	-0,218971	5,476803	-0,903713
C69	0,398999	4,371235	-1,272778	C69	0,258692	4,173788	-1,022535
C70	0,811268	3,612060	-2,375447	C70	0,731629	3,705884	-2,257196
C71	0,754237	4,171296	-3,653329	C71	0,721064	4,555210	-3,364454
C72	0,284924	5,473545	-3,832018	C72	0,242057	5,861763	-3,244811
C73	-0,128190	6,224991	-2,731954	C73	-0,228814	6,323424	-2,016294
H74	-0,387405	6,255175	-0,590556	H74	-0,583701	5,833480	0,055783
H75	0,434376	3,938545	-0,275232	H75	0,251277	3,516648	-0,155635
H76	1,054214	3,578804	-4,513925	H76	1,070741	4,190695	-4,327226
H77	0,238452	5,898302	-4,830549	H77	0,235075	6,515816	-4,111856
H78	-0,495151	7,237846	-2,870978	H78	-0,602093	7,339284	-1,923378
C79	1,234331	2,160616	-2,180669	C79	1,202844	2,260438	-2,380135
C80	-0,028667	1,299995	-1,888564	C80	-0,023708	1,324928	-2,245622
C81	3,535771	2,779196	-1,281010	C81	3,589418	2,629000	-1,625785
C82	2,163181	1,512559	0,089121	C82	2,171248	1,509691	-0,161698
C83	3,981630	2,883763	0,185494	C83	4,134573	2,679300	-0,186041
H84	3,400429	3,756041	-1,751579	H84	3,475853	3,625654	-2,062515
H85	4,215492	2,172963	-1,892436	H85	4,201226	2,010446	-2,292167
H86	5,037977	2,666304	0,345386	H86	5,156227	2,309536	-0,093192
H87	3,731636	3,853531	0,627148	H87	4,061088	3,676941	0,256763
N88	2,252544	2,093131	-1,124848	N88	2,282584	2,003428	-1,416785
O89	3,205303	1,864740	0,868029	O89	3,268966	1,791633	0,564729
O90	1,293346	0,744768	0,522096	O90	1,234611	0,872205	0,334947
H91	2,350490	-4,481807	2,247540	H91	2,381899	-4,274971	2,506027
N92	-2,095817	-2,186579	0,714740	N92	-2,112337	-2,240940	0,768604
C93	-1,409533	-3,399006	0,201883	C93	-1,384040	-3,457603	0,327998
H94	-1,459573	-3,478009	-0,893623	H94	-1,414827	-3,590080	-0,762850
C95	-2,728348	1,712190	-5,232967	C95	-2,760058	1,970142	-5,510160
C96	-2,942639	2,430669	-4,056686	C96	-2,965818	2,608997	-4,286439
C97	-2,073366	2,278924	-2,979099	C97	-2,085697	2,390993	-3,229156
C98	-0,988349	1,395038	-3,047184	C98	-0,996605	1,526163	-3,381610
C99	-0,782374	0,681325	-4,234254	C99	-0,791615	0,892765	-4,611354
C100	-1,641106	0,840875	-5,320824	C100	-1,669485	1,113286	-5,672393
H101	-3,404243	1,828893	-6,075306	H101	-3,447105	2,139060	-6,334508
H102	-3,782996	3,114304	-3,978144	H102	-3,811668	3,277507	-4,154591
H103	-2,218713	2,879667	-2,086619	H103	-2,232506	2,911938	-2,287743
H104	0,042985	-0,024426	-4,302397	H104	0,050277	0,213973	-4,734993

H105	-1,467376	0,277014	-6,233173
H106	-0,488911	1,729658	-0,998898
H107	1,900232	-0,915987	-1,512540
H108	0,778736	-0,055882	-2,351456

H105	-1,505450	0,613999	-6,623100
H106	-0,507002	1,525374	-1,295915
H107	1,854834	-1,015969	-1,578418
H108	0,372212	0,246418	-2,390326

C1_Z_S_tN_Chelbot

C1_ZS_Chelbot_SM

C1	-3,834726	-3,440265	-1,131187
C2	-1,572816	-4,361917	-1,123160
C3	-2,812893	-4,200243	-2,041485
C4	-3,021692	-3,235666	0,165912
H5	-4,724173	-4,045921	-0,928194
H6	-2,575031	-3,653776	-2,959722
H7	-3,602548	-2,928361	1,036048
H8	-4,166707	-2,490636	-1,552410
H9	-0,861328	-5,124834	-1,439320
H10	-3,191657	-5,183073	-2,335190
C11	-2,229218	-4,551012	0,263373
H12	-1,512414	-4,571388	1,089879
H13	-2,868602	-5,436774	0,313151
C14	0,434905	-2,948415	-0,321957
N15	1,051812	-1,877563	0,044736
C16	2,422087	-2,262006	0,566694
C17	2,271672	-3,816161	0,630724
P18	-1,808214	-0,647503	0,317043
Ir19	0,421412	0,131843	-0,397577
C20	-1,965545	-0,540345	2,136538
C21	-2,002193	-0,281379	4,929058
C22	-1,928253	-1,677905	2,953141
C23	-2,000260	0,731780	2,732831
C24	-2,023294	0,857373	4,119459
C25	-1,948875	-1,546283	4,343295
H26	-2,004269	1,625138	2,120603
H27	-2,051464	1,847789	4,564783
H28	-1,917082	-2,434786	4,967306
H29	-2,019406	-0,182267	6,010711
O30	1,053825	-4,111652	-0,113937
C31	2,677903	-1,750921	1,979388
C32	1,626172	-1,347827	2,807157
C33	3,971263	-1,819684	2,517085
C34	1,865360	-0,967490	4,128013
H35	0,614971	-1,322988	2,426435
C36	4,210325	-1,444381	3,838607
H37	4,798944	-2,155090	1,899054
C38	3,159461	-1,005775	4,646024
H39	1,030845	-0,645326	4,744237
H40	5,220649	-1,492080	4,234845
H41	3,349343	-0,704970	5,672467

C1_ZS_Chelbot_TS1

C1	-3,839587	-3,515745	-1,066689
C2	-1,575933	-4,433007	-1,087895
C3	-2,829196	-4,276061	-1,989470
C4	-3,013826	-3,318454	0,223940
H5	-4,729740	-4,118214	-0,857936
H6	-2,605583	-3,731558	-2,912478
H7	-3,587875	-3,018608	1,101266
H8	-4,171427	-2,562988	-1,481519
H9	-0,863987	-5,190538	-1,415702
H10	-3,210727	-5,260226	-2,275194
C11	-2,214637	-4,630962	0,305835
H12	-1,488869	-4,648383	1,124608
H13	-2,848735	-5,520335	0,359032
C14	0,444761	-2,993733	-0,339298
N15	1,042912	-1,913942	0,029475
C16	2,428546	-2,265621	0,510805
C17	2,340385	-3,828621	0,520510
P18	-1,807734	-0,724541	0,353267
Ir19	0,315968	0,087960	-0,358562
C20	-1,961349	-0,571470	2,168046
C21	-1,982061	-0,221112	4,945809
C22	-1,950994	-1,683645	3,017349
C23	-1,960295	0,721821	2,719103
C24	-1,975636	0,892298	4,100488
C25	-1,964036	-1,505812	4,402952
H26	-1,936613	1,594132	2,074645
H27	-1,974690	1,896473	4,514620
H28	-1,953283	-2,373454	5,056256
H29	-1,991991	-0,086167	6,023621
O30	1,105194	-4,144028	-0,186793
C31	2,692664	-1,795814	1,938142
C32	1,649040	-1,420832	2,788501
C33	3,993903	-1,870573	2,455769
C34	1,904650	-1,072818	4,115443
H35	0,632755	-1,394040	2,420868
C36	4,248527	-1,530636	3,783406
H37	4,814654	-2,182067	1,815950
C38	3,205636	-1,118599	4,614962
H39	1,077311	-0,770098	4,750946
H40	5,264009	-1,583484	4,165411
H41	3,407305	-0,844242	5,646437

C42	-3,362171	0,083848	-0,344786	C42	-3,342526	0,019026	-0,328493
C43	-4,366195	0,644260	0,453989	C43	-4,345788	0,597627	0,457650
C44	-3,536228	0,060501	-1,739925	C44	-3,502392	-0,017791	-1,725030
C45	-5,505322	1,202015	-0,132190	C45	-5,478335	1,151405	-0,143700
H46	-4,264363	0,659166	1,533363	H46	-4,249346	0,626495	1,537248
C47	-4,670570	0,622039	-2,322033	C47	-4,629942	0,540329	-2,321803
C48	-5,655535	1,207577	-1,518702	C48	-5,619089	1,134090	-1,530653
H49	-6,275271	1,633349	0,501262	H49	-6,250610	1,596775	0,476966
H50	-4,793466	0,590078	-3,401353	H50	-4,744319	0,500418	-3,401749
H51	-6,540186	1,646208	-1,970806	H51	-6,499687	1,569145	-1,994428
H52	0,808324	0,517362	1,054024	H52	0,654856	0,462048	1,110068
H53	1,980098	0,209429	-0,675304	H53	1,879183	0,443791	-0,659854
H54	-1,853471	-2,665274	2,511439	H54	-1,902489	-2,685759	2,605304
H55	-2,793125	-0,417423	-2,369373	H55	-2,752396	-0,500417	-2,342400
H56	3,097424	-4,358712	0,171070	H56	3,166532	-4,322317	0,009072
C57	5,131769	-0,895235	-2,541755	C57	5,106027	-0,719182	-2,541423
C58	4,366425	-2,048685	-2,720289	C58	4,360659	-1,876818	-2,768696
C59	3,527259	-2,496641	-1,700877	C59	3,519888	-2,373741	-1,773715
C60	3,442335	-1,807318	-0,483315	C60	3,415804	-1,730493	-0,532612
C61	4,201725	-0,639806	-0,323560	C61	4,145923	-0,552070	-0,327148
C62	5,043052	-0,191348	-1,341975	C62	4,989452	-0,055144	-1,321605
H63	5,782992	-0,541604	-3,335702	H63	5,760323	-0,328944	-3,315256
H64	4,419302	-2,599898	-3,654736	H64	4,431878	-2,394229	-3,721179
H65	2,921882	-3,383012	-1,873017	H65	2,932837	-3,265241	-1,979851
H66	4,131118	-0,070845	0,595712	H66	4,061018	-0,022215	0,614939
H67	5,619348	0,718048	-1,198826	H67	5,547850	0,859183	-1,143519
C68	-1,038242	4,284989	2,059129	C68	-0,997895	4,388338	1,966126
C69	-0,328236	3,512750	1,144220	C69	-0,275695	3,589226	1,083006
C70	-0,968516	2,947614	0,025963	C70	-0,905394	2,964960	-0,006302
C71	-2,342387	3,181662	-0,135903	C71	-2,283569	3,173987	-0,178956
C72	-3,053196	3,953527	0,784255	C72	-3,005924	3,974075	0,705983
C73	-2,406683	4,511475	1,885187	C73	-2,368563	4,586817	1,783752
H74	-0,519827	4,711517	2,913202	H74	-0,484962	4,860748	2,799164
H75	0,727512	3,342432	1,318330	H75	0,784620	3,447288	1,262401
H76	-2,871526	2,731724	-0,967181	H76	-2,804020	2,679889	-0,991040
H77	-4,118696	4,105315	0,638271	H77	-4,073110	4,105740	0,552366
H78	-2,958164	5,115122	2,600003	H78	-2,928829	5,212893	2,471882
C79	-0,214418	2,117247	-0,965847	C79	-0,161051	2,072606	-0,951256
C80	1,228678	2,056915	-0,985404	C80	1,305669	1,972765	-0,937986
H81	1,732139	2,471968	-0,118569	H81	1,785944	2,397967	-0,061938
C82	2,004538	2,282510	-2,252542	C82	2,089233	2,223535	-2,193803
C83	2,363752	3,616100	-2,516649	C83	2,526959	3,542133	-2,398084
C84	2,366656	1,294030	-3,171265	C84	2,355981	1,259032	-3,167415
C85	3,043921	3,954199	-3,685337	C85	3,196148	3,891980	-3,569282
H86	2,099964	4,391750	-1,800550	H86	2,332324	4,296180	-1,638479
C87	3,046943	1,634824	-4,343199	C87	3,021141	1,612678	-4,342848
H88	2,122759	0,258243	-2,969180	H88	2,055327	0,231377	-3,000767
C89	3,382493	2,961741	-4,609626	C89	3,437333	2,926892	-4,550783

H90	3,310174	4,990809	-3,872581
H91	3,318514	0,852444	-5,045966
H92	3,910057	3,222872	-5,522271
C93	-1,125803	3,157944	-3,157111
C94	-0,747045	0,882959	-3,005556
C95	-1,743835	2,412567	-4,349893
H96	-0,194523	3,674919	-3,412413
H97	-1,816589	3,862358	-2,692558
H98	-2,832711	2,338941	-4,267697
H99	-1,464594	2,816339	-5,322827
N100	-0,848723	2,017238	-2,271389
O101	-1,202356	1,062326	-4,251434
O102	-0,319315	-0,213301	-2,608826
H103	2,118656	-4,163603	1,653599
N104	-1,909566	-2,277621	-0,095536
C105	-0,927416	-2,963677	-0,971016
H106	-0,822518	-2,457924	-1,938426

C1_ZS_Chelbot_INT1

C1	-3,837347	-3,522150	-0,960991
C2	-1,577798	-4,444827	-0,996165
C3	-2,846078	-4,311452	-1,879954
C4	-2,994721	-3,304616	0,315783
H5	-4,730618	-4,111033	-0,728504
H6	-2,638445	-3,794510	-2,822435
H7	-3,558584	-2,991075	1,194818
H8	-4,166186	-2,575666	-1,392669
H9	-0,867489	-5,205364	-1,320568
H10	-3,235218	-5,302323	-2,130114
C11	-2,194007	-4,615715	0,410416
H12	-1,456471	-4,615400	1,218924
H13	-2,826998	-5,503814	0,490653
C14	0,469288	-2,972915	-0,364508
N15	1,035737	-1,884271	0,018868
C16	2,457731	-2,171467	0,385715
C17	2,494496	-3,734084	0,232243
P18	-1,802125	-0,710518	0,394982
Ir19	0,167131	0,087492	-0,298276
C20	-1,975078	-0,523245	2,198599
C21	-2,013227	-0,122602	4,966202
C22	-1,937078	-1,619906	3,066637
C23	-2,007600	0,781062	2,723329
C24	-2,032739	0,975654	4,100855
C25	-1,960534	-1,416572	4,448488
H26	-1,991213	1,640252	2,059728
H27	-2,056371	1,986385	4,497870
H28	-1,928516	-2,271166	5,118102
H29	-2,029689	0,032236	6,041196

H90	3,528618	4,915853	-3,715551
H91	3,220341	0,852245	-5,092339
H92	3,954929	3,199227	-5,466134
C93	-0,989990	3,153900	-3,169383
C94	-0,710500	0,865578	-3,000873
C95	-1,623077	2,426759	-4,366593
H96	-0,040547	3,639187	-3,417788
H97	-1,660992	3,883060	-2,712641
H98	-2,715191	2,398751	-4,299517
H99	-1,314991	2,812743	-5,338104
N100	-0,759634	2,007861	-2,278074
O101	-1,140133	1,054879	-4,254258
O102	-0,338831	-0,247097	-2,588449
H103	2,242713	-4,219143	1,534474
N104	-1,905234	-2,358794	-0,037298
C105	-0,938625	-3,029816	-0,943366
H106	-0,869127	-2,520223	-1,912415

C1_ZS_Chelbot_TS2

C1	-3,837789	-3,593314	-0,714641
C2	-1,564852	-4,485327	-0,782117
C3	-2,865712	-4,414655	-1,625164
C4	-2,938812	-3,254563	0,493634
H5	-4,691368	-4,196052	-0,386981
H6	-2,698177	-3,943045	-2,598883
H7	-3,459616	-2,871908	1,371848
H8	-4,230519	-2,698194	-1,196010
H9	-0,868271	-5,270108	-1,076028
H10	-3,246983	-5,422190	-1,812515
C11	-2,123785	-4,549971	0,656300
H12	-1,353407	-4,492479	1,430922
H13	-2,750074	-5,429238	0,830697
C14	0,474126	-3,004590	-0,224180
N15	1,082707	-1,910842	0,081487
C16	2,461772	-2,270252	0,585263
C17	2,387306	-3,843960	0,585499
P18	-1,767841	-0,663363	0,356286
Ir19	0,175377	0,074241	-0,508614
C20	-1,830650	-0,432306	2,165414
C21	-1,721250	-0,005526	4,934029
C22	-1,634747	-1,510546	3,038478
C23	-1,963092	0,863515	2,689047
C24	-1,909497	1,072634	4,064739
C25	-1,586712	-1,295306	4,417061
H26	-2,095532	1,710303	2,029405
H27	-2,009507	2,081840	4,453808
H28	-1,428657	-2,136741	5,085210
H29	-1,678418	0,158901	6,006819

O30	1,199418	-4,091119	-0,336952	O30	1,112646	-4,159262	-0,031778
C31	2,759205	-1,807739	1,835646	C31	2,716199	-1,806941	2,015858
C32	1,745117	-1,476803	2,737663	C32	1,754528	-1,134707	2,769821
C33	4,077543	-1,911777	2,302589	C33	3,925082	-2,169038	2,633272
C34	2,047419	-1,211038	4,074211	C34	2,003562	-0,797606	4,102323
H35	0,718739	-1,415311	2,402083	H35	0,809241	-0,869664	2,323173
C36	4,378189	-1,652038	3,637941	C36	4,176749	-1,827097	3,959325
H37	4,875053	-2,180279	1,614597	H37	4,676836	-2,718833	2,072729
C38	3,363473	-1,291093	4,527004	C38	3,216779	-1,132773	4,699304
H39	1,244250	-0,941931	4,754652	H39	1,237158	-0,272820	4,664622
H40	5,405907	-1,726610	3,981615	H40	5,122155	-2,106682	4,415400
H41	3,600024	-1,079900	5,565818	H41	3,413805	-0,864970	5,733297
C42	-3,315511	0,019933	-0,342815	C42	-3,371443	-0,056801	-0,297562
C43	-4,334176	0,617176	0,409333	C43	-4,370444	0,507813	0,504961
C44	-3,449595	-0,058037	-1,740609	C44	-3,635932	-0,286621	-1,658530
C45	-5,456940	1,149547	-0,228269	C45	-5,599769	0,865253	-0,051648
H46	-4,259051	0,675075	1,489335	H46	-4,202633	0,671441	1,562513
C47	-4,566524	0,481233	-2,372733	C47	-4,861131	0,075653	-2,210954
C48	-5,571391	1,093592	-1,616330	C48	-5,844630	0,662930	-1,408792
H49	-6,242012	1,609147	0,365332	H49	-6,366699	1,301083	0,581988
H50	-4,661563	0,410963	-3,452989	H50	-5,058169	-0,122308	-3,261030
H51	-6,444508	1,512722	-2,108110	H51	-6,802655	0,942046	-1,837729
H52	0,462729	0,495796	1,179312	H52	0,578113	0,656845	0,883632
H53	1,861260	1,043524	-0,849721	H53	1,465355	2,075189	-2,364341
H54	-1,859291	-2,627244	2,672564	H54	-1,487478	-2,510454	2,646801
H55	-2,687822	-0,556120	-2,330672	H55	-2,902092	-0,790054	-2,277264
H56	3,272865	-4,094156	-0,441231	H56	3,175801	-4,311039	-0,006108
C57	4,847820	-0,136681	-2,624073	C57	5,267274	-0,907435	-2,443701
C58	4,124017	-1,286590	-2,943968	C58	4,188968	-1,732908	-2,772732
C59	3,371984	-1,933191	-1,965124	C59	3,322853	-2,176948	-1,776615
C60	3,340941	-1,455107	-0,646628	C60	3,503959	-1,796062	-0,438524
C61	4,048334	-0,284893	-0,343871	C61	4,581429	-0,963909	-0,121026
C62	4,798190	0,366815	-1,325495	C62	5,461628	-0,532064	-1,116492
H63	5,428347	0,374294	-3,386314	H63	5,947351	-0,562061	-3,216918
H64	4,139309	-1,677536	-3,957445	H64	4,023807	-2,032829	-3,803722
H65	2,794487	-2,811871	-2,241629	H65	2,487064	-2,813848	-2,053946
H66	4,019274	0,119438	0,661801	H66	4,736720	-0,632155	0,898209
H67	5,339541	1,273895	-1,072915	H67	6,292305	0,115175	-0,850086
C68	-1,026272	4,593725	1,768782	C68	-1,314100	4,197665	1,693451
C69	-0,279187	3,743541	0,954655	C69	-0,606850	3,392347	0,806064
C70	-0,879905	3,029475	-0,094707	C70	-1,229477	2,809279	-0,315883
C71	-2,261880	3,204115	-0,292602	C71	-2,603208	3,028650	-0,474750
C72	-3,009120	4,052891	0,521691	C72	-3,320320	3,827433	0,422175
C73	-2,396205	4,756068	1,558492	C73	-2,681938	4,428811	1,504484
H74	-0,530829	5,133590	2,570976	H74	-0,796551	4,642900	2,538761
H75	0,778932	3,633701	1,164900	H75	0,447262	3,211035	0,986698
H76	-2,766475	2,640968	-1,069049	H76	-3,136556	2,553772	-1,288661
H77	-4,076519	4,153406	0,346039	H77	-4,385770	3,971783	0,266144

H78	-2,975224	5,421042	2,192414
C79	-0,131449	2,037913	-0,939082
C80	1,424826	2,098392	-0,954156
H81	1,798529	2,597557	-0,063360
C82	2,047455	2,666508	-2,203486
C83	2,314811	4,039939	-2,263699
C84	2,263533	1,875691	-3,336354
C85	2,771663	4,617833	-3,448737
H86	2,144928	4,660199	-1,386808
C87	2,713921	2,454788	-4,523295
H88	2,085275	0,805420	-3,285343
C89	2,963191	3,827524	-4,584490
H90	2,972542	5,684834	-3,486763
H91	2,872768	1,832768	-5,399818
H92	3,311619	4,278928	-5,509081
C93	-0,969772	3,055675	-3,238207
C94	-0,636837	0,786582	-2,964264
C95	-1,555897	2,257489	-4,416109
H96	-0,049273	3,582428	-3,503153
H97	-1,681185	3,764965	-2,813170
H98	-2,648590	2,207397	-4,381222
H99	-1,226570	2,605738	-5,394899
N100	-0,688826	1,955960	-2,300165
O101	-1,050853	0,899475	-4,229916
O102	-0,265761	-0,306320	-2,481778
H103	2,577112	-4,235153	1,197212
N104	-1,883906	-2,352752	0,030123
C105	-0,943369	-3,036259	-0,894683
H106	-0,914962	-2,548025	-1,877255

H78	-3,235574	5,059853	2,193189
C79	-0,394436	1,990400	-1,282199
C80	0,934290	2,735010	-1,674333
H81	1,560934	2,824439	-0,782346
C82	0,844499	4,086833	-2,375551
C83	0,235109	5,222153	-1,821270
C84	1,438762	4,210551	-3,642040
C85	0,198061	6,426272	-2,526458
H86	-0,212363	5,177098	-0,836828
C87	1,404929	5,413390	-4,347262
H88	1,932688	3,347332	-4,084370
C89	0,774121	6,527383	-3,792593
H90	-0,282879	7,291651	-2,078320
H91	1,871124	5,479253	-5,326564
H92	0,741127	7,466706	-4,337170
C93	-1,651536	2,657425	-3,558339
C94	-1,028250	0,492696	-3,106771
C95	-2,345253	1,675259	-4,510162
H96	-0,836664	3,198725	-4,041751
H97	-2,331122	3,382785	-3,112007
H98	-3,394344	1,509787	-4,247119
H99	-2,258212	1,940228	-5,563937
N100	-1,125197	1,709717	-2,550687
O101	-1,633195	0,417315	-4,300755
O102	-0,461024	-0,508073	-2,612405
H103	2,376630	-4,258659	1,594686
N104	-1,853878	-2,317861	0,066305
C105	-0,920298	-3,077369	-0,796788
H106	-0,876581	-2,664619	-1,811035
H107	2,798605	0,792432	-0,891140
H108	2,635385	0,542488	-1,580453

C1_ZS_Chelbot_INT2

C1	-3,895545	-3,652880	-0,713507
C2	-1,626151	-4,552766	-0,715590
C3	-2,910565	-4,501680	-1,584023
C4	-3,018669	-3,288625	0,504401
H5	-4,759287	-4,242718	-0,389050
H6	-2,723553	-4,057338	-2,566894
H7	-3,557316	-2,885563	1,362508
H8	-4,271790	-2,766874	-1,224608
H9	-0,924434	-5,344510	-0,977513
H10	-3,291585	-5,513052	-1,750435
C11	-2,211161	-4,581963	0,713693
H12	-1,454279	-4,507913	1,500501
H13	-2,842023	-5,456069	0,897304
C14	0,413430	-3,064571	-0,181271
N15	1,010237	-1,961689	0,102655

C1_ZS_Chelbot_TS3

C1	-3,902284	-3,697218	-0,835905
C2	-1,624458	-4,577357	-0,792315
C3	-2,881691	-4,511628	-1,699288
C4	-3,070342	-3,369664	0,423870
H5	-4,775422	-4,300515	-0,566125
H6	-2,668128	-4,037709	-2,662749
H7	-3,641581	-2,999325	1,275670
H8	-4,260816	-2,792942	-1,328562
H9	-0,905126	-5,352117	-1,057781
H10	-3,247251	-5,521263	-1,906217
C11	-2,254824	-4,660853	0,616068
H12	-1,523755	-4,603135	1,428160
H13	-2,878810	-5,548893	0,750006
C14	0,373186	-3,070636	-0,150979
N15	0,971173	-1,964390	0,137861

C16	2,407627	-2,272591	0,569457	C16	2,368904	-2,278364	0,625623
C17	2,376714	-3,843388	0,580535	C17	2,272664	-3,832500	0,768619
P18	-1,850054	-0,697769	0,340132	P18	-1,872667	-0,768230	0,391167
Ir19	0,230695	0,027190	-0,502825	Ir19	0,310792	-0,037404	-0,464531
C20	-1,933083	-0,449981	2,151729	C20	-1,963254	-0,568273	2,207142
C21	-1,837496	0,010991	4,919394	C21	-1,829772	-0,135324	4,978274
C22	-1,757045	-1,517716	3,041778	C22	-1,880591	-1,653008	3,089354
C23	-2,053569	0,852434	2,660388	C23	-1,969117	0,736699	2,725344
C24	-2,007009	1,078762	4,033933	C24	-1,907562	0,950008	4,100482
C25	-1,715042	-1,285940	4,418401	C25	-1,816000	-1,434337	4,467795
H26	-2,175141	1,692178	1,990550	H26	-2,006775	1,588260	2,057528
H27	-2,097807	2,094599	4,407569	H27	-1,910422	1,966837	4,482954
H28	-1,572515	-2,121796	5,097229	H28	-1,745190	-2,283084	5,141876
H29	-1,800681	0,188472	5,990252	H29	-1,776786	0,030755	6,050238
O30	1,085315	-4,201461	0,010998	O30	1,033922	-4,201704	0,095424
C31	2,677509	-1,767254	1,982263	C31	2,666172	-1,686209	1,997037
C32	1,685643	-1,180173	2,768317	C32	1,655712	-1,175713	2,815815
C33	3,944974	-1,984719	2,545124	C33	3,970070	-1,772112	2,506936
C34	1,960057	-0,780987	4,077975	C34	1,948423	-0,712697	4,099759
H35	0,694774	-1,033296	2,367365	H35	0,637161	-1,132140	2,458594
C36	4,219400	-1,590081	3,852437	C36	4,260951	-1,317594	3,791865
H37	4,727371	-2,451534	1,952592	H37	4,766454	-2,180831	1,891798
C38	3,227659	-0,977410	4,622285	C38	3,251877	-0,775092	4,589844
H39	1,169792	-0,322655	4,665187	H39	1,145800	-0,309440	4,710056
H40	5,208737	-1,758474	4,268057	H40	5,278593	-1,382693	4,166079
H41	3,443466	-0,664020	5,639669	H41	3,481499	-0,411723	5,587442
C42	-3,456961	-0,102161	-0,324571	C42	-3,458720	-0,105878	-0,260876
C43	-4,461872	0,469654	0,464540	C43	-4,439501	0,508904	0,524130
C44	-3,702352	-0,323572	-1,690841	C44	-3,692014	-0,279853	-1,636389
C45	-5,680596	0,838815	-0,108637	C45	-5,625991	0,960424	-0,059281
H46	-4,306063	0,629092	1,525177	H46	-4,286294	0,640900	1,589498
C47	-4,917030	0,051141	-2,259664	C47	-4,874612	0,173497	-2,215858
C48	-5,908408	0,640941	-1,469522	C48	-5,844096	0,802114	-1,427234
H49	-6,453393	1,278745	0,515101	H49	-6,381889	1,433181	0,561281
H50	-5,097872	-0,137720	-3,314384	H50	-5,048751	0,018469	-3,277422
H51	-6,858174	0,928404	-1,911057	H51	-6,768783	1,152952	-1,876328
H52	0,549595	0,583644	0,923200	H52	0,769510	0,510379	0,914300
H53	1,642442	2,071860	-2,289987	H53	1,824742	2,359993	-2,342432
H54	-1,619531	-2,524785	2,665738	H54	-1,831826	-2,666321	2,707266
H55	-2,959913	-0,821968	-2,303915	H55	-2,961431	-0,796678	-2,249286
H56	3,154592	-4,299422	-0,033262	H56	3,093835	-4,369551	0,294082
C57	4,912859	-0,644450	-2,583132	C57	4,921648	-1,009112	-2,650457
C58	4,045626	-1,711795	-2,831034	C58	4,141257	-2,161741	-2,758407
C59	3,283857	-2,245157	-1,793961	C59	3,356239	-2,575939	-1,684429
C60	3,372675	-1,727830	-0,493333	C60	3,336841	-1,850973	-0,484847
C61	4,241099	-0,656987	-0,255385	C61	4,112208	-0,688712	-0,392000
C62	5,008311	-0,121859	-1,293914	C62	4,903685	-0,275128	-1,465020
H63	5,508097	-0,225779	-3,389467	H63	5,538606	-0,687116	-3,484605

H64	3,959906	-2,125754	-3,831595
H65	2,595913	-3,058738	-2,011280
H66	4,314751	-0,226163	0,736530
H67	5,678123	0,708887	-1,090726
C68	-1,188624	4,134686	1,750431
C69	-0,478259	3,349420	0,847963
C70	-1,098358	2,781603	-0,283206
C71	-2,475114	2,991253	-0,432705
C72	-3,195723	3,770062	0,479378
C73	-2,558234	4,360044	1,568702
H74	-0,672280	4,567114	2,602948
H75	0,577330	3,173251	1,025213
H76	-3,006968	2,523965	-1,252136
H77	-4,263141	3,906525	0,329946
H78	-3,114799	4,975627	2,268852
C79	-0,261928	1,982961	-1,267334
C80	1,066715	2,737411	-1,639757
H81	1,657667	2,849603	-0,724864
C82	0,979396	4,079841	-2,351848
C83	0,356910	5,212611	-1,806403
C84	1,571383	4,199504	-3,619230
C85	0,307690	6,411315	-2,519822
H86	-0,091959	5,167363	-0,821936
C87	1,525846	5,396901	-4,332983
H88	2,070755	3,336558	-4,056117
C89	0,885166	6,509085	-3,785940
H90	-0,182826	7,275014	-2,078934
H91	1,990505	5,460564	-5,313162
H92	0,843245	7,444087	-4,337341
C93	-1,528772	2,680757	-3,518840
C94	-1,014776	0,484801	-3,066485
C95	-2,321423	1,732242	-4,424867
H96	-0,708413	3,170032	-4,046193
H97	-2,142492	3,447702	-3,046605
H98	-3,363627	1,624807	-4,108139
H99	-2,272265	1,981692	-5,484804
N100	-1,010084	1,713204	-2,526117
O101	-1,670572	0,439441	-4,235244
O102	-0,516313	-0,551943	-2,574121
H103	2,412300	-4,247834	1,593029
N104	-1,926512	-2,362183	0,077006
C105	-0,981312	-3,145705	-0,753144
H106	-0,922814	-2,756209	-1,776906
H107	1,884302	0,518632	-0,527380
H108	1,756145	0,220554	-1,314889

C1_ZS_Chelbot_PROD

H64	4,143235	-2,738437	-3,678837
H65	2,739464	-3,464016	-1,798671
H66	4,091517	-0,096501	0,515549
H67	5,504456	0,625312	-1,371579
C68	-1,125948	4,053193	1,738528
C69	-0,383698	3,313117	0,823553
C70	-0,979974	2,780156	-0,335914
C71	-2,356381	2,964756	-0,508322
C72	-3,103664	3,701497	0,415835
C73	-2,494798	4,264208	1,535379
H74	-0,634122	4,461959	2,616689
H75	0,670434	3,143767	1,015188
H76	-2,862287	2,518227	-1,355206
H77	-4,169641	3,826377	0,250844
H78	-3,073863	4,847961	2,244311
C79	-0,117395	2,087879	-1,373971
C80	1,158127	2,970867	-1,720780
H81	1,697544	3,127270	-0,782101
C82	0,953839	4,298715	-2,428380
C83	0,199081	5,350603	-1,888879
C84	1,576133	4,495239	-3,670693
C85	0,054727	6,550326	-2,587596
H86	-0,274443	5,242720	-0,921057
C87	1,435896	5,695081	-4,367427
H88	2,176342	3,695152	-4,099608
C89	0,667352	6,727353	-3,828538
H90	-0,535117	7,353264	-2,154006
H91	1,928189	5,823484	-5,327369
H92	0,553233	7,663768	-4,367105
C93	-1,341858	2,681314	-3,628340
C94	-0,932904	0,472894	-3,080181
C95	-2,225823	1,738818	-4,447768
H96	-0,516773	3,087644	-4,215855
H97	-1,882605	3,510921	-3,173386
H98	-3,256016	1,704722	-4,078956
H99	-2,213992	1,936920	-5,519546
N100	-0,842162	1,737788	-2,604328
O101	-1,633299	0,426785	-4,224314
O102	-0,478751	-0,564211	-2,569995
H103	2,178844	-4,136949	1,812087
N104	-1,981827	-2,418072	0,059691
C105	-1,001148	-3,160780	-0,767315
H106	-0,920236	-2,739986	-1,776791
H107	1,844101	0,190167	-0,909082
H108	1,016851	1,141453	-1,252165

C1_Z_R_tN_Chelbot

C1_ZR_Chelbot_SM

C1	-3,895922	-3,761888	-0,807289	C1	-3,911023	-3,442904	-0,965329
C2	-1,611516	-4,628001	-0,814296	C2	-1,654917	-4,372785	-1,080668
C3	-2,879615	-4,544402	-1,704816	C3	-2,937462	-4,192420	-1,935271
C4	-3,056682	-3,478251	0,458669	C4	-3,035457	-3,261598	0,294444
H5	-4,768217	-4,374019	-0,555182	H5	-4,791402	-4,049259	-0,728091
H6	-2,680477	-4,042137	-2,656970	H6	-2,742000	-3,632442	-2,855544
H7	-3,625711	-3,144465	1,326852	H7	-3,571256	-2,967028	1,197291
H8	-4,255978	-2,839789	-1,265641	H8	-4,260560	-2,487612	-1,358033
H9	-0,887972	-5,386720	-1,112821	H9	-0,962714	-5,132937	-1,442878
H10	-3,240886	-5,550362	-1,935843	H10	-3,334149	-5,169111	-2,225796
C11	-2,227490	-4,766916	0,597420	C11	-2,244867	-4,580882	0,332946
H12	-1,490650	-4,726185	1,405041	H12	-1,490737	-4,614585	1,124972
H13	-2,839974	-5,666642	0,704666	H13	-2,884962	-5,464971	0,399432
C14	0,379637	-3,118052	-0,154831	C14	0,390990	-2,971424	-0,353773
N15	1,009889	-2,014295	0,088871	N15	1,022528	-1,909009	0,013916
C16	2,406287	-2,342350	0,597189	C16	2,407443	-2,307558	0,479052
C17	2,238712	-3,874569	0,846809	C17	2,261580	-3,864107	0,505018
P18	-1,884586	-0,848083	0,416895	P18	-1,838355	-0,660972	0,350615
Ir19	0,301293	-0,167029	-0,395677	Ir19	0,382327	0,114335	-0,361366
C20	-2,006830	-0,593820	2,221393	C20	-1,997047	-0,498259	2,163913
C21	-1,965504	-0,049291	4,970549	C21	-1,988179	-0,148502	4,943616
C22	-1,993666	-1,643067	3,147286	C22	-1,869959	-1,604093	3,013718
C23	-1,976377	0,732811	2,683147	C23	-2,104079	0,789101	2,717042
C24	-1,962631	1,001893	4,048719	C24	-2,102112	0,958920	4,099060
C25	-1,974665	-1,368628	4,517379	C25	-1,869399	-1,427157	4,398861
H26	-1,950887	1,556347	1,976973	H26	-2,181556	1,659302	2,075570
H27	-1,939915	2,032487	4,391844	H27	-2,183760	1,959649	4,513403
H28	-1,962148	-2,188561	5,229816	H28	-1,769541	-2,290276	5,050508
H29	-1,951166	0,160294	6,036375	H29	-1,985690	-0,013659	6,021385
O30	1,016120	-4,249661	0,145983	O30	1,016107	-4,139259	-0,198722
C31	2,719210	-1,696690	1,942185	C31	2,706913	-1,836703	1,896549
C32	1,687964	-1,343276	2,821944	C32	1,686681	-1,425759	2,759101
C33	4,043923	-1,614331	2,390364	C33	4,010630	-1,953214	2,400467
C34	1,975249	-0,857107	4,096288	C34	1,968943	-1,081671	4,081188
H35	0,654114	-1,438278	2,515764	H35	0,666868	-1,367783	2,404916
C36	4,330796	-1,136369	3,669555	C36	4,292062	-1,615249	3,723370
H37	4,857998	-1,919662	1,740737	H37	4,813358	-2,297232	1,754612
C38	3,299247	-0,743910	4,522226	C38	3,273673	-1,166387	4,565712
H39	1,156348	-0,571742	4,750527	H39	1,159050	-0,751167	4,725345
H40	5,364684	-1,070759	3,996312	H40	5,309927	-1,699832	4,093027
H41	3,526146	-0,363505	5,514109	H41	3,496627	-0,894002	5,593210
C42	-3,466202	-0,190101	-0,258407	C42	-3,386154	0,036679	-0,351752
C43	-4,478542	0,383015	0,519570	C43	-4,437412	0,550272	0,415747
C44	-3,656243	-0,309531	-1,646717	C44	-3,513005	0,003555	-1,751945
C45	-5,654466	0,840743	-0,080383	C45	-5,583539	1,049693	-0,206959
H46	-4,356063	0,477679	1,593200	H46	-4,367779	0,571412	1,497470
C47	-4,828453	0,150374	-2,243062	C47	-4,654560	0,507128	-2,370444
C48	-5,830807	0,732526	-1,459732	C48	-5,691209	1,040649	-1,597060

H49	-6,435376	1,278214	0,535531	H49	-6,392840	1,446364	0,399296
H50	-4,968018	0,036990	-3,314963	H50	-4,742459	0,471032	-3,453101
H51	-6,747229	1,088094	-1,921989	H51	-6,582448	1,434197	-2,077303
H52	0,768505	0,254352	1,017848	H52	0,738871	0,482595	1,102281
H53	1,724053	2,564102	-2,332798	H53	1,947942	0,211072	-0,596350
H54	-1,970052	-2,672660	2,806899	H54	-1,742559	-2,598109	2,598445
H55	-2,895684	-0,783385	-2,258687	H55	-2,728184	-0,439748	-2,355317
H56	3,059862	-4,475110	0,457501	H56	3,068590	-4,391006	-0,003620
C57	4,991660	-1,403990	-2,761781	C57	5,092901	-0,927283	-2,646196
C58	4,256115	-2,589353	-2,740235	C58	4,269375	-2,035193	-2,851029
C59	3,455715	-2,894935	-1,640725	C59	3,429223	-2,479230	-1,831037
C60	3,377565	-2,026672	-0,543052	C60	3,401087	-1,830790	-0,588050
C61	4,102149	-0,828965	-0,586424	C61	4,211959	-0,702676	-0,404312
C62	4,909951	-0,525254	-1,682057	C62	5,055295	-0,259453	-1,423603
H63	5,621101	-1,166851	-3,614760	H63	5,748754	-0,579033	-3,438674
H64	4,304155	-3,277726	-3,578967	H64	4,279660	-2,555028	-3,804731
H65	2,875290	-3,813680	-1,659638	H65	2,782162	-3,332403	-2,019582
H66	4,028270	-0,125036	0,233279	H66	4,183688	-0,163721	0,535666
H67	5,471954	0,404514	-1,691203	H67	5,674687	0,617815	-1,262399
C68	-1,402244	4,278878	1,708394	C68	-1,258348	4,359429	1,974947
C69	-0,582999	3,674960	0,757209	C69	-0,494232	3,571748	1,117336
C70	-1,131351	3,080542	-0,390773	C70	-1,072780	2,945900	-0,000629
C71	-2,522014	3,068860	-0,537507	C71	-2,444334	3,144234	-0,226629
C72	-3,343085	3,676067	0,416119	C72	-3,209369	3,933587	0,633692
C73	-2,790032	4,292247	1,537199	C73	-2,623732	4,546338	1,740727
H74	-0,954303	4,739342	2,584305	H74	-0,782168	4,831908	2,829406
H75	0,490097	3,667517	0,917340	H75	0,559887	3,440894	1,335398
H76	-2,979220	2,560615	-1,379011	H76	-2,927555	2,657604	-1,065294
H77	-4,419607	3,649942	0,279652	H77	-4,270045	4,057061	0,435296
H78	-3,428908	4,769492	2,274167	H78	-3,217025	5,163940	2,408168
C79	-0,205978	2,450869	-1,423057	C79	-0,259672	2,105563	-0,938625
C80	1,099436	3,244404	-1,745554	C80	1,183331	2,057881	-0,890153
H81	1,628893	3,388465	-0,798352	H81	1,640315	2,470704	0,003330
C82	0,958265	4,564407	-2,477095	C82	2,017395	2,295927	-2,116026
C83	0,269826	5,658769	-1,934677	C83	2,420158	3,625387	-2,330373
C84	1,559312	4,710447	-3,735968	C84	2,388708	1,320183	-3,045345
C85	0,169959	6,856313	-2,643929	C85	3,153505	3,973944	-3,462733
H86	-0,186952	5,583352	-0,954823	H86	2,149118	4,389477	-1,604886
C87	1,463297	5,908410	-4,444405	C87	3,120243	1,672015	-4,181764
H88	2,109587	3,875735	-4,165320	H88	2,119311	0,284205	-2,876634
C89	0,761165	6,984962	-3,901313	C89	3,499620	2,995596	-4,398317
H90	-0,367228	7,693980	-2,207789	H90	3,455805	5,006587	-3,613165
H91	1,938853	6,000545	-5,416795	H91	3,400783	0,900464	-4,892908
H92	0,683352	7,919529	-4,449309	H92	4,069419	3,265086	-5,283021
C93	-1,440324	2,931061	-3,643066	C93	-1,052933	3,149969	-3,170443
C94	-0,959587	0,726682	-3,089247	C94	-0,699927	0,871607	-3,004040
C95	-2,302487	1,959610	-4,451759	C95	-1,608374	2,412051	-4,399016
H96	-0,635059	3,368874	-4,236449	H96	-0,106583	3,663042	-3,372847

H97	-2,007170	3,738898	-3,178476	H97	-1,764476	3,857272	-2,741883
H98	-3,328601	1,898876	-4,074182	H98	-2,701184	2,352893	-4,384631
H99	-2,305464	2,156233	-5,523808	H99	-1,266011	2,813458	-5,352711
N100	-0,898764	2,007069	-2,628628	N100	-0,828599	2,005139	-2,274738
O101	-1,671851	0,669221	-4,230032	O101	-1,091024	1,055182	-4,271082
O102	-0,486221	-0,299965	-2,592687	O102	-0,298411	-0,227067	-2,588307
H103	2,081190	-4,093352	1,904450	H103	2,150079	-4,241970	1,522430
N104	-1,977605	-2,508617	0,128879	N104	-1,928044	-2,307721	0,003075
C105	-1,005052	-3,204737	-0,750252	C105	-0,998812	-2,978426	-0,939137
H106	-0,951399	-2,745269	-1,746428	H106	-0,941017	-2,458745	-1,903402
H107	1,792663	0,188149	-0,856433				
H108	0,332760	1,550416	-0,984123				

C1_ZR_Chelbot_TS1				C1_ZR_Chelbot_INT1			
C1	-3,864759	-3,516923	-1,067888	C1	-3,907882	-3,536817	-0,962417
C2	-1,600828	-4,431882	-1,089187	C2	-1,664930	-4,499785	-0,956801
C3	-2,854837	-4,278750	-1,989746	C3	-2,921843	-4,361916	-1,855378
C4	-3,039585	-3,319404	0,223327	C4	-3,074916	-3,310526	0,318844
H5	-4,755778	-4,118286	-0,859452	H5	-4,814509	-4,104491	-0,728611
H6	-2,633169	-3,736841	-2,914779	H6	-2,696241	-3,868595	-2,806159
H7	-3,614560	-3,021147	1,100525	H7	-3,642533	-2,971044	1,185743
H8	-4,195639	-2,564345	-1,483698	H8	-4,213623	-2,592149	-1,414809
H9	-0,887309	-5,187953	-1,417097	H9	-0,965170	-5,279244	-1,258447
H10	-3,235561	-5,264266	-2,271972	H10	-3,325877	-5,351089	-2,088262
C11	-2,238363	-4,630723	0,304979	C11	-2,298196	-4,633339	0,446644
H12	-1,512358	-4,646825	1,123588	H12	-1,568846	-4,631249	1,262586
H13	-2,871083	-5,521105	0,358469	H13	-2,947156	-5,508962	0,536972
C14	0,418433	-2,990358	-0,344426	C14	0,396178	-3,060515	-0,312579
N15	1,017392	-1,909778	0,019982	N15	0,983531	-1,977408	0,055406
C16	2,403520	-2,259484	0,500815	C16	2,379121	-2,301557	0,494194
C17	2,321688	-3,823667	0,501877	C17	2,363393	-3,868664	0,400799
P18	-1,840371	-0,721124	0,345760	P18	-1,828624	-0,739123	0,373067
Ir19	0,287431	0,092211	-0,357916	Ir19	0,167751	0,014471	-0,322023
C20	-1,999137	-0,560474	2,157947	C20	-2,002419	-0,533823	2,177854
C21	-2,024060	-0,198613	4,935738	C21	-2,058397	-0,113456	4,944521
C22	-1,988501	-1,668839	3,012759	C22	-2,016855	-1,624699	3,054456
C23	-2,001892	0,734789	2,704783	C23	-1,992600	0,773519	2,695643
C24	-2,018990	0,910976	4,085486	C24	-2,025358	0,978352	4,071684
C25	-2,003735	-1,485359	4,397808	C25	-2,049241	-1,411643	4,434894
H26	-1,979577	1,604742	2,057421	H26	-1,941128	1,627695	2,028393
H27	-2,020633	1,917032	4,495065	H27	-2,015420	1,992319	4,460903
H28	-1,992779	-2,350452	5,054541	H28	-2,057770	-2,262793	5,109657
H29	-2,035222	-0,059195	6,012991	H29	-2,082024	0,048871	6,018357
O30	1,079275	-4,140173	-0,191907	O30	1,086804	-4,200282	-0,220805
C31	2,661574	-1,796163	1,931509	C31	2,638638	-1,904851	1,944521
C32	1,612886	-1,432147	2,780458	C32	1,599236	-1,544732	2,805097
C33	3,960813	-1,870081	2,454397	C33	3,937840	-2,021226	2,460233
C34	1,861288	-1,094446	4,111329	C34	1,857026	-1,261635	4,147457

H35	0,598045	-1,405448	2,408868	H35	0,586751	-1,475410	2,432622
C36	4,208285	-1,539470	3,785712	C36	4,194305	-1,745099	3,801673
H37	4,785743	-2,174077	1,816353	H37	4,755284	-2,313681	1,806563
C38	3,160205	-1,139032	4,616179	C38	3,154611	-1,354681	4,648461
H39	1,030150	-0,799896	4,745698	H39	1,033963	-0,970065	4,793890
H40	5,222331	-1,591504	4,171615	H40	5,207649	-1,830791	4,183464
H41	3,356319	-0,872528	5,650769	H41	3,357226	-1,131538	5,692032
C42	-3,376310	0,015579	-0,341393	C42	-3,329952	0,015568	-0,362756
C43	-4,386627	0,582865	0,444082	C43	-4,341373	0,618623	0,394769
C44	-3,533888	-0,020954	-1,738013	C44	-3,468612	-0,059094	-1,759878
C45	-5,524547	1,124844	-0,157832	C45	-5,464259	1,157167	-0,237127
H46	-4,291642	0,611643	1,523803	H46	-4,261148	0,674214	1,474579
C47	-4,666591	0,525989	-2,335582	C47	-4,586227	0,486031	-2,386269
C48	-5,663649	1,107125	-1,545062	C48	-5,585451	1,101433	-1,624833
H49	-6,302475	1,560747	0,462581	H49	-6,244482	1,619913	0,360472
H50	-4,778785	0,486388	-3,415759	H50	-4,685660	0,418113	-3,466218
H51	-6,548757	1,532405	-2,009430	H51	-6,459502	1,523607	-2,112357
H52	0,626889	0,460564	1,112018	H52	0,467885	0,413201	1,153718
H53	1,852467	0,443352	-0,648809	H53	1,877691	0,879817	-0,787723
H54	-1,936881	-2,672771	2,605577	H54	-1,971385	-2,637384	2,669513
H55	-2,778672	-0,495492	-2,354946	H55	-2,710435	-0,559092	-2,352973
H56	3,143119	-4,310474	-0,023792	H56	3,162040	-4,284680	-0,213991
C57	5,093236	-0,702864	-2,536596	C57	4,968388	-0,444405	-2,462052
C58	4,339454	-1,852997	-2,773952	C58	4,245945	-1,597677	-2,771766
C59	3,493654	-2,351350	-1,784179	C59	3,428778	-2,189766	-1,810405
C60	3,392427	-1,717475	-0,537950	C60	3,329812	-1,651331	-0,519367
C61	4,129472	-0,544932	-0,323087	C61	4,036636	-0,477562	-0,228499
C62	4,978448	-0,046620	-1,312524	C62	4,852481	0,118460	-1,192204
H63	5,751918	-0,311848	-3,306256	H63	5,600883	0,022483	-3,211021
H64	4,408031	-2,363266	-3,730465	H64	4,314195	-2,035279	-3,763716
H65	2,900697	-3,237236	-1,997937	H65	2,855710	-3,073592	-2,079709
H66	4,045526	-0,020054	0,621909	H66	3,957107	-0,029321	0,755770
H67	5,542323	0,862867	-1,126899	H67	5,393680	1,028120	-0,948713
C68	-1,018546	4,399570	1,971311	C68	-0,860448	4,488106	1,817789
C69	-0,294585	3,603150	1,086653	C69	-0,142710	3,641548	0,973494
C70	-0,925015	2,973972	0,000843	C70	-0,772418	2,969592	-0,085844
C71	-2,304763	3,176522	-0,168261	C71	-2,151909	3,181903	-0,264534
C72	-3,028405	3,973632	0,717655	C72	-2,869800	4,026042	0,580158
C73	-2,390716	4,590415	1,793004	C73	-2,228626	4,687129	1,628035
H74	-0,505783	4,875888	2,802251	H74	-0,343671	4,994852	2,628131
H75	0,767356	3,467625	1,261818	H75	0,914481	3,499336	1,169473
H76	-2,824680	2,679595	-0,978842	H76	-2,677011	2,648398	-1,048554
H77	-4,096673	4,100017	0,567126	H77	-3,936643	4,155340	0,420760
H78	-2,952219	5,214076	2,482271	H78	-2,784978	5,347762	2,286392
C79	-0,182349	2,081541	-0,945151	C79	-0,063987	1,974434	-0,959600
C80	1,283682	1,971606	-0,934368	C80	1,491258	1,952635	-0,971161
H81	1,769847	2,396435	-0,061141	H81	1,895851	2,481755	-0,110271
C82	2,063191	2,209391	-2,195562	C82	2,165732	2,387421	-2,246967

C83	2,503305	3,524422	-2,416308	C83	2,552612	3,726008	-2,389767
C84	2,322208	1,233306	-3,160021	C84	2,348640	1,502999	-3,314159
C85	3,168371	3,859371	-3,594266	C85	3,097648	4,177344	-3,592193
H86	2,313641	4,287365	-1,664301	H86	2,414463	4,417475	-1,561678
C87	2,983193	1,572368	-4,342021	C87	2,886986	1,956389	-4,518997
H88	2,019283	0,207858	-2,981141	H88	2,079360	0,457401	-3,196619
C89	3,402531	2,882899	-4,566045	C89	3,258731	3,294329	-4,662679
H90	3,503197	4,880574	-3,753224	H90	3,394057	5,217541	-3,693830
H91	3,176953	0,803356	-5,084104	H91	3,021086	1,261541	-5,343245
H92	3,916947	3,143612	-5,486523	H92	3,678367	3,647114	-5,600420
C93	-1,010792	3,172859	-3,158726	C93	-0,860106	3,074323	-3,222287
C94	-0,751064	0,881822	-2,993883	C94	-0,641907	0,785407	-3,004086
C95	-1,660842	2,452266	-4,351066	C95	-1,508255	2,335988	-4,405981
H96	-0,059061	3,650487	-3,415649	H96	0,088668	3,549379	-3,488266
H97	-1,671709	3,906852	-2,694850	H97	-1,521969	3,815820	-2,772391
H98	-2,752608	2,434966	-4,275335	H98	-2,601267	2,338919	-4,347608
H99	-1,356996	2,836020	-5,324804	H99	-1,183279	2,691472	-5,383603
N100	-0,782210	2,023887	-2,271215	N100	-0,633035	1,941099	-2,312165
O101	-1,190225	1,075696	-4,243615	O101	-1,066275	0,952252	-4,261755
O102	-0,384354	-0,233173	-2,584822	O102	-0,313698	-0,332843	-2,558744
H103	2,239179	-4,221656	1,514152	H103	2,375905	-4,332420	1,387800
N104	-1,931401	-2,358329	-0,035909	N104	-1,945580	-2,383268	0,025971
C105	-0,966151	-3,027444	-0,945347	C105	-1,005525	-3,100915	-0,873520
H106	-0,899334	-2,517034	-1,914169	H106	-0,951887	-2,629077	-1,863264

C1_ZR_Chelbot_TS2				C1_ZR_Chelbot_INT2			
C1	-4,265594	-2,911447	0,903202	C1	-4,350695	-2,897125	0,679996
C2	-2,396518	-4,291031	0,165889	C2	-2,435451	-4,275968	0,077365
C3	-3,898367	-4,035131	-0,120366	C3	-3,911982	-4,018420	-0,316786
C4	-2,917310	-2,661695	1,617148	C4	-3,055677	-2,647477	1,487462
H5	-5,011552	-3,251399	1,629273	H5	-5,143350	-3,240323	1,353281
H6	-4,075305	-3,733867	-1,157684	H6	-4,014260	-3,714519	-1,363519
H7	-2,982646	-2,098612	2,545761	H7	-3,189140	-2,087283	2,410874
H8	-4,654698	-2,013698	0,423532	H8	-4,710281	-2,001195	0,176063
H9	-2,006614	-5,230070	-0,225639	H9	-2,020161	-5,214869	-0,287350
H10	-4,474875	-4,946764	0,060265	H10	-4,499997	-4,930582	-0,180516
C11	-2,326206	-4,078948	1,693140	C11	-2,476944	-4,067165	1,604947
H12	-1,311902	-4,097489	2,103589	H12	-1,495429	-4,089749	2,088350
H13	-2,960318	-4,777223	2,246747	H13	-3,152024	-4,764194	2,109684
C14	-0,121802	-3,250116	-0,470988	C14	-0,121439	-3,245725	-0,430460
N15	0,759980	-2,320511	-0,391729	N15	0,775480	-2,331484	-0,330880
C16	2,114756	-2,922675	-0,616041	C16	2,119214	-2,954018	-0,597479
C17	1,758373	-4,447585	-0,738622	C17	1,743215	-4,473308	-0,677998
P18	-1,704937	-0,396317	0,284150	P18	-1,772386	-0,376043	0,245233
Ir19	0,414820	-0,129807	-0,293349	Ir19	0,492499	-0,117294	-0,203332
C20	-2,153345	0,511328	1,792027	C20	-2,264261	0,509633	1,759838
C21	-2,628221	1,934978	4,154547	C21	-2,825968	1,892088	4,135498
C22	-1,647200	0,042319	3,016967	C22	-1,815056	0,012627	2,995650

C23	-2,855496	1,722228	1,754785	C23	-2,957587	1,725415	1,722722
C24	-3,096422	2,424091	2,937023	C24	-3,240208	2,406704	2,908567
C25	-1,894255	0,744713	4,191699	C25	-2,103228	0,694946	4,174363
H26	-3,190591	2,141036	0,814286	H26	-3,254808	2,165057	0,778921
H27	-3,637962	3,364273	2,894569	H27	-3,774664	3,350875	2,861401
H28	-1,508953	0,370069	5,135530	H28	-1,760463	0,295188	5,124404
H29	-2,819112	2,483928	5,072179	H29	-3,049933	2,423756	5,055574
O30	0,305350	-4,492009	-0,736667	O30	0,291449	-4,493476	-0,696141
C31	3,013919	-2,741220	0,604716	C31	3,082186	-2,756101	0,568552
C32	2,486431	-2,362827	1,843855	C32	2,636806	-2,310285	1,817822
C33	4,371621	-3,082155	0,524347	C33	4,422356	-3,147379	0,429557
C34	3,312138	-2,267381	2,964965	C34	3,524897	-2,205613	2,890021
H35	1,432463	-2,126057	1,927854	H35	1,596985	-2,037029	1,950500
C36	5,194641	-2,991378	1,645861	C36	5,306179	-3,049492	1,502664
H37	4,793178	-3,409481	-0,421897	H37	4,780048	-3,522362	-0,525416
C38	4,669799	-2,570908	2,868809	C38	4,862554	-2,566443	2,735042
H39	2,889564	-1,955903	3,916297	H39	3,164857	-1,844209	3,849244
H40	6,246843	-3,247737	1,562029	H40	6,342390	-3,348501	1,373983
H41	5,312287	-2,491767	3,740816	H41	5,553018	-2,481575	3,569021
C42	-2,973783	-0,087851	-1,007621	C42	-3,032918	-0,069026	-1,053153
C43	-4,295991	0,248800	-0,671824	C43	-4,361120	0,277140	-0,755436
C44	-2,661149	-0,369479	-2,347382	C44	-2,689244	-0,366205	-2,382935
C45	-5,272282	0,340194	-1,661981	C45	-5,313326	0,358437	-1,769687
H46	-4,574170	0,436366	0,358944	H46	-4,662587	0,478847	0,266223
C47	-3,646470	-0,285959	-3,331568	C47	-3,649617	-0,292760	-3,392860
C48	-4,950611	0,077146	-2,994703	C48	-4,960820	0,076072	-3,091107
H49	-6,286984	0,614535	-1,388165	H49	-6,334094	0,637972	-1,524827
H50	-3,388687	-0,498364	-4,365255	H50	-3,367659	-0,518296	-4,417493
H51	-5,714413	0,149897	-3,763393	H51	-5,706445	0,139150	-3,878501
H52	0,606833	-0,127122	1,257815	H52	0,533159	-0,112644	1,364152
H53	2,634819	1,945031	-0,943192	H53	2,684589	2,064176	-0,876188
H54	-1,050745	-0,865083	3,043481	H54	-1,235837	-0,905682	3,030013
H55	-1,643052	-0,612305	-2,629515	H55	-1,664799	-0,614881	-2,636296
H56	2,119295	-4,912752	-1,656913	H56	2,107254	-4,969371	-1,578426
C57	3,511446	-1,200657	-4,357973	C57	3,350259	-1,387115	-4,467707
C58	2,474400	-2,135331	-4,340007	C58	2,293432	-2,292249	-4,358928
C59	2,052283	-2,686615	-3,134034	C59	1,927199	-2,791158	-3,112388
C60	2,663359	-2,329325	-1,923427	C60	2,612117	-2,410442	-1,949155
C61	3,691820	-1,380250	-1,952144	C61	3,660718	-1,490777	-2,069358
C62	4,113054	-0,819165	-3,160500	C62	4,027226	-0,983295	-3,318984
H63	3,843884	-0,773419	-5,299945	H63	3,639856	-1,000653	-5,440788
H64	1,986066	-2,424813	-5,265524	H64	1,747070	-2,601439	-5,244831
H65	1,223660	-3,390285	-3,137938	H65	1,085129	-3,475511	-3,047752
H66	4,179450	-1,089097	-1,029604	H66	4,215498	-1,179199	-1,191809
H67	4,925727	-0,097317	-3,160090	H67	4,857071	-0,284759	-3,389187
C68	1,343299	6,233598	-1,654507	C68	1,198887	6,248088	-1,818479
C69	1,152961	4,867377	-1,850035	C69	1,072993	4,865594	-1,937354
C70	1,945659	3,934266	-1,166486	C70	1,895505	4,006316	-1,194044

C71	2,925684	4,397748	-0,283650
C72	3,110953	5,766193	-0,079721
C73	2,320715	6,687526	-0,766586
H74	0,718517	6,944692	-2,187600
H75	0,368533	4,520631	-2,515903
H76	3,546461	3,683638	0,253600
H77	3,875136	6,110952	0,611055
H78	2,464311	7,753070	-0,611529
C79	1,714698	2,434511	-1,284130
C80	0,545071	1,985334	-0,320043
H81	0,942294	2,258358	0,664124
C82	-0,690208	2,826273	-0,538697
C83	-1,526114	2,661356	-1,652311
C84	-0,996731	3,859736	0,360279
C85	-2,640039	3,477883	-1,849368
H86	-1,321245	1,867815	-2,358688
C87	-2,100389	4,688124	0,163844
H88	-0,352781	4,019057	1,221034
C89	-2,932126	4,497760	-0,941536
H90	-3,282961	3,309496	-2,709210
H91	-2,310313	5,483549	0,874007
H92	-3,796525	5,137996	-1,094649
C93	2,391051	2,611983	-3,766876
C94	0,975105	0,915680	-3,159240
C95	1,705344	2,022441	-5,001092
H96	3,422897	2,255237	-3,646798
H97	2,388376	3,700905	-3,756395
H98	0,929669	2,684612	-5,399548
H99	2,390499	1,729226	-5,796980
N100	1,538125	2,056308	-2,703999
O101	1,061658	0,823442	-4,497743
O102	0,424807	0,003379	-2,523802
H103	2,105455	-5,015496	0,125902
N104	-1,926185	-2,036466	0,685811
C105	-1,611257	-3,054354	-0,345297
H106	-1,976861	-2,762982	-1,342890
H107	3,184696	0,236705	0,010217
H108	3,035165	0,236918	0,740057

C1_ZR_Chelbot_TS3

C1	-4,378606	-3,027690	0,449091
C2	-2,399283	-4,287599	-0,212623
C3	-3,873445	-4,043290	-0,626116
C4	-3,113888	-2,807595	1,310806
H5	-5,176735	-3,454033	1,066162
H6	-3,958454	-3,653164	-1,645464
H7	-3,289096	-2,328297	2,273199
H8	-4,755086	-2,102015	0,015707

C71	2,843583	4,564459	-0,331596
C72	2,965641	5,949964	-0,204699
C73	2,144353	6,797086	-0,949106
H74	0,549215	6,897440	-2,398688
H75	0,314634	4,448413	-2,592111
H76	3,491200	3,911784	0,250496
H77	3,706982	6,365945	0,471504
H78	2,239627	7,874800	-0,854434
C79	1,737191	2,490357	-1,233232
C80	0,605102	2,015829	-0,242737
H81	1,015116	2,301070	0,731798
C82	-0,653959	2,824311	-0,436937
C83	-1,499662	2,647572	-1,541205
C84	-0,959783	3,860860	0,458732
C85	-2,619370	3,456782	-1,735135
H86	-1,294833	1,854915	-2,247392
C87	-2,066832	4,684801	0,263435
H88	-0,308817	4,031783	1,312063
C89	-2,907136	4,483349	-0,833900
H90	-3,268798	3,277223	-2,587854
H91	-2,274331	5,484553	0,969317
H92	-3,774612	5,119832	-0,985318
C93	2,423898	2,556477	-3,713159
C94	0,963038	0,910143	-3,070061
C95	1,715806	1,967326	-4,934575
H96	3,446740	2,174282	-3,594589
H97	2,448771	3,645321	-3,718143
H98	0,959177	2,645886	-5,342335
H99	2,388717	1,639762	-5,727673
N100	1,565199	2,040366	-2,634777
O101	1,038656	0,798095	-4,408679
O102	0,379081	0,024021	-2,428622
H103	2,071683	-5,020397	0,207144
N104	-1,986555	-2,025764	0,644742
C105	-1,613213	-3,037865	-0,370724
H106	-1,917141	-2,742822	-1,388324
H107	2,235104	-0,062561	-0,578998
H108	2,180693	-0,027832	0,254869

C1_ZR_Chelbot_PROD

C1	-4,378606	-3,027690	0,449091
C2	-2,399283	-4,287599	-0,212623
C3	-3,873445	-4,043290	-0,626116
C4	-3,113888	-2,807595	1,310806
H5	-5,176735	-3,454033	1,066162
H6	-3,958454	-3,653164	-1,645464
H7	-3,289096	-2,328297	2,273199
H8	-4,755086	-2,102015	0,015707

H9	-1,945859	-5,179554	-0,643236
H10	-4,431990	-4,982843	-0,586428
C11	-2,489473	-4,213635	1,326896
H12	-1,520946	-4,245842	1,835469
H13	-3,152278	-4,974493	1,748723
C14	-0,106290	-3,144510	-0,569121
N15	0,777196	-2,219449	-0,410801
C16	2,151192	-2,817823	-0,582275
C17	1,795730	-4,331617	-0,776587
P18	-1,884972	-0,401510	0,276436
Ir19	0,516610	-0,091166	-0,174670
C20	-2,254069	0,359488	1,891262
C21	-2,491479	1,645197	4,370627
C22	-1,798057	-0,267141	3,062846
C23	-2,801552	1,647029	1,970346
C24	-2,920912	2,282236	3,207318
C25	-1,925921	0,369488	4,294891
H26	-3,114960	2,168770	1,073816
H27	-3,344445	3,281438	3,254547
H28	-1,579811	-0,127462	5,196822
H29	-2,587528	2,141654	5,331994
O30	0,344110	-4,370816	-0,844640
C31	2,985822	-2,697162	0,692122
C32	2,380252	-2,471807	1,932871
C33	4,361191	-2,970373	0,649928
C34	3,141876	-2,459749	3,101307
H35	1,311520	-2,300800	1,986672
C36	5,120158	-2,964980	1,819660
H37	4,844079	-3,184622	-0,298603
C38	4,515376	-2,696982	3,048172
H39	2,657789	-2,268118	4,054873
H40	6,185759	-3,169420	1,768577
H41	5,108889	-2,684373	3,957697
C42	-3,269587	0,017680	-0,861700
C43	-4,551839	0,398706	-0,437945
C44	-3,039119	-0,187254	-2,233093
C45	-5,572120	0,598045	-1,367679
H46	-4,760450	0,538173	0,617685
C47	-4,066751	-0,000257	-3,159052
C48	-5,333664	0,401748	-2,730334
H49	-6,557223	0,902835	-1,025546
H50	-3,874253	-0,158979	-4,216495
H51	-6,131488	0,556649	-3,450720
H52	0,562366	-0,390766	1,365799
H53	2,685002	1,971802	-1,117626
H54	-1,333226	-1,247151	3,005697
H55	-2,045266	-0,458891	-2,578724
H56	2,195709	-4,769962	-1,691014

H9	-1,945859	-5,179554	-0,643236
H10	-4,431990	-4,982843	-0,586428
C11	-2,489473	-4,213635	1,326896
H12	-1,520946	-4,245842	1,835469
H13	-3,152278	-4,974493	1,748723
C14	-0,106290	-3,144510	-0,569121
N15	0,777196	-2,219449	-0,410801
C16	2,151192	-2,817823	-0,582275
C17	1,795730	-4,331617	-0,776587
P18	-1,884972	-0,401510	0,276436
Ir19	0,516610	-0,091166	-0,174670
C20	-2,254069	0,359488	1,891262
C21	-2,491479	1,645197	4,370627
C22	-1,798057	-0,267141	3,062846
C23	-2,801552	1,647029	1,970346
C24	-2,920912	2,282236	3,207318
C25	-1,925921	0,369488	4,294891
H26	-3,114960	2,168770	1,073816
H27	-3,344445	3,281438	3,254547
H28	-1,579811	-0,127462	5,196822
H29	-2,587528	2,141654	5,331994
O30	0,344110	-4,370816	-0,844640
C31	2,985822	-2,697162	0,692122
C32	2,380252	-2,471807	1,932871
C33	4,361191	-2,970373	0,649928
C34	3,141876	-2,459749	3,101307
H35	1,311520	-2,300800	1,986672
C36	5,120158	-2,964980	1,819660
H37	4,844079	-3,184622	-0,298603
C38	4,515376	-2,696982	3,048172
H39	2,657789	-2,268118	4,054873
H40	6,185759	-3,169420	1,768577
H41	5,108889	-2,684373	3,957697
C42	-3,269587	0,017680	-0,861700
C43	-4,551839	0,398706	-0,437945
C44	-3,039119	-0,187254	-2,233093
C45	-5,572120	0,598045	-1,367679
H46	-4,760450	0,538173	0,617685
C47	-4,066751	-0,000257	-3,159052
C48	-5,333664	0,401748	-2,730334
H49	-6,557223	0,902835	-1,025546
H50	-3,874253	-0,158979	-4,216495
H51	-6,131488	0,556649	-3,450720
H52	0,562366	-0,390766	1,365799
H53	2,685002	1,971802	-1,117626
H54	-1,333226	-1,247151	3,005697
H55	-2,045266	-0,458891	-2,578724
H56	2,195709	-4,769962	-1,691014

C57	3,858221	-1,042968	-4,169130
C58	2,867649	-2,021125	-4,258645
C59	2,336077	-2,583068	-3,099465
C60	2,790417	-2,194554	-1,832636
C61	3,760650	-1,185846	-1,754136
C62	4,292003	-0,617820	-2,912825
H63	4,289609	-0,616598	-5,071041
H64	2,501754	-2,342710	-5,228881
H65	1,544020	-3,321161	-3,193693
H66	4,113765	-0,848815	-0,787401
H67	5,061072	0,145725	-2,829192
C68	1,267425	6,168859	-2,182380
C69	1,089014	4,787794	-2,213848
C70	1,966483	3,943095	-1,519425
C71	3,020425	4,506718	-0,794617
C72	3,195776	5,891311	-0,756804
C73	2,319642	6,724788	-1,451321
H74	0,578686	6,812693	-2,722001
H75	0,255430	4,360912	-2,762851
H76	3,710018	3,860502	-0,255127
H77	4,019319	6,316482	-0,190399
H78	2,455740	7,802153	-1,425316
C79	1,750251	2,436729	-1,455192
C80	0,673460	2,128733	-0,360302
H81	1,143442	2,615515	0,503572
C82	-0,636732	2,856515	-0,521587
C83	-1,526536	2,597372	-1,571205
C84	-0,945937	3,899458	0,364781
C85	-2,694719	3,342192	-1,723735
H86	-1,318852	1,790119	-2,258489
C87	-2,106949	4,655056	0,211602
H88	-0,262408	4,128945	1,178352
C89	-2,989624	4,376662	-0,833697
H90	-3,381904	3,098187	-2,528898
H91	-2,321268	5,460701	0,908448
H92	-3,899068	4,958890	-0,952251
C93	2,301501	2,339536	-3,949388
C94	0,873166	0,744320	-3,122369
C95	1,598722	1,605072	-5,095028
H96	3,335848	2,002917	-3,805729
H97	2,289182	3,422271	-4,068268
H98	0,843021	2,225603	-5,586954
H99	2,279890	1,187310	-5,836870
N100	1,474473	1,913520	-2,808324
O101	0,919527	0,503331	-4,441405
O102	0,289191	-0,064390	-2,385866
H103	2,097937	-4,931829	0,082197
N104	-2,045885	-2,084187	0,558289

C57	3,858221	-1,042968	-4,169130
C58	2,867649	-2,021125	-4,258645
C59	2,336077	-2,583068	-3,099465
C60	2,790417	-2,194554	-1,832636
C61	3,760650	-1,185846	-1,754136
C62	4,292003	-0,617820	-2,912825
H63	4,289609	-0,616598	-5,071041
H64	2,501754	-2,342710	-5,228881
H65	1,544020	-3,321161	-3,193693
H66	4,113765	-0,848815	-0,787401
H67	5,061072	0,145725	-2,829192
C68	1,267425	6,168859	-2,182380
C69	1,089014	4,787794	-2,213848
C70	1,966483	3,943095	-1,519425
C71	3,020425	4,506718	-0,794617
C72	3,195776	5,891311	-0,756804
C73	2,319642	6,724788	-1,451321
H74	0,578686	6,812693	-2,722001
H75	0,255430	4,360912	-2,762851
H76	3,710018	3,860502	-0,255127
H77	4,019319	6,316482	-0,190399
H78	2,455740	7,802153	-1,425316
C79	1,750251	2,436729	-1,455192
C80	0,673460	2,128733	-0,360302
H81	1,143442	2,615515	0,503572
C82	-0,636732	2,856515	-0,521587
C83	-1,526536	2,597372	-1,571205
C84	-0,945937	3,899458	0,364781
C85	-2,694719	3,342192	-1,723735
H86	-1,318852	1,790119	-2,258489
C87	-2,106949	4,655056	0,211602
H88	-0,262408	4,128945	1,178352
C89	-2,989624	4,376662	-0,833697
H90	-3,381904	3,098187	-2,528898
H91	-2,321268	5,460701	0,908448
H92	-3,899068	4,958890	-0,952251
C93	2,301501	2,339536	-3,949388
C94	0,873166	0,744320	-3,122369
C95	1,598722	1,605072	-5,095028
H96	3,335848	2,002917	-3,805729
H97	2,289182	3,422271	-4,068268
H98	0,843021	2,225603	-5,586954
H99	2,279890	1,187310	-5,836870
N100	1,474473	1,913520	-2,808324
O101	0,919527	0,503331	-4,441405
O102	0,289191	-0,064390	-2,385866
H103	2,097937	-4,931829	0,082197
N104	-2,045885	-2,084187	0,558289

C105	-1,610030	-2,987370	-0,532290	C105	-1,610030	-2,987370	-0,532290
H106	-1,900084	-2,619624	-1,528870	H106	-1,900084	-2,619624	-1,528870
H107	2,062945	0,012316	-0,199976	H107	2,062945	0,012316	-0,199976
H108	1,172634	1,006051	0,770765	H108	1,172634	1,006051	0,770765

C1_Z_R_tN_Cheltop

C1_ZR_Cheltop_SM				C1_ZR_Cheltop_TS1			
C1	-4,163822	-3,413246	0,500286	C1	-3,985440	-3,688238	-0,507837
C2	-2,022887	-4,484285	0,029911	C2	-1,734715	-4,631017	-0,543501
C3	-3,482708	-4,418509	-0,485656	C3	-3,047673	-4,600110	-1,368599
C4	-2,990179	-3,023927	1,429779	C4	-3,087472	-3,351155	0,705208
H5	-4,960655	-3,890715	1,080214	H5	-4,883957	-4,222408	-0,182525
H6	-3,537678	-4,090098	-1,528487	H6	-2,892273	-4,214193	-2,381196
H7	-3,266325	-2,517402	2,355083	H7	-3,608539	-2,947615	1,573525
H8	-4,593539	-2,548370	-0,004974	H8	-4,305188	-2,784063	-1,030201
H9	-1,449989	-5,341411	-0,322379	H9	-1,034948	-5,420752	-0,816730
H10	-3,942435	-5,409219	-0,430607	H10	-3,451332	-5,612221	-1,462982
C11	-2,222742	-4,350826	1,555913	C11	-2,280997	-4,646727	0,901562
H12	-1,291846	-4,256405	2,123303	H12	-1,506873	-4,558960	1,669698
H13	-2,827439	-5,159721	1,975102	H13	-2,906425	-5,520161	1,106645
C14	0,151678	-3,109060	-0,165865	C14	0,326072	-3,079454	-0,205062
N15	0,893574	-2,055927	-0,124078	N15	0,939385	-1,957501	-0,044093
C16	2,348581	-2,500516	-0,199586	C16	2,312987	-2,246486	0,502841
C17	2,186909	-4,027765	0,061654	C17	2,395602	-3,792228	0,269504
P18	-1,924114	-0,542219	0,464727	P18	-1,959485	-0,752685	0,333158
Ir19	0,274759	-0,006737	-0,426515	Ir19	0,057576	-0,027395	-0,574160
C20	-2,162716	0,089448	2,164020	C20	-2,264643	-0,340436	2,085234
C21	-2,175954	1,166236	4,755891	C21	-2,578878	0,396040	4,770759
C22	-1,462221	-0,543592	3,203525	C22	-2,042342	-1,275781	3,105793
C23	-2,866203	1,270886	2,439398	C23	-2,636043	0,971563	2,422520
C24	-2,872043	1,804441	3,729486	C24	-2,786680	1,335525	3,758526
C25	-1,474891	-0,012104	4,489994	C25	-2,207644	-0,908526	4,441686
H26	-3,422200	1,770175	1,654765	H26	-2,813548	1,707959	1,646633
H27	-3,430909	2,714014	3,931950	H27	-3,069697	2,354762	4,005273
H28	-0,929103	-0,512075	5,284699	H28	-2,040677	-1,643151	5,224339
H29	-2,180416	1,582866	5,758834	H29	-2,703695	0,679968	5,811790
O30	0,770845	-4,292897	-0,146065	O30	1,018348	-4,196436	0,026023
C31	3,198648	-1,909446	0,917743	C31	2,383891	-1,973353	2,009128
C32	2,602939	-1,431603	2,087810	C32	1,328318	-1,423166	2,731866
C33	4,598555	-1,958000	0,841655	C33	3,556796	-2,332258	2,692828
C34	3,385224	-0,937864	3,131366	C34	1,440773	-1,218145	4,108391
H35	1,525079	-1,409146	2,174545	H35	0,423527	-1,125694	2,225214
C36	5,380990	-1,479057	1,891304	C36	3,671468	-2,130130	4,065202
H37	5,081246	-2,358149	-0,044546	H37	4,395118	-2,753869	2,142682
C38	4,776182	-0,952864	3,034442	C38	2,610036	-1,567748	4,779095
H39	2,900404	-0,533988	4,015167	H39	0,606278	-0,778066	4,646106
H40	6,463771	-1,512751	1,811564	H40	4,589323	-2,406990	4,575872
H41	5,386241	-0,565986	3,845709	H41	2,698798	-1,404638	5,849405

C42	-3,402936	-0,059681	-0,500964	C42	-3,461673	-0,149575	-0,545760
C43	-4,683414	0,031264	0,073048	C43	-4,679724	0,053980	0,121837
C44	-3,265983	0,099566	-1,887507	C44	-3,424286	-0,001169	-1,942236
C45	-5,795759	0,294955	-0,724477	C45	-5,823651	0,422240	-0,587352
H46	-4,815878	-0,109619	1,141559	H46	-4,740781	-0,068436	1,198095
C47	-4,382791	0,355496	-2,682549	C47	-4,570765	0,359940	-2,649149
C48	-5,648482	0,457646	-2,104662	C48	-5,773174	0,580210	-1,972919
H49	-6,778963	0,367892	-0,268299	H49	-6,755678	0,583359	-0,053295
H50	-4,258583	0,492985	-3,752287	H50	-4,523722	0,472884	-3,728586
H51	-6,516452	0,664815	-2,723537	H51	-6,664589	0,867691	-2,522395
H52	-0,118265	-0,426696	-1,876820	H52	-0,510252	-0,644608	-1,887817
H53	1,756879	-0,019484	-0,994288	H53	1,397277	0,442583	-1,474525
H54	-0,898290	-1,447363	2,994827	H54	-1,721794	-2,282353	2,859900
H55	-2,280543	0,049830	-2,337724	H55	-2,495843	-0,169604	-2,477892
H56	2,759819	-4,655219	-0,620771	H56	2,982088	-4,077008	-0,606850
C57	3,440060	-1,516173	-4,289974	C57	5,278874	-0,073434	-1,818180
C58	2,651651	-2,634007	-4,010154	C58	4,545152	-1,111458	-2,396651
C59	2,347692	-2,964294	-2,691094	C59	3,583240	-1,788578	-1,648684
C60	2,827657	-2,190005	-1,625024	C60	3,359116	-1,467652	-0,302456
C61	3,616145	-1,069096	-1,919436	C61	4,079120	-0,405985	0,259426
C62	3,920989	-0,736308	-3,239883	C62	5,028943	0,286194	-0,493901
H63	3,677595	-1,258404	-5,318104	H63	6,032949	0,452262	-2,397083
H64	2,270926	-3,249316	-4,820205	H64	4,715479	-1,390389	-3,432589
H65	1,711277	-3,824846	-2,502155	H65	2,999655	-2,568986	-2,128895
H66	3,992277	-0,439695	-1,123062	H66	3,898699	-0,116723	1,287815
H67	4,533014	0,138668	-3,440284	H67	5,587728	1,096835	-0,033233
C68	-2,453897	3,302128	-3,398380	C68	0,931160	3,343253	-5,053464
C69	-1,343744	2,760440	-2,754242	C69	0,511598	3,021901	-3,761657
C70	-1,295880	2,674830	-1,354769	C70	1,250817	2,119419	-2,987345
C71	-2,394101	3,159978	-0,628321	C71	2,416733	1,552185	-3,512369
C72	-3,505105	3,700735	-1,270648	C72	2,833070	1,873488	-4,801413
C73	-3,541160	3,774906	-2,662024	C73	2,089144	2,768352	-5,575929
H74	-2,464601	3,362781	-4,483077	H74	0,352978	4,044331	-5,648205
H75	-0,513075	2,408586	-3,356781	H75	-0,378273	3,487901	-3,348754
H76	-2,371831	3,109198	0,453696	H76	2,989997	0,854594	-2,910692
H77	-4,342910	4,062848	-0,681596	H77	3,735000	1,422223	-5,204906
H78	-4,403005	4,199897	-3,167737	H78	2,411881	3,015712	-6,583039
C79	-0,105718	2,135046	-0,625453	C79	0,832097	1,869472	-1,562285
C80	1,125894	1,794340	-1,301997	C80	-0,606508	1,880847	-1,238592
H81	1,049895	1,681344	-2,378278	H81	-1,193797	1,718687	-2,138942
C82	2,448627	2,374831	-0,891429	C82	-1,284687	2,908453	-0,388149
C83	3,258886	1,897898	0,145576	C83	-2,452759	3,484804	-0,923395
C84	2,869544	3,501593	-1,616833	C84	-0,870269	3,344976	0,882602
C85	4,457747	2,542401	0,457150	C85	-3,160040	4,470630	-0,238679
H86	2,963486	1,017598	0,704094	H86	-2,818511	3,139636	-1,887186
C87	4,065072	4,145460	-1,302243	C87	-1,566655	4,346704	1,559318
H88	2,252098	3,875455	-2,430776	H88	-0,052512	2,862983	1,396188
C89	4,863186	3,667668	-0,260299	C89	-2,711491	4,918294	1,004833

H90	5,074813	2,151098	1,260956
H91	4,375024	5,015702	-1,874073
H92	5,797544	4,165669	-0,017714
C93	0,253719	4,152200	0,963127
C94	0,565857	1,992161	1,718499
C95	0,396116	4,111600	2,494225
H96	-0,600665	4,744427	0,631138
H97	1,160012	4,506365	0,458763
H98	1,140008	4,802905	2,890317
H99	-0,562381	4,255793	3,002762
N100	0,050859	2,720383	0,699010
O101	0,845388	2,755521	2,780340
O102	0,775563	0,772568	1,698640
H103	2,418786	-4,287215	1,096734
N104	-1,976677	-2,223764	0,684957
C105	-1,350842	-3,127565	-0,305465
H106	-1,566896	-2,828804	-1,342630

C1_ZR_Cheltop_INT1

C1	-3,965371	-3,603242	0,894195
C2	-1,807226	-4,586670	0,328842
C3	-3,308515	-4,668443	-0,044655
C4	-2,746872	-3,064935	1,680435
H5	-4,688654	-4,056746	1,579988
H6	-3,482983	-4,463705	-1,105671
H7	-2,981112	-2,496035	2,580074
H8	-4,477660	-2,811469	0,345697
H9	-1,203606	-5,431669	-0,001192
H10	-3,688950	-5,672947	0,161330
C11	-1,878268	-4,322733	1,849076
H12	-0,909819	-4,114419	2,314479
H13	-2,383090	-5,125848	2,393340
C14	0,233641	-3,113761	-0,248708
N15	0,898406	-2,015381	-0,211861
C16	2,362284	-2,337685	-0,304198
C17	2,306569	-3,856450	-0,681605
P18	-1,877231	-0,606696	0,489458
Ir19	0,052281	-0,009391	-0,441195
C20	-2,044303	0,117001	2,161606
C21	-1,972406	1,380194	4,668642
C22	-1,302231	-0,436064	3,217533
C23	-2,746097	1,312634	2,375558
C24	-2,710630	1,937755	3,623393
C25	-1,273186	0,188122	4,462641
H26	-3,332616	1,749932	1,575627
H27	-3,270119	2,856042	3,779407
H28	-0,697809	-0,251712	5,271768
H29	-1,945254	1,867889	5,638509

H90	-4,061421	4,888643	-0,678137
H91	-1,221336	4,661866	2,540376
H92	-3,254796	5,691960	1,539509
C93	2,382860	3,788424	-0,767224
C94	1,894209	1,996902	0,636393
C95	3,297123	3,777524	0,475681
H96	1,629901	4,584521	-0,736266
H97	2,941272	3,854964	-1,702679
H98	4,319853	3,477591	0,230771
H99	3,305754	4,720004	1,023389
N100	1,746671	2,480445	-0,618967
O101	2,735547	2,756401	1,350476
O102	1,327054	1,001924	1,100646
H103	2,750948	-4,333692	1,144298
N104	-1,989625	-2,441503	0,280211
C105	-1,113954	-3,213520	-0,634717
H106	-1,162456	-2,841813	-1,667793

C1_ZR_Cheltop_TS2

C1	-4,124279	-3,124928	0,159666
C2	-2,071143	-4,280357	-0,462415
C3	-3,526487	-4,057611	-0,943572
C4	-2,921098	-2,931449	1,113872
H5	-4,943845	-3,611002	0,699304
H6	-3,568062	-3,608201	-1,940951
H7	-3,164399	-2,523652	2,094954
H8	-4,504241	-2,184666	-0,236744
H9	-1,558463	-5,125849	-0,919556
H10	-4,054480	-5,014082	-0,992957
C11	-2,250662	-4,315372	1,071898
H12	-1,311238	-4,357489	1,631160
H13	-2,911579	-5,122046	1,401071
C14	0,190230	-3,038574	-0,521293
N15	0,982463	-2,039426	-0,338734
C16	2,375695	-2,606747	-0,134967
C17	2,158346	-4,105806	-0,527743
P18	-1,691060	-0,440560	0,483200
Ir19	0,241647	0,088237	-0,520707
C20	-1,692013	-0,024819	2,259580
C21	-1,509535	0,659845	4,970534
C22	-1,219419	-0,956484	3,195247
C23	-2,068589	1,255335	2,694128
C24	-1,982942	1,590901	4,044238
C25	-1,131467	-0,613943	4,543471
H26	-2,443652	1,980147	1,982429
H27	-2,296176	2,577192	4,375737
H28	-0,771434	-1,345983	5,260452
H29	-1,442334	0,923942	6,021850

O30	0,926436	-4,244285	-0,422043	O30	0,717182	-4,262340	-0,571687
C31	3,041728	-2,165921	1,055857	C31	2,773039	-2,551243	1,345502
C32	2,350929	-1,707868	2,178550	C32	1,977616	-1,946896	2,314873
C33	4,394523	-2,519961	1,188266	C33	3,973539	-3,163827	1,740280
C34	3,003761	-1,578190	3,406301	C34	2,375367	-1,932983	3,653712
H35	1,311543	-1,426684	2,093481	H35	1,063006	-1,456726	2,025763
C36	5,043550	-2,395515	2,413868	C36	4,369878	-3,155506	3,074522
H37	4,947073	-2,880981	0,324681	H37	4,612916	-3,631150	0,994762
C38	4,349258	-1,917785	3,528417	C38	3,571038	-2,533467	4,038366
H39	2,453574	-1,203494	4,265085	H39	1,747252	-1,436035	4,387808
H40	6,091784	-2,667457	2,498531	H40	5,305137	-3,627295	3,361900
H41	4,856531	-1,814740	4,483423	H41	3,883834	-2,518877	5,078564
C42	-3,410829	-0,181838	-0,416465	C42	-3,280746	0,135662	-0,205942
C43	-4,651108	-0,192902	0,248425	C43	-4,388259	0,383808	0,621354
C44	-3,379940	0,066275	-1,795605	C44	-3,470630	0,020260	-1,590665
C45	-5,829808	0,047598	-0,455155	C45	-5,658406	0,530328	0,066376
H46	-4,698822	-0,384023	1,316619	H46	-4,273720	0,436284	1,697996
C47	-4,563028	0,303476	-2,495379	C47	-4,744342	0,154177	-2,137193
C48	-5,788971	0,296108	-1,829957	C48	-5,840110	0,412826	-1,312053
H49	-6,780164	0,040644	0,070752	H49	-6,507920	0,722742	0,715304
H50	-4,519784	0,512808	-3,559690	H50	-4,878716	0,069294	-3,211426
H51	-6,708399	0,486914	-2,375627	H51	-6,831837	0,519912	-1,741657
H52	-0,499029	-0,476815	-1,835112	H52	-0,460208	-0,276032	-1,881130
H53	1,689038	0,816110	-1,337113	H53	2,059941	2,372488	-1,299922
H54	-0,742926	-1,352240	3,058460	H54	-0,920574	-1,946289	2,868888
H55	-2,429294	0,107492	-2,313095	H55	-2,621805	-0,170656	-2,237499
H56	2,511863	-4,048623	-1,737491	H56	2,549328	-4,370108	-1,511811
C57	3,867317	0,164987	-3,532433	C57	5,158228	-0,529783	-2,743216
C58	2,847442	-0,765338	-3,753614	C58	4,260433	-1,461032	-3,267941
C59	2,403420	-1,577635	-2,712860	C59	3,389585	-2,146684	-2,422345
C60	2,966512	-1,483406	-1,428689	C60	3,404316	-1,924236	-1,038340
C61	3,984541	-0,547275	-1,219068	C61	4,302632	-0,980139	-0,522822
C62	4,432934	0,267083	-2,263058	C62	5,168410	-0,284895	-1,369847
H63	4,211869	0,804624	-4,339553	H63	5,836225	0,007455	-3,399813
H64	2,392485	-0,854220	-4,736138	H64	4,232658	-1,650516	-4,337007
H65	1,582436	-2,265987	-2,893925	H65	2,667664	-2,827673	-2,863763
H66	4,420072	-0,429997	-0,234874	H66	4,336193	-0,793349	0,544504
H67	5,210415	0,999553	-2,068916	H67	5,865262	0,434807	-0,949454
C68	-2,424056	3,268821	-3,607780	C68	-2,906270	2,929868	-3,091969
C69	-1,309563	2,726601	-2,966457	C69	-1,709520	2,469765	-2,556167
C70	-1,245890	2,651581	-1,567599	C70	-1,389169	2,663024	-1,199533
C71	-2,346575	3,139198	-0,840517	C71	-2,349622	3,290150	-0,398445
C72	-3,458254	3,682495	-1,477034	C72	-3,562865	3,738518	-0,927104
C73	-3,503651	3,752468	-2,869559	C73	-3,842072	3,577863	-2,280280
H74	-2,442109	3,318209	-4,693081	H74	-3,113906	2,776989	-4,147555
H75	-0,493242	2,358373	-3,578541	H75	-1,007090	1,952684	-3,201373
H76	-2,329196	3,075794	0,241464	H76	-2,158260	3,439189	0,656797
H77	-4,292745	4,045800	-0,883513	H77	-4,285481	4,219204	-0,273454

H78	-4,366815	4,178710	-3,372117
C79	-0,093832	2,039747	-0,824433
C80	1,247613	1,836162	-1,588043
H81	1,072242	1,759075	-2,657857
C82	2,347522	2,822712	-1,288230
C83	3,198653	2,659181	-0,188976
C84	2,472330	3,966178	-2,085879
C85	4,147058	3,634875	0,119601
H86	3,118931	1,765432	0,423230
C87	3,425807	4,938979	-1,781682
H88	1,810970	4,100688	-2,938456
C89	4,261765	4,778211	-0,674464
H90	4,798463	3,500396	0,978552
H91	3,513367	5,823056	-2,406983
H92	5,001354	5,537014	-0,435650
C93	0,229008	4,166322	0,734180
C94	0,650785	2,029694	1,487534
C95	0,431488	4,137670	2,259971
H96	-0,672629	4,704801	0,439315
H97	1,088431	4,585541	0,203589
H98	1,163227	4,859632	2,622522
H99	-0,509794	4,245409	2,808366
N100	0,105592	2,723211	0,469756
O101	0,944106	2,800521	2,536980
O102	0,884939	0,805960	1,470536
H103	2,950629	-4,473271	-0,057339
N104	-1,859897	-2,275589	0,778054
C105	-1,271037	-3,225788	-0,192738
H106	-1,623720	-3,044151	-1,220328

C1_ZR_Cheltop_INT2

C1	-4,167331	-3,142396	0,230583
C2	-2,120207	-4,319958	-0,368700
C3	-3,574095	-4,097924	-0,855289
C4	-2,963069	-2,937606	1,181343
H5	-4,990534	-3,613905	0,777507
H6	-3,611611	-3,666187	-1,860660
H7	-3,206608	-2,511553	2,154529
H8	-4,540155	-2,206299	-0,182838
H9	-1,610507	-5,175872	-0,810352
H10	-4,108041	-5,051783	-0,888602
C11	-2,300112	-4,325805	1,165775
H12	-1,360844	-4,361659	1,726012
H13	-2,964177	-5,123839	1,509209
C14	0,146935	-3,089053	-0,471259
N15	0,931379	-2,083208	-0,313602

H78	-4,778638	3,936459	-2,696539
C79	-0,029569	2,227352	-0,697608
C80	1,102930	2,767369	-1,644807
H81	0,950791	2,328878	-2,635364
C82	1,273868	4,275258	-1,797267
C83	2,518075	4,834894	-1,463912
C84	0,272066	5,136045	-2,269301
C85	2,751471	6,205992	-1,570353
H86	3,314955	4,184711	-1,107691
C87	0,502362	6,508558	-2,373769
H88	-0,693117	4,744111	-2,560874
C89	1,737337	7,052208	-2,020228
H90	3,723183	6,611196	-1,301455
H91	-0,291972	7,154971	-2,737452
H92	1,909433	8,121722	-2,100760
C93	0,342734	4,149922	1,149049
C94	0,928539	1,965472	1,537772
C95	0,635785	3,913148	2,635092
H96	-0,575311	4,708366	0,971136
H97	1,161814	4,666400	0,646886
H98	1,326996	4,634651	3,071246
H99	-0,273279	3,849496	3,241941
N100	0,240239	2,745849	0,681762
O101	1,272573	2,604302	2,661610
O102	1,244135	0,773003	1,350070
H103	2,544312	-4,797287	0,219226
N104	-1,842443	-2,125764	0,460872
C105	-1,307514	-2,942947	-0,651962
H106	-1,522293	-2,504147	-1,636701
H107	2,650155	0,421250	-1,775188
H108	2,146376	0,370561	-2,334344

C1_ZR_Cheltop_TS3

C1	-4,121495	-3,584966	-0,338868
C2	-1,920310	-4,598277	-0,615310
C3	-3,301718	-4,495156	-1,312578
C4	-3,098773	-3,308374	0,787814
H5	-4,995754	-4,106900	0,063850
H6	-3,225791	-4,076935	-2,321620
H7	-3,524812	-2,913053	1,709774
H8	-4,470339	-2,661805	-0,804314
H9	-1,279640	-5,402957	-0,975753
H10	-3,748720	-5,489164	-1,404042
C11	-2,328372	-4,637079	0,874493
H12	-1,483219	-4,599438	1,568575
H13	-2,964832	-5,493754	1,113631
C14	0,224093	-3,145207	-0,445415
N15	0,903229	-2,061157	-0,268789

C16	2,333920	-2,614577	-0,131000	C16	2,318326	-2,448174	0,115215
C17	2,142515	-4,106971	-0,551656	C17	2,318386	-3,934280	-0,344257
P18	-1,721689	-0,457490	0,521379	P18	-1,929437	-0,728332	0,358471
Ir19	0,295014	0,053284	-0,531703	Ir19	0,197622	-0,117626	-0,671197
C20	-1,727228	-0,025904	2,298803	C20	-2,113877	-0,352477	2,140993
C21	-1,544160	0,697333	5,002364	C21	-2,191517	0,348189	4,859360
C22	-1,260312	-0,944768	3,249369	C22	-1,860449	-1,313139	3,130245
C23	-2,102608	1,259967	2,717323	C23	-2,399041	0,965678	2,530260
C24	-2,015790	1,615021	4,062524	C24	-2,436957	1,311914	3,879492
C25	-1,170112	-0,582833	4,592883	C25	-1,905859	-0,963457	4,480273
H26	-2,477884	1,976285	1,996279	H26	-2,602078	1,721886	1,780870
H27	-2,327062	2,606661	4,379640	H27	-2,665016	2,335394	4,165094
H28	-0,811567	-1,305714	5,320036	H28	-1,719882	-1,721085	5,236513
H29	-1,475801	0,976409	6,049642	H29	-2,225268	0,617060	5,911184
O30	0,699674	-4,302332	-0,537972	O30	0,902961	-4,288804	-0,397266
C31	2,754898	-2,555384	1,338414	C31	2,479782	-2,420228	1,641431
C32	1,952259	-1,988914	2,325148	C32	1,442956	-2,078213	2,506262
C33	3,985499	-3,121324	1,706841	C33	3,711068	-2,818651	2,184372
C34	2,372220	-1,968822	3,656748	C34	1,631994	-2,113368	3,888960
H35	1,014189	-1,532347	2,055051	H35	0,494635	-1,752053	2,108016
C36	4,403314	-3,108147	3,034566	C36	3,900250	-2,859391	3,562842
H37	4,629938	-3,556707	0,946893	H37	4,532873	-3,079900	1,522528
C38	3,596164	-2,527025	4,016744	C38	2,857824	-2,502975	4,422623
H39	1,737722	-1,501584	4,404695	H39	0,814851	-1,820907	4,541739
H40	5,361152	-3,544804	3,302191	H40	4,861921	-3,164119	3,965751
H41	3,925081	-2,509685	5,051782	H41	3,005324	-2,528356	5,498579
C42	-3,314848	0,127499	-0,159162	C42	-3,482109	-0,140448	-0,431548
C43	-4,431264	0,370387	0,657377	C43	-4,631542	0,244516	0,270053
C44	-3,491909	0,044539	-1,548380	C44	-3,529677	-0,192304	-1,835494
C45	-5,694622	0,537279	0,091236	C45	-5,795953	0,592205	-0,418428
H46	-4,327489	0,408672	1,736023	H46	-4,625522	0,278216	1,354455
C47	-4,758606	0,199519	-2,108092	C47	-4,697992	0,135933	-2,520128
C48	-5,864683	0,444709	-1,291441	C48	-5,835227	0,538207	-1,812428
H49	-6,549065	0,725545	0,735021	H49	-6,676834	0,897612	0,139357
H50	-4,879460	0,140351	-3,185676	H50	-4,720024	0,087414	-3,605367
H51	-6,851077	0,565263	-1,729275	H51	-6,744892	0,801923	-2,344130
H52	-0,430829	-0,307752	-1,884546	H52	-0,275949	-0,697879	-2,058817
H53	2,163318	2,376647	-1,207793	H53	1,571784	2,111698	-2,462171
H54	-0,964553	-1,940735	2,938582	H54	-1,616013	-2,331718	2,850557
H55	-2,636712	-0,129670	-2,192239	H55	-2,641774	-0,480784	-2,392231
H56	2,497371	-4,332806	-1,559657	H56	2,728151	-4,094984	-1,343810
C57	4,691268	-0,156526	-2,812719	C57	4,957007	0,270457	-2,004644
C58	3,812398	-1,117050	-3,320125	C58	4,357674	-0,795060	-2,679566
C59	3,107298	-1,947570	-2,450606	C59	3,547137	-1,696520	-1,989789
C60	3,266918	-1,836901	-1,061985	C60	3,327256	-1,558162	-0,613528
C61	4,148770	-0,870997	-0,562625	C61	3,933787	-0,486633	0,054649
C62	4,858022	-0,039053	-1,433118	C62	4,740374	0,418702	-0,633485
H63	5,238483	0,494492	-3,488241	H63	5,590123	0,971869	-2,540912

H64	3,671094	-1,214816	-4,392620
H65	2,394357	-2,654043	-2,866361
H66	4,267262	-0,749384	0,507867
H67	5,536867	0,705619	-1,027192
C68	-2,747438	2,847795	-3,230580
C69	-1,556215	2,420189	-2,655721
C70	-1,290833	2,613972	-1,287574
C71	-2,298593	3,204708	-0,517348
C72	-3,504173	3,622573	-1,085748
C73	-3,730178	3,462897	-2,448791
H74	-2,913282	2,696410	-4,293750
H75	-0,815526	1,930092	-3,279253
H76	-2,150677	3,344787	0,545501
H77	-4,263516	4,076283	-0,454953
H78	-4,661596	3,796784	-2,895926
C79	0,055921	2,204535	-0,732279
C80	1,227284	2,759930	-1,624911
H81	1,133091	2,307975	-2,617502
C82	1,395878	4,267404	-1,771264
C83	2,617232	4,838930	-1,379540
C84	0,407507	5,120230	-2,285922
C85	2,840169	6,213120	-1,469608
H86	3,403347	4,196109	-0,987705
C87	0,626489	6,495883	-2,373016
H88	-0,539970	4,718861	-2,620267
C89	1,838077	7,050499	-1,960721
H90	3,793895	6,628250	-1,155230
H91	-0,157221	7,136479	-2,768593
H92	2,002243	8,122265	-2,027678
C93	0,323720	4,117108	1,133968
C94	0,874088	1,931409	1,562927
C95	0,528788	3,883566	2,635121
H96	-0,582740	4,674733	0,900810
H97	1,170022	4,634125	0,681580
H98	1,195492	4,604109	3,109300
H99	-0,413627	3,823642	3,188553
N100	0,249495	2,714356	0,662047
O101	1,158301	2,572558	2,701524
O102	1,175888	0,731286	1,406825
H103	2,579859	-4,805299	0,159903
N104	-1,880640	-2,147989	0,518467
C105	-1,352098	-2,989662	-0,581753
H106	-1,570160	-2,567614	-1,573579
H107	1,956990	0,305012	-1,078480
H108	1,512272	0,162463	-1,775920

C1_ZR_Cheltop_PROD

C1	-4,025728	-3,711207	-0,533282

H64	4,513341	-0,921663	-3,747117
H65	3,055847	-2,485134	-2,551775
H66	3,752957	-0,340661	1,112220
H67	5,201233	1,241011	-0,092441
C68	-3,543250	3,354554	-1,335046
C69	-2,310112	2,748477	-1,546375
C70	-1,268525	2,875854	-0,614451
C71	-1,510962	3,619170	0,546282
C72	-2,745277	4,238194	0,757875
C73	-3,765526	4,110895	-0,181821
H74	-4,335918	3,220443	-2,063647
H75	-2,164493	2,160094	-2,446379
H76	-0,746648	3,707611	1,308161
H77	-2,905462	4,815646	1,664108
H78	-4,727147	4,587120	-0,015909
C79	0,089119	2,258797	-0,920868
C80	0,661459	2,696300	-2,315090
H81	-0,043760	2,347956	-3,077034
C82	0,953046	4,168194	-2,547633
C83	2,268305	4,555980	-2,841512
C84	-0,042096	5,156735	-2,520278
C85	2,591093	5,892927	-3,072396
H86	3,048730	3,798748	-2,885772
C87	0,279931	6,495558	-2,746363
H88	-1,073121	4,887400	-2,324982
C89	1,596621	6,869900	-3,016898
H90	3,617028	6,170474	-3,297258
H91	-0,504199	7,247092	-2,717130
H92	1,843324	7,912928	-3,192971
C93	1,734702	3,873847	0,336456
C94	1,402777	1,734554	1,145503
C95	2,124845	3,768592	1,818127
H96	1,066762	4,708503	0,123653
H97	2,604297	3,953271	-0,317753
H98	3,114541	4,167232	2,042286
H99	1,385450	4,225748	2,484651
N100	1,063341	2,571666	0,143243
O101	2,142175	2,339305	2,082120
O102	1,116572	0,532517	1,225294
H103	2,802108	-4,598458	0,369136
N104	-2,006714	-2,424261	0,292122
C105	-1,257369	-3,200046	-0,724320
H106	-1,387310	-2,797159	-1,739490
H107	1,588143	0,144206	-1,404078
H108	-0,370591	1,038500	-1,603213

C1_Z_S_tN_Cheltop

Z1

C1	-3,899940	-3,462945	-0,928246

C2	-1,807264	-4,712122	-0,673039	C2	-1,668150	-4,447952	-0,847333
C3	-3,148361	-4,630704	-1,446877	C3	-2,908687	-4,333484	-1,771731
C4	-3,078959	-3,432975	0,658050	C4	-3,090599	-3,210004	0,365116
H5	-4,930521	-4,223701	-0,190831	H5	-4,823053	-4,005966	-0,701228
H6	-3,021104	-4,229752	-2,457491	H6	-2,664269	-3,882194	-2,738946
H7	-3,565364	-3,043351	1,552091	H7	-3,666474	-2,842694	1,214719
H8	-4,332845	-2,784916	-1,023194	H8	-4,174585	-2,527456	-1,418895
H9	-1,136493	-5,509633	-0,992897	H9	-0,964753	-5,237641	-1,112651
H10	-3,583097	-5,629721	-1,543812	H10	-3,318084	-5,328024	-1,970189
C11	-2,301168	-4,754309	0,790645	C11	-2,327892	-4,533824	0,546328
H12	-1,498472	-4,705996	1,532418	H12	-1,614970	-4,513656	1,375746
H13	-2,940741	-5,618219	0,993068	H13	-2,986109	-5,401193	0,648800
C14	0,301125	-3,214794	-0,406079	C14	0,370360	-2,969254	-0,211017
N15	0,965741	-2,110595	-0,284999	N15	0,988499	-1,863789	0,036766
C16	2,355818	-2,442367	0,238217	C16	2,304697	-2,178942	0,710469
C17	2,393949	-3,956947	-0,111306	C17	2,329803	-3,740657	0,563276
P18	-1,886818	-0,845470	0,285838	P18	-1,899614	-0,617089	0,268061
Ir19	0,248955	-0,248985	-0,656488	Ir19	0,309386	0,048477	-0,606920
C20	-2,140431	-0,418736	2,045887	C20	-2,310644	-0,370818	2,034744
C21	-2,350192	0,392929	4,722770	C21	-2,684252	0,073870	4,785902
C22	-2,029939	-1,356699	3,080893	C22	-2,077025	-1,398318	2,962364
C23	-2,338958	0,934464	2,366200	C23	-2,726802	0,886496	2,504566
C24	-2,442645	1,335066	3,695343	C24	-2,909102	1,103280	3,869272
C25	-2,143725	-0,951072	4,412294	C25	-2,269806	-1,177798	4,326294
H26	-2,421185	1,676513	1,579077	H26	-2,910648	1,699578	1,813308
H27	-2,597511	2,385252	3,926506	H27	-3,225406	2,084042	4,212163
H28	-2,066162	-1,688867	5,206264	H28	-2,084218	-1,984585	5,029492
H29	-2,435405	0,705233	5,759706	H29	-2,829001	0,245551	5,848438
O30	0,987451	-4,337825	-0,205646	O30	0,993913	-4,099585	0,103606
C31	2,409873	-2,302231	1,765189	C31	2,289491	-1,814237	2,197503
C32	1,329469	-1,879714	2,535930	C32	1,248064	-1,117821	2,806107
C33	3,599331	-2,673048	2,413392	C33	3,371952	-2,231393	2,988953
C34	1,435093	-1,810941	3,926933	C34	1,282491	-0,836438	4,173890
H35	0,406172	-1,579376	2,063881	H35	0,412056	-0,769021	2,219705
C36	3,704490	-2,611083	3,799256	C36	3,410757	-1,949644	4,351999
H37	4,455477	-2,994767	1,826034	H37	4,203187	-2,763099	2,531884
C38	2,618625	-2,175109	4,563199	C38	2,361902	-1,247629	4,952731
H39	0,583589	-1,462166	4,503088	H39	0,454965	-0,293642	4,620360
H40	4,634190	-2,898258	4,282096	H40	4,261564	-2,275693	4,943400
H41	2,699680	-2,120353	5,645068	H41	2,390688	-1,025775	6,015430
C42	-3,401752	-0,207894	-0,543923	C42	-3,345893	0,023878	-0,679990
C43	-4,597138	0,050606	0,141999	C43	-4,582329	0,324457	-0,092610
C44	-3,365291	-0,036256	-1,937711	C44	-3,225158	0,105335	-2,079509
C45	-5,727357	0,489291	-0,549415	C45	-5,661634	0,727949	-0,882137
H46	-4,645541	-0,076188	1,218955	H46	-4,712070	0,246973	0,981260
C47	-4,497072	0,399416	-2,627799	C47	-4,305171	0,503342	-2,865420
C48	-5,680581	0,669962	-1,933964	C48	-5,527331	0,824184	-2,267477
H49	-6,645849	0,689646	-0,004745	H49	-6,610820	0,963565	-0,409445

H50	-4,454895	0,533685	-3,705100	H50	-4,191816	0,563227	-3,944302
H51	-6,561052	1,012675	-2,469670	H51	-6,368294	1,139251	-2,878042
H52	-0,197633	-0,791208	-2,051810	H52	-0,265516	-0,559521	-1,919076
H53	1,328472	2,542302	-2,714972	H53	1,715643	-0,015333	-1,341261
H54	-1,831776	-2,398739	2,854276	H54	-1,721178	-2,364000	2,620082
H55	-2,441941	-0,231626	-2,477157	H55	-2,282625	-0,144566	-2,558524
H56	2,853002	-4,180348	-1,076569	H56	3,041120	-4,098945	-0,183341
C57	5,174412	0,087456	-1,870824	C57	5,365918	-0,184809	-1,638215
C58	4,596081	-1,006562	-2,516046	C58	4,614325	-1,227355	-2,185385
C59	3,716546	-1,840461	-1,827851	C59	3,657574	-1,876826	-1,408726
C60	3,408994	-1,603773	-0,482614	C60	3,437291	-1,505394	-0,074208
C61	3,985906	-0,497108	0,153203	C61	4,191208	-0,457844	0,463037
C62	4,861156	0,341000	-0,535144	C62	5,148282	0,197153	-0,314396
H63	5,863398	0,734940	-2,406074	H63	6,117034	0,320482	-2,239194
H64	4,823435	-1,208339	-3,558751	H64	4,768641	-1,531178	-3,216852
H65	3,246060	-2,655854	-2,368622	H65	3,054294	-2,657610	-1,865254
H66	3,733298	-0,272324	1,181879	H66	4,014980	-0,130184	1,479628
H67	5,301348	1,191386	-0,020742	H67	5,725647	1,006967	0,123274
C68	-3,474147	3,527540	-0,366109	C68	0,130934	2,435432	-5,297935
C69	-2,297175	3,008367	-0,897531	C69	-0,107627	2,425524	-3,922096
C70	-1,064058	3,251792	-0,278975	C70	0,879381	1,976976	-3,035478
C71	-1,029942	4,029798	0,881576	C71	2,118938	1,564644	-3,547949
C72	-2,207908	4,563477	1,409366	C72	2,358885	1,587203	-4,918048
C73	-3,431838	4,312299	0,790325	C73	1,361903	2,016342	-5,799828
H74	-4,421530	3,300699	-0,844668	H74	-0,643063	2,786226	-5,974650
H75	-2,345121	2,386749	-1,785047	H75	-1,057181	2,791352	-3,544137
H76	-0,089614	4,220703	1,387102	H76	2,884404	1,204904	-2,865238
H77	-2,165753	5,170683	2,309172	H77	3,321380	1,261014	-5,302301
H78	-4,348133	4,718486	1,208553	H78	1,548152	2,028794	-6,869894
C79	0,195135	2,675298	-0,901786	C79	0,645922	1,978365	-1,553598
C80	0,483066	3,137736	-2,357131	C80	-0,679448	1,986896	-1,018926
H81	-0,385474	2,828300	-2,948148	H81	-1,426494	1,761687	-1,768261
C82	0,745800	4,616374	-2,571059	C82	-1,250093	2,870914	0,034383
C83	1,985200	5,034546	-3,073480	C83	-2,503414	3,439309	-0,268464
C84	-0,230265	5,588154	-2,303258	C84	-0,664684	3,196604	1,269417
C85	2,256213	6,387370	-3,280946	C85	-3,130471	4,321406	0,609194
H86	2,745917	4,292210	-3,306367	H86	-2,995079	3,176174	-1,201554
C87	0,041564	6,941422	-2,503585	C87	-1,288367	4,089290	2,141216
H88	-1,207800	5,291057	-1,941767	H88	0,235253	2,701757	1,598506
C89	1,286262	7,346520	-2,987740	C89	-2,519754	4,661096	1,818717
H90	3,222597	6,691134	-3,673151	H90	-4,097111	4,741884	0,346709
H91	-0,725201	7,680073	-2,287223	H91	-0,814224	4,318097	3,091567
H92	1,494627	8,400956	-3,144402	H92	-3,002538	5,352045	2,503443
C93	2,308075	3,895438	-0,002084	C93	2,278112	3,933621	-1,094251
C94	1,618688	1,894630	0,974436	C94	2,127988	2,147301	0,383256
C95	2,931424	3,705319	1,392575	C95	3,448174	3,931379	-0,091131
H96	1,801672	4,855115	-0,112208	H96	1,538700	4,717168	-0,889796
H97	3,039883	3,797663	-0,808085	H97	2,609428	4,010113	-2,132018

H98	4,015912	3,817263	1,405341
H99	2,486390	4,365538	2,144087
N100	1,357144	2,775736	-0,028851
O101	2,616095	2,337024	1,756080
O102	1,071150	0,811152	1,188685
H103	2,850827	-4,561010	0,669708
N104	-1,969066	-2,539188	0,230697
C105	-1,161648	-3,304292	-0,753602
H106	-1,254334	-2,898491	-1,771112
H107	1,640099	0,070385	-1,392492
H108	-0,005514	1,573306	-1,104460

TS_{Z1-2}

C1	-3,985439	-3,688239	-0,507839
C2	-1,734714	-4,631018	-0,543502
C3	-3,047672	-4,600110	-1,368600
C4	-3,087472	-3,351156	0,705207
H5	-4,883956	-4,222408	-0,182527
H6	-2,892271	-4,214192	-2,381197
H7	-3,608538	-2,947617	1,573524
H8	-4,305187	-2,784063	-1,030202
H9	-1,034947	-5,420752	-0,816732
H10	-3,451331	-5,612221	-1,462984
C11	-2,280996	-4,646728	0,901560
H12	-1,506873	-4,558962	1,669697
H13	-2,906425	-5,520162	1,106643
C14	0,326072	-3,079454	-0,205062
N15	0,939385	-1,957501	-0,044093
C16	2,312987	-2,246486	0,502841
C17	2,395602	-3,792227	0,269505
P18	-1,959484	-0,752686	0,333158
Ir19	0,057576	-0,027396	-0,574160
C20	-2,264643	-0,340436	2,085234
C21	-2,578879	0,396040	4,770758
C22	-2,042342	-1,275782	3,105793
C23	-2,636043	0,971562	2,422520
C24	-2,786681	1,335525	3,758525
C25	-2,207645	-0,908526	4,441686
H26	-2,813547	1,707959	1,646632
H27	-3,069698	2,354762	4,005272
H28	-2,040678	-1,643151	5,224340
H29	-2,703697	0,679968	5,811790
O30	1,018349	-4,196435	0,026023
C31	2,383889	-1,973352	2,009129
C32	1,328316	-1,423165	2,731866
C33	3,556794	-2,332257	2,692830
C34	1,440769	-1,218143	4,108391
H35	0,423525	-1,125693	2,225213

H98	4,384992	3,607142	-0,554384
H99	3,594003	4,881060	0,423496
N100	1,715003	2,612258	-0,815776
O101	3,081656	2,931881	0,905300
O102	1,685716	1,144473	0,960867
H103	2,497492	-4,249692	1,510825
N104	-1,950436	-2,298604	0,069645
C105	-1,012849	-3,045795	-0,801422
H106	-0,946036	-2,619786	-1,811643

E2

C1	-3,953441	-3,737875	-0,490279
C2	-1,738154	-4,720753	-0,164229
C3	-2,974412	-4,797818	-1,099743
C4	-3,138153	-3,203440	0,708846
H5	-4,887552	-4,196450	-0,149715
H6	-2,718083	-4,588487	-2,143136
H7	-3,699464	-2,631179	1,447934
H8	-4,208213	-2,935662	-1,186266
H9	-1,054985	-5,567017	-0,232123
H10	-3,403487	-5,803143	-1,065182
C11	-2,403616	-4,460701	1,204462
H12	-1,695799	-4,257593	2,013833
H13	-3,080965	-5,265083	1,504614
C14	0,352381	-3,208348	0,132350
N15	0,938930	-2,084612	0,353717
C16	2,312079	-2,357608	0,896507
C17	2,399386	-3,912739	0,712715
P18	-1,888703	-0,737937	0,044494
Ir19	0,187428	-0,161789	-0,454795
C20	-2,299889	-0,139517	1,721759
C21	-2,717579	0,786808	4,333224
C22	-1,938307	-0,932229	2,824504
C23	-2,851515	1,132469	1,941525
C24	-3,062560	1,586152	3,242312
C25	-2,151059	-0,472729	4,122167
H26	-3,111424	1,772299	1,106948
H27	-3,489709	2,572316	3,397605
H28	-1,870762	-1,096577	4,965992
H29	-2,885693	1,144881	5,345105
O30	1,044412	-4,324334	0,372189
C31	2,380339	-1,948186	2,370590
C32	1,487057	-1,022863	2,919123
C33	3,378638	-2,482996	3,198089
C34	1,580104	-0,650105	4,259946
H35	0,710247	-0,587287	2,305693

C36	3,671464	-2,130128	4,065205	C36	3,477552	-2,106914	4,536833
H37	4,395116	-2,753868	2,142685	H37	4,105214	-3,184048	2,796142
C38	2,610031	-1,567746	4,779096	C38	2,575112	-1,188640	5,074459
H39	0,606274	-0,778064	4,646105	H39	0,865005	0,061946	4,662431
H40	4,589318	-2,406988	4,575876	H40	4,261272	-2,531691	5,157336
H41	2,698792	-1,404636	5,849406	H41	2,648653	-0,897423	6,118272
C42	-3,461673	-0,149576	-0,545760	C42	-3,261470	-0,210928	-1,045786
C43	-4,679724	0,053978	0,121837	C43	-4,553910	0,010705	-0,542540
C44	-3,424286	-0,001170	-1,942236	C44	-3,050097	-0,132183	-2,431178
C45	-5,823651	0,422238	-0,587352	C45	-5,601979	0,333179	-1,404339
H46	-4,740781	-0,068438	1,198095	H46	-4,746681	-0,060747	0,522902
C47	-4,570765	0,359940	-2,649149	C47	-4,100752	0,187547	-3,290547
C48	-5,773174	0,580210	-1,972919	C48	-5,378494	0,428415	-2,779273
H49	-6,755678	0,583357	-0,053294	H49	-6,593981	0,509800	-0,998717
H50	-4,523722	0,472884	-3,728585	H50	-3,920001	0,253308	-4,359546
H51	-6,664589	0,867691	-2,522395	H51	-6,194848	0,684656	-3,447917
H52	-0,510252	-0,644608	-1,887816	H52	-0,210303	-0,892075	-1,766382
H53	1,397277	0,442584	-1,474526	H53	1,502939	1,415293	-2,584303
H54	-1,721795	-2,282353	2,859900	H54	-1,482651	-1,902800	2,663060
H55	-2,495843	-0,169605	-2,477892	H55	-2,060850	-0,315823	-2,838128
H56	2,982090	-4,077008	-0,606848	H56	3,048340	-4,200828	-0,118019
C57	5,278873	-0,073433	-1,818179	C57	5,095729	-0,261516	-1,699143
C58	4,545151	-1,111456	-2,396650	C58	4,071229	-1,048389	-2,231162
C59	3,583240	-1,788577	-1,648683	C59	3,210425	-1,737586	-1,379360
C60	3,359116	-1,467651	-0,302455	C60	3,347437	-1,643033	0,013897
C61	4,079121	-0,405985	0,259428	C61	4,380192	-0,862303	0,536560
C62	5,028944	0,286194	-0,493899	C62	5,250693	-0,179801	-0,315432
H63	6,032949	0,452263	-2,397081	H63	5,771836	0,274035	-2,359980
H64	4,715477	-1,390387	-3,432588	H64	3,945300	-1,129814	-3,307375
H65	2,999654	-2,568983	-2,128895	H65	2,406824	-2,333045	-1,803902
H66	3,898700	-0,116724	1,287817	H66	4,489380	-0,752496	1,608837
H67	5,587729	1,096834	-0,033231	H67	6,046153	0,425585	0,110067
C68	0,931158	3,343255	-5,053463	C68	-0,096490	5,541896	-3,622395
C69	0,511597	3,021903	-3,761656	C69	0,233323	4,590673	-2,658792
C70	1,250815	2,119419	-2,987346	C70	0,752785	3,342021	-3,038055
C71	2,416730	1,552183	-3,512371	C71	0,922666	3,071713	-4,400955
C72	2,833066	1,873487	-4,801416	C72	0,584494	4,021052	-5,366442
C73	2,089140	2,768352	-5,575930	C73	0,075345	5,260200	-4,978737
H74	0,352975	4,044333	-5,648204	H74	-0,497683	6,502327	-3,311211
H75	-0,378273	3,487903	-3,348752	H75	0,067290	4,815320	-1,611076
H76	2,989994	0,854591	-2,910695	H76	1,321711	2,108200	-4,710440
H77	3,734994	1,422221	-5,204911	H77	0,723160	3,793130	-6,419343
H78	2,411876	3,015713	-6,583040	H78	-0,186907	6,002171	-5,727390
C79	0,832097	1,869472	-1,562286	C79	1,044248	2,245448	-2,031076
C80	-0,606508	1,880847	-1,238591	C80	-0,265587	1,673943	-1,389529
H81	-1,193798	1,718687	-2,138941	C82	-1,024984	2,666259	-0,543314
C82	-1,284687	2,908453	-0,388148	C83	-2,245642	3,184831	-1,009500
C83	-2,452759	3,484804	-0,923393	C84	-0,556433	3,127941	0,700680

C84	-0,870267	3,344976	0,882603
C85	-3,160040	4,470629	-0,238676
H86	-2,818511	3,139636	-1,887184
C87	-1,566653	4,346704	1,559319
H88	-0,052510	2,862983	1,396188
C89	-2,711489	4,918293	1,004836
H90	-4,061420	4,888642	-0,678134
H91	-1,221333	4,661866	2,540377
H92	-3,254794	5,691960	1,539512
C93	2,382863	3,788423	-0,767227
C94	1,894211	1,996902	0,636392
C95	3,297126	3,777523	0,475678
H96	1,629906	4,584521	-0,736269
H97	2,941275	3,854960	-1,702682
H98	4,319856	3,477588	0,230767
H99	3,305758	4,720002	1,023385
N100	1,746671	2,480445	-0,618968
O101	2,735550	2,756400	1,350474
O102	1,327056	1,001924	1,100645
H103	2,750948	-4,333692	1,144300
N104	-1,989624	-2,441503	0,280211
C105	-1,113953	-3,213521	-0,634717
H106	-1,162455	-2,841813	-1,667793

C85	-2,957773	4,137519	-0,279817
H86	-2,636470	2,837610	-1,961654
C87	-1,254269	4,095756	1,422746
H88	0,337837	2,698355	1,137423
C89	-2,457290	4,609929	0,935008
H90	-3,898704	4,518811	-0,667116
H91	-0,866349	4,434102	2,379931
H92	-3,002307	5,361779	1,498799
C93	2,819586	3,935730	-1,053641
C94	2,402990	1,940863	0,020020
C95	3,929974	3,603574	-0,043900
H96	2,187015	4,765531	-0,724590
H97	3,201241	4,162454	-2,050567
H98	4,844259	3,248017	-0,529630
H99	4,163537	4,417729	0,642112
N100	2,062432	2,677108	-1,043492
O101	3,390188	2,498530	0,733012
O102	1,912842	0,853126	0,391025
H103	2,684994	-4,448523	1,616242
N104	-1,982215	-2,416679	0,197585
C105	-1,042152	-3,360401	-0,435020
H106	-0,945914	-3,193184	-1,518434
H107	-0,880113	1,465577	-2,269023

$TS_{Z2\text{-}3}$

C1	-3,784872	-3,856984	-0,824496
C2	-1,569341	-4,766921	-0,344530
C3	-2,715698	-4,853284	-1,386188
C4	-3,098714	-3,339821	0,460695
H5	-4,723555	-4,364540	-0,579080
H6	-2,380221	-4,590021	-2,394433
H7	-3,746237	-2,822051	1,168502
H8	-4,016533	-3,041789	-1,512317
H9	-0,848680	-5,583639	-0,382257
H10	-3,103853	-5,874842	-1,428056
C11	-2,358647	-4,587910	0,970445
H12	-1,730163	-4,390773	1,843949
H13	-3,026571	-5,427764	1,181450
C14	0,408191	-3,184663	0,211601
N15	0,963961	-2,043648	0,424959
C16	2,234757	-2,277792	1,195318
C17	2,317787	-3,844050	1,185816
P18	-1,927185	-0,803073	-0,036520
Ir19	0,150558	-0,146561	-0,464542
C20	-2,562811	-0,283833	1,596689
C21	-3,378933	0,513153	4,155455
C22	-2,260265	-1,074369	2,718773
C23	-3,275373	0,911749	1,770899

Z3

C1	-3,856889	-3,856465	-0,553987
C2	-1,628280	-4,774443	-0,179079
C3	-2,833221	-4,880180	-1,148429
C4	-3,096015	-3,315245	0,679386
H5	-4,786109	-4,345424	-0,243383
H6	-2,554541	-4,649644	-2,181748
H7	-3,703007	-2,779465	1,409012
H8	-4,117588	-3,057258	-1,249608
H9	-0,913511	-5,595105	-0,238761
H10	-3,232298	-5,898465	-1,137363
C11	-2,337480	-4,557487	1,175392
H12	-1,658438	-4,344296	2,006428
H13	-2,997146	-5,387381	1,443955
C14	0,394613	-3,204907	0,200224
N15	0,964978	-2,064246	0,360772
C16	2,270616	-2,287010	1,077682
C17	2,390061	-3,844310	0,996259
P18	-1,951647	-0,790950	0,060752
Ir19	0,181004	-0,192588	-0,581390
C20	-2,560999	-0,235709	1,694978
C21	-3,334231	0,660460	4,243187
C22	-2,235254	-0,978315	2,842144
C23	-3,291242	0,952905	1,839028

C24	-3,682050	1,302050	3,045624	C24	-3,670455	1,396542	3,106302
C25	-2,666817	-0,677168	3,990146	C25	-2,619812	-0,532462	4,105728
H26	-3,514773	1,540266	0,922877	H26	-3,568347	1,535444	0,969954
H27	-4,231236	2,231075	3,165647	H27	-4,230128	2,322998	3,197845
H28	-2,428892	-1,298223	4,848976	H28	-2,362485	-1,121331	4,981692
H29	-3,698175	0,822053	5,146911	H29	-3,633378	1,007546	5,228153
O30	1,037374	-4,277720	0,649649	O30	1,063457	-4,291504	0,594409
C31	2,121461	-1,783421	2,639149	C31	2,186846	-1,851379	2,542679
C32	1,055323	-1,009538	3,093943	C32	1,109868	-1,131274	3,053083
C33	3,146234	-2,119910	3,536720	C33	3,247294	-2,184252	3,400119
C34	0,999821	-0,591463	4,425336	C34	1,081330	-0,753876	4,397088
H35	0,275396	-0,709005	2,410679	H35	0,304162	-0,834685	2,401377
C36	3,096714	-1,698859	4,862403	C36	3,222430	-1,807167	4,740292
H37	4,000595	-2,697652	3,190527	H37	4,109551	-2,722997	3,012661
C38	2,017347	-0,934197	5,312969	C38	2,134379	-1,089355	5,244731
H39	0,156362	0,007346	4,757301	H39	0,230522	-0,190280	4,769109
H40	3,900508	-1,964860	5,542748	H40	4,053200	-2,068959	5,389546
H41	1,976513	-0,606295	6,347806	H41	2,114510	-0,793096	6,289748
C42	-3,227567	-0,375815	-1,264093	C42	-3,294248	-0,407222	-1,132691
C43	-4,579077	-0,300979	-0,885696	C43	-4,637592	-0,359212	-0,724268
C44	-2,902639	-0,282369	-2,626649	C44	-3,002002	-0,307137	-2,501511
C45	-5,573284	-0,101766	-1,843873	C45	-5,657091	-0,185109	-1,659605
H46	-4,864270	-0,396829	0,156077	H46	-4,896745	-0,455904	0,324835
C47	-3,899580	-0,085494	-3,582593	C47	-4,024330	-0,141085	-3,435431
C48	-5,237705	0,012566	-3,194218	C48	-5,355083	-0,071617	-3,017851
H49	-6,612007	-0,037879	-1,532655	H49	-6,689294	-0,140942	-1,323859
H50	-3,630380	-0,005726	-4,631850	H50	-3,778913	-0,057252	-4,490425
H51	-6,013455	0,171711	-3,937445	H51	-6,150555	0,065958	-3,744228
H52	-0,178846	-0,932762	-1,773420	H52	-0,253707	-1,007853	-1,852492
H53	1,417830	1,999250	-2,474531	H53	1,625402	2,047717	-2,297429
H54	-1,708400	-1,999381	2,594531	H54	-1,683996	-1,907067	2,746089
H55	-1,867634	-0,358005	-2,940213	H55	-1,971226	-0,343954	-2,835865
H56	3,099690	-4,243234	0,537836	H56	3,098841	-4,196180	0,243070
C57	5,417760	-0,357543	-1,055047	C57	5,156417	-0,021172	-1,243785
C58	4,844092	-1,543879	-1,516524	C58	4,640071	-1,221891	-1,738922
C59	3,834855	-2,162917	-0,781265	C59	3,753152	-1,972233	-0,967909
C60	3,393807	-1,621641	0,434641	C60	3,368536	-1,542072	0,310287
C61	3,966722	-0,427242	0,884233	C61	3,889611	-0,337266	0,797647
C62	4,968039	0,202075	0,141254	C62	4,777490	0,415551	0,025862
H63	6,206043	0,125908	-1,625169	H63	5,850697	0,563039	-1,841451
H64	5,174593	-1,982062	-2,453705	H64	4,925614	-1,572297	-2,726731
H65	3,375076	-3,062201	-1,182077	H65	3,332514	-2,880566	-1,390911
H66	3,621842	0,025615	1,805281	H66	3,585446	0,030906	1,769956
H67	5,403709	1,125267	0,513258	H67	5,177826	1,342755	0,427841
C68	-0,338272	6,178271	-2,151870	C68	-0,140097	6,219147	-2,100726
C69	0,171865	5,024770	-1,559865	C69	0,269783	5,044523	-1,473087
C70	0,438801	3,886833	-2,331875	C70	0,626590	3,920436	-2,228875
C71	0,194030	3,930504	-3,707664	C71	0,575491	4,000906	-3,624296

C72	-0,322333	5,082803	-4,301545
C73	-0,591723	6,209518	-3,523887
H74	-0,549836	7,049373	-1,538059
H75	0,329234	4,994483	-0,486377
H76	0,399072	3,053353	-4,318272
H77	-0,512062	5,101426	-5,370994
H78	-0,995834	7,106847	-3,983804
C79	0,870885	2,564541	-1,709824
C80	-0,390125	1,708108	-1,328323
C81	-1,408711	2,547235	-0,578130
C82	-2,579322	2,950786	-1,242120
C83	-1,203460	3,036118	0,724127
C84	-3,489703	3,825094	-0,650110
H85	-2,770196	2,586363	-2,247004
C86	-2,098210	3,931876	1,311315
H87	-0,355370	2,695743	1,303006
C88	-3,241657	4,339313	0,623696
H89	-4,382814	4,120691	-1,193890
H90	-1,906032	4,299293	2,316058
H91	-3,938105	5,037612	1,079725
C92	3,006057	3,660722	-0,798582
C93	1,950171	2,118464	0,541939
C94	3,485129	3,790304	0,649468
H95	2,737245	4,617234	-1,247008
H96	3,734115	3,152568	-1,444263
H97	4,568776	3,784792	0,767737
H98	3,059328	4,665816	1,150360
N99	1,822127	2,809784	-0,608577
O100	2,955222	2,601043	1,294723
O101	1,300125	1,141541	0,955497
H102	2,416310	-4,267053	2,184523
N103	-1,932908	-2,488360	0,088996
C104	-0,909328	-3,373464	-0,503324
H105	-0,725668	-3,150942	-1,563692
H106	-0,822903	1,508685	-2,312001
H107	2,625373	0,300477	-1,375021
H108	2,220954	0,038109	-1,947079

TS_{Z3-4}

C1	-3,825379	-3,978950	-0,556223
C2	-1,576345	-4,798897	-0,085304
C3	-2,744638	-4,978256	-1,089017
C4	-3,128953	-3,382549	0,689612
H5	-4,748573	-4,493732	-0,270380
H6	-2,441784	-4,767119	-2,119629
H7	-3,781483	-2,856239	1,385886
H8	-4,085984	-3,202514	-1,277820
H9	-0,826793	-5,589871	-0,103490

C72	0,160622	5,174091	-4,255184
C73	-0,200388	6,286549	-3,493775
H74	-0,422835	7,079149	-1,499705
H75	0,280368	4,988785	-0,389596
H76	0,854391	3,135934	-4,223399
H77	0,122779	5,220915	-5,340168
H78	-0,525082	7,200919	-3,982182
C79	0,965065	2,576034	-1,596113
C80	-0,327551	1,699787	-1,427245
C81	-1,440174	2,517312	-0,793926
C82	-2,542237	2,888274	-1,583666
C83	-1,378260	3,046641	0,507188
C84	-3,527117	3,753223	-1,109837
H85	-2,620601	2,502516	-2,595805
C86	-2,340355	3,943435	0,973046
H87	-0,592788	2,741910	1,183003
C88	-3,421465	4,303193	0,169464
H89	-4,366036	4,014228	-1,749664
H90	-2,253981	4,340675	1,981251
H91	-4,174299	4,996558	0,534567
C92	2,953821	3,607009	-0,345477
C93	1,689001	2,052610	0,790714
C94	3,208088	3,704574	1,160966
H95	2,766177	4,575036	-0,810113
H96	3,766242	3,100081	-0,882699
H97	4,261043	3,685333	1,442886
H98	2,719879	4,576847	1,607869
N99	1,746854	2,768046	-0,354989
O100	2,577639	2,510518	1,694860
O101	0,964152	1,087891	1,086083
H102	2,615897	-4,303519	1,957208
N103	-1,945661	-2,478546	0,233283
C104	-0,972728	-3,388514	-0,411890
H105	-0,861804	-3,188748	-1,487319
H106	-0,627785	1,522449	-2,464089
H107	1,939844	0,093834	-0,980810
H108	1,579833	-0,115056	-1,684124

Z4

C1	-3,724188	-4,145535	-0,601233
C2	-1,431953	-4,908094	-0,254999
C3	-2,624689	-5,062497	-1,234931
C4	-3,018518	-3,624586	0,673326
H5	-4,623784	-4,712282	-0,340194
H6	-2,363736	-4,769768	-2,257076
H7	-3,675693	-3,181735	1,421898
H8	-4,025449	-3,322328	-1,252660
H9	-0,651200	-5,662247	-0,355034

H10	-3,104497	-6,010701	-1,059569	H10	-2,945925	-6,107585	-1,266573
C11	-2,339022	-4,582128	1,239238	C11	-2,162442	-4,828263	1,104139
H12	-1,697124	-4,324657	2,086894	H12	-1,504510	-4,606817	1,949678
H13	-2,972779	-5,433253	1,504184	H13	-2,750512	-5,725124	1,319190
C14	0,368465	-3,124441	0,292926	C14	0,455248	-3,163681	0,156530
N15	0,940273	-1,967862	0,366923	N15	0,995364	-1,988681	0,220941
C16	2,224506	-2,122128	1,152780	C16	2,303748	-2,096125	0,978172
C17	2,351690	-3,680109	1,176471	C17	2,491733	-3,646664	0,958792
P18	-2,029434	-0,819072	0,075898	P18	-2,006428	-0,987823	0,159494
Ir19	0,234097	-0,237977	-0,717338	Ir19	0,176343	-0,294797	-0,592589
C20	-2,704035	-0,284353	1,693296	C20	-2,580001	-0,432357	1,804169
C21	-3,611371	0,585711	4,204250	C21	-3,269108	0,564140	4,339074
C22	-2,417799	-1,024897	2,851873	C22	-2,444862	-1,237277	2,944719
C23	-3,447714	0,898084	1,810267	C23	-3,050131	0,881667	1,949782
C24	-3,896438	1,328246	3,058224	C24	-3,391119	1,374245	3,207159
C25	-2,870377	-0,592937	4,097316	C25	-2,796029	-0,742295	4,202257
H26	-3,681044	1,485676	0,930558	H26	-3,158460	1,520852	1,082310
H27	-4,469102	2,248508	3,129380	H27	-3,752669	2,394561	3,297321
H28	-2,644592	-1,180420	4,982800	H28	-2,692055	-1,380042	5,075851
H29	-3,966761	0,920605	5,174686	H29	-3,540048	0,947236	5,318632
O30	1,027842	-4,160221	0,804419	O30	1,170030	-4,174026	0,646572
C31	2,052798	-1,611318	2,586572	C31	2,134933	-1,627453	2,426105
C32	0,919375	-0,925262	3,018126	C32	0,958853	-1,060130	2,911785
C33	3,071951	-1,877513	3,514847	C33	3,205847	-1,825326	3,314232
C34	0,801191	-0,508870	4,345291	C34	0,854210	-0,678978	4,251352
H35	0,128679	-0,692624	2,322030	H35	0,119707	-0,893153	2,255043
C36	2,959711	-1,459072	4,838030	C36	3,103441	-1,446772	4,649307
H37	3,971421	-2,399270	3,195803	H37	4,133812	-2,262880	2,953144
C38	1,819162	-0,771136	5,259057	C38	1,923176	-0,866953	5,122816
H39	-0,095297	0,020811	4,654131	H39	-0,072961	-0,234544	4,600040
H40	3,762356	-1,669459	5,538986	H40	3,944292	-1,603281	5,319026
H41	1,728416	-0,445221	6,291344	H41	1,841782	-0,568693	6,164165
C42	-3,385106	-0,460426	-1,120661	C42	-3,396488	-0,592776	-0,978443
C43	-4,736926	-0,485769	-0,738734	C43	-4,716497	-0,401762	-0,545497
C44	-3,075320	-0,278911	-2,477171	C44	-3,119902	-0,563499	-2,355554
C45	-5,746566	-0,310151	-1,684454	C45	-5,734605	-0,167900	-1,469867
H46	-5,007744	-0,637126	0,301230	H46	-4,951345	-0,424557	0,513704
C47	-4,086245	-0,107751	-3,423553	C47	-4,140777	-0,338903	-3,278760
C48	-5,425625	-0,116949	-3,029855	C48	-5,449801	-0,134676	-2,836335
H49	-6,785848	-0,327174	-1,368527	H49	-6,752623	-0,015893	-1,122323
H50	-3,826509	0,035619	-4,468699	H50	-3,915097	-0,319656	-4,341367
H51	-6,213194	0,020297	-3,764815	H51	-6,245267	0,045668	-3,553756
H52	-0,210499	-1,255857	-1,826913	H52	0,024073	-1,144867	-1,892784
H53	1,555422	2,119155	-2,491652	H53	1,525102	2,075720	-2,241352
H54	-1,840819	-1,940154	2,779619	H54	-2,044189	-2,241678	2,856304
H55	-2,038607	-0,271529	-2,795138	H55	-2,101054	-0,713552	-2,701930
H56	3,059101	-4,070821	0,441499	H56	3,173124	-3,991654	0,177702
C57	5,423022	-0,256283	-1,114905	C57	5,396341	-0,049999	-1,280131

C58	4,690083	-1,317715	-1,652307	C58	4,692204	-1,119556	-1,838605
C59	3,679599	-1,914083	-0,903705	C59	3,713598	-1,771950	-1,095099
C60	3,382766	-1,469006	0,393169	C60	3,416362	-1,372721	0,216041
C61	4,115293	-0,403166	0,919624	C61	4,109796	-0,288863	0,760279
C62	5,129488	0,197410	0,169748	C62	5,099276	0,361251	0,018187
H63	6,217297	0,207245	-1,693505	H63	6,170940	0,453099	-1,852148
H64	4,902721	-1,676915	-2,655068	H64	4,904792	-1,443622	-2,853274
H65	3,092411	-2,711368	-1,351616	H65	3,149595	-2,576766	-1,558513
H66	3,884092	-0,017208	1,903865	H66	3,874899	0,061389	1,757652
H67	5,695080	1,019084	0,601448	H67	5,650083	1,184497	0,466205
C68	-0,154047	6,292520	-1,925787	C68	0,564137	6,521623	-2,256494
C69	0,293322	5,082247	-1,399706	C69	0,665451	5,306467	-1,579982
C70	0,592160	4,009622	-2,248211	C70	0,902002	4,121891	-2,286465
C71	0,443625	4,171401	-3,629110	C71	1,041090	4,175192	-3,678027
C72	-0,010282	5,380510	-4,156680	C72	0,932811	5,388999	-4,356417
C73	-0,311338	6,443592	-3,304867	C73	0,692384	6,565482	-3,645707
H74	-0,389179	7,115755	-1,257266	H74	0,379615	7,434674	-1,697721
H75	0,384427	4,958186	-0,325385	H75	0,557109	5,278825	-0,500936
H76	0,680619	3,345609	-4,297449	H76	1,237158	3,261106	-4,235424
H77	-0,123623	5,493128	-5,231051	H77	1,042281	5,416194	-5,436682
H78	-0,664460	7,386026	-3,713399	H78	0,609390	7,512461	-4,171029
C79	0,973034	2,633749	-1,715121	C79	0,938567	2,753291	-1,609507
C80	-0,324363	1,790153	-1,491813	C80	-0,500876	2,188391	-1,621974
C81	-1,376192	2,505756	-0,653054	C81	-1,623274	2,876605	-0,877962
C82	-2,535834	2,947807	-1,312657	C82	-2,905501	2,807284	-1,446959
C83	-1,200065	2,901029	0,683563	C83	-1,467679	3,592655	0,316400
C84	-3,458783	3,783652	-0,684941	C84	-3,999342	3,421390	-0,840359
H85	-2,709510	2,648571	-2,342312	H85	-3,053510	2,253419	-2,369557
C86	-2,106987	3,761109	1,303069	C86	-2,557452	4,229488	0,912564
H87	-0,370707	2,522211	1,260981	H87	-0,500497	3,666445	0,791389
C88	-3,237602	4,215998	0,623966	C88	-3,829085	4,142116	0,343774
H89	-4,340851	4,110186	-1,228383	H89	-4,980951	3,336620	-1,296630
H90	-1,935223	4,057254	2,334134	H90	-2,409163	4,788470	1,832243
H91	-3,940728	4,886962	1,108887	H91	-4,675937	4,633481	0,813899
C92	3,095934	3,541071	-0,638378	C92	3,010360	3,358518	-0,302808
C93	1,900929	1,977082	0,553064	C93	1,445737	2,065438	0,790836
C94	3,520453	3,549604	0,833087	C94	3,298930	3,327357	1,199419
H95	2,905675	4,539019	-1,034557	H95	3,057612	4,362133	-0,726572
H96	3,818289	3,024534	-1,283904	H96	3,669594	2,688389	-0,870052
H97	4,595278	3,454299	0,988146	H97	4,321216	3,043962	1,450322
H98	3,140975	4,424625	1,370450	H98	3,041025	4,269817	1,693501
N99	1,855376	2,758419	-0,547637	N99	1,636308	2,834913	-0,315855
O100	2,881273	2,369690	1,385141	O100	2,410085	2,297530	1,700432
O101	1,171547	1,028199	0,875838	O101	0,523274	1,282289	1,044818
H102	2,581328	-4,076257	2,163749	H102	2,791400	-4,051417	1,923361
N103	-2,002558	-2,508662	0,260001	N103	-1,938419	-2,675849	0,295397
C104	-0,977166	-3,389805	-0,337436	C104	-0,905285	-3,459454	-0,422409
H105	-0,850098	-3,221421	-1,416639	H105	-0,846076	-3,196765	-1,488015

H106	-0,761489	1,776629	-2,495548	H106	-0,756238	2,197980	-2,687412
H107	1,679775	-0,119202	-1,392547	H107	1,628191	0,057484	-1,189735
H108	0,236253	0,431305	-2,148581	H108	-0,616550	1,043242	-1,516089

Isomerization

Iso1				TS_{Iso1-2}			
C1	-4,163957	-3,413621	0,495949	C1	4,346353	3,217037	0,263892
C2	-2,022982	-4,485459	0,026619	C2	2,259239	4,384293	-0,227178
C3	-3,482403	-4,418747	-0,490090	C3	3,701615	4,202789	-0,765849
C4	-2,991128	-3,026092	1,427059	C4	3,176618	2,961192	1,243615
H5	-4,961675	-3,891034	1,074636	H5	5,189183	3,675193	0,791983
H6	-3,536093	-4,089495	-1,532711	H6	3,714378	3,813073	-1,788864
H7	-3,267742	-2,520272	2,352589	H7	3,447576	2,499120	2,193547
H8	-4,592510	-2,548004	-0,008998	H8	4,704699	2,294690	-0,194802
H9	-1,449996	-5,342382	-0,325984	H9	1,725944	5,248340	-0,622460
H10	-3,942675	-5,409269	-0,436314	H10	4,218791	5,166392	-0,777937
C11	-2,224355	-4,353581	1,552521	C11	2,489743	4,336607	1,299949
H12	-1,293876	-4,260150	2,120793	H12	1,569152	4,334075	1,891728
H13	-2,829643	-5,162804	1,970202	H13	3,149606	5,133868	1,653067
C14	0,152012	-3,110362	-0,165796	C14	0,007517	3,108169	-0,317945
N15	0,893885	-2,057120	-0,123457	N15	-0,776244	2,091120	-0,209954
C16	2,348877	-2,501544	-0,197728	C16	-2,208721	2,585513	-0,307392
C17	2,186916	-4,028465	0,065158	C17	-1,986872	4,122475	-0,162750
P18	-1,924278	-0,543643	0,464644	P18	1,940575	0,495614	0,482512
Ir19	0,274502	-0,008131	-0,426442	Ir19	-0,226986	-0,002426	-0,404242
C20	-2,161935	0,087161	2,164131	C20	2,094370	-0,042618	2,218503
C21	-2,171526	1,163910	4,755967	C21	1,929672	-0,988937	4,852181
C22	-1,463363	-0,548212	3,203504	C22	1,506843	0,743900	3,220487
C23	-2,861686	1,270604	2,439483	C23	2,591663	-1,312005	2,548385
C24	-2,865622	1,804291	3,729439	C24	2,509920	-1,780063	3,860198
C25	-1,474395	-0,016881	4,490101	C25	1,432581	0,275533	4,529763
H26	-3,416156	1,771534	1,654956	H26	3,047757	-1,933487	1,786119
H27	-3,421403	2,715740	3,931945	H27	2,908258	-2,760313	4,107698
H28	-0,930393	-0,518863	5,284880	H28	0,978728	0,894090	5,298525
H29	-2,174407	1,580659	5,758915	H29	1,865129	-1,355148	5,872519
O30	0,771150	-4,294209	-0,144853	O30	-0,561993	4,315914	-0,381884
C31	3,198321	-1,908942	0,919348	C31	-3,068113	2,107681	0,855317
C32	2,602482	-1,431515	2,089597	C32	-2,482060	1,634149	2,031931
C33	4,598267	-1,955256	0,842561	C33	-4,462657	2,248537	0,800891
C34	3,384829	-0,936438	3,132724	C34	-3,275588	1,242064	3,110371
H35	1,524467	-1,410267	2,176880	H35	-1,406851	1,537510	2,100836
C36	5,380638	-1,474994	1,891534	C36	-5,254385	1,872044	1,884198
H37	5,081068	-2,354774	-0,043770	H37	-4,933773	2,640566	-0,095448
C38	4,775849	-0,949494	3,034920	C38	-4,663589	1,353918	3,038720
H39	2,899988	-0,532960	4,016736	H39	-2,801210	0,841484	4,001305
H40	6,463394	-1,507060	1,811104	H40	-6,333562	1,978923	1,822859
H41	5,385885	-0,561588	3,845728	H41	-5,282228	1,048285	3,877653
C42	-3,402865	-0,059708	-0,500188	C42	3,400544	-0,148548	-0,410044

C43	-4,682540	0,032883	0,074487	C43	4,637022	-0,363737	0,220583
C44	-3,266370	0,099402	-1,886597	C44	3,305935	-0,287794	-1,802633
C45	-5,794990	0,298304	-0,722171	C45	5,753766	-0,723571	-0,531561
H46	-4,814556	-0,107826	1,142984	H46	4,733825	-0,242250	1,294890
C47	-4,383129	0,357195	-2,681144	C47	4,427149	-0,640176	-2,551205
C48	-5,648418	0,461097	-2,102500	C48	5,650327	-0,861592	-1,917425
H49	-6,777827	0,372730	-0,265463	H49	6,706050	-0,890578	-0,036308
H50	-4,259303	0,494953	-3,750946	H50	4,340139	-0,759443	-3,626752
H51	-6,516448	0,669770	-2,720887	H51	6,522661	-1,141944	-2,500838
H52	-0,118809	-0,428638	-1,876377	H52	0,213168	0,348314	-1,859339
H53	1,756460	-0,020859	-0,994801	H53	-1,709866	-0,069468	-1,031224
H54	-0,902186	-1,453808	2,994702	H54	1,095098	1,715715	2,965622
H55	-2,281344	0,048333	-2,337410	H55	2,348110	-0,150459	-2,292681
H56	2,761357	-4,656663	-0,615218	H56	-2,534652	4,718544	-0,892680
C57	3,441736	-1,519157	-4,288053	C57	-3,380542	1,313445	-4,294481
C58	2,656782	-2,639437	-4,007968	C58	-2,534468	2,409817	-4,114400
C59	2,351987	-2,968942	-2,688848	C59	-2,204142	2,835734	-2,829575
C60	2,828100	-2,191625	-1,623160	C60	-2,714522	2,181545	-1,699572
C61	3,612994	-1,068426	-1,918028	C61	-3,563190	1,083499	-1,893208
C62	3,918381	-0,736228	-3,238228	C62	-3,893035	0,652750	-3,179390
H63	3,679810	-1,262062	-5,316241	H63	-3,638810	0,981313	-5,295981
H64	2,279453	-3,257252	-4,817746	H64	-2,129848	2,933481	-4,975774
H65	1,718127	-3,831351	-2,499850	H65	-1,524674	3,676047	-2,716567
H66	3,986116	-0,436856	-1,122065	H66	-3,969731	0,549772	-1,043602
H67	4,527618	0,140556	-3,439156	H67	-4,550803	-0,203286	-3,301388
C68	-2,454159	3,304004	-3,397543	C68	2,183042	-3,383957	-3,440746
C69	-1,344000	2,761373	-2,754102	C69	1,118782	-2,796816	-2,759807
C70	-1,296252	2,673925	-1,354775	C70	1,101550	-2,745230	-1,357878
C71	-2,394445	3,157909	-0,627800	C71	2,189606	-3,303290	-0,668332
C72	-3,505325	3,699669	-1,269321	C72	3,255000	-3,889432	-1,347517
C73	-3,541384	3,775807	-2,660551	C73	3,257004	-3,935284	-2,740328
H74	-2,464845	3,366183	-4,482193	H74	2,169500	-3,414206	-4,526705
H75	-0,513264	2,410466	-3,357111	H75	0,302662	-2,375385	-3,337364
H76	-2,372119	3,105654	0,454095	H76	2,202863	-3,265504	0,415075
H77	-4,343115	4,061073	-0,679835	H77	4,086985	-4,303722	-0,785321
H78	-4,403228	4,201513	-3,165751	H78	4,084166	-4,393350	-3,274316
C79	-0,106033	2,133807	-0,626018	C79	-0,029502	-2,139255	-0,585748
C80	1,125307	1,792608	-1,303040	C80	-1,278532	-1,740727	-1,230887
H81	1,048777	1,679271	-2,379219	H81	-1,234841	-1,675966	-2,313437
C82	2,448229	2,373226	-0,893344	C82	-2,608088	-2,259811	-0,762282
C83	3,259374	1,896668	0,143166	C83	-3,366989	-1,707009	0,274847
C84	2,868132	3,500193	-1,619064	C84	-3,090482	-3,398122	-1,427581
C85	4,457768	2,542121	0,454324	C85	-4,579246	-2,290845	0,647646
H86	2,964816	1,016009	0,701672	H86	-3,018353	-0,817984	0,787117
C87	4,063310	4,144893	-1,304865	C87	-4,299106	-3,981243	-1,051220
H88	2,250148	3,873636	-2,432802	H88	-2,512526	-3,827686	-2,243129
C89	4,862027	3,667689	-0,263125	C89	-5,046992	-3,429131	-0,008597
H90	5,075384	2,151381	1,257963	H90	-5,156448	-1,843327	1,451478

H91	4,372375	5,015353	-1,876865	H91	-4,658645	-4,861684	-1,576393
H92	5,795992	4,166521	-0,020807	H92	-5,991259	-3,880471	0,281643
C93	0,254094	4,151686	0,961488	C93	-0,441600	-4,136937	1,023317
C94	0,565770	1,991957	1,718085	C94	-0,644979	-1,957904	1,764513
C95	0,397076	4,111983	2,492557	C95	-0,572213	-4,076367	2,555191
H96	-0,600219	4,743908	0,629460	H96	0,381277	-4,771882	0,690919
H97	1,160255	4,505547	0,456694	H97	-1,366196	-4,460016	0,531103
H98	1,141533	4,803136	2,887851	H98	-1,344412	-4,730195	2,960863
H99	-0,561121	4,257198	3,001336	H99	0,380953	-4,266026	3,059316
N100	0,050990	2,719689	0,698213	N100	-0,181224	-2,718297	0,742361
O101	0,845583	2,755845	2,779472	O101	-0,953012	-2,700218	2,833473
O102	0,775321	0,772262	1,698901	O102	-0,798476	-0,729771	1,735516
H103	2,416940	-4,286348	1,101050	H103	-2,213139	4,468237	0,848174
N104	-1,976927	-2,225396	0,683726	N104	2,103820	2,178035	0,571835
C105	-1,350348	-3,128567	-0,306966	C105	1,508408	3,047827	-0,464122
H106	-1,565209	-2,828735	-1,344085	H106	1,696789	2,674951	-1,483108

Iso2				TS_{Iso2-3}			
C1	4,843561	2,498820	-0,482967	C1	-4,338247	2,694784	-1,634939
C2	2,981888	3,839251	-1,315714	C2	-4,312143	0,407061	-2,480902
C3	4,382473	3,321595	-1,731988	C3	-4,451374	1,874037	-2,962482
C4	3,642901	2,666109	0,476882	C4	-4,170576	1,580282	-0,575436
H5	5,750371	2,916876	-0,033693	H5	-5,246363	3,273413	-1,436174
H6	4,346168	2,720039	-2,645717	H6	-3,682692	2,143279	-3,693827
H7	3,829001	2,398250	1,517273	H7	-4,339121	1,887039	0,457034
H8	5,037176	1,448989	-0,709024	H8	-3,495340	3,388306	-1,629491
H9	2,595418	4,660659	-1,918503	H9	-4,629488	-0,349141	-3,199008
H10	5,049894	4,166554	-1,923308	H10	-5,423860	2,018465	-3,441791
C11	3,185366	4,106635	0,191923	C11	-5,059647	0,454014	-1,129735
H12	2,270165	4,379259	0,726734	H12	-4,993582	-0,470937	-0,548078
H13	3,960976	4,852394	0,387020	H13	-6,108235	0,745263	-1,238163
C14	0,555778	2,940029	-1,236661	C14	-2,381232	-1,207984	-1,850879
N15	-0,358347	2,145518	-0,806548	N15	-1,396209	-1,553909	-1,104642
C16	-1,690595	2,816668	-0,959944	C16	-1,238082	-3,038369	-1,168230
C17	-1,289397	4,113461	-1,748727	C17	-2,186359	-3,381344	-2,366780
P18	1,991962	0,342789	0,307287	P18	-1,414846	1,349289	0,095074
Ir19	-0,073215	-0,003165	-0,435216	Ir19	0,106655	-0,202197	-0,291685
C20	2,018273	0,280332	2,128194	C20	-1,889612	1,357047	1,852694
C21	1,641370	0,120230	4,900222	C21	-2,380294	1,086581	4,597613
C22	1,608052	1,408195	2,854719	C22	-2,881016	0,477863	2,311952
C23	2,239199	-0,929057	2,804339	C23	-1,142188	2,098289	2,782086
C24	2,052507	-1,005522	4,184384	C24	-1,391777	1,964028	4,147019
C25	1,426065	1,327932	4,233494	C25	-3,126111	0,348046	3,677748
H26	2,568168	-1,805532	2,256557	H26	-0,369203	2,777857	2,439124
H27	2,234305	-1,943571	4,701675	H27	-0,819442	2,550926	4,860545
H28	1,109658	2,207252	4,787112	H28	-3,898394	-0,332829	4,024012
H29	1,491790	0,057316	5,974100	H29	-2,570066	0,981790	5,661885
O30	0,167030	4,115478	-1,737839	O30	-2,980892	-2,171094	-2,556556

C31	-2,278706	3,197707	0,396966	C31	-1,758951	-3,693655	0,112502
C32	-1,658360	2,861172	1,599704	C32	-2,139546	-2,948186	1,228139
C33	-3,466153	3,945586	0,429776	C33	-1,858315	-5,092088	0,161802
C34	-2,227680	3,239694	2,817435	C34	-2,611304	-3,586560	2,376801
H35	-0,746941	2,282426	1,593911	H35	-2,044052	-1,872766	1,215750
C36	-4,031252	4,327533	1,643615	C36	-2,331096	-5,728997	1,306237
H37	-3,960194	4,215570	-0,500960	H37	-1,544776	-5,687768	-0,693003
C38	-3,413882	3,969676	2,845044	C38	-2,710158	-4,975562	2,420491
H39	-1,739223	2,951131	3,744044	H39	-2,890739	-2,988564	3,240044
H40	-4,954625	4,899653	1,651929	H40	-2,400185	-6,812753	1,330297
H41	-3,857450	4,261052	3,792803	H41	-3,076130	-5,472077	3,314604
C42	3,311368	-0,776198	-0,282387	C42	-1,043174	3,074582	-0,369582
C43	4,489556	-0,947278	0,465789	C43	-1,601144	4,154602	0,334706
C44	3,213191	-1,370297	-1,548818	C44	-0,346045	3,316340	-1,562379
C45	5,541302	-1,707847	-0,042925	C45	-1,450888	5,454781	-0,145483
H46	4,589032	-0,488319	1,444955	H46	-2,162357	3,988169	1,248218
C47	4,270648	-2,125073	-2,054765	C47	-0,204075	4,616890	-2,038597
C48	5,434979	-2,298252	-1,304711	C48	-0,755164	5,687715	-1,332579
H49	6,444348	-1,837384	0,546767	H49	-1,883394	6,284576	0,406479
H50	4,173041	-2,595672	-3,028103	H50	0,353092	4,795075	-2,953047
H51	6,254528	-2,892943	-1,697183	H51	-0,639272	6,701928	-1,704031
H52	0,512792	-0,095084	-1,888116	H52	0,264459	0,385997	-1,737970
H53	-1,908184	-0,614304	-1,123946	H53	2,453381	-1,490840	0,680694
H54	1,421649	2,343264	2,336171	H54	-3,450937	-0,110971	1,600720
H55	2,297886	-1,271168	-2,120767	H55	0,111077	2,491977	-2,097377
H56	-1,613290	4,105252	-2,791941	H56	-1,658086	-3,582087	-3,302155
C57	-4,049059	0,075552	-3,372072	C57	2,992897	-3,673457	-1,945403
C58	-2,848751	0,607543	-3,850578	C58	2,176119	-3,067224	-2,902874
C59	-2,124452	1,504140	-3,070110	C59	0,817147	-2,890511	-2,650107
C60	-2,581430	1,889875	-1,798802	C60	0,245390	-3,325960	-1,442849
C61	-3,779899	1,345487	-1,327514	C61	1,076485	-3,917714	-0,483601
C62	-4,509387	0,447392	-2,111173	C62	2,438960	-4,092721	-0,736746
H63	-4,614752	-0,628902	-3,974851	H63	4,054442	-3,800782	-2,134572
H64	-2,475100	0,320525	-4,829484	H64	2,598442	-2,725899	-3,843795
H65	-1,176175	1,882766	-3,442187	H65	0,203778	-2,386686	-3,392295
H66	-4,142737	1,605442	-0,340817	H66	0,662461	-4,237491	0,465926
H67	-5,428105	0,022187	-1,719355	H67	3,069105	-4,552585	0,019190
C68	1,381926	-4,589811	-2,459505	C68	3,252546	3,348508	-1,799379
C69	0,469924	-3,614166	-2,053382	C69	2,858619	2,099851	-1,326414
C70	0,486154	-3,110351	-0,745354	C70	2,338287	1,939718	-0,033293
C71	1,460406	-3,617419	0,133444	C71	2,172664	3,099609	0,741889
C72	2,367854	-4,592608	-0,266440	C72	2,556656	4,353692	0,269307
C73	2,331801	-5,089302	-1,569700	C73	3,117462	4,484516	-0,999096
H74	1,340046	-4,967045	-3,477565	H74	3,663822	3,434227	-2,801634
H75	-0,255023	-3,255223	-2,776100	H75	2,934463	1,242648	-1,980858
H76	1,506444	-3,226956	1,144191	H76	1,711460	3,031246	1,720527
H77	3,109519	-4,957749	0,438523	H77	2,407258	5,229278	0,895075
H78	3,034275	-5,854302	-1,886850	H78	3,427578	5,458307	-1,366766

C79	-0,455128	-2,050446	-0,249052	C79	1,900289	0,594367	0,463107
C80	-1,724319	-1,738381	-1,097761	C80	2,677043	-0,691837	-0,032987
H81	-1,551770	-1,976878	-2,144944	H81	2,236952	-1,030630	-0,992783
C82	-3,023016	-2,335138	-0,614414	C82	4,182318	-0,672268	-0,244258
C83	-3,793648	-1,707996	0,371142	C83	5,051211	-0,878796	0,834740
C84	-3,430210	-3,576776	-1,115837	C84	4,740418	-0,529958	-1,520965
C85	-4,947932	-2,321437	0,859073	C85	6,434129	-0,885105	0,656417
H86	-3,491226	-0,735734	0,750028	H86	4,643803	-1,076527	1,822409
C87	-4,587886	-4,187742	-0,632244	C87	6,122422	-0,536553	-1,707448
H88	-2,831370	-4,071392	-1,876943	H88	4,087903	-0,435125	-2,384391
C89	-5,347332	-3,563095	0,359302	C89	6,975628	-0,704329	-0,616525
H90	-5,538025	-1,827586	1,626036	H90	7,087177	-1,047014	1,509564
H91	-4,895104	-5,151805	-1,027957	H91	6,531464	-0,418820	-2,707065
H92	-6,247557	-4,039725	0,736492	H92	8,052357	-0,709386	-0,759101
C93	-1,250301	-3,555380	1,783132	C93	2,821267	0,923180	2,940787
C94	-1,114377	-1,255243	1,953235	C94	0,961978	-0,352135	2,470866
C95	-1,457783	-3,084619	3,235876	C95	2,104312	0,481807	4,228532
H96	-0,497522	-4,340545	1,699009	H96	3,053016	1,987921	2,930821
H97	-2,176498	-3,896790	1,314752	H97	3,750056	0,375376	2,765908
H98	-2,367533	-3,470552	3,696385	H98	2,759294	0,017511	4,966488
H99	-0,595346	-3,301705	3,873754	H99	1,545903	1,300918	4,692902
N100	-0,787489	-2,300227	1,168516	N100	1,798829	0,566746	1,941505
O101	-1,580779	-1,635455	3,146055	O101	1,139593	-0,512747	3,786849
O102	-1,023991	-0,058569	1,623522	O102	0,128047	-1,013839	1,825621
H103	-1,626603	5,026165	-1,259088	H103	-2,872777	-4,197104	-2,146128
N104	2,462291	1,926124	-0,051385	N104	-2,836648	0,931216	-0,722518
C105	2,028368	2,615268	-1,285238	C105	-2,843037	0,216407	-2,018754
H106	2,171109	1,994454	-2,183379	H106	-2,153691	0,670207	-2,748212

Iso3				TS_{Iso3-4}			
C1	0,752502	-4,421648	-2,686798	C1	-2,811440	-3,038130	-3,146331
C2	2,233990	-2,621454	-3,403070	C2	-0,422953	-2,999946	-3,658226
C3	1,197550	-3,629712	-3,961557	C3	-1,805447	-2,708276	-4,299074
C4	1,624601	-3,779605	-1,582144	C4	-1,862659	-3,459388	-2,000405
H5	0,975938	-5,489776	-2,773877	H5	-3,473487	-3,868878	-3,411446
H6	0,362918	-3,130510	-4,463699	H6	-1,888101	-1,674101	-4,648384
H7	1,692563	-4,347080	-0,653879	H7	-2,337359	-3,973386	-1,163952
H8	-0,312051	-4,321303	-2,467938	H8	-3,437549	-2,189735	-2,866396
H9	2,868743	-2,146360	-4,150831	H9	0,398516	-3,118128	-4,364443
H10	1,674306	-4,286817	-4,694343	H10	-1,959646	-3,358154	-5,164907
C11	2,938163	-3,472267	-2,322073	C11	-0,749461	-4,209618	-2,753001
H12	3,654048	-2,914746	-1,710427	H12	0,078246	-4,516938	-2,106781
H13	3,420670	-4,361940	-2,736115	H13	-1,114975	-5,072785	-3,315899
C14	2,181002	-0,324762	-2,190834	C14	1,238004	-1,797616	-2,070486
N15	1,821642	0,469845	-1,246896	N15	1,583019	-1,104094	-1,041237
C16	2,856950	1,553201	-1,125031	C16	3,069013	-1,264845	-0,826640
C17	3,621835	1,367451	-2,472698	C17	3,419875	-2,311481	-1,941339
P18	0,044718	-1,886140	-0,156067	P18	-1,488143	-1,209507	-0,280571

Ir19	-0,074724	0,341953	-0,157424	Ir19	0,309495	0,332786	-0,010396
C20	0,682992	-2,523155	1,434225	C20	-1,698765	-2,253244	1,199683
C21	1,639218	-3,226468	3,978610	C21	-1,741791	-3,673678	3,616442
C22	2,039424	-2,816486	1,624770	C22	-1,053890	-3,492444	1,303526
C23	-0,192584	-2,592966	2,529981	C23	-2,370524	-1,733691	2,317915
C24	0,284490	-2,939047	3,792915	C24	-2,389612	-2,440332	3,518308
C25	2,510866	-3,172251	2,890131	C25	-1,079542	-4,199287	2,506598
H26	-1,248236	-2,389593	2,388334	H26	-2,875000	-0,775287	2,248834
H27	-0,403611	-2,995231	4,631866	H27	-2,913151	-2,031021	4,377922
H28	3,562927	-3,407277	3,023701	H28	-0,578278	-5,160402	2,575778
H29	2,009899	-3,499342	4,962152	H29	-1,755886	-4,222692	4,553195
O30	3,255391	0,021584	-2,906000	O30	2,190533	-2,487053	-2,699188
C31	3,816903	1,266225	0,035096	C31	3,384238	-1,902589	0,523235
C32	3,659372	0,175601	0,886411	C32	2,396300	-2,534812	1,277849
C33	4,904755	2,130254	0,232025	C33	4,718005	-1,975879	0,952909
C34	4,559130	-0,044223	1,931082	C34	2,722366	-3,200069	2,459501
H35	2,816625	-0,484733	0,759355	H35	1,363858	-2,488442	0,963637
C36	5,806121	1,909509	1,269906	C36	5,045839	-2,648684	2,128015
H37	5,031990	2,994164	-0,416312	H37	5,501197	-1,496122	0,372534
C38	5,632652	0,819755	2,127614	C38	4,046318	-3,259604	2,889181
H39	4,402513	-0,887934	2,596876	H39	1,930472	-3,661023	3,042925
H40	6,638287	2,591598	1,415486	H40	6,081928	-2,691728	2,451024
H41	6,329608	0,652230	2,943749	H41	4,302200	-3,776494	3,809525
C42	-1,497643	-2,815638	-0,457087	C42	-3,148968	-0,577093	-0,700174
C43	-1,671097	-4,107415	0,066948	C43	-4,329557	-1,188110	-0,253027
C44	-2,452213	-2,299453	-1,344980	C44	-3,219881	0,447134	-1,656942
C45	-2,784695	-4,864564	-0,290885	C45	-5,563565	-0,774846	-0,755278
H46	-0,939110	-4,526214	0,750063	H46	-4,290618	-1,992333	0,474240
C47	-3,564097	-3,061113	-1,697823	C47	-4,453222	0,849289	-2,161579
C48	-3,732155	-4,343415	-1,173619	C48	-5,626629	0,240377	-1,710553
H49	-2,911220	-5,862219	0,119389	H49	-6,474366	-1,252133	-0,405164
H50	-4,307574	-2,644960	-2,370480	H50	-4,500632	1,650091	-2,892728
H51	-4,601242	-4,934202	-1,448395	H51	-6,588719	0,558342	-2,101597
H52	-0,831413	0,233738	-1,530023	H52	-0,012877	0,952030	-1,406386
H53	-1,147446	2,917124	1,330328	H53	1,347700	2,264984	1,677842
H54	2,729667	-2,758143	0,790274	H54	-0,519949	-3,896714	0,449707
H55	-2,337525	-1,295229	-1,735696	H55	-2,310870	0,938235	-1,988788
H56	3,315367	2,061206	-3,258926	H56	4,204264	-1,988179	-2,625661
C57	0,589788	5,230008	-0,684315	C57	4,790170	2,664396	-1,511134
C58	0,661250	4,590010	-1,922130	C58	4,415431	1,858109	-2,586533
C59	1,416480	3,427944	-2,066560	C59	3,881329	0,590448	-2,358162
C60	2,129181	2,892781	-0,983609	C60	3,726878	0,099275	-1,054940
C61	2,041888	3,536775	0,257661	C61	4,085367	0,926790	0,018399
C62	1,279490	4,697307	0,403694	C62	4,616218	2,197451	-0,209049
H63	-0,009204	6,128293	-0,568648	H63	5,200456	3,654195	-1,686624
H64	0,108745	4,982971	-2,769577	H64	4,533613	2,217026	-3,604795
H65	1,419355	2,922980	-3,027940	H65	3,572990	-0,008711	-3,211000
H66	2,553748	3,121326	1,117332	H66	3,951473	0,575039	1,035530

H67	1,225812	5,183365	1,373764	H67	4,889726	2,823672	0,635193
C68	-5,187056	-0,203434	-0,391292	C68	-3,273770	3,931915	-0,739652
C69	-3,969106	0,464308	-0,270602	C69	-2,050629	3,369276	-0,379862
C70	-3,097531	0,197801	0,797671	C70	-1,964398	2,458589	0,685894
C71	-3,491735	-0,799949	1,709194	C71	-3,158808	2,104656	1,338148
C72	-4,699831	-1,480005	1,580278	C72	-4,382114	2,658216	0,969973
C73	-5,565984	-1,176555	0,531856	C73	-4,445391	3,589508	-0,064515
H74	-5,840730	0,039617	-1,224591	H74	-3,307485	4,643123	-1,560350
H75	-3,703388	1,186518	-1,026165	H75	-1,167554	3,640644	-0,940228
H76	-2,833402	-1,059756	2,529947	H76	-3,133806	1,373037	2,138029
H77	-4,960665	-2,249343	2,301584	H77	-5,285620	2,354049	1,490350
H78	-6,513874	-1,696850	0,430651	H78	-5,395651	4,030484	-0,350528
C79	-1,754181	0,855415	0,975294	C79	-0,674308	1,848670	1,142784
C80	-1,433311	2,303456	0,470158	C80	0,677078	2,333190	0,817837
H81	-0,442221	2,301483	-0,111507	H81	1,674286	1,210019	0,023134
C82	-2,397498	3,068608	-0,396509	C82	1,002453	3,541341	-0,010799
C83	-3,443430	3,769730	0,212006	C83	0,849930	4,786776	0,616974
C84	-2,284835	3,080946	-1,789627	C84	1,461723	3,495144	-1,328820
C85	-4,374479	4,461708	-0,561081	C85	1,131741	5,965214	-0,068793
H86	-3,539360	3,760263	1,295517	H86	0,489269	4,830366	1,642118
C87	-3,216017	3,771514	-2,564848	C87	1,744624	4,677226	-2,015476
H88	-1,470901	2,536644	-2,259117	H88	1,604079	2,535368	-1,811644
C89	-4,264283	4,461779	-1,952618	C89	1,576531	5,913232	-1,392087
H90	-5,187530	4,995948	-0,078129	H90	1,000739	6,922706	0,426909
H91	-3,124619	3,770741	-3,647405	H91	2,103075	4,627843	-3,039582
H92	-4,991317	4,997147	-2,556197	H92	1,792903	6,831451	-1,930231
C93	-2,069338	1,373048	3,527580	C93	-0,988452	2,236675	3,683775
C94	-0,020531	0,780498	2,680540	C94	0,043969	0,333235	2,908198
C95	-1,068001	1,085416	4,656564	C95	-0,792624	1,216578	4,814844
H96	-3,040047	0,901017	3,681966	H96	-1,985230	2,678622	3,668672
H97	-2,223220	2,449446	3,365771	H97	-0,244580	3,045726	3,712420
H98	-1,006403	1,863886	5,416919	H98	-0,321846	1,622484	5,710269
H99	-1,250712	0,114678	5,128533	H99	-1,728991	0,715662	5,081658
N100	-1,343287	0,778175	2,396599	N100	-0,754382	1,364552	2,524922
O101	0,219484	1,014942	3,976008	O101	0,102043	0,222485	4,242407
O102	0,887860	0,618303	1,848544	O102	0,675679	-0,406576	2,144371
H103	4,703366	1,395318	-2,353093	H103	3,681303	-3,280425	-1,514458
N104	1,169658	-2,386403	-1,314869	N104	-1,085086	-2,285135	-1,519589
C105	1,451136	-1,597058	-2,540445	C105	-0,166208	-1,887841	-2,610269
H106	0,528312	-1,287902	-3,053367	H106	-0,410488	-0,899243	-3,024358

Iso4

C1	-2,836136	-2,994277	-3,124542
C2	-0,450569	-2,963668	-3,644260
C3	-1,833267	-2,668976	-4,280964
C4	-1,884882	-3,414609	-1,979532
H5	-3,498509	-3,826252	-3,385556
H6	-1,914933	-1,635029	-4,631322

H7	-2,356025	-3,925955	-1,139378
H8	-3,462063	-2,145142	-2,849296
H9	0,367323	-3,086668	-4,353920
H10	-1,991487	-3,320158	-5,145221
C11	-0,777723	-4,170020	-2,735348
H12	0,050888	-4,480338	-2,091780
H13	-1,149393	-5,032159	-3,296011
C14	1,222434	-1,798046	-2,055600
N15	1,591139	-1,119294	-1,023561
C16	3,085163	-1,303914	-0,838600
C17	3,374985	-2,412577	-1,903151
P18	-1,512349	-1,146720	-0,278427
Ir19	0,385131	0,355230	0,015098
C20	-1,759174	-2,193443	1,195869
C21	-1,842191	-3,619662	3,609432
C22	-1,065253	-3,403670	1,328428
C23	-2,501900	-1,707756	2,283579
C24	-2,542070	-2,418204	3,481844
C25	-1,109920	-4,112866	2,528905
H26	-3,053803	-0,778735	2,189890
H27	-3,125487	-2,037096	4,315687
H28	-0,569820	-5,051091	2,619469
H29	-1,871682	-4,170441	4,544929
O30	2,153004	-2,516198	-2,685065
C31	3,431866	-1,877448	0,529428
C32	2,462520	-2,502201	1,315113
C33	4,771822	-1,921453	0,943624
C34	2,809266	-3,108498	2,521850
H35	1,426669	-2,489493	1,007157
C36	5,120455	-2,537190	2,144093
H37	5,544465	-1,464235	0,332374
C38	4,137412	-3,124466	2,943437
H39	2,030365	-3,556081	3,132705
H40	6,160572	-2,552496	2,456912
H41	4,408855	-3,592054	3,885554
C42	-3,161180	-0,518910	-0,748129
C43	-4,351369	-1,160134	-0,373554
C44	-3,199992	0,509402	-1,701321
C45	-5,563476	-0,769334	-0,941032
H46	-4,335220	-1,970438	0,347993
C47	-4,412078	0,890008	-2,271523
C48	-5,594989	0,251682	-1,892604
H49	-6,481759	-1,269867	-0,647230
H50	-4,435678	1,693868	-3,000905
H51	-6,540056	0,550150	-2,337131
H52	0,123999	1,014749	-1,373579
H53	1,300700	2,218456	1,738425
H54	-0,481667	-3,783790	0,495881

H55	-2,282803	1,017716	-1,982130
H56	4,198267	-2,176342	-2,576931
C57	4,861690	2,526948	-1,860351
C58	4,381949	1,675848	-2,856953
C59	3,832401	0,440389	-2,516553
C60	3,762625	0,027138	-1,179094
C61	4,225034	0,901022	-0,185296
C62	4,774442	2,138128	-0,524585
H63	5,287987	3,490745	-2,122616
H64	4,433255	1,973200	-3,900497
H65	3,444393	-0,196141	-3,307995
H66	4,154411	0,615915	0,858308
H67	5,126530	2,801523	0,259915
C68	-3,333282	3,848014	-0,699825
C69	-2,107575	3,292534	-0,340852
C70	-2,012999	2,397543	0,737557
C71	-3,201390	2,047870	1,401614
C72	-4,428979	2,592048	1,032392
C73	-4,500033	3,509328	-0,013789
H74	-3,374321	4,547696	-1,530091
H75	-1,227523	3,554685	-0,910090
H76	-3,167602	1,330507	2,213504
H77	-5,328838	2,292690	1,562047
H78	-5,453296	3,943468	-0,300625
C79	-0,713307	1,823017	1,205902
C80	0,610364	2,326599	0,899325
H81	1,825470	1,026720	-0,007609
C82	0,941900	3,540869	0,082884
C83	0,641554	4,784539	0,661890
C84	1,567600	3,509123	-1,166016
C85	0,940059	5,970062	-0,004644
H86	0,147672	4,818704	1,630193
C87	1,869364	4,698634	-1,832049
H88	1,824146	2,556698	-1,613793
C89	1,551966	5,929622	-1,259931
H90	0,688676	6,923681	0,450897
H91	2,356015	4,656683	-2,802375
H92	1,779603	6,852897	-1,785106
C93	-1,034213	2,138634	3,742234
C94	0,008614	0,257199	2,933546
C95	-0,849707	1,095411	4,852201
H96	-2,028107	2,587372	3,732191
H97	-0,282415	2,939368	3,785461
H98	-0,387717	1,481131	5,761044
H99	-1,787233	0,584716	5,094972
N100	-0,804562	1,285238	2,566529
O101	0,054176	0,117640	4,265373
O102	0,656841	-0,458017	2,163041

H103	3,544406	-3,383175	-1,435082
N104	-1,099178	-2,242343	-1,501082
C105	-0,182291	-1,853139	-2,597381
H106	-0,411950	-0,859171	-3,006370

C3_ES_Cheltop

C3_ES_Cheltop_SM

C1	-4.0724	-3.51299	0.036369
C2	-1.8955	-4.6104	-0.0578
C3	-3.2708	-4.58461	-0.77373
C4	-3.0409	-3.0685	1.099679
H5	-4.9481	-3.95009	0.527236
H6	-3.1799	-4.33063	-1.83487
H7	-3.4541	-2.50923	1.939156
H8	-4.4174	-2.68016	-0.57691
H9	-1.2803	-5.48425	-0.27085
H10	-3.7418	-5.56971	-0.7131
C11	-2.3053	-4.38271	1.41487
H12	-1.4597	-4.25058	2.09635
H13	-2.9662	-5.16734	1.793514
C14	0.2855	-3.21475	-0.07198
N15	1.00283	-2.14212	-0.06856
C16	2.36915	-2.50715	0.481172
C17	2.31978	-4.05556	0.332861
P18	-1.8319	-0.65359	0.136554
Ir19	0.39762	-0.21244	-0.78742
C20	-2.0534	0.18371	1.747998
C21	-2.1596	1.602723	4.171654
C22	-1.8664	-0.47916	2.967765
C23	-2.2946	1.568215	1.757012
C24	-2.3451	2.270794	2.959086
C25	-1.926	0.227567	4.170467
H26	-2.4684	2.096825	0.823661
H27	-2.5383	3.339764	2.949911
H28	-1.7927	-0.3024	5.109459
H29	-2.2026	2.149461	5.108652
O30	0.89751	-4.35409	0.249968
C31	2.45183	-2.17837	1.977896
C32	1.37517	-1.6637	2.695179
C33	3.64318	-2.47288	2.659895
C34	1.48863	-1.41958	4.065322
H35	0.45567	-1.42313	2.184695
C36	3.75582	-2.23582	4.02645
H37	4.49366	-2.87101	2.112608
C38	2.67593	-1.70178	4.734896

C3_ES_Cheltop_INT2

C1	-4.296	-3.09198	0.982633
C2	-2.2841	-4.45618	0.821148
C3	-3.7472	-4.39582	0.316365
C4	-3.0709	-2.59069	1.783265
H5	-5.1262	-3.30296	1.664684
H6	-3.8076	-4.36995	-0.77618
H7	-3.2878	-1.83811	2.541337
H8	-4.643	-2.35523	0.258351
H9	-1.7969	-5.42435	0.710107
H10	-4.2955	-5.27988	0.653826
C11	-2.4264	-3.90436	2.257373
H12	-1.4743	-3.75305	2.775052
H13	-3.0917	-4.51103	2.877991
C14	-0.0022	-3.40527	0.212148
N15	0.80884	-2.43403	-0.01197
C16	2.19654	-2.90991	0.332237
C17	1.97075	-4.45157	0.379789
P18	-1.7918	-0.5564	0.233929
Ir19	0.20783	-0.45887	-0.85362
C20	-1.8784	0.533078	1.699952
C21	-1.8774	2.251068	3.918953
C22	-1.4465	0.075739	2.953735
C23	-2.2857	1.870496	1.564177
C24	-2.2954	2.718936	2.671326
C25	-1.4459	0.930976	4.054683
H26	-2.6228	2.244792	0.602152
H27	-2.6435	3.742448	2.560719
H28	-1.1148	0.561978	5.020978
H29	-1.8942	2.910789	4.781901
O30	0.52926	-4.58752	0.54402
C31	2.5885	-2.42971	1.733473
C32	1.70798	-1.71826	2.546477
C33	3.85136	-2.7709	2.239724
C34	2.08722	-1.32248	3.829412
H35	0.74018	-1.43794	2.161025
C36	4.22961	-2.38392	3.522989
H37	4.55006	-3.32621	1.618962
C38	3.34796	-1.6513	4.322025

H39	0.64488	-0.9919	4.598414	H39	1.3961	-0.74054	4.43307
H40	4.6875	-2.46151	4.537066	H40	5.21459	-2.64833	3.896729
H41	2.76496	-1.50769	5.799832	H41	3.64663	-1.34047	5.31905
C42	-3.3366	-0.25293	-0.82492	C42	-3.2935	-0.16732	-0.74574
C43	-4.5077	0.269182	-0.25842	C43	-4.4733	0.284508	-0.1253
C44	-3.3393	-0.64039	-2.17425	C44	-3.3284	-0.48705	-2.11096
C45	-5.6562	0.415519	-1.03712	C45	-5.65	0.423664	-0.85889
H46	-4.5322	0.553861	0.788137	H46	-4.4811	0.521324	0.933909
C47	-4.4888	-0.49943	-2.94592	C47	-4.5108	-0.35291	-2.84015
C48	-5.6489	0.03278	-2.37867	C48	-5.6736	0.103163	-2.21927
H49	-6.5594	0.822749	-0.59161	H49	-6.5505	0.778504	-0.36569
H50	-4.4744	-0.78734	-3.99244	H50	-4.5122	-0.59018	-3.89972
H51	-6.5449	0.148404	-2.98167	H51	-6.5919	0.212335	-2.78858
H52	0.07278	-0.86118	-2.16901	H52	-0.555	-1.17989	-2.01873
H53	1.90809	-0.24684	-1.26165	H53	1.88357	2.066437	-1.50151
H54	-1.6612	-1.54403	2.982211	H54	-1.1179	-0.95091	3.070574
H55	-2.4339	-1.03695	-2.62163	H55	-2.426	-0.81333	-2.61379
H56	2.78496	-4.43454	-0.57938	H56	2.24879	-4.9714	-0.54017
C57	5.36018	-0.5051	-1.9464	C57	4.5597	-1.35704	-2.95078
C58	4.70474	-1.63696	-2.43238	C58	3.69354	-2.43533	-3.14809
C59	3.76353	-2.29444	-1.64176	C59	2.98925	-2.97099	-2.07109
C60	3.47169	-1.84552	-0.34648	C60	3.1384	-2.44621	-0.77945
C61	4.12651	-0.70015	0.126049	C61	4.00547	-1.36318	-0.59212
C62	5.06208	-0.03638	-0.66684	C62	4.71354	-0.82563	-1.67003
H63	6.0927	0.008702	-2.56228	H63	5.10793	-0.93597	-3.78865
H64	4.91611	-2.00276	-3.43288	H64	3.55946	-2.85438	-4.14125
H65	3.23529	-3.14309	-2.06502	H65	2.285	-3.77627	-2.25852
H66	3.89157	-0.31695	1.112322	H66	4.10684	-0.91681	0.390129
H67	5.561	0.848177	-0.28036	H67	5.38169	0.01528	-1.50583
C68	1.93349	1.965337	-2.77994	C68	0.9006	3.912055	-1.96872
C69	1.02357	1.732293	-1.54504	C69	0.90492	2.510067	-1.28081
C70	-0.4129	1.716096	-1.49386	C70	-0.2166	1.493179	-1.67255
C71	1.90531	3.837858	-0.31361	C71	0.55144	3.929205	0.961451
C72	1.45963	1.912349	0.873904	C72	1.18452	1.720334	1.055645
C73	2.40814	3.951041	1.136305	C73	1.12996	3.581632	2.342373
H74	0.98897	4.416642	-0.49282	H74	-0.5337	4.064278	0.998729
H75	2.6623	4.142064	-1.03811	H75	1.00409	4.828546	0.544802
H76	3.498	3.875966	1.199972	H76	2.12813	4.004117	2.496873
H77	2.06841	4.848112	1.654792	H77	0.47448	3.857779	3.168377
N78	1.62659	2.398673	-0.37843	N78	0.89963	2.719439	0.201999
O79	1.83816	2.795953	1.808198	O79	1.24665	2.1386	2.331739
O80	1.01104	0.796259	1.159321	O80	1.38949	0.52762	0.782372
H81	2.7232	-4.57591	1.19933	H81	2.4523	-4.92467	1.233927
N82	-1.9205	-2.31229	0.468105	N82	-1.9946	-2.11531	0.864439
C83	-1.185	-3.27418	-0.39165	C83	-1.5052	-3.30924	0.124773

H84	-1.2822	-3.03959	-1.46126
C85	-3.1362	2.229675	-4.78445
C86	-3.2778	2.951964	-3.60017
C87	-2.3899	2.744747	-2.54629
C88	-1.3477	1.809618	-2.64569
C89	-1.243	1.06398	-3.829
C90	-2.1161	1.282377	-4.89112
H91	-3.8223	2.392279	-5.61051
H92	-4.0785	3.678302	-3.49481
H93	-2.5044	3.321872	-1.63128
H94	-0.4953	0.287295	-3.90029
H95	-2.0095	0.697646	-5.80077
H96	-0.8069	2.234557	-0.62012
C97	2.18427	0.805459	-3.75435
H98	3.00212	1.089173	-4.42542
H99	2.47506	-0.10467	-3.2305
H100	1.31774	0.591059	-4.37995
C101	1.49124	3.221134	-3.56327
H102	2.28103	3.519195	-4.25978
H103	0.58888	3.020677	-4.14375
H104	1.27717	4.075145	-2.91508
H105	2.91225	2.168069	-2.32793
X	0.28333	1.758175	-1.43512

C3_ES_Cheltop_TS1

C1	-3.9716	-3.84047	-0.22732
C2	-1.7434	-4.82485	-0.09421
C3	-3.0499	-4.89436	-0.927
C4	-3.0726	-3.33449	0.924586
H5	-4.8806	-4.29993	0.174117
H6	-2.8789	-4.67259	-1.98543
H7	-3.5945	-2.78729	1.709782
H8	-4.2737	-3.02599	-0.88813
H9	-1.061	-5.66305	-0.23328
H10	-3.476	-5.89997	-0.86912
C11	-2.2984	-4.60056	1.330739
H12	-1.5272	-4.40965	2.08309
H13	-2.9452	-5.41734	1.663052
C14	0.35836	-3.29588	-0.00243
N15	0.99191	-2.17409	0.02348
C16	2.36691	-2.43008	0.588551
C17	2.43741	-3.98411	0.464518
P18	-1.8699	-0.85554	0.159474
Ir19	0.21484	-0.29456	-0.75023
C20	-2.0886	-0.11032	1.817309

H84	-1.7551	-3.26332	-0.94538
C85	-1.0422	1.173228	-5.92307
C86	-2.0173	1.621529	-5.03434
C87	-1.7262	1.734964	-3.67359
C88	-0.466	1.395222	-3.15998
C89	0.50628	0.953458	-4.07494
C90	0.22617	0.846751	-5.43443
H91	-1.262	1.084877	-6.98299
H92	-3.0067	1.889193	-5.39599
H93	-2.4964	2.08598	-2.99232
H94	1.50704	0.712717	-3.72822
H95	1.00176	0.511658	-6.11771
H96	-1.1348	1.888391	-1.22404
C97	1.54501	3.922157	-3.36618
H98	1.74703	4.957562	-3.66039
H99	2.49809	3.38187	-3.37756
H100	0.89757	3.481589	-4.12383
C101	-0.4781	4.590549	-2.02084
H102	-0.3691	5.637388	-2.32172
H103	-1.1172	4.100269	-2.75763
H104	-1.0058	4.576261	-1.06305
H105	1.56698	4.527413	-1.35049
H106	1.87986	-0.2998	-1.55546
H107	1.43672	-0.66616	-2.13286

C3_ES_Cheltop_TS3

C1	-4.2969	-3.17722	0.770562
C2	-2.2627	-4.5163	0.671137
C3	-3.697	-4.45095	0.088825
C4	-3.1256	-2.69894	1.662421
H5	-5.1655	-3.42072	1.391329
H6	-3.6985	-4.38606	-1.00388
H7	-3.3927	-1.97884	2.436389
H8	-4.6052	-2.41508	0.054253
H9	-1.7576	-5.47402	0.548636
H10	-4.2524	-5.35222	0.363885
C11	-2.4933	-4.02561	2.118346
H12	-1.5739	-3.88736	2.694892
H13	-3.1851	-4.66536	2.673612
C14	0.02766	-3.39217	0.223953
N15	0.84302	-2.41704	0.001216
C16	2.22063	-2.85388	0.451641
C17	2.00124	-4.39214	0.574999
P18	-1.7776	-0.60947	0.230384
Ir19	0.34883	-0.55441	-0.93573

C21	-2.2847	1.161643	4.311486
C22	-2.0581	-0.86154	2.998594
C23	-2.1996	1.287729	1.898078
C24	-2.2986	1.917674	3.136015
C25	-2.1624	-0.22611	4.23858
H26	-2.2171	1.88275	0.988634
H27	-2.3876	2.999155	3.18524
H28	-2.1467	-0.82065	5.147644
H29	-2.3653	1.652995	5.276446
O30	1.04446	-4.39188	0.329487
C31	2.41909	-2.05501	2.073619
C32	1.32909	-1.51875	2.755174
C33	3.60457	-2.30859	2.782131
C34	1.42384	-1.21525	4.115006
H35	0.41065	-1.31082	2.228522
C36	3.69923	-2.01125	4.138392
H37	4.4656	-2.72241	2.262148
C38	2.60593	-1.45684	4.809953
H39	0.56648	-0.77921	4.61894
H40	4.62609	-2.20695	4.669889
H41	2.67987	-1.21718	5.866859
C42	-3.3695	-0.37018	-0.76992
C43	-4.4969	0.209728	-0.17081
C44	-3.4003	-0.67598	-2.13931
C45	-5.6279	0.493786	-0.93698
H46	-4.4984	0.441256	0.888989
C47	-4.5328	-0.39683	-2.89852
C48	-5.6469	0.194288	-2.29907
H49	-6.4953	0.947555	-0.46613
H50	-4.5371	-0.61909	-3.96082
H51	-6.5271	0.421404	-2.89347
H52	-0.1712	-1.04069	-2.06716
H53	1.70962	0.029888	-1.39183
H54	-1.9303	-1.9377	2.957132
H55	-2.5286	-1.11529	-2.61208
H56	2.96926	-4.33648	-0.42227
C57	5.22545	-0.28049	-1.87891
C58	4.58167	-1.41767	-2.37027
C59	3.67651	-2.11074	-1.5688
C60	3.41081	-1.69616	-0.25613
C61	4.04682	-0.54245	0.219896
C62	4.94723	0.157656	-0.58433
H63	5.93625	0.256804	-2.50039
H64	4.77694	-1.76097	-3.38186
H65	3.15795	-2.9677	-1.98837

C20	-1.8999	0.466114	1.704466
C21	-1.8669	2.193601	3.921646
C22	-1.5333	-0.00759	2.973085
C23	-2.2446	1.821271	1.560673
C24	-2.2346	2.675238	2.663863
C25	-1.5161	0.851101	4.071213
H26	-2.5574	2.206711	0.594505
H27	-2.532	3.713673	2.542584
H28	-1.2386	0.466128	5.048259
H29	-1.8676	2.856944	4.781884
O30	0.55416	-4.53716	0.655517
C31	2.52175	-2.30706	1.853458
C32	1.59517	-1.56411	2.580919
C33	3.75018	-2.62674	2.450624
C34	1.8934	-1.1173	3.868419
H35	0.65276	-1.29968	2.128198
C36	4.048	-2.19009	3.738933
H37	4.48606	-3.20322	1.89611
C38	3.11953	-1.42693	4.45214
H39	1.16707	-0.51052	4.401548
H40	5.00748	-2.4393	4.183255
H41	3.35581	-1.07727	5.453192
C42	-3.254	-0.20105	-0.77829
C43	-4.4454	0.282086	-0.20825
C44	-3.2433	-0.55299	-2.1366
C45	-5.5944	0.415703	-0.98481
H46	-4.483	0.544322	0.84443
C47	-4.3996	-0.43135	-2.90712
C48	-5.5748	0.054631	-2.33502
H49	-6.5082	0.792762	-0.53406
H50	-4.3718	-0.69657	-3.95962
H51	-6.4736	0.15507	-2.93688
H52	-0.3351	-1.35333	-2.08891
H53	1.9093	2.047066	-1.57735
H54	-1.2634	-1.04978	3.102968
H55	-2.3254	-0.90007	-2.59754
H56	2.33884	-4.96377	-0.2917
C57	4.9915	-1.65554	-2.66098
C58	4.11707	-2.72466	-2.86615
C59	3.25457	-3.12462	-1.84803
C60	3.25447	-2.47267	-0.60679
C61	4.12044	-1.38986	-0.41773
C62	4.98543	-0.98797	-1.43605
H63	5.66776	-1.34363	-3.452
H64	4.10185	-3.24424	-3.81998

H66	3.83825	-0.18886	1.223246
H67	5.44019	1.043113	-0.19106
C68	2.0338	1.6051	-3.00445
C69	1.18782	1.494998	-1.70724
C70	-0.2759	1.612147	-1.58891
C71	2.47026	3.511606	-0.65039
C72	1.61071	1.840935	0.690614
C73	2.9891	3.632853	0.791963
H74	1.70199	4.260161	-0.8841
H75	3.27423	3.584578	-1.38389
H76	4.03148	3.311369	0.880791
H77	2.86862	4.626473	1.224117
N78	1.88932	2.165835	-0.59849
O79	2.16814	2.705323	1.549753
O80	0.93699	0.876355	1.067473
H81	2.84166	-4.46317	1.354248
N82	-1.9461	-2.52795	0.378479
C83	-1.0877	-3.45325	-0.40004
H84	-1.1262	-3.24292	-1.47882
C85	-3.0469	2.291296	-4.82306
C86	-3.0363	3.103206	-3.68948
C87	-2.1341	2.851538	-2.65689
C88	-1.2321	1.777746	-2.72055
C89	-1.2909	0.943001	-3.84653
C90	-2.1698	1.207523	-4.8938
H91	-3.7395	2.491665	-5.63531
H92	-3.7251	3.939541	-3.61036
H93	-2.124	3.50449	-1.78674
H94	-0.6633	0.062566	-3.88473
H95	-2.1837	0.551273	-5.76007
H96	-0.547	2.28378	-0.77153
C97	2.01109	0.40436	-3.96409
H98	2.89172	0.458274	-4.6119
H99	2.04447	-0.54517	-3.42681
H100	1.13408	0.415496	-4.61069
C101	1.70977	2.89827	-3.78073
H102	2.44062	3.03112	-4.58456
H103	0.71803	2.837916	-4.2325
H104	1.73472	3.796926	-3.15982
H105	3.0639	1.659055	-2.63114
X	0.28333	1.758175	-1.43512

C3_ES_Cheltop_INT1

C1	-4.2887	-3.09818	0.869072
C2	-2.2605	-4.44892	0.851244

H65	2.55447	-3.93056	-2.04795
H66	4.10263	-0.84298	0.517314
H67	5.65427	-0.14781	-1.27046
C68	1.04971	3.919447	-2.09962
C69	0.96041	2.551368	-1.36479
C70	-0.21	1.553455	-1.70808
C71	0.56876	3.95237	0.859355
C72	1.26956	1.753956	0.95501
C73	1.13553	3.612246	2.248243
H74	-0.5221	4.051729	0.878148
H75	1.00098	4.867278	0.452636
H76	2.11767	4.06476	2.418486
H77	0.45931	3.863606	3.065402
N78	0.9749	2.762593	0.104361
O79	1.29638	2.173504	2.233194
O80	1.50393	0.574799	0.668829
H81	2.43029	-4.80858	1.484463
N82	-2.0064	-2.17609	0.828351
C83	-1.4686	-3.33114	0.061657
H84	-1.667	-3.24417	-1.01618
C85	-2.1183	1.710536	-5.59002
C86	-2.7241	2.279431	-4.47034
C87	-2.0957	2.21098	-3.22749
C88	-0.8467	1.593212	-3.0773
C89	-0.2611	1.004428	-4.2079
C90	-0.8853	1.067274	-5.4524
H91	-2.6066	1.754475	-6.55948
H92	-3.694	2.760861	-4.55748
H93	-2.5906	2.632302	-2.35614
H94	0.68585	0.478613	-4.11404
H95	-0.4125	0.606637	-6.31516
H96	-0.9951	1.774794	-0.98674
C97	1.64794	3.774623	-3.50914
H98	1.86282	4.76653	-3.91949
H99	2.58708	3.210564	-3.49326
H100	0.96012	3.274521	-4.19256
C101	-0.2393	4.758856	-2.15478
H102	0.01023	5.798225	-2.39101
H103	-0.907	4.395999	-2.93674
H104	-0.8024	4.760881	-1.21759
H105	1.79522	4.478239	-1.51703
H106	1.73418	-0.76873	-1.72083
H107	0.80616	0.406179	-2.08986

C3_ES_Cheltop_PROD

C3	-3.712	-4.44089	0.309091
C4	-3.0956	-2.54123	1.682443
H5	-5.1478	-3.26836	1.526023
H6	-3.747	-4.49469	-0.78348
H7	-3.3427	-1.74932	2.389882
H8	-4.6024	-2.4071	0.084879
H9	-1.7538	-5.41309	0.805324
H10	-4.2598	-5.30432	0.697088
C11	-2.449	-3.81708	2.248336
H12	-1.5144	-3.6214	2.783
H13	-3.1238	-4.39762	2.883751
C14	0.01735	-3.40518	0.187164
N15	0.78625	-2.38156	0.11211
C16	2.19563	-2.82656	0.311455
C17	2.03718	-4.35243	0.033904
P18	-1.7689	-0.56058	0.109939
Ir19	0.24654	-0.46169	-0.72223
C20	-1.8838	0.589153	1.525037
C21	-1.7337	2.453166	3.618267
C22	-1.4461	0.194344	2.797795
C23	-2.2315	1.933685	1.30663
C24	-2.1616	2.856345	2.350502
C25	-1.3769	1.121446	3.836931
H26	-2.5721	2.256652	0.327216
H27	-2.4539	3.888127	2.174886
H28	-1.042	0.802343	4.819226
H29	-1.687	3.170824	4.43206
O30	0.61293	-4.60071	0.276796
C31	2.60818	-2.59932	1.768861
C32	1.80981	-1.87379	2.655348
C33	3.81001	-3.1499	2.234439
C34	2.20688	-1.69649	3.981142
H35	0.89452	-1.4226	2.300153
C36	4.20715	-2.97452	3.558416
H37	4.45031	-3.70844	1.555879
C38	3.4042	-2.24514	4.43809
H39	1.57863	-1.11935	4.65434
H40	5.14249	-3.40626	3.902484
H41	3.7122	-2.10717	5.470548
C42	-3.1978	-0.20304	-0.96946
C43	-4.4048	0.27124	-0.42185
C44	-3.1535	-0.55379	-2.327
C45	-5.5367	0.399286	-1.22337
H46	-4.4654	0.534278	0.629909
C47	-4.2928	-0.43061	-3.12122

C1	-4.2226	-3.44318	0.909874
C2	-2.1136	-4.6673	0.87345
C3	-3.5567	-4.72665	0.312056
C4	-3.0717	-2.83842	1.747925
H5	-5.0735	-3.68934	1.553586
H6	-3.5755	-4.74437	-0.78215
H7	-3.3704	-2.08059	2.473023
H8	-4.5737	-2.74775	0.145653
H9	-1.5561	-5.60142	0.810693
H10	-4.0542	-5.63543	0.662617
C11	-2.3546	-4.08876	2.286179
H12	-1.4374	-3.85433	2.834697
H13	-2.9979	-4.72758	2.897866
C14	0.10437	-3.45875	0.31115
N15	0.88217	-2.47209	-0.00108
C16	2.27999	-2.80503	0.503924
C17	2.13293	-4.34277	0.657935
P18	-1.83	-0.7752	0.167791
Ir19	0.29932	-0.75864	-0.95688
C20	-1.9933	0.383525	1.579379
C21	-1.9587	2.28838	3.649577
C22	-1.6122	0.019712	2.878895
C23	-2.361	1.718558	1.332848
C24	-2.3421	2.662481	2.359841
C25	-1.5981	0.964542	3.9045
H26	-2.702	2.014873	0.344499
H27	-2.6506	3.684987	2.156997
H28	-1.3116	0.662647	4.907954
H29	-1.9563	3.019468	4.453202
O30	0.69735	-4.54033	0.816277
C31	2.50009	-2.2213	1.90987
C32	1.50297	-1.55338	2.616415
C33	3.73739	-2.44	2.535502
C34	1.73943	-1.08023	3.908242
H35	0.55062	-1.3664	2.145425
C36	3.97275	-1.97796	3.82831
H37	4.5284	-2.95766	1.998687
C38	2.97322	-1.28851	4.520964
H39	0.9562	-0.53256	4.424139
H40	4.93981	-2.15053	4.292156
H41	3.15949	-0.91898	5.525171
C42	-3.3052	-0.38891	-0.85908
C43	-4.5464	-0.05935	-0.28438
C44	-3.2055	-0.47118	-2.25575
C45	-5.6569	0.180191	-1.09075

C48	-5.4827	0.046799	-2.57403	H46	-4.6427	0.018874	0.795126
H49	-6.4615	0.771322	-0.79202	C47	-4.3191	-0.22718	-3.06176
H50	-4.2379	-0.68898	-4.17406	C48	-5.5456	0.097778	-2.48289
H51	-6.3667	0.148412	-3.19657	H49	-6.6094	0.434081	-0.63393
H52	-0.4056	-1.18071	-1.94516	H50	-4.218	-0.27412	-4.14182
H53	2.19711	1.507362	-1.53638	H51	-6.4115	0.291187	-3.10932
H54	-1.1575	-0.8371	2.972243	H52	-0.2991	-1.64707	-2.0953
H55	-2.2247	-0.89347	-2.77046	H53	1.95181	1.709941	-1.61163
H56	2.24539	-4.62643	-1.00474	H54	-1.328	-1.00509	3.091615
C57	4.3878	-0.50862	-2.6467	H55	-2.2516	-0.70747	-2.71474
C58	3.36216	-1.3746	-3.03228	H56	2.44209	-4.91357	-0.22007
C59	2.69127	-2.13721	-2.07366	C57	5.1334	-1.47704	-2.47679
C60	3.048	-2.06419	-0.71456	C58	4.38666	-2.63666	-2.68828
C61	4.07857	-1.19608	-0.34102	C59	3.49146	-3.07853	-1.71592
C62	4.7426	-0.42741	-1.29907	C60	3.33395	-2.37774	-0.51358
H63	4.9086	0.087731	-3.39016	C61	4.07319	-1.2048	-0.3196
H64	3.08076	-1.45922	-4.07807	C62	4.96857	-0.76072	-1.29114
H65	1.87816	-2.78446	-2.3892	H63	5.83474	-1.1336	-3.23214
H66	4.34905	-1.0986	0.703146	H64	4.4957	-3.19625	-3.61283
H67	5.53877	0.24223	-0.98656	H65	2.89333	-3.95946	-1.92725
C68	1.73891	3.446856	-2.29745	H66	3.93053	-0.62396	0.583503
C69	1.35772	2.207514	-1.42684	H67	5.53817	0.148754	-1.12023
C70	0.01132	1.44765	-1.67268	C68	1.55821	3.682572	-2.30062
C71	0.95755	3.875681	0.593364	C69	1.13433	2.426881	-1.49399
C72	1.47698	1.674373	0.972223	C70	-0.1853	1.64719	-1.86493
C73	1.35276	3.65142	2.06242	C71	0.69355	3.935947	0.633225
H74	-0.1254	4.004701	0.475823	C72	1.22131	1.698357	0.854679
H75	1.46985	4.731639	0.152773	C73	1.10604	3.605285	2.078696
H76	2.34374	4.054454	2.292877	H74	-0.3903	4.084448	0.539147
H77	0.61816	4.025331	2.77545	H75	1.20954	4.816931	0.248842
N78	1.39728	2.605518	0.006932	H76	2.09882	3.995537	2.324011
O79	1.40956	2.207839	2.201961	H77	0.37937	3.927256	2.824517
O80	1.60127	0.446749	0.812955	N78	1.11838	2.709479	-0.04458
H81	2.60273	-4.98606	0.713875	O79	1.16556	2.158513	2.116751
N82	-2.0016	-2.10573	0.765805	O80	1.35567	0.496284	0.615768
C83	-1.4853	-3.33066	0.103218	H81	2.62519	-4.72899	1.54824
H84	-1.7293	-3.35541	-0.97057	N82	-2.0041	-2.31227	0.855074
C85	-1.2722	0.973297	-5.78468	C83	-1.3976	-3.48474	0.168485
C86	-2.0209	1.737683	-4.8916	H84	-1.609	-3.48841	-0.91022
C87	-1.5912	1.89701	-3.57252	C85	-2.7345	2.650835	-5.19567
C88	-0.4063	1.305098	-3.1136	C86	-3.0151	3.136699	-3.91728
C89	0.33495	0.538516	-4.0288	C87	-2.1807	2.810211	-2.85106
C90	-0.0884	0.37327	-5.34475	C88	-1.0497	2.00881	-3.04858
H91	-1.603	0.846689	-6.81145	C89	-0.7881	1.506574	-4.32832
H92	-2.9452	2.208055	-5.21639	C90	-1.6225	1.830979	-5.39815

H93	-2.1925	2.484682	-2.88235
H94	1.2536	0.067191	-3.69478
H95	0.50584	-0.22291	-6.03247
H96	-0.7573	2.050167	-1.1726
C97	2.46702	3.049723	-3.59239
H98	2.88561	3.942112	-4.06913
H99	3.2926	2.357658	-3.39165
H100	1.79227	2.575037	-4.30616
C101	0.58469	4.413211	-2.61118
H102	0.98401	5.348959	-3.01587
H103	-0.085	3.986989	-3.35987
H104	-0.0208	4.664822	-1.73582
H105	2.48301	3.98452	-1.69237
X	0.28333	1.758175	-1.43512

C3_ES_Cheltop_TS2

C1	-4.297	-3.13353	0.572658
C2	-2.261	-4.47681	0.547246
C3	-3.6737	-4.40787	-0.08718
C4	-3.1563	-2.65575	1.502212
H5	-5.185	-3.37673	1.165185
H6	-3.6346	-4.34135	-1.17904
H7	-3.4472	-1.93238	2.264404
H8	-4.5822	-2.37148	-0.15352
H9	-1.7548	-5.43618	0.445561
H10	-4.2411	-5.30804	0.16545
C11	-2.5422	-3.98184	1.983488
H12	-1.6435	-3.84445	2.592057
H13	-3.255	-4.61759	2.516006
C14	0.05239	-3.3602	0.183945
N15	0.85883	-2.37189	0.013934
C16	2.22965	-2.81867	0.447998
C17	2.01637	-4.36618	0.561849
P18	-1.7687	-0.5562	0.167501
Ir19	0.19377	-0.39543	-0.80124
C20	-1.8301	0.451363	1.692376
C21	-1.7448	2.039978	4.003726
C22	-1.4901	-0.11299	2.9305
C23	-2.1173	1.824591	1.622403
C24	-2.0839	2.60993	2.773907
C25	-1.4456	0.678874	4.077815
H26	-2.3883	2.278589	0.674003
H27	-2.3342	3.665761	2.712956
H28	-1.186	0.228186	5.031213
H29	-1.7241	2.652354	4.900684

H91	-3.3856	2.901023	-6.02817
H92	-3.8886	3.759775	-3.749
H93	-2.4071	3.183842	-1.85502
H94	0.06227	0.846359	-4.48388
H95	-1.4087	1.437376	-6.38757
H96	-0.8217	1.674974	-0.98522
C97	2.19618	3.280483	-3.64076
H98	2.59896	4.166252	-4.14209
H99	3.01717	2.568907	-3.50215
H100	1.46075	2.830635	-4.31243
C101	0.48917	4.767987	-2.52982
H102	0.9802	5.715454	-2.77394
H103	-0.1631	4.511255	-3.36524
H104	-0.1515	4.942863	-1.66137
H105	2.3607	4.120634	-1.69076
H106	1.66898	-0.82986	-1.79182
H107	0.1548	0.579674	-2.16827

O30	0.56974	-4.52575	0.58197
C31	2.56953	-2.26758	1.835551
C32	1.70509	-1.43373	2.541956
C33	3.76833	-2.66539	2.446553
C34	2.03512	-0.98152	3.819648
H35	0.78722	-1.10609	2.079702
C36	4.1	-2.21866	3.723263
H37	4.45691	-3.31544	1.912834
C38	3.23308	-1.3695	4.415003
H39	1.35517	-0.3098	4.335723
H40	5.03709	-2.53026	4.17544
H41	3.49434	-1.01483	5.407901
C42	-3.2653	-0.1181	-0.79062
C43	-4.3998	0.435964	-0.17241
C44	-3.3588	-0.55733	-2.12047
C45	-5.5955	0.559855	-0.87831
H46	-4.3631	0.756535	0.863112
C47	-4.5608	-0.44506	-2.81636
C48	-5.6797	0.115874	-2.19942
H49	-6.4639	0.994379	-0.39157
H50	-4.6142	-0.77857	-3.84815
H51	-6.6143	0.208069	-2.74506
H52	-0.5612	-1.11977	-1.95976
H53	1.80926	2.099095	-1.7609
H54	-1.2572	-1.17014	2.995755
H55	-2.4877	-0.96664	-2.61967
H56	2.39973	-4.92418	-0.29542
C57	4.98382	-1.82318	-2.75871
C58	3.9189	-2.70024	-2.97358
C59	3.05283	-3.0113	-1.92794
C60	3.23588	-2.46174	-0.65006
C61	4.29033	-1.56665	-0.4507
C62	5.16064	-1.25425	-1.49855
H63	5.66373	-1.57985	-3.57007
H64	3.76037	-3.1391	-3.95453
H65	2.21237	-3.67303	-2.12049
H66	4.42853	-1.09801	0.516123
H67	5.97765	-0.55952	-1.32523
C68	0.78369	3.955274	-2.06348
C69	0.87697	2.545255	-1.39924
C70	-0.2934	1.53335	-1.63383
C71	0.69693	3.853125	0.91878
C72	1.51243	1.702277	0.82215
C73	1.35373	3.450031	2.253027
H74	-0.391	3.921546	1.006419

H75	1.07967	4.802444	0.54149
H76	2.30549	3.961607	2.424164
H77	0.69712	3.582201	3.113323
N78	1.08365	2.723545	0.066815
O79	1.62542	2.031423	2.117594
O80	1.79616	0.553856	0.426818
H81	2.41173	-4.78414	1.486162
N82	-2.0089	-2.14095	0.698381
C83	-1.4385	-3.29801	-0.03524
H84	-1.5934	-3.21741	-1.12069
C85	-1.5106	1.146554	-5.77937
C86	-2.3822	1.674082	-4.82801
C87	-1.9754	1.801174	-3.49897
C88	-0.6949	1.406252	-3.08397
C89	0.17208	0.883495	-4.05963
C90	-0.2274	0.754202	-5.3873
H91	-1.8225	1.047378	-6.81503
H92	-3.3822	1.987475	-5.11574
H93	-2.6708	2.201501	-2.76591
H94	1.17878	0.601135	-3.77538
H95	0.46648	0.353698	-6.12133
H96	-1.1522	1.964551	-1.10668
C97	1.25282	3.966439	-3.52888
H98	1.3788	5.002295	-3.86099
H99	2.21559	3.458305	-3.64978
H100	0.5326	3.488679	-4.19355
C101	-0.5917	4.633716	-1.95204
H102	-0.5142	5.683457	-2.25243
H103	-1.3102	4.153623	-2.6188
H104	-1.0104	4.612424	-0.94203
H105	1.51639	4.567496	-1.51991
H106	2.68306	-0.01544	-1.97187
H107	2.31957	-0.39918	-2.49978

C3_ES_Chelbot

C3_ES_Chelbot_SM

C1	-3.92334	-3.52563	-0.95247
C2	-1.6669	-4.46398	-0.96739
C3	-2.92551	-4.31793	-1.86331
C4	-3.09225	-3.32181	0.333851
H5	-4.82519	-4.10781	-0.73666
H6	-2.70519	-3.79957	-2.80185
H7	-3.65581	-3.0096	1.213869
H8	-4.23468	-2.57194	-1.38269
H9	-0.95787	-5.22735	-1.2881
H10	-3.31721	-5.30626	-2.1198

C3_ES_Chelbot_int2

C1	-3.64301	-3.342	-1.96871
C2	-1.38485	-4.21093	-1.64029
C3	-2.43784	-3.95995	-2.75276
C4	-3.07706	-3.23895	-0.53632
H5	-4.51478	-4.00482	-1.97916
H6	-2.05735	-3.29438	-3.53389
H7	-3.81167	-3.02735	0.241043
H8	-3.95996	-2.37275	-2.3521
H9	-0.60686	-4.92936	-1.89761
H10	-2.71222	-4.90405	-3.23129

C11	-2.3003	-4.63802	0.430447	C11	-2.27591	-4.5511	-0.42848
H12	-1.57272	-4.65033	1.247961	H12	-1.71426	-4.66434	0.503192
H13	-2.94055	-5.52192	0.499462	H13	-2.89355	-5.43976	-0.58551
C14	0.369131	-3.00871	-0.27084	C14	0.404818	-2.89054	-0.32633
N15	0.92404	-1.93078	0.158627	N15	0.87795	-1.88895	0.332738
C16	2.347562	-2.22891	0.526082	C16	2.162049	-2.31954	0.997783
C17	2.388381	-3.78733	0.327028	C17	2.190737	-3.85033	0.634477
P18	-1.92296	-0.72309	0.399554	P18	-1.9584	-0.74854	0.288742
Ir19	0.099792	0.075575	-0.19174	Ir19	0.164935	0.204216	-0.13281
C20	-2.18718	-0.6099	2.204439	C20	-2.10105	-1.0866	2.091348
C21	-2.3016	-0.37148	4.996218	C21	-2.24105	-1.58468	4.852575
C22	-1.91962	-1.71597	3.022034	C22	-2.1699	-2.39773	2.575872
C23	-2.50669	0.621292	2.800085	C23	-2.08061	-0.02465	3.011334
C24	-2.5647	0.73708	4.187008	C24	-2.16112	-0.27208	4.379964
C25	-1.97939	-1.59624	4.41165	C25	-2.23653	-2.64456	3.948152
H26	-2.70141	1.493899	2.185441	H26	-1.98644	0.998335	2.665624
H27	-2.81177	1.69432	4.636825	H27	-2.14932	0.56071	5.077464
H28	-1.76902	-2.46078	5.0347	H28	-2.2776	-3.66877	4.307221
H29	-2.34532	-0.27826	6.077518	H29	-2.29267	-1.77798	5.92009
O30	1.098317	-4.12724	-0.26138	O30	1.068448	-4.04604	-0.26674
C31	2.640009	-1.88956	1.983252	C31	2.173263	-2.16289	2.516515
C32	1.638424	-1.45846	2.857001	C32	1.238365	-1.41497	3.228407
C33	3.931119	-2.10161	2.490286	C33	3.20723	-2.80017	3.223892
C34	1.926937	-1.20446	4.199261	C34	1.337898	-1.29482	4.618064
H35	0.630036	-1.3172	2.495765	H35	0.430534	-0.92738	2.707074
C36	4.219535	-1.84991	3.830055	C36	3.303741	-2.68547	4.607849
H37	4.720206	-2.45339	1.831239	H37	3.960922	-3.37007	2.684816
C38	3.21848	-1.39083	4.689012	C38	2.367035	-1.92532	5.313566
H39	1.130455	-0.86488	4.855716	H39	0.59684	-0.70666	5.149342
H40	5.226995	-2.01201	4.202302	H40	4.114522	-3.18221	5.133048
H41	3.444835	-1.19092	5.732406	H41	2.44303	-1.82865	6.392534
C42	-3.41981	-0.01954	-0.39514	C42	-3.59498	-0.03835	-0.16662
C43	-4.58916	0.304597	0.305617	C43	-4.55131	0.333242	0.787611
C44	-3.4058	0.096919	-1.79534	C44	-3.9035	0.107644	-1.52884
C45	-5.71257	0.771721	-0.38099	C45	-5.78061	0.861629	0.388538
H46	-4.63462	0.193591	1.383547	H46	-4.34809	0.212828	1.845716
C47	-4.53065	0.555811	-2.47584	C47	-5.13497	0.626976	-1.92358
C48	-5.68554	0.905913	-1.76893	C48	-6.07584	1.015932	-0.96532
H49	-6.61134	1.024906	0.17397	H49	-6.5099	1.144413	1.142094
H50	-4.51022	0.635703	-3.55923	H50	-5.37182	0.709254	-2.98109
H51	-6.56144	1.268497	-2.29858	H51	-7.03563	1.419723	-1.2735
H52	0.269764	0.481403	1.299186	H52	0.247393	0.567546	1.376663
H53	1.855511	0.762868	-0.591	H53	1.342812	2.041705	-2.28691
H54	-1.64145	-2.66388	2.574681	H54	-2.14988	-3.23434	1.891416
H55	-2.52153	-0.1967	-2.34931	H55	-3.19864	-0.22024	-2.28441

H56	3.168344	-4.11754	-0.36028
C57	4.670766	-0.13685	-2.49537
C58	3.784599	-1.16014	-2.84473
C59	3.074856	-1.83394	-1.85571
C60	3.235688	-1.50546	-0.49948
C61	4.123618	-0.47986	-0.16227
C62	4.83581	0.2	-1.15369
H63	5.226377	0.390975	-3.26526
H64	3.643591	-1.43026	-3.88732
H65	2.372592	-2.60977	-2.14909
H66	4.254274	-0.18689	0.870528
H67	5.510014	1.002074	-0.86793
C68	-0.62429	3.259812	-0.19303
C69	0.024602	2.032898	-0.8655
C70	1.579927	1.862547	-0.82691
C71	-0.28766	3.105033	-3.24037
C72	-0.29748	0.824549	-2.96531
C73	-0.71953	2.395815	-4.53505
H74	0.742489	3.48602	-3.28762
H75	-0.95366	3.926479	-2.97187
H76	-1.7962	2.485915	-4.71018
H77	-0.17106	2.706907	-5.42425
N78	-0.38344	1.990335	-2.29227
O79	-0.4233	0.990938	-4.28982
O80	-0.12168	-0.29793	-2.45422
H81	2.470472	-4.32189	1.274348
N82	-1.97825	-2.37383	0.054894
C83	-1.02543	-3.05957	-0.84711
H84	-0.96204	-2.57133	-1.82906
H85	1.981595	1.925169	-1.84452
C86	4.055047	4.211489	1.8045
C87	3.697967	4.693083	0.543954
C88	2.898972	3.919948	-0.29866
C89	2.439582	2.664388	0.113423
C90	2.805336	2.185018	1.376311
C91	3.608374	2.954134	2.216858
H92	4.680635	4.811273	2.459089
H93	4.04499	5.668248	0.21427
H94	2.629798	4.295591	-1.28377
H95	2.446525	1.215099	1.706133
H96	3.884096	2.569337	3.194691
C97	-0.36298	3.398097	1.316545
H98	-0.61659	2.47241	1.839296
H99	0.670866	3.642688	1.55095
H100	-0.99114	4.200457	1.718792

H56	3.098247	-4.15753	0.113064
C57	5.450083	-0.28326	-0.98842
C58	4.573699	-1.10012	-1.70844
C59	3.523591	-1.74052	-1.05405
C60	3.325286	-1.57287	0.325557
C61	4.185331	-0.72515	1.030878
C62	5.248825	-0.09694	0.377753
H63	6.277528	0.207292	-1.49292
H64	4.713525	-1.24508	-2.77584
H65	2.854014	-2.37803	-1.62674
H66	4.041203	-0.56239	2.091621
H67	5.918106	0.542018	0.945789
C68	-0.8588	3.214511	0.184472
C69	-0.25795	2.195762	-0.81744
C70	0.991321	2.808932	-1.5869
C71	-1.87967	3.005975	-2.76986
C72	-0.99679	0.882997	-2.71721
C73	-2.39057	2.167577	-3.95803
H74	-1.17039	3.779501	-3.08386
H75	-2.69378	3.484488	-2.22395
H76	-3.44511	1.904876	-3.85014
H77	-2.21992	2.628417	-4.9315
N78	-1.21933	1.971741	-1.95805
O79	-1.61939	0.93375	-3.9033
O80	-0.3115	-0.10484	-2.38448
H81	2.024153	-4.48548	1.505407
N82	-1.97523	-2.22442	-0.52942
C83	-0.81266	-2.83566	-1.22049
H84	-0.52645	-2.2433	-2.09527
H85	0.593505	3.619824	-2.20985
C86	4.451846	4.573964	0.330182
C87	3.261144	5.295305	0.214795
C88	2.150006	4.714793	-0.38973
C89	2.189726	3.397545	-0.87533
C90	3.399925	2.698549	-0.77949
C91	4.51859	3.278304	-0.1774
H92	5.323446	5.027757	0.793108
H93	3.203195	6.315	0.585274
H94	1.234519	5.292507	-0.48808
H95	3.493864	1.705805	-1.21118
H96	5.447337	2.718932	-0.12652
C97	-0.06893	3.303609	1.506533
H98	-0.32646	2.465495	2.160047
H99	1.010091	3.286186	1.365043
H100	-0.32412	4.229422	2.033062

C101	-2.14562	3.273673	-0.42767
H102	-2.43258	3.138174	-1.47336
H103	-2.62218	2.465125	0.129834
H104	-2.57044	4.220022	-0.07832
H105	-0.19455	4.154821	-0.67461
X	0.511818	2.053523	-0.77184

C3_ES_Chelbot_TS1

C1	-3.93912	-3.46052	-0.95048
C2	-1.68073	-4.389	-1.04942
C3	-2.95225	-4.19683	-1.91795
C4	-3.08363	-3.30895	0.328034
H5	-4.82802	-4.0651	-0.74251
H6	-2.74585	-3.62404	-2.82787
H7	-3.63021	-3.03743	1.231817
H8	-4.274	-2.49489	-1.3317
H9	-0.97927	-5.13821	-1.41719
H10	-3.34461	-5.16992	-2.22635
C11	-2.29073	-4.62743	0.349901
H12	-1.54949	-4.6759	1.153507
H13	-2.93011	-5.51374	0.388837
C14	0.353981	-2.98157	-0.28065
N15	0.943549	-1.91756	0.139939
C16	2.349481	-2.27218	0.560244
C17	2.313694	-3.83328	0.416056
P18	-1.94784	-0.68812	0.391717
Ir19	0.239724	0.115333	-0.21513
C20	-2.21427	-0.56727	2.200572
C21	-2.29716	-0.30982	4.999506
C22	-1.90103	-1.65609	3.025823
C23	-2.57703	0.652043	2.795097
C24	-2.61699	0.778198	4.182915
C25	-1.9442	-1.52758	4.415856
H26	-2.82507	1.510643	2.180522
H27	-2.89632	1.729658	4.626431
H28	-1.69671	-2.38114	5.040668
H29	-2.32663	-0.20886	6.080452
O30	1.029682	-4.129	-0.20866
C31	2.621108	-1.90086	2.014627
C32	1.604092	-1.46639	2.867621
C33	3.910489	-2.07663	2.538807
C34	1.875217	-1.16388	4.202957
H35	0.596151	-1.35583	2.495609
C36	4.181858	-1.78024	3.873324
H37	4.712657	-2.43006	1.896988

C101	-2.33603	2.996794	0.536178
H102	-2.99866	2.905767	-0.32561
H103	-2.46209	2.087424	1.120759
H104	-2.6946	3.829948	1.149624
H105	-0.79168	4.200818	-0.30435
H106	1.739713	0.822751	0.183774
H107	1.806645	0.475789	-0.59764

C3_ES_Chelbot_TS3

C1	-3.70265	-3.39788	-1.93728
C2	-1.41948	-4.22001	-1.6525
C3	-2.49522	-3.98019	-2.74619
C4	-3.11803	-3.30314	-0.51145
H5	-4.56159	-4.07722	-1.94773
H6	-2.1408	-3.29882	-3.52618
H7	-3.84532	-3.11669	0.279092
H8	-4.04179	-2.42755	-2.30007
H9	-0.62895	-4.9167	-1.93069
H10	-2.75553	-4.92527	-3.23072
C11	-2.2856	-4.59739	-0.4326
H12	-1.70877	-4.70864	0.489979
H13	-2.88261	-5.49938	-0.59397
C14	0.349895	-2.86209	-0.34533
N15	0.825786	-1.85013	0.302742
C16	2.117715	-2.27304	0.971746
C17	2.138079	-3.80774	0.630135
P18	-2.01136	-0.79277	0.319203
Ir19	0.218239	0.201783	-0.11591
C20	-2.17321	-1.15057	2.117368
C21	-2.3223	-1.65812	4.87935
C22	-2.21903	-2.46333	2.600717
C23	-2.17818	-0.0919	3.041904
C24	-2.26471	-0.34359	4.409907
C25	-2.28987	-2.71509	3.97166
H26	-2.09856	0.934774	2.701866
H27	-2.27583	0.487615	5.109484
H28	-2.31622	-3.74083	4.327993
H29	-2.37884	-1.85488	5.946002
O30	1.01236	-4.01344	-0.26408
C31	2.122689	-2.10584	2.489756
C32	1.16943	-1.38156	3.203055
C33	3.168383	-2.72118	3.200545
C34	1.266119	-1.25701	4.592397
H35	0.341678	-0.92258	2.686928
C36	3.260986	-2.60397	4.584802

C38	3.165914	-1.31024	4.708664	H37	3.933107	-3.27802	2.663294
H39	1.066866	-0.8164	4.840453	C38	2.309129	-1.8621	5.289641
H40	5.189204	-1.9116	4.258047	H39	0.508499	-0.68862	5.122132
H41	3.380371	-1.07008	5.746084	H40	4.080878	-3.08492	5.110691
C42	-3.47249	-0.03508	-0.39704	H41	2.381956	-1.76347	6.368644
C43	-4.66907	0.188969	0.296533	C42	-3.64008	-0.06587	-0.14136
C44	-3.45054	0.125701	-1.79218	C43	-4.60816	0.318435	0.794268
C45	-5.81378	0.599273	-0.39078	C44	-3.91598	0.092109	-1.50976
H46	-4.71571	0.047969	1.371117	C45	-5.82072	0.866333	0.371135
C47	-4.59679	0.525205	-2.47563	H46	-4.42836	0.188418	1.855508
C48	-5.78131	0.772946	-1.77492	C47	-5.13168	0.62797	-1.92929
H49	-6.73412	0.776015	0.158482	C48	-6.08528	1.025504	-0.9876
H50	-4.56919	0.637667	-3.55623	H49	-6.56228	1.158335	1.10906
H51	-6.6744	1.089229	-2.30586	H50	-5.34782	0.716476	-2.99094
H52	0.440795	0.470646	1.277764	H51	-7.03318	1.44295	-1.31421
H53	1.827948	0.267238	-0.41732	H52	0.404685	0.542426	1.380155
H54	-1.60126	-2.59925	2.581476	H53	1.381403	2.223882	-2.38209
H55	-2.54418	-0.09506	-2.34329	H54	-2.17864	-3.2981	1.914452
H56	3.09997	-4.23074	-0.22647	H55	-3.19568	-0.2427	-2.24929
C57	4.811202	-0.34089	-2.45531	H56	3.042282	-4.12477	0.109227
C58	3.973102	-1.41001	-2.7855	C57	5.415678	-0.30581	-1.062
C59	3.223371	-2.03928	-1.79597	C58	4.521045	-1.11792	-1.76489
C60	3.298979	-1.62224	-0.45796	C59	3.468644	-1.73484	-1.09447
C61	4.141095	-0.55073	-0.13912	C60	3.282982	-1.54653	0.284037
C62	4.89061	0.085869	-1.13135	C61	4.162905	-0.70735	0.972182
H63	5.395223	0.153347	-3.22671	C62	5.230726	-0.10332	0.30413
H64	3.898705	-1.74818	-3.81509	H63	6.246651	0.165637	-1.57884
H65	2.554201	-2.84802	-2.07919	H64	4.649095	-1.27736	-2.83165
H66	4.201935	-0.18647	0.877702	H65	2.782203	-2.36479	-1.65513
H67	5.526882	0.923674	-0.86101	H66	4.028656	-0.52938	2.032488
C68	-0.77662	3.269234	-0.11252	H67	5.917714	0.526824	0.861312
C69	-0.05998	2.129942	-0.85972	C68	-0.78431	3.197684	0.206473
C70	1.387426	1.900124	-0.77461	C69	-0.15936	2.199771	-0.81446
C71	-0.47046	3.197312	-3.18824	C70	1.036423	2.955678	-1.64312
C72	-0.37428	0.911093	-2.96299	C71	-1.8223	2.945431	-2.73113
C73	-0.91455	2.499704	-4.48285	C72	-0.88198	0.836466	-2.71686
H74	0.548165	3.605702	-3.25223	C73	-2.33998	2.100282	-3.91093
H75	-1.15282	3.993635	-2.8869	H74	-1.14477	3.737279	-3.0649
H76	-2.0002	2.54132	-4.61692	H75	-2.63483	3.399817	-2.16412
H77	-0.41388	2.85718	-5.38276	H76	-3.38517	1.813991	-3.78079
N78	-0.51008	2.060421	-2.26151	H77	-2.20122	2.572888	-4.88378
O79	-0.54108	1.107856	-4.27999	N78	-1.11895	1.919991	-1.93939
O80	-0.1299	-0.21074	-2.48836	O79	-1.53912	0.887129	-3.88226
H81	2.329541	-4.33606	1.38428	O80	-0.16315	-0.12451	-2.4048
N82	-1.97318	-2.34877	0.07771	H81	1.971845	-4.43048	1.509982

C83	-1.03473	-2.99457	-0.87061
H84	-0.9708	-2.4579	-1.8265
H85	1.855524	1.751697	-1.75075
C86	4.155679	4.045976	1.72781
C87	3.755381	4.539545	0.484603
C88	2.85456	3.815572	-0.29483
C89	2.332918	2.595239	0.158598
C90	2.743471	2.105408	1.403216
C91	3.648534	2.82725	2.181093
H92	4.859471	4.607171	2.335643
H93	4.145381	5.485777	0.120094
H94	2.550732	4.201207	-1.2661
H95	2.350579	1.162868	1.765479
H96	3.955959	2.431117	3.144755
C97	-0.46543	3.355589	1.389859
H98	-0.66727	2.403298	1.885921
H99	0.570257	3.624309	1.587866
H100	-1.10109	4.121628	1.846742
C101	-2.29977	3.232371	-0.3164
H102	-2.59912	3.142018	-1.36368
H103	-2.74336	2.384278	0.206604
H104	-2.74903	4.146284	0.085089
H105	-0.40041	4.201564	-0.56716
X	0.511818	2.053523	-0.77184

C3_ES_Chelbot_int1

C1	-3.91123	-3.50203	-0.99682
C2	-1.6571	-4.44651	-1.00624
C3	-2.90812	-4.2842	-1.91053
C4	-3.08915	-3.31582	0.298042
H5	-4.81524	-4.08571	-0.79452
H6	-2.67865	-3.75283	-2.83965
H7	-3.65869	-3.01239	1.177278
H8	-4.2189	-2.54299	-1.41745
H9	-0.94828	-5.20843	-1.33104
H10	-3.30062	-5.26744	-2.1847
C11	-2.30239	-4.63589	0.384183
H12	-1.58123	-4.66096	1.207046
H13	-2.94635	-5.51823	0.436763
C14	0.377909	-3.00157	-0.27548
N15	0.930582	-1.9249	0.160199
C16	2.3488	-2.226	0.544272
C17	2.389129	-3.7848	0.344292
P18	-1.91029	-0.72241	0.40241
Ir19	0.105397	0.081757	-0.20228

N82	-2.04307	-2.26408	-0.50943
C83	-0.87777	-2.83401	-1.22635
H84	-0.61562	-2.22713	-2.09898
H85	0.548282	3.770826	-2.18092
C86	4.450712	4.65706	0.382524
C87	3.288276	5.410707	0.208427
C88	2.189689	4.854968	-0.44238
C89	2.227351	3.537071	-0.92249
C90	3.403644	2.79506	-0.75472
C91	4.506287	3.35222	-0.10558
H92	5.311831	5.090509	0.882878
H93	3.24188	6.433397	0.571413
H94	1.292342	5.452421	-0.58505
H95	3.469052	1.783275	-1.14562
H96	5.411688	2.764719	0.006815
C97	0.017014	3.294268	1.521415
H98	-0.20496	2.438105	2.164083
H99	1.095802	3.323015	1.370755
H100	-0.26978	4.204365	2.057732
C101	-2.24939	2.922729	0.565286
H102	-2.92111	2.838773	-0.28938
H103	-2.33625	1.991034	1.119844
H104	-2.62221	3.725831	1.209351
H105	-0.74317	4.184796	-0.27785
H106	1.765166	0.539234	-0.26164
H107	0.924033	1.60068	-0.38607

C3_ES_Chelbot_PROD

C1	-3.73433	-3.55763	-1.69487
C2	-1.43563	-4.35964	-1.54081
C3	-2.57349	-4.13509	-2.57037
C4	-3.08564	-3.49382	-0.29419
H5	-4.5989	-4.22958	-1.67685
H6	-2.27101	-3.45814	-3.37569
H7	-3.78632	-3.33831	0.526116
H8	-4.08259	-2.57843	-2.02384
H9	-0.64549	-5.03535	-1.86818
H10	-2.85425	-5.08605	-3.03158
C11	-2.22811	-4.77192	-0.27856
H12	-1.60789	-4.87147	0.617247
H13	-2.80946	-5.68654	-0.42582
C14	0.390649	-2.97744	-0.35521
N15	0.939	-1.93696	0.185548
C16	2.262718	-2.33553	0.814969
C17	2.174123	-3.89096	0.658129

C20	-2.16375	-0.61926	2.209298
C21	-2.28074	-0.39528	5.001425
C22	-1.92687	-1.73676	3.019722
C23	-2.45175	0.616348	2.811423
C24	-2.51167	0.724981	4.198476
C25	-1.98902	-1.62416	4.409958
H26	-2.62093	1.497405	2.201372
H27	-2.73448	1.685718	4.653539
H28	-1.80183	-2.49729	5.028376
H29	-2.32698	-0.30802	6.083131
O30	1.104072	-4.12179	-0.25827
C31	2.62649	-1.88796	2.004646
C32	1.619179	-1.4455	2.865453
C33	3.911336	-2.10609	2.524482
C34	1.896373	-1.18626	4.209149
H35	0.615538	-1.29965	2.493191
C36	4.188486	-1.84912	3.865446
H37	4.704512	-2.46476	1.873983
C38	3.181969	-1.37881	4.711978
H39	1.095943	-0.83796	4.856143
H40	5.191433	-2.01514	4.24805
H41	3.399846	-1.17427	5.756247
C42	-3.41111	-0.01333	-0.37431
C43	-4.5685	0.319822	0.340854
C44	-3.4101	0.100601	-1.77453
C45	-5.69601	0.792948	-0.33354
H46	-4.60122	0.211096	1.419419
C47	-4.53867	0.5666	-2.44254
C48	-5.68176	0.924306	-1.72127
H49	-6.58731	1.054037	0.229721
H50	-4.53133	0.645626	-3.52623
H51	-6.56117	1.292364	-2.24175
H52	0.28258	0.492499	1.285983
H53	1.856497	0.773653	-0.62434
H54	-1.6708	-2.68836	2.567142
H55	-2.53356	-0.20047	-2.33694
H56	3.175331	-4.11568	-0.33556
C57	4.690465	-0.11278	-2.44757
C58	3.809906	-1.1367	-2.80935
C59	3.096032	-1.81886	-1.82894
C60	3.247305	-1.49875	-0.46958
C61	4.130568	-0.47307	-0.1195
C62	4.846672	0.215223	-1.10265
H63	5.24829	0.422733	-3.21059
H64	3.67583	-1.40011	-3.85461

P18	-1.98692	-0.92546	0.430301
Ir19	0.230896	-0.04724	-0.0847
C20	-2.24544	-1.14402	2.231037
C21	-2.52222	-1.34378	5.01738
C22	-2.46055	-2.38951	2.834967
C23	-2.13315	-0.00302	3.043886
C24	-2.28226	-0.10047	4.425595
C25	-2.597	-2.48663	4.221317
H26	-1.91215	0.962815	2.597492
H27	-2.19833	0.790215	5.0417
H28	-2.75931	-3.45873	4.677956
H29	-2.63444	-1.42148	6.09497
O30	1.034369	-4.13384	-0.21612
C31	2.342458	-2.04144	2.31035
C32	1.197574	-1.80554	3.075817
C33	3.574476	-2.19037	2.964853
C34	1.287298	-1.66414	4.461423
H35	0.231104	-1.72666	2.599794
C36	3.662466	-2.05701	4.349477
H37	4.471219	-2.40478	2.391472
C38	2.51962	-1.78261	5.102208
H39	0.384658	-1.46636	5.031168
H40	4.626095	-2.1675	4.838564
H41	2.590025	-1.67212	6.180605
C42	-3.54673	-0.14124	-0.15392
C43	-4.53415	0.366815	0.699986
C44	-3.72948	-0.04193	-1.54383
C45	-5.67703	0.973485	0.173825
H46	-4.41996	0.289834	1.776223
C47	-4.87707	0.551679	-2.06492
C48	-5.85196	1.068253	-1.20608
H49	-6.43606	1.361468	0.847098
H50	-5.02023	0.593205	-3.14155
H51	-6.74763	1.529717	-1.61172
H52	0.630508	0.168779	1.395744
H53	1.563305	2.443327	-2.10695
H54	-2.49564	-3.29131	2.235925
H55	-2.98453	-0.45057	-2.21861
H56	3.05816	-4.3361	0.202291
C57	5.30449	-0.535	-1.70109
C58	4.584077	-1.64689	-2.13996
C59	3.629363	-2.23129	-1.30987
C60	3.379142	-1.7182	-0.03067
C61	4.089945	-0.589	0.39049
C62	5.051859	-0.00753	-0.43532

H65	2.397125	-2.59413	-2.13176
H66	4.254757	-0.18698	0.916668
H67	5.515982	1.018031	-0.80794
C68	-0.63392	3.263257	-0.21128
C69	0.017474	2.036464	-0.88228
C70	1.574521	1.873042	-0.85507
C71	-0.33149	3.101839	-3.25724
C72	-0.31878	0.82166	-2.97616
C73	-0.77425	2.385102	-4.54425
H74	0.693534	3.494035	-3.31968
H75	-1.00284	3.916639	-2.98185
H76	-1.85384	2.466057	-4.70556
H77	-0.24025	2.698467	-5.44141
N78	-0.40244	1.988372	-2.30547
O79	-0.46358	0.98327	-4.29933
O80	-0.13061	-0.2982	-2.46338
H81	2.459064	-4.32137	1.291749
N82	-1.96971	-2.36842	0.039461
C83	-1.01199	-3.04607	-0.86404
H84	-0.94098	-2.54627	-1.83945
H85	1.96699	1.939305	-1.87608
C86	4.076681	4.215674	1.755303
C87	3.727275	4.689898	0.489913
C88	2.917367	3.919772	-0.34499
C89	2.440812	2.675027	0.079826
C90	2.797315	2.203229	1.348203
C91	3.610594	2.969734	2.181086
H92	4.711037	4.812732	2.403844
H93	4.088653	5.656452	0.15044
H94	2.654171	4.28865	-1.33432
H95	2.424397	1.240989	1.686402
H96	3.880096	2.591899	3.163317
C97	-0.35325	3.419922	1.292897
H98	-0.57019	2.491204	1.826491
H99	0.67641	3.697727	1.508352
H100	-0.99846	4.206449	1.699437
C101	-2.15877	3.262884	-0.42543
H102	-2.45913	3.098405	-1.46323
H103	-2.62534	2.467864	0.159801
H104	-2.58328	4.216327	-0.09539
H105	-0.2192	4.157279	-0.70763
X	0.511818	2.053523	-0.77184

C3_ES_Chelbot_TS2

C1	-3.68141	-3.36122	-1.85634

H63	6.053466	-0.08075	-2.34334
H64	4.765954	-2.06096	-3.12749
H65	3.065622	-3.08448	-1.67971
H66	3.882648	-0.15165	1.360277
H67	5.592166	0.870175	-0.09334
C68	-0.91161	3.675132	0.098767
C69	-0.17357	2.762491	-0.91317
C70	1.171292	3.269402	-1.50383
C71	-1.5966	3.402589	-2.94597
C72	-0.99733	1.182798	-2.67897
C73	-2.37106	2.506183	-3.91871
H74	-0.80377	3.969588	-3.44755
H75	-2.24378	4.099869	-2.41104
H76	-3.40764	2.363013	-3.6015
H77	-2.33329	2.83839	-4.95617
N78	-1.03461	2.387237	-2.04415
O79	-1.69796	1.224172	-3.82733
O80	-0.45393	0.136854	-2.31161
H81	1.958406	-4.37788	1.609781
N82	-2.0151	-2.45713	-0.2842
C83	-0.9046	-2.96258	-1.13197
H84	-0.74158	-2.32223	-2.00847
H85	0.916737	4.079286	-2.19702
C86	4.232804	4.722049	1.173748
C87	3.426933	5.621739	0.474416
C88	2.440704	5.147983	-0.39185
C89	2.242928	3.772572	-0.56617
C90	3.065972	2.877833	0.12773
C91	4.050319	3.349026	0.994673
H92	5.001354	5.089317	1.847873
H93	3.568295	6.691586	0.599562
H94	1.817575	5.855286	-0.93549
H95	2.925945	1.812569	-0.0083
H96	4.677653	2.642074	1.531606
C97	-0.20241	3.694756	1.466703
H98	-0.14149	2.676294	1.870312
H99	0.806861	4.098821	1.423916
H100	-0.78145	4.30264	2.168627
C101	-2.36666	3.232732	0.314484
H102	-2.97543	3.221295	-0.59205
H103	-2.39219	2.219123	0.722088
H104	-2.8542	3.894846	1.036267
H105	-0.89449	4.692923	-0.31959
H106	1.728182	0.357927	-0.43177
H107	0.063298	1.836492	-0.30471

C2	-1.41707	-4.24234	-1.59908
C3	-2.50391	-3.98486	-2.67772
C4	-3.07329	-3.26947	-0.44108
H5	-4.55861	-4.01679	-1.84497
H6	-2.1444	-3.32141	-3.47057
H7	-3.78195	-3.05939	0.360619
H8	-4.00132	-2.38679	-2.22501
H9	-0.64903	-4.96182	-1.88241
H10	-2.79846	-4.92719	-3.14771
C11	-2.27231	-4.58392	-0.36166
H12	-1.68488	-4.69972	0.553866
H13	-2.89671	-5.47054	-0.50296
C14	0.417925	-2.91322	-0.34543
N15	0.88584	-1.91264	0.319402
C16	2.202731	-2.31759	0.923178
C17	2.265812	-3.84063	0.520313
P18	-1.92274	-0.78408	0.343949
Ir19	0.062728	0.156356	-0.04692
C20	-2.09303	-1.09834	2.144596
C21	-2.22985	-1.55747	4.910269
C22	-2.1031	-2.4036	2.648373
C23	-2.13109	-0.02038	3.045761
C24	-2.20773	-0.24941	4.417352
C25	-2.17082	-2.63091	4.023895
H26	-2.08073	0.999683	2.681054
H27	-2.23929	0.593437	5.101976
H28	-2.16779	-3.64974	4.400145
H29	-2.28068	-1.7358	5.980529
O30	1.117307	-4.04767	-0.3441
C31	2.242126	-2.18526	2.443986
C32	1.334746	-1.41439	3.171472
C33	3.246284	-2.87534	3.144246
C34	1.433469	-1.32327	4.562303
H35	0.54057	-0.89224	2.660654
C36	3.34523	-2.78597	4.531214
H37	3.973017	-3.47598	2.601628
C38	2.437504	-2.00343	5.24913
H39	0.708485	-0.72254	5.102736
H40	4.133416	-3.32533	5.048895
H41	2.513279	-1.93085	6.330108
C42	-3.50423	-0.0196	-0.17477
C43	-4.49135	0.388088	0.731878
C44	-3.74422	0.107368	-1.55295
C45	-5.6883	0.93533	0.267541
H46	-4.33865	0.277159	1.799394

C47	-4.94123	0.650187	-2.01233
C48	-5.91373	1.073702	-1.1015
H49	-6.44729	1.245734	0.979713
H50	-5.12692	0.723636	-3.08054
H51	-6.84898	1.494518	-1.45941
H52	0.090123	0.535517	1.452153
H53	1.68063	1.536088	-1.6961
H54	-2.03461	-3.24846	1.97655
H55	-3.01304	-0.2551	-2.26649
H56	3.161687	-4.09909	-0.0458
C57	5.401829	-0.27969	-1.21734
C58	4.309498	-0.81866	-1.90057
C59	3.285492	-1.44798	-1.19439
C60	3.33006	-1.54892	0.205333
C61	4.4192	-0.98926	0.882717
C62	5.448519	-0.36409	0.174061
H63	6.202386	0.209305	-1.76485
H64	4.25387	-0.75116	-2.98344
H65	2.443864	-1.85735	-1.74516
H66	4.469037	-1.02971	1.963985
H67	6.287324	0.062078	0.71722
C68	-0.80057	3.225367	0.180761
C69	-0.2314	2.124171	-0.73929
C70	1.201209	2.479946	-1.3985
C71	-1.61469	3.077921	-2.7763
C72	-0.87481	0.902571	-2.72411
C73	-2.13317	2.292979	-3.99522
H74	-0.81385	3.782326	-3.03905
H75	-2.40718	3.630749	-2.27011
H76	-3.2028	2.081635	-3.91684
H77	-1.91529	2.76275	-4.95474
N78	-1.10288	1.981173	-1.94733
O79	-1.42365	1.022016	-3.94354
O80	-0.24517	-0.11477	-2.37816
H81	2.152202	-4.50693	1.376643
N82	-1.96406	-2.26436	-0.45809
C83	-0.82963	-2.86834	-1.19528
H84	-0.57715	-2.27286	-2.07913
H85	0.958414	2.95414	-2.35696
C86	4.296773	5.040328	0.219116
C87	3.053652	5.574235	-0.1285
C88	2.051378	4.746211	-0.62778
C89	2.255968	3.364185	-0.7693
C90	3.520911	2.852047	-0.45424
C91	4.529693	3.677104	0.043164

H92	5.080128	5.685813	0.605966
H93	2.868958	6.639488	-0.02132
H94	1.09917	5.18237	-0.91658
H95	3.735915	1.800817	-0.61713
H96	5.500404	3.250865	0.28218
C97	-0.04282	3.345864	1.516131
H98	-0.30181	2.511761	2.175149
H99	1.039649	3.351101	1.394268
H100	-0.32492	4.274808	2.022359
C101	-2.29557	3.067988	0.491871
H102	-2.92452	2.922411	-0.38873
H103	-2.46053	2.204492	1.135819
H104	-2.65844	3.95187	1.026783
H105	-0.68006	4.182286	-0.3507
H106	1.991109	1.051144	1.195641
H107	2.467578	0.840205	0.659932

C3_ER_Cheltop

C3_ER_Cheltop_SM

C1	-4.24858	-3.40287	0.876158
C2	-2.19851	-4.54636	0.222072
C3	-3.70397	-4.46459	-0.13559
C4	-2.98079	-3.03809	1.685035
H5	-5.00531	-3.82988	1.54224
H6	-3.86632	-4.17636	-1.17917
H7	-3.16654	-2.51518	2.623062
H8	-4.69005	-2.53279	0.388719
H9	-1.67552	-5.41833	-0.17013
H10	-4.17723	-5.4395	0.011934
C11	-2.23552	-4.38247	1.758077
H12	-1.24827	-4.30122	2.222813
H13	-2.81224	-5.17038	2.250566
C14	-0.04929	-3.19441	-0.3028
N15	0.663073	-2.12554	-0.36302
C16	2.108723	-2.53303	-0.27201
C17	1.994708	-4.06112	-0.5708
P18	-1.92685	-0.59942	0.626944
Ir19	-0.12601	-0.07979	-0.60076
C20	-1.77477	0.092013	2.312401
C21	-1.26204	1.281777	4.802089
C22	-1.28723	-0.67666	3.377904
C23	-1.99657	1.465526	2.503436
C24	-1.74324	2.053451	3.741555
C25	-1.03854	-0.08306	4.616192
H26	-2.35908	2.076643	1.684825

C3_ER_Cheltop_int2

C1	-3.64301	-3.342	-1.96871
C2	-1.38485	-4.21093	-1.64029
C3	-2.43784	-3.95995	-2.75276
C4	-3.07706	-3.23895	-0.53632
H5	-4.51478	-4.00482	-1.97916
H6	-2.05735	-3.29438	-3.53389
H7	-3.81167	-3.02735	0.241043
H8	-3.95996	-2.37275	-2.3521
H9	-0.60686	-4.92936	-1.89761
H10	-2.71222	-4.90405	-3.23129
C11	-2.27591	-4.5511	-0.42848
H12	-1.71426	-4.66434	0.503192
H13	-2.89355	-5.43976	-0.58551
C14	0.404818	-2.89054	-0.32633
N15	0.87795	-1.88895	0.332738
C16	2.162049	-2.31954	0.997783
C17	2.190737	-3.85033	0.634477
P18	-1.9584	-0.74854	0.288742
Ir19	0.164935	0.204216	-0.13281
C20	-2.10105	-1.0866	2.091348
C21	-2.24105	-1.58468	4.852575
C22	-2.1699	-2.39773	2.575872
C23	-2.08061	-0.02465	3.011334
C24	-2.16112	-0.27208	4.379964
C25	-2.23653	-2.64456	3.948152
H26	-1.98644	0.998335	2.665624

H27	-1.92598	3.115739	3.879585	H27	-2.14932	0.56071	5.077464
H28	-0.66799	-0.69078	5.436761	H28	-2.2776	-3.66877	4.307221
H29	-1.06391	1.741177	5.765789	H29	-2.29267	-1.77798	5.92009
O30	0.59354	-4.36538	-0.31403	O30	1.068448	-4.04604	-0.26674
C31	2.622259	-2.35892	1.16244	C31	2.173263	-2.16289	2.516515
C32	1.812878	-1.89252	2.195652	C32	1.238365	-1.41497	3.228407
C33	3.940743	-2.74288	1.453906	C33	3.20723	-2.80017	3.223892
C34	2.312431	-1.78603	3.495098	C34	1.337898	-1.29482	4.618064
H35	0.80116	-1.58235	1.985305	H35	0.430534	-0.92738	2.707074
C36	4.438046	-2.64467	2.750236	C36	3.303741	-2.68547	4.607849
H37	4.586342	-3.10323	0.656334	H37	3.960922	-3.37007	2.684816
C38	3.623435	-2.16002	3.777756	C38	2.367035	-1.92532	5.313566
H39	1.670324	-1.39544	4.279544	H39	0.59684	-0.70666	5.149342
H40	5.462377	-2.94024	2.95835	H40	4.114522	-3.18221	5.133048
H41	4.013202	-2.07532	4.788103	H41	2.44303	-1.82865	6.392534
C42	-3.58158	-0.12243	0.003396	C42	-3.59498	-0.03835	-0.16662
C43	-4.60661	0.330204	0.847351	C43	-4.55131	0.333242	0.787611
C44	-3.87987	-0.40833	-1.33952	C44	-3.9035	0.107644	-1.52884
C45	-5.89822	0.513382	0.348975	C45	-5.78061	0.861629	0.388538
H46	-4.40612	0.53439	1.893808	H46	-4.34809	0.212828	1.845716
C47	-5.1718	-0.23345	-1.83025	C47	-5.13497	0.626976	-1.92358
C48	-6.18519	0.232727	-0.98783	C48	-6.07584	1.015932	-0.96532
H49	-6.68165	0.870797	1.010891	H49	-6.5099	1.144413	1.142094
H50	-5.38639	-0.45527	-2.87162	H50	-5.37182	0.709254	-2.98109
H51	-7.19122	0.373969	-1.37124	H51	-7.03563	1.419723	-1.2735
H52	-0.8981	-0.54383	-1.88353	H52	0.247393	0.567546	1.376663
H53	1.409335	0.461057	-1.67983	H53	1.342812	2.041705	-2.28691
H54	-1.08557	-1.7332	3.239491	H54	-2.14988	-3.23434	1.891416
H55	-3.09717	-0.76644	-2.00083	H55	-3.19864	-0.22024	-2.28441
H56	2.204227	-4.33156	-1.60787	H56	3.098247	-4.15753	0.113064
C57	4.364205	-0.28039	-3.22523	C57	5.450083	-0.28326	-0.98842
C58	3.541895	-1.33511	-3.62218	C58	4.573699	-1.10012	-1.70844
C59	2.824133	-2.05997	-2.67248	C59	3.523591	-1.74052	-1.05405
C60	2.929988	-1.76078	-1.30622	C60	3.325286	-1.57287	0.325557
C61	3.744627	-0.68763	-0.92082	C61	4.185331	-0.72515	1.030878
C62	4.455947	0.044947	-1.87256	C62	5.248825	-0.09694	0.377753
H63	4.916825	0.289559	-3.96607	H63	6.277528	0.207292	-1.49292
H64	3.444642	-1.58297	-4.67476	H64	4.713525	-1.24508	-2.77584
H65	2.163517	-2.85163	-3.01334	H65	2.854014	-2.37803	-1.62674
H66	3.819095	-0.4187	0.126063	H66	4.041203	-0.56239	2.091621
H67	5.086296	0.869622	-1.55136	H67	5.918106	0.542018	0.945789
C68	-1.24031	2.787857	-1.78502	C68	-0.8588	3.214511	0.184472
C69	-0.16018	1.953707	-1.06278	C69	-0.25795	2.195762	-0.81744
C70	1.14421	1.571587	-1.84497	C70	0.991321	2.808932	-1.5869
C71	0.681335	4.089993	0.21131	C71	-1.87967	3.005975	-2.76986

C72	0.933303	1.996982	1.109036
C73	1.157755	4.170013	1.670497
H74	-0.13753	4.77855	-0.00167
H75	1.493071	4.27488	-0.50732
H76	2.027916	4.807761	1.826996
H77	0.350068	4.465143	2.347887
N78	0.255094	2.685692	0.162502
O79	1.534553	2.802234	1.994194
O80	1.03332	0.760221	1.157098
H81	2.598643	-4.67009	0.098754
N82	-2.01218	-2.27816	0.841469
C83	-1.55239	-3.20774	-0.2213
H84	-1.91945	-2.91651	-1.21669
C85	1.429962	2.168658	-6.10242
C86	1.67575	3.221285	-5.21928
C87	1.583128	3.014114	-3.84295
C88	1.236783	1.756973	-3.33585
C89	0.999274	0.705823	-4.22574
C90	1.094105	0.909324	-5.60132
H91	1.5033	2.328487	-7.17422
H92	1.942904	4.202407	-5.60153
H93	1.782758	3.835665	-3.15807
H94	0.732758	-0.26941	-3.83108
H95	0.90559	0.08489	-6.28344
H96	2.008898	2.037484	-1.36006
C97	-2.37539	3.202775	-0.8314
H98	-2.0229	3.684175	0.084488
H99	-3.04764	3.903648	-1.33642
H100	-2.96752	2.33007	-0.54796
C101	-1.87503	2.125358	-3.01609
H102	-1.16726	1.954688	-3.82284
H103	-2.32138	1.170245	-2.73907
H104	-2.6719	2.769437	-3.40286
H105	-0.74097	3.706022	-2.13749
X	0.335144	1.892742	-1.24452

C3_ER_Cheltop_TS1

C1	-4.23697	-3.40379	1.037604
C2	-2.21525	-4.53811	0.282813
C3	-3.7345	-4.45052	-0.01029
C4	-2.93366	-3.03925	1.788053
H5	-4.95871	-3.84224	1.734542
H6	-3.93999	-4.14694	-1.04179
H7	-3.07343	-2.52002	2.736178
H8	-4.7069	-2.53277	0.579937

C72	-0.99679	0.882997	-2.71721
C73	-2.39057	2.167577	-3.95803
H74	-1.17039	3.779501	-3.08386
H75	-2.69378	3.484488	-2.22395
H76	-3.44511	1.904876	-3.85014
H77	-2.21992	2.628417	-4.9315
N78	-1.21933	1.971741	-1.95805
O79	-1.61939	0.93375	-3.9033
O80	-0.3115	-0.10484	-2.38448
H81	2.024153	-4.48548	1.505407
N82	-1.97523	-2.22442	-0.52942
C83	-0.81266	-2.83566	-1.22049
H84	-0.52645	-2.2433	-2.09527
H85	0.593505	3.619824	-2.20985
C86	4.451846	4.573964	0.330182
C87	3.261144	5.295305	0.214795
C88	2.150006	4.714793	-0.38973
C89	2.189726	3.397545	-0.87533
C90	3.399925	2.698549	-0.77949
C91	4.51859	3.278304	-0.1774
H92	5.323446	5.027757	0.793108
H93	3.203195	6.315	0.585274
H94	1.234519	5.292507	-0.48808
H95	3.493864	1.705805	-1.21118
H96	5.447337	2.718932	-0.12652
C97	-0.06893	3.303609	1.506533
H98	-0.32646	2.465495	2.160047
H99	1.010091	3.286186	1.365043
H100	-0.32412	4.229422	2.033062
C101	-2.33603	2.996794	0.536178
H102	-2.99866	2.905767	-0.32561
H103	-2.46209	2.087424	1.120759
H104	-2.6946	3.829948	1.149624
H105	-0.79168	4.200818	-0.30435
H106	1.739713	0.822751	0.183774
H107	1.806645	0.475789	-0.59764

C3_ER_Cheltop_TS3

C1	-4.17391	-3.53776	1.247801
C2	-2.1636	-4.5345	0.299761
C3	-3.70167	-4.50714	0.116194
C4	-2.83619	-3.12007	1.903142
H5	-4.79723	-4.0525	1.986577
H6	-3.99192	-4.16217	-0.88136
H7	-2.93018	-2.64016	2.876748

H9	-1.71352	-5.40991	-0.13599
H10	-4.20183	-5.42745	0.142464
C11	-2.18494	-4.38295	1.82002
H12	-1.17844	-4.30363	2.241419
H13	-2.73946	-5.17339	2.333472
C14	-0.0814	-3.18831	-0.30531
N15	0.643368	-2.13224	-0.43258
C16	2.090921	-2.55746	-0.38447
C17	1.950272	-4.10526	-0.55063
P18	-1.9657	-0.60077	0.636026
Ir19	-0.0877	-0.05098	-0.60624
C20	-1.87383	0.132562	2.307403
C21	-1.42639	1.390446	4.773565
C22	-1.25744	-0.56303	3.357272
C23	-2.24852	1.47126	2.500243
C24	-2.02879	2.093043	3.728476
C25	-1.04127	0.06256	4.584289
H26	-2.71134	2.029504	1.695049
H27	-2.33575	3.125749	3.871008
H28	-0.57086	-0.48897	5.393277
H29	-1.25641	1.875971	5.73006
O30	0.546699	-4.3669	-0.27301
C31	2.680056	-2.28384	1.00556
C32	1.884968	-1.89087	2.080368
C33	4.045358	-2.53309	1.224749
C34	2.441624	-1.71512	3.348114
H35	0.835585	-1.68915	1.925743
C36	4.59977	-2.36339	2.490416
H37	4.676775	-2.84811	0.398189
C38	3.798395	-1.9465	3.557258
H39	1.808562	-1.38029	4.165074
H40	5.658517	-2.55141	2.643984
H41	4.233734	-1.80462	4.542261
C42	-3.61124	-0.15826	-0.03348
C43	-4.69075	0.199727	0.789577
C44	-3.83181	-0.34322	-1.40733
C45	-5.95668	0.400633	0.239718
H46	-4.54959	0.319936	1.858643
C47	-5.10061	-0.15226	-1.95152
C48	-6.16365	0.228655	-1.13025
H49	-6.7827	0.688323	0.883795
H50	-5.25682	-0.29101	-3.01734
H51	-7.15035	0.387963	-1.55524
H52	-0.80705	-0.47018	-1.92904
H53	1.251525	0.226844	-1.45559

H8	-4.74112	-2.68715	0.873269
H9	-1.65632	-5.36863	-0.18411
H10	-4.11283	-5.51197	0.248048
C11	-2.02579	-4.42616	1.835053
H12	-0.99492	-4.31405	2.184016
H13	-2.504	-5.25724	2.360976
C14	-0.12996	-3.08624	-0.34417
N15	0.558306	-2.00326	-0.46557
C16	2.024689	-2.37885	-0.45344
C17	1.92601	-3.9284	-0.65016
P18	-2.08363	-0.59104	0.763588
Ir19	-0.20852	0.007456	-0.70942
C20	-1.93811	0.058438	2.469403
C21	-1.52665	1.140584	5.028787
C22	-1.2344	-0.66373	3.445348
C23	-2.42027	1.338387	2.786728
C24	-2.22747	1.866176	4.063956
C25	-1.02797	-0.12369	4.713677
H26	-2.95392	1.92142	2.043852
H27	-2.63462	2.843955	4.308701
H28	-0.48389	-0.69688	5.458863
H29	-1.37429	1.556237	6.020518
O30	0.535733	-4.23937	-0.35949
C31	2.623819	-2.12287	0.936338
C32	1.853023	-1.683	2.01034
C33	3.970043	-2.45173	1.167851
C34	2.415396	-1.52924	3.278408
H35	0.814834	-1.43705	1.859354
C36	4.532551	-2.30494	2.433098
H37	4.586802	-2.81657	0.350896
C38	3.756133	-1.83469	3.49549
H39	1.796188	-1.15936	4.090675
H40	5.57764	-2.55715	2.588886
H41	4.195968	-1.71371	4.481261
C42	-3.81763	-0.28998	0.234054
C43	-4.86586	-0.00383	1.121163
C44	-4.11651	-0.55465	-1.11309
C45	-6.18303	0.038101	0.661658
H46	-4.66377	0.176963	2.171543
C47	-5.4346	-0.52498	-1.56506
C48	-6.47248	-0.22529	-0.67875
H49	-6.98544	0.266988	1.357191
H50	-5.65058	-0.72894	-2.6098
H51	-7.49947	-0.19702	-1.0302
H52	-1.02025	-0.54962	-1.92828

H54	-0.94196	-1.591	3.215367	H53	1.72159	2.527477	-1.16878
H55	-3.00516	-0.62808	-2.05025	H54	-0.8435	-1.64797	3.21409
H56	2.159778	-4.47268	-1.55661	H55	-3.31237	-0.77063	-1.81049
C57	4.265378	-0.62228	-3.61509	H56	2.134075	-4.25923	-1.66958
C58	3.4549	-1.72636	-3.87634	C57	4.141677	-0.59936	-3.80512
C59	2.752238	-2.34205	-2.84157	C58	3.091771	-1.49604	-4.01554
C60	2.868658	-1.88696	-1.52082	C59	2.413107	-2.04204	-2.92887
C61	3.663171	-0.75735	-1.27609	C60	2.772866	-1.71436	-1.6126
C62	4.35317	-0.13035	-2.31377	C61	3.78943	-0.77264	-1.41492
H63	4.805531	-0.13706	-4.42229	C62	4.474126	-0.22692	-2.50331
H64	3.356692	-2.10313	-4.89021	H63	4.681405	-0.17975	-4.64905
H65	2.103388	-3.1785	-3.08312	H64	2.801152	-1.77137	-5.02519
H66	3.744371	-0.36799	-0.26724	H65	1.584362	-2.72014	-3.1136
H67	4.965157	0.741514	-2.10039	H66	4.057208	-0.46601	-0.41082
C68	-1.21423	2.858865	-1.70985	H67	5.26941	0.491987	-2.32837
C69	-0.14824	2.030781	-0.9646	C68	-1.74472	2.884943	-1.57412
C70	1.110848	1.5549	-1.65304	C69	-0.4359	2.338554	-0.955
C71	0.58543	4.109454	0.445775	C70	0.844195	2.987513	-1.63784
C72	0.819649	1.980157	1.269154	C71	-0.27929	4.129116	0.97043
C73	0.942734	4.131703	1.940644	C72	0.509064	1.992303	1.285203
H74	-0.23169	4.789703	0.200734	C73	0.183688	3.918564	2.421509
H75	1.445765	4.342036	-0.19867	H74	-1.2642	4.59254	0.909279
H76	1.78012	4.781681	2.19478	H75	0.429755	4.743695	0.406546
H77	0.075272	4.374053	2.562758	H76	0.887585	4.673435	2.773308
N78	0.196579	2.700948	0.305887	H77	-0.65339	3.831909	3.117806
O79	1.329242	2.759828	2.232073	N78	-0.29775	2.733756	0.483452
O80	0.947665	0.747029	1.2716	O79	0.867881	2.639222	2.398083
H81	2.544921	-4.65831	0.174872	O80	0.894917	0.838256	1.051822
N82	-2.00716	-2.27384	0.904676	H81	2.546666	-4.48105	0.053274
C83	-1.58403	-3.19817	-0.17796	N82	-2.01352	-2.27862	0.981213
H84	-1.98428	-2.90141	-1.1587	C83	-1.62168	-3.15344	-0.15296
C85	1.726606	2.093438	-5.87365	H84	-2.08381	-2.84674	-1.10204
C86	1.975347	3.128744	-4.96989	C85	1.207018	2.861139	-5.95217
C87	1.782523	2.922795	-3.60511	C86	0.571408	3.93651	-5.3266
C88	1.334152	1.682992	-3.13131	C87	0.469592	3.978625	-3.93687
C89	1.094321	0.649345	-4.03786	C88	0.990167	2.945631	-3.14357
C90	1.290546	0.85443	-5.40304	C89	1.654616	1.889966	-3.78082
H91	1.87403	2.252987	-6.93777	C90	1.757587	1.84405	-5.17149
H92	2.317142	4.095709	-5.32771	H91	1.286373	2.827794	-7.03479
H93	1.975725	3.730972	-2.90259	H92	0.158827	4.746536	-5.9215
H94	0.757907	-0.31303	-3.66846	H93	-0.02145	4.824981	-3.46217
H95	1.101824	0.043332	-6.10058	H94	2.117976	1.108381	-3.19184
H96	2.01225	1.837107	-1.10037	H95	2.280251	1.014164	-5.6382
C97	-2.41007	3.197392	-0.8025	H96	0.845648	4.043328	-1.34881
H98	-2.11585	3.624467	0.159458	C97	-2.951	2.928912	-0.6296

H99	-3.06351	3.91886	-1.3027
H100	-3.00106	2.299935	-0.60673
C101	-1.75216	2.244487	-3.0099
H102	-0.99658	2.161507	-3.78665
H103	-2.15892	1.250364	-2.8201
H104	-2.5623	2.872087	-3.39539
H105	-0.72062	3.806319	-1.98683
X	0.335144	1.892742	-1.24452

C3_ER_Cheltop_int1

C1	-4.24811	-3.40733	0.803086
C2	-2.18985	-4.55236	0.175567
C3	-3.68894	-4.4622	-0.208
C4	-2.99282	-3.05031	1.633905
H5	-5.01504	-3.83888	1.454416
H6	-3.83165	-4.16453	-1.25186
H7	-3.19331	-2.5321	2.571516
H8	-4.68157	-2.53295	0.316267
H9	-1.6633	-5.42391	-0.21309
H10	-4.16831	-5.43652	-0.07767
C11	-2.25264	-4.39728	1.711644
H12	-1.27282	-4.32177	2.192787
H13	-2.83988	-5.18627	2.189713
C14	-0.0273	-3.2004	-0.30039
N15	0.679364	-2.12709	-0.33716
C16	2.124962	-2.5263	-0.21621
C17	2.026636	-4.04805	-0.54646
P18	-1.90389	-0.60785	0.624448
Ir19	-0.11649	-0.09114	-0.62362
C20	-1.72672	0.063225	2.315852
C21	-1.21879	1.218265	4.822343
C22	-1.29919	-0.73137	3.387349
C23	-1.89023	1.444918	2.508807
C24	-1.63879	2.015544	3.75474
C25	-1.05324	-0.15435	4.634624
H26	-2.20453	2.074789	1.683938
H27	-1.77441	3.084683	3.893851
H28	-0.72933	-0.78174	5.46021
H29	-1.02328	1.664189	5.792851
O30	0.623376	-4.367	-0.31047
C31	2.605647	-2.37186	1.23196
C32	1.788996	-1.8707	2.242788
C33	3.906854	-2.78757	1.554141
C34	2.263792	-1.76778	3.551829
H35	0.792442	-1.53115	2.007884

H98	-2.79653	3.555426	0.251551
H99	-3.81787	3.329763	-1.16489
H100	-3.21708	1.929716	-0.2924
C101	-2.14769	2.146223	-2.86327
H102	-1.30106	1.94138	-3.51701
H103	-2.61038	1.190749	-2.60853
H104	-2.87584	2.741054	-3.42328
H105	-1.51825	3.926135	-1.85453
H106	1.104133	0.10416	-1.60944
H107	0.114904	1.046027	-1.83155

C3_ER_Cheltop_PROD

C1	-4.26675	-3.67258	0.615979
C2	-2.1218	-4.62818	-0.05525
C3	-3.61261	-4.57791	-0.48109
C4	-3.05703	-3.32986	1.516942
H5	-5.0251	-4.21571	1.18934
H6	-3.74165	-4.17818	-1.49199
H7	-3.30099	-2.91577	2.496103
H8	-4.73625	-2.77643	0.207913
H9	-1.53944	-5.42995	-0.50896
H10	-4.03503	-5.58662	-0.47236
C11	-2.24177	-4.63387	1.484689
H12	-1.28254	-4.55527	2.005016
H13	-2.79388	-5.49923	1.862213
C14	-0.02563	-3.12019	-0.26549
N15	0.652759	-2.01852	-0.29784
C16	2.133951	-2.34172	-0.23135
C17	2.081967	-3.90841	-0.16193
P18	-2.09326	-0.73994	0.729234
Ir19	-0.13178	-0.14897	-0.56584
C20	-1.94911	-0.22305	2.478863
C21	-1.38944	0.705265	5.073425
C22	-1.35148	-1.06792	3.424222
C23	-2.26196	1.096297	2.84868
C24	-1.98592	1.552634	4.136944
C25	-1.07609	-0.60577	4.712407
H26	-2.72181	1.768936	2.129723
H27	-2.24339	2.571478	4.4144
H28	-0.61547	-1.27446	5.434251
H29	-1.1736	1.063146	6.075562
O30	0.667528	-4.25132	-0.18437
C31	2.76164	-1.83765	1.06586
C32	1.992218	-1.36108	2.127628
C33	4.145165	-1.99225	1.2508

C36	4.379958	-2.69146	2.859351
H37	4.560612	-3.16998	0.774192
C38	3.557648	-2.17623	3.865234
H39	1.616536	-1.35218	4.318873
H40	5.391548	-3.01213	3.090809
H41	3.928305	-2.09351	4.882891
C42	-3.56426	-0.10201	0.03967
C43	-4.55538	0.380686	0.905932
C44	-3.89771	-0.38602	-1.29464
C45	-5.85131	0.598784	0.435092
H46	-4.32536	0.583035	1.946516
C47	-5.19309	-0.1737	-1.75821
C48	-6.17205	0.324665	-0.89443
H49	-6.61094	0.97954	1.111632
H50	-5.43809	-0.39142	-2.79371
H51	-7.1814	0.495517	-1.25717
H52	-0.90346	-0.58102	-1.88882
H53	1.393004	0.43891	-1.77407
H54	-1.13969	-1.79503	3.247921
H55	-3.14004	-0.77127	-1.96974
H56	2.248036	-4.29336	-1.58771
C57	4.348209	-0.15448	-3.09485
C58	3.518542	-1.19073	-3.52488
C59	2.821912	-1.96063	-2.59579
C60	2.953724	-1.723	-1.2193
C61	3.776654	-0.67029	-0.79885
C62	4.469797	0.105197	-1.73047
H63	4.883622	0.451519	-3.81952
H64	3.397339	-1.38608	-4.58587
H65	2.151145	-2.73462	-2.9574
H66	3.868504	-0.44745	0.257741
H67	5.107519	0.913973	-1.38428
C68	-1.25734	2.752592	-1.83533
C69	-0.15888	1.934141	-1.12312
C70	1.135685	1.550476	-1.92553
C71	0.701422	4.091289	0.102896
C72	0.967784	2.013326	1.030969
C73	1.201786	4.195465	1.55248
H74	-0.12109	4.7761	-0.10829
H75	1.501354	4.264344	-0.6316
H76	2.074722	4.835085	1.683988
H77	0.405683	4.503034	2.238068
N78	0.274806	2.686389	0.084182
O79	1.583139	2.832843	1.893129
O80	1.06656	0.777357	1.098932

C34	2.592126	-1.00091	3.3353
H35	0.923606	-1.24532	2.019398
C36	4.742825	-1.64162	2.458466
H37	4.758846	-2.38191	0.443134
C38	3.967723	-1.13605	3.504888
H39	1.971375	-0.6093	4.136293
H40	5.815419	-1.76112	2.580891
H41	4.435676	-0.8554	4.443988
C42	-3.77867	-0.23573	0.204778
C43	-4.83242	0.015874	1.095695
C44	-4.03445	-0.23328	-1.17659
C45	-6.11396	0.278878	0.61055
H46	-4.65618	0.004359	2.166855
C47	-5.31793	0.021617	-1.65779
C48	-6.36071	0.281649	-0.76438
H49	-6.92261	0.474954	1.308859
H50	-5.50311	0.022905	-2.72808
H51	-7.36032	0.483987	-1.13711
H52	-0.70661	-0.74134	-1.88963
H53	1.664342	2.510185	-1.33961
H54	-1.08698	-2.08362	3.147017
H55	-3.22166	-0.4323	-1.87047
H56	2.559862	-4.40029	-1.01002
C57	3.806147	-0.8761	-3.96026
C58	2.943297	-1.97246	-3.94563
C59	2.442104	-2.44879	-2.73539
C60	2.793261	-1.8431	-1.52201
C61	3.643716	-0.73188	-1.54973
C62	4.151593	-0.25747	-2.75944
H63	4.197514	-0.50036	-4.90097
H64	2.657998	-2.4563	-4.87534
H65	1.747777	-3.28578	-2.74901
H66	3.898903	-0.22157	-0.62877
H67	4.810748	0.605876	-2.76218
C68	-1.77011	3.35101	-1.52363
C69	-0.43466	2.744915	-1.01147
C70	0.859572	3.090628	-1.80201
C71	-0.07164	4.481822	0.846769
C72	0.429445	2.271054	1.29545
C73	0.17306	4.264663	2.344684
H74	-0.97448	5.060878	0.646022
H75	0.778801	4.973861	0.360319
H76	0.873541	4.972524	2.788078
H77	-0.75983	4.251474	2.91802
N78	-0.20625	3.086009	0.40329

H81	2.626367	-4.66822	0.116959
N82	-2.0094	-2.2882	0.80949
C83	-1.53143	-3.21418	-0.24843
H84	-1.87774	-2.91657	-1.24924
C85	1.348045	2.218715	-6.1783
C86	1.604241	3.257122	-5.28135
C87	1.536953	3.025198	-3.90749
C88	1.206226	1.757836	-3.41625
C89	0.956684	0.721235	-4.31988
C90	1.026147	0.949045	-5.6934
H91	1.400754	2.397733	-7.24837
H92	1.858882	4.246408	-5.65104
H93	1.743873	3.836097	-3.21228
H94	0.697361	-0.26078	-3.93705
H95	0.826798	0.136676	-6.38686
H96	2.007019	2.011555	-1.4491
C97	-2.37398	3.176806	-0.86352
H98	-2.00478	3.684591	0.031543
H99	-3.06606	3.858927	-1.36764
H100	-2.94945	2.305824	-0.54335
C101	-1.91004	2.065256	-3.04328
H102	-1.2186	1.905675	-3.86645
H103	-2.32568	1.101075	-2.75047
H104	-2.73147	2.687567	-3.41416
H105	-0.77086	3.668125	-2.21289
X	0.335144	1.892742	-1.24452

C3_ER_Cheltop_TS2

C1	-4.17685	-3.43818	1.005979
C2	-2.15991	-4.48112	0.118916
C3	-3.6871	-4.39428	-0.12836
C4	-2.8513	-3.05527	1.705867
H5	-4.82221	-3.95886	1.721568
H6	-3.92205	-4.01206	-1.12693
H7	-2.96074	-2.57438	2.677412
H8	-4.72994	-2.57901	0.631905
H9	-1.66751	-5.33303	-0.34795
H10	-4.13797	-5.38696	-0.04308
C11	-2.08036	-4.38612	1.659261
H12	-1.06114	-4.31292	2.049305
H13	-2.60798	-5.2015	2.161882
C14	-0.03018	-3.112	-0.34897
N15	0.715403	-2.06246	-0.38933
C16	2.157765	-2.55361	-0.25169
C17	1.976885	-4.10576	-0.38143
O79	0.756296	2.941839	2.412915
O80	0.683466	1.072865	1.180199
H81	2.496237	-4.28957	0.77115
N82	-2.11476	-2.43393	0.794299
C83	-1.52831	-3.22106	-0.31676
H84	-1.83197	-2.8464	-1.30422
C85	0.886794	2.58168	-6.09768
C86	0.67256	3.84214	-5.53559
C87	0.675388	3.998196	-4.14913
C88	0.880588	2.900135	-3.30224
C89	1.118418	1.644244	-3.87493
C90	1.118048	1.48763	-5.26127
H91	0.885641	2.457743	-7.17661
H92	0.506847	4.704498	-6.17526
H93	0.507897	4.984302	-3.71995
H94	1.300421	0.786941	-3.23694
H95	1.307383	0.504988	-5.6846
H96	1.064601	4.147302	-1.59497
C97	-2.88915	3.263644	-0.47365
H98	-2.65602	3.77273	0.464138
H99	-3.80506	3.707949	-0.87328
H100	-3.11079	2.219078	-0.2425
C101	-2.25545	2.666225	-2.81468
H102	-1.56994	2.79278	-3.64878
H103	-2.38958	1.593542	-2.64225
H104	-3.22617	3.080232	-3.10374
H105	-1.5626	4.408594	-1.74717
H106	1.192192	0.157971	-1.38755
H107	-0.63743	1.627002	-1.08584

P18	-1.99026	-0.55544	0.60646
Ir19	-0.24064	0.065871	-0.61511
C20	-1.84075	0.08007	2.310682
C21	-1.35336	1.111739	4.873133
C22	-1.14716	-0.68124	3.265106
C23	-2.27699	1.370396	2.650242
C24	-2.04371	1.874605	3.929493
C25	-0.90585	-0.16673	4.537233
H26	-2.79629	1.981349	1.922895
H27	-2.40764	2.864528	4.19122
H28	-0.37013	-0.76799	5.2659
H29	-1.16665	1.511102	5.865641
O30	0.550552	-4.31232	-0.28136
C31	2.722904	-2.25972	1.140182
C32	2.009082	-1.5838	2.124021
C33	3.995174	-2.76635	1.456795
C34	2.555674	-1.39353	3.395513
H35	1.037492	-1.18172	1.895534
C36	4.538567	-2.58591	2.724671
H37	4.570688	-3.29353	0.699942
C38	3.817545	-1.89464	3.702247
H39	1.986355	-0.84284	4.138932
H40	5.52529	-2.98114	2.948464
H41	4.243022	-1.74731	4.690678
C42	-3.70514	-0.26474	0.03704
C43	-4.74078	0.055213	0.928994
C44	-4.02662	-0.63668	-1.27832
C45	-6.06848	0.031734	0.500598
H46	-4.52296	0.307521	1.960444
C47	-5.35519	-0.67096	-1.69708
C48	-6.3809	-0.33388	-0.80978
H49	-6.8599	0.287311	1.199168
H50	-5.58921	-0.9589	-2.71772
H51	-7.41589	-0.35881	-1.13691
H52	-1.0512	-0.40472	-1.87381
H53	1.604243	2.033348	-1.52451
H54	-0.78821	-1.67173	3.009966
H55	-3.23699	-0.89639	-1.97592
H56	2.309284	-4.51035	-1.33881
C57	4.48604	-1.02211	-3.57563
C58	3.458785	-1.94488	-3.78727
C59	2.731169	-2.43399	-2.70657
C60	3.013447	-2.01625	-1.39696
C61	4.037932	-1.08542	-1.19331
C62	4.769951	-0.59389	-2.27905

H63	5.059169	-0.6403	-4.41547
H64	3.221603	-2.27858	-4.79336
H65	1.912368	-3.12271	-2.89632
H66	4.261384	-0.73194	-0.19328
H67	5.563427	0.127526	-2.10657
C68	-1.82594	2.795402	-1.51803
C69	-0.53954	2.174965	-0.91833
C70	0.751081	2.677219	-1.75756
C71	-0.26387	4.184056	0.813806
C72	0.603967	2.104505	1.225311
C73	0.337841	4.105342	2.22836
H74	-1.26054	4.62477	0.810426
H75	0.369017	4.759736	0.126776
H76	1.049192	4.90012	2.454355
H77	-0.43221	4.058668	3.003167
N78	-0.28636	2.757303	0.449865
O79	1.053171	2.840366	2.245915
O80	0.996956	0.938144	1.03372
H81	2.451591	-4.6524	0.432234
N82	-1.97116	-2.23799	0.808409
C83	-1.53666	-3.12285	-0.29666
H84	-1.91212	-2.79755	-1.27613
C85	0.471733	3.547247	-6.00807
C86	-0.00924	4.447917	-5.05685
C87	0.108751	4.159079	-3.69936
C88	0.682333	2.95683	-3.25118
C89	1.217507	2.095345	-4.2194
C90	1.107863	2.382773	-5.58083
H91	0.378717	3.766584	-7.06782
H92	-0.46774	5.381375	-5.37106
H93	-0.24445	4.888598	-2.97533
H94	1.782586	1.229931	-3.9066
H95	1.535918	1.697509	-6.30753
H96	1.016175	3.636175	-1.30036
C97	-3.02773	2.95987	-0.57784
H98	-2.80676	3.540735	0.320343
H99	-3.82688	3.486256	-1.11082
H100	-3.43455	2.001417	-0.26957
C101	-2.28892	2.070368	-2.79435
H102	-1.45954	1.758878	-3.42805
H103	-2.86086	1.181097	-2.5311
H104	-2.93719	2.726273	-3.3846
H105	-1.54777	3.815605	-1.8139
H106	2.170064	0.082601	-2.28846
H107	1.522537	0.013901	-2.64664

C3_ER_Chelbot

C3_ER_Chelbot_SM

C1	-4.16247	-2.95873	-0.95958
C2	-2.08426	-4.23421	-1.11606
C3	-3.34145	-3.85776	-1.94525
C4	-3.25484	-2.93737	0.290598
H5	-5.13432	-3.40233	-0.71983
H6	-3.0825	-3.34128	-2.87486
H7	-3.72516	-2.57343	1.204659
H8	-4.33844	-1.95088	-1.34004
H9	-1.51971	-5.08643	-1.49506
H10	-3.89434	-4.76162	-2.21589
C11	-2.67699	-4.36381	0.304223
H12	-1.92464	-4.5257	1.081746
H13	-3.44503	-5.13927	0.372556
C14	0.172051	-3.1261	-0.46
N15	0.971339	-2.14999	-0.17699
C16	2.297827	-2.71908	0.275285
C17	1.936137	-4.23983	0.36871
P18	-1.63417	-0.58196	0.429118
Ir19	0.558135	-0.10417	-0.60809
C20	-1.64813	-0.55929	2.259773
C21	-1.48251	-0.50155	5.062194
C22	-1.68372	-1.75012	2.999481
C23	-1.50772	0.66191	2.940944
C24	-1.42883	0.687585	4.331736
C25	-1.6058	-1.71889	4.392288
H26	-1.45714	1.594778	2.394279
H27	-1.31979	1.641329	4.839939
H28	-1.63404	-2.64903	4.952397
H29	-1.42187	-0.47965	6.146553
O30	0.624701	-4.36086	-0.25733
C31	2.715175	-2.24987	1.666948
C32	1.824404	-1.61469	2.534679
C33	3.990506	-2.59299	2.14188
C34	2.21117	-1.28088	3.833846
H35	0.824429	-1.37546	2.207525
C36	4.37565	-2.26491	3.440322
H37	4.691361	-3.10652	1.489818
C38	3.489776	-1.59735	4.288616
H39	1.499661	-0.77923	4.483244
H40	5.370464	-2.52895	3.78748
H41	3.794257	-1.33591	5.298047
C42	-3.07062	0.438329	-0.08711
C43	-3.95768	1.062592	0.79685

jag_C3_ER_Chelbot_int2

C1	-3.98064	-2.87851	-1.56255
C2	-1.97142	-4.23452	-1.30007
C3	-3.05847	-3.87029	-2.3449
C4	-3.26549	-2.77463	-0.19825
H5	-4.98754	-3.28614	-1.42376
H6	-2.62646	-3.42486	-3.24667
H7	-3.85739	-2.31033	0.590348
H8	-4.08311	-1.9052	-2.04289
H9	-1.4068	-5.13951	-1.52113
H10	-3.60406	-4.76731	-2.65063
C11	-2.77689	-4.21882	0.017655
H12	-2.15703	-4.34498	0.910738
H13	-3.5883	-4.95192	0.028658
C14	0.257452	-3.24788	-0.43211
N15	1.022963	-2.3415	0.066027
C16	2.257395	-3.00326	0.613071
C17	2.066254	-4.4705	0.096664
P18	-1.50175	-0.57464	0.241258
Ir19	0.680155	-0.17332	-0.31493
C20	-1.71208	-0.57676	2.065442
C21	-1.95621	-0.48352	4.85948
C22	-2.23479	-1.66955	2.767177
C23	-1.27847	0.552572	2.78231
C24	-1.40865	0.600596	4.168017
C25	-2.35613	-1.6202	4.157772
H26	-0.81499	1.386821	2.264213
H27	-1.06759	1.479826	4.70711
H28	-2.75594	-2.4779	4.690881
H29	-2.05426	-0.44758	5.94084
O30	0.701515	-4.50792	-0.41095
C31	2.282337	-2.98186	2.141578
C32	1.357362	-2.26918	2.904046
C33	3.274915	-3.72335	2.802345
C34	1.422823	-2.29068	4.300564
H35	0.585658	-1.69267	2.416964
C36	3.34062	-3.74553	4.193463
H37	4.014735	-4.27235	2.223557
C38	2.412151	-3.02551	4.950792
H39	0.689475	-1.72865	4.870658
H40	4.118305	-4.32238	4.68585
H41	2.462754	-3.04148	6.035533
C42	-2.77663	0.601839	-0.36746
C43	-3.37323	1.576111	0.445117

C44	-3.27062	0.572715	-1.47319	C44	-3.11386	0.55392	-1.72933
C45	-5.02089	1.822292	0.302518	C45	-4.28165	2.488439	-0.09659
H46	-3.82823	0.960116	1.868777	H46	-3.14435	1.624374	1.50374
C47	-4.33383	1.328102	-1.96066	C47	-4.03466	1.455684	-2.2618
C48	-5.20864	1.9607	-1.07174	C48	-4.61615	2.433425	-1.44947
H49	-5.70428	2.302611	0.997054	H49	-4.7353	3.236489	0.547224
H50	-4.48375	1.421335	-3.03268	H50	-4.30573	1.392572	-3.31253
H51	-6.03671	2.552356	-1.45113	H51	-5.33058	3.13748	-1.86538
H52	1.232255	0.274951	0.732036	H52	0.856304	0.196388	1.185598
H53	2.041136	-0.16673	-1.16949	H53	1.930231	1.627962	-2.38154
H54	-1.74936	-2.70472	2.490644	H54	-2.52309	-2.57397	2.248344
H55	-2.59798	0.071916	-2.16408	H55	-2.66155	-0.19391	-2.37265
H56	2.630441	-4.89079	-0.16234	H56	2.733184	-4.73308	-0.72775
C57	5.062536	-1.846	-2.95821	C57	5.431445	-0.75467	-1.31211
C58	4.068543	-2.81416	-3.11075	C58	4.54984	-1.53859	-2.06276
C59	3.205649	-3.09994	-2.05468	C59	3.576191	-2.30318	-1.42243
C60	3.318497	-2.42618	-0.83091	C60	3.463142	-2.30316	-0.02382
C61	4.299605	-1.43691	-0.69808	C61	4.353574	-1.5225	0.719839
C62	5.172486	-1.1582	-1.75044	C62	5.332017	-0.75605	0.079408
H63	5.742194	-1.62677	-3.77658	H63	6.190154	-0.15619	-1.80788
H64	3.966549	-3.34929	-4.05037	H64	4.614804	-1.54867	-3.14709
H65	2.427861	-3.84595	-2.19968	H65	2.866354	-2.86177	-2.02652
H66	4.38079	-0.87566	0.22538	H66	4.27611	-1.49198	1.800532
H67	5.933391	-0.39309	-1.62567	H67	6.013393	-0.15515	0.674918
C68	2.309309	2.455737	-1.49248	C68	1.267965	3.624811	-2.61702
C69	0.934827	1.780133	-1.62323	C69	0.966953	2.140863	-2.27297
C70	-0.15309	1.9816	-0.71704	C70	0.39377	1.879605	-0.8438
C71	0.175032	2.689067	-3.91877	C71	-0.9903	2.103963	-4.05254
C72	-0.168	0.472023	-3.38791	C72	-0.07348	0.143384	-3.27566
C73	-0.34023	1.903016	-5.13678	C73	-1.43782	0.916168	-4.91742
H74	1.050246	3.290774	-4.16111	H74	-0.62476	2.928618	-4.66089
H75	-0.58792	3.336661	-3.46649	H75	-1.78481	2.467628	-3.39287
H76	-1.20814	2.351157	-5.62132	H76	-2.51871	0.847872	-5.04619
H77	0.447452	1.725379	-5.87479	H77	-0.94685	0.904423	-5.89517
N78	0.50486	1.583079	-3.01121	N78	0.085507	1.479657	-3.27198
O79	-0.74132	0.616614	-4.59082	O79	-0.99701	-0.25243	-4.17831
O80	-0.29765	-0.5654	-2.72174	O80	0.498928	-0.7007	-2.56511
H81	1.831765	-4.56827	1.403657	H81	2.144535	-5.21322	0.888689
N82	-2.02534	-2.1575	-0.01944	N82	-1.95835	-2.07174	-0.37376
C83	-1.22942	-2.95015	-0.99106	C83	-1.06262	-2.99079	-1.12609
H84	-1.13974	-2.44407	-1.95665	H84	-0.79183	-2.56127	-2.09773
H85	-1.128	1.97256	-1.19895	H85	-0.67982	2.081642	-0.91194
C86	-0.39296	4.457059	2.795261	C86	1.91942	4.359174	2.367505
C87	-1.42421	4.414711	1.856955	C87	0.548644	4.250401	2.136297
C88	-1.31236	3.597593	0.731763	C88	0.074549	3.472479	1.077767

C89	-0.16923	2.807728	0.518994
C90	0.857271	2.854749	1.478022
C91	0.747029	3.672669	2.598791
H92	-0.47652	5.091213	3.673177
H93	-2.31939	5.013105	2.000562
H94	-2.12809	3.558954	0.01425
H95	1.732368	2.227258	1.365247
H96	1.551994	3.690129	3.328051
C97	3.320351	1.927417	-2.51739
H98	3.403517	0.838951	-2.47912
H99	3.044847	2.20957	-3.53925
H100	4.308763	2.35267	-2.3169
C101	2.210186	3.995759	-1.5534
H102	1.489046	4.385324	-0.83206
H103	3.185816	4.434558	-1.32159
H104	1.926762	4.355938	-2.54702
H105	2.680801	2.195098	-0.49962
X	0.332262	1.867163	-1.24276

C3_ER_Chelbot_TS1

C1	-4.04644	-3.46425	-0.71608
C2	-1.84423	-4.39029	-1.23333
C3	-3.22304	-4.103	-1.88623
C4	-3.03158	-3.4971	0.449095
H5	-4.93318	-4.05787	-0.47131
H6	-3.14036	-3.44356	-2.7556
H7	-3.45343	-3.34172	1.442844
H8	-4.37348	-2.44348	-0.9267
H9	-1.21016	-5.08718	-1.78164
H10	-3.67512	-5.03915	-2.22582
C11	-2.2647	-4.8066	0.194982
H12	-1.42352	-4.95549	0.877228
H13	-2.90782	-5.69123	0.202801
C14	0.283957	-3.02344	-0.59805
N15	1.008106	-1.9833	-0.32666
C16	2.367552	-2.47742	0.128748
C17	2.162501	-4.04193	0.083344
P18	-1.75216	-0.93888	0.700363
Ir19	0.055377	0.062793	-0.42751
C20	-1.59505	-0.98887	2.515724
C21	-1.25069	-0.94337	5.292134
C22	-1.47975	-2.18672	3.229712
C23	-1.50547	0.236699	3.197202
C24	-1.34008	0.256823	4.579812
C25	-1.31213	-2.16101	4.615524
H26	-1.55713	1.172607	2.645753

C89	0.951682	2.769932	0.2371
C90	2.330434	2.903611	0.477478
C91	2.808725	3.687861	1.524393
H92	2.2933	4.965252	3.187525
H93	-0.15486	4.780433	2.773226
H94	-0.99649	3.408295	0.893141
H95	3.042701	2.385335	-0.16082
H96	3.880038	3.774795	1.685741
C97	1.965817	3.776531	-3.97815
H98	2.836915	3.116051	-4.05248
H99	1.309334	3.549225	-4.82434
H100	2.316021	4.805328	-4.10944
C101	0.106503	4.618155	-2.43587
H102	-0.38832	4.485015	-1.47103
H103	0.500016	5.639279	-2.4627
H104	-0.6512	4.55254	-3.22049
H105	2.010597	3.901652	-1.86166
H107	2.413561	0.218992	-0.11672
H108	2.386856	0.00773	-0.9041

jag_C3_ER_Chelbot_TS3

C1	-4.012	-2.73533	-1.59376
C2	-2.00349	-4.11457	-1.44359
C3	-3.08622	-3.65558	-2.45668
C4	-3.29984	-2.75221	-0.22349
H5	-5.02015	-3.15218	-1.49745
H6	-2.6488	-3.13192	-3.31303
H7	-3.889	-2.35529	0.603053
H8	-4.10901	-1.72254	-1.98651
H9	-1.4424	-4.99995	-1.7408
H10	-3.63093	-4.52097	-2.84431
C11	-2.81339	-4.20937	-0.13028
H12	-2.19671	-4.40831	0.751455
H13	-3.62492	-4.94066	-0.18328
C14	0.203929	-3.19711	-0.44986
N15	1.02082	-2.31578	0.022021
C16	2.225764	-3.02071	0.599144
C17	1.877203	-4.51748	0.271715
P18	-1.51434	-0.55741	0.284461
Ir19	0.829392	-0.20234	-0.28619
C20	-1.72257	-0.57271	2.103038
C21	-1.92291	-0.4747	4.897485
C22	-2.25907	-1.65484	2.810322
C23	-1.25206	0.54711	2.811072
C24	-1.36218	0.598399	4.198833

H27	-1.27669	1.207668	5.101256	C25	-2.35799	-1.60341	4.202486
H28	-1.22247	-3.09378	5.164715	H26	-0.78612	1.373166	2.280057
H29	-1.12206	-0.92646	6.37057	H27	-0.99728	1.470149	4.734617
O30	0.83295	-4.22812	-0.45184	H28	-2.7695	-2.45088	4.743136
C31	2.721528	-2.11291	1.573256	H29	-2.00439	-0.43686	5.980206
C32	1.889365	-1.37885	2.416814	O30	0.557571	-4.4761	-0.34142
C33	3.890286	-2.67283	2.123425	C31	2.329028	-2.86367	2.116258
C34	2.231406	-1.15705	3.753509	C32	1.382276	-2.18322	2.880503
H35	0.957843	-0.98718	2.047808	C33	3.383851	-3.51564	2.776771
C36	4.235015	-2.4537	3.454174	C34	1.500326	-2.12396	4.27244
H37	4.538278	-3.28983	1.506754	H35	0.547223	-1.69623	2.402275
C38	3.408695	-1.68371	4.275987	C36	3.500066	-3.46115	4.163565
H39	1.554254	-0.58413	4.379656	H37	4.127556	-4.05868	2.19862
H40	5.147404	-2.89056	3.849953	C38	2.559122	-2.75657	4.919818
H41	3.677117	-1.51216	5.314395	H39	0.750725	-1.58211	4.840562
C42	-3.33643	-0.06356	0.411673	H40	4.327353	-3.96725	4.652911
C43	-4.32139	0.056699	1.400843	H41	2.651062	-2.7099	6.001003
C44	-3.58471	0.434204	-0.87886	C42	-2.79119	0.638453	-0.29319
C45	-5.53502	0.679203	1.106524	C43	-3.47381	1.517243	0.558723
H46	-4.14198	-0.32458	2.401333	C44	-3.05118	0.689139	-1.673
C47	-4.80159	1.048281	-1.1677	C45	-4.39512	2.430211	0.040389
C48	-5.77534	1.177384	-0.17379	H46	-3.29772	1.490354	1.628361
H49	-6.29104	0.776213	1.880369	C47	-3.98149	1.592618	-2.18467
H50	-4.99094	1.428366	-2.16763	C48	-4.65161	2.471601	-1.32948
H51	-6.72051	1.662925	-0.3987	H49	-4.91974	3.102484	0.713386
H52	0.636068	0.434376	0.953909	H50	-4.19359	1.60617	-3.25081
H53	-0.93457	1.286653	-0.21567	H51	-5.37493	3.176802	-1.7285
H54	-1.49954	-3.1365	2.705756	H52	1.090456	0.062014	1.219535
H55	-2.82974	0.326939	-1.6542	H53	1.957465	1.769654	-2.35906
H56	2.867565	-4.5479	-0.5771	H54	-2.57446	-2.5512	2.290208
C57	5.406552	-1.8087	-2.90378	H55	-2.5413	0.005935	-2.3454
C58	4.148684	-2.31692	-3.2376	H56	2.561175	-4.97728	-0.44335
C59	3.174041	-2.46572	-2.25514	C57	5.606568	-1.5839	-1.71472
C60	3.429426	-2.10857	-0.92062	C58	4.586871	-2.32435	-2.31716
C61	4.677613	-1.56359	-0.6064	C59	3.525699	-2.79807	-1.55079
C62	5.661625	-1.42692	-1.58939	C60	3.462658	-2.54538	-0.17281
H63	6.174769	-1.70448	-3.66429	C61	4.478439	-1.78808	0.418355
H64	3.92989	-2.60628	-4.26159	C62	5.546635	-1.3167	-0.34773
H65	2.205963	-2.87581	-2.52923	H63	6.43932	-1.21709	-2.30816
H66	4.895028	-1.24102	0.404317	H64	4.616846	-2.5298	-3.38345
H67	6.628604	-1.0113	-1.32046	H65	2.724047	-3.34304	-2.04325
C68	2.693942	1.977072	-1.32243	H66	4.436322	-1.5527	1.475356
C69	1.23654	1.559297	-1.58239	H67	6.331087	-0.73593	0.129284
C70	0.104461	2.231961	-0.93567	C68	1.273816	3.764729	-2.5825
C71	1.106622	2.255408	-4.09628	C69	0.989798	2.274564	-2.26464

C72	-0.01579	0.400865	-3.34079
C73	0.556625	1.430962	-5.26994
H74	2.149033	2.534687	-4.24074
H75	0.520301	3.163592	-3.89854
H76	-0.02043	2.00709	-5.99355
H77	1.344869	0.870208	-5.78158
N78	0.941108	1.29127	-2.99689
O79	-0.33879	0.473228	-4.63902
O80	-0.59216	-0.39249	-2.57561
H81	2.182525	-4.49436	1.075773
N82	-1.94802	-2.51809	0.175222
C83	-1.17343	-3.01343	-0.99311
H84	-1.28225	-2.36842	-1.86839
H85	-0.69008	2.503883	-1.63444
C86	0.244302	5.197605	2.18891
C87	-0.55738	5.368053	1.061697
C88	-0.58648	4.385569	0.071624
C89	0.187105	3.222924	0.18807
C90	0.980556	3.055529	1.334278
C91	1.011848	4.037161	2.320338
H92	0.267521	5.958906	2.963118
H93	-1.16455	6.261773	0.951622
H94	-1.21725	4.527285	-0.80332
H95	1.570436	2.154562	1.457466
H96	1.636014	3.893162	3.197695
C97	3.668859	1.187585	-2.20236
H98	3.399218	0.136489	-2.24905
H99	3.695625	1.573259	-3.22677
H100	4.68378	1.267871	-1.80703
C101	2.951455	3.492084	-1.4894
H102	2.32968	4.101127	-0.83208
H103	3.999097	3.71216	-1.25778
H104	2.773769	3.822388	-2.51849
H105	2.90378	1.717739	-0.28057
X	0.332262	1.867163	-1.24276

C3_ER_Chelbot_INT1

C1	-4.09098	-2.80674	-1.48151
C2	-2.12993	-4.23952	-1.26557
C3	-3.22642	-3.83432	-2.28533
C4	-3.3638	-2.75963	-0.11992
H5	-5.11855	-3.1598	-1.34587
H6	-2.80042	-3.40824	-3.19907
H7	-3.93375	-2.29583	0.685279
H8	-4.13848	-1.82023	-1.94491
C70	0.419159	1.988379	-0.82771
C71	-0.93907	2.217706	-4.06158
C72	-0.01035	0.25541	-3.27669
C73	-1.36959	1.026064	-4.93044
H74	-0.56567	3.040042	-4.66864
H75	-1.74474	2.584788	-3.41666
H76	-2.4488	0.950207	-5.07022
H77	-0.86999	1.02057	-5.90393
N78	0.127588	1.600881	-3.26381
O79	-0.9275	-0.1385	-4.1894
O80	0.561049	-0.58487	-2.57159
H81	1.803675	-5.13306	1.1677
N82	-1.99369	-2.04192	-0.33925
C83	-1.09558	-2.89226	-1.16295
H84	-0.80905	-2.38757	-2.0928
H85	-0.65963	1.897418	-0.93612
C86	1.109505	4.83202	2.358038
C87	-0.18355	4.56797	1.908414
C88	-0.39302	3.657404	0.870292
C89	0.684591	2.993707	0.268484
C90	1.979735	3.259025	0.740673
C91	2.192437	4.174557	1.768149
H92	1.274816	5.539511	3.16545
H93	-1.03254	5.070532	2.363713
H94	-1.40454	3.455354	0.525116
H95	2.830257	2.730565	0.313457
H96	3.202706	4.367472	2.117795
C97	2.047816	3.925021	-3.90062
H98	2.957111	3.314592	-3.90723
H99	1.452758	3.639791	-4.77452
H100	2.344649	4.969682	-4.03695
C101	0.082584	4.73904	-2.49807
H102	-0.56064	4.535265	-1.63971
H103	0.462826	5.758671	-2.38302
H104	-0.5366	4.737145	-3.39835
H105	1.960288	4.055384	-1.77973
H107	1.632158	1.140709	-0.40673
H108	2.416745	-0.2278	-0.52064

jag_C3_ER_Chelbot_PROD

C1	-4.01655	-2.80759	-1.58036
C2	-1.97983	-4.15199	-1.50539
C3	-3.08118	-3.67378	-2.48845
C4	-3.3011	-2.88207	-0.21324
H5	-5.02029	-3.23891	-1.50526

H9	-1.58913	-5.15454	-1.50817
H10	-3.81373	-4.71033	-2.57443
C11	-2.91759	-4.22244	0.062781
H12	-2.29281	-4.38237	0.94697
H13	-3.75078	-4.93091	0.071166
C14	0.148229	-3.28522	-0.45041
N15	0.856372	-2.36121	0.088198
C16	2.188286	-2.90165	0.458431
C17	1.999161	-4.41818	0.131104
P18	-1.56489	-0.59349	0.311555
Ir19	0.531074	-0.24443	-0.25293
C20	-1.80218	-0.5906	2.129078
C21	-2.03512	-0.50326	4.922381
C22	-2.26282	-1.70839	2.83525
C23	-1.42929	0.564898	2.839112
C24	-1.55353	0.608315	4.224745
C25	-2.38051	-1.66099	4.226677
H26	-1.01668	1.421771	2.315286
H27	-1.25964	1.506968	4.759502
H28	-2.73571	-2.53517	4.76457
H29	-2.12908	-0.469	6.004052
O30	0.692243	-4.50457	-0.52298
C31	2.48147	-2.70372	1.940532
C32	1.485854	-2.298	2.832866
C33	3.7669	-2.97856	2.424167
C34	1.77495	-2.15452	4.190815
H35	0.490595	-2.07474	2.47052
C36	4.052005	-2.84364	3.781567
H37	4.550717	-3.28242	1.734228
C38	3.056581	-2.42613	4.668356
H39	0.991949	-1.82441	4.867687
H40	5.052784	-3.05692	4.145871
H41	3.282378	-2.31266	5.724755
C42	-2.79216	0.587897	-0.35958
C43	-3.49569	1.510278	0.424831
C44	-2.98341	0.581275	-1.75178
C45	-4.37367	2.415717	-0.1759
H46	-3.3726	1.522838	1.502177
C47	-3.86656	1.480007	-2.34424
C48	-4.55982	2.404905	-1.55718
H49	-4.91762	3.124557	0.441674
H50	-4.0251	1.455748	-3.41917
H51	-5.24833	3.106257	-2.01939
H52	0.721199	0.121231	1.243231
H53	2.103883	1.376409	-2.05265
H6	-2.66377	-3.10957	-3.32869
H7	-3.89626	-2.54299	0.634902
H8	-4.12292	-1.77723	-1.92283
H9	-1.39944	-5.00867	-1.84734
H10	-3.61274	-4.53445	-2.90385
C11	-2.78144	-4.33048	-0.19538
H12	-2.15765	-4.5573	0.67442
H13	-3.57539	-5.07763	-0.28302
C14	0.204752	-3.2235	-0.47408
N15	1.022059	-2.34155	0.004248
C16	2.253178	-3.04554	0.54285
C17	1.780557	-4.53247	0.447995
P18	-1.56107	-0.64539	0.335218
Ir19	0.768721	-0.31138	-0.16947
C20	-1.80969	-0.67278	2.147238
C21	-1.97619	-0.62124	4.945992
C22	-2.27343	-1.79223	2.847977
C23	-1.39751	0.464654	2.864902
C24	-1.49138	0.492725	4.253827
C25	-2.35595	-1.76397	4.242504
H26	-0.98525	1.3223	2.33779
H27	-1.17233	1.377509	4.797186
H28	-2.71368	-2.63917	4.777156
H29	-2.04366	-0.60126	6.029945
O30	0.565422	-4.50135	-0.35517
C31	2.503357	-2.74057	2.017661
C32	1.459045	-2.31605	2.847911
C33	3.738379	-3.05404	2.601702
C34	1.659815	-2.14632	4.217649
H35	0.482347	-2.10901	2.430973
C36	3.937299	-2.89117	3.973622
H37	4.552453	-3.42311	1.985632
C38	2.903492	-2.42406	4.786586
H39	0.835388	-1.797	4.83251
H40	4.905362	-3.13063	4.404523
H41	3.063437	-2.29017	5.852456
C42	-2.81568	0.557488	-0.28286
C43	-3.5405	1.432196	0.537752
C44	-2.99022	0.631781	-1.676
C45	-4.4163	2.365186	-0.02349
H46	-3.43204	1.387163	1.616037
C47	-3.87502	1.554587	-2.23119
C48	-4.5862	2.43048	-1.40592
H49	-4.975	3.033297	0.625838
H50	-4.02312	1.582476	-3.30757

H54	-2.50772	-2.62648	2.317034
H55	-2.44797	-0.13379	-2.36902
H56	2.744611	-4.83927	-0.54387
C57	4.567235	-0.44255	-2.20631
C58	4.182349	-1.72133	-2.60795
C59	3.470527	-2.55073	-1.73927
C60	3.148923	-2.12076	-0.4505
C61	3.536357	-0.82716	-0.04841
C62	4.243195	0.002164	-0.92413
H63	5.124034	0.198646	-2.88347
H64	4.428535	-2.07556	-3.60465
H65	3.14682	-3.52358	-2.09586
H66	3.326659	-0.4919	0.962639
H67	4.559419	0.987672	-0.59455
C68	1.71383	3.425077	-2.40923
C69	1.199236	1.996283	-2.09428
C70	0.380633	1.81254	-0.77585
C71	-0.58963	2.09958	-4.02803
C72	0.164015	0.082816	-3.2251
C73	-1.07184	0.946048	-4.9214
H74	-0.14683	2.903677	-4.61395
H75	-1.38901	2.508786	-3.39938
H76	-2.1477	0.945805	-5.1006
H77	-0.53776	0.907919	-5.87553
N78	0.412098	1.406074	-3.20895
O79	-0.73898	-0.25133	-4.17128
O80	0.645034	-0.79081	-2.48201
H81	1.949805	-5.01838	1.040343
N82	-2.03512	-2.09888	-0.28789
C83	-1.19038	-3.01988	-1.09569
H84	-0.9444	-2.57786	-2.07002
H85	-0.6559	2.065792	-1.02365
C86	1.476662	4.231194	2.652851
C87	0.16842	4.22948	2.167538
C88	-0.16548	3.471683	1.042057
C89	0.791993	2.685588	0.38009
C90	2.105478	2.705188	0.880277
C91	2.445538	3.466707	1.99628
H92	1.740926	4.823509	3.523761
H93	-0.5953	4.827294	2.6587
H94	-1.18818	3.483388	0.668918
H95	2.86392	2.097948	0.394857
H96	3.470063	3.462017	2.359131
C97	2.570356	3.447136	-3.68442
H98	3.36074	2.689028	-3.64489

H51	-5.27479	3.150635	-1.83803
H52	1.0547	-0.16117	1.347757
H53	2.010871	1.676168	-2.08355
H54	-2.54582	-2.6977	2.318115
H55	-2.45155	-0.04981	-2.32654
H56	2.498866	-5.19209	-0.03787
C57	5.371148	-2.05573	-2.30999
C58	4.396291	-3.00495	-2.61979
C59	3.421106	-3.33457	-1.68081
C60	3.402687	-2.72744	-0.41785
C61	4.369372	-1.75807	-0.12746
C62	5.351851	-1.43259	-1.06262
H63	6.13783	-1.80272	-3.03687
H64	4.394166	-3.48996	-3.59167
H65	2.657997	-4.05899	-1.95391
H66	4.349612	-1.24524	0.826149
H67	6.100426	-0.68541	-0.81421
C68	1.698653	3.739191	-2.44813
C69	1.140419	2.338497	-2.09593
C70	0.447678	2.189351	-0.69207
C71	-0.74088	2.475152	-3.92716
C72	0.033522	0.424001	-3.19935
C73	-1.19851	1.354391	-4.87189
H74	-0.30708	3.307234	-4.47719
H75	-1.54993	2.848212	-3.28684
H76	-2.27132	1.351674	-5.06746
H77	-0.64983	1.36396	-5.81869
N78	0.266298	1.760406	-3.13554
O79	-0.86446	0.133398	-4.16755
O80	0.504733	-0.47538	-2.49857
H81	1.509126	-4.93085	1.426538
N82	-2.01174	-2.14202	-0.28888
C83	-1.10216	-2.9203	-1.17341
H84	-0.84028	-2.36011	-2.07808
H85	-0.59485	1.923004	-0.84061
C86	0.586994	5.297269	2.297997
C87	-0.62895	4.926494	1.723177
C88	-0.66714	3.92555	0.75099
C89	0.513209	3.292641	0.340802
C90	1.727017	3.652649	0.941362
C91	1.764922	4.655474	1.90776
H92	0.617492	6.076494	3.054031
H93	-1.54902	5.414802	2.031659
H94	-1.61675	3.633261	0.308345
H95	2.641646	3.133371	0.663655

H99	1.980768	3.257212	-4.58784
H100	3.047819	4.424405	-3.80649
C101	0.662947	4.548641	-2.39246
H102	0.039299	4.502636	-1.49687
H103	1.171706	5.517842	-2.38749
H104	0.007682	4.542803	-3.26724
H105	2.387238	3.635049	-1.57062
X	0.332262	1.867163	-1.24276

jag_C3_ER_Chelbot_TS2

C1	-3.93835	-2.71353	-1.61154
C2	-1.94822	-4.11256	-1.40996
C3	-3.01803	-3.66788	-2.44291
C4	-3.23306	-2.69667	-0.23871
H5	-4.95068	-3.11741	-1.50608
H6	-2.56753	-3.17168	-3.30865
H7	-3.81893	-2.26952	0.575367
H8	-4.02504	-1.71236	-2.03494
H9	-1.39594	-5.01285	-1.67749
H10	-3.57076	-4.53602	-2.81215
C11	-2.76658	-4.1583	-0.10134
H12	-2.15694	-4.34639	0.787458
H13	-3.5901	-4.8769	-0.13766
C14	0.271274	-3.1912	-0.43425
N15	1.046664	-2.29268	0.064292
C16	2.255605	-2.9703	0.643295
C17	2.007553	-4.4593	0.200706
P18	-1.44647	-0.53418	0.292733
Ir19	0.630642	-0.13472	-0.34062
C20	-1.6352	-0.61003	2.116049
C21	-1.87272	-0.63973	4.913751
C22	-2.09244	-1.75669	2.776128
C23	-1.2641	0.514683	2.874361
C24	-1.39146	0.499953	4.261742
C25	-2.21082	-1.76842	4.167921
H26	-0.85565	1.394848	2.385931
H27	-1.10118	1.376694	4.833601
H28	-2.55878	-2.6678	4.667472
H29	-1.96804	-0.65154	5.99545
O30	0.665949	-4.46322	-0.36379
C31	2.306781	-2.87324	2.167851
C32	1.490874	-2.01426	2.903449
C33	3.218629	-3.68889	2.858258
C34	1.580194	-1.96806	4.297551
H35	0.784096	-1.37792	2.395775
H96	2.71147	4.929833	2.364379
C97	2.541071	3.679928	-3.73269
H98	3.326913	2.919992	-3.6655
H99	1.930905	3.4468	-4.61215
H100	3.02155	4.646226	-3.91418
C101	0.703813	4.915921	-2.48409
H102	-0.01253	4.886058	-1.66145
H103	1.259911	5.854216	-2.39663
H104	0.14614	4.972166	-3.42212
H105	2.38753	3.946443	-1.62141
H107	1.095206	1.412215	-0.10894
H108	2.353954	-0.16609	-0.38121

C36	3.311017	-3.64122	4.247275
H37	3.879437	-4.352	2.304102
C38	2.488365	-2.77803	4.975652
H39	0.928893	-1.29321	4.843372
H40	4.02731	-4.27707	4.759521
H41	2.558141	-2.74074	6.058642
C42	-2.74423	0.635362	-0.26987
C43	-3.44738	1.489025	0.589122
C44	-3.01244	0.674544	-1.64846
C45	-4.40055	2.370801	0.074995
H46	-3.26736	1.464508	1.657804
C47	-3.97115	1.548411	-2.15469
C48	-4.66391	2.403703	-1.29288
H49	-4.943	3.026041	0.750013
H50	-4.18637	1.560633	-3.22004
H51	-5.41117	3.085739	-1.68726
H52	0.832294	0.290297	1.136683
H53	1.991924	1.703458	-2.30892
H54	-2.33147	-2.65384	2.219396
H55	-2.47454	0.011494	-2.31992
H56	2.693958	-4.79274	-0.58062
C57	5.69995	-1.33801	-1.44137
C58	4.629551	-1.89549	-2.14518
C59	3.538269	-2.4169	-1.4548
C60	3.496933	-2.39373	-0.05219
C61	4.566038	-1.822	0.643636
C62	5.661984	-1.30081	-0.04912
H63	6.55383	-0.93292	-1.97651
H64	4.642854	-1.92181	-3.23109
H65	2.698177	-2.81598	-2.01678
H66	4.549257	-1.77651	1.7263
H67	6.485744	-0.86281	0.50701
C68	1.248223	3.664067	-2.62831
C69	1.0011	2.171349	-2.2773
C70	0.333892	1.903567	-0.89098
C71	-0.88843	1.991412	-4.11181
C72	0.146961	0.108107	-3.28964
C73	-1.29296	0.749917	-4.92372
H74	-0.56405	2.806314	-4.75729
H75	-1.69095	2.34937	-3.45922
H76	-2.3715	0.619157	-5.02249
H77	-0.82587	0.727932	-5.91262
N78	0.221717	1.450154	-3.31815
O79	-0.77211	-0.36784	-4.1565
O80	0.792387	-0.67613	-2.56799

H81	2.01526	-5.16092	1.033633
N82	-1.91532	-2.00493	-0.37435
C83	-1.02277	-2.89758	-1.15789
H84	-0.72636	-2.42906	-2.10386
H85	-0.73382	2.091403	-1.0369
C86	1.583404	4.379187	2.436092
C87	0.231317	4.214188	2.133408
C88	-0.15291	3.441708	1.036759
C89	0.798423	2.802731	0.224756
C90	2.155806	2.997413	0.531949
C91	2.543951	3.774395	1.622061
H92	1.886444	4.981967	3.287193
H93	-0.52658	4.696749	2.745363
H94	-1.2093	3.328009	0.800711
H95	2.91436	2.53783	-0.09359
H96	3.600763	3.90925	1.836453
C97	1.991623	3.828135	-3.96315
H98	2.892933	3.206213	-3.99487
H99	1.376276	3.559339	-4.82801
H100	2.30001	4.869712	-4.0981
C101	0.032529	4.601154	-2.51609
H102	-0.49233	4.468025	-1.56709
H103	0.375315	5.639957	-2.55593
H104	-0.6892	4.477899	-3.32748
H105	1.942313	3.987905	-1.84608
H107	3.310868	0.471611	-0.37389
H108	3.287537	0.320133	-1.10657

C3_ZS_Cheltop

C3_ZS_Cheltop_SM

C1	-3.9825	-3.39713	-0.74319
C2	-1.7632	-4.41248	-0.77251
C3	-3.0528	-4.29503	-1.62635
C4	-3.0978	-3.13606	0.497881
H5	-4.8983	-3.92313	-0.45436
H6	-2.8584	-3.86286	-2.61329
H7	-3.6204	-2.74861	1.372277
H8	-4.273	-2.466	-1.23281
H9	-1.0871	-5.21641	-1.06389
H10	-3.486	-5.28678	-1.78401
C11	-2.3435	-4.46683	0.658105
H12	-1.5851	-4.44182	1.44604
H13	-3.0059	-5.32316	0.813004
C14	0.32964	-2.96266	-0.27405
N15	0.98401	-1.87155	-0.05726

C3_ZS_Cheltop_INT2

C1	-3.7376	-4.15979	-0.76753
C2	-1.432	-4.92519	-0.55026
C3	-2.6547	-5.02208	-1.49666
C4	-2.9794	-3.68937	0.496529
H5	-4.6097	-4.75812	-0.48455
H6	-2.4282	-4.65487	-2.50254
H7	-3.5991	-3.28239	1.295316
H8	-4.0903	-3.32031	-1.36709
H9	-0.6593	-5.67511	-0.71646
H10	-2.9689	-6.06521	-1.59248
C11	-2.1124	-4.91296	0.8363
H12	-1.4262	-4.73742	1.67059
H13	-2.6975	-5.81664	1.028415
C14	0.47108	-3.26417	-0.02371
N15	0.93988	-2.12147	0.334915

C16	2.32103	-2.22668	0.558691	C16	2.30778	-2.35072	0.940872
C17	2.32208	-3.77854	0.351499	C17	2.52132	-3.87585	0.654105
P18	-1.8892	-0.56048	0.26754	P18	-2.0564	-1.03234	0.131689
Ir19	0.31609	0.07191	-0.64967	Ir19	0.03653	-0.19328	-0.42745
C20	-2.2825	-0.27233	2.032207	C20	-2.5999	-0.70382	1.859106
C21	-2.7077	0.214137	4.76517	C21	-3.2091	-0.1671	4.556167
C22	-2.0092	-1.26689	2.984809	C22	-2.1183	-1.53418	2.882844
C23	-2.765	0.972762	2.468162	C23	-3.4236	0.379044	2.206667
C24	-2.973	1.211003	3.825363	C24	-3.7209	0.644946	3.543337
C25	-2.2257	-1.02519	4.340898	C25	-2.4164	-1.26529	4.218784
H26	-2.9872	1.757406	1.755898	H26	-3.8595	1.000343	1.435235
H27	-3.3438	2.180879	4.143893	H27	-4.3655	1.483966	3.790869
H28	-2.0117	-1.80622	5.064843	H28	-2.0284	-1.91936	4.99442
H29	-2.8743	0.401608	5.822092	H29	-3.4402	0.042843	5.59625
O30	0.9477	-4.10796	-0.00103	O30	1.24134	-4.34086	0.147558
C31	2.35088	-1.93246	2.061578	C31	2.32196	-2.11945	2.453381
C32	1.31717	-1.28778	2.735512	C32	1.23682	-1.59399	3.147793
C33	3.46863	-2.36598	2.792426	C33	3.48614	-2.45099	3.16584
C34	1.39442	-1.07034	4.112693	C34	1.30573	-1.39205	4.528236
H35	0.45896	-0.92485	2.191456	H35	0.352	-1.30103	2.610501
C36	3.54931	-2.14905	4.164828	C36	3.55548	-2.25661	4.542703
H37	4.29237	-2.85675	2.278972	H37	4.35258	-2.8435	2.638747
C38	2.50845	-1.4971	4.831146	C38	2.46192	-1.72291	5.230427
H39	0.57602	-0.56196	4.613712	H39	0.44988	-0.96321	5.042171
H40	4.4252	-2.48433	4.71274	H40	4.4648	-2.51418	5.078011
H41	2.57059	-1.3238	5.901709	H41	2.5182	-1.5645	6.303616
C42	-3.3552	0.064711	-0.66294	C42	-3.5059	-0.69772	-0.93287
C43	-4.6009	0.297382	-0.06158	C43	-4.8176	-0.83564	-0.44913
C44	-3.2508	0.177469	-2.06033	C44	-3.3001	-0.54858	-2.31151
C45	-5.7027	0.670074	-0.83362	C45	-5.8984	-0.81198	-1.32918
H46	-4.7213	0.189345	1.010628	H46	-5.0013	-0.97959	0.610312
C47	-4.3536	0.545513	-2.83013	C47	-4.384	-0.53364	-3.18825
C48	-5.5832	0.802022	-2.2176	C48	-5.6855	-0.66581	-2.7015
H49	-6.6575	0.852884	-0.34896	H49	-6.9075	-0.91888	-0.94148
H50	-4.2526	0.631355	-3.90841	H50	-4.2102	-0.40952	-4.253
H51	-6.4418	1.093309	-2.81522	H51	-6.5283	-0.65375	-3.38593
H52	-0.278	-0.51192	-1.96569	H52	-0.2656	-0.82386	-1.8367
H53	1.72312	0.018467	-1.38447	H53	1.4913	2.42071	-0.29219
H54	-1.6123	-2.22501	2.667749	H54	-1.5102	-2.39589	2.631909
H55	-2.304	-0.02859	-2.54969	H55	-2.2917	-0.43277	-2.6936
H56	2.96013	-4.11436	-0.46819	H56	3.2779	-4.08512	-0.10448
C57	5.38909	-0.18815	-1.74243	C57	4.83632	0.391301	-1.26731
C58	4.64442	-1.2223	-2.31284	C58	4.39103	-0.79103	-1.86378
C59	3.67557	-1.88073	-1.55887	C59	3.62799	-1.69943	-1.13127
C60	3.44125	-1.53001	-0.22158	C60	3.30143	-1.44583	0.207408

C61	4.18192	-0.48283	0.336297	C61	3.75252	-0.25864	0.797945
C62	5.14882	0.182018	-0.4195	C62	4.51625	0.652745	0.064992
H63	6.14786	0.324595	-2.32684	H63	5.43075	1.099959	-1.83643
H64	4.81325	-1.51227	-3.34587	H64	4.63402	-1.00395	-2.90052
H65	3.08002	-2.65579	-2.03404	H65	3.25573	-2.59104	-1.62821
H66	3.99335	-0.17132	1.356091	H66	3.48854	-0.0352	1.825336
H67	5.71955	0.98767	0.034945	H67	4.85908	1.56821	0.539122
C68	1.09976	2.184765	-2.9974	C68	0.61954	4.232777	-0.98429
C69	0.72311	2.029038	-1.51524	C69	0.47036	2.815358	-0.36949
C70	-0.6365	2.027849	-1.07288	C70	-0.3684	1.781021	-1.19394
H71	-1.3414	1.839404	-1.8711	H71	0.12005	1.751331	-2.17207
C72	-1.2567	2.891685	-0.02846	C72	-1.7801	2.237385	-1.43712
C73	-2.494	3.467449	-0.37839	C73	-2.2329	2.490674	-2.74035
C74	-0.7233	3.206348	1.233934	C74	-2.6927	2.435501	-0.39206
C75	-3.1499	4.352326	0.474519	C75	-3.5329	2.932917	-2.98667
H76	-2.9489	3.20921	-1.33088	H76	-1.5608	2.32184	-3.57856
C77	-1.3723	4.106141	2.07915	C77	-3.9919	2.880278	-0.62724
H78	0.15165	2.699014	1.609064	H78	-2.3883	2.202501	0.618987
C79	-2.5826	4.68962	1.704875	C79	-4.4205	3.134903	-1.93038
H80	-4.1035	4.778263	0.176008	H80	-3.8544	3.114044	-4.00859
H81	-0.9381	4.327624	3.050099	H81	-4.6761	3.017064	0.20715
H82	-3.0865	5.385187	2.369746	H82	-5.4339	3.475646	-2.12008
C83	2.16468	4.04194	-0.72817	C83	-0.7229	4.060095	1.656579
C84	2.03844	2.114143	0.546573	C84	-0.0161	1.865865	1.84657
C85	3.2258	4.039402	0.390344	C85	-0.8247	3.565468	3.114851
H86	1.33201	4.726794	-0.5272	H86	-1.7007	4.203193	1.188538
H87	2.59721	4.272823	-1.70367	H87	-0.1655	4.994315	1.586522
H88	4.23325	3.865243	0.001162	H88	-0.0809	4.031958	3.767636
H89	3.21596	4.936214	1.010168	H89	-1.8188	3.68607	3.547654
N90	1.71021	2.651726	-0.64702	N90	-0.0034	2.938811	1.035855
O91	2.87277	2.906447	1.232989	O91	-0.5383	2.144989	3.051984
O92	1.60677	1.047171	1.004686	O92	0.38397	0.719762	1.585328
H93	2.56858	-4.32406	1.260484	H93	2.74333	-4.44132	1.557728
N94	-1.9616	-2.24698	0.126793	N94	-1.9113	-2.72187	0.117564
C95	-1.0863	-3.01881	-0.78487	C95	-0.8895	-3.47394	-0.6391
H96	-1.0701	-2.60576	-1.80287	H96	-0.8168	-3.14834	-1.68664
C97	2.51758	1.68591	-3.30861	C97	1.7652	4.996274	-0.28963
H98	2.82564	2.021413	-4.30402	H98	1.7662	6.048927	-0.58843
H99	3.25446	2.04314	-2.58374	H99	1.72078	4.95623	0.802357
H100	2.55352	0.593142	-3.28845	H100	2.73041	4.567942	-0.58464
C101	0.09302	1.580093	-3.98267	C101	0.90929	4.189731	-2.49148
H102	0.03463	0.495229	-3.85661	H102	1.76381	3.537814	-2.71031
H103	-0.9119	1.998579	-3.87343	H103	0.05217	3.843484	-3.06905
H104	0.4195	1.785086	-5.00638	H104	1.16325	5.193263	-2.84652
H105	1.08242	3.275146	-3.16346	H105	-0.3265	4.771469	-0.84293

H106	1.73351	0.347158	-0.61627
H107	1.51709	0.051438	-1.35887

C3_ZS_Cheltop_TS1

C1	-3.91	-3.63089	-0.75081
C2	-1.6608	-4.58155	-0.77398
C3	-2.9491	-4.49655	-1.63398
C4	-3.0448	-3.3646	0.502611
H5	-4.8186	-4.1791	-0.48216
H6	-2.7627	-4.05438	-2.61798
H7	-3.5858	-3.00428	1.377872
H8	-4.212	-2.69729	-1.23025
H9	-0.9576	-5.36003	-1.06979
H10	-3.3528	-5.49962	-1.79807
C11	-2.2494	-4.67313	0.651305
H12	-1.497	-4.63363	1.444612
H13	-2.8844	-5.55259	0.790649
C14	0.39179	-3.06336	-0.2698
N15	1.00515	-1.95275	-0.04248
C16	2.36602	-2.26487	0.520077
C17	2.41361	-3.82331	0.335154
P18	-1.8945	-0.75864	0.333207
Ir19	0.14087	-0.0026	-0.53731
C20	-2.2189	-0.49817	2.114683
C21	-2.5651	-0.02649	4.858484
C22	-1.9696	-1.51598	3.046768
C23	-2.6428	0.760085	2.572792
C24	-2.8099	0.992894	3.936373
C25	-2.1467	-1.28027	4.410581
H26	-2.8472	1.557901	1.868008
H27	-3.1364	1.971798	4.275951
H28	-1.9523	-2.07779	5.121962
H29	-2.6998	0.155421	5.920827
O30	1.05944	-4.19522	-0.04456
C31	2.45415	-1.95778	2.018061
C32	1.38484	-1.44976	2.750322
C33	3.6483	-2.26713	2.688585
C34	1.50483	-1.22995	4.123914
H35	0.46419	-1.19136	2.25049
C36	3.77094	-2.0491	4.057707
H37	4.49383	-2.66225	2.130435
C38	2.69696	-1.52368	4.781013
H39	0.65927	-0.82146	4.669488
H40	4.7055	-2.28414	4.55893
H41	2.79342	-1.34691	5.848429
C42	-3.3921	-0.08984	-0.50653
C43	-4.6165	0.062927	0.162903

C3_ZS_Cheltop_TS3

C1	-3.7035	-4.30885	-0.77303
C2	-1.3634	-4.96963	-0.53593
C3	-2.5698	-5.10392	-1.50255
C4	-2.9828	-3.82418	0.505637
H5	-4.5503	-4.95318	-0.51435
H6	-2.348	-4.70777	-2.49872
H7	-3.6313	-3.45583	1.300976
H8	-4.088	-3.47261	-1.35855
H9	-0.5572	-5.68285	-0.7048
H10	-2.8339	-6.15837	-1.62245
C11	-2.0631	-5.01059	0.840208
H12	-1.3946	-4.8156	1.684245
H13	-2.6064	-5.94408	1.011308
C14	0.45449	-3.21383	0.034232
N15	0.92178	-2.04434	0.323096
C16	2.28042	-2.22378	0.978105
C17	2.52015	-3.7501	0.736559
P18	-2.1402	-1.11693	0.162751
Ir19	0.07188	-0.24591	-0.52912
C20	-2.6946	-0.81578	1.892165
C21	-3.3157	-0.28866	4.588139
C22	-2.2649	-1.67949	2.910715
C23	-3.4647	0.30364	2.246915
C24	-3.7645	0.568593	3.582774
C25	-2.5749	-1.42012	4.245445
H26	-3.865	0.950268	1.477098
H27	-4.366	1.437612	3.836093
H28	-2.2374	-2.10738	5.016287
H29	-3.5545	-0.08546	5.628127
O30	1.23277	-4.26168	0.29257
C31	2.23057	-1.97755	2.489511
C32	1.08399	-1.55828	3.155965
C33	3.39625	-2.22032	3.233982
C34	1.0966	-1.36228	4.538742
H35	0.18925	-1.34272	2.597543
C36	3.41016	-2.03416	4.613355
H37	4.30485	-2.53728	2.727935
C38	2.25672	-1.59818	5.272162
H39	0.19289	-1.01307	5.030254
H40	4.32185	-2.22224	5.173285
H41	2.26884	-1.44323	6.347447

C44	-3.3451	0.156321	-1.88887	C42	-3.6231	-0.77465	-0.86957
C45	-5.7548	0.481733	-0.52705	C43	-4.9328	-0.91727	-0.3837
H46	-4.6896	-0.14178	1.225516	C44	-3.4315	-0.51578	-2.23415
C47	-4.4852	0.56886	-2.57693	C45	-6.0228	-0.79361	-1.2447
C48	-5.6932	0.740943	-1.89669	H46	-5.1058	-1.12888	0.666886
H49	-6.6916	0.602089	0.009444	C47	-4.5223	-0.39665	-3.09451
H50	-4.43	0.757688	-3.64538	C48	-5.8215	-0.53534	-2.60279
H51	-6.5798	1.068543	-2.43125	H49	-7.0306	-0.90637	-0.85479
H52	-0.4406	-0.58929	-1.8569	H50	-4.3569	-0.19028	-4.14788
H53	1.50773	0.4522	-1.39359	H51	-6.6716	-0.44394	-3.27239
H54	-1.6195	-2.48555	2.709389	H52	0.05352	-1.07572	-1.85972
H55	-2.4149	0.017838	-2.42951	H53	1.5718	2.334211	-0.39753
H56	3.08423	-4.15662	-0.4589	H54	-1.6863	-2.5606	2.658095
C57	5.4102	-0.25761	-1.8489	H55	-2.4253	-0.40569	-2.62573
C58	4.68722	-1.32584	-2.38282	H56	3.2502	-3.97119	-0.04415
C59	3.70013	-1.95102	-1.62236	C57	4.92059	0.446754	-1.18575
C60	3.43373	-1.54002	-0.30877	C58	4.49951	-0.75059	-1.76711
C61	4.13543	-0.4406	0.201231	C59	3.68733	-1.62597	-1.04982
C62	5.11759	0.192437	-0.56215	C60	3.28881	-1.32665	0.258959
H63	6.18631	0.224565	-2.43626	C61	3.70875	-0.121	0.831094
H64	4.89042	-1.67322	-3.39174	C62	4.5211	0.758276	0.114417
H65	3.13399	-2.76498	-2.06745	H63	5.55488	1.130635	-1.74286
H66	3.92273	-0.08528	1.202473	H64	4.79645	-1	-2.78167
H67	5.6645	1.031587	-0.14036	H65	3.33399	-2.52828	-1.54088
C68	1.37409	2.091291	-2.95459	H66	3.38424	0.140654	1.831515
C69	0.90983	1.88153	-1.49978	H67	4.84277	1.688568	0.575181
C70	-0.5246	1.916966	-1.16603	C68	0.79136	4.242575	-1.01953
H71	-1.1501	1.804603	-2.04546	C69	0.58921	2.812254	-0.43721
C72	-1.1164	2.97213	-0.27627	C70	-0.3231	1.908398	-1.32692
C73	-1.943	3.926776	-0.89593	H71	0.14077	1.970092	-2.31207
C74	-0.9013	3.091127	1.105744	C72	-1.7577	2.366717	-1.44575
C75	-2.5104	4.973689	-0.173	C73	-2.2796	2.718212	-2.69791
H76	-2.1423	3.843322	-1.96179	C74	-2.6197	2.400693	-0.34261
C77	-1.4602	4.14685	1.829348	C75	-3.6116	3.109815	-2.83893
H78	-0.3438	2.331837	1.637202	H76	-1.6446	2.665475	-3.57881
C79	-2.2616	5.095292	1.195486	C77	-3.9469	2.802609	-0.47586
H80	-3.1475	5.694178	-0.67819	H78	-2.2617	2.067475	0.62235
H81	-1.2784	4.211956	2.898904	C79	-4.4506	3.15924	-1.72683
H82	-2.6998	5.912006	1.761771	H80	-3.9961	3.366094	-3.82179
C83	2.14994	3.949141	-0.57458	H81	-4.598	2.817878	0.394356
C84	1.88702	2.056346	0.731206	H82	-5.4896	3.455396	-1.83458
C85	3.00501	4.031088	0.704255	C83	-0.5756	4.053318	1.594783
H86	1.24854	4.571071	-0.52483	C84	0.08366	1.84138	1.761543
H87	2.7194	4.201694	-1.47095	C85	-0.6308	3.562215	3.055282
H88	4.0711	3.899721	0.497033	H86	-1.5688	4.197218	1.158361

H89	2.8458	4.941639	1.281978
N90	1.78969	2.528226	-0.53809
O91	2.565	2.904304	1.513238
O92	1.41138	0.995151	1.152714
H93	2.65266	-4.34421	1.261074
N94	-1.9365	-2.43773	0.149965
C95	-1.0294	-3.16641	-0.76805
H96	-1.0343	-2.74172	-1.7813
C97	2.85395	1.750256	-3.15634
H98	3.17849	2.053686	-4.15606
H99	3.50629	2.232909	-2.42473
H100	3.01056	0.671739	-3.06306
C101	0.51847	1.334646	-3.97813
H102	0.57625	0.256293	-3.79968
H103	-0.5332	1.631349	-3.95598
H104	0.89425	1.534529	-4.98554
H105	1.22566	3.169104	-3.12834
X	0	0	0

C3_ZS_Cheltop_INT1

C1	-3.775	-3.59274	-1.4966
C2	-1.6	-4.576	-0.94604
C3	-2.641	-4.49469	-2.09664
C4	-3.2128	-3.26743	-0.09513
H5	-4.7247	-4.13207	-1.42037
H6	-2.211	-4.08094	-3.01426
H7	-3.9209	-2.84378	0.618627
H8	-3.9495	-2.68055	-2.07191
H9	-0.8793	-5.39021	-1.03086
H10	-3.012	-5.49581	-2.33324
C11	-2.5173	-4.58368	0.296334
H12	-1.9747	-4.53444	1.243917
H13	-3.1988	-5.43891	0.312738
C14	0.27475	-3.07158	0.064446
N15	0.8996	-1.96467	0.258982
C16	2.07136	-2.2253	1.156048
C17	1.72484	-3.6687	1.659285
P18	-1.9343	-0.71875	0.014156
Ir19	0.15034	-0.01561	-0.45145
C20	-2.2917	-0.51682	1.80394
C21	-2.5102	-0.16175	4.582705
C22	-2.1097	-1.59938	2.677713
C23	-2.5765	0.749392	2.339839
C24	-2.6882	0.92145	3.719272
C25	-2.2219	-1.42242	4.056431

H87	-0.0169	4.984954	1.505753
H88	0.16253	3.996259	3.671123
H89	-1.5977	3.722531	3.533734
N90	0.1204	2.924472	0.959146
O91	-0.4073	2.131447	2.976296
O92	0.42694	0.688309	1.476885
H93	2.78602	-4.27954	1.649801
N94	-1.9627	-2.80318	0.145774
C95	-0.8905	-3.49354	-0.59803
H96	-0.8179	-3.15922	-1.64275
C97	1.97797	4.926278	-0.31328
H98	2.02045	5.987636	-0.57608
H99	1.93787	4.849801	0.776792
H100	2.91896	4.465253	-0.63377
C101	1.04546	4.257512	-2.5347
H102	1.85864	3.5762	-2.81122
H103	0.15662	3.993632	-3.11055
H104	1.34124	5.264822	-2.84392
H105	-0.1275	4.815356	-0.83933
H106	1.53035	0.14181	-1.03154
H107	-0.5987	0.498669	-1.74381

C3_ZS_Cheltop_PROD

C1	-3.6213	-4.5574	-0.57437
C2	-1.2541	-5.14108	-0.46282
C3	-2.5077	-5.33251	-1.35532
C4	-2.853	-4.05082	0.668342
H5	-4.4425	-5.21751	-0.2763
H6	-2.3564	-4.95179	-2.3703
H7	-3.4746	-3.70869	1.495984
H8	-4.0484	-3.729	-1.14314
H9	-0.4299	-5.82252	-0.67284
H10	-2.7451	-6.397	-1.43767
C11	-1.8718	-5.20119	0.952056
H12	-1.1652	-4.97539	1.756313
H13	-2.3679	-6.15464	1.15455
C14	0.52596	-3.30366	-0.04938
N15	0.98376	-2.10547	0.120783
C16	2.35118	-2.20387	0.777918
C17	2.65831	-3.70554	0.512692
P18	-2.0959	-1.31184	0.254542
Ir19	0.03309	-0.39534	-0.46867
C20	-2.6864	-0.96114	1.957182
C21	-3.3987	-0.27784	4.590768
C22	-2.3166	-1.78124	3.032876

H26	-2.7006	1.605836	1.688882
H27	-2.9045	1.909789	4.114076
H28	-2.0788	-2.27013	4.720446
H29	-2.5918	-0.02442	5.656941
O30	0.70572	-4.14987	0.73142
C31	2.12061	-1.27102	2.341874
C32	1.16527	-0.27809	2.565047
C33	3.13745	-1.43815	3.294206
C34	1.2251	0.547175	3.688947
H35	0.31467	-0.16436	1.897435
C36	3.20038	-0.62263	4.421713
H37	3.89711	-2.19951	3.138383
C38	2.24575	0.377936	4.621669
H39	0.45631	1.29976	3.8319
H40	3.99918	-0.76617	5.143647
H41	2.29524	1.011754	5.502392
C42	-3.3393	0.034811	-0.8805
C43	-4.556	0.352476	-0.26127
C44	-3.2016	0.24118	-2.26412
C45	-5.6077	0.885682	-1.00978
H46	-4.687	0.191005	0.803678
C47	-4.2536	0.771408	-3.00772
C48	-5.459	1.100967	-2.38035
H49	-6.5441	1.132345	-0.51785
H50	-4.1318	0.932844	-4.0749
H51	-6.2772	1.519921	-2.95851
H52	-0.0822	-0.42782	-1.91848
H53	0.91132	1.784722	-2.73638
H54	-1.8654	-2.57814	2.279956
H55	-2.2654	-0.00863	-2.75545
H56	2.5682	-4.35782	1.627458
C57	5.61452	-2.25936	-1.36428
C58	4.64914	-3.2619	-1.47285
C59	3.52544	-3.23837	-0.65018
C60	3.34572	-2.21711	0.294636
C61	4.31147	-1.21164	0.38589
C62	5.439	-1.23556	-0.43504
H63	6.49461	-2.27763	-2.00082
H64	4.77224	-4.06417	-2.19482
H65	2.78407	-4.0268	-0.75387
H66	4.18081	-0.39236	1.080457
H67	6.17878	-0.44483	-0.34753
C68	0.24467	3.811325	-2.79744
C69	0.64965	2.549386	-1.99459
C70	-0.4359	1.924864	-1.06901

C23	-3.4293	0.201321	2.220871
C24	-3.7756	0.543068	3.526847
C25	-2.6769	-1.44454	4.338001
H26	-3.7712	0.823301	1.400316
H27	-4.3557	1.443168	3.712431
H28	-2.3931	-2.09873	5.157753
H29	-3.6751	-0.01601	5.607998
O30	1.35264	-4.30169	0.257148
C31	2.25292	-1.98992	2.29345
C32	1.06243	-1.68253	2.94527
C33	3.42015	-2.152	3.057466
C34	1.03307	-1.51975	4.332137
H35	0.15917	-1.53189	2.376346
C36	3.39236	-1.99753	4.440049
H37	4.3615	-2.37824	2.56322
C38	2.19407	-1.67621	5.083893
H39	0.09528	-1.25791	4.812808
H40	4.30622	-2.12084	5.014128
H41	2.17192	-1.54749	6.162318
C42	-3.5577	-0.89683	-0.78122
C43	-4.8734	-1.12257	-0.33984
C44	-3.3566	-0.36561	-2.06368
C45	-5.9573	-0.80904	-1.15753
H46	-5.0523	-1.53001	0.651097
C47	-4.4431	-0.04813	-2.87993
C48	-5.7451	-0.2659	-2.42841
H49	-6.9686	-0.98557	-0.80179
H50	-4.2701	0.382785	-3.86154
H51	-6.5917	-0.01386	-3.06032
H52	-0.1584	-1.05495	-1.86805
H53	1.34781	2.234774	-0.39873
H54	-1.7434	-2.68413	2.852234
H55	-2.3465	-0.18672	-2.41827
H56	3.27379	-3.88478	-0.37115
C57	4.93522	0.651001	-1.21606
C58	4.60069	-0.54924	-1.84499
C59	3.79966	-1.48257	-1.1896
C60	3.32752	-1.23865	0.105945
C61	3.65757	-0.02607	0.723652
C62	4.45732	0.909963	0.068982
H63	5.56281	1.377733	-1.7244
H64	4.95785	-0.75778	-2.8493
H65	3.51775	-2.38689	-1.72037
H66	3.26816	0.198287	1.709534
H67	4.70763	1.843067	0.566961

H71	-1.3187	1.829074	-1.69939
C72	-0.8148	2.793762	0.11024
C73	-2.0258	3.50798	0.11357
C74	-0.0114	2.888425	1.263902
C75	-2.4148	4.282545	1.209213
H76	-2.6869	3.435027	-0.74554
C77	-0.3935	3.663187	2.359049
H78	0.91795	2.330142	1.329558
C79	-1.6007	4.365574	2.34082
H80	-3.3595	4.818627	1.178883
H81	0.25245	3.711555	3.231972
H82	-1.9	4.969362	3.192456
C83	2.38703	4.097468	-0.71774
C84	2.53266	1.807914	-0.61935
C85	3.74365	3.656882	-0.1421
H86	1.70413	4.476919	0.049998
H87	2.49937	4.848359	-1.49948
H88	4.56114	3.785301	-0.8585
H89	3.99967	4.132581	0.804692
N90	1.90339	2.813137	-1.24497
O91	3.58266	2.230963	0.10227
O92	2.22367	0.598981	-0.63056
H93	1.27842	-3.65946	2.656875
N94	-2.0344	-2.37767	-0.26651
C95	-0.9318	-3.18544	-0.83842
H96	-0.6232	-2.81404	-1.82383
C97	1.30797	4.137455	-3.86368
H98	1.09238	5.09622	-4.34492
H99	2.32526	4.183004	-3.46521
H100	1.30525	3.366674	-4.64332
C101	-1.1118	3.628601	-3.49809
H102	-1.1178	2.713879	-4.10384
H103	-1.9466	3.574113	-2.79782
H104	-1.2995	4.470655	-4.17154
H105	0.14957	4.653262	-2.09788

C3_ZS_Cheltop_TS2

C1	-3.7143	-4.14033	-0.8828
C2	-1.4083	-4.90543	-0.6499
C3	-2.6176	-4.98212	-1.61608
C4	-2.9741	-3.69225	0.399187
H5	-4.5862	-4.7492	-0.62197
H6	-2.3775	-4.58913	-2.60899
H7	-3.6053	-3.30213	1.197983
H8	-4.0649	-3.29063	-1.46945
C68	0.87419	4.214919	-1.05562
C69	0.44656	2.849626	-0.47657
C70	-0.5474	2.055606	-1.36904
H71	-0.0648	1.780351	-2.30429
C72	-1.9007	2.688943	-1.6101
C73	-2.225	3.273979	-2.83797
C74	-2.8715	2.662637	-0.59931
C75	-3.4831	3.847623	-3.03709
H76	-1.5019	3.272745	-3.64835
C77	-4.1279	3.22718	-0.7953
H78	-2.6421	2.16818	0.338948
C79	-4.4356	3.82767	-2.01835
H80	-3.7209	4.301139	-3.99495
H81	-4.8714	3.184382	-0.00421
H82	-5.4163	4.265217	-2.17993
C83	-0.7744	4.114747	1.496899
C84	-0.0349	1.923297	1.754138
C85	-0.7938	3.685413	2.978016
H86	-1.7763	4.220352	1.071959
H87	-0.2386	5.05419	1.353575
H88	-0.003	4.167413	3.56121
H89	-1.7566	3.844177	3.465583
N90	-0.0616	2.978957	0.900925
O91	-0.5332	2.259895	2.958356
O92	0.36848	0.780295	1.524938
H93	3.0907	-4.20746	1.375865
N94	-1.8889	-2.99336	0.264407
C95	-0.8489	-3.64742	-0.56643
H96	-0.8774	-3.30971	-1.61198
C97	2.01826	4.811874	-0.21628
H98	2.26559	5.817725	-0.56815
H99	1.78557	4.878083	0.849715
H100	2.91691	4.19181	-0.31401
C101	1.34846	4.074443	-2.51218
H102	2.12025	3.300162	-2.59753
H103	0.53734	3.8253	-3.19878
H104	1.78436	5.017481	-2.85527
H105	0.00858	4.891409	-1.03911
H106	1.4367	0.080955	-1.06961
H107	-0.9092	1.117059	-0.8077

H9	-0.6332	-5.65227	-0.81928
H10	-2.9281	-6.02304	-1.74268
C11	-2.1078	-4.92027	0.726849
H12	-1.4328	-4.7599	1.573234
H13	-2.6939	-5.82809	0.894533
C14	0.47555	-3.24371	-0.04475
N15	0.93222	-2.0996	0.326698
C16	2.28836	-2.32164	0.953298
C17	2.48668	-3.86376	0.73203
P18	-2.036	-1.02985	0.090818
Ir19	-0.0368	-0.18229	-0.38279
C20	-2.5787	-0.729	1.819034
C21	-3.1834	-0.25152	4.521759
C22	-2.1051	-1.58941	2.821706
C23	-3.3872	0.358137	2.189081
C24	-3.6831	0.594244	3.530743
C25	-2.4021	-1.34987	4.162232
H26	-3.8101	1.003853	1.430901
H27	-4.3162	1.434914	3.800715
H28	-2.0214	-2.02446	4.923506
H29	-3.413	-0.06428	5.56669
O30	1.23696	-4.32155	0.152152
C31	2.30125	-2.04126	2.456965
C32	1.20836	-1.52504	3.144915
C33	3.47157	-2.34201	3.173528
C34	1.27524	-1.30307	4.522412
H35	0.31551	-1.2609	2.605322
C36	3.53868	-2.12984	4.5474
H37	4.34295	-2.72754	2.64948
C38	2.43541	-1.60842	5.22911
H39	0.41213	-0.88345	5.031633
H40	4.4525	-2.36569	5.085033
H41	2.48798	-1.43655	6.300413
C42	-3.4697	-0.67781	-0.98697
C43	-4.7832	-0.7922	-0.4999
C44	-3.2634	-0.57599	-2.37032
C45	-5.8636	-0.79377	-1.38131
H46	-4.9704	-0.90249	0.562553
C47	-4.3468	-0.5885	-3.24704
C48	-5.6495	-0.69661	-2.7572
H49	-6.8729	-0.88165	-0.98963
H50	-4.172	-0.50401	-4.31545
H51	-6.4918	-0.70494	-3.44229
H52	-0.2787	-0.78902	-1.81011
H53	1.43881	2.429561	-0.32464

H54	-1.5043	-2.44942	2.549775
H55	-2.2541	-0.48025	-2.75561
H56	3.2919	-4.11465	0.039888
C57	5.1019	0.120271	-1.27686
C58	4.59336	-1.05562	-1.83196
C59	3.69896	-1.84001	-1.10602
C60	3.31089	-1.47431	0.190012
C61	3.81533	-0.28716	0.733946
C62	4.70408	0.505296	0.003513
H63	5.79996	0.732486	-1.84023
H64	4.88896	-1.35984	-2.83171
H65	3.28978	-2.73349	-1.57
H66	3.50683	0.02292	1.725967
H67	5.08999	1.421237	0.442275
C68	0.54595	4.22739	-1.02625
C69	0.41615	2.821715	-0.3813
C70	-0.4489	1.773788	-1.16305
H71	0.03277	1.7035	-2.14425
C72	-1.8553	2.249838	-1.40081
C73	-2.3329	2.471906	-2.70036
C74	-2.7402	2.497692	-0.34239
C75	-3.6312	2.928917	-2.92976
H76	-1.6837	2.265369	-3.54805
C77	-4.0373	2.956311	-0.56005
H78	-2.4124	2.288285	0.66623
C79	-4.4913	3.177453	-1.86091
H80	-3.9738	3.083971	-3.94904
H81	-4.6989	3.129918	0.28563
H82	-5.5029	3.529795	-2.03864
C83	-0.7053	4.072239	1.694843
C84	0.07568	1.899275	1.852638
C85	-0.7141	3.576553	3.158937
H86	-1.7099	4.204066	1.283988
H87	-0.165	5.013656	1.591363
H88	0.04773	4.06749	3.771345
H89	-1.6877	3.666554	3.642613
N90	-0.0067	2.960858	1.036051
O91	-0.3848	2.164354	3.085363
O92	0.51692	0.773671	1.563023
H93	2.6297	-4.40009	1.669535
N94	-1.9021	-2.71692	0.047217
C95	-0.8653	-3.45327	-0.70251
H96	-0.7697	-3.10747	-1.74133
C97	1.70015	5.011922	-0.37121
H98	1.6917	6.056384	-0.69739

H99	1.67375	5.001517	0.721877
H100	2.66208	4.579516	-0.67072
C101	0.80414	4.155383	-2.53824
H102	1.65702	3.502998	-2.76106
H103	-0.0633	3.793635	-3.09019
H104	1.04567	5.152635	-2.91877
H105	-0.4016	4.761621	-0.87492
H106	2.24919	0.591353	-1.18911
H107	1.9958	0.311567	-1.83756

C3_ZR_Cheltop

C3_ZR_Cheltop_SM

C1	-3.42767	-4.99928	0.479053
C2	-1.07632	-5.06677	-0.16005
C3	-2.47179	-5.55021	-0.62902
C4	-2.44773	-4.25965	1.419601
H5	-3.92578	-5.80825	1.023599
H6	-2.72446	-5.17828	-1.6271
H7	-2.84639	-4.00546	2.401367
H8	-4.2017	-4.34184	0.084042
H9	-0.23432	-5.58061	-0.62223
H10	-2.49288	-6.64278	-0.67149
C11	-1.20833	-5.16983	1.375254
H12	-0.35672	-4.76126	1.92764
H13	-1.41156	-6.18957	1.714036
C14	0.343637	-2.90881	-0.32145
N15	0.617797	-1.65944	-0.16291
C16	2.110915	-1.46655	-0.38425
C17	2.587364	-2.95173	-0.44308
P18	-2.54607	-1.49083	0.626891
Ir19	-0.74214	0.008598	-0.05545
C20	-3.15637	-1.11929	2.307915
C21	-3.81411	-0.25983	4.904451
C22	-2.39589	-1.53501	3.413806
C23	-4.24099	-0.25313	2.520374
C24	-4.56959	0.166839	3.81054
C25	-2.7274	-1.1138	4.700168
H26	-4.83341	0.102073	1.683925
H27	-5.42115	0.825411	3.95831
H28	-2.12771	-1.44353	5.543398
H29	-4.07096	0.067071	5.907833
O30	1.367497	-3.73463	-0.55405
C31	2.786102	-0.80053	0.808679
C32	2.180973	-0.81334	2.068814
C33	4.088303	-0.29117	0.687809

C3_ZR_Cheltop_INT2

C1	-4.04825	-3.70792	1.012992
C2	-1.9792	-4.72145	0.235016
C3	-3.50205	-4.72514	-0.04046
C4	-2.76	-3.26768	1.751572
H5	-4.73054	-4.18775	1.722404
H6	-3.73764	-4.43444	-1.06916
H7	-2.92599	-2.76692	2.70453
H8	-4.57931	-2.87114	0.561246
H9	-1.42924	-5.55957	-0.19259
H10	-3.90919	-5.72776	0.118339
C11	-1.93794	-4.56798	1.771924
H12	-0.93228	-4.43476	2.181759
H13	-2.44246	-5.38834	2.290096
C14	0.068039	-3.27072	-0.36372
N15	0.725981	-2.17015	-0.4416
C16	2.191876	-2.51519	-0.31192
C17	2.14716	-4.02739	-0.67101
P18	-1.95202	-0.75675	0.642316
Ir19	-0.13697	-0.13893	-0.6763
C20	-1.93831	-0.0852	2.3461
C21	-1.7045	1.084397	4.891056
C22	-1.29752	-0.77028	3.389358
C23	-2.46	1.194817	2.593705
C24	-2.34871	1.77067	3.858899
C25	-1.1837	-0.18811	4.651593
H26	-2.95836	1.740253	1.800765
H27	-2.77457	2.754057	4.039657
H28	-0.68912	-0.73362	5.450106
H29	-1.61662	1.534331	5.875428
O30	0.770934	-4.407	-0.37184
C31	2.638369	-2.38495	1.151956
C32	1.760272	-2.02653	2.171628
C33	3.968007	-2.69164	1.478231

C34	2.831787	-0.26469	3.174009	C34	2.199799	-1.95382	3.494336
H35	1.186292	-1.22162	2.187385	H35	0.741626	-1.77114	1.927233
C36	4.743273	0.244827	1.795902	C36	4.406276	-2.62908	2.798097
H37	4.590628	-0.3026	-0.27381	H37	4.668203	-2.96374	0.692386
C38	4.112107	0.272108	3.041183	C38	3.52112	-2.25554	3.813298
H39	2.330197	-0.25528	4.13737	H39	1.505488	-1.6454	4.270714
H40	5.747219	0.644208	1.68376	H40	5.43973	-2.86558	3.034388
H41	4.619502	0.699724	3.901403	H41	3.864698	-2.1986	4.842115
C42	-4.02203	-1.64208	-0.45836	C42	-3.64214	-0.4778	-0.01453
C43	-5.29444	-2.01298	0.008349	C43	-4.74183	-0.23665	0.822926
C44	-3.81647	-1.51141	-1.841	C44	-3.86232	-0.69748	-1.38419
C45	-6.33915	-2.22659	-0.89005	C45	-6.03139	-0.18926	0.293165
H46	-5.47045	-2.14381	1.071482	H46	-4.60221	-0.09367	1.888864
C47	-4.86112	-1.73847	-2.73778	C47	-5.1529	-0.66211	-1.90685
C48	-6.12624	-2.09063	-2.26467	C48	-6.24053	-0.40201	-1.07004
H49	-7.32008	-2.50596	-0.5161	H49	-6.87389	0.007151	0.949963
H50	-4.68747	-1.63125	-3.80473	H50	-5.30811	-0.82795	-2.96891
H51	-6.94214	-2.25963	-2.96111	H51	-7.24611	-0.36635	-1.47853
H52	-0.9641	-0.20249	-1.58421	H52	-1.02533	-0.56929	-1.89993
H53	0.603721	0.70044	-0.51552	H53	1.82407	1.934043	-1.0084
H54	-1.52663	-2.16616	3.26072	H54	-0.88531	-1.75781	3.218586
H55	-2.83929	-1.21677	-2.21048	H55	-3.02059	-0.87606	-2.0449
H56	3.22176	-3.17837	-1.29987	H56	2.320471	-4.24483	-1.72748
C57	2.321582	0.587778	-4.21352	C57	4.2689	0.170129	-3.02006
C58	2.014686	-0.77314	-4.14429	C58	3.673177	-0.99382	-3.51075
C59	1.991402	-1.42516	-2.91217	C59	3.050436	-1.8898	-2.63963
C60	2.276879	-0.73721	-1.72451	C60	3.017315	-1.6454	-1.26051
C61	2.575563	0.630247	-1.80541	C61	3.620658	-0.47315	-0.77729
C62	2.601756	1.284365	-3.03829	C62	4.241509	0.424243	-1.64805
H63	2.343903	1.096566	-5.17272	H63	4.749803	0.86802	-3.6988
H64	1.793317	-1.32933	-5.0507	H64	3.687318	-1.20558	-4.57593
H65	1.727386	-2.47966	-2.88384	H65	2.564479	-2.76438	-3.06046
H66	2.779374	1.200601	-0.90674	H66	3.573091	-0.24459	0.280608
H67	2.838969	2.344021	-3.07225	H67	4.693874	1.328521	-1.25091
C68	-3.37037	1.810127	-0.49846	C68	-1.53135	2.731533	-1.54233
C69	-1.99558	1.794022	0.208331	C69	-0.35681	2.037547	-0.81149
C70	-0.78518	2.165951	-0.47248	C70	1.018176	2.528895	-1.44575
H71	-0.86157	2.263781	-1.55008	H71	1.004688	2.293321	-2.5135
C72	0.237111	3.104047	0.106612	C72	1.400541	3.97954	-1.24622
C73	1.243911	2.754967	1.014981	C73	2.261755	4.32726	-0.1933
C74	0.141255	4.441415	-0.31288	C74	0.934449	5.003921	-2.08309
C75	2.120615	3.724479	1.50613	C75	2.607977	5.657297	0.048605
H76	1.355637	1.725691	1.333058	H76	2.665509	3.542453	0.443958
C77	1.016515	5.408282	0.179155	C77	1.277677	6.336352	-1.845
H78	-0.62742	4.723938	-1.02925	H78	0.306494	4.75673	-2.9353

C79	2.00888	5.052267	1.095543
H80	2.896732	3.427687	2.205952
H81	0.926633	6.437406	-0.15734
H82	2.693689	5.804302	1.476796
C83	-2.26456	3.417386	2.256212
C84	-1.16508	1.414925	2.476444
C85	-2.01	3.089677	3.732503
H86	-3.28111	3.76281	2.094297
H87	-1.56032	4.155563	1.857353
H88	-1.53896	3.897483	4.293031
H89	-2.91812	2.750595	4.240893
N90	-2.01116	2.090075	1.649971
O91	-1.08719	1.971033	3.688848
O92	-0.50664	0.407783	2.185037
H93	3.084848	-3.25259	0.480306
N94	-1.87492	-3.04733	0.762315
C95	-1.03227	-3.52829	-0.359
H96	-1.45464	-3.26274	-1.34117
C97	-3.25087	1.834973	-2.02974
H98	-2.56416	1.075944	-2.40638
H99	-4.23217	1.644289	-2.47331
H100	-2.91182	2.814856	-2.38537
C101	-4.30004	2.959375	-0.06328
H102	-4.6357	2.846688	0.970915
H103	-3.82318	3.939212	-0.18037
H104	-5.19623	2.954035	-0.69179
H105	-3.88348	0.884143	-0.22924
X	-1.52878	1.835509	-0.4773

C3_ZR_Cheltop_TS1

C1	-3.33943	-5.09715	0.414086
C2	-0.97801	-5.11036	-0.19421
C3	-2.35639	-5.62371	-0.6825
C4	-2.38775	-4.34241	1.371528
H5	-3.83085	-5.91819	0.946416
H6	-2.60298	-5.25479	-1.68321
H7	-2.80471	-4.10025	2.348941
H8	-4.11906	-4.44978	0.011861
H9	-0.11802	-5.60225	-0.64709
H10	-2.35363	-6.71632	-0.72803
C11	-1.12682	-5.2235	1.338969
H12	-0.29169	-4.79687	1.902811
H13	-1.30882	-6.24949	1.670621
C14	0.392069	-2.91542	-0.32938
N15	0.620199	-1.65852	-0.17185

C79	2.10584	6.670413	-0.77131
H80	3.277641	5.901939	0.868949
H81	0.902927	7.114462	-2.50418
H82	2.372863	7.706937	-0.58776
C83	-0.58832	3.745983	1.265506
C84	0.53149	1.732297	1.411706
C85	0.326214	3.700887	2.512399
H86	-1.64203	3.80595	1.54916
H87	-0.34771	4.578185	0.604267
H88	1.166075	4.395125	2.439018
H89	-0.21444	3.858501	3.44654
N90	-0.29666	2.44947	0.634061
O91	0.867303	2.349787	2.546083
O92	0.966781	0.59557	1.128987
H93	2.805421	-4.63385	-0.05179
N94	-1.87523	-2.44395	0.869288
C95	-1.42839	-3.34704	-0.22671
H96	-1.85503	-3.06363	-1.20001
C97	-2.8909	2.719906	-0.8342
H98	-2.87489	3.229021	0.131039
H99	-3.6279	3.24399	-1.4519
H100	-3.25767	1.708994	-0.68442
C101	-1.70994	2.189399	-2.97052
H102	-0.77746	2.178565	-3.54159
H103	-2.09496	1.166793	-2.94353
H104	-2.42572	2.808119	-3.52152
H105	-1.25057	3.790536	-1.63366
H106	1.39454	0.10461	-1.54425
H107	0.813622	0.006655	-2.13567

C3_ZR_Cheltop_TS3

C1	-4.09624	-3.79857	0.772442
C2	-1.96725	-4.7499	0.07422
C3	-3.46996	-4.74996	-0.29848
C4	-2.86148	-3.38455	1.610061
H5	-4.81353	-4.32526	1.410508
H6	-3.6418	-4.40382	-1.32286
H7	-3.08931	-2.93089	2.574517
H8	-4.6113	-2.94377	0.335428
H9	-1.38322	-5.56176	-0.35878
H10	-3.8749	-5.7632	-0.22387
C11	-2.02593	-4.67589	1.616452
H12	-1.04964	-4.55571	2.095247
H13	-2.55171	-5.52689	2.058451
C14	0.100802	-3.2539	-0.32013

C16	2.106337	-1.41687	-0.33658	N15	0.753395	-2.15026	-0.39112
C17	2.638021	-2.88274	-0.48067	C16	2.222503	-2.46804	-0.2396
P18	-2.51562	-1.5642	0.630303	C17	2.203716	-4.01353	-0.46457
Ir19	-0.84634	-0.04572	-0.08317	P18	-2.03999	-0.81503	0.677837
C20	-3.04679	-1.20148	2.336987	Ir19	-0.07992	-0.10995	-0.65665
C21	-3.60158	-0.35085	4.95183	C20	-2.00301	-0.20454	2.39839
C22	-2.27038	-1.65748	3.414224	C21	-1.74116	0.878128	4.971821
C23	-4.08611	-0.29066	2.58215	C22	-1.42157	-0.95701	3.427685
C24	-4.36473	0.125249	3.884773	C23	-2.4485	1.099873	2.66954
C25	-2.55362	-1.24221	4.712948	C24	-2.32114	1.633641	3.950836
H26	-4.68164	0.094648	1.760244	C25	-1.2949	-0.41688	4.707381
H27	-5.18248	0.817671	4.064607	H26	-2.90121	1.694396	1.882905
H28	-1.94784	-1.60474	5.538057	H27	-2.68393	2.637625	4.154425
H29	-3.81954	-0.02665	5.96526	H28	-0.84917	-1.01204	5.499112
O30	1.44439	-3.71097	-0.5451	H29	-1.64175	1.295621	5.969482
C31	2.727112	-0.79952	0.912217	O30	0.808812	-4.38499	-0.26402
C32	2.070743	-0.8553	2.144522	C31	2.677047	-2.21286	1.204081
C33	4.030926	-0.28323	0.861857	C32	1.764975	-1.96687	2.22875
C34	2.675384	-0.34096	3.292568	C33	4.038554	-2.33413	1.526464
H35	1.072257	-1.26702	2.206602	C34	2.197608	-1.80383	3.545229
C36	4.638924	0.219522	2.010642	H35	0.715997	-1.86567	1.995816
H37	4.570297	-0.26218	-0.08042	C36	4.471342	-2.17987	2.841513
C38	3.957027	0.20395	3.229476	H37	4.764671	-2.5359	0.744174
H39	2.135542	-0.36345	4.234653	C38	3.550915	-1.90602	3.857046
H40	5.64438	0.626389	1.951287	H39	1.470341	-1.57954	4.320438
H41	4.427151	0.605615	4.122642	H40	5.529069	-2.26697	3.073453
C42	-4.05985	-1.67954	-0.35768	H41	3.89043	-1.77369	4.880508
C43	-5.27456	-2.11585	0.200088	C42	-3.74492	-0.49473	0.072961
C44	-3.99523	-1.42923	-1.73666	C43	-4.82354	-0.25152	0.935842
C45	-6.3991	-2.28454	-0.60591	C44	-3.99094	-0.64374	-1.30171
H46	-5.34384	-2.32261	1.263816	C45	-6.11778	-0.13451	0.42735
C47	-5.12146	-1.60705	-2.5414	H46	-4.66272	-0.15479	2.004265
C48	-6.32521	-2.03358	-1.97873	C47	-5.28506	-0.53818	-1.80477
H49	-7.33316	-2.6165	-0.16186	C48	-6.3516	-0.27665	-0.94084
H50	-5.05835	-1.40615	-3.60696	H49	-6.94456	0.063959	1.103522
H51	-7.20326	-2.16649	-2.60351	H50	-5.46076	-0.64755	-2.87088
H52	-1.10952	-0.32876	-1.59764	H51	-7.36006	-0.1832	-1.33324
H53	0.335167	0.868692	-0.62722	H52	-1.08873	-0.56604	-1.78024
H54	-1.43134	-2.32036	3.228584	H53	1.891189	1.968537	-1.04992
H55	-3.06421	-1.08337	-2.17291	H54	-1.06356	-1.9611	3.230689
H56	3.221172	-3.05305	-1.38629	H55	-3.1656	-0.82112	-1.98465
C57	2.269449	0.878293	-4.02202	H56	2.484442	-4.32665	-1.47214
C58	1.967556	-0.48551	-4.03701	C57	4.260118	-0.1143	-3.27425
C59	1.967269	-1.21442	-2.85008	C58	3.575202	-1.27978	-3.62474
C60	2.27396	-0.6028	-1.62601	C59	2.956635	-2.05534	-2.64309

C61	2.571226	0.765302	-1.62362
C62	2.570839	1.499309	-2.81154
H63	2.268936	1.448597	-4.9465
H64	1.727816	-0.98071	-4.97356
H65	1.698283	-2.26755	-2.88231
H66	2.78857	1.276851	-0.69506
H67	2.80055	2.560747	-2.78018
C68	-3.28177	1.908127	-0.69198
C69	-1.93526	1.80131	0.064831
C70	-0.65601	2.111446	-0.59181
H71	-0.71565	2.212906	-1.67131
C72	0.341703	3.069449	0.001052
C73	1.28685	2.730358	0.975421
C74	0.287484	4.392216	-0.4644
C75	2.153678	3.699991	1.483453
H76	1.358014	1.708212	1.329438
C77	1.149419	5.360203	0.048422
H78	-0.43687	4.662928	-1.22956
C79	2.085218	5.016228	1.027137
H80	2.886499	3.414159	2.23254
H81	1.094581	6.380506	-0.3206
H82	2.760796	5.769357	1.422225
C83	-2.2004	3.519579	2.058818
C84	-1.18666	1.485621	2.36388
C85	-1.98614	3.228868	3.549703
H86	-3.20001	3.897031	1.867719
H87	-1.46327	4.221609	1.655202
H88	-1.49412	4.035386	4.093909
H89	-2.91608	2.943834	4.05209
N90	-1.98398	2.165674	1.495123
O91	-1.10817	2.074036	3.560048
O92	-0.55897	0.446862	2.115617
H93	3.208414	-3.19737	0.394493
N94	-1.83826	-3.11703	0.723071
C95	-0.96755	-3.57092	-0.38788
H96	-1.38236	-3.30885	-1.37408
C97	-3.10726	1.888518	-2.21924
H98	-2.44655	1.087631	-2.55661
H99	-4.08111	1.739719	-2.69468
H100	-2.70713	2.84206	-2.58372
C101	-4.15499	3.123543	-0.32364
H102	-4.54282	3.057081	0.696048
H103	-3.61416	4.069987	-0.43916
H104	-5.02275	3.159889	-0.99003
H105	-3.85779	1.018983	-0.41679

C60	3.017476	-1.69142	-1.29193
C61	3.697581	-0.51202	-0.95278
C62	4.315758	0.266239	-1.93279
H63	4.743556	0.488675	-4.03726
H64	3.519535	-1.58789	-4.6649
H65	2.403319	-2.93669	-2.95337
H66	3.728581	-0.18839	0.08063
H67	4.835855	1.175244	-1.6435
C68	-1.47732	2.720463	-1.55914
C69	-0.28132	2.062959	-0.8288
C70	1.078998	2.56529	-1.47042
H71	1.05324	2.326705	-2.53699
C72	1.454195	4.016416	-1.26366
C73	2.341045	4.345526	-0.22618
C74	0.951858	5.057448	-2.05817
C75	2.677745	5.671707	0.045382
H76	2.771569	3.546708	0.375214
C77	1.282564	6.387146	-1.7884
H78	0.303506	4.827783	-2.89978
C79	2.136663	6.699753	-0.72912
H80	3.367145	5.90235	0.853432
H81	0.877345	7.180761	-2.41004
H82	2.391449	7.734615	-0.51921
C83	-0.53115	3.793354	1.238435
C84	0.456759	1.726906	1.458604
C85	0.357897	3.727406	2.499569
H86	-1.59067	3.865638	1.499945
H87	-0.26562	4.623769	0.584586
H88	1.266215	4.325873	2.398713
H89	-0.16837	3.989395	3.418094
N90	-0.24742	2.489803	0.614725
O91	0.757395	2.329573	2.607475
O92	0.814764	0.546596	1.230735
H93	2.801491	-4.55052	0.27019
N94	-1.93989	-2.50692	0.822906
C95	-1.40234	-3.35031	-0.28064
H96	-1.76522	-3.02458	-1.26597
C97	-2.82108	2.670843	-0.8252
H98	-2.81168	3.213121	0.122037
H99	-3.59913	3.130954	-1.44322
H100	-3.12812	1.648139	-0.63032
C101	-1.66111	2.16241	-2.9805
H102	-0.73353	2.156427	-3.55945
H103	-2.03567	1.135729	-2.94205
H104	-2.39001	2.766065	-3.53089

X	-1.52878	1.835509	-0.4773

C3_ZR_Cheltop_INT1

C1	-4.16881	-3.50899	0.506112
C2	-2.03965	-4.58626	0.001888
C3	-3.51107	-4.52878	-0.48183
C4	-2.9837	-3.1361	1.427926
H5	-4.96996	-3.97141	1.091729
H6	-3.59065	-4.21593	-1.52784
H7	-3.25526	-2.6401	2.359979
H8	-4.58635	-2.63467	0.004602
H9	-1.46428	-5.43527	-0.3664
H10	-3.96976	-5.51827	-0.40083
C11	-2.21201	-4.46334	1.532112
H12	-1.27078	-4.37058	2.082655
H13	-2.80734	-5.27735	1.954972
C14	0.114089	-3.1731	-0.29997
N15	0.804969	-2.09257	-0.21828
C16	2.259598	-2.46234	-0.18792
C17	2.188668	-3.99546	-0.51573
P18	-1.90602	-0.64746	0.535682
Ir19	0.002896	-0.04179	-0.45029
C20	-1.90991	-0.03114	2.248604
C21	-1.52752	1.058013	4.799838
C22	-1.39165	-0.82106	3.283906
C23	-2.22585	1.314009	2.498288
C24	-2.03754	1.85158	3.770072
C25	-1.2085	-0.27796	4.555018
H26	-2.60555	1.942061	1.69866
H27	-2.28875	2.891798	3.958427
H28	-0.80947	-0.89826	5.352441
H29	-1.37646	1.481025	5.788596
O30	0.776185	-4.32882	-0.39754
C31	2.838274	-2.26456	1.212629
C32	2.074336	-1.77405	2.271891
C33	4.164632	-2.6538	1.459255
C34	2.633082	-1.63801	3.5445
H35	1.049782	-1.47556	2.10786
C36	4.72069	-2.52427	2.728843
H37	4.771542	-3.05021	0.649063
C38	3.955718	-2.00739	3.777933
H39	2.02495	-1.2334	4.348859
H40	5.750873	-2.82431	2.898472
H41	4.390405	-1.89956	4.767593
C42	-3.50326	-0.16208	-0.21818
H105	-1.22423	3.785339	-1.66326
H106	1.373267	0.154683	-1.24307
H107	0.360374	-0.09257	-2.17009

C3_ZR_Cheltop_PROD

C1	-4.09679	-4.04262	0.439749
C2	-1.88516	-4.92213	-0.08026
C3	-3.34824	-4.951	-0.59184
C4	-2.96634	-3.66201	1.426152
H5	-4.88842	-4.58965	0.961883
H6	-3.43607	-4.5877	-1.62081
H7	-3.307	-3.26592	2.382567
H8	-4.54804	-3.15841	-0.01427
H9	-1.23401	-5.69158	-0.49552
H10	-3.72954	-5.97602	-0.57337
C11	-2.09654	-4.93151	1.450527
H12	-1.17529	-4.80856	2.027391
H13	-2.63319	-5.82057	1.793913
C14	0.13068	-3.28926	-0.27381
N15	0.723453	-2.1401	-0.32536
C16	2.205187	-2.35396	-0.05016
C17	2.289334	-3.87585	-0.35086
P18	-2.03108	-1.0282	0.755381
Ir19	-0.18	-0.35528	-0.65146
C20	-1.9677	-0.52344	2.517177
C21	-1.71841	0.420869	5.153291
C22	-1.65892	-1.40505	3.560874
C23	-2.13341	0.84145	2.807367
C24	-2.00812	1.308713	4.113448
C25	-1.54512	-0.93422	4.871653
H26	-2.36517	1.539226	2.006855
H27	-2.14244	2.366324	4.323716
H28	-1.31461	-1.63188	5.67206
H29	-1.62629	0.783657	6.172535
O30	0.922281	-4.35298	-0.15835
C31	2.51758	-2.1429	1.43766
C32	1.560097	-1.77272	2.378275
C33	3.831013	-2.38841	1.869086
C34	1.906213	-1.63164	3.724124
H35	0.546193	-1.56853	2.070045
C36	4.176078	-2.25611	3.210286
H37	4.592584	-2.66627	1.145068
C38	3.210949	-1.87235	4.145106
H39	1.146275	-1.32352	4.435216
H40	5.198119	-2.447	3.524688

C43	-4.6144	0.201497	0.557499
C44	-3.65872	-0.32131	-1.60454
C45	-5.85035	0.428996	-0.05031
H46	-4.52313	0.305212	1.63355
C47	-4.89591	-0.10075	-2.20576
C48	-5.99448	0.282053	-1.43087
H49	-6.70158	0.718329	0.559175
H50	-5.0018	-0.2207	-3.27995
H51	-6.95711	0.461151	-1.90019
H52	-0.58754	-0.49503	-1.83036
H53	1.675255	0.601349	-1.35197
H54	-1.11229	-1.85157	3.090307
H55	-2.80604	-0.60918	-2.21116
H56	2.501406	-4.23505	-1.53463
C57	4.143622	-0.2551	-3.42499
C58	3.070523	-1.118	-3.66719
C59	2.495079	-1.82451	-2.61526
C60	2.97756	-1.6885	-1.30213
C61	4.041405	-0.81267	-1.07016
C62	4.621998	-0.10402	-2.12598
H63	4.594497	0.300216	-4.24235
H64	2.681303	-1.2375	-4.67444
H65	1.642499	-2.46835	-2.81413
H66	4.414964	-0.66412	-0.06431
H67	5.436121	0.584429	-1.92117
C68	-1.01604	2.82103	-1.72386
C69	-0.02291	2.00203	-0.87771
C70	1.318599	1.65783	-1.6137
H71	1.136887	1.583456	-2.68374
C72	2.512067	2.537494	-1.33726
C73	3.324749	2.357653	-0.21103
C74	2.790397	3.592198	-2.21555
C75	4.384307	3.229528	0.041538
H76	3.137375	1.524494	0.459169
C77	3.853701	4.461202	-1.96623
H78	2.172065	3.732702	-3.09915
C79	4.650993	4.284722	-0.83369
H80	5.006951	3.078553	0.918941
H81	4.060031	5.273383	-2.65765
H82	5.479195	4.960122	-0.63971
C83	0.480585	4.164482	0.573524
C84	0.758787	2.039441	1.422353
C85	0.794052	4.199421	2.083073
H86	-0.39661	4.76341	0.322686
H87	1.325616	4.49353	-0.03774

H41	3.478973	-1.76225	5.191988
C42	-3.71906	-0.54524	0.21562
C43	-4.77097	-0.28861	1.106341
C44	-3.96823	-0.5138	-1.16766
C45	-6.04644	0.003278	0.621363
H46	-4.59633	-0.31121	2.177546
C47	-5.24536	-0.23031	-1.64818
C48	-6.28587	0.032158	-0.75356
H49	-6.85447	0.204884	1.318821
H50	-5.42729	-0.2059	-2.71886
H51	-7.28015	0.258969	-1.12724
H52	-0.86383	-1.01244	-1.89314
H53	1.478939	1.962218	-1.61077
H54	-1.47995	-2.4542	3.352447
H55	-3.15469	-0.70044	-1.86338
H56	2.568873	-4.11303	-1.37944
C57	4.424899	0.287389	-2.67331
C58	3.884776	-0.89819	-3.17246
C59	3.200133	-1.76794	-2.32446
C60	3.053602	-1.47419	-0.96366
C61	3.591133	-0.27628	-0.4736
C62	4.272543	0.595594	-1.32093
H63	4.958236	0.96553	-3.33336
H64	3.989563	-1.1454	-4.22489
H65	2.753445	-2.66026	-2.75184
H66	3.450251	-0.01176	0.567471
H67	4.68554	1.518601	-0.92273
C68	-1.80027	3.235484	-1.45325
C69	-0.50647	2.514691	-1.00054
C70	0.738614	2.70347	-1.91536
H71	0.461773	2.451004	-2.94153
C72	1.350897	4.082673	-1.82073
C73	2.456756	4.284062	-0.981
C74	0.829999	5.184268	-2.51402
C75	3.009661	5.55478	-0.81321
H76	2.889179	3.431682	-0.46099
C77	1.379298	6.457215	-2.34733
H78	-0.00819	5.046799	-3.19259
C79	2.464709	6.649083	-1.48897
H80	3.869309	5.689592	-0.16219
H81	0.961828	7.299659	-2.89164
H82	2.891054	7.639549	-1.36026
C83	-0.26741	4.174904	0.982672
C84	0.605711	2.021948	1.168595
C85	0.79025	4.090237	2.099288

H88	1.633957	4.845304	2.340257
H89	-0.07985	4.466603	2.685019
N90	0.231728	2.725134	0.389567
O91	1.159077	2.832748	2.420673
O92	0.8975	0.8037	1.461012
H93	2.738228	-4.60739	0.197615
N94	-1.9751	-2.33218	0.679515
C95	-1.39066	-3.22158	-0.34925
H96	-1.66917	-2.9188	-1.37052
C97	-2.323	3.110926	-0.96829
H98	-2.15067	3.540773	0.022244
H99	-2.93944	3.816733	-1.53388
H100	-2.90118	2.195754	-0.83766
C101	-1.34254	2.196347	-3.08706
H102	-0.47022	2.11717	-3.74207
H103	-1.76226	1.196931	-2.96091
H104	-2.08101	2.81295	-3.60907
H105	-0.52391	3.788502	-1.92576

C3_ZR_Cheltop_TS2

C1	-3.98524	-3.88293	0.681175
C2	-1.82005	-4.74932	-0.02122
C3	-3.3232	-4.8089	-0.39123
C4	-2.7683	-3.42863	1.522743
H5	-4.6862	-4.43561	1.315278
H6	-3.51052	-4.4698	-1.41511
H7	-3.01474	-2.98963	2.489112
H8	-4.52662	-3.04274	0.246129
H9	-1.20364	-5.53391	-0.45951
H10	-3.68798	-5.83713	-0.31507
C11	-1.88052	-4.68561	1.521458
H12	-0.90958	-4.52668	1.999985
H13	-2.37033	-5.55995	1.95921
C14	0.175	-3.14618	-0.42073
N15	0.765578	-2.00379	-0.43106
C16	2.246123	-2.26439	-0.28724
C17	2.302232	-3.77706	-0.65444
P18	-1.99793	-0.82781	0.61814
Ir19	-0.2979	-0.05879	-0.57307
C20	-1.95251	-0.23698	2.348416
C21	-1.69324	0.804446	4.942641
C22	-1.4005	-1.01699	3.374571
C23	-2.37294	1.072138	2.635272
C24	-2.24414	1.586359	3.924692
C25	-1.27835	-0.4987	4.66369

H86	-1.27227	4.357446	1.375741
H87	-0.02351	4.947676	0.253114
H88	1.719449	4.59329	1.819108
H89	0.438881	4.461446	3.062747
N90	-0.17477	2.827715	0.395331
O91	1.070829	2.674793	2.242359
O92	0.872883	0.8321	0.983386
H93	2.930423	-4.41182	0.345737
N94	-2.0068	-2.72545	0.77891
C95	-1.36021	-3.4839	-0.32501
H96	-1.67734	-3.1263	-1.31475
C97	-2.97824	2.982361	-0.50003
H98	-2.77829	3.300267	0.525364
H99	-3.86118	3.529027	-0.84466
H100	-3.23716	1.921439	-0.47961
C101	-2.19623	2.812016	-2.87673
H102	-1.45467	3.09252	-3.62815
H103	-2.34028	1.726081	-2.92971
H104	-3.14167	3.286554	-3.15381
H105	-1.57166	4.309596	-1.46331
H106	0.992971	-0.06361	-1.69198
H107	-0.83815	1.420418	-1.05735

H26	-2.80139	1.687366	1.853224
H27	-2.58187	2.597354	4.136319
H28	-0.85857	-1.11707	5.451935
H29	-1.59414	1.205529	5.94688
O30	0.941475	-4.2396	-0.42589
C31	2.671494	-2.11982	1.183345
C32	1.763252	-1.84751	2.202437
C33	4.019419	-2.3261	1.513892
C34	2.188264	-1.76501	3.52969
H35	0.729173	-1.66417	1.957088
C36	4.444329	-2.25442	2.837342
H37	4.743527	-2.52666	0.728473
C38	3.526832	-1.97033	3.853138
H39	1.466881	-1.52427	4.305264
H40	5.492016	-2.41361	3.076061
H41	3.858807	-1.90612	4.885489
C42	-3.70002	-0.53486	0.010257
C43	-4.77059	-0.32988	0.893557
C44	-3.96232	-0.71209	-1.35778
C45	-6.07831	-0.28245	0.409657
H46	-4.5925	-0.21141	1.956965
C47	-5.26985	-0.66989	-1.83469
C48	-6.33075	-0.45134	-0.952
H49	-6.90031	-0.11705	1.100133
H50	-5.46091	-0.80292	-2.89552
H51	-7.3501	-0.41443	-1.32497
H52	-1.10979	-0.50009	-1.8372
H53	1.415467	1.300055	-1.44288
H54	-1.05717	-2.02433	3.16864
H55	-3.14081	-0.87414	-2.04749
H56	2.535195	-3.98084	-1.70101
C57	4.543432	0.405966	-2.84612
C58	4.103117	-0.82021	-3.34333
C59	3.368501	-1.68763	-2.53262
C60	3.065855	-1.35363	-1.20625
C61	3.505494	-0.11196	-0.71989
C62	4.239351	0.755673	-1.52985
H63	5.1188	1.079638	-3.47411
H64	4.327171	-1.10655	-4.36673
H65	3.022674	-2.61895	-2.96748
H66	3.271265	0.16915	0.301459
H67	4.57267	1.708972	-1.13297
C68	-1.78142	2.761061	-1.44996
C69	-0.60596	2.015559	-0.77901
C70	0.724671	2.149384	-1.62794

H71	0.473431	2.0746	-2.68425
C72	1.521492	3.412556	-1.39065
C73	2.506844	3.479488	-0.39616
C74	1.252689	4.560191	-2.14852
C75	3.191692	4.67018	-0.14842
H76	2.737284	2.595234	0.189754
C77	1.931075	5.754048	-1.8984
H78	0.507909	4.518517	-2.93948
C79	2.898816	5.814408	-0.8934
H80	3.954184	4.703267	0.625474
H81	1.707035	6.636	-2.49188
H82	3.428477	6.742956	-0.70081
C83	-0.48701	3.973941	1.050756
C84	0.474633	1.9	1.384183
C85	0.457112	3.979658	2.278704
H86	-1.51942	4.19798	1.330276
H87	-0.16425	4.682079	0.285696
H88	1.347587	4.589296	2.114738
H89	-0.03979	4.278499	3.203258
N90	-0.35932	2.584515	0.582028
O91	0.89237	2.60116	2.43921
O92	0.856548	0.731041	1.173597
H93	2.967122	-4.34739	-0.00836
N94	-1.88249	-2.51579	0.737858
C95	-1.31906	-3.32493	-0.37369
H96	-1.70418	-3.00863	-1.35402
C97	-3.07624	2.807662	-0.62511
H98	-2.94988	3.266606	0.357284
H99	-3.8269	3.402774	-1.15538
H100	-3.48933	1.810269	-0.48733
C101	-2.12221	2.211609	-2.84551
H102	-1.26434	2.178554	-3.52152
H103	-2.52067	1.198036	-2.76657
H104	-2.88443	2.840375	-3.31654
H105	-1.4441	3.80245	-1.58497
H106	1.195932	-0.30113	-2.95842
H107	0.503769	-0.27478	-3.22418

C3_ZS_Chelbot

C3_ZS_Chelbot_SM

C1	-3.79966	-3.44435	-1.29761
C2	-1.52645	-4.33553	-1.22873
C3	-2.73555	-4.17059	-2.18718
C4	-3.03668	-3.26023	0.032425
H5	-4.68885	-4.06414	-1.14223

C3_ZS_Chelbot_INT2

C1	-3.75023	-3.44428	-1.46494
C2	-1.54938	-4.41945	-1.07503
C3	-2.63794	-4.28781	-2.17153
C4	-3.12083	-3.16724	-0.08256
H5	-4.67708	-4.01518	-1.34578

H6	-2.47343	-3.60082	-3.08425	H6	-2.25319	-3.80744	-3.07646
H7	-3.65228	-2.98187	0.888381	H7	-3.81307	-2.78556	0.667985
H8	-4.12598	-2.48768	-1.70743	H8	-3.99517	-2.52087	-1.98952
H9	-0.79217	-5.08004	-1.5367	H9	-0.82705	-5.21896	-1.23754
H10	-3.08999	-5.15188	-2.51454	H10	-3.00602	-5.2772	-2.45676
C11	-2.22916	-4.56731	0.12707	C11	-2.4007	-4.49747	0.209719
H12	-1.54074	-4.60091	0.976913	H12	-1.80518	-4.48976	1.127558
H13	-2.85777	-5.46209	0.13285	H13	-3.07607	-5.35746	0.222241
C14	0.429156	-2.90221	-0.32813	C14	0.40533	-3.02	-0.12743
N15	1.006666	-1.82654	0.087315	N15	0.976495	-1.95737	0.323075
C16	2.382772	-2.19424	0.605014	C16	2.326165	-2.34275	0.877168
C17	2.252397	-3.74862	0.660058	C17	2.245159	-3.90668	0.801315
P18	-1.85716	-0.67337	0.334943	P18	-1.80935	-0.66996	0.317875
Ir19	0.318295	0.191319	-0.35401	Ir19	0.314328	0.092851	-0.31879
C20	-1.9784	-0.686	2.165398	C20	-1.90806	-0.69876	2.158825
C21	-1.94525	-0.63074	4.973078	C21	-2.01122	-0.70538	4.969021
C22	-1.93619	-1.87835	2.898717	C22	-2.17422	-1.88145	2.860367
C23	-1.98453	0.53702	2.856947	C23	-1.6704	0.478994	2.888631
C24	-1.97527	0.564489	4.249356	C24	-1.73211	0.477651	4.280151
C25	-1.92072	-1.84861	4.294778	C25	-2.22212	-1.8839	4.255745
H26	-1.98471	1.472943	2.310057	H26	-1.41112	1.397334	2.376082
H27	-1.98145	1.518352	4.769131	H27	-1.54895	1.399083	4.825708
H28	-1.88041	-2.78044	4.851275	H28	-2.41967	-2.81246	4.783222
H29	-1.93194	-0.61044	6.059004	H29	-2.0512	-0.70879	6.054379
O30	1.074409	-4.0559	-0.14127	O30	1.038862	-4.18381	0.036874
C31	2.655773	-1.69109	2.017398	C31	2.528845	-1.92101	2.325928
C32	1.6288	-1.24947	2.855021	C32	1.548041	-1.26219	3.066561
C33	3.954181	-1.80284	2.536884	C33	3.73355	-2.27556	2.954
C34	1.901031	-0.8774	4.172624	C34	1.777551	-0.9341	4.405302
H35	0.614761	-1.19208	2.485765	H35	0.605436	-1.0052	2.6071
C36	4.224577	-1.43668	3.853796	C36	3.96001	-1.95231	4.289045
H37	4.761611	-2.1623	1.905247	H37	4.505735	-2.79076	2.38764
C38	3.199328	-0.96198	4.673913	C38	2.98186	-1.27209	5.018489
H39	1.088104	-0.52599	4.801205	H39	1.002038	-0.41729	4.962063
H40	5.238032	-1.51866	4.236188	H40	4.900877	-2.22628	4.757479
H41	3.412523	-0.66801	5.69766	H41	3.159503	-1.0134	6.058333
C42	-3.44751	0.039784	-0.2504	C42	-3.40543	0.058362	-0.21252
C43	-4.46565	0.475696	0.606205	C43	-4.33169	0.597357	0.688167
C44	-3.64125	0.120185	-1.64015	C44	-3.70044	0.057905	-1.58535
C45	-5.64458	1.011407	0.08193	C45	-5.52688	1.146238	0.219557
H46	-4.34485	0.405498	1.68225	H46	-4.12565	0.597948	1.753294
C47	-4.81904	0.65364	-2.15844	C47	-4.89102	0.611	-2.0492
C48	-5.82201	1.111316	-1.29771	C48	-5.80573	1.161888	-1.14651
H49	-6.42561	1.347793	0.757728	H49	-6.23999	1.56058	0.926271
H50	-4.95977	0.702468	-3.23495	H50	-5.1137	0.59828	-3.11266

H51	-6.7394	1.529389	-1.70103
H52	0.681668	0.604731	1.095665
H53	1.888502	0.314765	-0.58516
H54	-1.88573	-2.83159	2.386372
H55	-2.88234	-0.26333	-2.31376
H56	3.105699	-4.27993	0.239506
C57	5.176147	-0.84002	-2.43866
C58	4.417824	-1.99494	-2.6342
C59	3.535797	-2.42883	-1.64562
C60	3.40223	-1.72374	-0.441
C61	4.150038	-0.55054	-0.26708
C62	5.033246	-0.11605	-1.25624
H63	5.86251	-0.49929	-3.2081
H64	4.512195	-2.56065	-3.55678
H65	2.944355	-3.3225	-1.8274
H66	4.047576	0.023866	0.6469
H67	5.603498	0.795479	-1.10267
C68	-0.80095	3.322061	-0.21356
C69	-0.16946	2.180599	-1.02677
C70	1.275227	2.022899	-1.04517
H71	1.793935	2.471395	-0.20294
C72	2.061803	2.11181	-2.32132
C73	2.505902	3.397129	-2.67696
C74	2.34916	1.040213	-3.16987
C75	3.197021	3.607598	-3.8687
H76	2.306077	4.235423	-2.01247
C77	3.037744	1.252991	-4.36567
H78	2.044007	0.038347	-2.89284
C79	3.457856	2.53292	-4.72286
H80	3.533437	4.607526	-4.1285
H81	3.252494	0.408028	-5.0134
H82	3.993504	2.694653	-5.65396
C83	-1.05436	3.145787	-3.27797
C84	-0.75856	0.872557	-3.01119
C85	-1.67341	2.363948	-4.45001
H86	-0.11481	3.643055	-3.54466
H87	-1.7477	3.878216	-2.86046
H88	-2.76653	2.347837	-4.40039
H89	-1.34867	2.705584	-5.43283
N90	-0.80047	2.043621	-2.34093
O91	-1.20215	0.99882	-4.26874
O92	-0.38622	-0.22136	-2.55253
H93	2.055625	-4.09861	1.674906
N94	-1.93371	-2.27836	-0.17242
C95	-0.9095	-2.93138	-1.02311

H51	-6.73607	1.590768	-1.50727
H52	0.478218	0.637759	1.132868
H53	1.740688	1.53362	-2.432
H54	-2.32342	-2.8123	2.33072
H55	-3.015	-0.39849	-2.29029
H56	3.088112	-4.36283	0.280835
C57	5.103771	-0.63532	-1.99461
C58	4.229922	-1.66129	-2.36313
C59	3.377613	-2.2242	-1.41579
C60	3.381477	-1.77737	-0.086
C61	4.261064	-0.75014	0.27381
C62	5.117552	-0.18517	-0.67488
H63	5.767466	-0.19218	-2.73129
H64	4.208969	-2.01977	-3.38817
H65	2.688606	-3.00576	-1.72633
H66	4.271966	-0.37579	1.29113
H67	5.793021	0.612209	-0.37823
C68	-0.60698	3.202018	-0.44636
C69	-0.03252	2.012649	-1.25399
C70	1.342016	2.422099	-1.93156
H71	2.053138	2.679734	-1.14228
C72	1.296647	3.527084	-2.96424
C73	1.321223	4.884089	-2.61118
C74	1.226392	3.198004	-4.32664
C75	1.240151	5.879053	-3.58657
H76	1.412897	5.166858	-1.56605
C77	1.144042	4.188878	-5.30541
H78	1.243198	2.150261	-4.62124
C79	1.141979	5.535113	-4.93594
H80	1.259703	6.924781	-3.29243
H81	1.093804	3.911677	-6.35502
H82	1.079644	6.310373	-5.69403
C83	-1.79824	2.566101	-3.20811
C84	-0.81093	0.489663	-2.97785
C85	-1.92035	1.74246	-4.50668
H86	-1.328	3.533783	-3.38013
H87	-2.76776	2.711045	-2.72285
H88	-2.94451	1.646894	-4.86941
H89	-1.27524	2.125835	-5.30091
N90	-0.94748	1.685285	-2.39385
O91	-1.44774	0.406953	-4.15569
O92	-0.1978	-0.48891	-2.49687
H93	2.121578	-4.35855	1.786948
N94	-1.95159	-2.25	-0.25528
C95	-0.87997	-3.03157	-0.91791

H96	-0.78175	-2.40568	-1.97583
C97	-2.32249	3.207897	-0.06731
H98	-2.73955	4.16126	0.272268
H99	-2.81861	2.928824	-0.99974
H100	-2.59257	2.449973	0.667636
C101	-0.14497	3.542552	1.157206
H102	-0.1797	2.63138	1.760199
H103	0.901073	3.851691	1.078523
H104	-0.67448	4.33462	1.69576
H105	-0.59912	4.231462	-0.80532

C3_ZS_Chelbot_TS1

C1	-3.77593	-3.44484	-1.26031
C2	-1.5019	-4.33488	-1.20938
C3	-2.71883	-4.17131	-2.15817
C4	-3.00516	-3.26653	0.066385
H5	-4.66613	-4.06245	-1.10207
H6	-2.46469	-3.60233	-3.05806
H7	-3.61664	-2.99287	0.926887
H8	-4.10198	-2.48611	-1.66566
H9	-0.76757	-5.07637	-1.52462
H10	-3.07566	-5.15308	-2.48159
C11	-2.19464	-4.57252	0.150817
H12	-1.50074	-4.60588	0.996243
H13	-2.82113	-5.46882	0.157723
C14	0.460238	-2.89096	-0.33058
N15	1.032018	-1.81046	0.078378
C16	2.412682	-2.16388	0.584943
C17	2.304591	-3.72148	0.630678
P18	-1.82859	-0.67719	0.359109
Ir19	0.295375	0.204808	-0.33683
C20	-1.95857	-0.67384	2.187404
C21	-1.9475	-0.58476	4.992918
C22	-1.91885	-1.85762	2.933964
C23	-1.97444	0.557584	2.863304
C24	-1.97574	0.601872	4.254811
C25	-1.91439	-1.8108	4.329935
H26	-1.97381	1.486046	2.303818
H27	-1.98916	1.561986	4.762684
H28	-1.87625	-2.73582	4.897817
H29	-1.94314	-0.55119	6.078527
O30	1.118829	-4.03966	-0.15521
C31	2.685456	-1.66526	1.999358
C32	1.654782	-1.24309	2.842306
C33	3.987049	-1.7573	2.514198

H96	-0.63211	-2.59868	-1.89385
C97	-2.04015	3.026089	0.065809
H98	-2.4106	3.976503	0.464536
H99	-2.74107	2.693379	-0.69944
H100	-2.08602	2.298072	0.873769
C101	0.297069	3.608859	0.729321
H102	0.372138	2.797583	1.459782
H103	1.3133	3.867137	0.420768
H104	-0.12079	4.484773	1.2363
H105	-0.63061	4.057682	-1.13745
H106	1.951417	0.617822	-0.12919
H107	1.959393	0.244907	-0.88899

C3_ZS_Chelbot_TS3

C1	-3.77689	-3.40923	-1.57868
C2	-1.54655	-4.33801	-1.24718
C3	-2.64318	-4.17961	-2.3324
C4	-3.15265	-3.19663	-0.18206
H5	-4.68829	-4.01069	-1.49532
H6	-2.27607	-3.64159	-3.212
H7	-3.857	-2.87853	0.586339
H8	-4.04644	-2.46216	-2.04688
H9	-0.80095	-5.10392	-1.45772
H10	-2.9829	-5.16312	-2.66896
C11	-2.39106	-4.51746	0.033093
H12	-1.79479	-4.53897	0.950432
H13	-3.03571	-5.4002	-0.00406
C14	0.371836	-2.93321	-0.23554
N15	0.964548	-1.88976	0.244537
C16	2.316257	-2.30677	0.78952
C17	2.181037	-3.86222	0.717324
P18	-1.88087	-0.68643	0.362552
Ir19	0.395203	0.137373	-0.14334
C20	-2.03336	-0.81458	2.195006
C21	-2.17754	-0.90494	5.002916
C22	-2.35749	-2.00737	2.854848
C23	-1.75809	0.327954	2.967044
C24	-1.84026	0.286663	4.357014
C25	-2.42462	-2.05148	4.249183
H26	-1.4513	1.249175	2.483649
H27	-1.62682	1.180954	4.935196
H28	-2.66867	-2.98665	4.744448
H29	-2.23406	-0.94122	6.086888
O30	0.997021	-4.10239	-0.09336
C31	2.524558	-1.92089	2.248036

C34	1.924951	-0.86891	4.159549
H35	0.63883	-1.20224	2.477049
C36	4.255941	-1.38918	3.831148
H37	4.798068	-2.10235	1.879161
C38	3.226255	-0.93255	4.6558
H39	1.108374	-0.53182	4.791164
H40	5.271858	-1.45527	4.209905
H41	3.438614	-0.63632	5.679054
C42	-3.41032	0.038384	-0.23961
C43	-4.43564	0.473967	0.608031
C44	-3.59048	0.113428	-1.6311
C45	-5.61177	1.002787	0.071908
H46	-4.32327	0.407783	1.685137
C47	-4.76522	0.639915	-2.16119
C48	-5.77627	1.094626	-1.3093
H49	-6.40047	1.339805	0.738397
H50	-4.89781	0.685605	-3.23884
H51	-6.6923	1.50704	-1.72212
H52	0.642254	0.609789	1.120284
H53	1.868241	0.456635	-0.59671
H54	-1.8615	-2.81661	2.432401
H55	-2.82335	-0.26894	-2.29558
H56	3.158594	-4.2386	0.193976
C57	5.180103	-0.7539	-2.4572
C58	4.434668	-1.91629	-2.65882
C59	3.5577	-2.36532	-1.67225
C60	3.417479	-1.66875	-0.46345
C61	4.148255	-0.48595	-0.28559
C62	5.026589	-0.03638	-1.27236
H63	5.863049	-0.40174	-3.22454
H64	4.535412	-2.47617	-3.58431
H65	2.976911	-3.26522	-1.85772
H66	4.040197	0.082083	0.631589
H67	5.584847	0.881854	-1.11539
C68	-0.7413	3.3491	-0.2103
C69	-0.12148	2.190382	-1.01012
C70	1.336222	2.019712	-1.01909
H71	1.846072	2.47554	-0.17493
C72	2.120305	2.126307	-2.29581
C73	2.57817	3.410662	-2.63444
C74	2.372827	1.065707	-3.16872
C75	3.248028	3.632044	-3.83629
H76	2.403324	4.239851	-1.95191
C77	3.039658	1.289851	-4.37465
H78	2.058361	0.063705	-2.90148

C32	1.466731	-1.5155	3.065612
C33	3.788612	-2.12129	2.822963
C34	1.678897	-1.26471	4.422554
H35	0.474389	-1.39793	2.652879
C36	3.997223	-1.87866	4.178948
H37	4.616444	-2.45785	2.20537
C38	2.943621	-1.43916	4.981867
H39	0.844543	-0.93895	5.035806
H40	4.984328	-2.03053	4.605884
H41	3.108472	-1.24264	6.037221
C42	-3.46927	0.0808	-0.15047
C43	-4.3863	0.631768	0.752266
C44	-3.74779	0.134363	-1.52675
C45	-5.55694	1.234423	0.28612
H46	-4.19301	0.59574	1.819355
C47	-4.91729	0.733314	-1.98831
C48	-5.82415	1.290076	-1.08105
H49	-6.26242	1.655924	0.996329
H50	-5.13007	0.755736	-3.05387
H51	-6.73715	1.756229	-1.43946
H52	0.580581	0.432393	1.372975
H53	1.849203	1.619074	-2.21304
H54	-2.53914	-2.91435	2.293976
H55	-3.05992	-0.31141	-2.23841
H56	3.028658	-4.35404	0.240233
C57	5.115569	-0.7084	-2.12005
C58	4.299215	-1.79601	-2.43515
C59	3.436059	-2.32024	-1.47598
C60	3.370422	-1.77035	-0.18772
C61	4.183094	-0.67166	0.113809
C62	5.054868	-0.15031	-0.84355
H63	5.792312	-0.29973	-2.86501
H64	4.333439	-2.236	-3.42774
H65	2.793969	-3.15347	-1.7506
H66	4.127431	-0.20959	1.092653
H67	5.682385	0.699322	-0.58963
C68	-0.63834	3.387669	-0.48266
C69	0.019565	2.201782	-1.22763
C70	1.40046	2.560668	-1.88662
H71	2.067763	2.954448	-1.11581
C72	1.318417	3.502348	-3.06558
C73	1.227805	4.891823	-2.90059
C74	1.306817	2.982579	-4.36829
C75	1.0963	5.734987	-4.0046
H76	1.267027	5.319982	-1.90261

C79	3.472481	2.570098	-4.71626
H80	3.595347	4.631051	-4.08477
H81	3.227448	0.454255	-5.04269
H82	3.990281	2.741291	-5.65573
C83	-0.96489	3.181887	-3.27539
C84	-0.71761	0.903716	-3.00525
C85	-1.59256	2.411345	-4.45059
H86	-0.01581	3.662545	-3.538
H87	-1.64728	3.926838	-2.862
H88	-2.68614	2.414964	-4.40658
H89	-1.25675	2.746474	-5.43196
N90	-0.73599	2.075341	-2.33643
O91	-1.14743	1.037674	-4.26664
O92	-0.37166	-0.19787	-2.54223
H93	2.126582	-4.08144	1.645976
N94	-1.90335	-2.28457	-0.13769
C95	-0.8881	-2.92884	-1.00624
H96	-0.77787	-2.39927	-1.95928
C97	-2.26693	3.247506	-0.08707
H98	-2.68165	4.197051	0.266401
H99	-2.7539	2.991144	-1.03161
H100	-2.55132	2.475331	0.627562
C101	-0.10304	3.564337	1.169217
H102	-0.16186	2.657314	1.776455
H103	0.94902	3.856939	1.105457
H104	-0.62716	4.367748	1.69631
H105	-0.52106	4.255593	-0.79979
X	0	0	0

C3_ZS_Chelbot_INT1

C1	-3.86714	-3.49565	-1.04459
C2	-1.61234	-4.43598	-1.02658
C3	-2.85772	-4.28916	-1.94076
C4	-3.05305	-3.28624	0.250898
H5	-4.77059	-4.0783	-0.83687
H6	-2.62283	-3.77081	-2.87571
H7	-3.63099	-2.96776	1.119241
H8	-4.17533	-2.54332	-1.4796
H9	-0.90169	-5.20335	-1.334
H10	-3.24739	-5.27674	-2.20297
C11	-2.26477	-4.6039	0.363186
H12	-1.54562	-4.61598	1.188101
H13	-2.90762	-5.48628	0.427405
C14	0.415709	-2.98534	-0.2993
N15	0.980825	-1.90046	0.100692
C77	1.176178	3.822383	-5.47513
H78	1.409564	1.909314	-4.51478
C79	1.060894	5.202096	-5.29467
H80	1.026451	6.809065	-3.85796
H81	1.175055	3.401876	-6.4771
H82	0.958866	5.858981	-6.15344
C83	-1.76305	2.530114	-3.14681
C84	-0.63927	0.515022	-2.86962
C85	-1.86743	1.647426	-4.40919
H86	-1.32274	3.501764	-3.37263
H87	-2.73137	2.666554	-2.65871
H88	-2.89482	1.465449	-4.72718
H89	-1.28052	2.045974	-5.23946
N90	-0.8741	1.713204	-2.3013
O91	-1.28752	0.365022	-4.03144
O92	0.055702	-0.38931	-2.37921
H93	1.997117	-4.2991	1.699956
N94	-2.01528	-2.23595	-0.29798
C95	-0.92349	-2.93723	-1.01507
H96	-0.69478	-2.44519	-1.96666
C97	-2.01468	3.06898	0.108839
H98	-2.46609	3.981009	0.512012
H99	-2.71183	2.640358	-0.6099
H100	-1.91963	2.354753	0.927808
C101	0.260634	3.965516	0.622817
H102	0.455637	3.214637	1.398234
H103	1.223812	4.326736	0.254673
H104	-0.24304	4.810252	1.101654
H105	-0.76909	4.173234	-1.24021
H106	0.780395	1.650332	0.028914
H107	1.946964	0.42717	-0.30181

C3_ZS_Chelbot_PROD

C1	-3.86701	-3.58765	-1.17036
C2	-1.5816	-4.4505	-1.13453
C3	-2.79723	-4.27619	-2.08318
C4	-3.10609	-3.44363	0.166522
H5	-4.75298	-4.21764	-1.03894
H6	-2.54725	-3.68191	-2.96789
H7	-3.72708	-3.20586	1.030753
H8	-4.19831	-2.61889	-1.54778
H9	-0.83626	-5.17194	-1.46941
H10	-3.13998	-5.25406	-2.43292
C11	-2.2784	-4.7406	0.214365
H12	-1.58987	-4.78885	1.06337

C16	2.377932	-2.21969	0.548556	H13	-2.89048	-5.64665	0.188429
C17	2.35054	-3.78656	0.494668	C14	0.363243	-3.0087	-0.21871
P18	-1.83208	-0.71421	0.405865	N15	0.966965	-1.93913	0.192842
Ir19	0.139123	0.090396	-0.3147	C16	2.360533	-2.31106	0.673789
C20	-1.97328	-0.63626	2.22913	C17	2.212608	-3.86311	0.720786
C21	-1.97906	-0.44791	5.027153	P18	-1.92986	-0.83339	0.502179
C22	-1.88074	-1.79024	3.016284	Ir19	0.278946	-0.04765	-0.10712
C23	-2.0522	0.617186	2.858553	C20	-2.13695	-0.8426	2.322886
C24	-2.05986	0.709558	4.24747	C21	-2.20493	-0.76308	5.130964
C25	-1.88657	-1.69446	4.409505	C22	-2.12806	-2.02872	3.068356
H26	-2.09381	1.523687	2.264679	C23	-2.15204	0.385691	3.004961
H27	-2.12058	1.684662	4.722174	C24	-2.19434	0.425532	4.396168
H28	-1.8081	-2.59598	5.010153	C25	-2.16403	-1.98706	4.464212
H29	-1.98127	-0.37518	6.110938	H26	-2.11449	1.317931	2.449578
O30	1.112609	-4.12084	-0.1991	H27	-2.20877	1.383538	4.908106
C31	2.66203	-1.79038	1.984137	H28	-2.15492	-2.91473	5.029326
C32	1.653657	-1.34966	2.842342	H29	-2.2345	-0.73282	6.216288
C33	3.961568	-1.95047	2.48882	O30	1.013468	-4.1601	-0.05447
C34	1.942751	-1.0351	4.171837	C31	2.640181	-1.83203	2.093611
H35	0.641896	-1.24608	2.47918	C32	1.585716	-1.58563	2.981542
C36	4.249259	-1.64165	3.816396	C33	3.953766	-1.80069	2.578841
H37	4.756401	-2.30056	1.835396	C34	1.840589	-1.255	4.31119
C38	3.240354	-1.17377	4.661463	H35	0.5601	-1.64196	2.639156
H39	1.142969	-0.6849	4.818129	C36	4.207909	-1.47559	3.911659
H40	5.262631	-1.76211	4.188751	H37	4.783882	-2.02526	1.916519
H41	3.467139	-0.92536	5.694334	C38	3.153717	-1.19014	4.779153
C42	-3.3743	0.030086	-0.24789	H39	1.005595	-1.05061	4.975417
C43	-4.41622	0.494077	0.564297	H40	5.233289	-1.4452	4.268874
C44	-3.51483	0.080107	-1.64491	H41	3.354825	-0.92865	5.814113
C45	-5.56853	1.030857	-0.0136	C42	-3.45071	-0.04473	-0.1665
H46	-4.3382	0.440625	1.64477	C43	-4.46546	0.526575	0.60919
C47	-4.66598	0.61467	-2.21616	C44	-3.5543	-0.00019	-1.56804
C48	-5.69203	1.101374	-1.40024	C45	-5.55571	1.147785	-0.00622
H49	-6.37013	1.391474	0.624526	H46	-4.41415	0.48849	1.692413
H50	-4.76878	0.643885	-3.29755	C47	-4.64395	0.614729	-2.17917
H51	-6.5892	1.521431	-1.84562	C48	-5.6462	1.198214	-1.39677
H52	0.435022	0.538428	1.146957	H49	-6.33868	1.586177	0.605956
H53	1.885237	0.859331	-0.83792	H50	-4.71823	0.62748	-3.26343
H54	-1.77295	-2.7615	2.548031	H51	-6.49757	1.678605	-1.87015
H55	-2.7336	-0.32413	-2.27922	H52	0.61502	0.261026	1.3761
H56	3.182676	-4.22662	-0.05501	H53	1.825206	1.824707	-1.88888
C57	5.035773	-0.50388	-2.43298	H54	-2.06599	-2.98757	2.566751
C58	4.301522	-1.65702	-2.71372	H55	-2.79178	-0.47098	-2.18052
C59	3.455256	-2.1993	-1.7482	H56	3.051804	-4.39836	0.278028
C60	3.338706	-1.60964	-0.48118	C57	4.944933	-0.91295	-2.52291

C61	4.057912	-0.43642	-0.22029
C62	4.902289	0.109842	-1.18875
H63	5.692529	-0.07716	-3.18504
H64	4.385148	-2.13472	-3.68582
H65	2.878189	-3.08742	-1.9935
H66	3.968688	0.047935	0.745925
H67	5.453635	1.019337	-0.96855
C68	-0.60136	3.274113	-0.35147
C69	-0.03452	2.021722	-1.04687
C70	1.529001	1.932435	-1.05798
H71	1.933054	2.481155	-0.20942
C72	2.232673	2.306457	-2.3369
C73	2.672248	3.625836	-2.50938
C74	2.405643	1.388517	-3.37741
C75	3.255218	4.025057	-3.71285
H76	2.554476	4.342686	-1.69975
C77	2.982809	1.788823	-4.58285
H78	2.105192	0.355274	-3.23533
C79	3.404328	3.108323	-4.75628
H80	3.593483	5.050254	-3.83501
H81	3.110853	1.06578	-5.38348
H82	3.855612	3.419447	-5.6941
C83	-0.76816	3.018391	-3.38751
C84	-0.58674	0.744452	-3.04942
C85	-1.3351	2.229259	-4.58362
H86	0.188196	3.499327	-3.6132
H87	-1.47133	3.76869	-3.02159
H88	-2.42782	2.268696	-4.62675
H89	-0.91383	2.524147	-5.5448
N90	-0.58457	1.92995	-2.41557
O91	-0.9543	0.845944	-4.33161
O92	-0.295	-0.35483	-2.53377
H93	2.292542	-4.22237	1.493268
N94	-1.93674	-2.33879	-0.02316
C95	-0.96692	-3.03385	-0.90477
H96	-0.89162	-2.54792	-1.88494
C97	-2.13619	3.249847	-0.28306
H98	-2.51976	4.229475	0.020031
H99	-2.6053	2.976742	-1.23168
H100	-2.47531	2.51655	0.450319
C101	-0.03006	3.523597	1.052677
H102	-0.17111	2.649043	1.693684
H103	1.036951	3.764362	1.045764
H104	-0.54287	4.3741	1.513299
H105	-0.30504	4.138053	-0.97136

C58	4.215272	-2.09404	-2.66263
C59	3.420192	-2.55165	-1.61329
C60	3.340271	-1.84195	-0.40713
C61	4.062559	-0.64861	-0.28532
C62	4.864911	-0.19274	-1.33118
H63	5.569651	-0.557	-3.33728
H64	4.263794	-2.66103	-3.58787
H65	2.843711	-3.46175	-1.75697
H66	3.988995	-0.06486	0.62388
H67	5.423434	0.731641	-1.2137
C68	-0.81401	3.701828	-0.505
C69	-0.08695	2.539966	-1.22669
C70	1.389494	2.792707	-1.63657
H71	1.940335	3.157269	-0.76743
C72	1.518563	3.722145	-2.82183
C73	1.457674	5.115937	-2.68981
C74	1.662478	3.17602	-4.10601
C75	1.517981	5.941272	-3.81406
H76	1.368116	5.561673	-1.70277
C77	1.723561	3.997791	-5.23203
H78	1.736649	2.096384	-4.22031
C79	1.644021	5.384453	-5.08842
H80	1.470842	7.020032	-3.69505
H81	1.842849	3.557459	-6.21834
H82	1.692033	6.027634	-5.96232
C83	-1.61103	2.930912	-3.28985
C84	-0.72394	0.811556	-2.9017
C85	-1.65682	2.062898	-4.55989
H86	-1.08735	3.871379	-3.4657
H87	-2.60821	3.134676	-2.8878
H88	-2.64513	2.010224	-5.01773
H89	-0.91528	2.379946	-5.29769
N90	-0.85687	2.057529	-2.37682
O91	-1.30065	0.728737	-4.11167
O92	-0.20406	-0.18001	-2.37881
H93	2.034033	-4.21746	1.737162
N94	-2.01572	-2.44415	0.006624
C95	-0.99204	-3.03968	-0.88623
H96	-0.88857	-2.47969	-1.8243
C97	-2.21275	3.276718	-0.03532
H98	-2.75881	4.137632	0.3601
H99	-2.81929	2.817391	-0.81702
H100	-2.1243	2.539558	0.767939
C101	-0.00053	4.203792	0.698554
H102	0.234439	3.375776	1.378808

C3_ZS_Chelbot_TS2

C1	-3.76072	-3.46184	-1.38819
C2	-1.5185	-4.39179	-1.11872
C3	-2.65125	-4.24252	-2.16953
C4	-3.08706	-3.22574	-0.01968
H5	-4.66694	-4.06347	-1.26187
H6	-2.31234	-3.71298	-3.06551
H7	-3.75462	-2.89488	0.776458
H8	-4.04801	-2.52295	-1.86263
H9	-0.78202	-5.16381	-1.34114
H10	-3.00297	-5.22849	-2.48555
C11	-2.32138	-4.54678	0.189609
H12	-1.69383	-4.56253	1.085699
H13	-2.97442	-5.42398	0.189927
C14	0.429822	-2.95281	-0.18358
N15	0.964612	-1.87664	0.279781
C16	2.375102	-2.1909	0.6919
C17	2.372391	-3.75723	0.591887
P18	-1.84293	-0.69913	0.389213
Ir19	0.136313	0.151505	-0.16543
C20	-1.97499	-0.76774	2.220683
C21	-2.02304	-0.84252	5.026898
C22	-2.01928	-1.99166	2.898022
C23	-1.93737	0.42253	2.967949
C24	-1.96981	0.38485	4.359226
C25	-2.04291	-2.02736	4.293841
H26	-1.85774	1.37998	2.465257
H27	-1.94223	1.313178	4.922533
H28	-2.06809	-2.98524	4.805106
H29	-2.04018	-0.87191	6.112462
O30	1.144115	-4.07949	-0.12057
C31	2.667406	-1.77214	2.127318
C32	1.698162	-1.17666	2.939993
C33	3.917862	-2.07684	2.687451
C34	1.98836	-0.8442	4.264757
H35	0.712323	-0.9745	2.546968
C36	4.206059	-1.74915	4.011496
H37	4.677225	-2.56727	2.08387
C38	3.244496	-1.11889	4.804785
H39	1.216731	-0.379	4.871711
H40	5.183724	-1.98598	4.421331
H41	3.47107	-0.85819	5.834324
C42	-3.40844	0.030915	-0.20636
C43	-4.39476	0.537434	0.647701
H103	0.939601	4.679982	0.411199
H104	-0.58485	4.940577	1.257282
H105	-0.90428	4.521882	-1.22915
H106	-0.1176	1.763073	-0.38246
H107	1.792077	0.372584	-0.36784

C44	-3.61933	0.049476	-1.59529
C45	-5.56814	1.07663	0.117483
H46	-4.2545	0.515212	1.723094
C47	-4.7881	0.593928	-2.11983
C48	-5.76294	1.114958	-1.26269
H49	-6.33029	1.4652	0.786554
H50	-4.94767	0.598479	-3.19457
H51	-6.67652	1.537166	-1.67106
H52	0.188189	0.675502	1.296074
H53	1.883042	1.376416	-1.68324
H54	-2.00914	-2.92233	2.346688
H55	-2.88353	-0.38639	-2.26243
H56	3.208629	-4.15562	0.016549
C57	4.842256	-0.46862	-2.47046
C58	3.774796	-1.31875	-2.7651
C59	3.01536	-1.86861	-1.7355
C60	3.297827	-1.58153	-0.38924
C61	4.378388	-0.73796	-0.10706
C62	5.141977	-0.18608	-1.13908
H63	5.43215	-0.03149	-3.27082
H64	3.527486	-1.55193	-3.79695
H65	2.184129	-2.51512	-1.99702
H66	4.615931	-0.48111	0.918181
H67	5.969269	0.474137	-0.8952
C68	-0.76158	3.271041	-0.33962
C69	-0.14525	2.04535	-1.05148
C70	1.364898	2.323674	-1.47201
H71	1.888592	2.765671	-0.62555
C72	1.539736	3.195363	-2.69524
C73	1.501474	4.593953	-2.60338
C74	1.715453	2.611246	-3.9576
C75	1.602621	5.386473	-3.74769
H76	1.397225	5.066006	-1.62989
C77	1.820095	3.401316	-5.10366
H78	1.779374	1.528026	-4.03912
C79	1.753466	4.792303	-5.0025
H80	1.56912	6.468813	-3.65919
H81	1.961614	2.931824	-6.07359
H82	1.83318	5.409682	-5.89259
C83	-1.59266	2.695411	-3.21356
C84	-0.66544	0.595891	-2.92555
C85	-1.60795	1.888447	-4.53217
H86	-1.05034	3.635653	-3.32305
H87	-2.60008	2.89961	-2.83892
H88	-2.59893	1.804737	-4.98097

H89	-0.89645	2.280298	-5.26194
N90	-0.89047	1.767844	-2.31625
O91	-1.17067	0.546725	-4.16633
O92	-0.07694	-0.38136	-2.41539
H93	2.321379	-4.23419	1.572748
N94	-1.94764	-2.27404	-0.19804
C95	-0.8815	-2.9939	-0.92706
H96	-0.67773	-2.51887	-1.89464
C97	-2.23853	3.097608	0.031933
H98	-2.66606	4.057841	0.338182
H99	-2.84834	2.700195	-0.77951
H100	-2.34359	2.409499	0.87104
C101	0.015307	3.693025	0.91923
H102	0.073087	2.864703	1.632952
H103	1.035089	4.025827	0.708418
H104	-0.49866	4.526103	1.409487
H105	-0.70235	4.11117	-1.04903
H106	2.116397	1.057975	0.892576
H107	2.528315	0.737021	0.356272

C3_ZR_Chelbot

C3_ZR_Chelbot_SM

C1	-4.21652	-2.94392	1.121838
C2	-2.20859	-4.24784	0.653239
C3	-3.71566	-4.17308	0.294898
C4	-2.92291	-2.47574	1.826573
H5	-4.965	-3.23304	1.867033
H6	-3.88076	-4.0532	-0.78058
H7	-3.06798	-1.78553	2.656694
H8	-4.65393	-2.16417	0.49822
H9	-1.7287	-5.19659	0.416817
H10	-4.21713	-5.09487	0.603061
C11	-2.21911	-3.80897	2.133135
H12	-1.22462	-3.67605	2.569894
H13	-2.81392	-4.47316	2.766494
C14	-0.0145	-3.11205	-0.13523
N15	0.818947	-2.13676	-0.27555
C16	2.19706	-2.7214	-0.52892
C17	1.931055	-4.23502	-0.25357
P18	-1.86464	-0.34886	0.181523
Ir19	0.44357	-0.02243	-0.46204
C20	-2.27295	0.737486	1.586155
C21	-2.59362	2.490097	3.752764
C22	-1.65536	0.466598	2.819937
C23	-3.03022	1.908058	1.443134

C3_ZR_Chelbot_INT2

C1	-4.25596	-3.0884	-0.67751
C2	-2.13637	-4.28408	-0.8835
C3	-3.45882	-4.01753	-1.65133
C4	-3.25246	-2.90055	0.484326
H5	-5.16212	-3.57841	-0.30566
H6	-3.28095	-3.55056	-2.62581
H7	-3.66835	-2.47324	1.396806
H8	-4.55504	-2.14431	-1.13124
H9	-1.57071	-5.14953	-1.22844
H10	-3.98581	-4.95868	-1.83195
C11	-2.61962	-4.2996	0.583735
H12	-1.81021	-4.35776	1.317605
H13	-3.35145	-5.08779	0.781659
C14	0.121669	-3.09627	-0.46512
N15	0.863027	-2.10455	-0.12261
C16	2.247223	-2.61782	0.155324
C17	2.017945	-4.16898	0.068319
P18	-1.86832	-0.43784	0.074375
Ir19	0.34326	0.040969	-0.47435
C20	-2.1679	0.019014	1.814285
C21	-2.2944	0.781642	4.509212
C22	-1.88101	-0.90905	2.826608
C23	-2.50089	1.337005	2.160346

C24	-3.18929	2.776458	2.525611	C24	-2.5662	1.710614	3.50258
C25	-1.82268	1.333004	3.896662	C25	-1.9502	-0.5285	4.166677
H26	-3.47646	2.164658	0.489931	H26	-2.69178	2.07892	1.394317
H27	-3.77836	3.680672	2.40186	H27	-2.82312	2.734972	3.756681
H28	-1.34774	1.109049	4.847558	H28	-1.73344	-1.25679	4.943246
H29	-2.72138	3.167164	4.592386	H29	-2.34502	1.076862	5.553151
O30	0.485638	-4.35054	-0.17942	O30	0.669656	-4.31628	-0.45745
C31	3.234277	-2.24123	0.481078	C31	2.710993	-2.25974	1.563376
C32	2.854172	-1.676	1.703655	C32	1.863741	-1.6419	2.488062
C33	4.591622	-2.50687	0.253476	C33	3.988474	-2.6619	1.983926
C34	3.815251	-1.33001	2.653322	C34	2.298258	-1.39241	3.79185
H35	1.806321	-1.49302	1.907831	H35	0.863304	-1.35212	2.19556
C36	5.551677	-2.1659	1.205929	C36	4.420004	-2.41658	3.286376
H37	4.903563	-2.97576	-0.67493	H37	4.65333	-3.16365	1.285236
C38	5.167863	-1.56497	2.404798	C38	3.578297	-1.77067	4.195523
H39	3.503743	-0.87827	3.591077	H39	1.624244	-0.90329	4.489972
H40	6.599813	-2.3707	1.007933	H40	5.414825	-2.73006	3.589815
H41	5.915892	-1.29208	3.143433	H41	3.915965	-1.57506	5.208938
C42	-3.20959	-0.25349	-1.05862	C42	-3.30086	0.161913	-0.8979
C43	-4.54327	0.067682	-0.76053	C43	-4.47754	0.663323	-0.33006
C44	-2.87983	-0.67835	-2.3567	C44	-3.25706	-0.07164	-2.28197
C45	-5.52074	-0.0068	-1.75127	C45	-5.57299	0.969147	-1.13973
H46	-4.82557	0.367744	0.243336	H46	-4.55187	0.817346	0.739841
C47	-3.86568	-0.76988	-3.33934	C47	-4.34966	0.23566	-3.0881
C48	-5.18449	-0.4262	-3.04034	C48	-5.51062	0.767915	-2.51856
H49	-6.54772	0.256163	-1.5142	H49	-6.47849	1.363797	-0.68791
H50	-3.59978	-1.09725	-4.34043	H50	-4.30176	0.042447	-4.15664
H51	-5.95071	-0.48618	-3.80787	H51	-6.36559	1.008494	-3.144
H52	0.645051	0.162578	1.066484	H52	0.40047	0.499997	1.018
H53	2.029701	-0.07706	-0.56749	H53	1.741568	1.732302	-2.53119
H54	-1.0371	-0.41977	2.928618	H54	-1.59116	-1.92159	2.564402
H55	-1.84619	-0.90565	-2.60519	H55	-2.38266	-0.52378	-2.73059
H56	2.291223	-4.89514	-1.04279	H56	2.704944	-4.67379	-0.61167
C57	2.925968	-1.86614	-4.72741	C57	4.652468	-1.18439	-3.17684
C58	2.016611	-2.85214	-4.34397	C58	3.587322	-2.06522	-3.3792
C59	1.821273	-3.13479	-2.99424	C59	2.850208	-2.52866	-2.29296
C60	2.529178	-2.44322	-2.00169	C60	3.158877	-2.12842	-0.9837
C61	3.432877	-1.45123	-2.39764	C61	4.226327	-1.24425	-0.79198
C62	3.632812	-1.16865	-3.74923	C62	4.968436	-0.7775	-1.88071
H63	3.079416	-1.64383	-5.77936	H63	5.231688	-0.82208	-4.02158
H64	1.455038	-3.39893	-5.09574	H64	3.327569	-2.38864	-4.38331
H65	1.089616	-3.89076	-2.71921	H65	2.010625	-3.19252	-2.47653
H66	3.98487	-0.89125	-1.6546	H66	4.480217	-0.90707	0.206828
H67	4.349505	-0.4027	-4.03442	H67	5.796261	-0.09496	-1.70967
C68	2.465488	2.507405	-0.24266	C68	1.096119	3.716227	-2.94917

C69	1.24798	1.918139	-0.97523
C70	-0.09223	2.12739	-0.51047
H71	-0.15605	2.423168	0.531472
C72	-1.18225	2.76164	-1.31438
C73	-1.67947	2.307169	-2.54349
C74	-1.71754	3.953567	-0.79016
C75	-2.66696	3.018931	-3.22605
H76	-1.34206	1.366479	-2.95007
C77	-2.69767	4.669481	-1.47441
H78	-1.359	4.319016	0.168784
C79	-3.17889	4.205291	-2.70081
H80	-3.0477	2.627984	-4.16532
H81	-3.08882	5.588119	-1.04591
H82	-3.94928	4.755927	-3.2323
C83	1.720448	3.081158	-3.23612
C84	0.956708	0.892903	-3.18789
C85	1.785861	2.433048	-4.63325
H86	2.665167	3.551617	-2.9552
H87	0.907032	3.80994	-3.14045
H88	1.293496	3.015814	-5.41215
H89	2.812113	2.200903	-4.93325
N90	1.439524	1.897542	-2.41911
O91	1.070252	1.176013	-4.49251
O92	0.433237	-0.15187	-2.77618
H93	2.334705	-4.55032	0.709856
N94	-1.94463	-1.91462	0.849378
C95	-1.51783	-3.02952	-0.02075
H96	-1.88903	-2.92365	-1.05365
C97	2.345648	2.526671	1.286042
H98	3.217197	3.034777	1.709588
H99	1.456433	3.056842	1.637901
H100	2.321386	1.507047	1.680008
C101	3.795623	1.857814	-0.65106
H102	3.876284	1.718789	-1.73315
H103	4.634022	2.480297	-0.32331
H104	3.900941	0.878071	-0.17477
H105	2.487743	3.558994	-0.57544

C3_ZR_Chelbot_TS1

C1	-4.25737	-3.20512	0.941546
C2	-2.16515	-4.42446	0.664067
C3	-3.63754	-4.42084	0.177519
C4	-3.0567	-2.6892	1.769711
H5	-5.06679	-3.51558	1.610456
H6	-3.71546	-4.31998	-0.90975

C69	0.784658	2.269143	-2.47878
C70	0.282515	2.115589	-1.01348
H71	1.120146	2.50071	-0.42457
C72	-0.89232	2.972601	-0.63121
C73	-2.09278	3.023884	-1.35195
C74	-0.7834	3.803626	0.499431
C75	-3.13428	3.871763	-0.97255
H76	-2.23762	2.3676	-2.19763
C77	-1.81709	4.655448	0.882858
H78	0.132241	3.776108	1.085686
C79	-3.0015	4.696713	0.143579
H80	-4.05472	3.876092	-1.54967
H81	-1.69577	5.290324	1.756429
H82	-3.80896	5.361862	0.435384
C83	-0.90954	2.188204	-4.54136
C84	-0.22711	0.251538	-3.49323
C85	-1.16733	0.964369	-5.43454
H86	-0.39264	2.981787	-5.07809
H87	-1.83183	2.599505	-4.12041
H88	-2.1927	0.897763	-5.79991
H89	-0.46981	0.904613	-6.27589
N90	-0.07643	1.587629	-3.48821
O91	-0.92393	-0.17411	-4.56839
O92	0.160346	-0.57302	-2.64768
H93	2.043936	-4.6438	1.05041
N94	-2.05932	-2.1208	0.037119
C95	-1.31193	-2.9743	-0.91562
H96	-1.27868	-2.55232	-1.92929
C97	1.8095	4.539395	-1.86541
H98	2.129699	5.498464	-2.28432
H99	1.165163	4.749512	-1.011
H100	2.708296	4.022617	-1.5068
C101	1.987428	3.694447	-4.20726
H102	1.635111	3.0129	-4.987
H103	2.066199	4.694221	-4.6451
H104	3.000023	3.37071	-3.93912
H105	0.144199	4.217668	-3.16782
H106	2.015286	0.395229	-0.12616
H107	2.053062	0.219239	-0.94206

C3_ZR_Chelbot_TS3

C1	-4.21821	-3.17512	-0.93054
C2	-2.04941	-4.28667	-1.09864
C3	-3.35532	-4.04691	-1.90187
C4	-3.25904	-2.98074	0.265129

H7	-3.3129	-2.01096	2.582884
H8	-4.65643	-2.44209	0.272758
H9	-1.62062	-5.34793	0.470834
H10	-4.12492	-5.35967	0.455664
C11	-2.3281	-3.9935	2.137783
H12	-1.38441	-3.81974	2.663165
H13	-2.94906	-4.68675	2.712257
C14	0.026798	-3.1879	-0.01965
N15	0.801081	-2.1729	-0.19728
C16	2.197439	-2.68214	-0.46835
C17	2.033372	-4.19587	-0.12132
P18	-1.93497	-0.51394	0.240788
Ir19	0.258475	-0.07233	-0.37705
C20	-2.40698	0.607713	1.594656
C21	-2.79473	2.475528	3.646149
C22	-1.98906	0.319317	2.903142
C23	-2.99303	1.851853	1.316486
C24	-3.18639	2.778056	2.341953
C25	-2.19296	1.245478	3.924055
H26	-3.27943	2.112503	0.30332
H27	-3.63868	3.739113	2.115024
H28	-1.87581	1.010421	4.936043
H29	-2.94888	3.197717	4.442678
O30	0.596865	-4.4001	-0.02778
C31	3.239056	-2.08122	0.470459
C32	2.868406	-1.49583	1.686712
C33	4.604169	-2.23127	0.187076
C34	3.83706	-1.01513	2.569233
H35	1.819095	-1.40269	1.938961
C36	5.572462	-1.75368	1.0708
H37	4.915924	-2.7155	-0.73346
C38	5.193181	-1.1294	2.260267
H39	3.526928	-0.54918	3.500409
H40	6.624166	-1.87045	0.825668
H41	5.946393	-0.74999	2.944195
C42	-3.24447	-0.40871	-1.03571
C43	-4.57276	-0.06002	-0.74178
C44	-2.91247	-0.8109	-2.3398
C45	-5.54441	-0.09161	-1.74061
H46	-4.85128	0.237681	0.264261
C47	-3.89233	-0.85292	-3.33245
C48	-5.20644	-0.48812	-3.03645
H49	-6.56637	0.192606	-1.50606
H50	-3.62472	-1.15842	-4.33994
H51	-5.96648	-0.5106	-3.81216

H5	-5.11768	-3.70714	-0.60266
H6	-3.1615	-3.54818	-2.85721
H7	-3.71957	-2.58887	1.172405
H8	-4.53367	-2.22718	-1.366
H9	-1.44348	-5.12371	-1.44501
H10	-3.8405	-5.00135	-2.12458
C11	-2.57671	-4.35688	0.352688
H12	-1.78688	-4.40336	1.108745
H13	-3.28185	-5.17798	0.509525
C14	0.144111	-3.04461	-0.55803
N15	0.871926	-2.04637	-0.19501
C16	2.226116	-2.56166	0.22966
C17	1.996385	-4.11063	0.137179
P18	-1.90974	-0.46773	0.011619
Ir19	0.454693	0.04955	-0.54822
C20	-2.11103	-0.11136	1.794885
C21	-2.10907	0.533643	4.521472
C22	-1.95877	-1.11663	2.757623
C23	-2.24926	1.223978	2.206708
C24	-2.25015	1.540685	3.563277
C25	-1.96312	-0.79354	4.116114
H26	-2.34572	2.018284	1.474655
H27	-2.35568	2.577593	3.870036
H28	-1.84738	-1.58076	4.855955
H29	-2.10879	0.783551	5.57871
O30	0.685769	-4.26144	-0.47481
C31	2.535408	-2.21277	1.68208
C32	1.558459	-1.70073	2.53986
C33	3.79377	-2.53756	2.211125
C34	1.842999	-1.47187	3.886172
H35	0.570031	-1.47861	2.163541
C36	4.076495	-2.31586	3.558023
H37	4.560371	-2.95831	1.565734
C38	3.103518	-1.77299	4.39974
H39	1.068529	-1.06001	4.527265
H40	5.058968	-2.56626	3.948349
H41	3.327463	-1.59434	5.447508
C42	-3.43373	0.20279	-0.78308
C43	-4.52733	0.717987	-0.07919
C44	-3.52165	0.081659	-2.1802
C45	-5.672	1.137746	-0.7622
H46	-4.49372	0.803494	1.001341
C47	-4.66253	0.501895	-2.85983
C48	-5.74228	1.041792	-2.15217
H49	-6.51116	1.540105	-0.20152

H52	0.440278	0.084173	1.15842
H53	1.86831	0.317359	-0.59038
H54	-1.48816	-0.62062	3.11586
H55	-1.88336	-1.0529	-2.58854
H56	2.427902	-4.86776	-0.88403
C57	2.646714	-1.93252	-4.72961
C58	1.828242	-2.96208	-4.26297
C59	1.729582	-3.21262	-2.89671
C60	2.44214	-2.44207	-1.96579
C61	3.250958	-1.40684	-2.44542
C62	3.359358	-1.15984	-3.81522
H63	2.7241	-1.73536	-5.79478
H64	1.262636	-3.56919	-4.9638
H65	1.068933	-4.00697	-2.55841
H66	3.801898	-0.78563	-1.7538
H67	4.005807	-0.35811	-4.1637
C68	2.636455	2.404166	-0.24224
C69	1.403406	1.777333	-0.92357
C70	0.04975	2.0483	-0.41573
H71	0.051917	2.388221	0.616187
C72	-0.94932	2.844561	-1.20218
C73	-1.67241	2.371555	-2.30493
C74	-1.18439	4.165213	-0.77771
C75	-2.59381	3.188057	-2.96344
H76	-1.55306	1.348178	-2.62764
C77	-2.10011	4.983943	-1.43506
H78	-0.6468	4.547701	0.086935
C79	-2.8108	4.497206	-2.53482
H80	-3.15444	2.786827	-3.80329
H81	-2.26417	5.999429	-1.08482
H82	-3.53256	5.129998	-3.04338
C83	1.814573	2.950593	-3.17802
C84	0.852302	0.841652	-3.12521
C85	1.688353	2.357505	-4.59428
H86	2.815866	3.339299	-2.98163
H87	1.072394	3.731576	-2.97612
H88	1.178002	3.013168	-5.30016
H89	2.655878	2.049462	-5.00165
N90	1.526683	1.756236	-2.37603
O91	0.878655	1.164276	-4.42359
O92	0.265918	-0.16019	-2.70061
H93	2.464844	-4.43919	0.851179
N94	-2.01016	-2.0818	0.896864
C95	-1.48334	-3.17327	0.044746
H96	-1.8062	-3.06981	-1.00516

H50	-4.71835	0.395272	-3.94027
H51	-6.63332	1.370401	-2.67919
H52	0.552303	0.305987	0.983879
H53	1.679679	1.687643	-2.7629
H54	-1.8147	-2.14695	2.449804
H55	-2.70771	-0.36344	-2.7414
H56	2.726918	-4.62508	-0.48802
C57	4.977717	-1.12396	-2.80688
C58	4.004234	-2.07769	-3.11107
C59	3.152354	-2.5462	-2.11466
C60	3.255556	-2.07561	-0.79839
C61	4.22422	-1.10845	-0.50632
C62	5.083358	-0.64131	-1.50318
H63	5.648752	-0.76185	-3.5807
H64	3.907707	-2.45608	-4.1247
H65	2.383864	-3.26901	-2.37668
H66	4.303286	-0.70481	0.496472
H67	5.834712	0.103466	-1.25572
C68	1.039768	3.673031	-3.2634
C69	0.747901	2.260826	-2.67778
C70	0.395602	2.242903	-1.16787
H71	1.260483	2.778225	-0.75432
C72	-0.7762	3.03963	-0.66912
C73	-2.04293	3.010194	-1.26079
C74	-0.58329	3.895835	0.429095
C75	-3.08025	3.81288	-0.78578
H76	-2.23755	2.328797	-2.07415
C77	-1.61664	4.69502	0.913426
H78	0.391565	3.933043	0.910699
C79	-2.87337	4.659738	0.303576
H80	-4.05549	3.757743	-1.26027
H81	-1.439	5.349374	1.762585
H82	-3.68052	5.284499	0.675284
C83	-1.17082	2.129644	-4.51806
C84	-0.3312	0.214372	-3.52983
C85	-1.52262	0.882243	-5.34711
H86	-0.71943	2.907113	-5.13298
H87	-2.04173	2.554141	-4.00933
H88	-2.58539	0.796242	-5.57621
H89	-0.93868	0.815159	-6.27021
N90	-0.21973	1.556713	-3.55384
O91	-1.15234	-0.23728	-4.50137
O92	0.197393	-0.58005	-2.74061
H93	1.952306	-4.57746	1.122408
N94	-2.09074	-2.14402	-0.13494

C97	2.543235	2.408693	1.28839
H98	3.458512	2.838192	1.705893
H99	1.703017	2.999955	1.65984
H100	2.44498	1.386924	1.665381
C101	3.959994	1.762522	-0.68229
H102	4.022308	1.634758	-1.76704
H103	4.803351	2.382578	-0.36436
H104	4.080044	0.77977	-0.21293
H105	2.621023	3.452434	-0.58052
X	0	0	0

C3_ZR_Chelbot_INT1

C1	-4.23933	-3.19229	0.91985
C2	-2.16468	-4.43755	0.641566
C3	-3.63943	-4.42341	0.164499
C4	-3.03039	-2.68661	1.742571
H5	-5.05267	-3.48444	1.592062
H6	-3.72271	-4.33334	-0.92322
H7	-3.27931	-2.00419	2.553573
H8	-4.62666	-2.42831	0.244808
H9	-1.62824	-5.36659	0.450766
H10	-4.13595	-5.35324	0.456259
C11	-2.31317	-3.9957	2.11338
H12	-1.36462	-3.82758	2.631808
H13	-2.93778	-4.67874	2.696137
C14	0.035695	-3.23848	-0.07425
N15	0.804372	-2.2197	-0.22512
C16	2.199635	-2.70115	-0.50777
C17	2.038988	-4.23977	-0.26109
P18	-1.88672	-0.51556	0.223606
Ir19	0.208571	-0.1153	-0.33082
C20	-2.44956	0.61371	1.52999
C21	-2.95049	2.529793	3.507231
C22	-2.10456	0.357168	2.865851
C23	-3.01412	1.851881	1.186159
C24	-3.26503	2.802092	2.175773
C25	-2.36685	1.307219	3.850134
H26	-3.23346	2.090952	0.151195
H27	-3.69703	3.759378	1.899225
H28	-2.10588	1.098896	4.88366
H29	-3.14694	3.272217	4.275355
O30	0.603607	-4.45178	-0.14037
C31	3.219542	-2.14032	0.481007
C32	2.826242	-1.53009	1.677327
C33	4.588608	-2.33483	0.243158

C95	-1.27158	-2.94799	-1.07346
H96	-1.21088	-2.5	-2.07307
C97	1.851326	4.563928	-2.30989
H98	2.131374	5.488303	-2.82456
H99	1.285708	4.845583	-1.41888
H100	2.780693	4.07165	-1.99783
C101	1.807454	3.544827	-4.59325
H102	1.360359	2.828352	-5.28879
H103	1.869291	4.512615	-5.1001
H104	2.831701	3.205036	-4.40073
H105	0.078188	4.175526	-3.43089
H106	2.032027	0.121197	-0.7808
H107	1.298462	1.321552	-0.23247

C3_ZR_Chelbot_PROD

C1	-3.99134	-3.54621	-1.33026
C2	-1.73604	-4.48176	-1.32272
C3	-2.97647	-4.31617	-2.23979
C4	-3.18068	-3.35633	-0.02853
H5	-4.88974	-4.14067	-1.13331
H6	-2.73711	-3.7761	-3.16145
H7	-3.7664	-3.05314	0.839581
H8	-4.30949	-2.59064	-1.74923
H9	-1.02682	-5.24493	-1.64293
H10	-3.36239	-5.2982	-2.52774
C11	-2.39438	-4.67637	0.062637
H12	-1.67961	-4.69983	0.89091
H13	-3.03558	-5.56141	0.106135
C14	0.291581	-3.06843	-0.56376
N15	0.945595	-2.01167	-0.20298
C16	2.332989	-2.42151	0.260229
C17	2.161978	-3.97397	0.291795
P18	-1.92075	-0.77208	0.118369
Ir19	0.333539	-0.08013	-0.47957
C20	-2.1105	-0.62058	1.930281
C21	-2.14445	-0.26058	4.711994
C22	-2.18217	-1.72664	2.784985
C23	-2.0294	0.669285	2.483886
C24	-2.05476	0.847568	3.864429
C25	-2.20118	-1.54457	4.170565
H26	-1.92804	1.534962	1.833762
H27	-1.99215	1.849252	4.28011
H28	-2.25492	-2.40984	4.825172
H29	-2.15889	-0.12172	5.7892
O30	0.910885	-4.2378	-0.40569

C34	3.781917	-1.07018	2.587292	C31	2.623803	-1.96193	1.685595
H35	1.77328	-1.40601	1.897237	C32	1.57535	-1.6764	2.568752
C36	5.541296	-1.88296	1.155359	C33	3.933915	-1.9893	2.180517
H37	4.913466	-2.82945	-0.66799	C34	1.83522	-1.36173	3.901231
C38	5.142222	-1.2349	2.326809	H35	0.550121	-1.68813	2.220784
H39	3.45615	-0.58707	3.504339	C36	4.193266	-1.68187	3.516459
H40	6.596569	-2.03553	0.947828	H37	4.758276	-2.2468	1.523363
H41	5.884451	-0.87507	3.033118	C38	3.146483	-1.35471	4.378354
C42	-3.1109	-0.43864	-1.14043	H39	1.005239	-1.12321	4.560472
C43	-4.45739	-0.11283	-0.90371	H40	5.216766	-1.69785	3.880155
C44	-2.71855	-0.83709	-2.42916	H41	3.351584	-1.1062	5.415706
C45	-5.3854	-0.1633	-1.94251	C42	-3.42831	0.021433	-0.58895
H46	-4.78507	0.183131	0.087578	C43	-4.38016	0.734165	0.150913
C47	-3.65541	-0.8937	-3.46174	C44	-3.57191	-0.05625	-1.98544
C48	-4.98712	-0.55296	-3.22287	C45	-5.43996	1.37692	-0.49661
H49	-6.42086	0.103158	-1.75056	H46	-4.30014	0.79741	1.230978
H50	-3.33885	-1.19472	-4.45633	C47	-4.62928	0.584017	-2.62758
H51	-5.713	-0.58912	-4.0301	C48	-5.56364	1.31437	-1.8851
H52	0.33306	0.011689	1.218594	H49	-6.17158	1.924796	0.090945
H53	2.07818	0.822325	-0.78257	H50	-4.73189	0.502498	-3.70643
H54	-1.60763	-0.57193	3.129201	H51	-6.38805	1.815833	-2.38363
H55	-1.67867	-1.0629	-2.63848	H52	0.639647	0.251027	1.002766
H56	2.409456	-4.85871	-1.07898	H53	1.512485	1.472658	-2.55182
C57	2.776642	-1.65677	-4.69703	H54	-2.19433	-2.73193	2.378809
C58	1.9412	-2.71292	-4.32605	H55	-2.86002	-0.62562	-2.57253
C59	1.797564	-3.05569	-2.98395	H56	2.960265	-4.51435	-0.21635
C60	2.483593	-2.35656	-1.97995	C57	4.937644	-1.06502	-2.93959
C61	3.310043	-1.29458	-2.36193	C58	4.129558	-2.19106	-3.10355
C62	3.461828	-0.95275	-3.70812	C59	3.328035	-2.63284	-2.0526
H63	2.890478	-1.39009	-5.74357	C60	3.31831	-1.96333	-0.82127
H64	1.396407	-3.26815	-5.08405	C61	4.116745	-0.82268	-0.67512
H65	1.12255	-3.86598	-2.71945	C62	4.926774	-0.3832	-1.72279
H66	3.843897	-0.72484	-1.61186	H63	5.57	-0.72299	-3.75422
H67	4.119865	-0.13051	-3.97717	H64	4.121972	-2.72656	-4.04868
C68	2.806322	2.784248	-0.59527	H65	2.688167	-3.49688	-2.21352
C69	1.663286	1.847357	-1.06117	H66	4.09771	-0.26551	0.253682
C70	0.266099	1.975282	-0.40227	H67	5.54809	0.497628	-1.58558
H71	0.383889	2.329021	0.622428	C68	1.467396	3.498842	-3.20628
C72	-0.70713	2.894172	-1.09493	C69	0.770785	2.275493	-2.57005
C73	-1.54045	2.49056	-2.14944	C70	0.300309	2.475579	-1.10183
C74	-0.78622	4.235682	-0.68102	H71	1.163351	2.611197	-0.45348
C75	-2.40844	3.389112	-2.77283	C72	-0.723	3.570935	-0.87106
H76	-1.53549	1.457843	-2.46948	C73	-2.09269	3.3045	-0.98899
C77	-1.65106	5.138394	-1.30095	C74	-0.31137	4.871754	-0.55349
H78	-0.16249	4.57261	0.144264	C75	-3.03334	4.319689	-0.81939

C79	-2.4666	4.719773	-2.35539
H80	-3.0479	3.040087	-3.57938
H81	-1.69028	6.169093	-0.95853
H82	-3.14322	5.419645	-2.83716
C83	1.665454	2.975128	-3.41518
C84	0.838254	0.839741	-3.1359
C85	1.395239	2.302421	-4.77273
H86	2.653252	3.43726	-3.37564
H87	0.905237	3.718551	-3.15387
H88	0.772574	2.896591	-5.44178
H89	2.318584	2.008474	-5.28137
N90	1.568928	1.80769	-2.53055
O91	0.666246	1.090574	-4.43992
O92	0.351514	-0.16335	-2.5946
H93	2.498858	-4.54959	0.6788
N94	-1.98268	-2.09191	0.859294
C95	-1.47365	-3.19896	0.009547
H96	-1.80595	-3.09347	-1.03729
C97	2.910491	2.818103	0.936414
H98	3.799162	3.382356	1.23436
H99	2.044626	3.291226	1.405032
H100	3.006251	1.803011	1.341203
C101	4.156678	2.349424	-1.19196
H102	4.117726	2.199149	-2.27456
H103	4.927471	3.096919	-0.98149
H104	4.483128	1.405018	-0.7393
H105	2.554848	3.794708	-0.94768

C3_ZR_Chelbot_TS2

C1	-4.20889	-3.12099	-0.45781
C2	-2.10398	-4.32199	-0.75642
C3	-3.46948	-4.0765	-1.45166
C4	-3.14849	-2.91973	0.648492
H5	-5.10037	-3.59331	-0.03187
H6	-3.35074	-3.63722	-2.44748
H7	-3.52047	-2.48004	1.573576
H8	-4.51946	-2.1808	-0.91349
H9	-1.5519	-5.18988	-1.11685
H10	-4.00487	-5.02223	-1.57324
C11	-2.50487	-4.31455	0.735745
H12	-1.65604	-4.35664	1.425054
H13	-3.22103	-5.10206	0.986596
C14	0.167897	-3.1041	-0.50403
N15	0.897243	-2.08954	-0.20541
C16	2.282591	-2.56733	0.097778

H76	-2.42789	2.296241	-1.20717
C77	-1.25112	5.890238	-0.39174
H78	0.743735	5.093433	-0.42716
C79	-2.61348	5.618255	-0.52743
H80	-4.0902	4.086523	-0.90826
H81	-0.91682	6.895188	-0.15029
H82	-3.34281	6.411961	-0.39433
C83	-1.30862	2.517154	-4.18624
C84	-0.5824	0.434411	-3.47316
C85	-1.82441	1.407239	-5.11703
H86	-0.85292	3.341977	-4.73533
H87	-2.09209	2.917636	-3.53665
H88	-2.90171	1.440895	-5.28079
H89	-1.30136	1.387806	-6.07833
N90	-0.32008	1.76041	-3.40947
O91	-1.51684	0.173423	-4.41296
O92	-0.09676	-0.46804	-2.78078
H93	2.047658	-4.3456	1.311009
N94	-2.06095	-2.40592	-0.27029
C95	-1.09204	-3.08081	-1.17018
H96	-1.00652	-2.57196	-2.13756
C97	2.691787	3.911714	-2.37023
H98	3.219997	4.734543	-2.86075
H99	2.435086	4.244929	-1.36277
H100	3.393293	3.073802	-2.27682
C101	1.941228	3.169391	-4.6343
H102	1.132958	2.863775	-5.30351
H103	2.427895	4.041086	-5.08155
H104	2.672459	2.352965	-4.61289
H105	0.761002	4.339507	-3.23558
H106	1.870265	0.27823	-0.71043
H107	-0.3393	1.577574	-0.74075

C17	2.141688	-4.11496	-0.16697
P18	-1.76925	-0.46762	0.162986
Ir19	0.270487	0.016136	-0.56511
C20	-1.99038	-0.00409	1.910211
C21	-2.03481	0.78258	4.594724
C22	-1.73025	-0.93887	2.922789
C23	-2.25248	1.332272	2.249153
C24	-2.27872	1.718582	3.587382
C25	-1.75874	-0.54451	4.260562
H26	-2.41511	2.075462	1.477666
H27	-2.48309	2.754758	3.840775
H28	-1.56162	-1.27467	5.040322
H29	-2.05384	1.087934	5.636978
O30	0.742211	-4.3078	-0.51606
C31	2.637079	-2.32664	1.565644
C32	1.772667	-1.65921	2.437713
C33	3.825306	-2.86739	2.081412
C34	2.103501	-1.50024	3.784645
H35	0.838606	-1.26041	2.069817
C36	4.157187	-2.70748	3.425119
H37	4.50058	-3.41457	1.428297
C38	3.299087	-2.01502	4.281963
H39	1.414338	-0.97378	4.439588
H40	5.085284	-3.12664	3.802666
H41	3.558475	-1.88942	5.329078
C42	-3.21604	0.149191	-0.76931
C43	-4.35307	0.698841	-0.16601
C44	-3.23966	-0.13806	-2.14483
C45	-5.4817	0.989378	-0.93542
H46	-4.3728	0.898673	0.898684
C47	-4.36484	0.155491	-2.90872
C48	-5.48944	0.727952	-2.30524
H49	-6.35702	1.420363	-0.45828
H50	-4.36881	-0.0755	-3.9705
H51	-6.37072	0.955871	-2.89787
H52	0.506534	0.478395	0.902394
H53	1.710037	1.589955	-2.53459
H54	-1.48562	-1.96425	2.666476
H55	-2.38691	-0.60952	-2.61639
H56	2.746324	-4.46048	-1.00777
C57	4.857778	-0.75288	-2.90473
C58	3.661022	-1.37934	-3.25823
C59	2.865116	-1.96667	-2.27748
C60	3.241758	-1.93305	-0.92523
C61	4.43959	-1.30007	-0.5809

C62	5.243615	-0.71936	-1.56488
H63	5.483401	-0.29608	-3.66625
H64	3.344274	-1.41114	-4.29693
H65	1.92336	-2.42025	-2.56808
H66	4.742775	-1.2303	0.45688
H67	6.169677	-0.23022	-1.27658
C68	1.252289	3.624233	-2.96111
C69	0.798328	2.205177	-2.52408
C70	0.19877	2.078314	-1.09327
H71	1.01039	2.436468	-0.45151
C72	-0.96827	2.978197	-0.78647
C73	-2.1548	3.005133	-1.53346
C74	-0.87646	3.860325	0.306133
C75	-3.19501	3.879935	-1.21824
H76	-2.29389	2.301844	-2.34227
C77	-1.91347	4.733877	0.63054
H78	0.024989	3.853163	0.91467
C79	-3.08057	4.752087	-0.13601
H80	-4.10413	3.863717	-1.81298
H81	-1.80674	5.405149	1.478548
H82	-3.88932	5.434266	0.109593
C83	-0.84306	2.29292	-4.61829
C84	-0.3063	0.287301	-3.59884
C85	-1.27968	1.097398	-5.48646
H86	-0.23974	3.002965	-5.18356
H87	-1.68844	2.832203	-4.18187
H88	-2.33044	1.131039	-5.77701
H89	-0.65535	0.976095	-6.37662
N90	-0.06213	1.607128	-3.57949
O91	-1.084	-0.06732	-4.64158
O92	0.078425	-0.57131	-2.78415
H93	2.347715	-4.71606	0.71895
N94	-1.9804	-2.14594	0.126764
C95	-1.2918	-3.00803	-0.86222
H96	-1.33228	-2.59979	-1.88146
C97	1.977854	4.37744	-1.83597
H98	2.395184	5.310049	-2.2282
H99	1.310213	4.634868	-1.01275
H100	2.811512	3.78615	-1.43867
C101	2.188216	3.536146	-4.18202
H102	1.799453	2.905834	-4.98741
H103	2.380186	4.530503	-4.59647
H104	3.152754	3.110031	-3.88171
H105	0.355333	4.203061	-3.21728
H106	2.609021	0.994144	-0.01636

H107 2.958095 0.80448 -0.64938

13. References

[1] Massaro, L.; Yang, J.; Krajangsri, S.; Silvi, E.; Singh, T.; Andersson, P. G. *J. Org. Chem*. **2019**, *84*, 21, 13540-13548

[2] Adam, W.; Bosio, S. G.; Turro, N. J. *J. Am. Chem. Soc*, **2002**, *124*(47),14004-14005.

[3] Gourdet, B; Lam, H. W. *J. Am. Chem. Soc*, **2009**, *131*(11), 3802-3803.

[4] Song, P.; Yu, P.; Lin, J. S.; Li, Y.; Yang, N. Y.; Liu, X. Y. *Org. lett.*, **2017**,*19*(6), 1330-1333.

[5] Tischler, A. N.; Tischler, M. H. *Tetrahedron Lett.*, **1978**, *19(37)*, 3407-3410.

[6] Terada, M.; Soga, K.; Momiyama, N. *Angew. Chem. Int. Ed*, **2008***,* *47*(22), 4122-4125.

[7] Chang, L.; Kuang, Y.; Qin, B.; Zhou, X.; Liu, X.; Lin, L.; Feng, X. *Org. lett.*, **2010**, *12*(10), 2214-2217.

[8] Kim, M. J.; Kim, W. H.; Han, K.; Choi, Y. K.; Park, J. *Org. lett.*, **2007**, *9*(6), 1157-1159.

[9] Kotani, E.; Kobayashi, S.; Ishii, Y.; Tobinaga, S., *Chem. Pharm. Bull.*, **1984**, *32(11)*, 4281-4291.

[10] Roe, C.; Hobbs, H.; Stockman, R. A. *J. Org. Chem*. **2011**. *76*(22), 9452-9459.

[11] Dai, X.; Nakai, T.; Romero, J. A.; Fu, G. C. *Angew. Chem*., *Int. Ed*, 2**007**, *119*(23), 4445-4447.

[12] Kameyama, M.; Kamigata, N. *Bull. Chem. Soc. Jpn*, **1987**, *60*(10), 3687-3691.

[13]Jaguar, version 7.9, Schrodinger, LLC, New York, NY, 2011.

[14] (a) Becke, A. D.; *J. Chem. Phys.* 1993, **98**, 5648–5652. (b) Lee, C.; Yang, W.; Parr, R. G. *Phys. Rev. B.* 1988, **37**, 785–789.

[15] Grimme, S.; Antony, J.; Ehrlich, S.; Krieg, H. *J. Chem. Phys.* 2010, **132**, 154104.

[16] Hay, P. J.; Wadt, W. R. *J. Chem. Phys.* 1985, **82**, 270–283.

[17] (a) Goodman, J. M.; Silva, M. A. *Tetrahedron Lett*. 2003, **44**, 8233-8236. (b) Goodman, J. M.; Silva, M. A. *Tetrahedron Lett*. 2005, **46**, 2067-2069.
